# Bifunctional Iminophosphorane Superbases Enable the Highly Enantioselective Sulfa-Michael Addition to Fully Substituted Cyclopropene Carboxylic Acid Derivatives

**DOI:** 10.1021/jacs.5c07849

**Published:** 2025-10-22

**Authors:** Kang Yuan, Alberto I. Ristache, Szymon M. Kosc, Agamemnon Crumpton, Darren J. Dixon

**Affiliations:** Department of Chemistry, Chemistry Research Laboratory, 6396 University of Oxford, 12 Mansfield Road, Oxford OX1 3TA, U.K.

## Abstract

The metal-free catalytic enantioselective intermolecular conjugate addition of alkyl thiols to unactivated β-substituted cyclopropene carboxylic acid derivatives has been developed. High enantiomeric excesses and yields were consistently achieved across a broad range of thiol pronucleophiles and fully substituted cyclopropene electrophiles under mild reaction conditions, enabled by a novel *tert*-leucine-derived amide-containing bifunctional iminophosphorane (BIMP) catalyst. Additionally, lowering the catalyst loading to 5.0 mol % was possible and allowed the reaction to be carried out on a gram scale.

## Graphical abstract

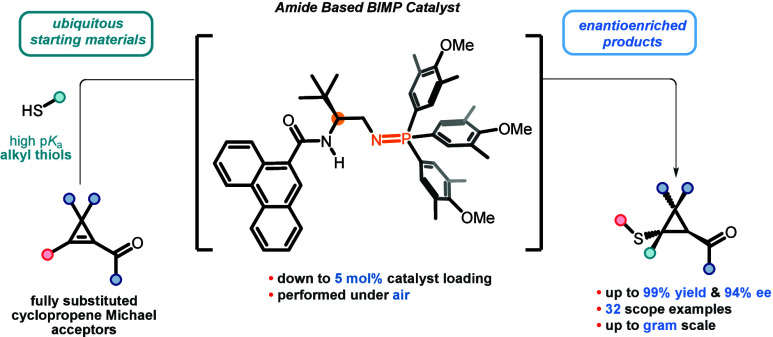

Bifunctional iminophosphorane (BIMP) superbases have recently emerged as synthetically powerful metal-free catalyst systems for asymmetric conjugate additions.^1^ These catalysts, featuring a strongly basic iminophosphorane moiety and a hydrogen-bond-donor group, enable dual activation of both nucleophiles and electrophiles, providing mutually enhanced reactivity and selectivity across a wide range of reactions.^1^ BIMP catalysis has proven effective for enantioselective sulfa-Michael addition (SMA) to unactivated α,β-unsaturated esters and amides, achieving high yields and excellent stereocontrol (Figure 1).^2^

**Figure 1:**
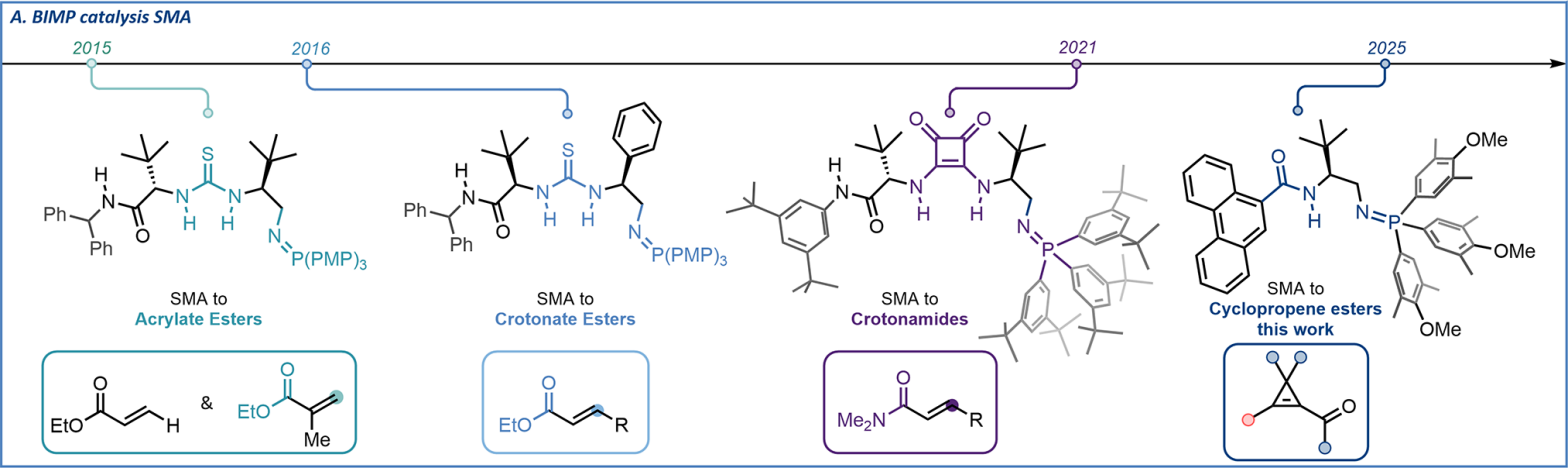
(left, middle) Previous BIMP catalysts for SMAs to unsaturated carboxylic acid derivatives and (right) this work.

Building on our previous work, in the current study we chose to explore the sulfa-Michael addition to fully substituted cyclopropene carboxylate derivatives, a reaction that would directly afford chiral cyclopropane sulfides in enantioenriched form. Chiral cyclopropanes are widely recognized as privileged structural motifs in pharmaceuticals, natural products, and functional materials.^3^ Their rigid three-dimensional architecture contribute to improved biological properties, including enhanced metabolic stability and selective molecular recognition.^4^ In particular, cyclopropanes bearing carbon–sulfur (C–S) bonds are of growing interest due to the ability of sulfur atoms to adopt various stable oxidation levels, modulate electronic properties, and serve as handles for further derivatization.^5^ Over the past several decades, numerous strategies have been established for the synthesis of cyclopropanes. These include Simmons–Smith cyclopropanation,^6^ transition-metal-catalyzed decomposition of diazo compounds,^7^ Michael-initiated ring closures,^8^ enzymatic carbene transfer reactions,^9^ and hydrofunctionalization of cyclopropenes.^10,11^ These methods have enabled the synthesis of a variety of 1,2-disubstituted, 1,2,3-trisubstituted, and 1,1,2,3-tetrasubstituted chiral cyclopropanes. However, the asymmetric synthesis of quaternary 1,1,2,2-tetrasubstituted cyclopropanes remains a significant challenge due to their high ring strain, steric congestion, and difficulty in simultaneously controlling multiple stereocenters.^7b,10g,12^ Conjugate addition reactions have emerged as attractive alternatives for constructing cyclopropane frameworks, offering excellent atom economy and synthetic efficiency.^13^ In a typical Michael addition, a nucleophile adds to an electron-deficient olefin under redox-neutral conditions, enabling the rapid assembly of complexity.^14^ While enantioselective SMAs have been successfully applied to various α,β-unsaturated carbonyls, their application to the fully substituted cyclopropene scaffold remains underexplored.^15^

Complementary and synthetically powerful enantioselective metal-catalyzed direct hydrofunctionalization reactions of cyclopropenes have been reported.^3b,11c,16^ In fact, impressive enantioselectivities and broad scopes have been achieved in certain cases. However, these approaches are mostly restricted to 1,1′-disubstituted cyclopropene substrates and rely on air- and moisture-sensitive ligand/catalyst systems, and ring-opening reaction pathways can compete. Consequently, approaches for the enantioselective functionalization of fully substituted cyclopropenes remain an attractive and underexplored challenge.

In the present study, a novel amide-containing BIMP catalyst was employed to promote the enantioselective sulfa-Michael addition of alkyl thiols to unactivated α,β-unsaturated cyclopropene esters. This transformation achieves the simultaneous formation of a C–S bond and two contiguous stereocenters, including a fully substituted carbon atom. The reaction proceeds under mild conditions with high yields, excellent enantioselectivities, and diastereoselectivities. Together, this work establishes a practical metal-free strategy for the asymmetric construction of densely functionalized cyclopropanes containing sulfur.

Readily available trimethyl 3-phenylcycloprop-2-ene-1,1,2-tricarboxylate (**1a**), selected for its modest steric demand and electronic neutrality, was chosen as the model substrate for the enantioselective SMA. A preliminary investigation of catalyst performance (10 mol %) was conducted in THF at room temperature in the presence of 2.0 equiv of 1-propanethiol (**2a**) (Table 1). Initial experiments revealed that the commonly employed P1 phosphazene base BEMP was active in the transformation, giving a 73% yield of product **3a** as a 4:1 mixture of diastereomers after 24 h (entry 1). Subsequent studies focused on thiourea-containing BIMP catalysts, including first-generation catalyst **B1** with a single stereocenter and second-generation variant **B2**. These catalysts provided reasonable diastereoselectivies and yields (up to 76%), though the enantioselectivities remained low, with 25% ee obtained for **B1** and 0% ee for **B2** (entries 2 and 3).^17^ To improve the selectivity, squaramide-containing BIMP catalysts, previously reported to be effective in sulfa-Michael additions with unactivated α,β-unsaturated amides, were explored.^2^ Both double-stereocenter (**B3**) and single-stereocenter (**B4**) catalysts were tested and afforded moderate yields (up to 67%). However, the enantioselectivities improved, with 25% ee for **B3** and 40% ee for **B4** (entries 4 and 5). These results indicated that catalysts possessing a single stereocenter, on balance, performed better than those with two.^18^ Attention then shifted to the nature of the hydrogen-bond-donor moiety in the catalyst. The existing data suggested that catalysts with two hydrogen-bond donors might not be ideal for this reaction. Based on this observation and inspired by previous studies on BIMP-mediated Michael additions, amide-containing single-stereocenter BIMP catalysts were considered, given their single hydrogen-bond donor properties. Switching to the amide-containing catalyst **B5** provided **3a** in 87% yield with 50% ee when toluene was used as the solvent. To further enhance the enantioselectivity, a 1-naphthoic acid-derived amide was introduced, leading to the development of BIMP catalyst **B6**.^19^ This modification successfully increased the enantioselectivity to 67% ee while maintaining a high yield of 92% (entry 7). Phenanthrene-9-carboxylic acid-derived amide catalyst **B7** further improved the ee to 78% (entry 8). The late-stage formation of the iminophosphorane moiety allowed for further catalyst optimization through variations in the phosphine component of the Staudinger reaction. This systematic structural tuning highlighted the importance of peripheral and electron-donating groups, ultimately leading to catalyst **B8**, which initially provided 90% ee under the standard conditions. Further optimization of reaction solvent and concentration, reducing it from 1 to 0.1 M, resulted in a 99% yield of isolated product **3a** with 94% ee (entry 9). Additionally, exposure of the reaction mixture to air during the reaction did not affect the transformation, and temperature screening confirmed that room temperature (21 °C) was optimal for the reaction (detailed condition screening is available in the Supporting Information).

**Table 1:**
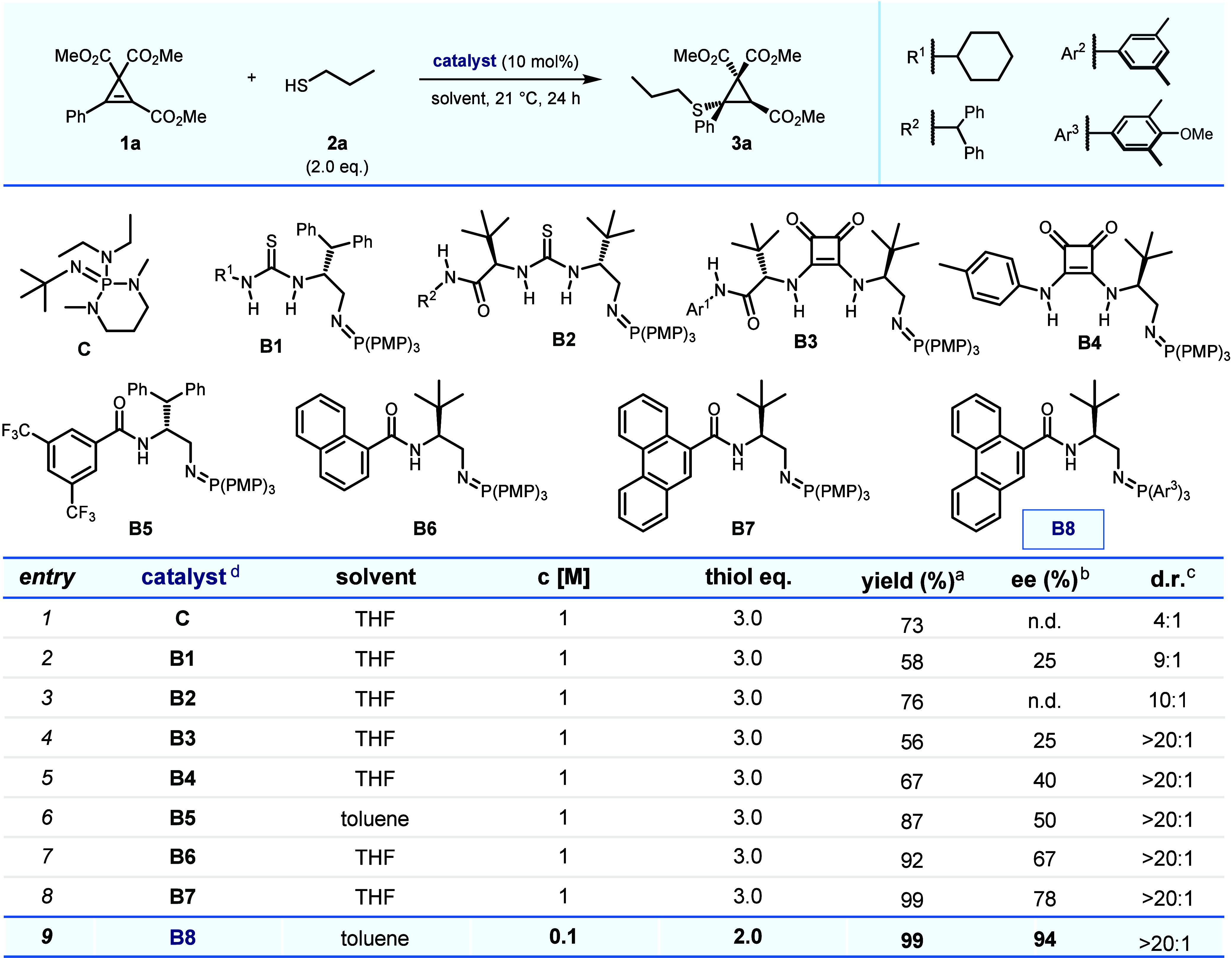
Selected Reaction Optimization (0.1 mmol Scale)

a Isolated yield.

b Enantiomeric excess (ee) of the major diastereomer determined by HPLC on a chiral stationary phase.

c Diastereomeric ratio (dr) determined by ^1^H NMR analysis of crude reaction mixture.

d PMP: *p*-methoxyphenyl.

The scope of the protocol was then explored (Scheme 1). A comprehensive evaluation of the nucleophile scope was first conducted using primary and secondary alkyl thiols, benzyl thiols, and thiophenols. Both primary and secondary alkyl thiols were well-tolerated, affording the corresponding thioethers with high enantioselectivity and reactivity (**3b**–**3f**). Notably, thiols containing a trimethylsilyl (TMS) group and an ester functionality yielded excellent results in terms of yield, enantioselectivity (ee), and diastereoselectivity (dr). However, a decrease in dr (6:1) was observed in product **3d**, which contained a long alkyl chain.^17^ Benzyl thiols underwent the transformation with high reactivity and maintained good enantioselectivity and diastereoselectivity (**3g**–**3k**). Notably, substituents on the aromatic ring did not significantly impact the reactivity or selectivity, except for the difluoro-substituted thiol (**3j**), which exhibited a slight decrease in ee (86%). Additionally, thiophenols (**3l**–**3n**) performed well, although a slight reduction in ee (80–87%) was observed.^20^ Attention then shifted to β-substituents on the enoate backbone (**3o**–**3u**). All variations gave high enantioselectivity and product yields (both exceeding 90%). However, methyl substitution at the ortho and para positions of the aromatic ring resulted in lower dr, while substitution at the meta position yielded excellent reactivity and selectivity (**3o** and **3p**). An electron-donating *tert*-butyl (tBu) group at the para position caused a slight decrease in the dr (15:1) (**3u**). Electron-withdrawing groups, including halogens and trifluoromethyl (CF_3_), provided excellent results (**3r**–**3t**). However, alkyl-substituted enoate backbones were less effective, likely due to competing deconjugation of the double bond.^21^ The effects of substituents on the diester moiety were also examined (**3v** and **3w**). Both variations provided high enantioselectivity and yield, though the benzyl ester resulted in a lower dr (4:1)^22^ Modifications to the enoate ester substituents were explored. Encouragingly, all employed substrates demonstrated excellent reactivity and selectivity (over 90% yield, 90% ee, and 10:1 dr) (**3x**–**3ad**). Functional groups, including ether and alkyne moieties, were well-tolerated under these conditions. A selection of amide-substituted cyclopropenes were also tested in the sulfa-Michael addition. With an *N*-benzyl substituent, the expected addition product **3ae** was obtained in 62% yield with 87% ee, whereas with *N*-Ph, *N*-Cy, and *N*-PMP amides, sulfa-Michael addition was followed by succinimide formation to afford bicyclic products **3af**–**3ah** in good yields (78–81%) with high enantiomeric excesses (90–93%).^23^ Although the substrate scope was broad with respect to the thiol pronucleophile and the aryl group at the β-position, the *gem*-diester substituents as presented in Scheme 1 were important for reactivity and selectivity. Substrates possessing other groups in this position were indeed found to be suboptimal (see the Supporting Information).

**Scheme 1:**
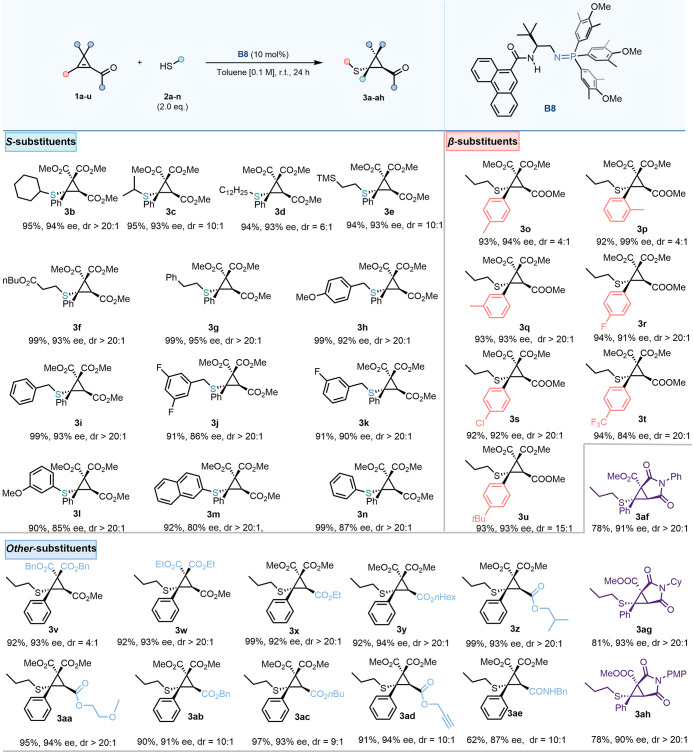
Reaction Scope for the BIMP **B8**-Catalyzed Enantioselective SMA to Fully Substituted Cyclopropene Carboxylates^a^

a Reactions were run on a 0.1 mmol scale and conducted under air with 2.0 equiv of thiol at room temperature in toluene at 0.1 M concentration.

After the scope and limitations of this methodology were established, the scalability of the reaction was evaluated using model substrate **1a** and thiol **2a**. By doubling the reaction concentration and reducing the catalyst loading to 5.0 mol %, a 38-fold scale-up (3.8 mmol) was achieved. Under these conditions, 1.4 g of product **3a** was obtained (99% yield) with 93% ee (Scheme 2). To demonstrate the synthetic utility of **3a**, a series of transformations were then conducted. Oxidation with Oxone provided sulfone **4a** in 87% yield without any loss of optical purity (93% ee).^24^ Oxidation with *m*-CPBA furnished separable sulfoxides **4b** and **4b′** with minimal erosion in enantiopurity in a combined yield of 87% but with essentially no diastereoselectivity. Furthermore, a tributyltin hydride-mediated desulfurization gave product **4c** as an inseparable 3:2 mixture of two diastereomers, each with 93% ee. Finally, in the presence of anisaldehyde and catalytic SnCl_4_, **3a** underwent a fast and efficient ring-opening cycloaddition/desulfurization reaction to give the dihydrofuran product **4d** in 85% yield, albeit as a racemate.^25^

**Scheme 2:**
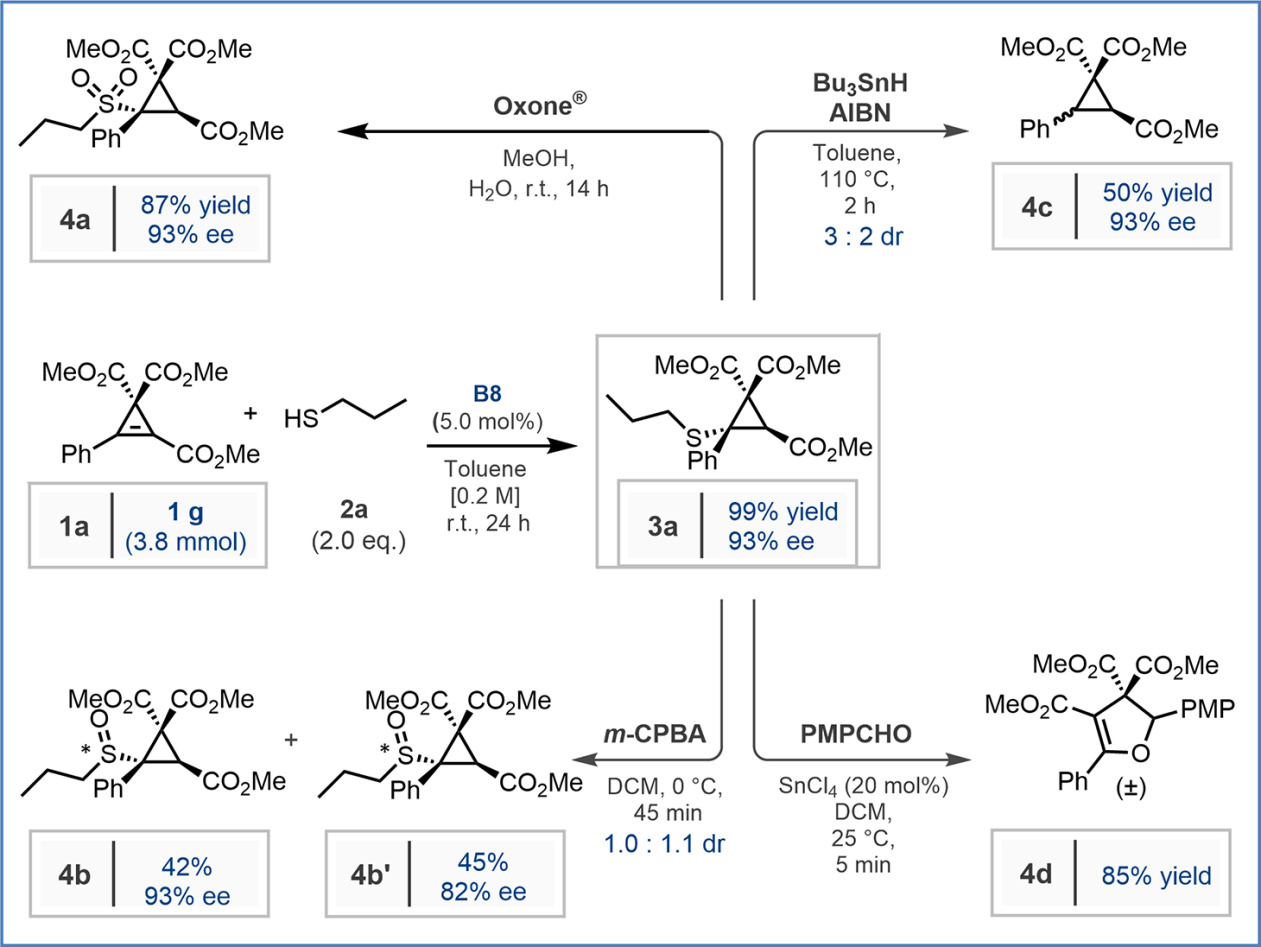
Gram-Scale Enantioselective SMA to Cyclopropene Carboxylate **1a** and Downstream Derivatization of Product **3a**

A metal-free catalytic enantioselective intermolecular sulfa-Michael addition to fully substituted cyclopropene carboxylates has been developed. A thorough investigation of substrate scope established a general methodology capable of delivering a diverse range of SMA products with good functional group tolerance, high yields, high diastereoselectivities, and good to excellent enantioselectivities. Ongoing research continues to focus on the development of new BIMP catalysts and their reactions, and the results will be disclosed in due course.

## Supplementary Material



## Notes

Reaction optimization tables, detailed experimental procedures, and analytical data for all new compounds, including ^1^H and ^13^C NMR spectra, crystallographic data, and HPLC traces (PDF)
