# Supplementary material for: Bifunctional Iminophosphorane Superbases Enable the Highly Enantioselective Sulfa-Michael Addition to Fully Substituted Cyclopropene Carboxylic Acid Derivatives
Source: J Am Chem Soc. 2025 Oct 22;147(44):40045–50. doi: 10.1021/jacs.5c07849 (PMC12593379; doi:10.1021/jacs.5c07849)
Supplement: Supplementary file 1 [file ja5c07849_si_001.pdf]

Bifunctional Iminophosphorane Superbases Enable the Highly Enantioselective Sulfa-Michael Addition to Fully-substituted Cyclopropene Carboxylic Acid Derivatives

Supplementary Information

Kang Yuan, Alberto I. Ristache, Szymon M. Kosc, Agamemnon Crumpton, Darren J. Dixon*

General Experimental **1**
Model Reaction Optimization **2**
Catalyst Screen 2

Condition Screen 7

Key Experiments 8

Control Experiments 10

Preparative Scale Synthesis **11**
Determination of Absolute Stereochemical Configuration **12**
Unsuccessful Substrates **15**
General Procedures **16**
Synthesis of Amino Azides and Protected Amino Amides 16

Synthesis of BIMP Precursors 16

Synthesis of Starating material 18

Synthesis of Sulfa-Michael Addition Products 19

Synthesis of Sulfa-Michael Addition Product Derivatives 20

Analytical and Synthetic Details **22**
Catalyst Synthesis Intermediates 22

Starting matrial and products 33

NMR SPECTRA **103**
CATALYST SYNTHESIS INTERMEDIATES 103

STARTING MATERIAL AND PRODUCTS 114

HPLC Traces **190**
Sulfa-Michael Addition Products 190

References **229**

[back to Table of Contents]

General Experimental

Solvents and Reagents

Bulk solutions were evaporated under reduced pressure using a Büchi rotary evaporator. All solvents were commercially supplied or dried by filtration through activated alumina (powder ~150 mesh, pore size 58 Å, basic, Sigma-Aldrich) columns. Petroleum ether refers to the fraction collected between 30-40 °C. All water used was purified *via* a Merck Millipore reverse osmosis purification system prior to use. All reagents were obtained from commercial suppliers and used without further purification. All reactions were performed under an inert atmosphere using oven-dried glassware and standard Schlenk technique, unless otherwise stated.

Chromatography

Flash column chromatography was carried out using Merck Silicagel 60, particle size 40-63 µm. All reactions were followed by thin-layer chromatography (TLC) when practical, using Merck aluminium-backed Silicagel 60 F254 fluorescent treated silica which was visualised under UV light (λ_{max} = 254 or 365 nm) or by staining with aqueous basic $KMnO_4$, I_2, aqueous acidic vanillin or acidic ninhydrin in *n*-butanol.

Enantiomeric excesses (ee) were determined by chiral high-performance liquid chromatography (HPLC) and chiral supercritical fluid chromatography (SFC) analysis.

Chiral HPLC analysis was performed on an Agilent 1200 series instrument using an appropriate chiral stationary phase column, specified in the individual experiment, and by comparing the samples with the appropriate racemic mixtures.

Chiral SFC analysis was performed on a Waters Acquity UPC2 instrument using an appropriate chiral stationary phase column, specified in the individual experiment, and by comparing the samples with the appropriate racemic mixtures.

Spectroscopy and Spectrometry

^1H and ^{13}C and NMR spectra were recorded using Bruker AVIII HD 400, and Bruker AVII 500 spectrometers using $CDCl_3$ and Benzene-d_6. Chemical shifts (δ) are quoted in parts per million (ppm) relative to tetramethylsilane (δ_{TMS} 0.00 ppm) and referenced to the solvent residual peak (^1H: δ_{CDCl3} 7.26 ppm, ^{13}C: δ_{CDCl3} 77.16 ppm). Coupling constants (*J*) are quoted in Hertz (Hz), rounded to the nearest 0.1 Hz. The ^1H NMR spectra are reported as follows: ppm (multiplicity, coupling constants, number of protons, assignment). Two-dimensional (COSY, HSQC, HMBC) NMR spectroscopy was utilised to assist the assignment. Spectra were analyzed using Mestrelab MestReNova 14.2.0 software.

Low resolution mass spectra (LRMS) were recorded on a Waters LCT Premier mass spectrometer operating in positive and negative ionisation modes. High resolution mass spectra (HRMS) were recorded on a Bruker µTOF mass spectrometer.

Melting Points and Specific Rotations

Melting points were recorded in degrees Celsius (°C), using a Leica Galen III hot-stage microscope apparatus and are reported uncorrected.

Specific rotations ($[\alpha]_D^T$) are reported in 10^{-1} deg·cm^2 g^{-1}; D refers to the D-line of sodium (589 nm); temperatures (T) are given in degrees Celsius (°C). Specific rotations were calculated from optical rotations measured using a Perkin Elmer Model 341 polarimeter with a sodium lamp and a cell length of 1 dm, concentrations (*c*) are reported in g/100 mL.

Naming of Compounds

Compound names are those generated by PerkinElmer ChemDraw 20.0.0.41 software, according to IUPAC nomenclature.

[back to Table of Contents]

Model Reaction Optimization

In this section detailed information can be found about the reaction optimization. The optimization table has been divided into different parts for the sake of clarity. Throughout the process multiple methodical screenings of multiple conditions have been performed. In the key experiments section are highlighted the most important experiments, that ultimately led to the optimized catalyst structure.

Catalyst Screen

In this section all the different iminophosphorane catalysts tested in this project are listed (**Table S1**). At the end of the section are three figures (**Figure S1**, **S2** and **S3**) containing all the organic chiral azides (**A2-A58**) and phosphines (**P1-P19**) that were used to form the corresponding iminophosphorane catalysts *in situ*. Reactions were run according to general procedures described here.

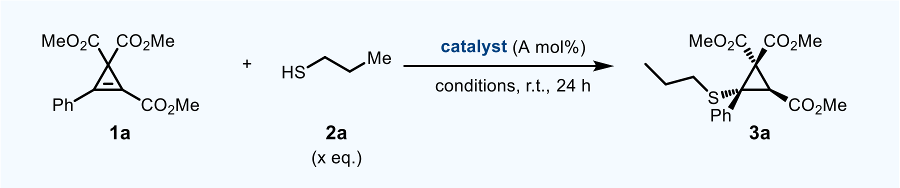

entry	c (M)	thiol (eq.)	solvent	phosphine	mol% P	azide	mol% A	yield (%)	ee (%)
**1**	1	3.0	THF	-	-	**BEMP**	10	78	0
**2**	1	3.0	THF	**P1**	10	**A2**	10	66	9
**3**	1	3.0	THF	**P1**	10	**A3**	10	76	16
**4**	1	3.0	THF	**P1**	10	**A4**	10	68	27
**5**	1	3.0	THF	**P1**	10	**A5**	10	76	0
**6**	1	3.0	THF	**P1**	10	**A6**	10	78	20
**7**	1	3.0	THF	**P1**	10	**A7**	10	76	5
**8**	1	3.0	THF	**P1**	10	**A9**	10	87	25
**9**	1	3.0	THF	**P1**	10	**A10**	10	82	7
**10**	1	3.0	THF	**P1**	10	**A11**	10	69	20
**11**	1	3.0	THF	**P1**	10	**A12**	10	74	10
**12**	1	3.0	THF	**P1**	10	**A13**	10	68	35
**13**	1	3.0	THF	**P1**	10	**A14**	10	74	31
**14**	1	3.0	THF	**P1**	10	**A15**	10	76	23
**15**	1	3.0	THF	**P1**	10	**A16**	10	79	27
**16**	1	3.0	THF	**P1**	10	**A17**	10	73	20
**17**	1	3.0	THF	**P1**	10	**A18**	10	78	40
**18**	1	3.0	THF	**P1**	10	**A19**	10	89	67
**19**	1	3.0	THF	**P1**	10	**A20**	10	52	0
**20**	1	3.0	THF	**P1**	10	**A21**	10	56	8
**21**	1	3.0	THF	**P1**	10	**A22**	10	58	5
**22**	1	3.0	THF	**P1**	10	**A23**	10	58	25
**23**	1	3.0	THF	**P1**	10	**A24**	10	80	43
**24**	1	3.0	THF	**P1**	10	**A25**	10	99	23
**25**	1	3.0	THF	**P1**	10	**A26**	10	75	23
**26**	1	3.0	THF	**P1**	10	**A27**	10	81	45
**27**	1	3.0	THF	**P1**	10	**A28**	10	80	43
**28**	1	3.0	THF	**P1**	10	**A29**	10	99	40

**29**	1	3.0	THF	**P1**	10	**A30**	10	71	31
**30**	1	3.0	THF	**P1**	10	**A31**	10	56	25
**31**	1	3.0	THF	**P1**	10	**A32**	10	85	0
**32**	1	3.0	THF	**P1**	10	**A33**	10	76	11
**33**	1	3.0	THF	**P1**	10	**A34**	10	98	45
**34**	1	3.0	THF	**P1**	10	**A35**	10	83	57
**35**	1	3.0	THF	**P1**	10	**A36**	10	67	32
**36**	1	3.0	THF	**P1**	10	**A37**	10	83	48
**37**	1	3.0	Toluene	**P1**	10	**A37**	10	83	50
**38**	1	3.0	THF	**P1**	10	**A38**	10	72	20
**39**	1	3.0	THF	**P1**	10	**A39**	10	89	33
**40**	1	3.0	THF	**P1**	10	**A40**	10	78	15
**41**	1	3.0	THF	**P1**	10	**A41**	10	60	13
**42**	1	3.0	THF	**P1**	10	**A42**	10	82	13
**43**	1	3.0	THF	**P1**	10	**A43**	10	67	35
**44**	1	3.0	THF	**P1**	10	**A44**	10	95	7
**45**	1	3.0	THF	**P1**	10	**A45**	10	94	25
**46**	1	3.0	THF	**P1**	10	**A46**	10	89	30
**47**	1	3.0	THF	**P1**	10	**A47**	10	83	31
**48**	1	3.0	THF	**P1**	10	**A48**	10	75	52
**49**	0.1	2.0	Toluene	**P8**	10	**A49**	10	82	87
**50**	0.1	2.0	Toluene	**P8**	10	**A50**	10	99	50
**51**	0.1	2.0	Toluene	**P8**	10	**A51**	10	99	77
**52**	0.1	2.0	Toluene	**P8**	10	**A52**	10	86	86
**53**	0.1	2.0	Toluene	**P8**	10	**A53**	10	69	60
**54**	0.1	2.0	Toluene	**P8**	10	**A54**	10	83	60
**55**	0.1	2.0	Toluene	**P8**	10	**A55**	10	99	63
**56**	0.1	2.0	Toluene	**P8**	10	**A56**	10	68	55
**57**	0.1	2.0	Toluene	**P8**	10	**A57**	10	96	78
**58**	0.1	2.0	Toluene	**P8**	10	**A58**	10	70	67
**59**	1	3.0	THF	**P1**	10	**A8**	10	99	78
**60**	1	3.0	THF	**P2**	10	**A8**	10	99	80
**61**	1	3.0	THF	**P3**	10	**A8**	10	92	78
**62**	1	3.0	THF	**P4**	10	**A8**	10	99	85
**63**	1	3.0	THF	**P5**	10	**A8**	10	30	60
**64**	1	3.0	THF	**P6**	10	**A8**	10	75	65
**65**	1	3.0	THF	**P7**	10	**A8**	10	99	88
**66**	1	3.0	THF	**P8**	10	**A8**	10	99	90
**67**	1	3.0	THF	**P9**	10	**A8**	10	71	62
**68**	1	3.0	THF	**P10**	10	**A8**	10	90	83
**69**	1	3.0	THF	**P11**	10	**A8**	10	85	57
**70**	1	3.0	THF	**P12**	10	**A8**	10	65	65
**71**	1	3.0	THF	**P13**	10	**A8**	10	82	80
**72**	1	3.0	THF	**P14**	10	**A8**	10	36	30
**73**	1	3.0	THF	**P15**	10	**A8**	10	78	70
**74**	1	3.0	THF	**P16**	10	**A8**	20	93	84
**75**	1	3.0	THF	**P17**	10	**A8**	10	85	73
**76**	1	3.0	THF	**P18**	10	**A8**	10	63	60
**77**	1	3.0	THF	**P19**	10	**A8**	10	70	84
**78**	1	2.0	Toluene	**P7**	10	**A8**	10	98	92

[back to Table of Contents]

**79**	1	1.6	Toluene	**P7**	10	**A8**	10	98	92
**80**	1	1.2	Toluene	**P7**	10	**A8**	10	99	92
**81**	0.5	2.0	Toluene	**P7**	10	**A8**	10	99	92
**82**	0.2	2.0	Toluene	**P7**	10	**A8**	10	99	93
**83**	0.1	2.0	Toluene	**P7**	10	**A8**	10	99	94
**84**	1	3.0	Toluene	**P7**	10	**A8**	10	99	90
**85**	1	3.0	EtOAc	**P7**	10	**A8**	10	99	85
**86**	1	3.0	TBME	**P7**	10	**A8**	10	85	85
**87**	1	3.0	DCM	**P7**	10	**A8**	10	76	80
**88**	0.1	2.0	Toluene	**P7**	10	**A57**	10	94	74
**89**	0.1	2.0	Toluene	**P1**	10	**A57**	10	85	70
**90**	0.1	2.0	Toluene	**P8**	10	**A8**	10	99	94
**91**	0.1	2.0	Toluene	**P8**	5	**A8**	5	99	94
**92**	0.1	2.0	Toluene	**P8**	10		0	0	0
**93**	0.1	2.0	Toluene		0	**A8**	10	0	0

Table S1. Selected examples of catalyst screen. All yields are isolated yields. Ee was determined by HPLC on a chiral stationary phase.

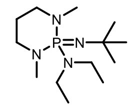
BEMP

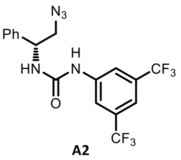
A2

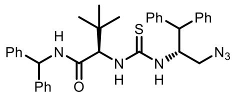
A3

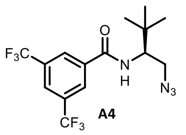
A4

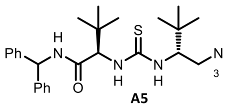
A5

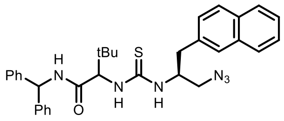
A6

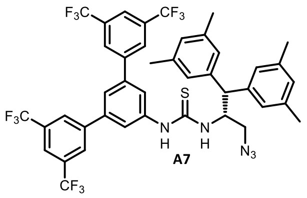
A7

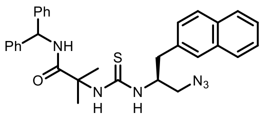
A9

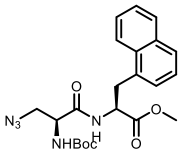
A10

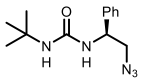
A11

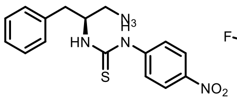
A12

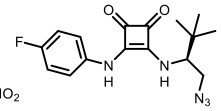
A13

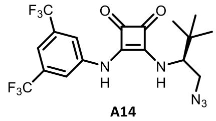
A14

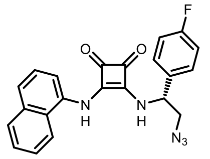
A15

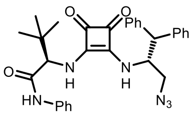
A16

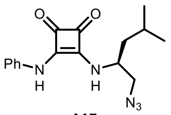
A17

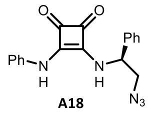
A18

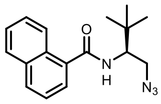
A19

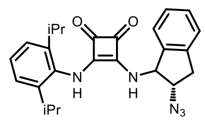
A20

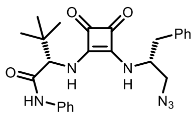
A21

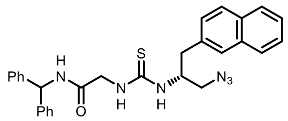
A22

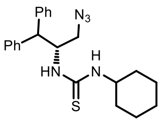
A23

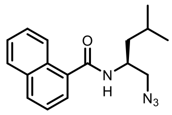
A24

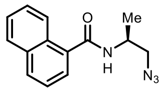
A25

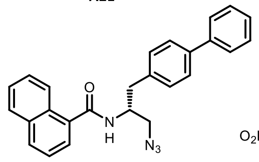
A26

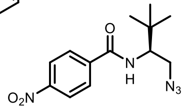
A27

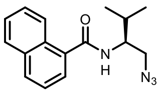
A28

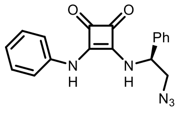
A29

[back to Table of Contents]

Figure S1. Organic azides used for BIMP formation.

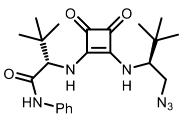
A30

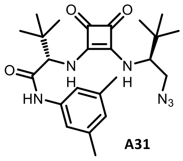
A31

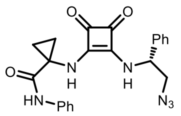
A32

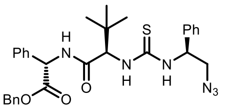
A33

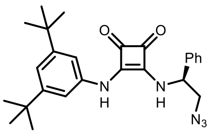
A34

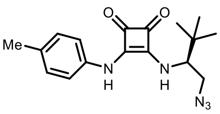
A35

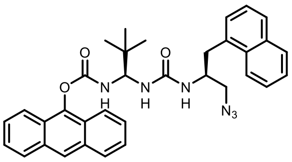
A36

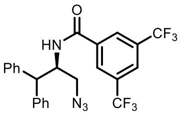
A37

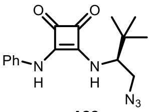
A38

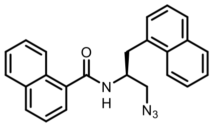
A39

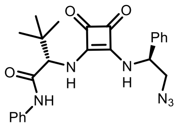
A40

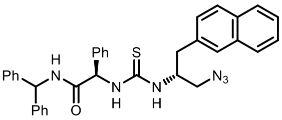
A41

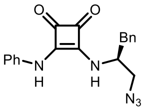
A42

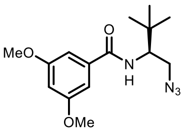
A43

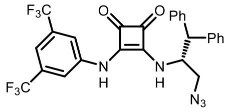
A44

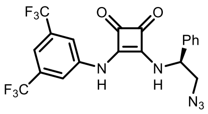
A45

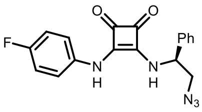
A46

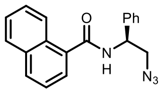
A47

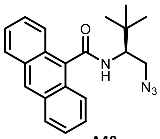
A48

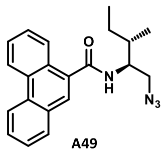
A49

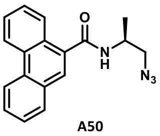
A50

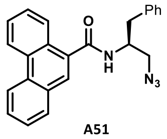
A51

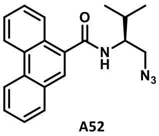
A52

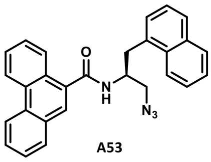
A53

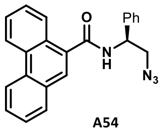
A54

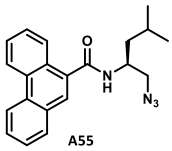
A55

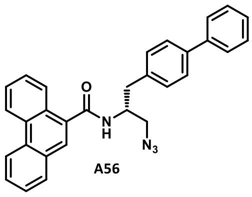
A56

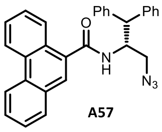
A57

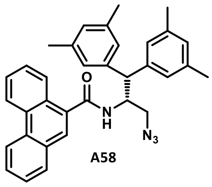
A58

Figure S2. Organic azides used for BIMP formation.

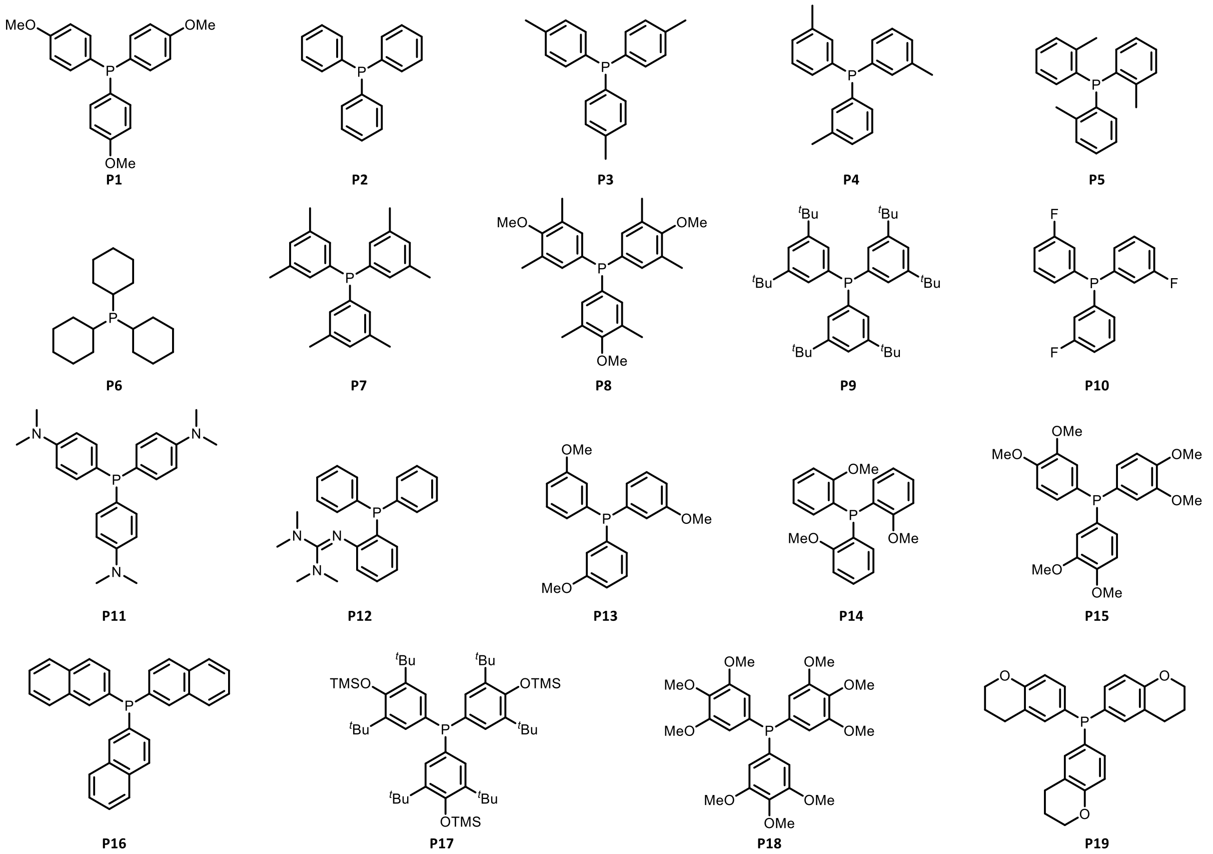

Figure S3. Phosphines used for BIMP formation.

[back to Table of Contents]

Condition Screen

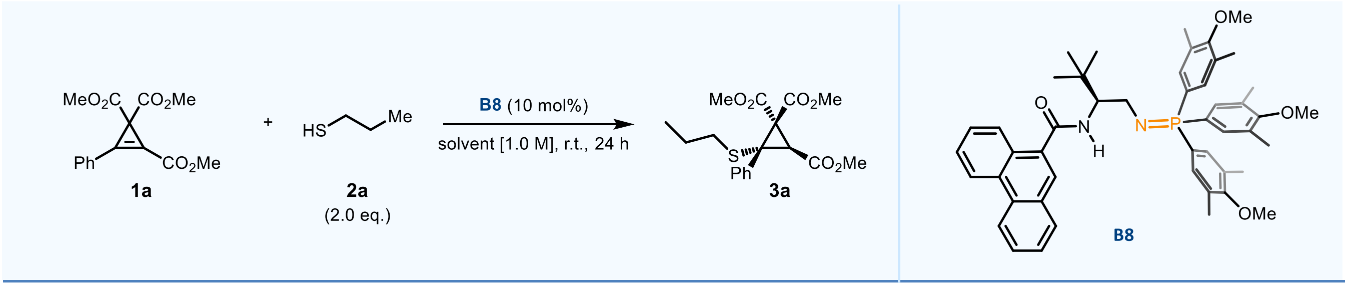

entry	solvent	yield (%)	ee (%)
**1**	THF	99	88
**2**	Toluene	99	90
**3**	DCM	76	80
**4**	TBME	85	85
**5**	EtOAc	99	85

Table S2. Initial solvent screen. All yields are isolated yields. Ee was determined by HPLC on a chiral stationary phase. PMP= *para*-methoxy phenyl.

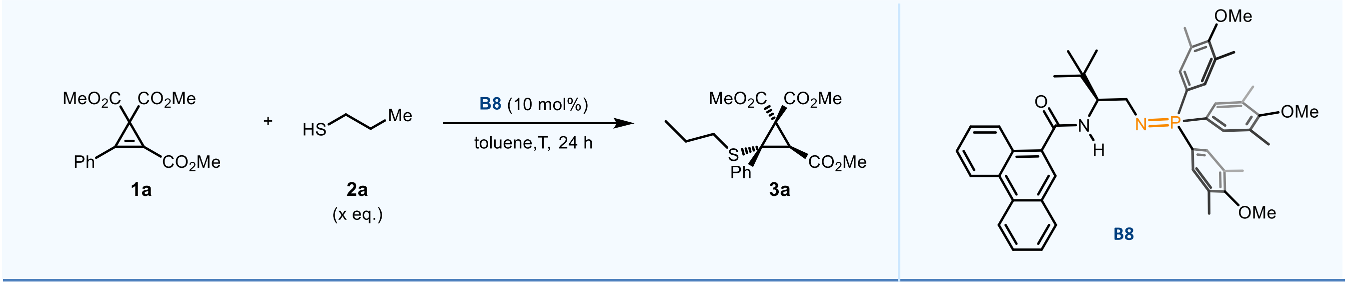

entry	c 1a [M]	eq. 2a(x)	T (°C)	yield (%)	ee (%)
**1**	1	3.0	r.t.	96	90
**2**	1	**2.0**	r.t.	98	92
**3**	1	**1.2**	r.t.	98	92
**4**	**0.5**	**2.0**	r.t.	99	92
**5**	**0.2**	**2.0**	r.t.	99	93
**6**	**0.1**	**2.0**	r.t.	99	94
**7**	**0.1**	**2.0**	**0**	95	85
**8**	**0.1**	**2.0**	**40**	99	86

Table S3. Screening of conditions. All yields are isolated yields. Ee was determined by HPLC on a chiral stationary phase. all the starting materials (including corresponding phosphine and azide) were added to the reaction mixture in Toluene. Changes in conditions compared to Entry 1 are highlighted.

[back to Table of Contents]

KEY EXPERIMENTS

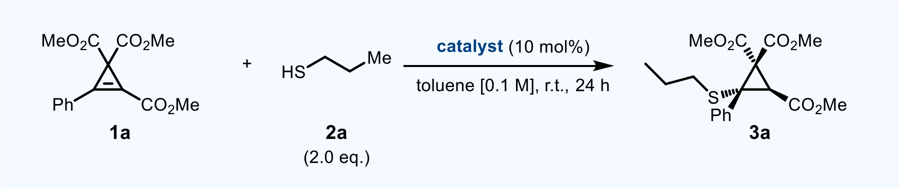

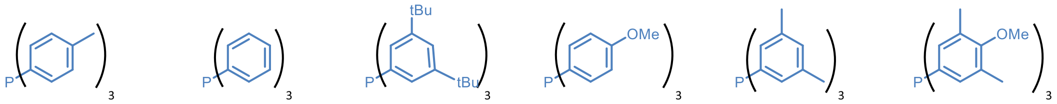

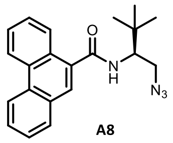

	P3	P2	P9	P1	P7	P8
A8	**78%** ee<br>**92%** yield	**80%** ee<br>**99%** yield	**62%** ee<br>**71%** yield	**78%** ee<br>**99%** yield	**88%** ee<br>**99%** yield	**90%** ee<br>**99%** yield

Table S4. Systematic screening of iminophosphorane substituents. All yields are isolated yields. Ee was determined by HPLC on a chiral stationary phase. In the intersections are results obtained from the reaction catalysed by the corresponding BIMP.

[back to Table of Contents]

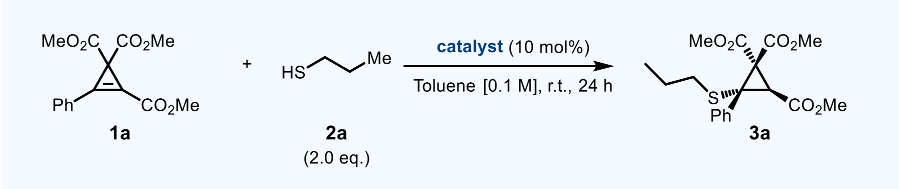

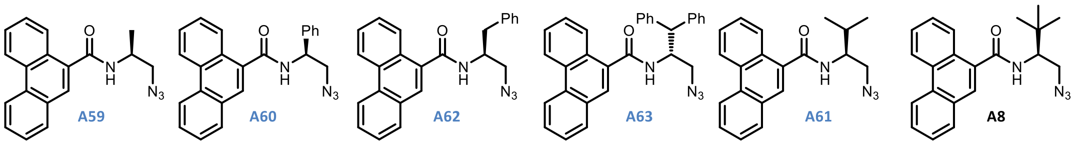

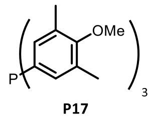

	A59	A60	A62	A63	A61	A8
P17	50% ee<br>99% yield	60% ee<br>83% yield	77% ee<br>99% yield	78% ee<br>96% yield	86% ee<br>89% yield	94% ee<br>99% yield

Table S5. Systematic screening of right-hand side substituents. All yields are isolated yields. Ee was determined by HPLC on a chiral stationary phase. In the intersections are results obtained from the reaction catalysed by the corresponding BIMP.

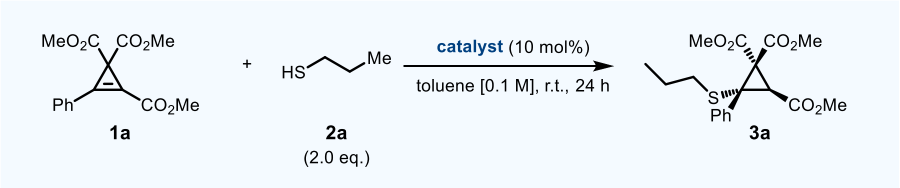

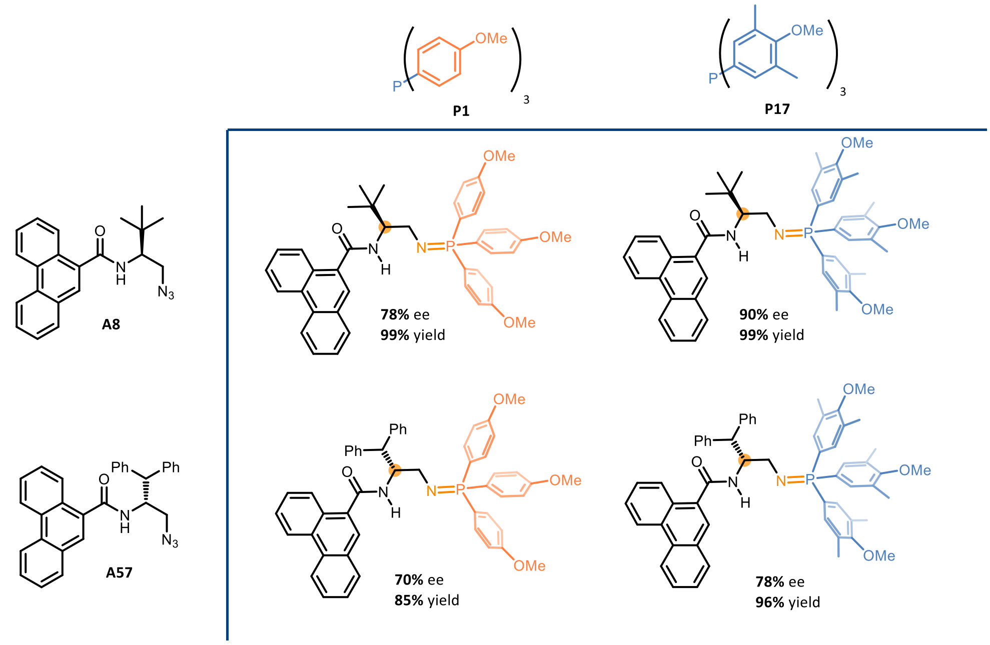

Table S6. Four experiments that demonstrate the importance of iminophosphorane substituents. All yields are isolated yields. Ee was determined by HPLC on a chiral stationary phase.

[back to Table of Contents]

Control Experiments

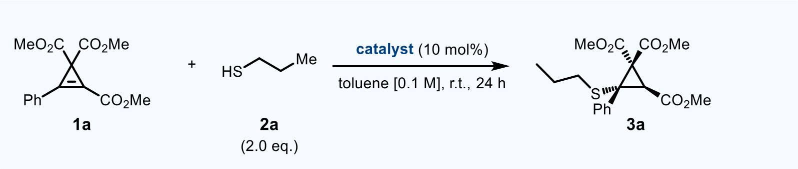

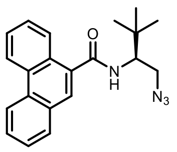

A8

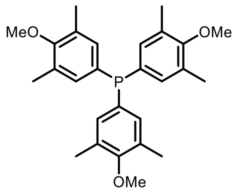

P8

entry	catalyst	conversion (%)
1	-	0
2	**A8**	0
3	**P8**	0

Table S7. Control experiments to reveal background reactions. Conversion was determined by NMR analysis.

[back to Table of Contents]

PREPARATIVE SCALE SYNTHESIS

Azide **A8** (64 mg, 0.19 mmol, 5.0 mol%, **Scheme S1, II**, left) and phosphine **P8** (80 mg, 0.19 mmol, 5.0 mol%, **I**, right) were weighed in a three-neck 50 mL round-bottom flask equipped with a stir bar, glass stopper, rubber septum and swan neck adapter connected to a Schlenk-line. The flask was degassed and refilled with nitrogen using standard Schlenk-technique three times. Degassed anhydrous THF (3.8 mL, 0.05 M) was added then the mixture was stirred for 24 hours at room temperature (**II**). THF was then removed with a stream of nitrogen and the vessel was degassed and refilled with nitrogen three times using standard Schlenk-technique (**III**). Substrate **1a** (1.0 g, 3.8 mmol, 1.0 eq., **IV**) was added to catalyst **B8** then the flask was sealed and degassed and refilled with nitrogen three times using standard Schlenk-technique. Toluene (dried over molecular sieves [4 Å] for 48 hours, 19 mL, 0.20 M) then thiol **2a** (0.7 mL, 7.6 mmol, 2.0 eq., dropwise over 10 minutes) were added to the reaction mixture then it was stirred for 24 hours at room temperature (**V**). The reaction mixture was then filtered through a short silica plug eluting with pentane : EtOAc 50% to quench the BIMP catalyst. Volatiles were removed *in vacuo* and flash column chromatography (pentane : EtOAc 0% to 15%) afforded product **3a** (1.39 g, 3.8 mmol, 99% yield, 94% ee, yellow oil). Analytical data were identical with those of product **3a**. [analytical data]

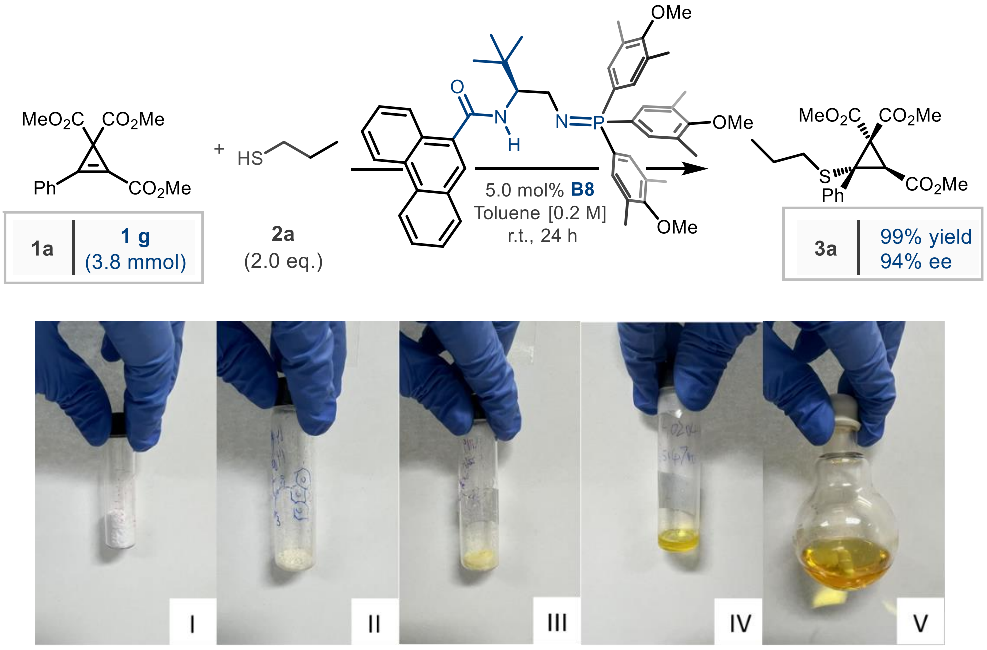

Scheme S1. Preparative scale synthesis (picture: **I**: phosphine **P19**; **II**: azide **A8**; **III**: substrate **1a**; **IV**: product **3a**; **V**: reaction flask).

[back to Table of Contents]

DETERMINATION OF ABSOLUTE STEREOCHEMICAL CONFIGURATION

XRD for trimethyl (2s,3r)-3-(phenethylthio)-3-phenylcyclopropane-1,1,2-tricarboxylate (**3g**)

(CCDC: 2449826)

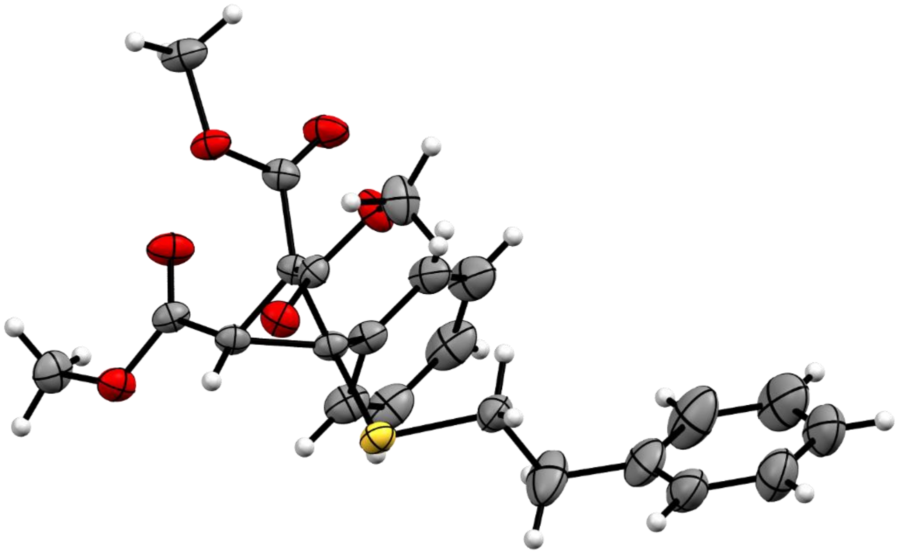

Table 1. Crystal data and structure refinement for compound **3g**.

Identification code 7869

Empirical formula $C_{23} H_{24} O_6 S$

Formula weight 428.51

Temperature 150 K

Wavelength 1.54184 Å

Crystal system Monoclinic

Space group P 1 21 1

Unit cell dimensions a = 17.6487(2) Å α= 90°.

b = 8.13700(10) Å β= 111.4760(10)°.

c = 15.95770(10) Å γ = 90°.

[back to Table of Contents]

Volume 2132.54(4) Å^3

Z 4

Density (calculated) 1.335 Mg/m^3

Absorption coefficient 1.665 mm^{-1}

F(000) 904

Crystal size 0.380 x 0.270 x 0.050 mm^3

Theta range for data collection 3.199 to 74.497°.

Index ranges -22<=h<=22, -10<=k<=9, -19<=l<=19

Reflections collected 81653

Independent reflections 8508 [R(int) = 0.036]

Completeness to theta = 74.497° 100.0 %

Absorption correction Semi-empirical from equivalents

Max. and min. transmission 0.92 and 0.38

Refinement method Full-matrix least-squares on F2

Data / restraints / parameters 8507 / 1 / 606

Goodness-of-fit on F2 0.9954

Final R indices [I>2sigma(I)] R1 = 0.0301, wR2 = 0.0896

R indices (all data) R1 = 0.0351, wR2 = 0.0980

Absolute structure parameter 0.003(3)

Largest diff. peak and hole 0.19 and -0.18 e.Å^3

[back to Table of Contents]

XRD for trimethyl (2S,3R)-3-phenyl-3-(propylsulfonyl)cyclopropane-1,1,2-tricarboxylate (**4a)**
(CCDC: 2476604)

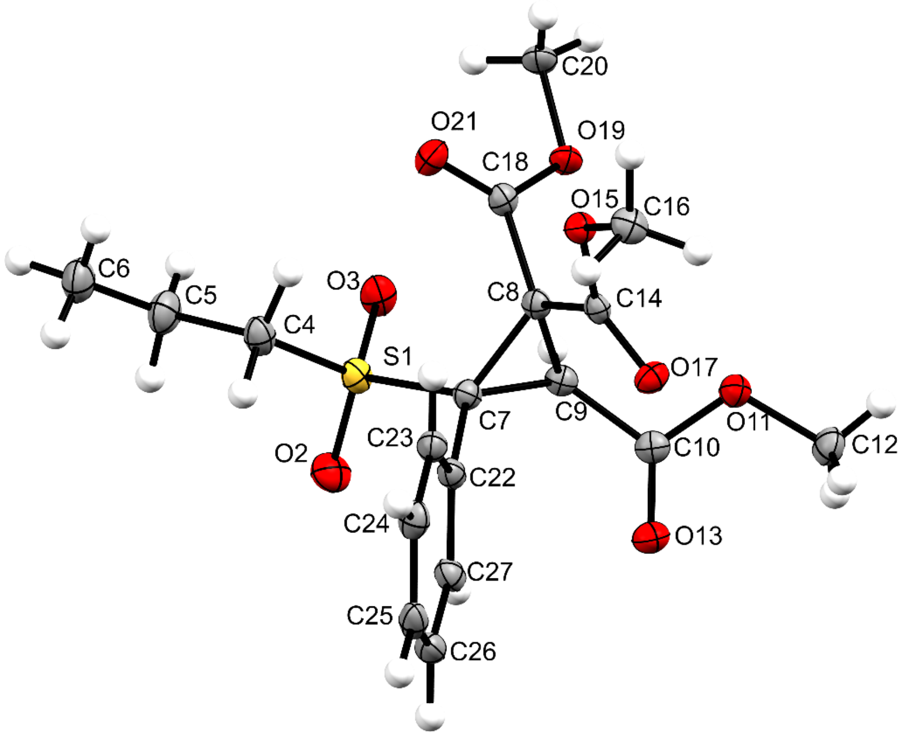

Table 1. Crystal data and structure refinement for 7889.

Identification code 7886

Empirical formula $C_{18} H_{22} O_8 S$

Formula weight 398.43

Temperature 100 K

Wavelength 1.54184 Å

Crystal system Orthorhombic

Space group P 21 21 21

Unit cell dimensions a = 8.43620(10) Å α= 90°.

b = 13.91410(10) Å β= 90°.

c = 15.96890(10) Å γ = 90°.

Volume 1874.46(3) Å^3

[back to Table of Contents]

Z 4

Density (calculated) 1.412 Mg/m^3

Absorption coefficient 1.928 mm^{-1}

F(000) 840

Crystal size 0.366 x 0.304 x 0.179 mm^3

Theta range for data collection 4.214 to 75.876°.

Index ranges -10<=h<=9, -17<=k<=17, -19<=l<=19

Reflections collected 74923

Independent reflections 3857 [R(int) = 0.031]

Completeness to theta = 73.600° 100.0 %

Absorption correction Semi-empirical from equivalents

Max. and min. transmission 0.71 and 0.19

Refinement method Full-matrix least-squares on F2

Data / restraints / parameters 3857 / 0 / 246

Goodness-of-fit on F2 1.0061

Final R indices [I>2sigma(I)] R1 = 0.0205, wR2 = 0.0561

R indices (all data) R1 = 0.0206, wR2 = 0.0561

Absolute structure parameter -0.002(2)

Extinction coefficient 72(4)

Largest diff. peak and hole 0.25 and -0.17 e.Å^{-3}

Unsuccessful Substrates

These examples didn't undergo the desired sulfa-Michael addition, or did so with unsatisfactory results, highlighting the limitations of our methodology. Reactions were run according to **GP-08** (**Figure S5**).

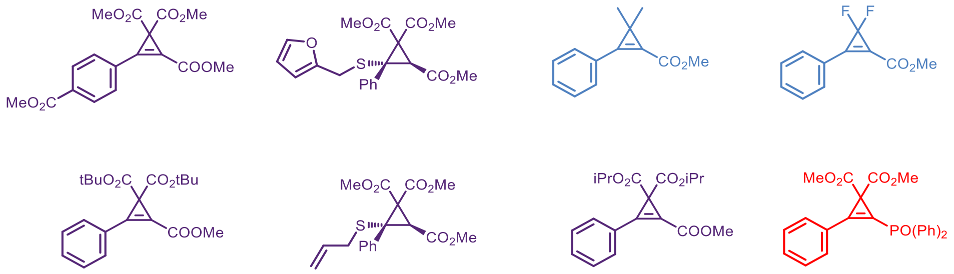

Figure S5. Unsuccessful substrates (purple: inability to fully characterize; red: no reactivity, blue: low ee (up to 53%)).

[back to Table of Contents]

GENERAL PROCEDURES

SYNTHESIS OF AMINO AZIDES AND PROTECTED AMINO AMIDES

The aqueous phase— containing NaN_3 — was quenched with a concentrated bleach solution overnight at room temperature before being discarded.

GP-01
[synthesis of *N*-trifluoroacetate-protected *tert*-leucine derived amino azide][1]

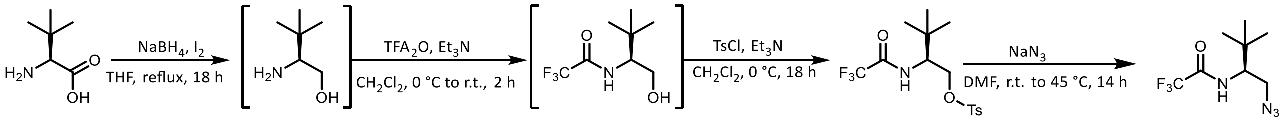

According to the modified literature procedure[1], *N*-trifluoroacetate-protected *tert*-leucine derived amino azide was synthesised as shown. (The reaction was repeated multiple times on different scales (0.2 – 26.2 mmol) with similar results.

GP-02
[deprotection of *N*-trifluoroacetate-protected *tert*-leucine derived amino azide][1]

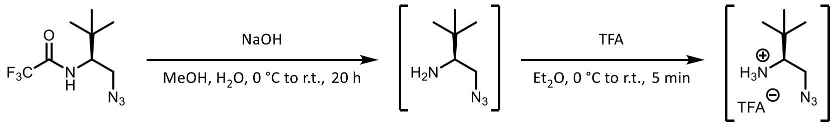

S1

According to the modified literature procedure[1], *N*-Trifluoroacetate-protected *tert*-leucine derived amino azide (714 mg, 3.0 mmol, 1.0 eq.) was dissolved in a mixture of MeOH (8.0 mL, 0.38 M) and water (4.0 mL 0.75 M). The mixture was cooled to 0 °C, then NaOH (1.44 g, 36 mmol, 12.0 eq.) was added, then the reaction was warmed to room temperature. After full conversion (20 hours), the mixture was extracted with Et_2O (4 × 20 mL), then the organic phase was dried over anhydrous $MgSO_4$ (warning: the deprotected amino azide is volatile). The ether solution was cooled to 0 °C, then TFA (3.0 mL, 1.0 mL/mmol) was added to form the TFA salt of the amino azide. Volatiles were removed under a stream of nitrogen. The corresponding ammonium azide was used without further purification and was free based using Et_3N in any consecutive synthetic step *in situ*. The reaction was repeated multiple times on different scales (0.1 – 3.0 mmol) with similar results.

SYNTHESIS OF BIMP PRECURSORS

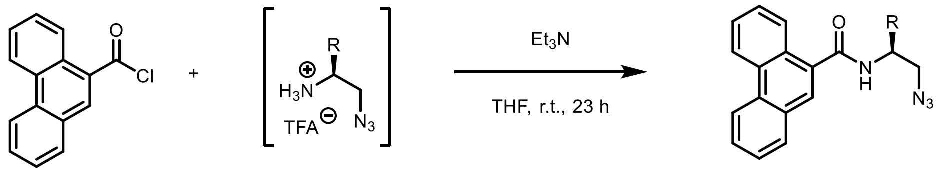

S1

GP-03
[synthesis of BIMP precursors][2]

According to the modified literature procedure[2], the ammonium azide (**S1**, 0.1 mmol, 1.0 eq) was dissolved in anhydrous THF (2 mL) under a nitrogen atmosphere. Phenanthrene-9-carbonyl chloride (29 mg, 0.12 mmol, 1.2 eq.) and triethylamine (30 mg, 0.3 mmol, 3.0 eq.) were added. The resulting suspension was stirred at room temperature for 23

hours before quenching with water (2 mL). The reaction mixture was extracted with Et_2O (3 x 3 mL). The combined organic layers were dried over anhydrous $MgSO_4$ and the volatiles were removed in vacuo. The azide product was obtained as a colourless solid after FCC (pentane : EtOAc = 9:1) and trituration with pentane.

[back to Table of Contents]

SYNTHESIS OF STARATING MATERIAL

GP-04
[synthesis of diazomalonate][3]

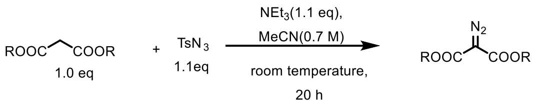

According to the literature procedure, related malonate (15 mmol, 1 eq), triethylamine (16.5 mmol, 1.1 eq) and tosyl azide (16.5 mmol, 1.1 eq) were dissolved in acetonitrile (20 mL). The solution was stirred at room temperature for 20 h. The solution was concentrated under reduced pressure and partitioned between dichloromethane and water. The layers were separated and the aqueous layer was extracted with DCM. The organic layers were combined and dried over $MgSO_4$. The crude was filtered througha plug of celite to remove most of the tosylamide formed during the reaction. Then, purification by column chromatography afforded thediazomalonate as a yellow oil which solidified instorage at 0 °C.

GP-05
[synthesis of terminal cyclopropene][4]

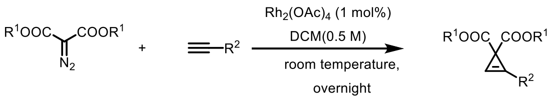

According to the literature procedure[4], the desired diazonium compound (1.0 eq) in CH_2Cl_2 (0.5 M) was added dropwise overnight using a syringe pump to a solution of $Rh_2(OAc)_4$ (1.0 mol%) in the desired terminal alkyne (3.0 eq). Following the addition, the reaction was monitored by TLC and further equivalents of alkene and $Rh_2(OAc)_4$ were added until complete disappearance of the diazonium compound was observed. The crude product was flushed through a plug of celite with CH_2Cl_2 and purified by silica gel column chromatography to provide the desired terminal cyclopropene.

GP-06
[synthesis of cyclopropene starting material][5]

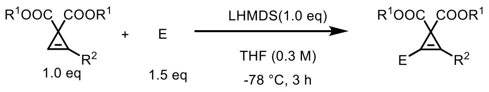

LiHMDS (1.0 M in THF, 1.0 eq) was added dropwise to a stirred solution of the desired terminal cyclopropene (1.0 eq) and electrophile (1.5 eq) at −78 °C in THF (0.3 M). The solution was stirred at −78 °C until completion was observed by TLC. The reaction was quenched with sat. aq. NH_4Cl and the aqueous phase was extracted with Et_2O (3 x 20 ml) (if an isocyante was used the reaction was quenched with 1.0 M HCl(aq)). The combined organic extracts were washed with brine then dried over Na_2SO_4, filtered and concentrated in vacuo. The crude material was purified by silica gel column chromatography to provide the desired cyclopropene

[back to Table of Contents]

SYNTHESIS OF SULFA-MICHAEL ADDITION PRODUCTS

Solvents used for enantioselective sulfa-Michael additions were dried over molecular sieves (4 Å) for at least 48 hours. Reactions were conducted under air.

GP-07
[*in situ* generation of BIMP catalysts][2]

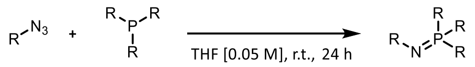

To the corresponding organoazide (0.010 mmol) and trivalent phosphine (0.010 mmol, 1.0 eq.) under argon atmosphere was added THF (0.20 mL, 0.05 M) and the reaction mixture was stirred at 40 °C for 24 hours. The BIMP formation was monitored by TLC. Upon completion, volatiles were removed under a stream of nitrogen, yielding the crude BIMP catalyst, which was used without further purification.

GP-08
[synthesis of enantiomerically enriched 3-thiocyclopropanes]

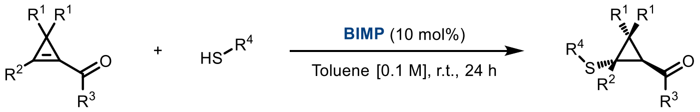

If the substrate was an oil, the corresponding cyclopropene (0.10 mmol, 1.0 eq.) and thiol (2.0–3.0 eq.) were dissolved in toluene (1.0 mL, 0.1 M), then the solution was added to the corresponding BIMP catalyst (0.010 mmol, 10 mol%). In case of solid substrates, the corresponding cyclopropene (0.10 mmol, 1.0 eq.) was added to the BIMP catalyst (0.010 mmol, 10 mol%), then toluene (1.0 mL, 0.1 M), finally the corresponding thiol (2.0–3.0 eq.) were added to the mixture. Reactions were monitored by TLC; crude products were purified by silica gel chromatography as specified in the individual experiment to afford Michael-addition products. The four enantiomers were separated by chiral HPLC using conditions specified in the individual experiment. The ee was determined with HPLC and the d.r. wasdetermined by NMR. [analytical details] [NMR spectra] [HPLC traces]

GP-09
[synthesis of racemic 3-thiocyclopropanes]

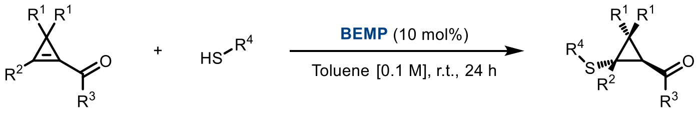

The corresponding unsaturated amide (0.10 mmol, 1.0 eq.) and thiol (0.20 mmol, 2.0 eq.) were dissolved in toluene (1.0 mL, 0.10 M). To the solution was added BEMP (2-*tert*-butylimino-2-diethylamino-1,3-dimethylperhydro-1,3,2-diazaphosphorine) achiral superbase (2.9 µL, 0.010 mmol, 10 mol%). Reactions were monitored by TLC, crude products were purified by silica gel chromatography to afford racemic Michael-addition products. The four enantiomers were separated by chiral HPLC using conditions specified in the individual experiment.

[back to Table of Contents]

Synthesis of Sulfa-Michael Addition Product Derivatives

GP-10

[oxidation to sulfone][1]

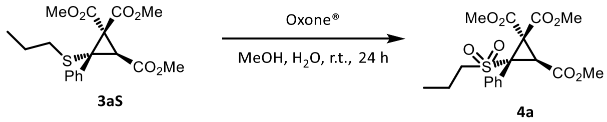

Thiocyclopropane **3a** (74 mg, 0.2 mmol, 1.0 eq.) was dissolved in a 1:1 mixture of MeOH and water (1.0 mL, 0.20 M), then Oxone® (184 mg, 0.6 mmol, 3.0 eq.) was added. The solution was stirred for 24 hours at ambient temperature. The reaction mixture was quenched with NaSCN (200 mg in 3 mL water), then it was further diluted with 3 mL water. The mixture was then washed with CH_2Cl_2 (3 x 3 mL). Combined organics were extracted with concentrated aqueous $NaHCO_3$ (3 mL), then were dried over anhydrous $MgSO_4$. Volatiles were removed *in vacuo* and flash column chromatography (pentane : EtOAc 33% to 50%) afforded product **4a** (93% ee, 69 mg, 86%, colorless solid).

GP-11

[oxidation to sulfoxide][6]

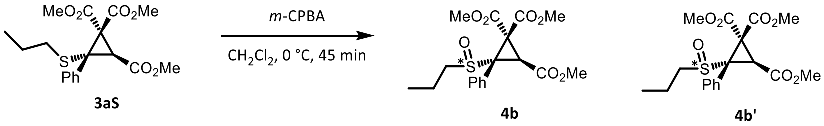

Thioamide **3a** (74 mg, 0.2 mmol, 1.0 eq.) was dissolved in CH_2Cl_2 (5.0 mL, 0.04 M). The solution was cooled to 0 °C, then *m*-CPBA (92%, 56 mg, 0.3 mmol, 1.5 eq.) was added, and the mixture was stirred at 0 °C for 45 min. The reaction mixture was extracted with concentrated aqueous $NaHCO_3$ (3 mL), then the aqueous layer was washed with CH_2Cl_2 (2 x 3 mL). Combined organics were dried over anhydrous $MgSO_4$. Volatiles were removed *in vacuo* and flash column chromatography (pentane : EtOAc 25% to 33%) afforded products **4b** and **4b'** as a 1.1 : 1.0 mixture of diastereoisomers (67 mg, 0.18 mmol, 87%). The two diastereoisomers wereseparated by preparative TLC (hexane : EtOAc 33%, **4b**: 94% ee, **4b'**: 85% ee). The reported sulfoxides slowly decompose when stored at ambient temperature.

GP-12

[desulfurization]

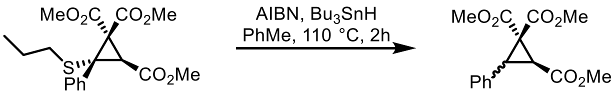

A vial was charged with **3h** (5.0 mg, 0.014 mmol, 1 eq.). The vial was evacuated and backfilled with N_2 (g) three times. To the vial was added anhydrous toluene (0.5 mL), and a solution (0.10 mL) of $^nBu_3SnH$ (0.56 M, 0.056 mmol, 4 eq.) and AIBN (0.012 M, 0.0012 mmol, 12 mol%). The solution was heated to reflux and stirred under Ar (g) for 2 h. The mixture was cooled down to rt, and pushed down a silica plug containing 10% (w/w) of K_2CO_3, and the plug was washed with Et_2O (50 mL). The solution was concentrated and purified by preparative TLC (1:4 EtOAc/hexane) yielding **4c** as a colourless oil (2.0 mg, 0.0068 mmol) in a 50% yield with a 3:2 d.r. (trans/cis) and with 93% ee. The data is in good agreement with the literature reported compound.[19]

[back to Table of Contents]

GP-13

[cycloaddition/desulfurization]

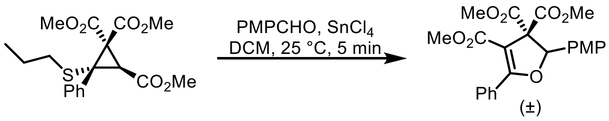

According to a modified literature procedure, compound 3a (22mg, 1.0 eq) and 4-methoxybenzaldehyde (22 µL, 3.0 eq) were dissolved in dry DCM (0.05 M) at r.t. under Ar. Tin tetrachloride in THF (1.0M, 0.2 eq) was added to the reaction mixture dropwise.[20] The light brown solution was stirred at r.t. and monitored by TLC. After 5 minutes, the reaction was quenched with ether and passed through a silica plug. The crude was purified by flash column chromatography using a pentane/ethyl acetate gradient from 5% ethyl acetate/95% pentane to 30% ethyl acetate/70% pentane to yield a thick colourless oil (22mg, 85%, racemic).

[back to Table of Contents]

Analytical and Synthetic Details

Catalyst Synthesis Intermediates

(S)-N-(1-azido-3,3-dimethylbutan-2-yl)phenanthrene-9-carboxamide (**A8**)

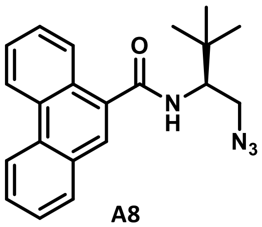

A8

Product **S04** was prepared according to general procedure **GP-03** and was obtained as a colorless solid.

yield: 27 mg (0.079 mmol, 79%).

^{1}H NMR (400 MHz, $CDCl_3$) δ 8.70 – 8.56 (m, 2H), 8.31 – 8.24 (m, 1H), 7.89 – 7.80 (m, 2H), 7.72 – 7.50 (m, 4H), 5.99 (d, *J* = 10.2 Hz, 1H), 4.31 (ddd, *J* = 10.1, 7.8, 4.0 Hz, 1H), 3.71 (dd, *J* = 12.7, 3.9 Hz, 1H), 3.37 (dd, *J* = 12.8, 7.8 Hz, 1H), 1.02 (s, 9H).

^{13}C NMR (101 MHz, $CDCl_3$) δ 175.3, 128.0, 127.1, 126.3, 125.9, 122.9, 122.7, 77.34, 77.0, 76.7, 56.5, 26.8.

HRMS (ESI+, m/Z): exact mass calculated for $C_{21}H_{22}N_4O$ $[M+H]^+$ 347.1866, found 347.1869.

m. p.: 60 – 62 °C.

$[\alpha]_D^{25}$ = 5.1 (c = 1.0, CH_2Cl_2).

[back to Table of Contents]

N-((2S,3S)-1-azido-3-methylpentan-2-yl)phenanthrene-9-carboxamide (**A49**)

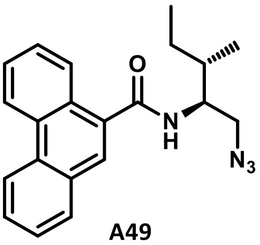

A49

Product **S04** was prepared according to general procedure **GP-03** and was obtained as a colorless solid.

yield: 19 mg (0.054 mmol, 54%).

^{1}H NMR (400 MHz, $CDCl_3$) δ 8.70 – 8.54 (m, 2H), 7.82 (ddd, *J* = 11.5, 7.9, 1.5 Hz, 2H), 7.66 – 7.48 (m, 4H), 5.22 (d, *J* = 1.2 Hz, 1H), 3.75 – 3.61 (m, 1H), 3.61 – 3.48 (m, 1H), 1.65 (m, 5H), 1.00 (m, 5H).

^{13}C NMR (101 MHz, $CDCl_3$) δ 169.9, 135.5, 131.1, 130.5, 130.1, 128.8, 128.6, 127.1, 127.0, 126.9, 125.7, 123.2, 122.8, 122.5, 77.3, 77.0, 76.7, 53.4, 51.1, 46.0, 20.8, 20.7, 20.7, 20.6.

HRMS (ESI+, m/Z): exact mass calculated for $C_{21}H_{22}N_4O$ $[M+H]^+$ 347.1866, found 347.1863.

m. p.: 70 – 72 °C.

$[\alpha]_D^{25}$ = -13.8 (c = 0.8, CH_2Cl_2).

[back to Table of Contents]

(S)-N-(1-azidopropan-2-yl)phenanthrene-9-carboxamide (**A50**)

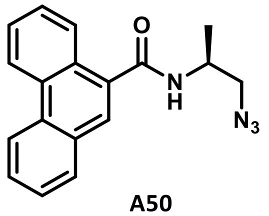

A50

Product **S04** was prepared according to general procedure **GP-03** and was obtained as a colorless solid.

yield: 21 mg (0.072 mmol, 72%).

^{1}H NMR (400 MHz, $CDCl_3$) δ 8.69 – 8.56 (m, 2H), 8.29 – 8.23 (m, 1H), 7.87 – 7.79 (m, 2H), 7.70 – 7.51 (m, 4H), 6.06 (d, *J* = 8.2 Hz, 1H), 4.55 – 4.41 (m, 1H), 3.68 (dd, *J* = 12.3, 4.5 Hz, 1H), 3.46 (dd, *J* = 12.3, 4.3 Hz, 1H), 1.33 (d, *J* = 6.8 Hz, 3H).

^{13}C NMR (101 MHz, $CDCl_3$) δ 129.1, 128.0, 127.3, 127.2, 127.1, 126.3, 126.1, 122.9, 122.6, 77.3, 77.2, 77.0, 76.7, 55.8, 45.4, 18.1.

HRMS (ESI+, m/Z): exact mass calculated for $C_{18}H_{16}N_4O$ $[M+H]^+$ 305.1397, found 305.1400.

m. p.: 50 – 52 °C.

$[\alpha]_D^{25}$ = 7.7 (c = 0.5, CH_2Cl_2).

[back to Table of Contents]

(S)-N-(1-azido-3-phenylpropan-2-yl)phenanthrene-9-carboxamide (**A51**)

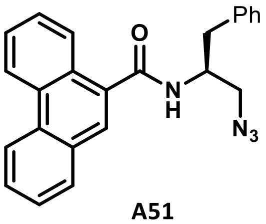

A51

Product **S04** was prepared according to general procedure **GP-03** and was obtained as a colorless solid.

yield: 27 mg (0.072 mmol, 72%).

^{1}H NMR (400 MHz, $CDCl_3$) δ 8.62 (dd, *J* = 12.5, 8.3 Hz, 1H), 8.01 (d, *J* = 8.3 Hz, 1H), 7.80 (d, *J* = 7.9 Hz, 1H), 7.70 – 7.47 (m, 5H), 7.35 – 7.10 (m, 6H), 6.06 (d, *J* = 8.4 Hz, 1H), 4.65 (d, *J* = 8.8 Hz, 1H), 3.74 – 3.39 (m, 2H), 2.98 (d, *J* = 7.5 Hz, 2H).

^{13}C NMR (101 MHz, $CDCl_3$) δ 173.7, 145.0, 128.9, 122.6, 112.1, 77.3, 77.0, 76.7, 63.7, 45.5, 41.5, 13.6.

HRMS (ESI+, m/Z): exact mass calculated for $C_{24}H_{20}N_4O$ $[M+H]^+$ 381.1710, found 381.1720.

m. p.: 67 – 69 °C.

$[\alpha]_D^{25}$ = 11.4 (c = 1.0, CH_2Cl_2).

[back to Table of Contents]

(S)-N-(1-azido-3-methylbutan-2-yl)phenanthrene-9-carboxamide (**A52**)

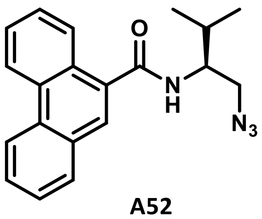

A52

Product **S04** was prepared according to general procedure **GP-03** and was obtained as a colorless solid.

yield: 27 mg (0.062 mmol, 62%).

^{1}H NMR (400 MHz, $CDCl_3$) δ 8.69 – 8.59 (m, 2H), 8.29 – 8.22 (m, 1H), 7.88 – 7.79 (m, 2H), 7.68 – 7.53 (m, 4H), 6.04 (d, *J* = 9.2 Hz, 1H), 4.17 (dddd, *J* = 9.2, 7.8, 5.0, 4.1 Hz, 1H), 3.75 – 3.48 (m, 2H), 1.92 (dp, *J* = 7.7, 6.7 Hz, 1H), 1.02 (dd, *J* = 6.8, 1.8 Hz, 6H).

^{13}C NMR (101 MHz, $CDCl_3$) δ 169.4, 129.1, 128.0, 127.3, 127.2, 127.1, 126.2, 126.1, 122.9, 122.7, 77.3, 77.2, 77.0, 76.7, 54.6, 53.0, 29.7, 19.6, 19.0.

HRMS (ESI+, m/Z): exact mass calculated for $C_{20}H_{20}N_4O$ $[M+Na]^+$ 355.1529, found 355.1543.

m. p.: 58 – 60 °C.

$[\alpha]_D^{25}$ = 5.5 (c = 1.0, CH_2Cl_2).

[back to Table of Contents]

(S)-N-(1-azido-3-(naphthalen-1-yl)propan-2-yl)phenanthrene-9-carboxamide (**A53**)

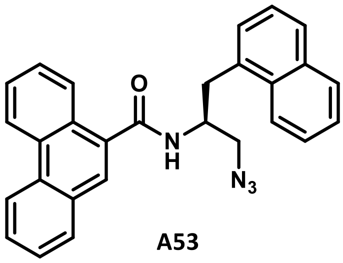

A53

Product **S04** was prepared according to general procedure **GP-03** and was obtained as a colorless solid.

yield: 29 mg (0.067 mmol, 67%).

^{1}H NMR (400 MHz, $CDCl_3$) δ 8.72 (dd, *J* = 13.8, 8.3 Hz, 2H), 8.30 (dd, *J* = 62.6, 8.3 Hz, 2H), 8.04 – 7.37 (m, 12H), 6.32 (d, *J* = 8.3 Hz, 1H), 4.87 (d, *J* = 5.4 Hz, 1H), 3.82 – 3.28 (m, 4H).

^{13}C NMR (101 MHz, $CDCl_3$) δ 198.4, 166.0, 138.8, 130.3, 128.9, 128.0, 123.8, 110.5, 77.3, 77.0, 76.7, 52.1, 50.0, 35.1, 19.7.

HRMS (ESI+, m/Z): exact mass calculated for $C_{28}H_{22}N_4O$ $[M+H]^+$ 431.1866, found 431.1870.

m. p.: 80 – 82 °C.

$[\alpha]_D^{25}$ = 12.7 (c = 1.0, CH_2Cl_2).

[back to Table of Contents]

(S)-N-(2-azido-1-phenylethyl)phenanthrene-9-carboxamide (**A54**)

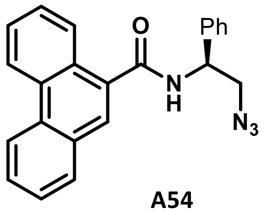

A54

Product **S04** was prepared according to general procedure **GP-03** and was obtained as a colorless solid.

yield: 25 mg (0.067 mmol, 67%).

^{1}H NMR (400 MHz, $CDCl_3$) δ 8.80 – 8.48 (m, 2H), 8.37 – 8.13 (m, 1H), 7.95 – 7.78 (m, 2H), 7.74 – 7.48 (m, 3H), 7.48 – 7.26 (m, 6H), 6.54 (d, *J* = 8.1 Hz, 1H), 5.51 (dt, *J* = 8.1, 5.3 Hz, 1H), 3.94 – 3.67 (m, 2H).

^{13}C NMR (101 MHz, $CDCl_3$) δ 129.1, 127.3, 126.7, 126.1, 77.3, 77.2, 77.0, 76.7, 55.3, 48.7.

HRMS (ESI+, m/Z): exact mass calculated for $C_{23}H_{18}N_4O$ $[M+H]^+$ 367.1553, found 367.1565.

m. p.: 70 – 72 °C.

$[\alpha]_D^{25}$ = 6.6 (c = 0.5, CH_2Cl_2).

[back to Table of Contents]

(S)-N-(1-azido-4-methylpentan-2-yl)phenanthrene-9-carboxamide (**A55**)

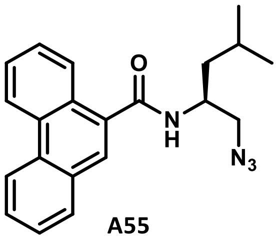

A55

Product **S04** was prepared according to general procedure **GP-03** and was obtained as a colorless solid.

yield: 26 mg (0.075 mmol, 75%).

^{1}H NMR (400 MHz, $CDCl_3$) δ 8.83 – 8.36 (m, 2H), 8.38 – 8.17 (m, 1H), 7.96 – 7.73 (m, 2H), 7.70 – 7.34 (m, 4H), 5.97 (d, *J* = 8.7 Hz, 1H), 4.56 – 4.38 (m, 1H), 3.71 (dd, *J* = 12.4, 4.2 Hz, 1H), 3.47 (dd, *J* = 12.3, 4.0 Hz, 1H), 1.70 (ddt, *J* = 13.0, 8.5, 6.4 Hz, 1H), 1.54 (ddd, *J* = 13.9, 9.4, 5.9 Hz, 1H), 1.41 (ddd, *J* = 13.9, 8.5, 5.5 Hz, 1H), 0.99 (d, *J* = 6.5 Hz, 3H), 0.94 (d, *J* = 6.7 Hz, 3H).

^{13}C NMR (101 MHz, $CDCl_3$) δ 169.2, 131.0, 130.3, 129.1, 128.0, 127.3, 127.2, 127.1, 126.2, 126.1, 122.9, 122.6, 77.3, 77.2, 77.0, 76.7, 55.2, 47.6, 41.1, 25.0, 23.0, 22.2.

HRMS (ESI+, m/Z): exact mass calculated for $C_{21}H_{22}N_4O$ $[M+H]^+$ 347.1866, found 347.1869.

m. p.: 60 – 62 °C

$[\alpha]_D^{25}$ = 13.6 (c = 1.0, CH_2Cl_2).

[back to Table of Contents]

N-((2S,3S)-1-azido-3-methylpentan-2-yl)phenanthrene-9-carboxamide (**A56**)

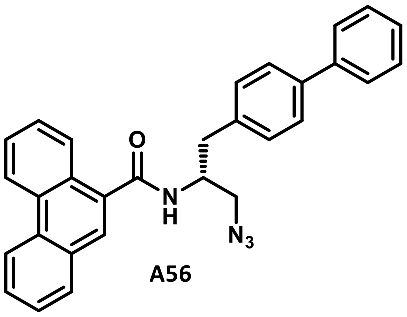

A56

Product **S04** was prepared according to general procedure **GP-03** and was obtained as a colorless solid.

yield: 34 mg (0.074 mmol, 74%).

^{1}H NMR (400 MHz, $CDCl_3$) δ 8.72 – 8.54 (m, 3H), 8.01 (dd, *J* = 8.2, 1.3 Hz, 1H), 7.78 (dd, *J* = 7.9, 1.4 Hz, 1H), 7.72 – 7.23 (m, 13H), 6.10 (d, *J* = 8.5 Hz, 1H), 4.69 (tq, *J* = 7.7, 4.0 Hz, 1H), 3.62 (ddd, *J* = 64.9, 12.4, 4.3 Hz, 2H), 3.01 (d, *J* = 7.5 Hz, 2H).

^{13}C NMR (101 MHz, $CDCl_3$) δ 135.9, 129.7, 129.1, 128.8, 128.0, 127.5, 127.3, 127.3, 127.1, 127.0, 126.3, 126.1, 122.8, 122.6, 77.3, 77.2, 77.0, 76.7, 50.4, 37.6.

HRMS (ESI+, m/Z): exact mass calculated for $C_{30}H_{24}N_4O$ $[M+H]^+$ 457.2023, found 457.2035.

m. p.: 95 – 97 °C.

$[\alpha]_D^{25}$ = -34.2 (c = 1.3, CH_2Cl_2).

[back to Table of Contents]

(R)-N-(3-azido-1,1-diphenylpropan-2-yl)phenanthrene-9-carboxamide (**A57**)

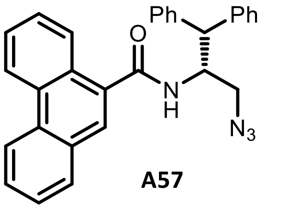

A57

Product **S04** was prepared according to general procedure **GP-03** and was obtained as a colorless solid.

yield: 34 mg (0.074 mmol, 74%).

^{1}H NMR (400 MHz, $CDCl_3$) δ 8.62 – 8.53 (m, 2H), 7.19 (s, 17H), 6.00 (d, *J* = 8.9 Hz, 1H), 5.29 – 5.22 (m, 1H), 4.19 (d, *J* = 11.7 Hz, 1H), 3.75 (dd, *J* = 12.5, 3.3 Hz, 1H), 3.36 (dd, *J* = 12.5, 3.3 Hz, 1H).

^{13}C NMR (101 MHz, $CDCl_3$) δ 169.2, 141.1, 140.9, 130.9, 130.2, 129.2, 129.1, 129.1, 128.2, 127.9, 127.3, 127.2, 127.2, 127.1, 127.0, 126.4, 126.0, 122.7, 122.5, 77.3, 77.0, 76.7, 53.4, 53.2, 51.9.

HRMS (ESI+, m/Z): exact mass calculated for $C_{30}H_{24}N_4O$ $[M+H]^+$ 457.2023, found 457.2012.

m. p.: 97 – 99 °C.

$[\alpha]_D^{25}$ = -13.5 (c = 1.0, CH_2Cl_2).

[back to Table of Contents]

(R)-N-(3-azido-1,1-bis(3,5-dimethylphenyl)propan-2-yl)phenanthrene-9-carboxamide (**A58**)

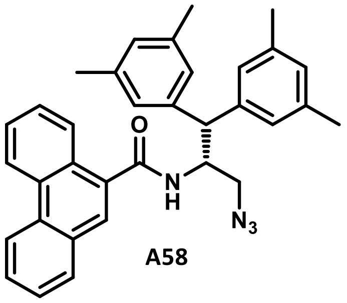

A58

Product **S04** was prepared according to general procedure **GP-03** and was obtained as a colorless solid.

yield: 39 mg (0.076 mmol, 76%).

^{1}H NMR (400 MHz, $CDCl_3$) δ 8.67 (dd, *J* = 10.1, 7.6 Hz, 2H), 7.17 (d, *J* = 109.3 Hz, 13H), 6.04 (d, *J* = 8.5 Hz, 1H), 5.27 (ddt, *J* = 11.9, 8.9, 3.3 Hz, 1H), 4.12 (dd, *J* = 11.7, 2.7 Hz, 1H), 3.84 (dt, *J* = 12.5, 3.2 Hz, 1H), 3.43 (dd, *J* = 12.5, 3.2 Hz, 1H), 2.34 (d, *J* = 4.0 Hz, 12H).

^{13}C NMR (101 MHz, $CDCl_3$) δ 169.2, 138.6, 138.4, 129.0, 128.9, 128.8, 127.9, 127.1, 127.1, 127.0, 126.3, 126.0, 126.0, 125.7, 122.7, 122.6, 77.3, 77.0, 76.7, 53.2, 52.2, 21.4, 21.4.

HRMS (ESI+, m/Z): exact mass calculated for $C_{34}H_{32}N_4O$ $[M+H]^+$ 513.2649, found 513.2662

m. p.: 100 – 102 °C.

$[\alpha]_D^{25}$ = -11.1 (c = 1.0, CH_2Cl_2).

[back to Table of Contents]

STARTING MATRIAL AND PRODUCTS

dimethyl 2-phenylcycloprop-2-ene-1,1-dicarboxylate (1a')

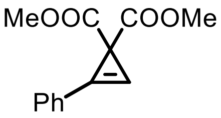

Starting material for compound **1a** was synthesised according to general procedure **GP-05**:

The desired diazonium compound[3] (1.0 eq, 10 mmol, 1.6 g) was dissolved in CH_2Cl_2 (0.5 M, 20 mL) and added dropwise overnight using a syringe pump to a solution of $Rh_2(OAc)_4$ (1.0 mol%, 44 mg) in phenylacetylene (3.0 eq, 30 mmol, 3.1 g). Following the addition, the reaction was monitored by TLC and further equivalents of alkene and $Rh_2(OAc)_4$ were added until complete disappearance of the diazonium compound was observed. The crude product was flushed through a plug of celite with CH_2Cl_2 and purified by flash silica gel column chromatography with pentane/EtOAc = 9/1 to provide the title compound as a yellow solid. (1.5 g, 6.6 mmol, 66%). The spectra is same with the reported literature.[7]

^{1}H NMR (400 MHz, $CDCl_3$) δ 7.66 – 7.41 (m, 5H), 6.89 (s, 1H), 3.73 (s, 6H).

^{13}C NMR (101 MHz, $CDCl_3$) δ 168.9, 132.6, 132.2, 129.1, 122.9, 120.5, 98.2, 52.9, 31.9.

HRMS (ESI) m/z calcd. for $C_{13}H_{12}O_4$ ($[M+Na]^+$) 255.0628, found 255.0637

m. p.: 60 – 62 °C.

[back to Table of Contents]

dimethyl 2-phenylcycloprop-2-ene-1,1-dicarboxylate (1b')

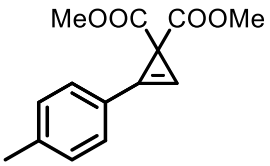

Starting material for **1b** was synthesised according to general procedure **GP-05**:

The desired diazonium compound (1.0 eq, 5 mmol, 800 mg) was dissolved in CH_2Cl_2 (0.5 M, 10 mL) and added dropwise overnight using a syringe pump to a solution of $Rh_2(OAc)_4$ (1.0 mol%, 22 mg) in phenylacetylene (3.0 eq, 15 mmol, 1.5 g). Following the addition, the reaction was monitored by TLC and further equivalents of alkene and $Rh_2(OAc)_4$ were added until complete disappearance of the diazonium compound was observed. The crude product was flushed through a plug of celite with CH_2Cl_2 and purified by flash silica gel column chromatography with pentane/EtOAc = 9/1 to provide the title compound as a yellow solid. (739 mg, 3 mmol, 66%). The spectrua is similar with the reported literature.[8]

^{1}H NMR (400 MHz, $CDCl_3$) δ 7.55 – 7.48 (m, 2H), 7.26 (s, 2H), 6.81 (s, 1H), 3.72 (s, 6H), 2.39 (s, 3H).

^{13}C NMR (101 MHz, $CDCl_3$) (101 MHz, $CDCl_3$) δ 171.3, 141.1, 130.3, 129.6, 121.1, 112.1, 93.8, 52.3, 32.7, 21.6.

HRMS (ESI) m/z calcd. for $C_{14}H_{14}O_4$ ($[M+H]^+$) 247.0965, found 247.0962

m. p.: 62 – 64 °C.

[back to Table of Contents]

dimethyl 2-(o-tolyl)cycloprop-2-ene-1,1-dicarboxylate (1c')

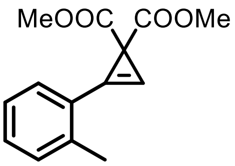

Starting material for Compound 12 was synthesised according to general procedure **GP-05**:

The desired diazonium compound (1.0 eq, 5 mmol, 800 mg) was dissolved in CH_2Cl_2 (0.5 M, 10 mL) and added dropwise overnight using a syringe pump to a solution of $Rh_2(OAc)_4$ (1.0 mol%, 22 mg) in phenylacetylene (3.0 eq, 15 mmol, 1.5 g). Following the addition, the reaction was monitored by TLC and further equivalents of alkene and $Rh_2(OAc)_4$ were added until complete disappearance of the diazonium compound was observed. The crude product was flushed through a plug of celite with CH_2Cl_2 and purified by flash silica gel column chromatography with pentane/EtOAc = 9/1 to provide the title compound as a yellow solid. (739 mg, 3 mmol, 66%). The spectrua is similar with the reported literature.[9]

^{1}H NMR (400 MHz, $CDCl_3$) δ 7.52 (d, *J* = 7.4 Hz, 1H), 7.40 – 7.26 (m, 3H), 6.95 (s, 1H), 3.76 (s, 6H), 2.54 (s, 3H).

^{13}C NMR (101 MHz, $CDCl_3$) δ 220.5, 171.2, 140.3, 131.1, 130.5, 130.2, 126.2, 111.3, 96.9, 52.4, 31.9, 20.0.

HRMS (ESI) m/z calcd. for $C_{14}H_{14}O_4$ ($[M+H]^+$) 247.0965, found 247.0959

m. p.: 62 – 64 °C.

[back to Table of Contents]

dimethyl 2-(m-tolyl)cycloprop-2-ene-1,1-dicarboxylate (1d')

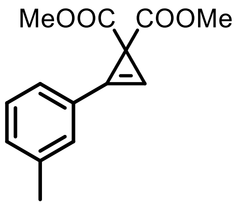

Starting material for Compound 12 was synthesised according to general procedure **GP-05**:

The desired diazonium compound (1.0 eq, 5 mmol, 800 mg) was dissolved in CH_2Cl_2 (0.5 M, 10 mL) and added dropwise overnight using a syringe pump to a solution of $Rh_2(OAc)_4$ (1.0 mol%, 22 mg) in 1-ethynyl-3-methylbenzene (3.0 eq, 15 mmol). Following the addition, the reaction was monitored by TLC and further equivalents of alkene and $Rh_2(OAc)_4$ were added until complete disappearance of the diazonium compound was observed. The crude product was flushed through a plug of celite with CH_2Cl_2 and purified by flash silica gel column chromatography with pentane/EtOAc = 9/1 to provide the title compound as a yellow solid. (700 mg, 57%). The spectrua is similar with the reported literature.[10]

^{1}H NMR (400 MHz, $CDCl_3$) δ 7.46 (d, *J* = 6.2 Hz, 2H), 7.35 (t, *J* = 7.8 Hz, 1H), 7.27 (d, *J* = 7.8 Hz, 1H), 6.88 (s, 1H), 3.76 (s, 6H), 2.41 (s, 3H).

^{13}C NMR (101 MHz, $CDCl_3$) δ 171.2, 138.7, 131.5, 130.8, 128.8, 127.5, 123.8, 112.3, 94.9, 52.4, 32.8, 21.2.

HRMS (ESI) m/z calcd. for $C_{14}H_{14}O_4$ ($[M+Na]^+$) 269.0784, found 269.0788

m. p.: 63 – 65 °C.

[back to Table of Contents]

dimethyl 2-(4-fluorophenyl)cycloprop-2-ene-1,1-dicarboxylate (1e')

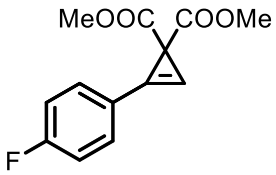

Starting material for Compound 12 was synthesised according to general procedure GP-05:

The desired diazonium compound (1.0 eq, 5 mmol, 800 mg) was dissolved in CH_2Cl_2 (0.5 M, 10 mL) and added dropwise overnight using a syringe pump to a solution of $Rh_2(OAc)_4$ (1.0 mol%, 22 mg) in 1-ethynyl-4-fluorobenzene (3.0 eq, 15 mmol). Following the addition, the reaction was monitored by TLC and further equivalents of alkene and $Rh_2(OAc)_4$ were added until complete disappearance of the diazonium compound was observed. The crude product was flushed through a plug of celite with CH_2Cl_2 and purified by flash silica gel column chromatography with pentane/EtOAc = 9/1 to provide the title compound as a yellow solid. (775 mg, 62%). The spectrua is similar with the reported literature.[11]

^{1}H NMR (400 MHz, $CDCl_3$) δ 7.69 – 7.57 (m, 2H), 7.19 – 7.06 (m, 2H), 6.86 (s, 1H), 3.73 (s, 6H).

^{13}C NMR (101 MHz, $CDCl_3$) δ 171.0, 165.2, 162.7, 135.2, 132.5, 132.4, 120.3, 120.3, 116.4, 116.1, 111.4, 94.9, 94.8, 52.4, 32.9.

^{19}F NMR (377 MHz, $CDCl_3$) δ -108.09.

HRMS (ESI) m/z calcd. for $C_{13}H_{11}FO_4$ ($[M+H]^+$) 251.0714, found 251.0718

m. p.: 62 – 64 °C.

[back to Table of Contents]

dimethyl 2-(4-chlorophenyl)cycloprop-2-ene-1,1-dicarboxylate (1f')

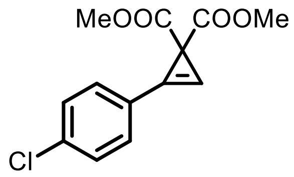

Starting material for Compound 12 was synthesised according to general procedure **GP-05**:

The desired diazonium compound (1.0 eq, 5 mmol, 800 mg) was dissolved in CH_2Cl_2 (0.5 M, 10 mL) and added dropwise overnight using a syringe pump to a solution of $Rh_2(OAc)_4$ (1.0 mol%, 22 mg) in 1-chloro-4-ethynylbenzene (3.0 eq, 15 mmol). Following the addition, the reaction was monitored by TLC and further equivalents of alkene and $Rh_2(OAc)_4$ were added until complete disappearance of the diazonium compound was observed. The crude product was flushed through a plug of celite with CH_2Cl_2 and purified by flash silica gel column chromatography with pentane/EtOAc = 9/1 to provide the title compound as a yellow solid. (784 mg, 59%). The spectrua is similar with the reported literature.[12]

^{1}H NMR (400 MHz, $CDCl_3$) δ 7.60 – 7.52 (m, 2H), 7.46 – 7.37 (m, 2H), 6.92 (s, 1H), 3.73 (s, 6H).

^{13}C NMR (101 MHz, $CDCl_3$) δ 170.9, 136.8, 131.5, 129.3, 122.5, 111.4, 96.0, 52.5, 32.8.

HRMS (ESI) m/z calcd. for $C_{13}H_{11}ClO_4$ ($[M+H]^+$) 267.0419, found 267.0419

m. p.: 65 – 67°C.

[back to Table of Contents]

dimethyl 2-(4-(trifluoromethyl)phenyl)cycloprop-2-ene-1,1-dicarboxylate (1g')

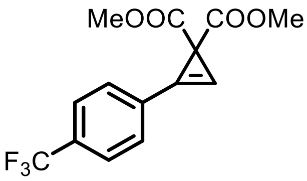

Starting material for Compound 12 was synthesised according to general procedure GP-05:

The desired diazonium compound (1.0 eq, 5 mmol, 800 mg) was dissolved in CH_2Cl_2 (0.5 M, 10 mL) and added dropwise overnight using a syringe pump to a solution of $Rh_2(OAc)_4$ (1.0 mol%, 22 mg) in 1-ethynyl-4-(trifluoromethyl)benzene (3.0 eq, 15 mmol). Following the addition, the reaction was monitored by TLC and further equivalents of alkene and $Rh_2(OAc)_4$ were added until complete disappearance of the diazonium compound was observed. The crude product was flushed through a plug of celite with CH_2Cl_2 and purified by flash silica gel column chromatography with pentane/EtOAc = 9/1 to provide the title compound as a yellow solid. (900 mg, 65%). The spectrua is similar with the reported literature.[13]

^{1}H NMR (400 MHz, $CDCl_3$) δ 7.73 (m, 4H), 7.06 (s, 1H), 3.75 (s, 6H).

^{13}C NMR (101 MHz, $CDCl_3$) δ 170.6, 130.5, 127.4, 125.9, 125.9, 125.9, 125.8, 111.5, 98.3, 52.5, 32.9.

^{19}F NMR (377 MHz, $CDCl_3$) δ -62.82.

HRMS (ESI) m/z calcd. for $C_{14}H_{11}F_3O_4$ ($[M+H]^+$) 301.0682, found 301.0684

m. p.: 82 – 84 °C.

[back to Table of Contents]

dimethyl 2-(4-(tert-butyl)phenyl)cycloprop-2-ene-1,1-dicarboxylate (1h')

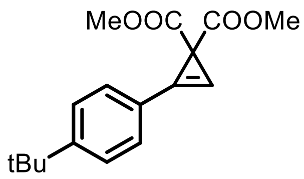

Starting material for Compound 12 was synthesised according to general procedure **GP-05**:

The desired diazonium compound (1.0 eq, 5 mmol, 800 mg) was dissolved in CH_2Cl_2 (0.5 M, 10 mL) and added dropwise overnight using a syringe pump to a solution of $Rh_2(OAc)_4$ (1.0 mol%, 22 mg) in 1-(tert-butyl)-4-ethynylbenzene (3.0 eq, 15 mmol). Following the addition, the reaction was monitored by TLC and further equivalents of alkene and $Rh_2(OAc)_4$ were added until complete disappearance of the diazonium compound was observed. The crude product was flushed through a plug of celite with CH_2Cl_2 and purified by flash silica gel column chromatography with pentane/EtOAc = 9/1 to provide the title compound as a yellow solid. (864 mg, 67%). The spectra are consistent with the ones reported in the literature.[14]

^{1}H NMR (400 MHz, $CDCl_3$) δ 7.60 – 7.52 (m, 2H), 7.52 – 7.41 (m, 2H), 6.82 (s, 1H), 3.73 (s, 6H), 1.33 (s, 9H).

^{13}C NMR (101 MHz, $CDCl_3$) δ 171.3, 154.1, 130.1, 125.9, 121.0, 112.0, 94.1, 52.3, 35.0, 31.1.

HRMS (ESI) m/z calcd. for $C_{17}H_{20}O_4$ ($[M+H]^+$) 288.0784, found 288.0780

m. p.: 85 – 87 °C.

[back to Table of Contents]

diethyl 2-phenylcycloprop-2-ene-1,1-dicarboxylate (1i')

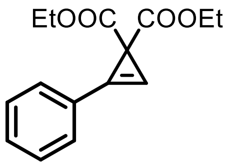

Starting material for Compound 12 was synthesised according to general procedure **GP-05**:

The desired diazonium compound[15] (1.0 eq, 5 mmol, 930 mg) was dissolved in CH_2Cl_2 (0.5 M, 10 mL) and added dropwise overnight using a syringe pump to a solution of $Rh_2(OAc)_4$ (1.0 mol%, 22 mg) in 1-(tert-butyl)-4-ethynylbenzene (3.0 eq, 15 mmol). Following the addition, the reaction was monitored by TLC and further equivalents of alkene and $Rh_2(OAc)_4$ were added until complete disappearance of the diazonium compound was observed. The crude product was flushed through a plug of celite with CH_2Cl_2 and purified by flash silica gel column chromatography with pentane/EtOAc = 9/1 to provide the title compound as a yellow solid. (832 mg, 64%). The spectra are consistent with the ones reported in the literature.[11]

^{1}H NMR (400 MHz, $CDCl_3$) δ 7.67 – 7.59 (m, 2H), 7.43 (m, 3H), 6.89 (s, 1H), 4.20 (q, *J* = 7.2 Hz, 4H), 1.25 (t, *J* = 7.1 Hz, 6H).

^{13}C NMR (101 MHz, $CDCl_3$) δ 170.8, 130.4, 130.3, 128.8, 124.2, 112.5, 95.4, 61.2, 33.3, 14.1.

HRMS (ESI) m/z calcd. for $C_{15}H_{16}O_4$ ($[M+H]^+$) 261.1121, found 261.1115

m. p.: 70 – 72 °C.

[back to Table of Contents]

dibenzyl 2-phenylcycloprop-2-ene-1,1-dicarboxylate (1j')

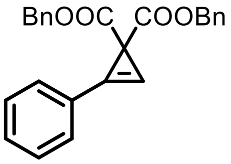

Starting material for Compound 12 was synthesised according to general procedure **GP-05**:

The desired diazonium compound[16] (1.0 eq, 5 mmol, 1550 mg) was dissolved in CH_2Cl_2 (0.5 M, 10 mL) and added dropwise overnight using a syringe pump to a solution of $Rh_2(OAc)_4$ (1.0 mol%, 22 mg) in 1-(tert-butyl)-4-ethynylbenzene (3.0 eq, 15 mmol). Following the addition, the reaction was monitored by TLC and further equivalents of alkene and $Rh_2(OAc)_4$ were added until complete disappearance of the diazonium compound was observed. The crude product was flushed through a plug of celite with CH_2Cl_2 and purified by flash silica gel column chromatography with pentane/EtOAc = 9/1 to provide the title compound as a yellow solid. (1190 mg, 62%). The spectra are consistent with the ones reported in the literature.[17]

^{1}H NMR (400 MHz, $CDCl_3$) δ 7.66 (m, 2H), 7.46 (m, 3H), 7.32 (t, *J* = 1.9 Hz, 10H), 6.95 (s, 1H), 5.23 (s, 4H).

^{13}C NMR (101 MHz, $CDCl_3$) δ 170.5, 135.8, 130.6, 130.4, 129.1, 128.9, 128.4, 128.3, 128.0, 127.9, 127.9, 127.8, 127.7, 123.9, 112.4, 95.1, 66.9, 33.3.

HRMS (ESI) m/z calcd. for $C_{25}H_{20}O_4$ ($[M+H]^+$) 385.1434, found 385.1441

m. p.: 100 – 102 °C.

[back to Table of Contents]

trimethyl 3-phenylcycloprop-2-ene-1,1,2-tricarboxylate (1a)

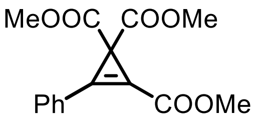

Compound **1a** was synthesised according to general procedure **GP-06**:

LiHMDS (1.0 M in THF, 1.0 eq, 3 mL) was added dropwise to a stirred solution of the starting material for compound **1a'** (1.0 eq, 3.0 mmol) and methyl chloroformate (1.5 eq, 4.5 mmol) at −78 °C in THF (0.3 M, 10 mL). The solution was stirred at −78 °C until completion was observed by TLC. The reaction was quenched with sat. aq. NH_4Cl and the aqueous phase was extracted with Et_2O (3 x 20 ml). The combined organic extracts were washed with brine then dried over Na_2SO_4, filtered and concentrated in vacuo. The crude product was purified by silica gel column chromatography (pentane/Et_2O =9/1) to provide the title compound **1a** as a yellow solid (461 mg, 1.6 mmol, 53%)

The spectra are consistent with the ones reported in the literature[18]

^{1}H NMR (400 MHz, $CDCl_3$) δ 7.89 – 7.43 (m, 5H), 3.93 (s, 3H), 3.76 (s, 6H).

^{13}C NMR (101 MHz, $CDCl_3$) δ 168.9, 157.9, 132.6, 132.2, 129.1, 122.9, 120.5, 98.2, 52.9, 52.7, 32.8.

HRMS (ESI) m/z calcd. for $C_{15}H_{14}O_6$ ($[M+Na]^+$) 313.0683, found 313.0683

m. p.: 64 – 66 °C.

[back to Table of Contents]

trimethyl 3-(p-tolyl)cycloprop-2-ene-1,1,2-tricarboxylate (1b)

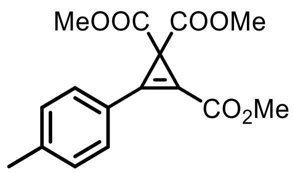

Compound **1b** was synthesised according to general procedure **GP-06**:

LiHMDS (1.0 M in THF, 1.0 eq, 1.5 mL) was added dropwise to a stirred solution of the starting material for **1b'** (1.0 eq, 1.5 mmol) and methyl chloroformate (1.5 eq, 2.25 mmol) at −78 °C in THF (0.3 M, 4.5 mL). The solution was stirred at −78 °C until completion was observed by TLC. The reaction was quenched with sat. aq. NH_4Cl and the aqueous phase was extracted with Et_2O (3 x 20 ml). The combined organic extracts were washed with brine then dried over Na_2SO_4, filtered and concentrated in vacuo. The crude product was purified by silica gel column chromatography (pentane/Et_2O =9/1) to provide the title compound **1b** as a yellow solid (243 mg, 0.8 mmol, 53%)

^{1}H NMR (400 MHz, $CDCl_3$) δ 7.74 – 7.67 (m, 2H), 7.31 (s, 2H), 3.92 (s, 3H), 3.74 (s, 6H), 2.43 (s, 3H).

^{13}C NMR (101 MHz, $CDCl_3$) δ 169.0, 143.7, 132.3, 129.9, 52.8, 52.6, 21.9.

HRMS (ESI) m/z calcd. for $C_{16}H_{16}O_6$ ([M+H] $^+$) 305.1020, found 305.1013

m. p.: 65 – 67 °C.

[back to Table of Contents]

trimethyl 3-(o-tolyl)cycloprop-2-ene-1,1,2-tricarboxylate (1c)

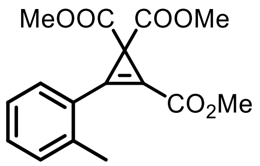

Compound **1c** was synthesised according to general procedure **GP-06**:

LiHMDS (1.0 M in THF, 1.0 eq, 1.5 mL) was added dropwise to a stirred solution of the starting material for **1c'** (1.0 eq, 1.5 mmol) and methyl chloroformate (1.5 eq, 2.25 mmol) at −78 °C in THF (0.3 M, 4.5 mL). The solution was stirred at −78 °C until completion was observed by TLC. The reaction was quenched with sat. aq. NH_4Cl and the aqueous phase was extracted with Et_2O (3 x 20 ml). The combined organic extracts were washed with brine then dried over Na_2SO_4, filtered and concentrated in vacuo. The crude product was purified by silica gel column chromatography (pentane/Et_2O =9/1) to provide the title compound **1c** as a yellow solid (263 mg, 0.87 mmol, 58%)

^{1}H NMR (400 MHz, $CDCl_3$) δ 7.63 (d, *J* = 7.6 Hz, 1H), 7.43 (t, *J* = 7.5 Hz, 1H), 7.35 – 7.24 (m, 2H), 3.91 (s, 3H), 3.75 (s, 6H), 2.68 (s, 3H).

^{13}C NMR (101 MHz, $CDCl_3$) δ 169.2, 158.2, 142.1, 133.6, 132.8, 130.8, 126.3, 122.4, 118.4, 97.8, 52.9, 52.7, 20.4.

HRMS (ESI) m/z calcd. for $C_{16}H_{16}O_6$ ([M+H] $^+$) 305.1020, found 305.1016

m. p.: 64 – 66 °C.

[back to Table of Contents]

trimethyl 3-(m-tolyl)cycloprop-2-ene-1,1,2-tricarboxylate (1d)

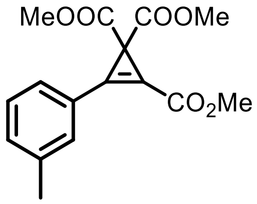

Compound **1d** was synthesised according to general procedure **GP-06**:

LiHMDS (1.0 M in THF, 1.0 eq, 1.5 mL) was added dropwise to a stirred solution of the starting material for **1d'** (1.0 eq, 1.5 mmol) and methyl chloroformate (1.5 eq, 2.25 mmol) at −78 °C in THF (0.3 M, 4.5 mL). The solution was stirred at −78 °C until completion was observed by TLC. The reaction was quenched with sat. aq. NH_4Cl and the aqueous phase was extracted with Et_2O (3 x 20 ml). The combined organic extracts were washed with brine then dried over Na_2SO_4, filtered and concentrated in vacuo. The crude product was purified by silica gel column chromatography (pentane/Et_2O =9/1) to provide the title compound **1d** as a yellow solid (253 mg, 0.85 mmol, 57%)

^{1}H NMR (400 MHz, $CDCl_3$) δ 7.62 (m, 2H), 7.43 – 7.31 (m, 2H), 3.93 (s, 3H), 3.75 (s, 6H), 2.41 (s, 3H).

^{13}C NMR (101 MHz, $CDCl_3$) δ 169.0, 139.1, 133.6, 132.5, 129.5, 129.0, 122.8, 97.9, 52.9, 52.7, 21.2.

HRMS (ESI) m/z calcd. for $C_{16}H_{16}O_6$ ($[M+H]^+$) 305.1020, found 305.1023

m. p.: 64 – 66 °C.

[back to Table of Contents]

trimethyl 3-(4-fluorophenyl)cycloprop-2-ene-1,1,2-tricarboxylate (1e)

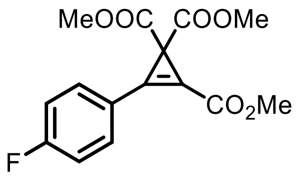

Compound **1e** was synthesised according to general procedure GP-06:

LiHMDS (1.0 M in THF, 1.0 eq, 1.5 mL) was added dropwise to a stirred solution of the starting material for **1e’** (1.0 eq, 1.5 mmol) and methyl chloroformate (1.5 eq, 2.25 mmol) at −78 °C in THF (0.3 M, 4.5 mL). The solution was stirred at −78 °C until completion was observed by TLC. The reaction was quenched with sat. aq. NH_4Cl and the aqueous phase was extracted with Et_2O (3 x 20 ml). The combined organic extracts were washed with brine then dried over Na_2SO_4, filtered and concentrated in vacuo. The crude product was purified by silica gel column chromatography (pentane/Et_2O =9/1) to provide the title compound **1e** as a yellow solid (277 mg, 0.9 mmol, 60%)

^{1}H NMR (400 MHz, $CDCl_3$) δ 7.83 (m, 2H), 7.19 (m, 2H), 3.93 (s, 3H), 3.75 (s, 6H).

^{13}C NMR (101 MHz, $CDCl_3$) δ 168.8, 166.4, 163.9, 157.9, 134.7, 134.6, 119.6, 119.4, 119.4, 116.8, 116.6, 97.9, 97.9, 52.9, 52.8, 36.5.

^{19}F NMR (377 MHz, $CDCl_3$) δ -103.88.

HRMS (ESI) m/z calcd. for $C_{15}H_{13}FO_6$ ([M+Na] $^+$) 331.0588, found 331.0594

m. p.: 65 – 67 °C.

[back to Table of Contents]

trimethyl 3-(4-chlorophenyl)cycloprop-2-ene-1,1,2-tricarboxylate (1f)

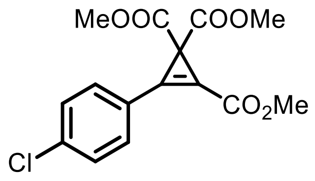

Compound **1f** was synthesised according to general procedure **GP-06**:

LiHMDS (1.0 M in THF, 1.0 eq, 1.5 mL) was added dropwise to a stirred solution of the starting material for **1f'** (1.0 eq, 1.5 mmol) and methyl chloroformate (1.5 eq, 2.25 mmol) at −78 °C in THF (0.3 M, 4.5 mL). The solution was stirred at −78 °C until completion was observed by TLC. The reaction was quenched with sat. aq. NH_4Cl and the aqueous phase was extracted with Et_2O (3 x 20 ml). The combined organic extracts were washed with brine then dried over Na_2SO_4, filtered and concentrated in vacuo. The crude product was purified by silica gel column chromatography (pentane/Et_2O =9/1) to provide the title compound **1f** as a yellow solid (259 mg, 0.8 mmol, 53%)

^{1}H NMR (400 MHz, $CDCl_3$) δ 7.75 (m, 2H), 7.48 (m, 2H), 3.93 (s, 3H), 3.76 (s, 6H).

^{13}C NMR (101 MHz, $CDCl_3$) δ 168.7, 139.1, 133.3, 129.6, 121.4, 119.5, 98.9, 53.0, 52.8.

HRMS (ESI) m/z calcd. for $C_{15}H_{13}ClO_6$ ($[M+H]^+$) 325.0473, found 325.0476

m. p.: 67 – 69 °C.

[back to Table of Contents]

trimethyl 3-(4-(trifluoromethyl)phenyl)cycloprop-2-ene-1,1,2-tricarboxylate (1g)

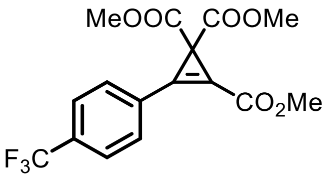

Compound **1g** was synthesised according to general procedure **GP-06**:

LiHMDS (1.0 M in THF, 1.0 eq, 1.5 mL) was added dropwise to a stirred solution of the starting material for **1g'** (1.0 eq, 1.5 mmol) and methyl chloroformate (1.5 eq, 2.25 mmol) at −78 °C in THF (0.3 M, 4.5 mL). The solution was stirred at −78 °C until completion was observed by TLC. The reaction was quenched with sat. aq. NH_4Cl and the aqueous phase was extracted with Et_2O (3 x 20 ml). The combined organic extracts were washed with brine then dried over Na_2SO_4, filtered and concentrated in vacuo. The crude product was purified by silica gel column chromatography (pentane/Et_2O =9/1) to provide the title compound **1g** as a yellow solid (304 mg, 0.85 mmol, 57%)

^{1}H NMR (400 MHz, $CDCl_3$) δ 7.97 – 7.89 (m, 2H), 7.79 – 7.65 (m, 2H), 3.95 (s, 3H), 3.77 (s, 6H).

^{13}C NMR (101 MHz, $CDCl_3$) δ 168.5, 157.5, 134.0, 133.6, 132.3, 126.2, 126.1, 126.1, 126.0, 124.8, 119.2, 101.1, 53.2, 52.9, 36.6.

^{19}F NMR (376 MHz, $CDCl_3$) δ -63.13.

HRMS (ESI) m/z calcd. for $C_{16}H_{13}F_3O_6$ ([M+H] $^+$) 359.0753, found 359.0759

m. p.: 72 – 74 °C.

[*back to Table of Contents*]

trimethyl 3-(4-(tert-butyl)phenyl)cycloprop-2-ene-1,1,2-tricarboxylate (1h)

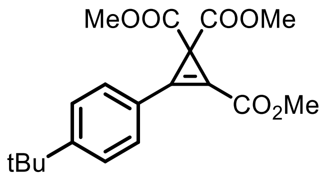

Compound **1h** was synthesised according to general procedure **GP-06**:

LiHMDS (1.0 M in THF, 1.0 eq, 1.5 mL) was added dropwise to a stirred solution of the starting material for **1h'** (1.0 eq, 1.5 mmol) and methyl chloroformate (1.5 eq, 2.25 mmol) at −78 °C in THF (0.3 M, 4.5 mL). The solution was stirred at −78 °C until completion was observed by TLC. The reaction was quenched with sat. aq. NH_4Cl and the aqueous phase was extracted with Et_2O (3 x 20 ml). The combined organic extracts were washed with brine then dried over Na_2SO_4, filtered and concentrated in vacuo. The crude product was purified by silica gel column chromatography (pentane/Et_2O =9/1) to provide the title compound **1h** as a yellow solid (284 mg, 0.82 mmol, 55%)

^{1}H NMR (400 MHz, $CDCl_3$) δ 7.78 – 7.71 (m, 2H), 7.52 (m, 2H), 3.92 (s, 3H), 3.75 (s, 6H), 1.33 (s, 9H).

^{13}C NMR (101 MHz, $CDCl_3$) δ 219.9, 169.0, 158.1, 156.6, 132.1, 126.2, 120.4, 120.1, 97.1, 52.8, 52.7, 36.3, 35.3, 31.0.

HRMS (ESI) m/z calcd. for $C_{19}H_{22}O_6$ ([M+Na] $^+$) 369.1309, found 369.1301

m. p.: 80 – 82 °C.

[back to Table of Contents]

1,1-diethyl 2-methyl 3-phenylcycloprop-2-ene-1,1,2-tricarboxylate (1i)

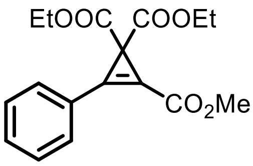

Compound **1i** was synthesised according to general procedure **GP-06**:

LiHMDS (1.0 M in THF, 1.0 eq, 1.5 mL) was added dropwise to a stirred solution of the starting material for **1i'** (1.0 eq, 1.5 mmol) and methyl chloroformate (1.5 eq, 2.25 mmol) at −78 °C in THF (0.3 M, 4.5 mL). The solution was stirred at −78 °C until completion was observed by TLC. The reaction was quenched with sat. aq. NH_4Cl and the aqueous phase was extracted with Et_2O (3 x 20 ml). The combined organic extracts were washed with brine then dried over Na_2SO_4, filtered and concentrated in vacuo. The crude product was purified by silica gel column chromatography (pentane/Et_2O =9/1) to provide the title compound **1i** as a yellow solid (246 mg, 0.79 mmol, 53%)

^{1}H NMR (400 MHz, $CDCl_3$) δ 7.83 (m, 2H), 7.58 – 7.45 (m, 3H), 4.22 (m, 4H), 3.93 (s, 3H), 1.26 (t, *J* = 7.1 Hz, 6H).

^{13}C NMR (101 MHz, $CDCl_3$) δ 168.6, 132.4, 132.2, 129.1, 123.2, 120.8, 98.5, 61.6, 52.8, 14.1.

HRMS (ESI) m/z calcd. for $C_{17}H_{18}O_6$ ([M+H] $^+$) 319.1176, found 319.1179

m. p.: 80 – 82 °C.

[back to Table of Contents]

1,1-dibenzyl 2-methyl 3-phenylcycloprop-2-ene-1,1,2-tricarboxylate (1j)

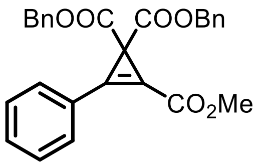

Compound **1j** was synthesised according to general procedure **GP-06**:
LiHMDS (1.0 M in THF, 1.0 eq, 1.5 mL) was added dropwise to a stirred solution of the starting material for **1j'** (1.0 eq, 1.5 mmol) and methyl chloroformate (1.5 eq, 2.25 mmol) at −78 °C in THF (0.3 M, 4.5 mL). The solution was stirred at −78 °C until completion was observed by TLC. The reaction was quenched with sat. aq. NH_4Cl and the aqueous phase was extracted with Et_2O (3 x 20 ml). The combined organic extracts were washed with brine then dried over Na_2SO_4, filtered and concentrated in vacuo. The crude product was purified by silica gel column chromatography (pentane/Et_2O =9/1) to provide the title compound **1j** as a yellow solid (309 mg, 0.7 mmol, 47%)

^{1}H NMR (400 MHz, $CDCl_3$) δ 7.81 – 7.74 (m, 2H), 7.53 – 7.42 (m, 3H), 7.31 – 7.22 (m, 10H), 5.20 (s, 4H), 3.89 (s, 3H).

^{13}C NMR (101 MHz, $CDCl_3$) δ 168.3, 157.9, 135.6, 132.6, 132.2, 129.1, 128.4, 128.0, 127.7, 122.9, 120.6, 98.2, 67.1, 52.90, 37.0.

HRMS (ESI) m/z calcd. for $C_{27}H_{22}O_6$ ([M+H] $^+$) 443.1489, found 443.148

m. p.: 100 – 102 °C.

[back to Table of Contents]

2-butyl 1,1-dimethyl 3-phenylcycloprop-2-ene-1,1,2-tricarboxylate (1k)

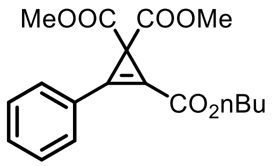

Compound **1k** was synthesised according to general procedure **GP-06**:
LiHMDS (1.0 M in THF, 1.0 eq, 1.5 mL) was added dropwise to a stirred solution of the starting material for **1a'** (1.0 eq, 1.5 mmol) and butyl chloroformate (1.5 eq, 2.25 mmol) at −78 °C in THF (0.3 M, 4.5 mL). The solution was stirred at −78 °C until completion was observed by TLC. The reaction was quenched with sat. aq. NH_4Cl and the aqueous phase was extracted with Et_2O (3 x 20 ml). The combined organic extracts were washed with brine then dried over Na_2SO_4, filtered and concentrated in vacuo. The crude product was purified by silica gel column chromatography (pentane/Et_2O =9/1) to provide the title compound **1k** as a yellow solid (264 mg, 0.8 mmol, 53%)

^{1}H NMR (400 MHz, $CDCl_3$) δ 7.84 – 7.77 (m, 2H), 7.58 – 7.45 (m, 3H), 4.33 (t, *J* = 6.6 Hz, 2H), 3.75 (s, 6H), 1.80 – 1.69 (m, 2H), 1.52 – 1.38 (m, 2H), 0.97 (t, *J* = 7.4 Hz, 3H).

^{13}C NMR (101 MHz, $CDCl_3$) δ 169.0, 157.6, 132.5, 132.2, 129.1, 123.0, 119.9, 98.6, 52.6, 30.5, 19.0, 13.7.

HRMS (ESI) m/z calcd. for $C_{18}H_{20}O_6$ ($[M+H]^+$) 333.1333, found 333.1339

m. p.: 80 – 82 °C.

[back to Table of Contents]

2-hexyl 1,1-dimethyl 3-phenylcycloprop-2-ene-1,1,2-tricarboxylate (1l)

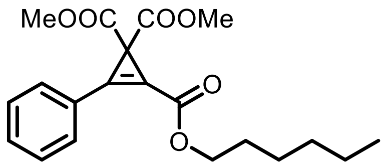

Compound **1l** was synthesised according to general procedure **GP-06**:

LiHMDS (1.0 M in THF, 1.0 eq, 1.5 mL) was added dropwise to a stirred solution of the starting material for **1a'** (1.0 eq, 1.5 mmol) and hexyl chloroformate (1.5 eq, 2.25 mmol) at −78 °C in THF (0.3 M, 4.5 mL). The solution was stirred at −78 °C until completion was observed by TLC. The reaction was quenched with sat. aq. NH_4Cl and the aqueous phase was extracted with Et_2O (3 x 20 ml). The combined organic extracts were washed with brine then dried over Na_2SO_4, filtered and concentrated in vacuo. The crude product was purified by silica gel column chromatography (pentane/Et_2O =9/1) to provide the title compound **1l** as a yellow solid (252 mg, 0.7 mmol, 47%)

^{1}H NMR (400 MHz, $CDCl_3$) δ 7.84 – 7.77 (m, 2H), 7.58 – 7.45 (m, 3H), 4.32 (t, *J* = 6.7 Hz, 2H), 3.75 (s, 6H), 1.81 – 1.70 (m, 2H), 1.38 (m, 6H), 0.96 – 0.88 (m, 3H).

^{13}C NMR (101 MHz, $CDCl_3$) δ 169.0, 157.6, 132.5, 132.2, 129.1, 123.0, 119.9, 98.6, 66.2, 52.6, 31.3, 28.4, 25.4, 22.5, 13.9.

HRMS (ESI) m/z calcd. for $C_{20}H_{24}O_6$ ($[M+H]^+$) 361.1646, found 361.1662

m. p.: 80 – 82 °C.

[back to Table of Contents]

2-isobutyl 1,1-dimethyl 3-phenylcycloprop-2-ene-1,1,2-tricarboxylate (1m)

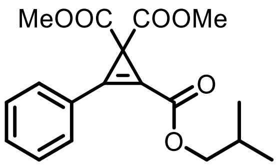

Compound **1m** was synthesised according to general procedure **GP-06**:
LiHMDS (1.0 M in THF, 1.0 eq, 1.5 mL) was added dropwise to a stirred solution of the starting material for **1a'** (1.0 eq, 1.5 mmol) and isobutyl chloroformate (1.5 eq, 2.25 mmol) at −78 °C in THF (0.3 M, 4.5 mL). The solution was stirred at −78 °C until completion was observed by TLC. The reaction was quenched with sat. aq. NH_4Cl and the aqueous phase was extracted with Et_2O (3 x 20 ml). The combined organic extracts were washed with brine then dried over Na_2SO_4, filtered and concentrated in vacuo. The crude product was purified by silica gel column chromatography (pentane/Et_2O =9/1) to provide the title compound **1m** as a yellow solid (200 mg, 0.6 mmol, 40%)

^{1}H NMR (400 MHz, $CDCl_3$) δ 7.84 – 7.77 (m, 2H), 7.58 – 7.45 (m, 3H), 4.11 (d, *J* = 6.6 Hz, 2H), 3.75 (s, 6H), 2.07 (m, 1H), 1.00 (d, *J* = 6.7 Hz, 6H).

^{13}C NMR (101 MHz, $CDCl_3$) δ 169.0, 157.5, 132.5, 132.1, 129.1, 123.0, 119.9, 98.6, 71.9, 52.6, 36.4, 27.7, 18.9.

HRMS (ESI) m/z calcd. for $C_{18}H_{20}O_6$ ([M+H] $^+$) 333.1333, found 333.1337

m. p.: 64 – 66 °C.

[back to Table of Contents]

1,1-dimethyl 2-(prop-2-yn-1-yl) 3-phenylcycloprop-2-ene-1,1,2-tricarboxylate (1n)

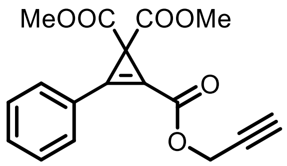

Compound **1n** was synthesised according to general procedure **GP-06**:

LiHMDS (1.0 M in THF, 1.0 eq, 1.5 mL) was added dropwise to a stirred solution of the starting material for **1a'** (1.0 eq, 1.5 mmol) and propargylchloroformate (1.5 eq, 2.25 mmol) at −78 °C in THF (0.3 M, 4.5 mL). The solution was stirred at −78 °C until completion was observed by TLC. The reaction was quenched with sat. aq. NH_4Cl and the aqueous phase was extracted with Et_2O (3 x 20 ml). The combined organic extracts were washed with brine then dried over Na_2SO_4, filtered and concentrated in vacuo. The crude product was purified by silica gel column chromatography (pentane/Et_2O =9/1) to provide the title compound **1n** as a yellow solid (219 mg, 0.7 mmol, 46%)

^{1}H NMR (400 MHz, $CDCl_3$) δ 7.77 – 7.70 (m, 2H), 7.56 – 7.36 (m, 3H), 4.82 (d, *J* = 2.4 Hz, 2H), 3.66 (s, 6H), 2.45 (t, *J* = 2.5 Hz, 1H).

^{13}C NMR (101 MHz, $CDCl_3$) δ 168.7, 156.7, 132.8, 132.4, 129.2, 122.8, 121.5, 97.6, 75.7, 53.3, 52.8, 36.4.

HRMS (ESI) m/z calcd. for $C_{17}H_{14}O_6$ ([M+H] $^+$) 315.0863, found 315.0866

m. p.: 64 – 66 °C.

[back to Table of Contents]

2-benzyl 1,1-dimethyl 3-phenylcycloprop-2-ene-1,1,2-tricarboxylate (1o)

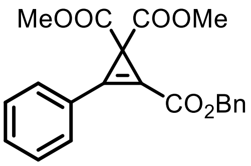

Compound **1o** was synthesised according to general procedure **GP-06**:

LiHMDS (1.0 M in THF, 1.0 eq, 1.5 mL) was added dropwise to a stirred solution of the starting material for **1a'** (1.0 eq, 1.5 mmol) and benzyl chloroformate (1.5 eq, 2.25 mmol) at −78 °C in THF (0.3 M, 4.5 mL). The solution was stirred at −78 °C until completion was observed by TLC. The reaction was quenched with sat. aq. NH_4Cl and the aqueous phase was extracted with Et_2O (3 x 20 ml). The combined organic extracts were washed with brine then dried over Na_2SO_4, filtered and concentrated in vacuo. The crude product was purified by silica gel column chromatography (pentane/Et_2O =9/1) to provide the title compound **1o** as a yellow solid (311 mg, 0.85 mmol, 57%)

^{1}H NMR (400 MHz, $CDCl_3$) δ 7.84 – 7.76 (m, 2H), 7.58 – 7.29 (m, 8H), 5.37 (s, 2H), 3.74 (s, 6H).

^{13}C NMR (101 MHz, $CDCl_3$) δ 168.9, 135.2, 132.6, 132.3, 129.1, 128.6, 128.4, 128.1, 122.9, 120.6, 98.3, 67.5, 52.7.

HRMS (ESI) m/z calcd. for $C_{21}H_{18}O_6$ ([M+H] $^+$) 367.1176, found 367.1169

m. p.: 72 – 74 °C.

[back to Table of Contents]

2-ethyl 1,1-dimethyl 3-phenylcycloprop-2-ene-1,1,2-tricarboxylate (1p)

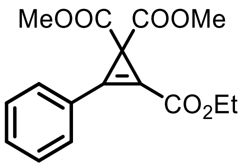

Compound **1p** was synthesised according to general procedure **GP-06**:

LiHMDS (1.0 M in THF, 1.0 eq, 1.5 mL) was added dropwise to a stirred solution of the starting material for **1a’** (1.0 eq, 1.5 mmol) and ethyl chloroformate (1.5 eq, 2.25 mmol) at −78 °C in THF (0.3 M, 4.5 mL). The solution was stirred at −78 °C until completion was observed by TLC. The reaction was quenched with sat. aq. NH_4Cl and the aqueous phase was extracted with Et_2O (3 x 20 ml). The combined organic extracts were washed with brine then dried over Na_2SO_4, filtered and concentrated in vacuo. The crude product was purified by silica gel column chromatography (pentane/Et_2O =9/1) to provide the title compound **1p** as a yellow solid (150 mg, 0.5 mmol, 32%)

^{1}H NMR (400 MHz, $CDCl_3$) δ 7.85 – 7.77 (m, 2H), 7.58 – 7.41 (m, 3H), 4.39 (q, *J* = 7.2 Hz, 2H), 3.75 (s, 6H), 1.40 (t, *J* = 7.1 Hz, 3H).

^{13}C NMR (101 MHz, $CDCl_3$) δ 169.0, 157.5, 132.5, 132.2, 129.1, 123.0, 119.9, 98.6, 62.2, 52.7, 14.2.

HRMS (ESI) m/z calcd. for $C_{16}H_{16}O_6$ ([M+H] $^+$) 305.1020, found 305.1033

m. p.: 60 – 62 °C.

[back to Table of Contents]

2-(2-methoxyethyl) 1,1-dimethyl 3-phenylcycloprop-2-ene-1,1,2-tricarboxylate (1q)

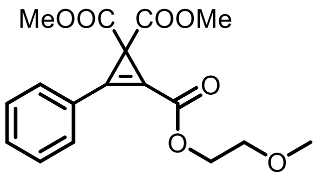

Compound **1q** was synthesised according to general procedure **GP-06**:

LiHMDS (1.0 M in THF, 1.0 eq, 1.5 mL) was added dropwise to a stirred solution of the starting material for **1a’** (1.0 eq, 1.5 mmol) and 2-methoxyethyl carbonochloridate (1.5 eq, 2.25 mmol) at −78 °C in THF (0.3 M, 4.5 mL). The solution was stirred at −78 °C until completion was observed by TLC. The reaction was quenched with sat. aq. NH_4Cl and the aqueous phase was extracted with Et_2O (3 x 20 ml). The combined organic extracts were washed with brine then dried over Na_2SO_4, filtered and concentrated in vacuo. The crude product was purified by silica gel column chromatography (pentane/Et_2O =9/1) to provide the title compound **1q** as a yellow solid (234 mg, 0.7 mmol, 47%)

^{1}H NMR (400 MHz, $CDCl_3$) δ 7.85 – 7.78 (m, 2H), 7.59 – 7.45 (m, 3H), 4.51 – 4.44 (m, 2H), 3.75 (s, 6H), 3.73 – 3.68 (m, 2H), 3.43 (s, 3H).

^{13}C NMR (101 MHz, $CDCl_3$) δ 168.9, 157.4, 132.6, 132.3, 129.1, 122.9, 120.4, 98.3, 70.1, 66.9, 65.0, 59.1, 52.7, 36.4.

HRMS (ESI) m/z calcd. for $C_{17}H_{18}O_7$ ([M+Na] $^+$) 357.0945, found 357.0949

m. p.: 60 – 62 °C.

[back to Table of Contents]

dimethyl 2-(benzylcarbamoyl)-3-phenylcycloprop-2-ene-1,1-dicarboxylate (1r)

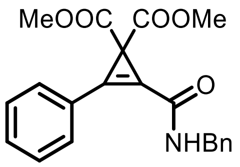

Compound **1r** was synthesised according to general procedure **GP-06**:

LiHMDS (1.0 M in THF, 1.0 eq, 1.5 mL) was added dropwise to a stirred solution of the starting material for **1a'** (1.0 eq, 1.5 mmol) and benzyl isocyanate (1.5 eq, 2.25 mmol) at −78 °C in THF (0.3 M, 4.5 mL). The solution was stirred at −78 °C until completion was observed by TLC. The reaction was quenched with sat. aq. NH_4Cl and the aqueous phase was extracted with Et_2O (3 x 20 ml). The combined organic extracts were washed with brine then dried over Na_2SO_4, filtered and concentrated in vacuo. The crude product was purified by silica gel column chromatography (pentane/Et_2O =9/1) to provide the title compound **1r** as a yellow solid (165 mg, 0.45 mmol, 30%)

^{1}H NMR (400 MHz, $CDCl_3$) δ 7.88 – 7.78 (m, 2H), 7.52 – 7.46 (m, 3H), 7.37 – 7.30 (m, 5H), 6.86 (s, 1H), 4.61 (d, *J* = 6.0 Hz, 2H), 3.74 (s, 6H).

^{13}C NMR (101 MHz, $CDCl_3$) δ 132.0, 131.9, 129.0, 128.8, 127.6, 52.8, 43.7.

HRMS (ESI) m/z calcd. for $C_{21}H_{19}NO_5$ ([M+H] $^+$) 366.1336, found 366.1340

m. p.: 80 – 82 °C.

[back to Table of Contents]

dimethyl 2-phenyl-3-(phenylcarbamoyl)cycloprop-2-ene-1,1-dicarboxylate (1s)

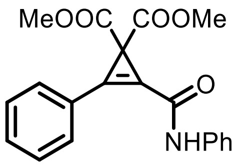

Compound **1s** was synthesised according to general procedure **GP-06**:

LiHMDS (1.0 M in THF, 1.0 eq, 1.5 mL) was added dropwise to a stirred solution of the starting material for **1a'** (1.0 eq, 1.5 mmol) and phenyl isocyanate (1.5 eq, 2.25 mmol) at −78 °C in THF (0.3 M, 4.5 mL). The solution was stirred at −78 °C until completion was observed by TLC. The reaction was quenched with sat. aq. NH_4Cl and the aqueous phase was extracted with Et_2O (3 x 20 ml). The combined organic extracts were washed with brine then dried over Na_2SO_4, filtered and concentrated in vacuo. The crude product was purified by silica gel column chromatography (pentane/Et_2O =9/1) to provide the title compound **1s** as a yellow solid (173 mg, 0.49 mmol, 33%)

^{1}H NMR (400 MHz, $CDCl_3$) δ 8.52 (s, 1H), 7.86 (d, *J* = 6.8 Hz, 2H), 7.65 (d, *J* = 8.0 Hz, 2H), 7.51 (d, *J* = 6.7 Hz, 3H), 7.37 (t, *J* = 7.7 Hz, 2H), 7.17 (t, *J* = 7.5 Hz, 1H), 3.81 (s, 6H).

^{13}C NMR (101 MHz, $CDCl_3$) δ 132.2, 131.9, 129.1, 125.0, 119.7, 53.0.

HRMS (ESI) m/z calcd. for $C_{20}H_{17}NO_5$ ($[M+H]^+$) 352.1180, found 352.1192

m. p.: 80 – 82 °C.

[back to Table of Contents]

dimethyl 2-(cyclohexylcarbamoyl)-3-phenylcycloprop-2-ene-1,1-dicarboxylate (1t)

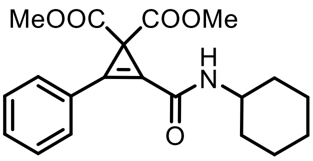

Compound **1t** was synthesised according to general procedure **GP-06**:

LiHMDS (1.0 M in THF, 1.5 eq, 1.5 mL) was added dropwise to a stirred solution of the starting material for **1a'** (1.0 eq, 1.0 mmol) and phenyl isocyanate (1.5 eq, 1.5 mmol) at −78 °C in THF (0.3 M, 3.4 mL). The solution was stirred at −78 °C until completion was observed by TLC. The reaction was quenched with sat. aq. NH_4Cl and the aqueous phase was extracted with Et_2O (3 x 20 ml). The combined organic extracts were washed with brine then dried over Na_2SO_4, filtered and concentrated in vacuo. The crude product was purified by silica gel column chromatography (pentane/EtOAc =7/3) to provide the title compound **1t** as a white solid (114 mg, 0.32 mmol, 32%).

^{1}H NMR (500 MHz, $CDCl_3$) δ 7.83 – 7.77 (m, 2H), 7.53 – 7.43 (m, 3H), 6.46 (d, *J* = 8.2 Hz, 1H), 3.96 – 3.87 (m, 1H), 3.75 (d, *J* = 1.0 Hz, 6H), 1.98 (dq, *J* = 12.1, 3.9 Hz, 2H), 1.73 (dq, *J* = 11.9, 3.9 Hz, 2H), 1.62 (dt, *J* = 11.5, 3.3 Hz, 1H), 1.47 – 1.34 (m, 2H), 1.26 (qd, *J* = 12.8, 6.1 Hz, 3H).

^{13}C NMR (126 MHz, $CDCl_3$) δ 170.0, 155.8, 132.0, 131.9 129.2, 123.5, 116.5, 101.6, 52.9, 48.8, 37.3, 32.9, 25.6, 24.8.

HRMS (ESI) m/z calcd. for $C_{20}H_{23}NO_5$ ([M+H] $^+$) 358.1649, found 358.1664

m. p.: 77 – 79 °C.

[back to Table of Contents]

dimethyl 2-((4-methoxyphenyl)carbamoyl)-3-phenylcycloprop-2-ene-1,1-dicarboxylate (1u)

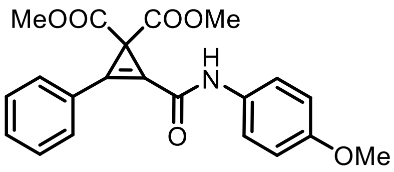

Compound **1u** was synthesised according to general procedure **GP-06**:

LiHMDS (1.0 M in THF, 1.5 eq, 1.5 mL) was added dropwise to a stirred solution of the starting material for **1a'** (1.0 eq, 1.0 mmol) and phenyl isocyanate (1.5 eq, 1.5 mmol) at −78 °C in THF (0.3 M, 3.4 mL). The solution was stirred at −78 °C until completion was observed by TLC. The reaction was quenched with sat. aq. NH_4Cl and the aqueous phase was extracted with Et_2O (3 x 20 ml). The combined organic extracts were washed with brine then dried over Na_2SO_4, filtered and concentrated in vacuo. The crude product was purified by silica gel column chromatography (pentane/EtOAc =7/3) to provide the title compound **1u** as a yellow solid (73 mg, 0.19 mmol, 19%).

^{1}H NMR (500 MHz, $CDCl_3$) δ 8.39 (s, 1H), 7.88 – 7.82 (m, 2H), 7.61 – 7.55 (m, 2H), 7.54 – 7.46 (m, 3H), 6.94 – 6.87 (m, 2H), 3.82 (s, 3H), 3.80 (s, 6H).

^{13}C NMR (126 MHz, C_6D_6) δ 170.2, 157.2, 154.3, 144.5, 132.1, 131.9, 131.5, 129.3, 124.1, 121.4, 114.5, 54.9, 52.4, 32.4, 29.9, 23.1, 14.4.

HRMS (ESI) m/z calcd. for C21H19NO6 ([M+H] $^+$) 382.1285, found 382.1295.

m. p.: 81 – 83 °C

[back to Table of Contents]

trimethyl (2S,3R)-3-phenyl-3-(propylthio)cyclopropane-1,1,2-tricarboxylate (3a)

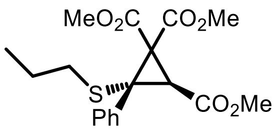

The BIMP catalysed reaction was carried with 0.1 mmol scale (1.0 eq, 29 mg) under room temperature and for 24 h with 10 mol% catalyst in 1 ml toluene. The reaction procedure was as follows,

Compound **1a** (1.0 eq, 0.1 mmol) and propane-1-thiol (3.0 eq, 0.3 mmol, 20 µL), was added into a small vial at room temperature and air. The BIMP catalyst was dissolved in 1 mL toluene and slowly added to the reaction mixture. After full conversion, as indicated by TLC analysis, the crude mixture was directly purified by flash column chromatography using pentane/EtOAc = 7/1. Giving a yellow oil in (36 mg, 0.099 mmol, 99% yield) and 94% ee with d.r.> 20/1 (for gram scale : 99% yield and 94% ee with d.r.> 20/1)

^{1}H NMR (400 MHz, C_6D_6) δ 7.72 – 7.65 (m, 2H), 7.12 (m, 2H), 7.00 (m, 1H), 3.51 (s, 3H), 3.45 (s, 1H), 3.26 (s, 3H), 3.19 (s, 3H), 2.38 (m, 2H), 1.34 – 1.17 (m, 2H), 0.63 (t, *J* = 7.3 Hz, 3H).

^{13}C NMR (151 MHz, $CDCl_3$) δ 166.7, 165.7, 164.7, 128.9, 128.5, 127.9, 127.6, 52.4, 52.3, 51.6, 22.6, 22.4, 22.3, 22.0, 13.6, 13.5

HRMS (ESI) m/z calcd. for $C_{18}H_{22}O_6S$ ([M+H] $^+$) 367.1210, found 367.1219

$[\alpha]_D^{25}$ = -15.1 (c = 1.0, CH_2Cl_2).

[HPLC traces]

[back to Table of Contents]

trimethyl (2S,3R)-3-(cyclohexylthio)-3-phenylcyclopropane-1,1,2-tricarboxylate (3b)

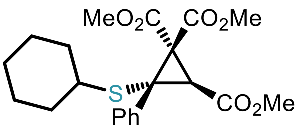

The BIMP catalysed reaction was carried with 0.1 mmol scale (1.0 eq) under room temperature and for 24 h with 10 mol% catalyst in 1 ml toluene. The reaction procedure was as follows,

Compound **1a** (1.0 eq, 0.1 mmol) and cyclohexanethiol (2.0 eq, 0.2 mmol), was added into a small vial at room temperature and air. The BIMP catalyst was dissolved in 1 mL toluene and slowly added to the reaction mixture. After full conversion, as indicated by TLC analysis, the crude mixture was directly purified by flash column chromatography using pentane/EtOAc = 7/1. Giving a colourless oil in (38.6 mg, 0.095 mmol, 95% yield) and 94% ee with d.r.> 20/1

^{1}H NMR (400 MHz, C_6D_6) δ 7.81 – 7.74 (m, 2H), 7.13 (d, *J* = 8.0 Hz, 2H), 7.04 – 6.95 (m, 1H), 3.55 (s, 1H), 3.53 (s, 3H), 3.27 (s, 3H), 3.19 (s, 3H), 2.69 (tt, *J* = 10.4, 3.7 Hz, 1H), 1.90 – 1.70 (m, 2H), 1.51 – 1.37 (m, 2H), 1.25 – 1.15 (m, 3H), 1.02 – 0.88 (m, 3H).

^{13}C NMR (101 MHz, C_6D_6) δ 166.6, 165.5, 164.5, 135.5, 130.8, 65.5, 52.5, 51.9, 51.5, 46.8, 44.6, 43.9, 38.6, 33.3, 33.2, 25.7, 25.4, 15.2.

HRMS (ESI) m/z calcd. for $C_{21}H_{26}O_6S$ ([M+H] $^+$) 407.1523, found 407.1513

$[\alpha]_D^{25}$ = -26.3 (c = 1.0, CH_2Cl_2).

[HPLC traces]

[back to Table of Contents]

trimethyl (2S,3R)-3-(isopropylthio)-3-phenylcyclopropane-1,1,2-tricarboxylate (3c)

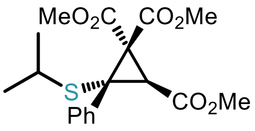

The BIMP catalysed reaction was carried with 0.1 mmol scale (1.0 eq) under room temperature and for 24 h with 10 mol% catalyst in 1 ml toluene. The reaction procedure was as follows,

Compound **1a** (1.0 eq, 0.1 mmol) and propane-2-thiol (2.0 eq, 0.2 mmol), was added into a small vial at room temperature and air. The BIMP catalyst was dissolved in 1 mL toluene and slowly added to the reaction mixture. After full conversion, as indicated by TLC analysis, the crude mixture was directly purified by flash column chromatography using pentane/EtOAc = 7/1. Giving a colourless oil in (34.8 mg, 0.095 mmol, 95% yield) and 93% ee with d.r. 10/1

^{1}H NMR (400 MHz, C_6D_6) δ 7.78 – 7.70 (m, 2H), 7.13 (s, 1H), 7.04 – 6.93 (m, 2H), 3.53 (s, 1H), 3.51 (s, 3H), 3.27 (s, 3H), 3.20 (s, 3H), 2.75 (hept, J = 6.8 Hz, 1H), 1.05 (d, J = 6.6 Hz, 3H), 0.93 (d, J = 6.9 Hz, 3H).

^{13}C NMR (101 MHz, C_6D_6) δ 166.5, 165.5, 164.4, 135.3, 130.8, 127.3, 52.5, 51.9, 51.5, 44.7, 38.7, 35.5, 22.9, 22.7.

HRMS (ESI) m/z calcd. for $C_{18}H_{22}O_6S$ ([M+Na]$^+$) 389.1029, found 389.1026

$[\alpha]_D^{25}$ = -12.6 (c = 1.0, CH_2Cl_2).

. [HPLC traces]

[back to Table of Contents]

trimethyl (2S,3R)-3-(dodecylthio)-3-phenylcyclopropane-1,1,2-tricarboxylate (3d)

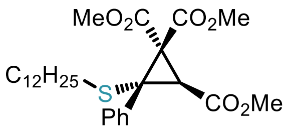

The BIMP catalysed reaction was carried with 0.1 mmol scale (1.0 eq) under room temperature and for 24 h with 10 mol% catalyst in 1 ml toluene. The reaction procedure was as follows,

Compound **1a** (1.0 eq, 0.1 mmol) and 1-dodecylthiol (2.0 eq, 0.2 mmol), was added into a small vial at room temperature and air. The BIMP catalyst was dissolved in 1 mL toluene and slowly added to the reaction mixture. After full conversion, as indicated by TLC analysis, the crude mixture was directly purified by flash column chromatography using pentane/EtOAc = 7/1. Giving a colourless oil in (46.2 mg, 0.094 mmol 94% yield) and 93% ee with d.r. 6/1

^{1}H NMR (400 MHz, C_6D_6) δ 7.76 – 7.62 (m, 2H), 7.13 (s, 1H), 7.05 – 6.94 (m, 2H), 3.54 (s, 3H), 3.48 (s, 1H), 3.27 (s, 3H), 3.20 (s, 3H), 2.61 – 2.37 (m, 2H), 1.30 – 1.08 (m, 20H), 0.91 (t, *J* = 6.6 Hz, 3H).

^{13}C NMR (101 MHz, C_6D_6) δ 166.4, 164.4, 134.9, 130.9, 129.1, 127.9, 127.6, 127.4, 127.3, 52.4, 51.9, 51.5, 44.9, 38.0, 31.9, 31.5, 29.7, 29.6, 29.4, 29.1, 28.8, 28.4, 22.7, 14.0.

HRMS (ESI) m/z calcd. for $C_{27}H_{40}O_6S$ ([M+H] $^{+}$) 493.2618, found 493.2610

$[\alpha]_D^{25}$ = -21.88 (c = 1.0, CH_2Cl_2).

[HPLC traces]

[back to Table of Contents]

trimethyl (2S,3R)-3-phenyl-3-((2-(trimethylsilyl)ethyl)thio)cyclopropane-1,1,2-tricarboxylate (3e)

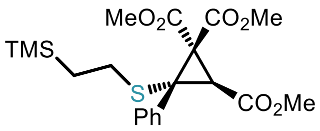

The BIMP catalysed reaction was carried with 0.1 mmol scale (1.0 eq) under room temperature and for 24 h with 10 mol% catalyst in 1 ml toluene. The reaction procedure was as follows,

Compound **1a** (1.0 eq, 0.1 mmol) and 2-(trimethylsilyl)ethane-1-thiol (2.0 eq, 0.2 mmol), was added into a small vial at room temperature and air. The BIMP catalyst was dissolved in 1 mL toluene and slowly added to the reaction mixture. After full conversion, as indicated by TLC analysis, the crude mixture was directly purified by flash column chromatography using pentane/EtOAc = 7/1. Giving a colourless oil in (40.0 mg, 0.094 mmol, 94%) and 93% ee with d.r. 10/1

^{1}H NMR (400 MHz, C_6D_6) δ 7.90 (dt, *J* = 8.1, 1.6 Hz, 2H), 7.32 – 7.27 (m, 2H), 7.17 (dd, *J* = 7.1, 1.6 Hz, 1H), 3.71 (s, 3H), 3.69 (s, 1H), 3.44 (s, 3H), 3.36 (s, 3H), 2.88 – 2.61 (m, 2H), 0.87 – 0.77 (m, 2H), 0.03 – -0.05 (m, 9H).

^{13}C NMR (101 MHz, C_6D_6) δ 130.9, 127.9, 127.6, 127.4, 127.3, 52.4, 51.9, 51.4, 37.9, 27.2, 15.8, -2.2.

HRMS (ESI) m/z calcd. for $C_{20}H_{28}O_6SSi$ ($[M+H]^+$) 425.1449, found 425.1449

$[\alpha]_D^{25}$ = -20.8 (c = 1.0, CH_2Cl_2).

. [HPLC traces]

[back to Table of Contents]

trimethyl (2S,3R)-3-((3-butoxy-3-oxopropyl)thio)-3-phenylcyclopropane-1,1,2-tricarboxylate (3f)

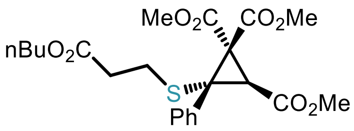

The BIMP catalysed reaction was carried with 0.1 mmol scale (1.0 eq) under room temperature and for 24 h with 10 mol% catalyst in 1 ml toluene. The reaction procedure was as follows,

Compound **1a** (1.0 eq, 0.1 mmol) and butyl 3-mercaptopropanoate (2.0 eq, 0.2 mmol), was added into a small vial at room temperature and air. The BIMP catalyst was dissolved in 1 mL toluene and slowly added to the reaction mixture. After full conversion, as indicated by TLC analysis, the crude mixture was directly purified by flash column chromatography using pentane/EtOAc = 7/1. Giving a colourless oil (44.8 mg, 0.099 mmol 99%) and 93% ee with d.r. 20/1

^{1}H NMR (400 MHz, $CDCl_3$) δ 7.45 – 7.38 (m, 2H), 7.33 – 7.26 (m, 3H), 4.03 (t, *J* = 6.7 Hz, 2H), 3.87 (s, 3H), 3.67 (s, 3H), 3.61 (s, 3H), 3.16 (s, 1H), 2.81 – 2.63 (m, 2H), 2.30 (t, *J* = 7.5 Hz, 2H), 1.56 (m, 2H), 1.40 – 1.28 (m, 2H), 0.92 (t, *J* = 7.3 Hz, 3H).

^{13}C NMR (101 MHz, $CDCl_3$) δ 171.4, 166.5, 165.6, 164.5, 133.7, 130.4, 128.9, 128.4, 128.0, 127.6, 64.6, 53.3, 52.7, 52.4, 47.1, 44.7, 37.6, 33.6, 30.5, 26.5, 19.0, 14.2, 13.6.

HRMS (ESI) m/z calcd. for $C_{22}H_{28}O_8S$ ([M+H] $^+$) 453.1578, found 453.1586

$[\alpha]_D^{25}$ = -16.1 (c = 1.0, CH_2Cl_2).

. [HPLC traces]

[back to Table of Contents]

trimethyl (2S,3R)-3-(phenethylthio)-3-phenylcyclopropane-1,1,2-tricarboxylate (3g)

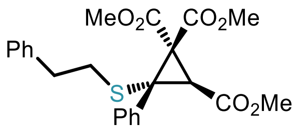

The BIMP catalysed reaction was carried with 0.1 mmol scale (1.0 eq) under room temperature and for 24 h with 10 mol% catalyst in 1 ml toluene. The reaction procedure was as follows,

Compound **1a** (1.0 eq, 0.1 mmol) and butyl 2-phenylethane-1-thiol (2.0 eq, 0.2 mmol), was added into a small vial at room temperature and air. The BIMP catalyst was dissolved in 1 mL toluene and slowly added to the reaction mixture. After full conversion, as indicated by TLC analysis, the crude mixture was directly purified by flash column chromatography using pentane/EtOAc = 7/1. Giving a colourless solid (42.4 mg, 0.099 mmol 99%) and 95% ee with d.r.> 20/1

^{1}H NMR (400 MHz, $CDCl_3$) δ 7.49 – 7.41 (m, 2H), 7.37 – 7.26 (m, 3H), 7.26 – 7.14 (m, 3H), 7.08 – 7.00 (m, 2H), 3.81 (s, 3H), 3.67 (s, 3H), 3.61 (s, 3H), 3.17 (s, 1H), 2.81 – 2.56 (m, 4H).

^{13}C NMR (101 MHz, C_6D_6) δ 203.4, 166.2, 164.3, 140.0, 134.7, 130.9, 128.3, 127.9, 127.6, 127.5, 127.5, 127.4, 126.2, 52.4, 51.9, 51.5, 47.8, 44.9, 37.8, 35.0, 32.9, 29.7.

HRMS (ESI) m/z calcd. for $C_{23}H_{24}O_6S$ ([M+H] $^+$) 429.1366, found 429.1376

$[\alpha]_D^{25}$ = -17.1 (c = 1.0, CH_2Cl_2).

m. p.: 60 – 62 °C.

. [HPLC traces]

[back to Table of Contents]

trimethyl (2S,3R)-3-((4-methoxybenzyl)thio)-3-phenylcyclopropane-1,1,2-tricarboxylate (3h)

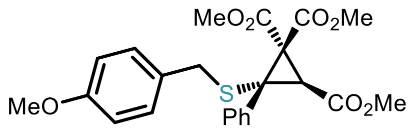

The BIMP catalysed reaction was carried with 0.1 mmol scale (1.0 eq) under room temperature and for 24 h with 10 mol% catalyst in 1 ml toluene. The reaction procedure was as follows,

Compound **1a** (1.0 eq, 0.1 mmol) and (4-methoxyphenyl)methanethiol (2.0 eq, 0.2 mmol), was added into a small vial at room temperature and air. The BIMP catalyst was dissolved in 1 mL toluene and slowly added to the reaction mixture. After full conversion, as indicated by TLC analysis, the crude mixture was directly purified by flash column chromatography using pentane/EtOAc = 7/1. Giving a colourless oil (44 mg, 0.099 mmol, 99%) and 92% ee with d.r.> 20/1

^{1}H NMR (400 MHz, C_6D_6) δ 7.76 – 7.69 (m, 2H), 7.19 (s, 1H), 7.10 – 7.00 (m, 2H), 6.98 – 6.90 (m, 2H), 6.60 (dq, *J* = 9.4, 2.4 Hz, 2H), 3.87 (dd, *J* = 11.5, 2.1 Hz, 1H), 3.59 (dd, *J* = 11.6, 2.1 Hz, 1H), 3.52 – 3.48 (s, 3H), 3.43 – 3.40 (s, 1H), 3.28 – 3.24 (s, 3H), 3.22 – 3.20 (s, 3H), 3.20 – 3.17 (s, 3H).

^{13}C NMR (101 MHz, C_6D_6) δ 178.4, 143.7, 130.9, 130.4, 127.9, 127.7, 127.4, 113.9, 54.3, 52.4, 51.9, 51.4, 37.9, 35.8.
HRMS (ESI) m/z calcd. for $C_{23}H_{24}O_7S$ ([M+H] $^+$) 445.1316, found 445.1308

$[\alpha]_D^{25}$ = -32.7 (c = 1.0, CH_2Cl_2).

. [HPLC traces]

[back to Table of Contents]

trimethyl (2S,3R)-3-(benzylthio)-3-phenylcyclopropane-1,1,2-tricarboxylate (3i)

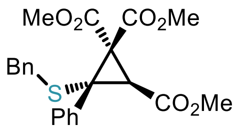

The BIMP catalysed reaction was carried with 0.1 mmol scale (1.0 eq) under room temperature and for 24 h with 10 mol% catalyst in 1 ml toluene. The reaction procedure was as follows,

Compound **1a** (1.0 eq, 0.1 mmol) and benzylthiol (2.0 eq, 0.2 mmol), was added into a small vial at room temperature and air. The BIMP catalyst was dissolved in 1 mL toluene and slowly added to the reaction mixture. After full conversion, as indicated by TLC analysis, the crude mixture was directly purified by flash column chromatography using pentane/EtOAc = 7/1. Giving a colourless oil (41 mg, 0.099 mmol, 99%) and 93% ee with d.r.> 20/1

^{1}H NMR (400 MHz, C_6D_6) δ 7.76 – 7.61 (m, 2H), 7.13 (s, 2H), 7.06 – 6.90 (m, 6H), 3.83 (d, *J* = 11.6 Hz, 1H), 3.56 (d, *J* = 11.6 Hz, 1H), 3.47 (s, 3H), 3.38 (s, 1H), 3.25 (s, 3H), 3.18 (s, 3H).

^{13}C NMR (101 MHz, C_6D_6) δ 166.4, 165.7, 159.7, 134.9, 131.0, 129.5, 127.3, 127.1, 126.9, 119.2, 116.0, 54.4, 52.6, 51.9, 51.5, 47.7, 37.5.

HRMS (ESI) m/z calcd. for $C_{23}H_{24}O_7S$ ([M+H] $^+$) 445.1316, found 445.1326

$[\alpha]_D^{25}$ = -16.9 (c = 1.0, CH_2Cl_2).

. [HPLC traces]

[back to Table of Contents]

trimethyl (2S,3R)-3-((3,5-difluorobenzyl)thio)-3-phenylcyclopropane-1,1,2-tricarboxylate (3j)

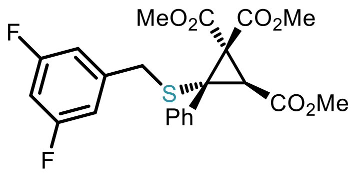

The BIMP catalysed reaction was carried with 0.1 mmol scale (1.0 eq) under room temperature and for 24 h with 10 mol% catalyst in 1 ml toluene. The reaction procedure was as follows,

Compound **1a** (1.0 eq, 0.1 mmol) and (3,5-difluorophenyl)methanethiol (2.0 eq, 0.2 mmol), was added into a small vial at room temperature and air. The BIMP catalyst was dissolved in 1 mL toluene and slowly added to the reaction mixture. After full conversion, as indicated by TLC analysis, the crude mixture was directly purified by flash column chromatography using pentane/EtOAc = 7/1. Giving a colourless oil (41 mg, 0.091 mmol, 91%) and 86% ee with d.r.> 20/1

^{1}H NMR (400 MHz, C_6D_6) δ 7.76 (s, 1H), 7.48 – 7.43 (m, 2H), 7.37 (d, *J* = 1.4 Hz, 2H), 6.95 – 6.83 (m, 3H), 3.76 (s, 1H), 3.60 (s, 3H), 3.31 – 3.20 (m, 5H), 3.10 (s, 3H).

^{13}C NMR (101 MHz, C_6D_6) δ 166.4, 165.7, 164.1, 135.2, 134.8, 133.5, 133.2, 131.5, 131.0, 128.3, 127.9, 127.6, 127.5, 127.4, 127.3, 126.9, 126.7, 126.2, 65.5, 5.70, 51.9, 51.5, 48.1, 37.6, 15.2.

^{19}F NMR (377 MHz, C_6D_6) δ -109.87.

HRMS (ESI) m/z calcd. for $C_{22}H_{20}F_2O_6S$ ([M+H] $^+$) 451.1021, found 451.1031

$[\alpha]_D^{25}$ = -31.7 (c = 1.0, CH_2Cl_2).

. [HPLC traces]

[back to Table of Contents]

trimethyl (2S,3R)-3-((3-fluorobenzyl)thio)-3-phenylcyclopropane-1,1,2-tricarboxylate (3k)

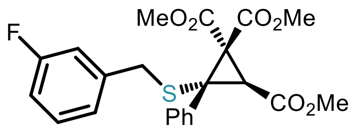

The BIMP catalysed reaction was carried with 0.1 mmol scale (1.0 eq) under room temperature and for 24 h with 10 mol% catalyst in 1 ml toluene. The reaction procedure was as follows,

Compound **1a** (1.0 eq, 0.1 mmol) and (3-fluorophenyl)methanethiol (2.0 eq, 0.2 mmol), was added into a small vial at room temperature and air. The BIMP catalyst was dissolved in 1 mL toluene and slowly added to the reaction mixture. After full conversion, as indicated by TLC analysis, the crude mixture was directly purified by flash column chromatography using pentane/EtOAc = 7/1. Giving a colourless oil (39.4 mg, 0.091 mmol, 91%) and 90% ee with d.r.> 20/1

^{1}H NMR (400 MHz, C_6D_6) δ 7.75 – 7.66 (m, 2H), 7.12 (d, *J* = 1.6 Hz, 3H), 7.06 – 6.98 (m, 1H), 6.86 (td, *J* = 7.6, 1.8 Hz, 1H), 6.74 – 6.54 (m, 3H), 3.86 (d, *J* = 12.0 Hz, 1H), 3.65 (d, *J* = 12.0 Hz, 1H), 3.51 (s, 3H), 3.39 (s, 1H), 3.24 (s, 3H), 3.17 (s, 3H).

^{13}C NMR (101 MHz, C_6D_6) δ 166.1, 165.4, 134.5, 131.2, 131.0, 128.9, 128.8, 127.9, 127.6, 127.4, 123.9, 52.5, 51.9, 51.4, 37.7, 28.9.

^{19}F NMR (377 MHz, C_6D_6) δ -117.48.

HRMS (ESI) m/z calcd. for $C_{22}H_{21}FO_6S$ ([M+H] $^+$) 433.1116, found 433.1117

$[\alpha]_D^{25}$ = -36.9 (c = 1.0, CH_2Cl_2).

. [HPLC traces]

[back to Table of Contents]

trimethyl (2S,3R)-3-((3-methoxyphenyl)thio)-3-phenylcyclopropane-1,1,2-tricarboxylate (3l)

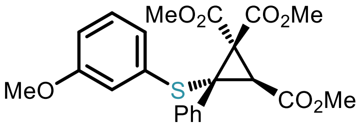

The BIMP catalysed reaction was carried with 0.1 mmol scale (1.0 eq) under room temperature and for 24 h with 10 mol% catalyst in 1 ml toluene. The reaction procedure was as follows,

Compound **1a** (1.0 eq, 0.1 mmol) and 3-methoxybenzenethiol (2.0 eq, 0.2 mmol), was added into a small vial at room temperature and air. The BIMP catalyst was dissolved in 1 mL toluene and slowly added to the reaction mixture. After full conversion, as indicated by TLC analysis, the crude mixture was directly purified by flash column chromatography using pentane/EtOAc = 7/1. Giving a colourless oil (38.7 mg, 0.09 mmol, 90%) and 85% ee with d.r.> 20/1

^{1}H NMR (400 MHz, C_6D_6) δ 7.54 – 7.46 (m, 2H), 7.02 – 6.79 (m, 6H), 6.67 (dd, *J* = 8.4, 2.7 Hz, 1H), 3.69 (s, 1H), 3.55 (s, 3H), 3.26 (s, 3H), 3.19 (s, 3H), 3.12 (s, 3H).

^{13}C NMR (101 MHz, C_6D_6) δ 166.4, 165.7, 159.7, 134.9, 131.0, 129.5, 127.3, 127.1, 126.9, 119.2, 116.0, 54.4, 52.6, 51.9, 51.5, 47.7, 37.5.

HRMS (ESI) m/z calcd. for $C_{22}H_{22}O_7S$ ([M+H] $^+$) 430.1316, found 430.1326

$[\alpha]_D^{25}$ = -33.6 (c = 1.0, CH_2Cl_2).

. [HPLC traces]

[back to Table of Contents]

trimethyl (2S,3R)-3-(naphthalen-2-ylthio)-3-phenylcyclopropane-1,1,2-tricarboxylate (3m)

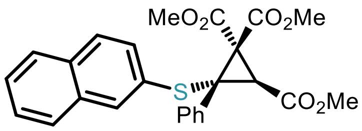

The BIMP catalysed reaction was carried with 0.1 mmol scale (1.0 eq) under room temperature and for 24 h with 10 mol% catalyst in 1 ml toluene. The reaction procedure was as follows,

Compound **1a** (1.0 eq, 0.1 mmol) and naphthalene-2-thiol (2.0 eq, 0.2 mmol), was added into a small vial at room temperature and air. The BIMP catalyst was dissolved in 1 mL toluene and slowly added to the reaction mixture. After full conversion, as indicated by TLC analysis, the crude mixture was directly purified by flash column chromatography using pentane/EtOAc = 7/1. Giving a colourless oil (41.3 mg, 0.092 mmol, 92%) and 80% ee with d.r.> 20/1

^{1}H NMR (400 MHz, C_6D_6) δ 7.76 (s, *J* = 1.2 Hz, 1H), 7.50 – 7.35 (m, 6H), 7.14 – 7.09 (m, 2H), 6.94 – 6.82 (m, 3H), 3.76 (s, 1H), 3.60 (s, 3H), 3.26 (s, 3H), 3.10 (s, 3H).

^{13}C NMR (101 MHz, C_6D_6) δ 166.4, 165.7, 135.2, 134.8, 133.5, 133.2, 131.5, 131.0, 128.6, 128.3, 127.9, 127.6, 127.4, 127.3, 126.9, 126.7, 126.2, 52.7, 51.9, 51.5, 48.1, 37.6.

HRMS (ESI) m/z calcd. for $C_{25}H_{22}O_6S$ ([M+H] $^+$) 451.1210, found 451.1197

$[\alpha]_D^{25}$ = -41.8 (c = 1.0, CH_2Cl_2).

. [HPLC traces]

[back to Table of Contents]

trimethyl (2S,3R)-3-phenyl-3-(phenylthio)cyclopropane-1,1,2-tricarboxylate (3n)

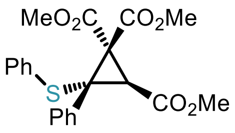

The BIMP catalysed reaction was carried with 0.1 mmol scale (1.0 eq) under room temperature and for 24 h with 10 mol% catalyst in 1 ml toluene. The reaction procedure was as follows,

Compound **1a** (1.0 eq, 0.1 mmol) and benzenethiol (2.0 eq, 0.2 mmol), was added into a small vial at room temperature and air. The BIMP catalyst was dissolved in 1 mL toluene and slowly added to the reaction mixture. After full conversion, as indicated by TLC analysis, the crude mixture was directly purified by flash column chromatography using pentane/EtOAc = 7/1. Giving a colourless oil (39.5 mg, 0.099 mmol, 99%) and 87% ee with d.r.> 20/1

^{1}H NMR (400 MHz, C_6D_6) δ 7.48 – 7.40 (m, 2H), 7.26 (dt, *J* = 6.5, 1.6 Hz, 2H), 6.98 (dd, *J* = 8.2, 6.7 Hz, 2H), 6.94 – 6.83 (m, 4H), 3.67 (s, 1H), 3.55 (s, 3H), 3.26 (s, 3H), 3.12 (s, 3H).

^{13}C NMR (101 MHz, C_6D_6) δ 166.4, 165.7, 135.3, 134.8, 131.2, 131.0, 128.9, 128.6, 127.9, 127.6, 127.5, 127.4, 127.3, 126.9, 52.6, 51.9, 51.5, 37.4.

HRMS (ESI) m/z calcd. for $C_{21}H_{20}O_6S$ ([M+H] $^+$) 401.1053, found 401.1058

$[\alpha]_D^{25}$ = -28.1 (c = 1.0, CH_2Cl_2).

. [HPLC traces]

[back to Table of Contents]

trimethyl (2S,3R)-3-(propylthio)-3-(p-tolyl)cyclopropane-1,1,2-tricarboxylate (3o)

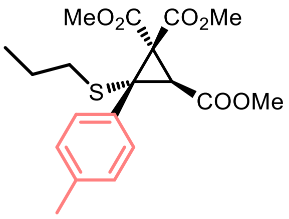

The BIMP catalysed reaction was carried with 0.1 mmol scale (1.0 eq) under room temperature and for 24 h with 10 mol% catalyst in 1 ml toluene. The reaction procedure was as follows,

Compound **1b** (1.0 eq, 0.1 mmol) and propane-1-thiol (2.0 eq, 0.2 mmol), was added into a small vial at room temperature and air. The BIMP catalyst was dissolved in 1 mL toluene and slowly added to the reaction mixture. After full conversion, as indicated by TLC analysis, the crude mixture was directly purified by flash column chromatography using pentane/EtOAc = 7/1. Giving a colourless oil (35.3 mg, 0.093 mmol, 93%) and 94% ee with d.r. 4/1

^{1}H NMR (400 MHz, C_6D_6) δ 7.69 (d, *J* = 7.8 Hz, 2H), 7.04 (d, *J* = 7.8 Hz, 2H), 3.71 (s, 1H), 3.59 (s, 3H), 3.37 (s, 3H), 3.30 (s, 3H), 2.50 (m, 2H), 2.10 (s, 3H), 1.38 (q, *J* = 7.4 Hz, 2H), 0.73 (t, *J* = 7.4 Hz, 3H).

^{13}C NMR (101 MHz, C_6D_6) δ 220.0, 166.6, 166.4, 165.6, 164.5, 137.1, 131.8, 130.9, 129.2, 128.8, 128.1, 128.0, 127.7, 127.5, 52.5, 52.3, 52.2, 51.9, 51.5, 47.9, 44.8, 38.0, 33.5, 33.5, 22.0, 21.8, 20.8, 13.3, 13.2.

HRMS (ESI) m/z calcd. for $C_{19}H_{24}O_6S$ ([M+H] $^+$) 381.1366, found 381.1367

$[\alpha]_D^{25}$ = -33.1 (c = 1.0, CH_2Cl_2).

. [HPLC traces]

[back to Table of Contents]

trimethyl (2S,3R)-3-(propylthio)-3-(o-tolyl)cyclopropane-1,1,2-tricarboxylate (3p)

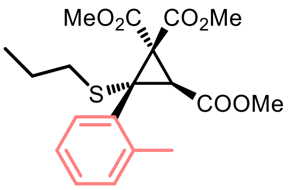

The BIMP catalysed reaction was carried with 0.1 mmol scale (1.0 eq) under room temperature and for 24 h with 10 mol% catalyst in 1 ml toluene. The reaction procedure was as follows,

Compound **1c** (1.0 eq, 0.1 mmol) and propane-1-thiol (2.0 eq, 0.2 mmol), was added into a small vial at room temperature and air. The BIMP catalyst was dissolved in 1 mL toluene and slowly added to the reaction mixture. After full conversion, as indicated by TLC analysis, the crude mixture was directly purified by flash column chromatography using pentane/EtOAc = 7/1. Giving a colourless oil in (34.8 mg, 0.092 mmol, 92%) and 99% ee with d.r. 4/1

^{1}H NMR (400 MHz, $CDCl_3$) δ 7.11 (tt, *J* = 16.5, 7.9 Hz, 3H), 6.95 (t, *J* = 7.5 Hz, 1H), 6.81 (d, *J* = 7.7 Hz, 1H), 3.82 (s, 3H), 3.66 (s, 3H), 3.52 (s, 3H), 3.12 (s, 1H), 2.53 – 2.39 (m, 5H), 1.40 (m, 2H), 0.80 (t, *J* = 7.3 Hz, 3H).

^{13}C NMR (101 MHz, $CDCl_3$) δ 167.1, 165.9, 164.2, 140.2, 132.7, 130.9, 128.0, 127.8, 124.3, 77.3, 77.0, 76.7, 53.3, 52.6, 52.6, 46.5, 44.3, 39.1, 34.0, 21.6, 20.1, 15.2, 13.5.

HRMS (ESI) m/z calcd. for $C_{19}H_{24}O_6S$ ([M+H] $^+$) 381.1366, found 381.1369

$[\alpha]_D^{25}$ = -27.7 (c = 1.0, CH_2Cl_2).

. [HPLC traces]

[back to Table of Contents]

trimethyl (2S,3R)-3-(propylthio)-3-(m-tolyl)cyclopropane-1,1,2-tricarboxylate (3q)

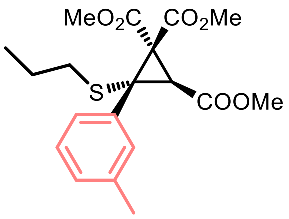

The BIMP catalysed reaction was carried with 0.1 mmol scale (1.0 eq) under room temperature and for 24 h with 10 mol% catalyst in 1 ml toluene. The reaction procedure was as follows,

Compound **1d** (1.0 eq, 0.1 mmol) and propane-1-thiol (2.0 eq, 0.2 mmol), was added into a small vial at room temperature and air. The BIMP catalyst was dissolved in 1 mL toluene and slowly added to the reaction mixture. After full conversion, as indicated by TLC analysis, the crude mixture was directly purified by flash column chromatography using pentane/EtOAc = 7/1. Giving a colourless oil (35.2 mg, 0.093 mmol, 93%) and 93% ee with d.r. 20/1

^{1}H NMR (400 MHz, C_6D_6) δ 7.62 (s, 1H), 7.47 (d, *J* = 7.8 Hz, 1H), 7.07 (t, *J* = 7.6 Hz, 1H), 6.86 (d, *J* = 7.6 Hz, 1H), 3.51 (s, 3H), 3.47 (s, 1H), 3.28 (s, 3H), 3.21 (s, 3H), 2.53 – 2.32 (m, 2H), 2.12 (s, 3H), 1.35 – 1.21 (m, 2H), 0.64 (t, *J* = 7.3, 3H).

^{13}C NMR (101 MHz, C_6D_6) δ 166.4, 165.5, 164.4, 136.7, 134.7, 131.8, 128.2, 127.9, 127.8, 127.6, 127.4, 127.1, 52.4, 51.9, 51.4, 47.8, 44.9, 37.9, 33.4, 21.7, 21.0, 13.1.

HRMS (ESI) m/z calcd. for $C_{19}H_{24}O_6S$ ([M+H] $^+$) 381.1366, found 381.1376

$[\alpha]_D^{25}$ = -25.5 (c = 1.0, CH_2Cl_2).

. [HPLC traces]

[back to Table of Contents]

trimethyl (2S,3R)-3-(4-fluorophenyl)-3-(propylthio)cyclopropane-1,1,2-tricarboxylate (3r)

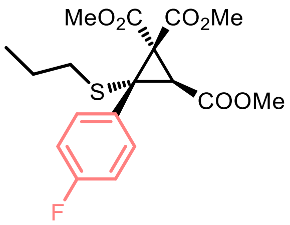

The BIMP catalysed reaction was carried with 0.1 mmol scale (1.0 eq) under room temperature and for 24 h with 10 mol% catalyst in 1 ml toluene. The reaction procedure was as follows,

Compound **1e** (1.0 eq, 0.1 mmol) and propane-1-thiol (2.0 eq, 0.2 mmol), was added into a small vial at room temperature and air. The BIMP catalyst was dissolved in 1 mL toluene and slowly added to the reaction mixture. After full conversion, as indicated by TLC analysis, the crude mixture was directly purified by flash column chromatography using pentane/EtOAc = 7/1. Giving a colourless oil (36.1 mg, 0.094 mmol, 94%) and 91% ee with d.r. 20/1

^{1}H NMR (400 MHz, C_6D_6) δ 7.56 – 7.46 (m, 2H), 6.82 – 6.72 (m, 2H), 3.49 (s, 3H), 3.40 (s, 1H), 3.26 (s, 3H), 3.17 (s, 3H), 2.30 (m, 2H), 1.25 (m, 2H), 0.64 (t, J = 7.3 Hz, 3H).

^{13}C NMR (101 MHz, C_6D_6) δ 166.3, 165.3, 164.3, 132.7, 132.6, 130.6, 130.6, 127.9, 127.6, 127.4, 114.3, 114.1, 52.4, 51.9, 51.5, 47.8, 44.0, 37.9, 33.3, 21.7, 13.1.

^{19}F NMR (377 MHz, C_6D_6) δ -114.37.

HRMS (ESI) m/z calcd. for $C_{18}H_{21}FO_6S$ ([M+H] $^+$) 385.1116, found 385.1099

$[\alpha]_D^{25}$ = -18.7 (c = 1.0, CH_2Cl_2).

. [HPLC traces]

[back to Table of Contents]

trimethyl (2S,3R)-3-(4-chlorophenyl)-3-(propylthio)cyclopropane-1,1,2-tricarboxylate (3s)

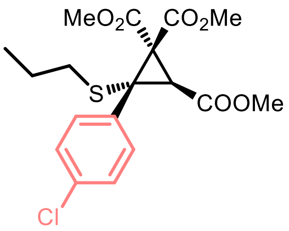

The BIMP catalysed reaction was carried with 0.1 mmol scale (1.0 eq) under room temperature and for 24 h with 10 mol% catalyst in 1 ml toluene. The reaction procedure was as follows,

Compound **1f** (1.0 eq, 0.1 mmol) and propane-1-thiol (2.0 eq, 0.2 mmol), was added into a small vial at room temperature and air. The BIMP catalyst was dissolved in 1 mL toluene and slowly added to the reaction mixture. After full conversion, as indicated by TLC analysis, the crude mixture was directly purified by flash column chromatography using pentane/EtOAc = 7/1. Giving a colourless oil (36.7 mg, 0.094 mmol, 92%) and 92% ee with d.r. > 20/1

^{1}H NMR (400 MHz, C_6D_6) δ 7.50 – 7.41 (m, 2H), 7.13 – 7.04 (m, 2H), 3.48 (s, 3H), 3.39 (s, 1H), 3.25 (s, 3H), 3.16 (s, 3H), 2.29 (m, 2H), 1.24 (m, 2H), 0.63 (t, *J* = 7.3 Hz, 3H).

^{13}C NMR (101 MHz, C_6D_6) δ 166.2, 165.2, 133.4, 133.4, 132.3, 127.9, 127.6, 127.6, 127.4, 52.4, 52.0, 51.5, 47.7, 44.0, 37.8, 33.3, 21.6, 13.1.

HRMS (ESI) m/z calcd. for $C_{18}H_{21}ClO_6S$ ([M+H] $^{+}$) 401.0820, found 401.0824

$[\alpha]_D^{25}$ = -23.1 (c = 1.0, CH_2Cl_2).

. [HPLC traces]

[back to Table of Contents]

trimethyl (2S,3R)-3-(propylthio)-3-(4-(trifluoromethyl)phenyl)cyclopropane-1,1,2-tricarboxylate (3t)

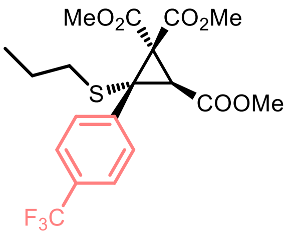

The BIMP catalysed reaction was carried with 0.1 mmol scale (1.0 eq) under room temperature and for 24 h with 10 mol% catalyst in 1 ml toluene. The reaction procedure was as follows,

Compound **1g** (1.0 eq, 0.1 mmol) and propane-1-thiol (2.0 eq, 0.2 mmol), was added into a small vial at room temperature and air. The BIMP catalyst was dissolved in 1 mL toluene and slowly added to the reaction mixture. After full conversion, as indicated by TLC analysis, the crude mixture was directly purified by flash column chromatography using pentane/EtOAc = 7/1. Giving a colourless oil (41 mg, 0.094 mmol, 94%) and 84% ee with d.r. 20/1

^{1}H NMR (400 MHz, C_6D_6) δ 7.60 (d, *J* = 8.1 Hz, 2H), 7.35 (d, *J* = 8.1 Hz, 2H), 3.48 (s, 3H), 3.40 (s, 1H), 3.23 (s, 3H), 3.16 (s, 3H), 2.26 (m, 2H), 1.22 (m, 2H), 0.61 (t, *J* = 7.3 Hz, 3H).

^{13}C NMR (101 MHz, C_6D_6) δ 166.1, 165.1, 164.2, 138.9, 131.2, 127.9, 127.6, 127.4, 124.3, 124.3, 124.2, 52.5, 52.0, 51.6, 37.8, 33.3, 21.5, 13.0.

^{19}F NMR (377 MHz, C_6D_6) δ -61.99.

HRMS (ESI) m/z calcd. for $C_{19}H_{21}F_3O_6S$ ($[M+H]^+$) 435.1084, found 435.1095

$[\alpha]_D^{25}$ = -19.1 (c = 1.0, CH_2Cl_2).

. [HPLC traces]

[back to Table of Contents]

trimethyl (2S,3R)-3-(4-(tert-butyl)phenyl)-3-(propylthio)cyclopropane-1,1,2-tricarboxylate (3u)

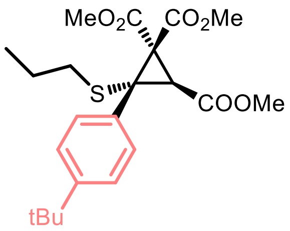

The BIMP catalysed reaction was carried with 0.1 mmol scale (1.0 eq) under room temperature and for 24 h with 10 mol% catalyst in 1 ml toluene. The reaction procedure was as follows,

Compound **1h** (1.0 eq, 0.1 mmol) and propane-1-thiol (2.0 eq, 0.2 mmol), was added into a small vial at room temperature and air. The BIMP catalyst was dissolved in 1 mL toluene and slowly added to the reaction mixture. After full conversion, as indicated by TLC analysis, the crude mixture was directly purified by flash column chromatography using pentane/EtOAc = 7/1. Giving a colourless oil (39.3 mg, 0.093 mmol, 93%) and 93% ee with d.r. 15/1

^{1}H NMR (400 MHz, C_6D_6) δ 7.81 – 7.62 (m, 2H), 7.32 – 7.25 (m, 2H), 3.51 (s, 3H), 3.49 (s, 1H), 3.28 (s, 3H), 3.23 (s, 3H), 2.55 – 2.32 (m, 2H), 1.32 – 1.26 (m, 2H), 1.13 (s, 9H), 0.63 (t, *J* = 7.3 Hz, 3H).

^{13}C NMR (101 MHz, C_6D_6) δ 166.5, 165.6, 164.3, 150.1, 131.8, 130.7, 127.9, 127.7, 127.4, 124.3, 52.4, 51.9, 51.5, 47.8, 44.7, 38.0, 34.1, 33.4, 30.9, 21.7, 13.1.

HRMS (ESI) m/z calcd. for $C_{22}H_{30}O_6S$ ([M+H] $^+$) 423.1836, found 423.1826

$[\alpha]_D^{25}$ = -24.4 (c = 1.0, CH_2Cl_2).

. [HPLC traces]

[back to Table of Contents]

1,1-dibenzyl 2-methyl (2S,3R)-3-phenyl-3-(propylthio)cyclopropane-1,1,2-tricarboxylate (3v)

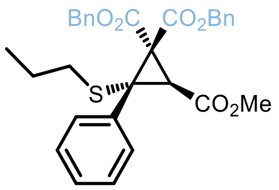

The BIMP catalysed reaction was carried with 0.1 mmol scale (1.0 eq) under room temperature and for 24 h with 10 mol% catalyst in 1 ml toluene. The reaction procedure was as follows,

Compound **1i** (1.0 eq, 0.1 mmol) and propane-1-thiol (2.0 eq, 0.2 mmol), was added into a small vial at room temperature and air. The BIMP catalyst was dissolved in 1 mL toluene and slowly added to the reaction mixture. After full conversion, as indicated by TLC analysis, the crude mixture was directly purified by flash column chromatography using pentane/EtOAc = 7/1. Giving a colourless oil (48.7 mg, 0.092 mmol, 92%) and 93% ee with d.r. 4/1

^{1}H NMR (400 MHz, C_6D_6) δ 7.69 – 7.62 (m, 2H), 7.29 – 7.23 (m, 2H), 7.07 – 6.92 (m, 11H), 5.26 – 5.12 (m, 2H), 5.01 – 4.77 (m, 2H), 3.50 (s, 1H), 3.08 (s, 3H), 2.43 – 2.24 (m, 2H), 1.20 (m, 2H), 0.59 (t, *J* = 7.4 Hz, 3H).

^{13}C NMR (101 MHz, C_6D_6) δ 166.2, 130.9, 129.2, 128.4, 128.3, 128.2, 128.1, 128.0, 127.9, 127.8, 127.6, 127.4, 127.3, 67.8, 67.1, 51.3, 38.2, 33.4, 21.6, 13.1.

HRMS (ESI) m/z calcd. for $C_{30}H_{30}O_6S$ ([M+H] $^+$) 519.1836, found 519.1848

$[\alpha]_D^{25}$ = -19.8 (c = 1.0, CH_2Cl_2).

. [HPLC traces]

[back to Table of Contents]

1,1-diethyl 2-methyl (2S,3R)-3-phenyl-3-(propylthio)cyclopropane-1,1,2-tricarboxylate (3w)

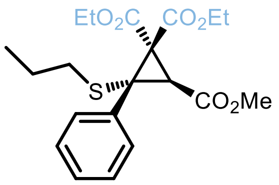

The BIMP catalysed reaction was carried with 0.1 mmol scale (1.0 eq) under room temperature and for 24 h with 10 mol% catalyst in 1 ml toluene. The reaction procedure was as follows,

Compound **1j** (1.0 eq, 0.1 mmol) and propane-1-thiol (2.0 eq, 0.2 mmol), was added into a small vial at room temperature and air. The BIMP catalyst was dissolved in 1 mL toluene and slowly added to the reaction mixture. After full conversion, as indicated by TLC analysis, the crude mixture was directly purified by flash column chromatography using pentane/EtOAc = 7/1. Giving a colourless oil (36.3 mg, 0.092 mmol, 92%) and 93% ee with d.r. > 20/1

^{1}H NMR (400 MHz, C_6D_6) δ 7.75 – 7.66 (m, 2H), 7.11 (d, *J* = 7.6 Hz, 2H), 6.99 (t, *J* = 7.4 Hz, 1H), 4.25 – 4.10 (m, 2H), 3.90 (m, 2H), 3.48 (s, 1H), 3.20 (s, 3H), 2.41 (m, 2H), 1.28 (m, 2H), 1.07 (t, *J* = 7.1 Hz, 3H), 0.77 (t, *J* = 7.1 Hz, 3H), 0.64 (t, *J* = 7.4 Hz, 3H).

^{13}C NMR (101 MHz, C_6D_6) δ 166.5, 165.1, 135.2, 131.0, 127.9, 127.7, 127.4, 127.3, 127.2, 61.9, 61.2, 51.3, 44.8, 38.1, 33.4, 21.7, 13.7, 13.3, 13.1.

HRMS (ESI) m/z calcd. for $C_{20}H_{26}O_6S$ ([M+H] $^{+}$) 395.1523, found 395.1531

$[\alpha]_D^{25}$ = -18.4 (c = 1.0, CH_2Cl_2).

. [HPLC traces]

[back to Table of Contents]

2-ethyl 1,1-dimethyl (2S,3R)-3-phenyl-3-(propylthio)cyclopropane-1,1,2-tricarboxylate (3x)

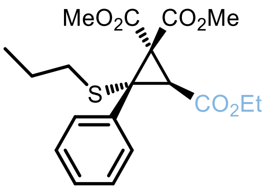

The BIMP catalysed reaction was carried with 0.1 mmol scale (1.0 eq) under room temperature and for 24 h with 10 mol% catalyst in 1 ml toluene. The reaction procedure was as follows,

Compound **1k** (1.0 eq, 0.1 mmol) and propane-1-thiol (2.0 eq, 0.2 mmol), was added into a small vial at room temperature and air. The BIMP catalyst was dissolved in 1 mL toluene and slowly added to the reaction mixture. After full conversion, as indicated by TLC analysis, the crude mixture was directly purified by flash column chromatography using pentane/EtOAc = 7/1. Giving a colourless oil (37.6 mg, 0.099 mmol, 99%) and 92% ee with d.r. > 20/1

^{1}H NMR (400 MHz, $CDCl_3$) δ 7.38 – 7.32 (m, 2H), 7.26 – 7.17 (m, 3H), 4.11 – 3.95 (m, 2H), 3.80 (s, 3H), 3.53 (s, 3H), 3.07 (s, 1H), 2.34 (m, 2H), 1.35 (m, 2H), 1.12 (t, J = 7.1 Hz, 3H), 0.78 (t, J = 7.4 Hz, 3H).

^{13}C NMR (101 MHz, $CDCl_3$) δ 166.3, 165.8, 164.7, 134.1, 130.5, 127.7, 127.3, 77.3, 77.0, 76.7, 61.5, 53.2, 52.6, 47.3, 44.6, 37.7, 33.4, 21.8, 13.9, 13.5.

HRMS (ESI) m/z calcd. for $C_{19}H_{24}O_6S$ ([M+H] $^+$) 381.1366, found 381.1374

$[\alpha]_D^{25}$ = -14.7 (c = 1.0, CH_2Cl_2).

. [HPLC traces]

[back to Table of Contents]

2-hexyl 1,1-dimethyl (2S,3R)-3-phenyl-3-(propylthio)cyclopropane-1,1,2-tricarboxylate (3y)

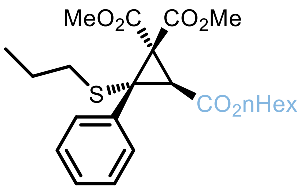

The BIMP catalysed reaction was carried with 0.1 mmol scale (1.0 eq) under room temperature and for 24 h with 10 mol% catalyst in 1 ml toluene. The reaction procedure was as follows,

Compound **1m** (1.0 eq, 0.1 mmol) and propane-1-thiol (2.0 eq, 0.2 mmol), was added into a small vial at room temperature and air. The BIMP catalyst was dissolved in 1 mL toluene and slowly added to the reaction mixture. After full conversion, as indicated by TLC analysis, the crude mixture was directly purified by flash column chromatography using pentane/EtOAc = 7/1. Giving a colourless oil (28.3 mg, 0.092 mmol, 92%) and 94% ee with d.r. > 20/1

^{1}H NMR (400 MHz, C_6D_6) δ 7.79 – 7.71 (m, 2H), 7.13 (s, 2H), 7.06 – 6.94 (m, 1H), 3.88 (m, 2H), 3.54 (s, 1H), 3.51 (s, 3H), 3.31 (s, 3H), 2.40 (m, 2H), 1.32 – 1.01 (m, 10H), 0.82 (t, *J* = 7.2 Hz, 3H), 0.63 (t, *J* = 7.4 Hz, 3H).

^{13}C NMR (101 MHz, C_6D_6) δ 131.0, 127.9, 127.6, 127.5, 127.4, 127.2, 65.3, 52.4, 51.8, 38.1, 33.4, 31.3, 28.2, 25.3, 22.4, 21.7, 13.8, 13.1.

HRMS (ESI) m/z calcd. for $C_{23}H_{32}O_6S$ ([M+H]$^+$) 437.1992, found 437.2003

$[\alpha]_D^{25}$ = -8.8 (c = 1.0, CH_2Cl_2).

. [HPLC traces]

[back to Table of Contents]

2-isobutyl 1,1-dimethyl (2S,3R)-3-phenyl-3-(propylthio)cyclopropane-1,1,2-tricarboxylate (3z)

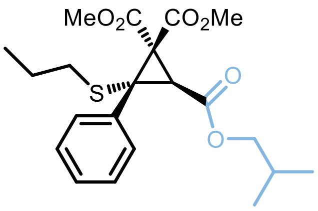

The BIMP catalysed reaction was carried with 0.1 mmol scale (1.0 eq) under room temperature and for 24 h with 10 mol% catalyst in 1 ml toluene. The reaction procedure was as follows,

Compound **1l** (1.0 eq, 0.1 mmol) and propane-1-thiol (2.0 eq, 0.2 mmol), was added into a small vial at room temperature and air. The BIMP catalyst was dissolved in 1 mL toluene and slowly added to the reaction mixture. After full conversion, as indicated by TLC analysis, the crude mixture was directly purified by flash column chromatography using pentane/EtOAc = 7/1. Giving a colourless oil (40.4 mg, 0.099 mmol, 99%) and 93% ee with d.r. > 20/1

^{1}H NMR (400 MHz, $CDCl_3$) δ 7.37 – 7.32 (m, 2H), 7.26 – 7.17 (m, 3H), 3.80 (s, 3H), 3.79 – 3.67 (m, 2H), 3.53 (s, 3H), 3.10 (s, 1H), 2.45 – 2.24 (m, 2H), 1.79 (m, 1H), 1.36 (m, 2H), 0.85 – 0.73 (m, 9H).

^{13}C NMR (101 MHz, $CDCl_3$) δ 166.4, 165.8, 164.6, 134.1, 130.5, 127.6, 127.4, 77.3, 77.0, 76.7, 71.7, 53.2, 52.6, 47.2, 44.5, 37.8, 33.4, 27.4, 21.8, 19.0, 19.0, 13.5.

HRMS (ESI) m/z calcd. for $C_{21}H_{28}O_6S$ ([M+H] $^+$) 409.1679, found 409.1694

$[\alpha]_D^{25}$ = -13.7 (c = 1.0, CH_2Cl_2).

. [HPLC traces]

[back to Table of Contents]

2-(2-methoxyethyl) 1,1-dimethyl (2S,3R)-3-phenyl-3-(propylthio)cyclopropane-1,1,2-tricarboxylate (3aa)

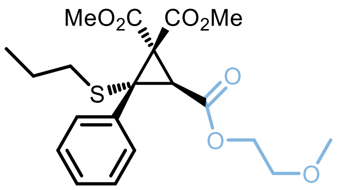

The BIMP catalysed reaction was carried with 0.1 mmol scale (1.0 eq) under room temperature and for 24 h with 10 mol% catalyst in 1 ml toluene. The reaction procedure was as follows,

Compound **1n** (1.0 eq, 0.1 mmol) and propane-1-thiol (2.0 eq, 0.2 mmol), was added into a small vial at room temperature and air. The BIMP catalyst was dissolved in 1 mL toluene and slowly added to the reaction mixture. After full conversion, as indicated by TLC analysis, the crude mixture was directly purified by flash column chromatography using pentane/EtOAc = 7/1. Giving a colourless oil (40mg, 0.095 mmol, 95%) and 94% ee with d.r. > 20/1

^{1}H NMR (400 MHz, C_6D_6) δ 7.81 – 7.72 (m, 2H), 7.15 (m, 2H), 7.05 – 6.97 (m, 1H), 4.09 (ddd, *J* = 11.9, 6.1, 4.0 Hz, 1H), 3.81 (ddd, *J* = 11.9, 5.1, 3.8 Hz, 1H), 3.53 (s, 1H), 3.50 (s, 3H), 3.33 (s, 3H), 3.07 – 3.01 (m, 2H), 2.94 (s, 3H), 2.38 (m, 2H), 1.26 (m, 2H), 0.62 (t, *J* = 7.3 Hz, 3H).

^{13}C NMR (101 MHz, C_6D_6) δ 166.1, 165.5, 134.9, 131.1, 127.9, 127.6, 127.4, 127.2, 69.6, 63.9, 57.9, 52.4, 51.9, 44.9, 38.1, 33.4, 21.7, 13.1.

HRMS (ESI) m/z calcd. for $C_{20}H_{26}O_7S$ ([M+H] $^+$) 411.1472, found 411.1489

$[\alpha]_D^{25}$ = -18.8 (c = 1.0, CH_2Cl_2).

. [HPLC traces]

[back to Table of Contents]

2-benzyl 1,1-dimethyl (2S,3R)-3-phenyl-3-(propylthio)cyclopropane-1,1,2-tricarboxylate (3ab)

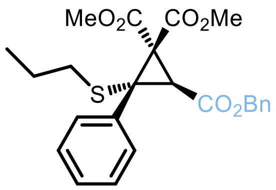

The BIMP catalysed reaction was carried with 0.1 mmol scale (1.0 eq) under room temperature and for 24 h with 10 mol% catalyst in 1 ml toluene. The reaction procedure was as follows,

Compound **1o** (1.0 eq, 0.1 mmol) and propane-1-thiol (2.0 eq, 0.2 mmol), was added into a small vial at room temperature and air. The BIMP catalyst was dissolved in 1 mL toluene and slowly added to the reaction mixture. After full conversion, as indicated by TLC analysis, the crude mixture was directly purified by flash column chromatography using pentane/EtOAc = 7/1. Giving a colourless oil (39.8 mg, 0.09 mmol 90%) and 91% ee with d.r. 10/1

^{1}H NMR (400 MHz, C_6D_6) δ 7.68 – 7.58 (m, 2H), 7.15 (s, 5H), 7.09 – 7.03 (m, 3H), 4.97 (dd, *J* = 11.9, 2.2 Hz, 1H), 4.70 (dd, *J* = 12.2, 2.7 Hz, 1H), 3.54 (s, 1H), 3.49 (s, 3H), 3.18 (s, 3H), 2.47 – 2.26 (m, 2H), 1.29 – 1.20 (m, 2H), 0.61 (t, *J* = 7.4 Hz, 3H).

^{13}C NMR (101 MHz, C_6D_6) δ 166.0, 165.4, 164.2, 135.3, 134.7, 131.0, 129.1, 128.8, 128.7, 128.3, 128.2, 128.0, 128.0, 127.9, 127.7, 127.4, 127.3, 127.2, 66.8, 52.4, 51.8, 47.9, 44.9, 38.0, 33.4, 21.7, 13.1.

HRMS (ESI) m/z calcd. for $C_{24}H_{26}O_6S$ ($[M+H]^+$) 443.1523, found 443.1521

$[\alpha]_D^{25}$ = -2.7 (c = 1.0, CH_2Cl_2).

. [HPLC traces]

[back to Table of Contents]

2-butyl 1,1-dimethyl (2S,3R)-3-phenyl-3-(propylthio)cyclopropane-1,1,2-tricarboxylate (3ac)

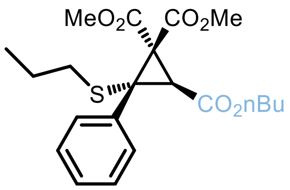

The BIMP catalysed reaction was carried with 0.1 mmol scale (1.0 eq) under room temperature and for 24 h with 10 mol% catalyst in 1 ml toluene. The reaction procedure was as follows,

Compound **1p** (1.0 eq, 0.1 mmol) and propane-1-thiol (2.0 eq, 0.2 mmol), was added into a small vial at room temperature and air. The BIMP catalyst was dissolved in 1 mL toluene and slowly added to the reaction mixture. After full conversion, as indicated by TLC analysis, the crude mixture was directly purified by flash column chromatography using pentane/EtOAc = 7/1. Giving a colourless oil (39.6 mg, 0.097 mmol, 97%) and 93% ee with d.r. 9/1

^{1}H NMR (400 MHz, C_6D_6) δ 7.79 – 7.71 (m, 2H), 7.13 (d, *J* = 1.5 Hz, 2H), 7.04 – 6.96 (m, 1H), 3.94 – 3.78 (m, 2H), 3.53 (s, 1H), 3.51 (s, 3H), 3.30 (s, 3H), 2.39 (m, 2H), 1.30 – 1.21 (m, 4H), 1.07 – 0.97 (m, 2H), 0.64 (m, 6H).

^{13}C NMR (101 MHz, C_6D_6) δ 165.6, 134.9, 131.0, 129.1, 127.9, 127.6, 127.4, 127.2, 64.9, 52.4, 51.8, 47.8, 44.7, 38.1, 33.4, 30.2, 21.7, 18.8, 13.3, 13.1.

HRMS (ESI) m/z calcd. for $C_{21}H_{28}O_6S$ ([M+H] $^+$) 409.1679, found 409.1674

$[\alpha]_D^{25}$ = -13.7 (c = 1.0, CH_2Cl_2).

. [HPLC traces]

[back to Table of Contents]

1,1-dimethyl 2-(prop-2-yn-1-yl) (2S,3R)-3-phenyl-3-(propylthio)cyclopropane-1,1,2-tricarboxylate (3ad)

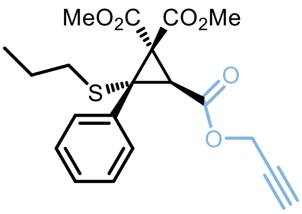

The BIMP catalysed reaction was carried with 0.1 mmol scale (1.0 eq) under room temperature and for 24 h with 10 mol% catalyst in 1 ml toluene. The reaction procedure was as follows,

Compound **1q** (1.0 eq, 0.1 mmol) and propane-1-thiol (2.0 eq, 0.2 mmol), was added into a small vial at room temperature and air. The BIMP catalyst was dissolved in 1 mL toluene and slowly added to the reaction mixture. After full conversion, as indicated by TLC analysis, the crude mixture was directly purified by flash column chromatography using pentane/EtOAc = 7/1. Giving a colourless oil (35.5 mg, 0.091 mmol, 91%) and 94% ee with d.r. 10/1

^{1}H NMR (400 MHz, C_6D_6) δ 7.74 – 7.65 (m, 2H), 7.14 – 7.08 (m, 2H), 7.03 – 6.95 (m, 1H), 4.38 (dd, *J* = 15.6, 2.5 Hz, 1H), 4.27 (s, 1H), 4.10 (dd, *J* = 15.6, 2.5 Hz, 1H), 3.49 (s, 3H), 3.43 (s, 1H), 3.27 (s, 3H), 2.36 (m, 2H), 1.30 – 1.17 (m, 2H), 0.61 (t, *J* = 7.4 Hz, 3H).

^{13}C NMR (101 MHz, C_6D_6) δ 165.3, 165.3, 164.2, 134.5, 131.0, 129.1, 127.9, 127.6, 127.4, 127.3, 77.1, 74.9, 52.4, 52.0, 51.9, 47.9, 45.3, 37.6, 33.4, 21.6, 13.1.

HRMS (ESI) m/z calcd. for $C_{20}H_{22}O_6S$ ($[M+H]^+$) 391.1210, found 391.1208

$[\alpha]_D^{25}$ = -14.3 (c = 1.0, CH_2Cl_2).

. [HPLC traces]

[back to Table of Contents]

dimethyl (2R,3S)-3-(benzylcarbamoyl)-2-phenyl-2-(propylthio)cyclopropane-1,1-dicarboxylate (3ae)

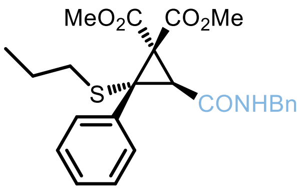

The BIMP catalysed reaction was carried with 0.1 mmol scale (1.0 eq) under room temperature and for 24 h with 10 mol% catalyst in 1 ml toluene. The reaction procedure was as follows,

Compound **1r** (1.0 eq, 0.1 mmol) and propane-1-thiol (2.0 eq, 0.2 mmol), was added into a small vial at room temperature and air. The BIMP catalyst was dissolved in 1 mL toluene and slowly added to the reaction mixture. After full conversion, as indicated by TLC analysis, the crude mixture was directly purified by flash column chromatography using pentane/EtOAc = 7/1. Giving a colourless oil (27.4 mg, 0.062 mmol 62%) and 87% ee with d.r. 10/1

^{1}H NMR (400 MHz, C_6D_6) δ 7.32 (d, *J* = 7.2 Hz, 2H), 7.00 – 6.92 (s, 1H), 6.92 – 6.80 (m, 5H), 6.79 – 6.69 (m, 3H), 4.27 (s, 3H), 3.93 (s, 2H), 3.43 (s, 3H), 3.23 (s, 1H), 2.40 – 2.31 (m, 1H), 2.13 (m, 1H), 1.10 (m, 2H), 0.54 (t, *J* = 7.4 Hz, 3H).

^{13}C NMR (101 MHz, C_6D_6) δ 164.3, 134.9, 129.0, 128.6, 128.5, 128.3, 127.9, 127.6, 127.4, 127.1, 52.3, 41.9, 37.5, 33.4, 21.7, 12.9.

HRMS (ESI) m/z calcd. for $C_{24}H_{27}NO_5S$ ([M+H] $^+$) 442.1683, found 442.1695

$[\alpha]_D^{25}$ = -33.0 (c = 1.0, CH_2Cl_2).

. [HPLC traces]

[back to Table of Contents]

methyl (1R,5S,6R)-2,4-dioxo-3,6-diphenyl-6-(propylthio)-3-azabicyclo[3.1.0]hexane-1-carboxylate (3af)

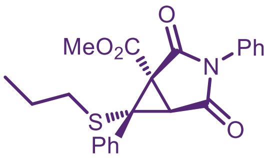

The BIMP catalysed reaction was carried with 0.1 mmol scale (1.0 eq) under room temperature and for 24 h with 10 mol% catalyst in 1 ml toluene. The reaction procedure was as follows,
Compound **1s** (1.0 eq, 0.1 mmol) and propane-1-thiol (2.0 eq, 0.2 mmol), was added into a small vial at room temperature and air. The BIMP catalyst was dissolved in 1 mL toluene and slowly added to the reaction mixture. After full conversion, as indicated by TLC analysis, the crude mixture was directly purified by flash column chromatography using pentane/EtOAc = 7/1. Giving a colourless oil (30.8 mg, 0.078 mmol, 78%) and 91% ee, dr > 20:1

^{1}H NMR (400 MHz, C_6D_6) δ 7.50 (s, 1H), 6.97 – 6.74 (m, 8H), 6.37 (dt, *J* = 6.9, 1.4 Hz, 2H), 3.50 (s, 3H), 3.40 (s, 1H), 2.46 (dt, *J* = 12.1, 7.2 Hz, 1H), 2.23 (dt, *J* = 12.0, 7.3 Hz, 1H), 1.21 – 1.14 (m, 2H), 0.58 (t, *J* = 7.3 Hz, 3H).

^{13}C NMR (101 MHz, C_6D_6) δ 168.2, 134.7, 131.3, 128.9, 128.5, 128.4, 128.1, 127.9, 127.6, 127.4, 126.4, 52.4, 37.6, 33.5, 21.7, 12.9.

HRMS (ESI) m/z calcd. for $C_{22}H_{21}NO_4S$ ([M+H] $^+$) 396.1264, found 396.1264

$[\alpha]_D^{25}$ = -26.5 (c = 1.0, CH_2Cl_2).

. [HPLC traces]

[back to Table of Contents]

methyl (1S,5R,6S)-3-cyclohexyl-2,4-dioxo-6-phenyl-6-(propylthio)-3-azabicyclo[3.1.0]hexane-1-carboxylate (3ag)

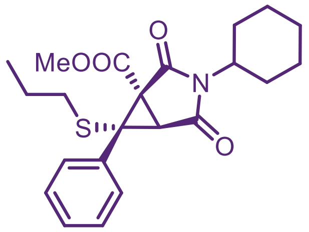

The BIMP catalysed reaction was carried with 0.1 mmol scale (1.0 eq) under room temperature and for 24 h with 10 mol% catalyst in 1 ml toluene. The reaction procedure was as follows,

Compound **1t** (1.0 eq, 0.1 mmol) and propane-1-thiol (2.0 eq, 0.2 mmol), was added into a small vial at room temperature and air. The BIMP catalyst was dissolved in 1 mL toluene and slowly added to the reaction mixture. After full conversion, as indicated by TLC analysis, the crude mixture was directly purified by flash column chromatography using pentane/EtOAc = 4/1 to give a colourless oil (32.5 mg, 0.081 mmol, 81%) and 93% ee, d.r. > 20:1

^{1}H NMR (400 MHz, $CDCl_3$) δ 7.47 (s, 2H), 7.01 – 6.78 (m, 3H), 3.60 (tt, *J* = 12.3, 3.9 Hz, 1H), 3.47 (s, 3H), 3.28 (s, 1H), 2.47 (ddd, *J* = 11.8, 7.8, 7.0 Hz, 1H), 2.22 (ddd, *J* = 11.8, 7.5, 6.9 Hz, 1H), 1.83 – 1.68 (m, 2H), 1.42 – 1.29 (m, 2H), 1.25 – 1.08 (m, 3H), 0.98 – 0.73 (m, 3H), 0.65 (dt, *J* = 3.9, 2.0 Hz, 2H), 0.59 (t, *J* = 7.4 Hz, 3H).

^{13}C NMR (101 MHz, $CDCl_3$) δ 169.5, 168.2, 163.0, 134.9, 129.8, 128.7, 128.5, 52.4, 51.4, 50.3, 47.2, 37.5, 33.6, 27.9, 25.5, 24.8, 21.8, 13.1.

HRMS (ESI) m/z calcd. for $C_{21}H_{19}NO_6$ ([M+H] $^+$) 402.1734, found 402.1735.

$[\alpha]_D^{25}$ = -50.1 (c = 1.3, CH_2Cl_2).

[back to Table of Contents]

methyl (5R,6S)-3-(4-methoxyphenyl)-2,4-dioxo-6-phenyl-6-(propylthio)-3-azabicyclo[3.1.0]hexane-1-carboxylate (3ah)

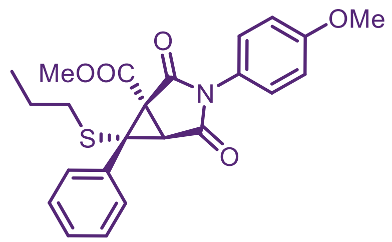

The BIMP catalysed reaction was carried with 0.1 mmol scale (1.0 eq) under room temperature and for 24 h with 10 mol% catalyst in 1 ml toluene. The reaction procedure was as follows,

Compound **1u** (1.0 eq, 0.1 mmol) and propane-1-thiol (2.0 eq, 0.2 mmol), was added into a small vial at room temperature and air. The BIMP catalyst was dissolved in 1 mL toluene and slowly added to the reaction mixture. After full conversion, as indicated by TLC analysis, the crude mixture was directly purified by flash column chromatography using pentane/EtOAc = 4/1 to give a colourless oil (33.2 mg, 0.078 mmol, 78%) and 90% ee, dr > 20:1

^{1}H NMR (400 MHz, C_6D_6) δ 7.54 (s, 2H), 6.97 – 6.87 (m, 3H), 6.52 – 6.43 (m, 2H), 6.31 – 6.23 (m, 2H), 3.52 (s, 1H), 3.43 (s, 3H), 3.07 (s, 1H), 2.61 – 2.38 (m, 1H), 2.32 – 2.15 (m, 1H), 1.20 (hd, *J* = 7.2, 1.2 Hz, 2H), 0.61 (t, *J* = 7.4 Hz, 3H).

^{13}C NMR (101 MHz, $CDCl_3$) δ 169.0, 167.8, 163.2, 159.9, 135.3, 129.9, 129.23, 128.9, 127.9 124.3, 114.3, 54.76, 52.8, 50.7, 47.7, 37.9, 33.9, 22.1, 13.4.

HRMS (ESI) m/z calcd. for C21H19NO6 ([M+K] $^{+}$) 464.0929, found 464.0951.

$[\alpha]_D^{25}$ = -33.6 (c = 0.5, CH_2Cl_2).

[back to Table of Contents]

trimethyl (2S,3R)-3-phenyl-3-(propylsulfonyl)cyclopropane-1,1,2-tricarboxylate (4a)

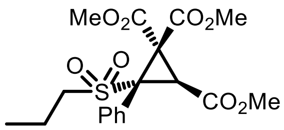

Following on general procedure G10, After full conversion, as indicated by TLC analysis, the crude mixture was directly purified by flash column chromatography using pentane/EtOAc = 2/1. Giving a colourless oil 87% yield and 93% ee

^{1}H NMR (400 MHz, $CDCl_3$) δ 7.64 (d, *J* = 7.1 Hz, 2H), 7.44 – 7.34 (m, 3H), 3.87 (s, 3H), 3.75 (s, 1H), 3.71 (s, 3H), 3.62 (s, 3H), 2.98 (m, 2H), 1.87 – 1.60 (m, 2H), 0.96 (t, *J* = 7.4 Hz, 3H).

^{13}C NMR (101 MHz, $CDCl_3$) δ 165.2, 164.8, 163.7, 133.1, 129.8, 127.9, 126.2, 77.3, 77.0, 76.7, 59.4, 53.8, 53.4, 52.7, 51.4, 43.8, 31.6, 14.6, 13.1.

HRMS (ESI) m/z calcd. for $C_{18}H_{22}O_8S$ ([M+H] $^+$) 399.1108, found 399.1119

$[\boldsymbol{\alpha}]_D^{25}$ = -8.6 (c = 1.0, CH_2Cl_2).

. [HPLC traces]

[back to Table of Contents]

trimethyl (2S,3R)-3-phenyl-3-((R)-propylsulfinyl)cyclopropane-1,1,2-tricarboxylate (4b)

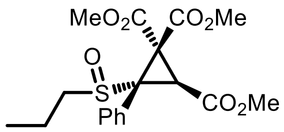

Following on general procedure G11, After full conversion, as indicated by TLC analysis, the crude mixture was directly purified by flash column chromatography using pentane/EtOAc = 2/1. Giving a colourless oil in total (32.1 mg, 0.084 mmol, 42%) and 93% ee

^{1}H NMR (400 MHz, C_6D_6) δ 7.13 – 6.91 (m, 5H), 4.07 (s, 1H), 3.67 (s, 3H), 3.32 (s, 3H), 3.07 (s, 3H), 2.32 – 2.21 (m, 1H), 2.13 (m, 1H), 1.55 – 1.39 (m, 2H), 0.52 (t, J = 7.4 Hz, 3H).

^{13}C NMR (101 MHz, C_6D_6) δ 189.0, 170.2, 150.5, 135.4, 128.6, 128.4, 127.9, 127.6, 127.4, 52.0, 51.8, 51.2, 27.6, 15.3, 12.6.

HRMS (ESI) m/z calcd. for $C_{18}H_{22}O_7S$ ([M+Na] $^+$) 405.0978, found 405.0979

$[\alpha]_D^{25}$ = -38.6 (c = 1.0, CH_2Cl_2).

. [HPLC traces]

[back to Table of Contents]

trimethyl (2S,3R)-3-phenyl-3-((R)-propylsulfinyl)cyclopropane-1,1,2-tricarboxylate (4b')

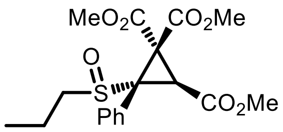

Following on general procedure G11, After full conversion, as indicated by TLC analysis, the crude mixture was directly purified by flash column chromatography using pentane/EtOAc = 2/1. Giving a colourless oil in total (34.4 mg, 0.09 mmol, 45%) and 82% ee

^{1}H NMR (400 MHz, C_6D_6) δ 7.14 – 6.98 (m, 5H), 4.08 (s, 1H), 3.57 (s, 3H), 3.18 (s, 3H), 3.12 (s, 3H), 3.09 – 3.02 (m, 1H), 2.76 (m, 1H), 1.64 – 1.47 (m, 2H), 0.50 (t, *J* = 7.4 Hz, 3H).

^{13}C NMR (101 MHz, C_6D_6) δ 199.4, 175.6, 164.9, 133.6, 129.2, 127.9, 127.6, 127.4, 53.0, 52.4, 51.6, 51.3, 31.9, 14.5, 12.5.

HRMS (ESI) m/z calcd. for $C_{18}H_{22}O_7S$ ($[M+H]^+$) 383.1159, found 383.1167

$[\alpha]_D^{25}$ = -13.7 (c = 1.0, CH_2Cl_2).

. [HPLC traces]

[back to Table of Contents]

trimethyl (2S)-3-phenylcyclopropane-1,1,2-tricarboxylate (4c)

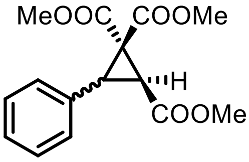

Following on general procedure G12, after full conversion, as indicated by TLC analysis, the crude mixture was directly purified by preparative TLC (1:4 EtOAc/hexane) yielding **4c** as a colourless oil (2.0 mg, 0.0068 mmol) in a 50% yield with a 3:2 d.r. (trans/cis) and with 93% ee. The data is in good agreement with the literature reported compound.[19]

^{1}H NMR (500 MHz, C_6D_6) δ 7.52 (d, J = 8.4 Hz, 1H, major), 7.15 – 6.95 (m, 4H, major), 3.89 (d, J = 7.3 Hz, 1H, major), 3.54 (s, 3H, major), 3.49 (d, J = 7.5 Hz, 1H, major), 3.29 (s, 3H, major), 2.99 (s, 3H, major).

^{13}C NMR (126 MHz, C_6D_6) δ 169.6, 169.5, 167.6, 166.4, 165.7, 164.9, 133.4, 132.1, 131.0, 129.0, 128.5, 127.5, 52.9, 52.8, 52.4, 52.2, 52.0, 51.6, 45.1, 40.2, 36.6, 36.5, 31.6, 31.2.

HRMS (ESI) m/z calcd. for $C_{15}H_{17}O_6$ ($[M+H]^+$) 293.1020, found 293.1020

$[\alpha]_D^{25}$ = +20.1 (c = 0.8, Et_2O).

[back to Table of Contents]

trimethyl 2-(4-methoxyphenyl)-5-phenylfuran-3,3,4(2H)-tricarboxylate (4d)

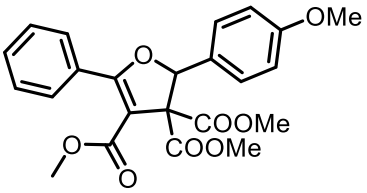

Following on general procedure G13, after full conversion, as indicated by TLC analysis, the crude mixture was directly purified by flash column chromatography using a pentane/ethyl acetate gradient from 5% ethyl acetate/95% pentane to 30% ethyl acetate/70% pentane. Giving a colourless oil in total (22 mg, 85%) and racemic.

^{1}H NMR (500 MHz, $CDCl_3$) δ 8.04 – 7.97 (m, 2H), 7.52 – 7.45 (m, 2H), 7.11 (qd, J = 3.9, 1.7 Hz, 3H), 6.75 – 6.69 (m, 2H), 6.49 (s, 1H), 3.48 (s, 3H), 3.42 (s, 3H), 3.24 (s, 3H), 3.00 (s, 3H).
^{13}C NMR (126 MHz, $CDCl_3$) δ 170.6, 167.7, 166.9, 164.7, 160.4, 131.0, 130.0, 129.9, 128.8, 128.5, 128.4, 113.8, 104.7, 87.5, 72.2, 54.7, 53.0, 52.0, 51.0.
HRMS (ESI) m/z calcd. for C21H19NO6 ($[M+Na]^+$) 449.1207, found 449.1219.

[back to Table of Contents]

NMR Spectra

Catalyst Synthesis Intermediates

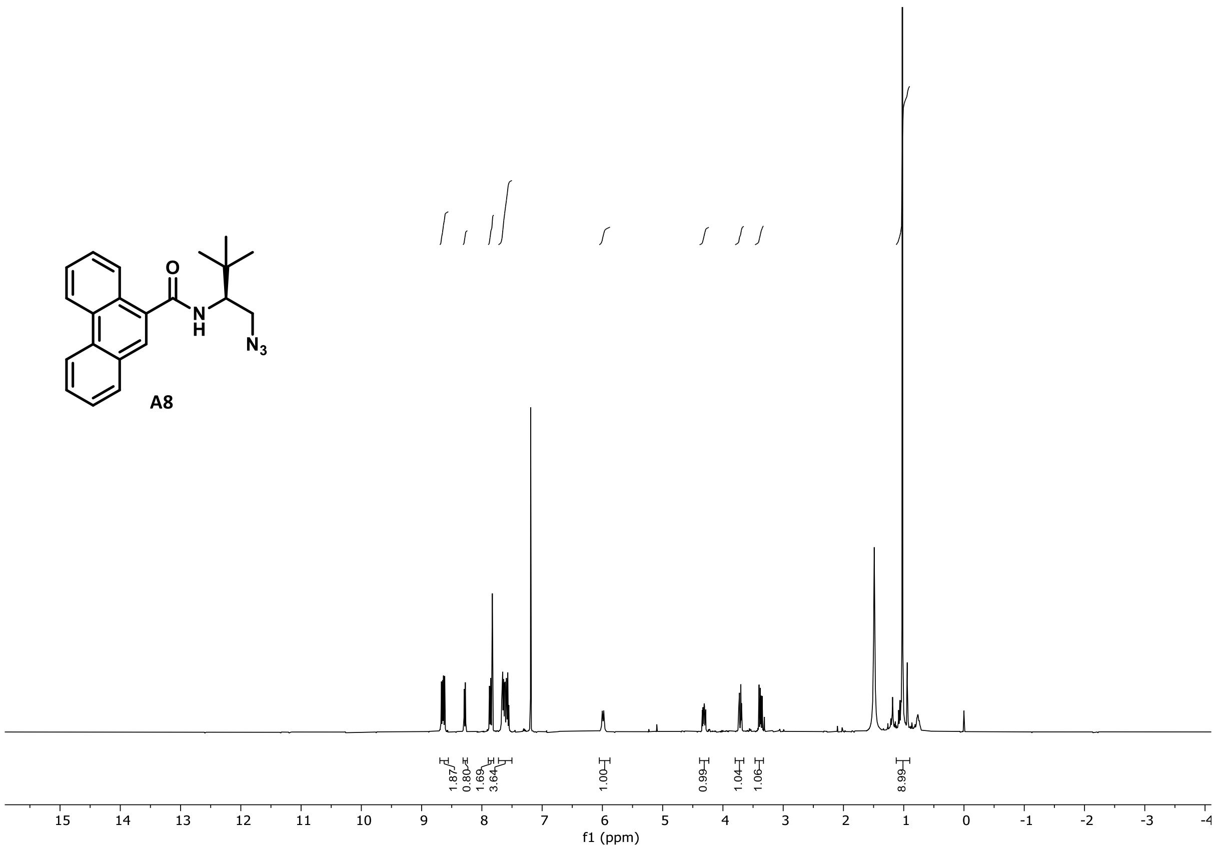

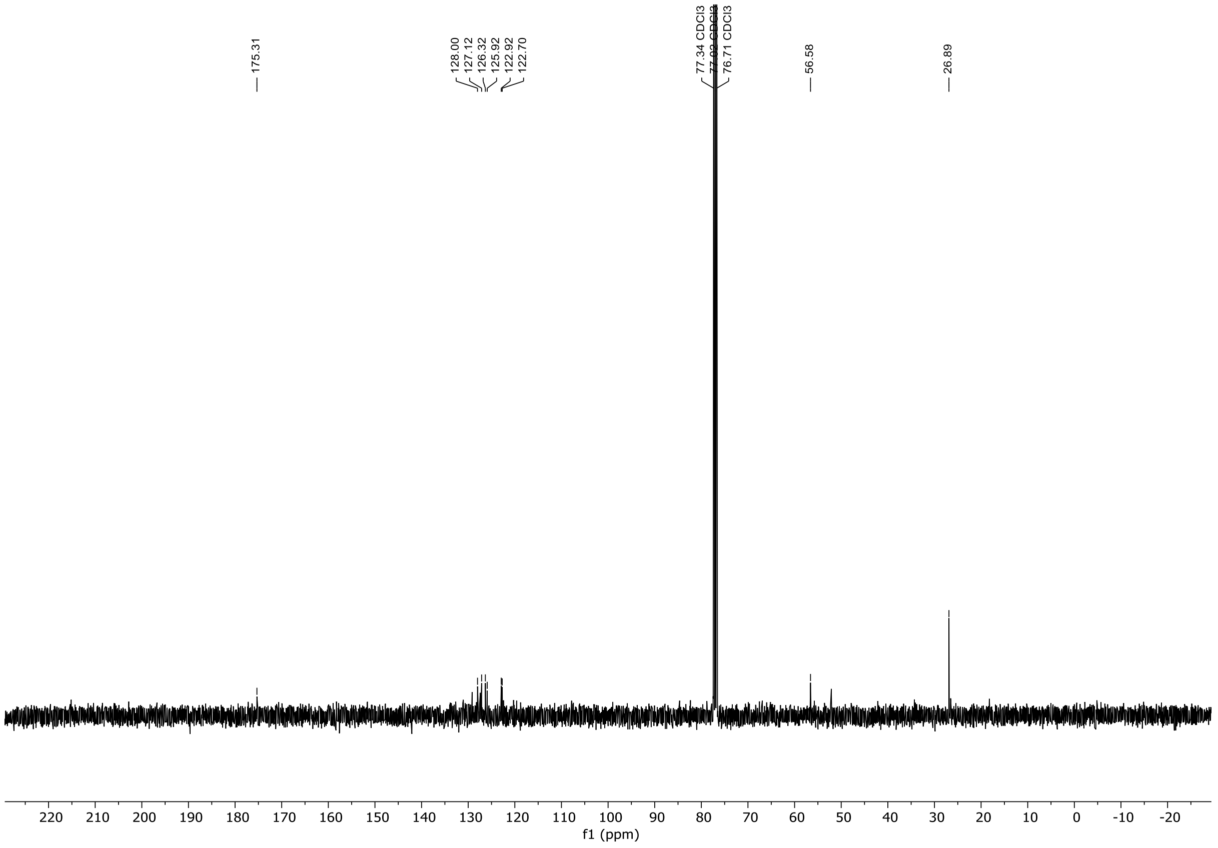

[back to Table of Contents]

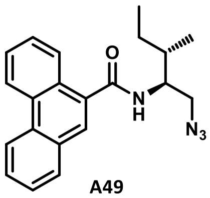

A49

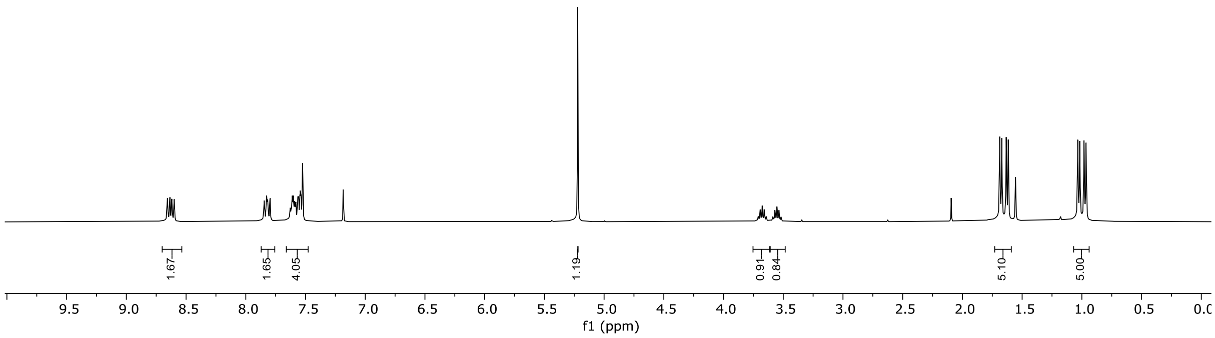

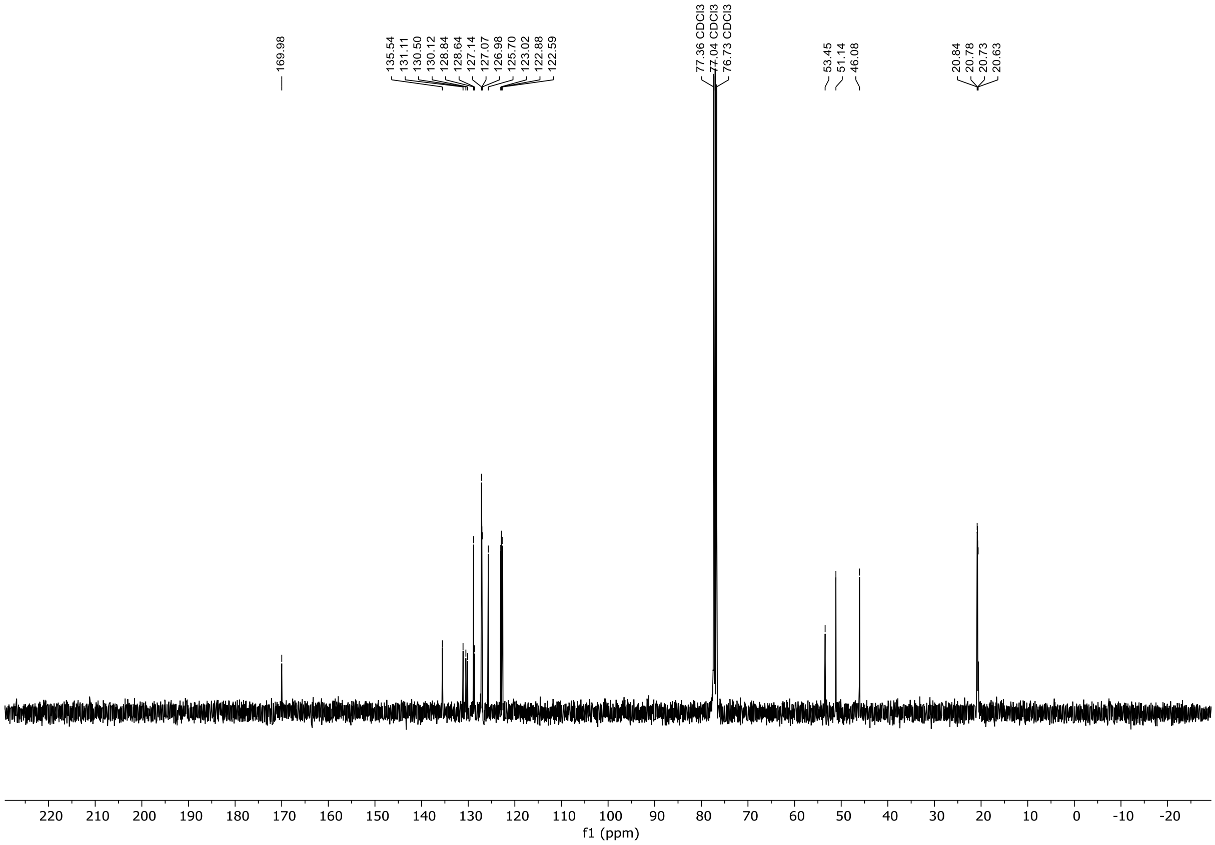

[back to Table of Contents]

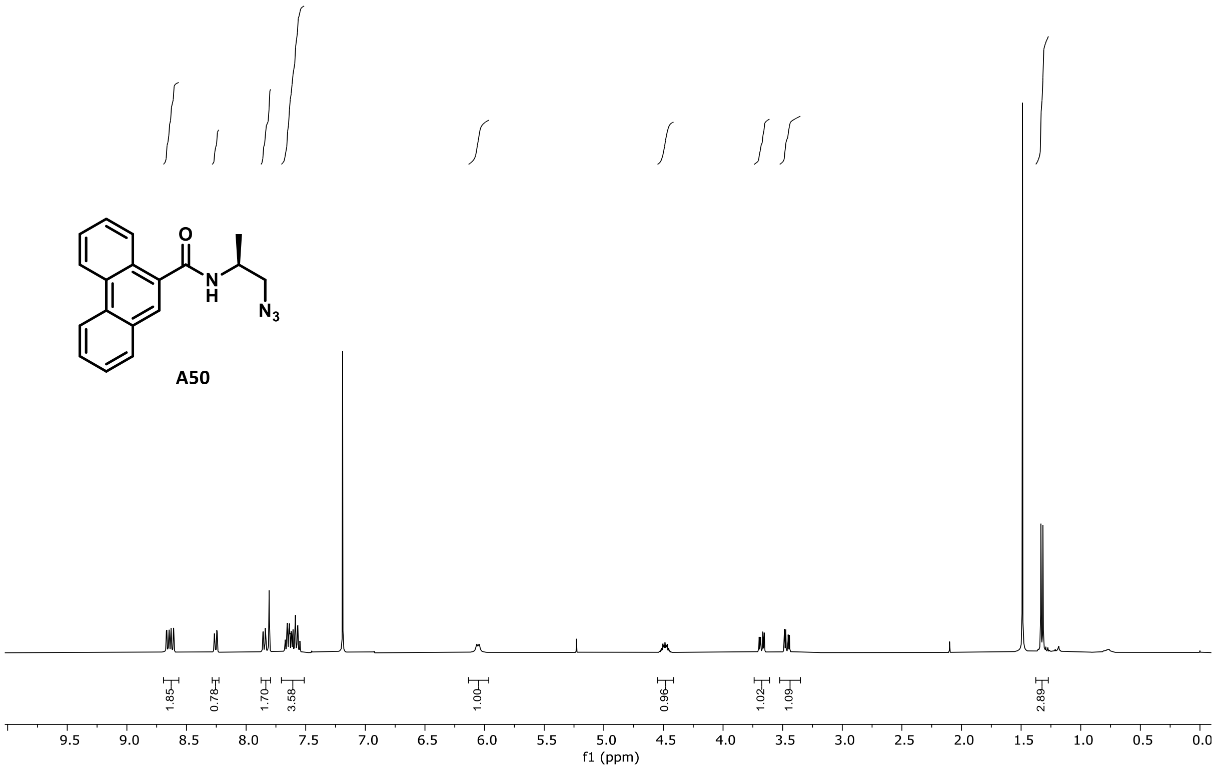

A50

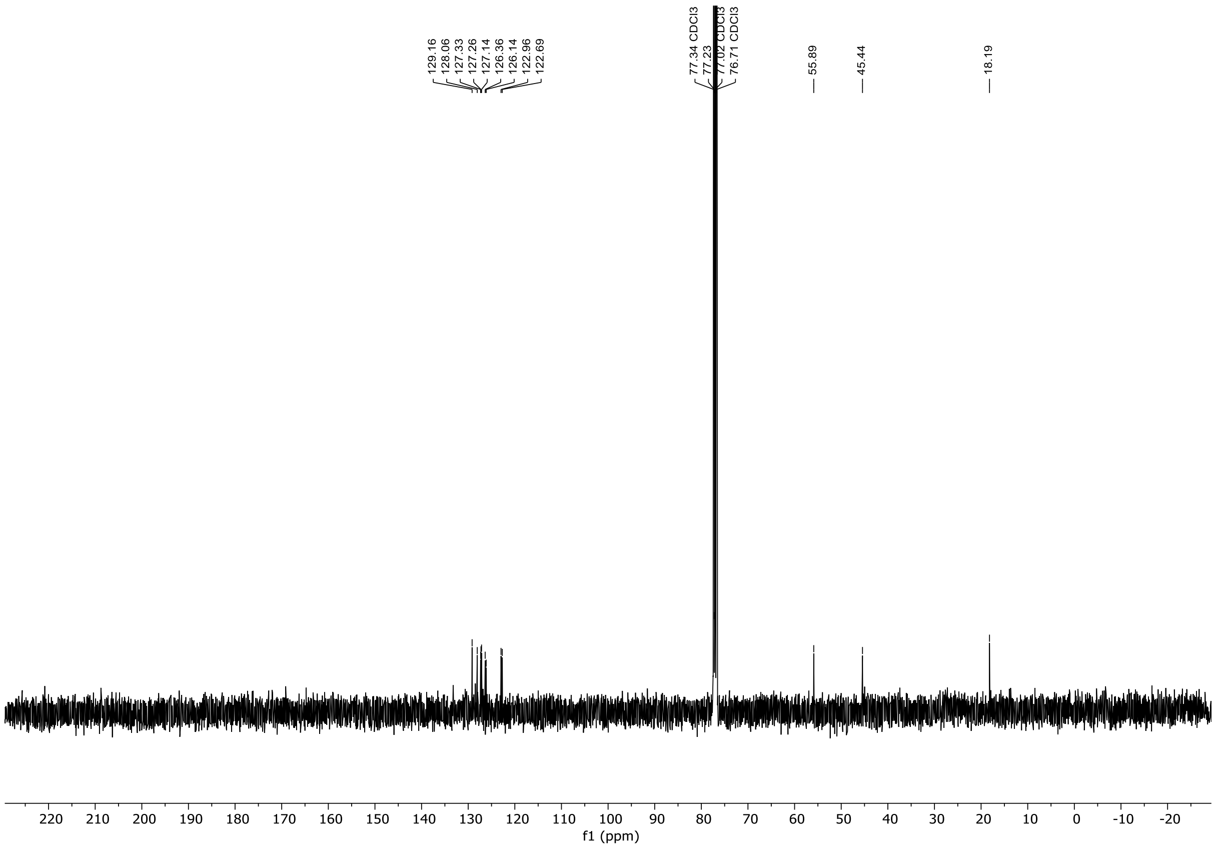

[back to Table of Contents]

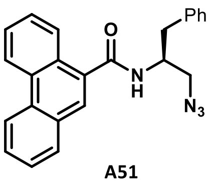

A51

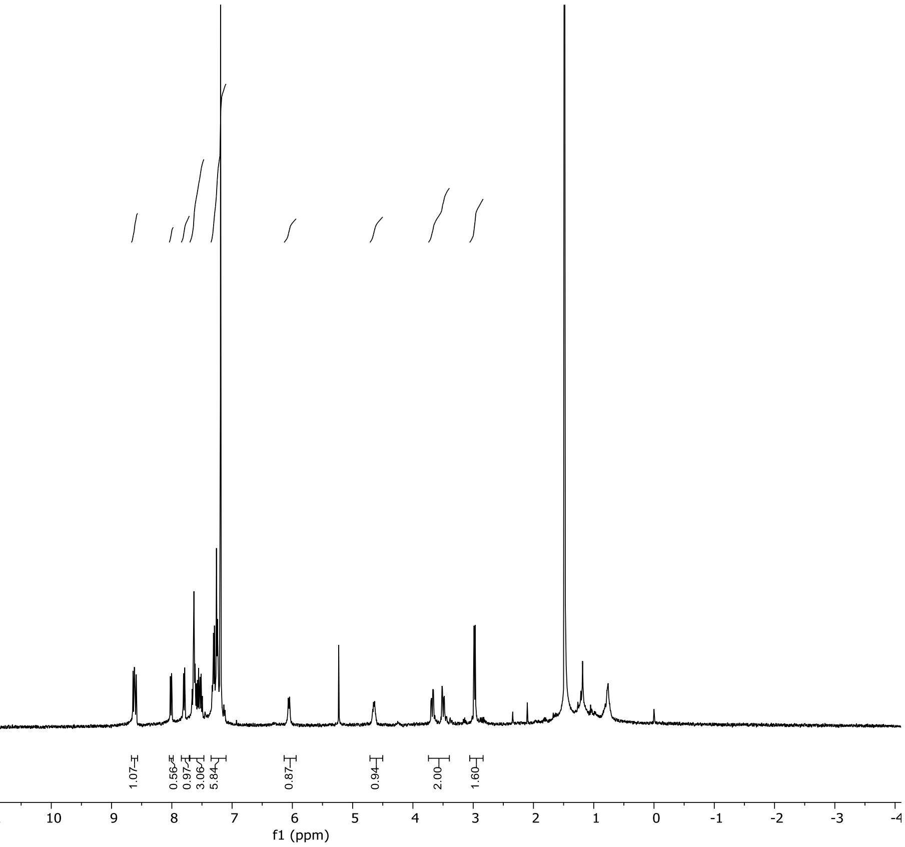

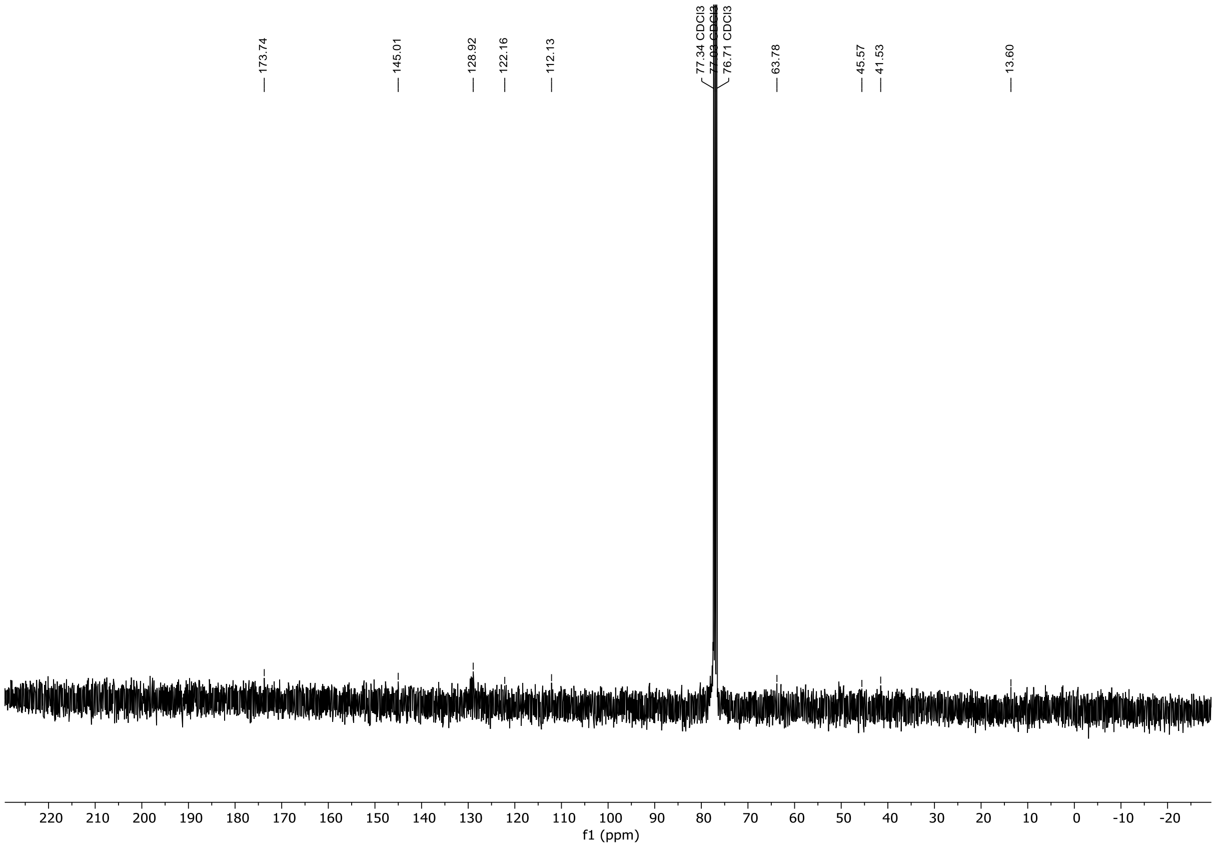

[back to Table of Contents]

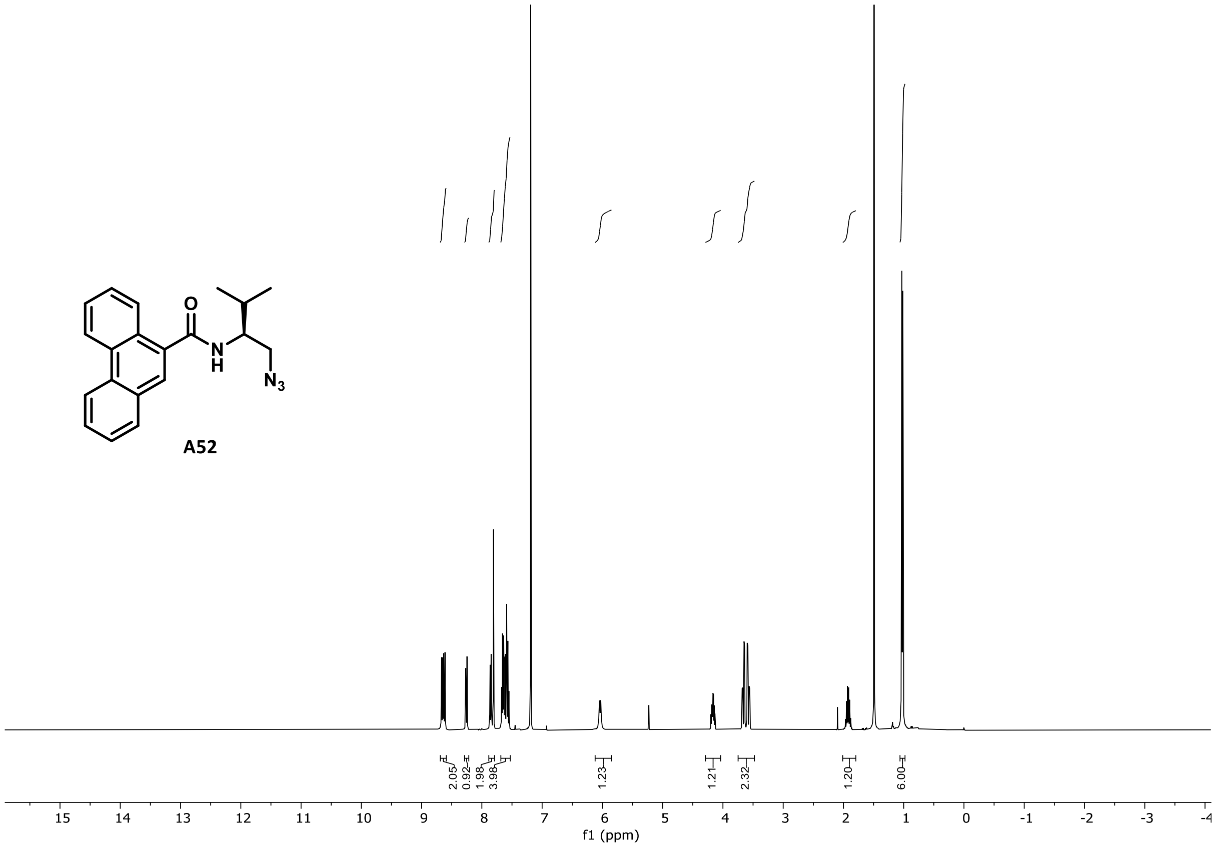

A52

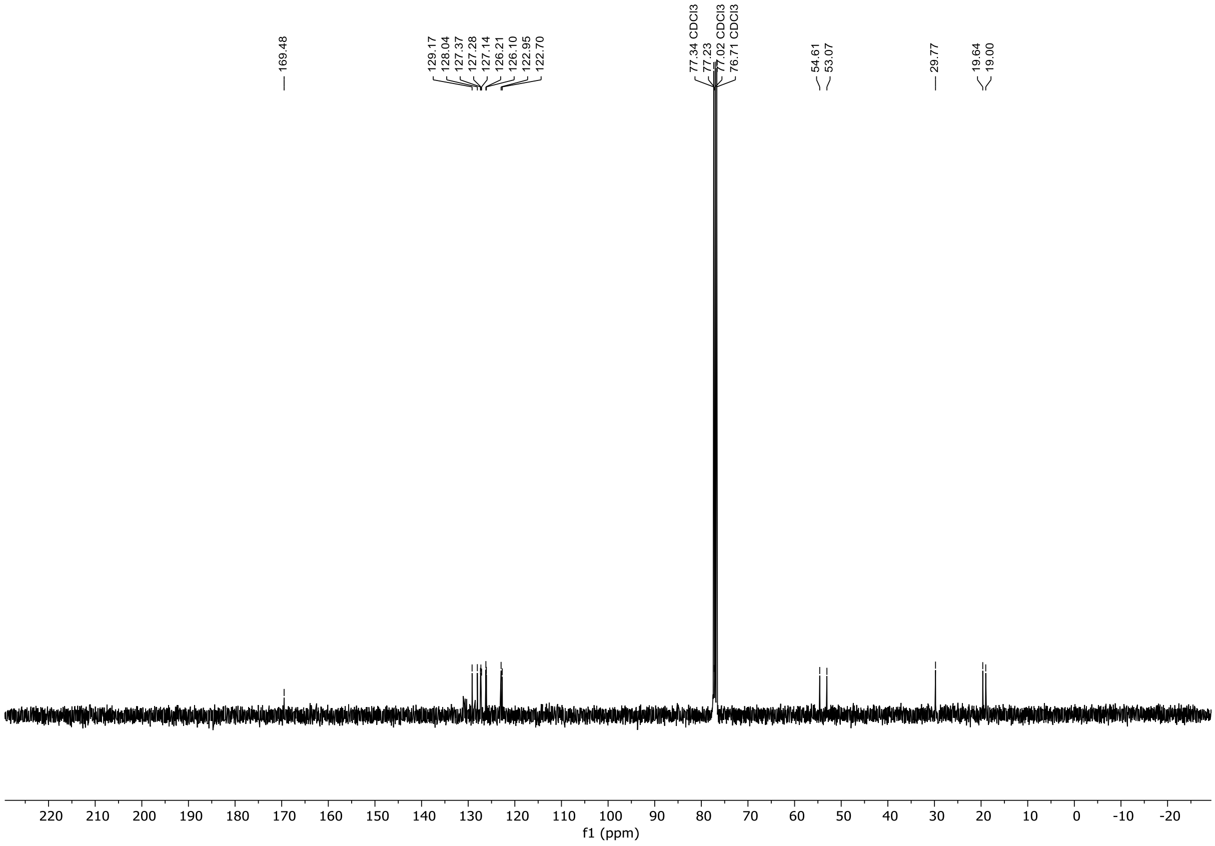

[back to Table of Contents]

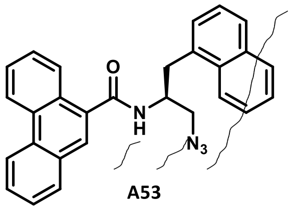

A53

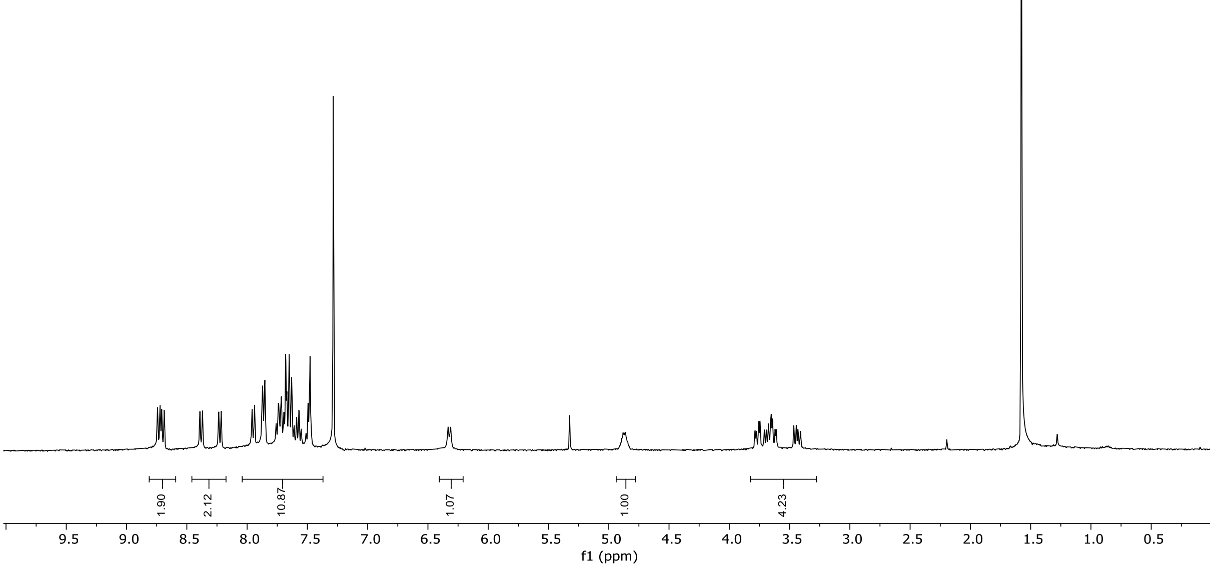

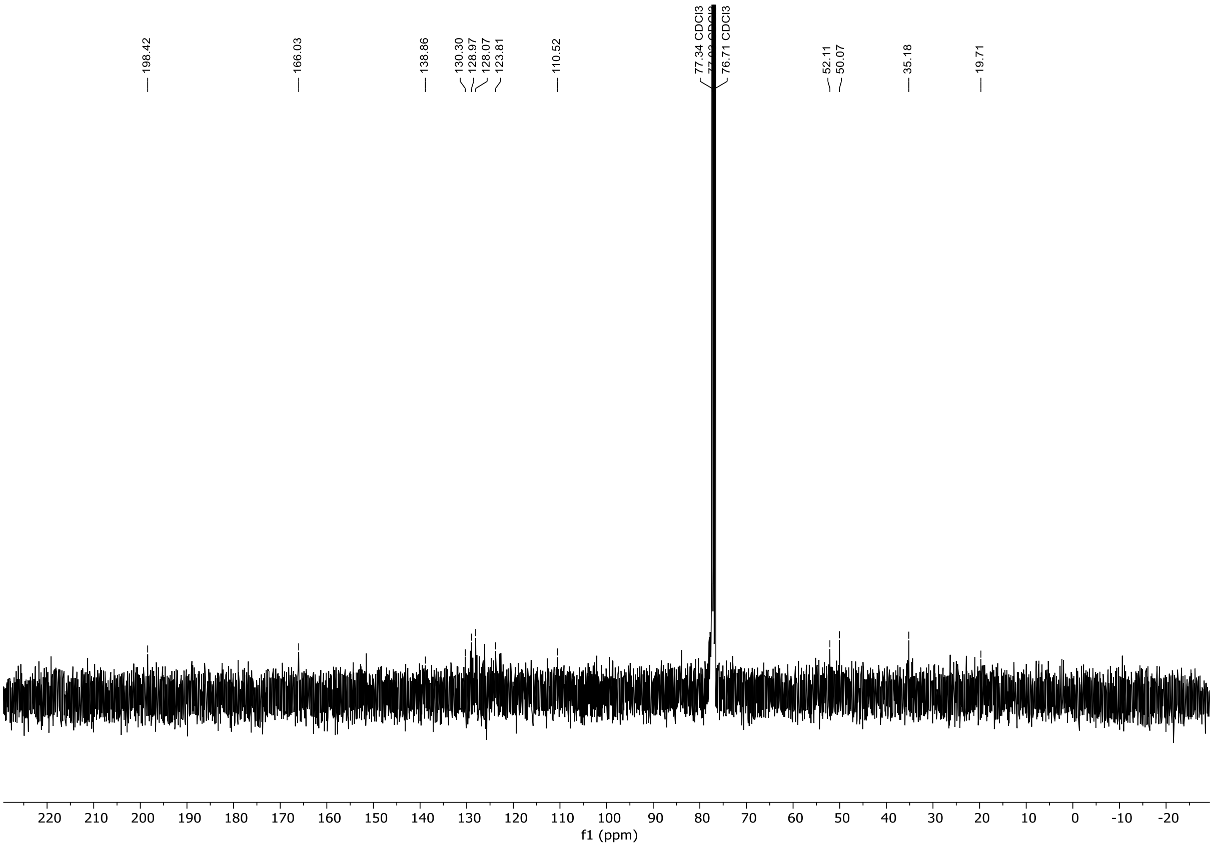

[back to Table of Contents]

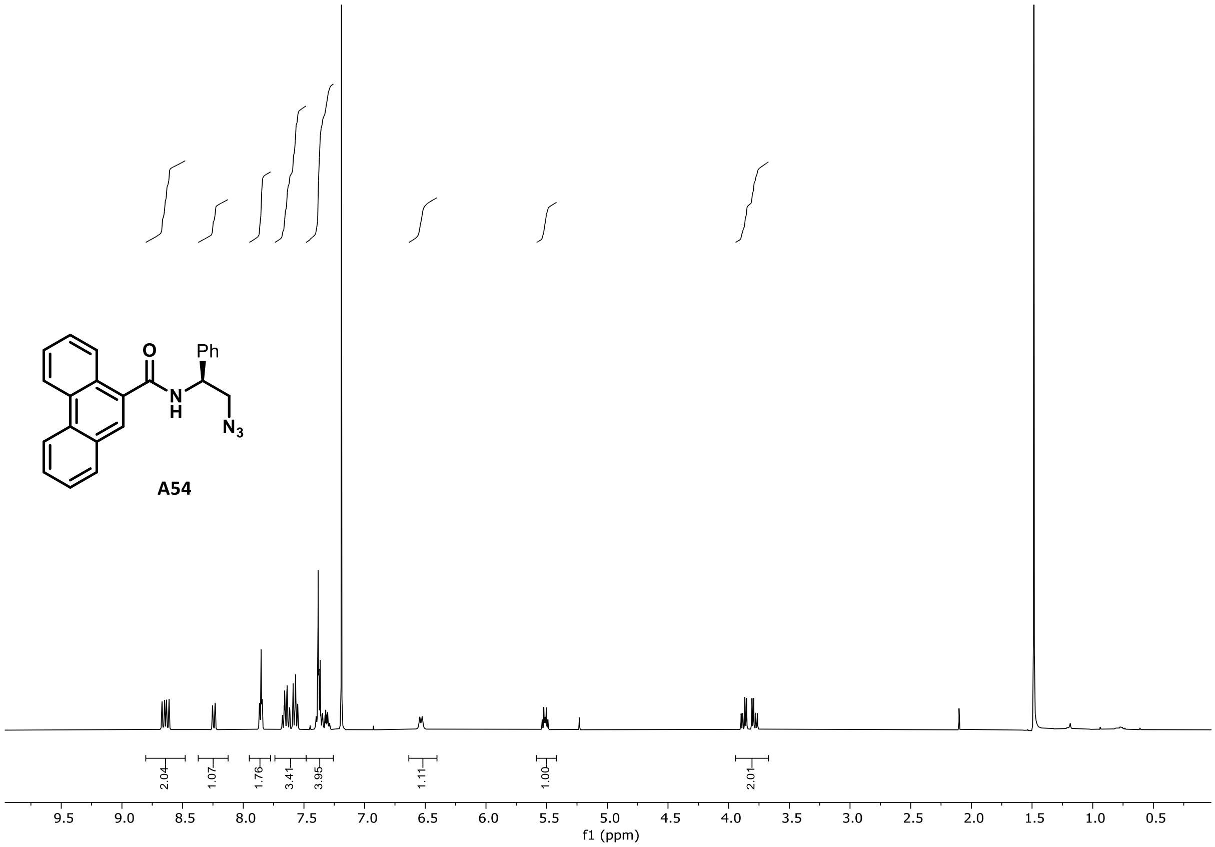

A54

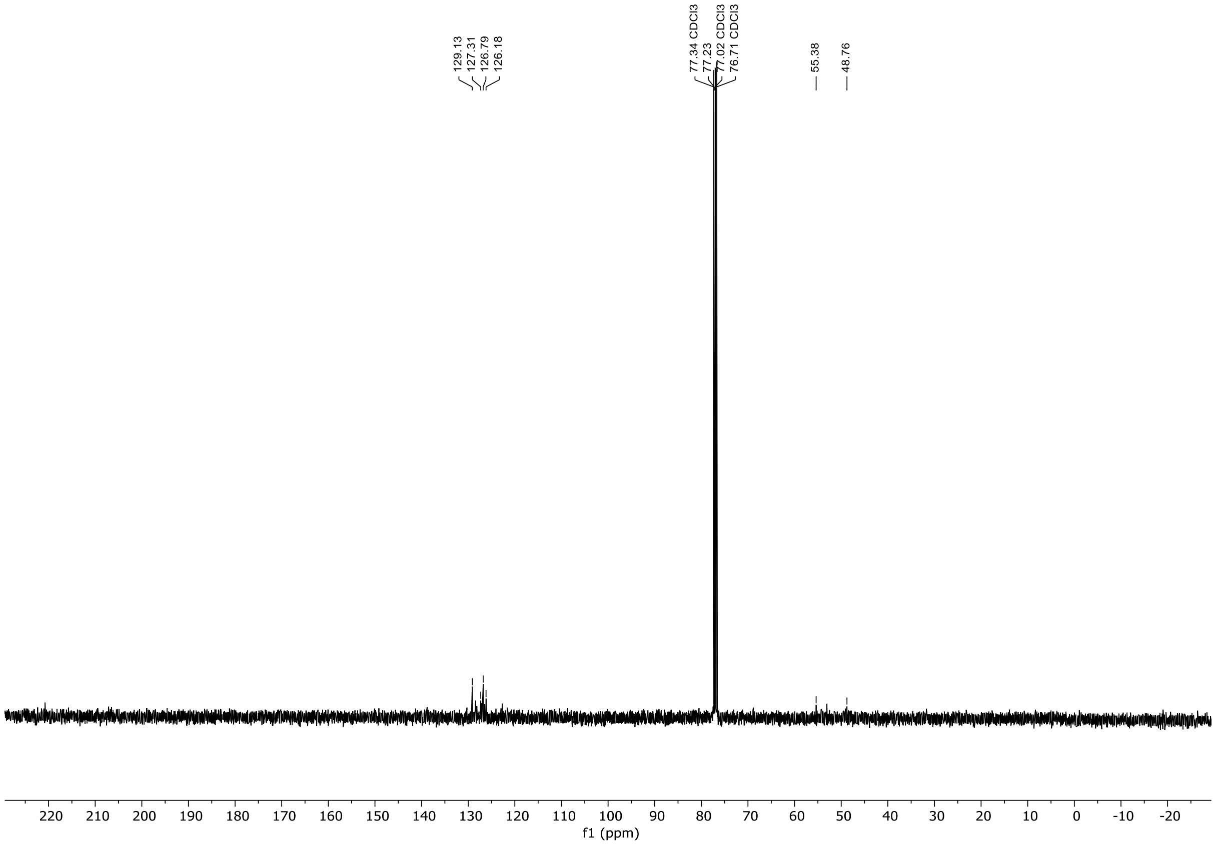

[back to Table of Contents]

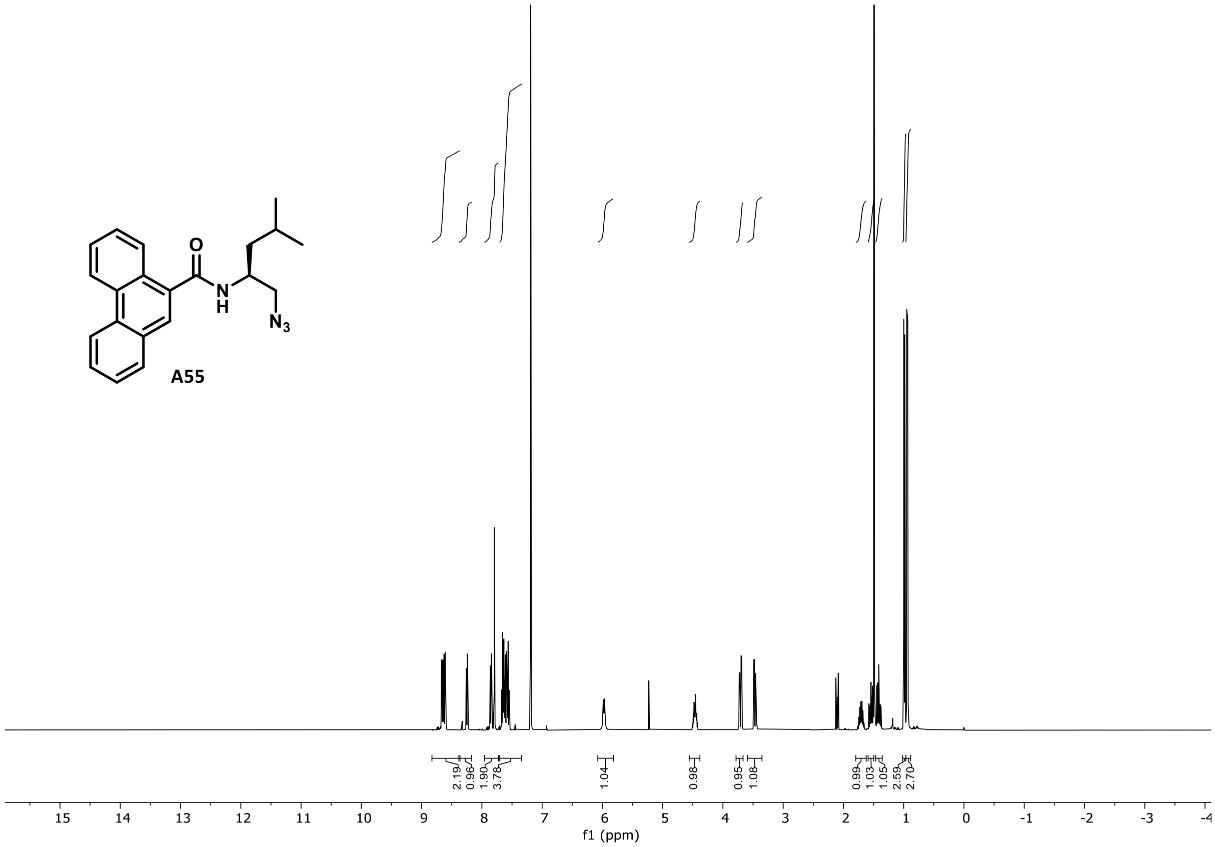

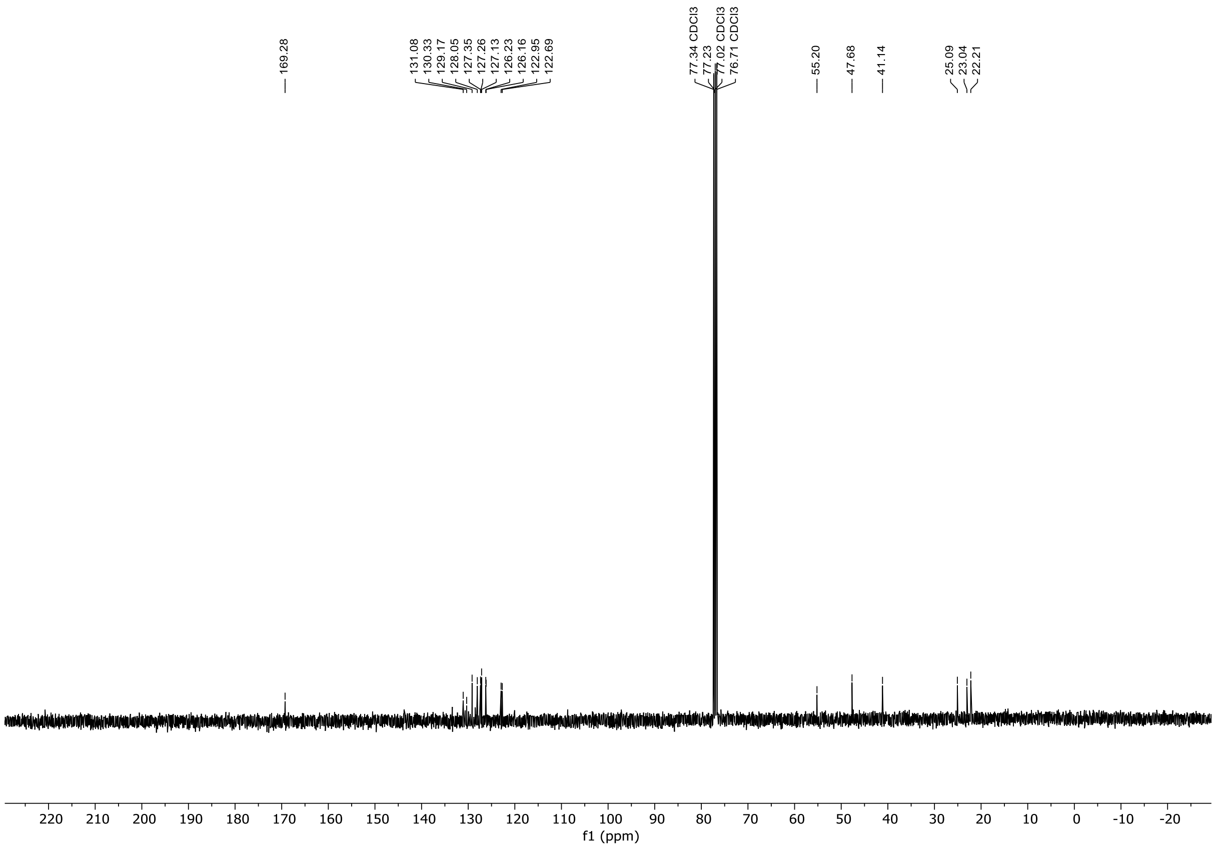

[back to Table of Contents]

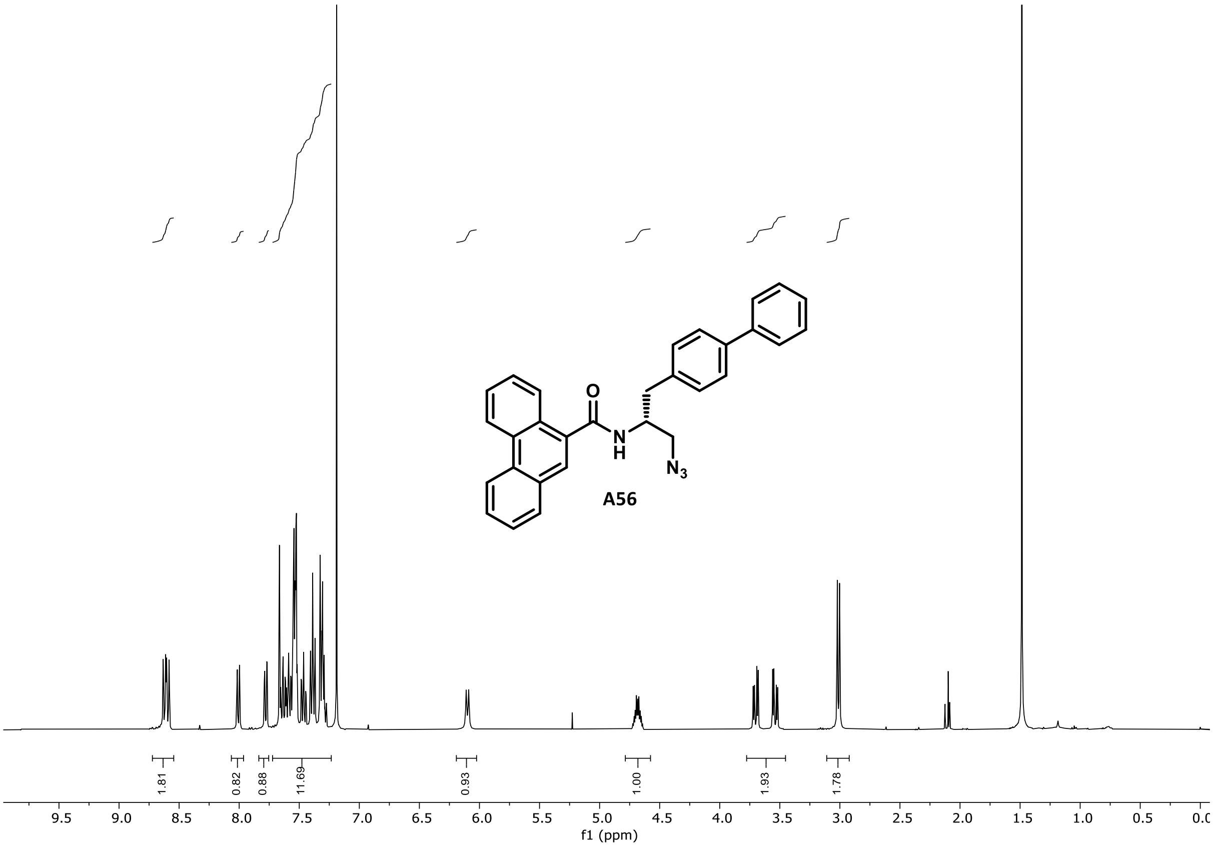

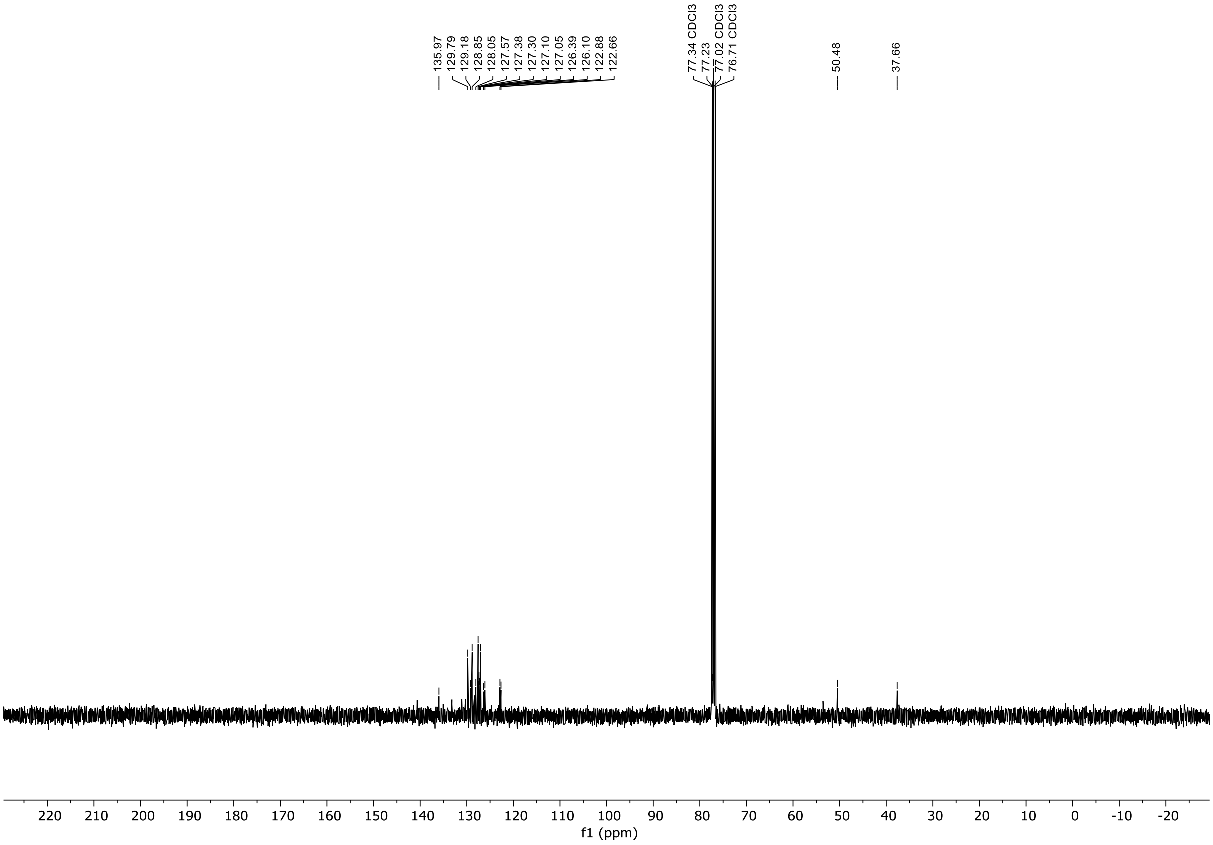

[back to Table of Contents]

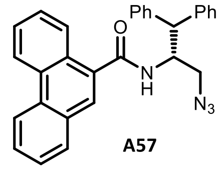

A57

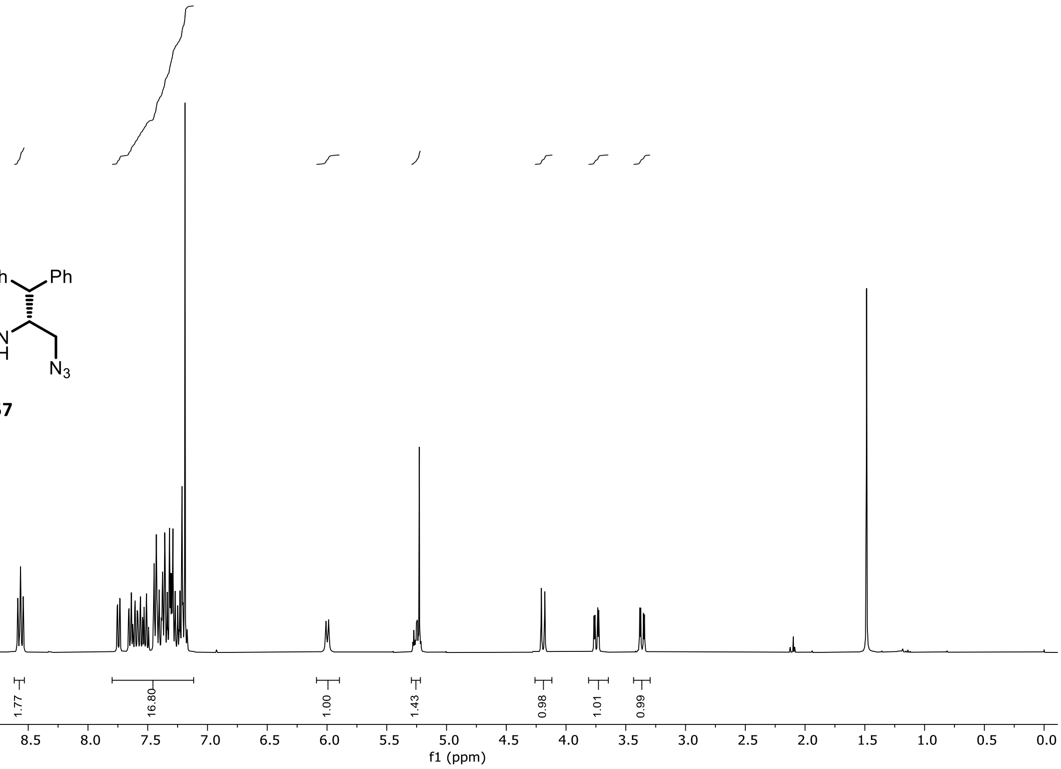

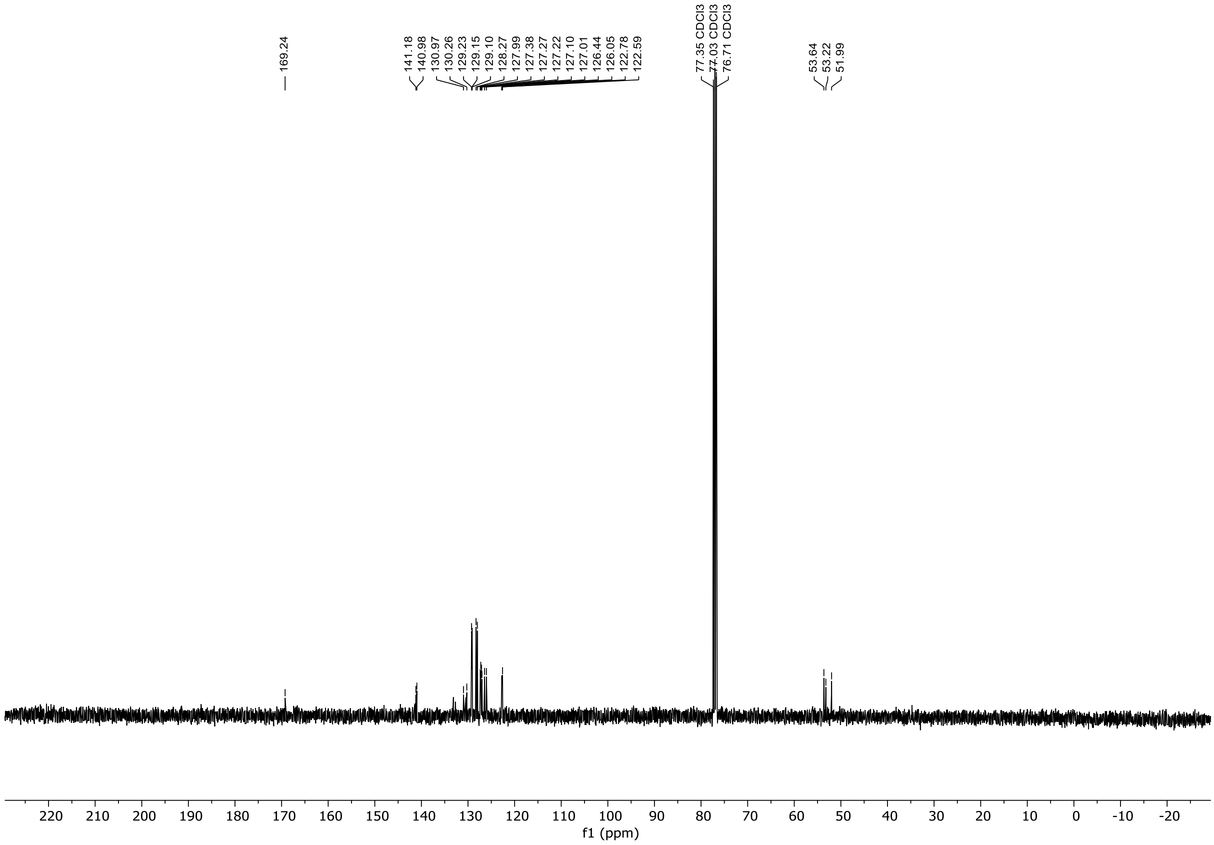

[back to Table of Contents]

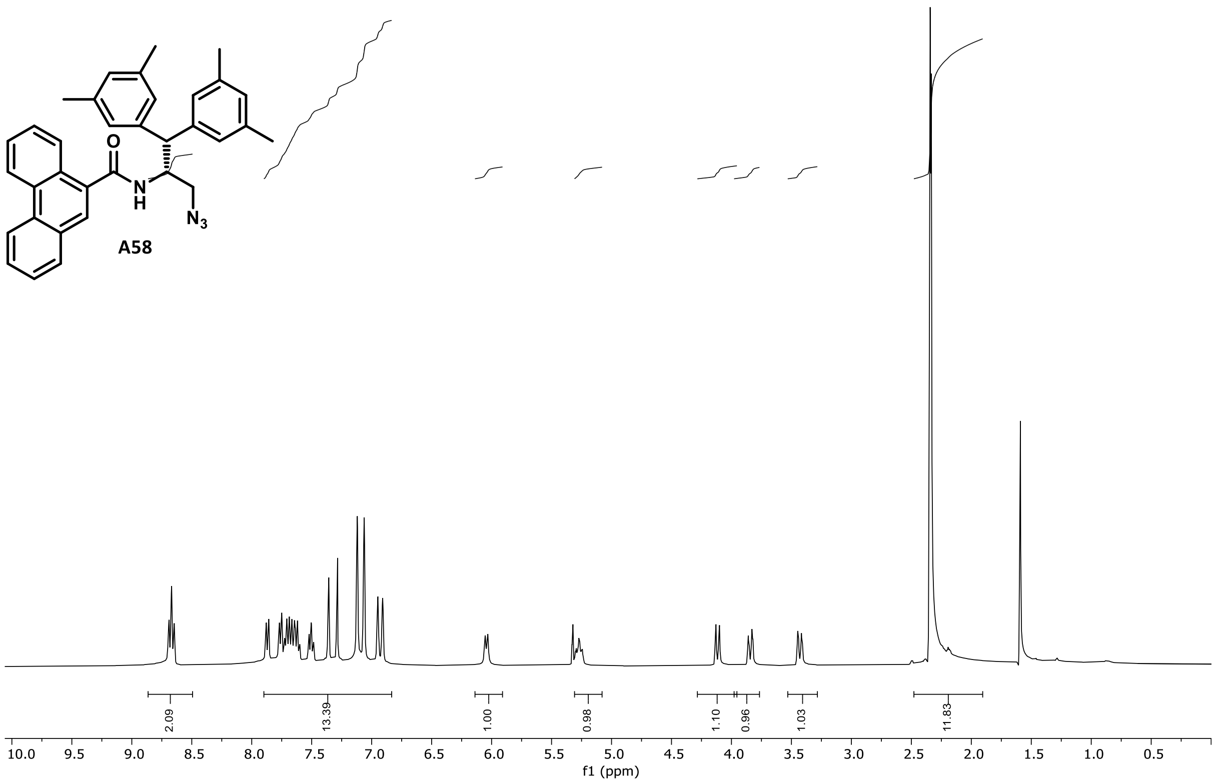

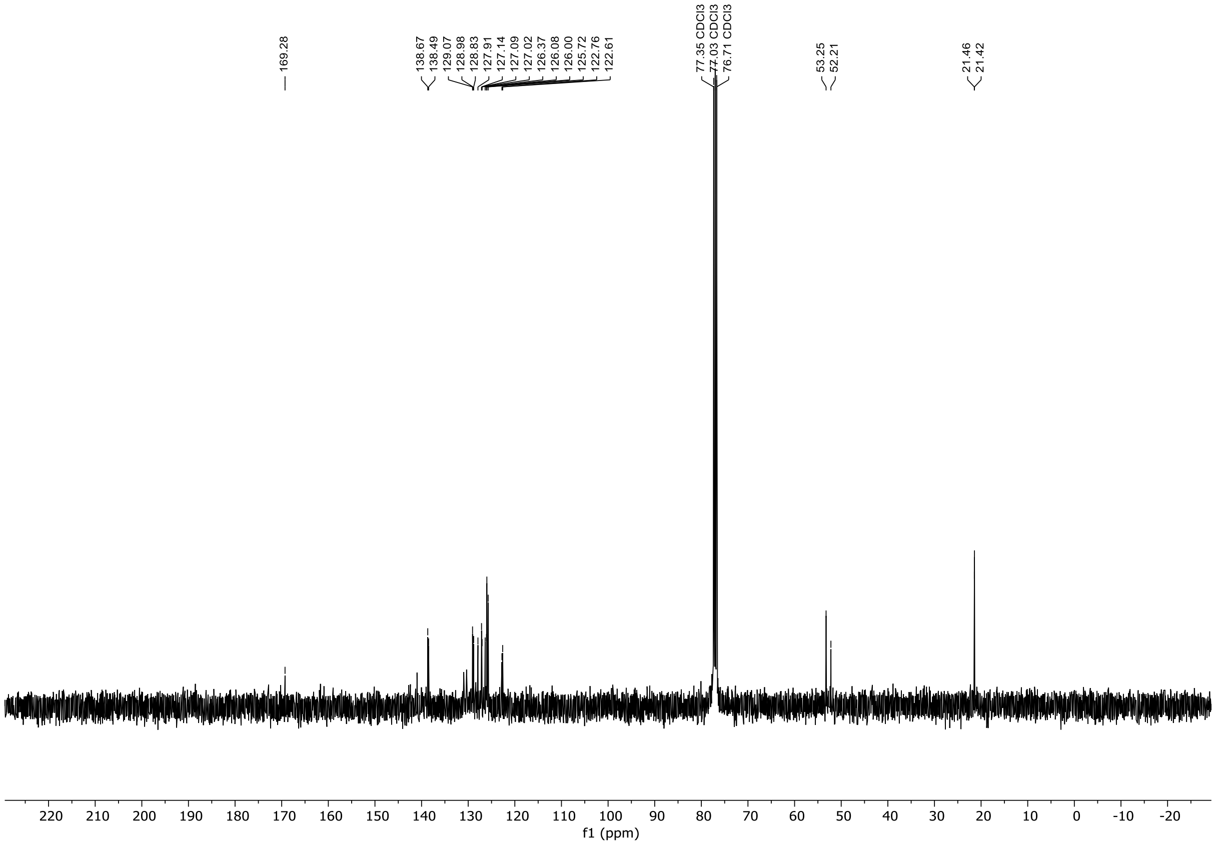

[back to Table of Contents]

Starting material and Products

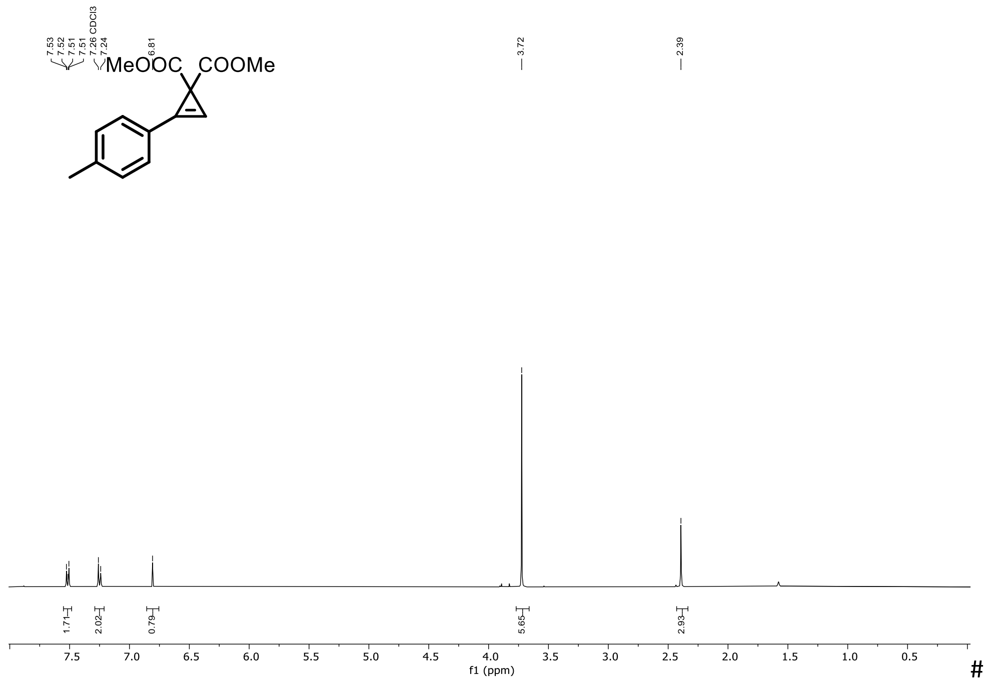

#

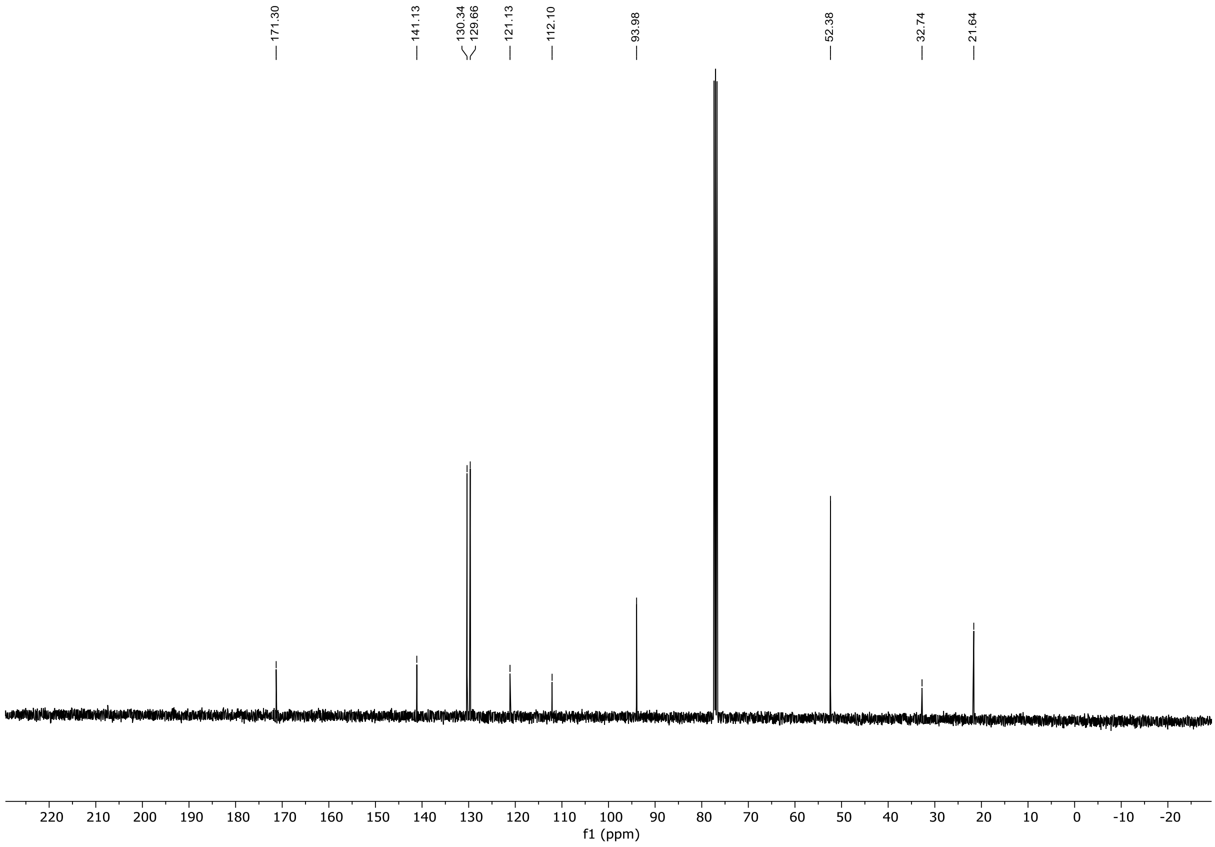

[back to Table of Contents]

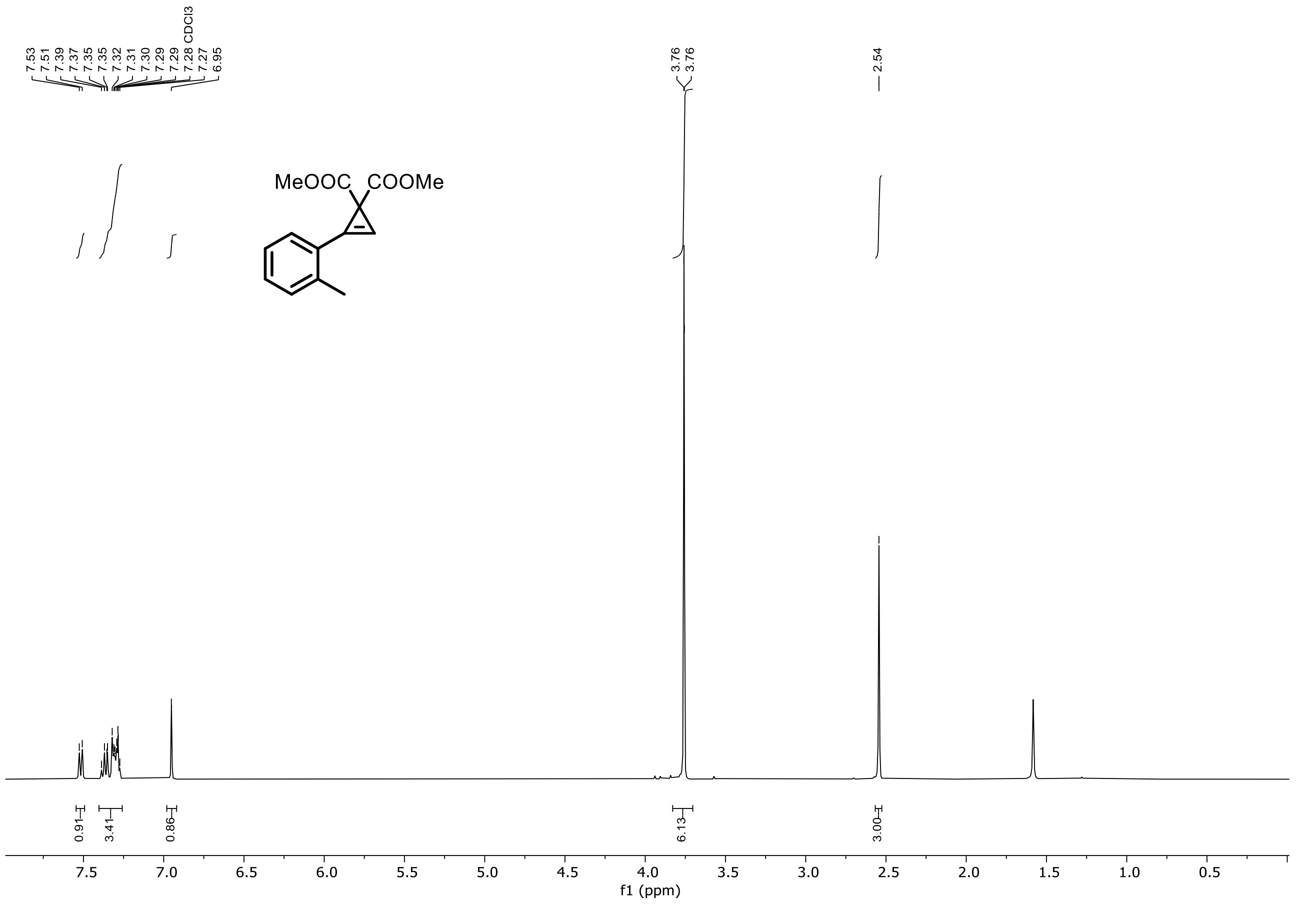

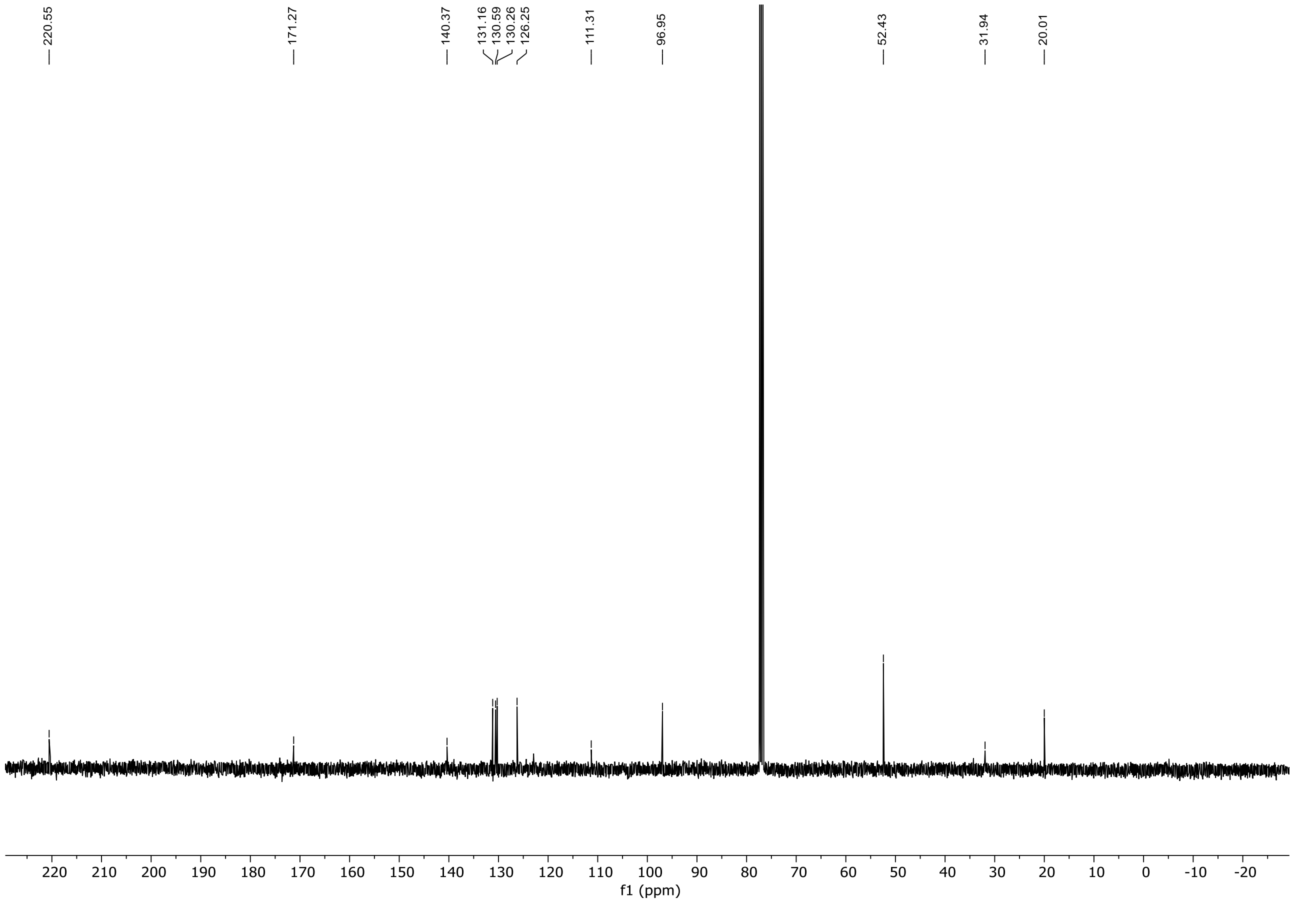

[back to Table of Contents]

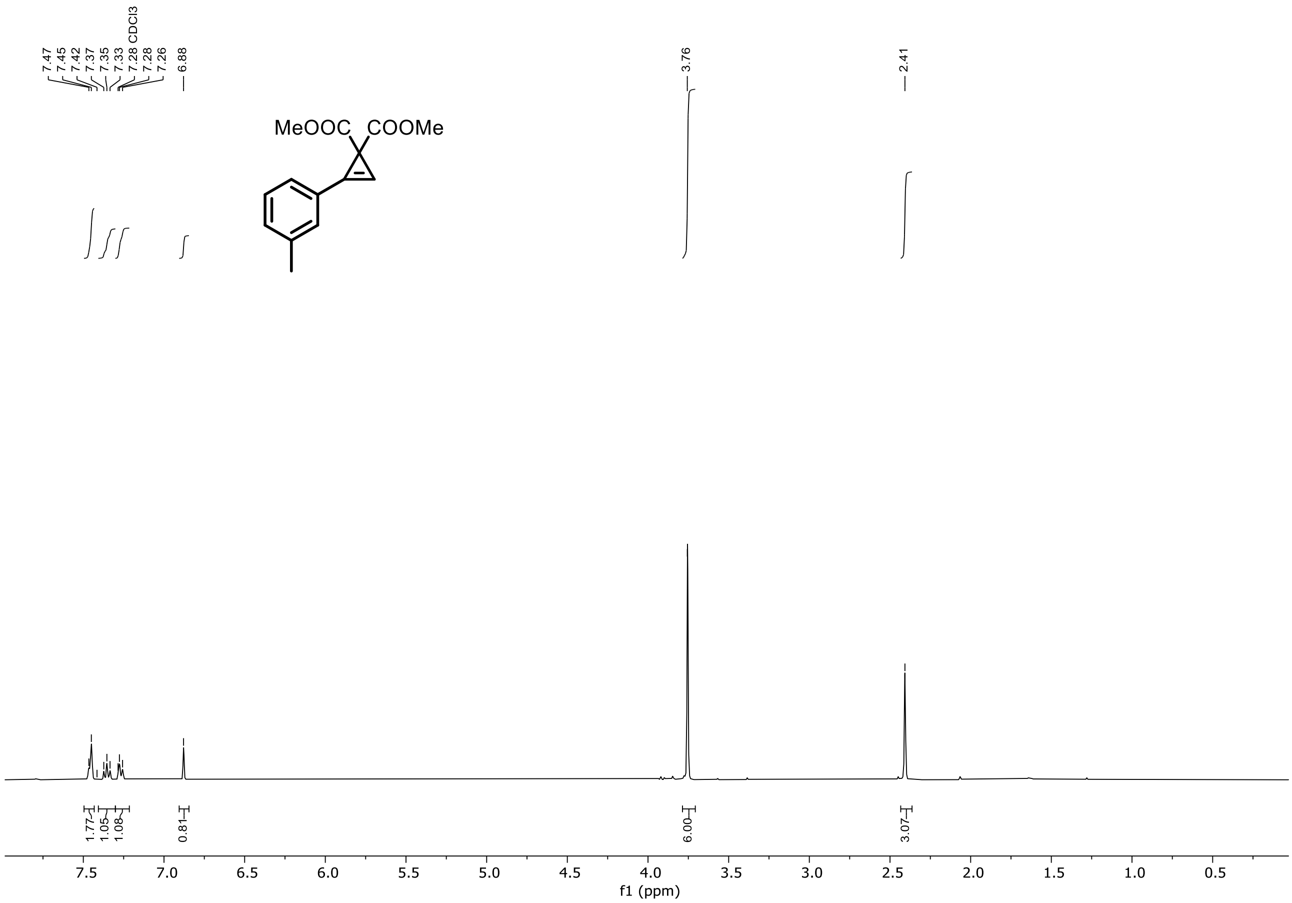

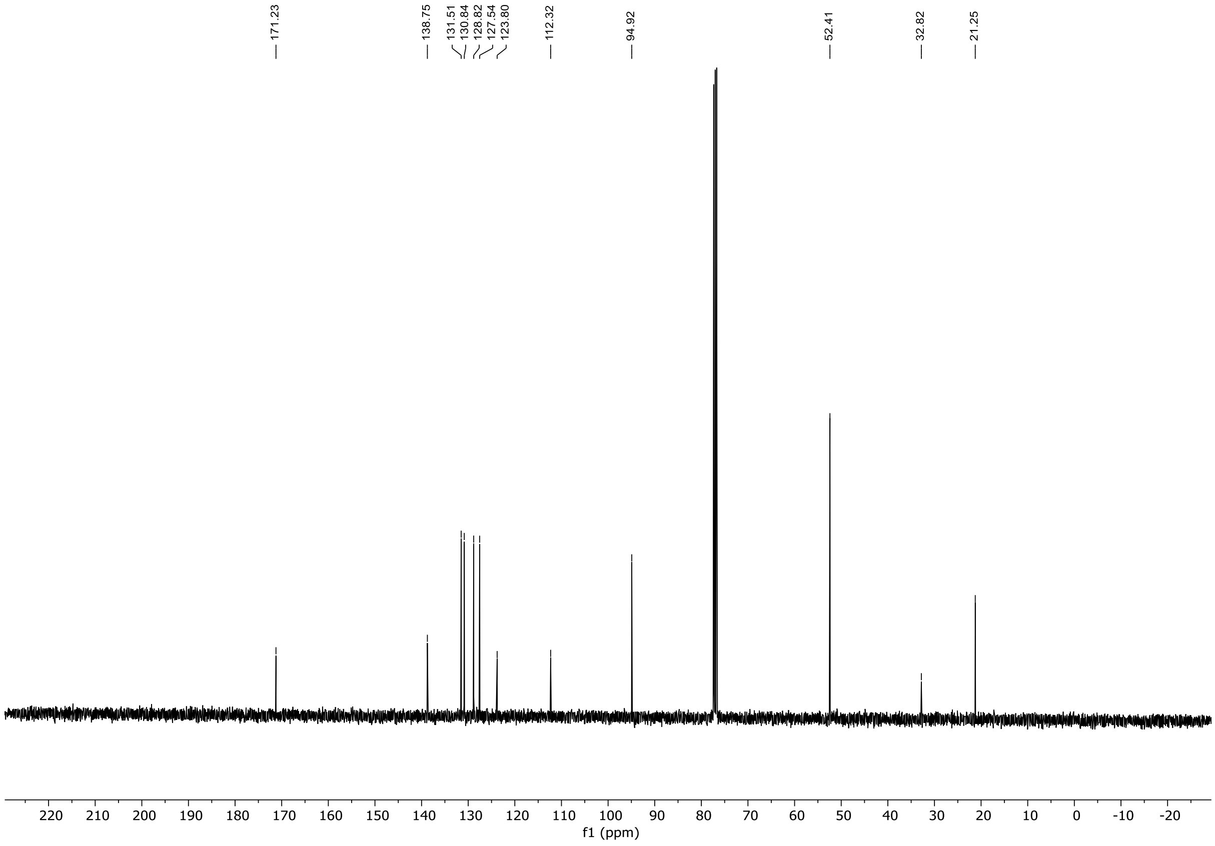

[back to Table of Contents]

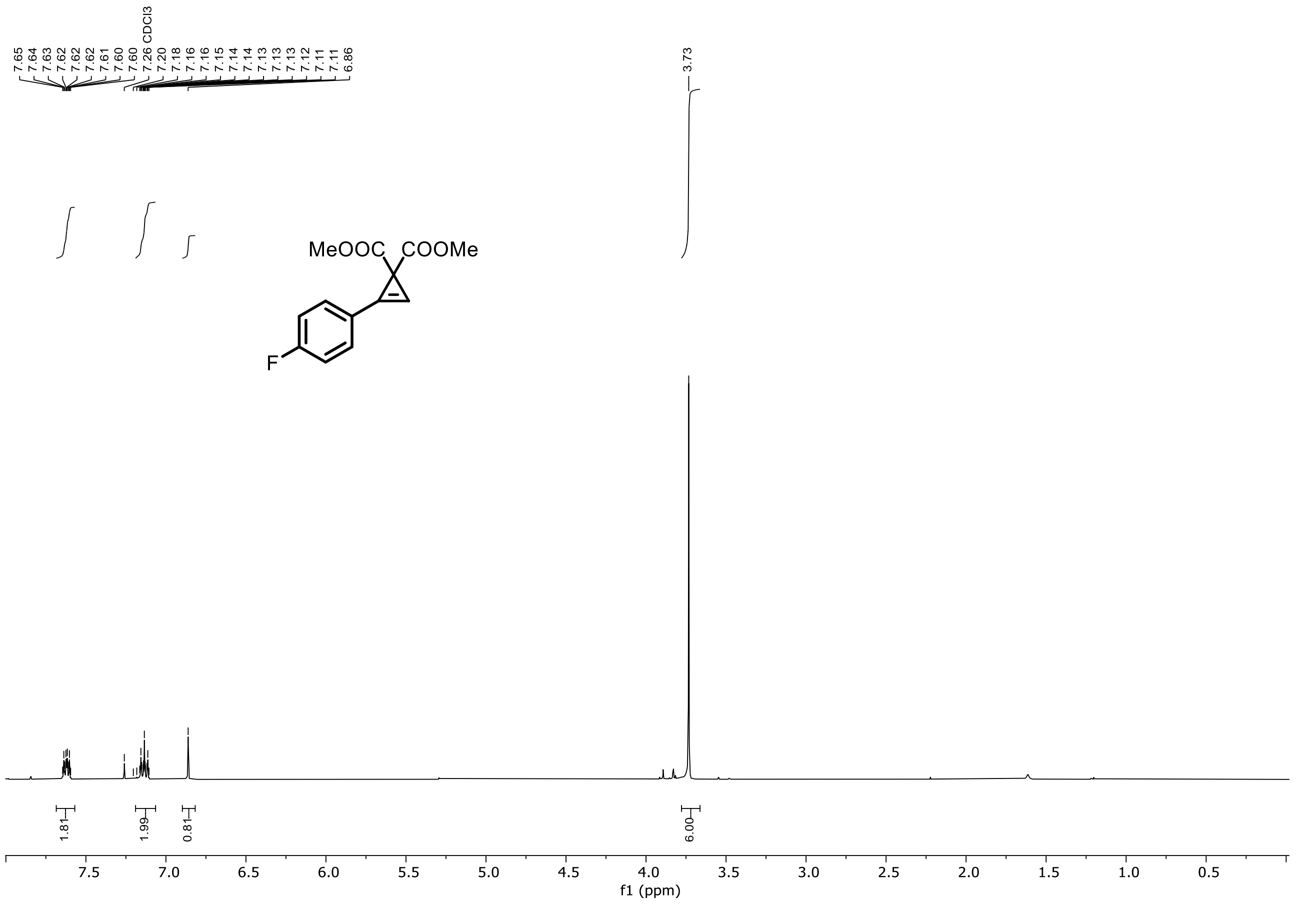

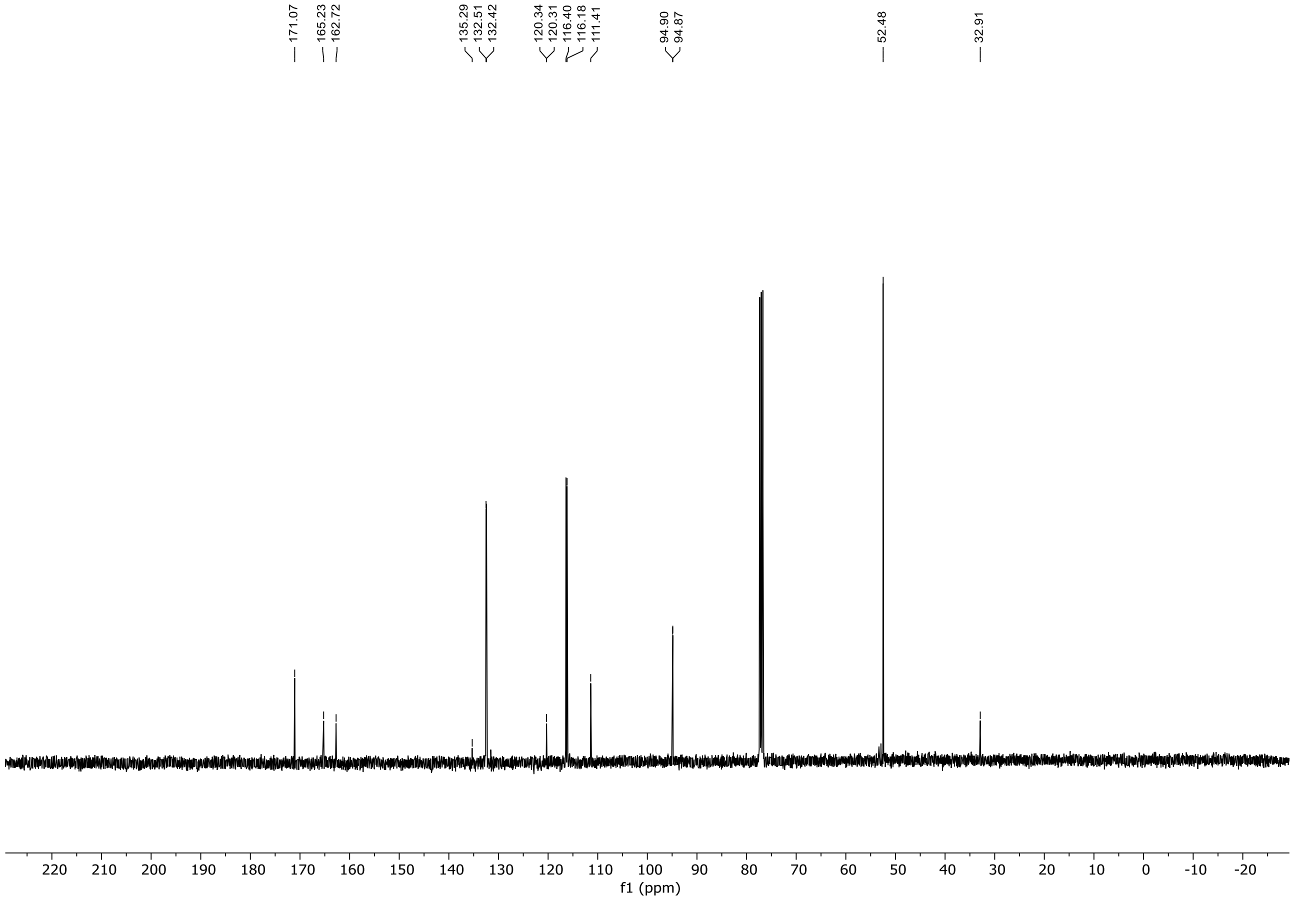

[back to Table of Contents]

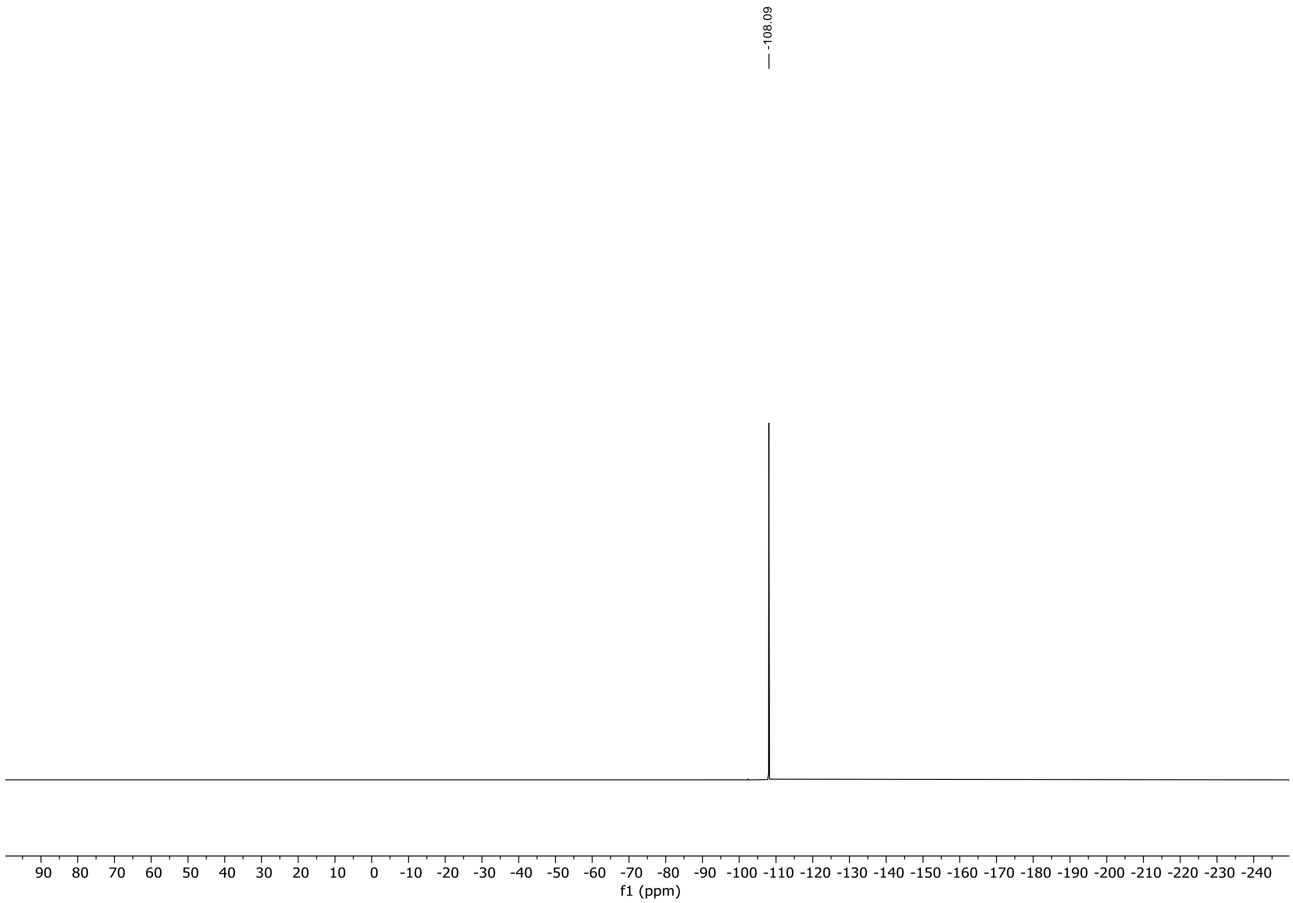

[back to Table of Contents]

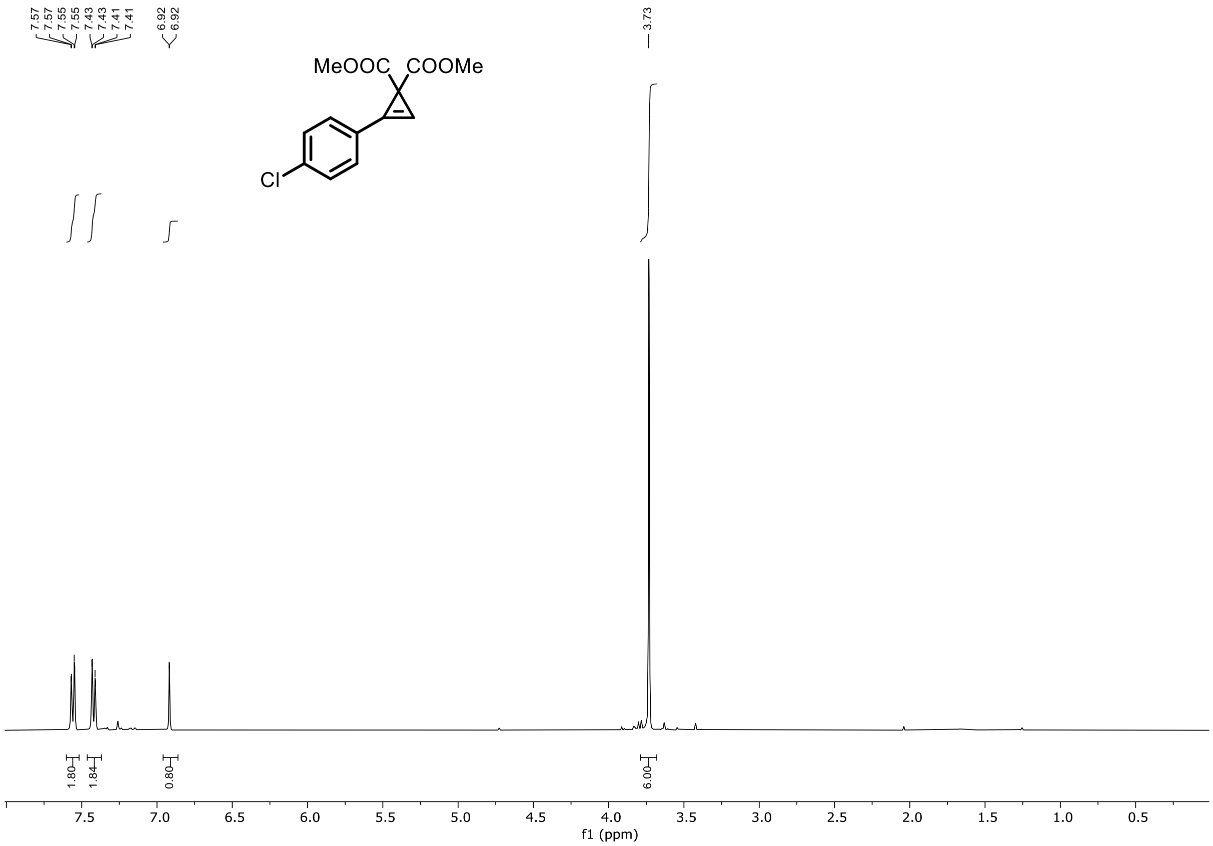

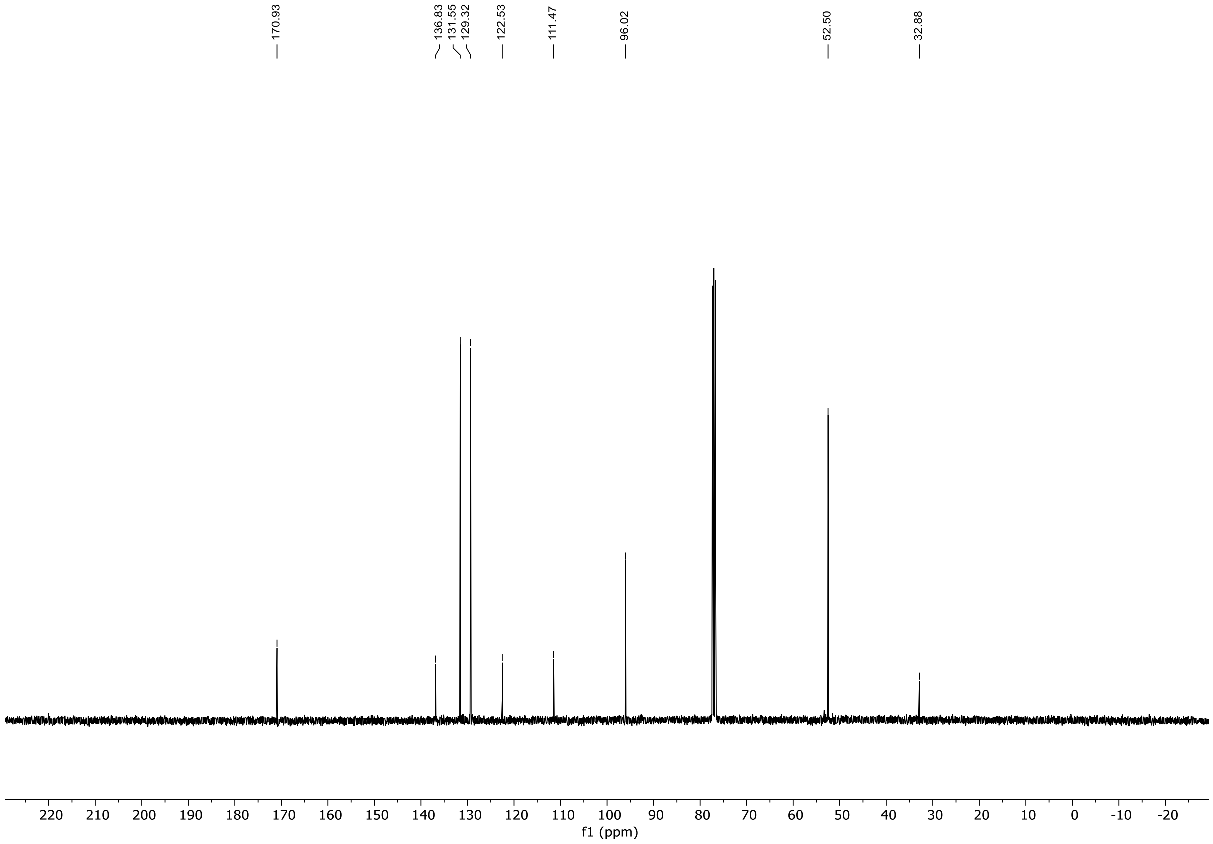

[back to Table of Contents]

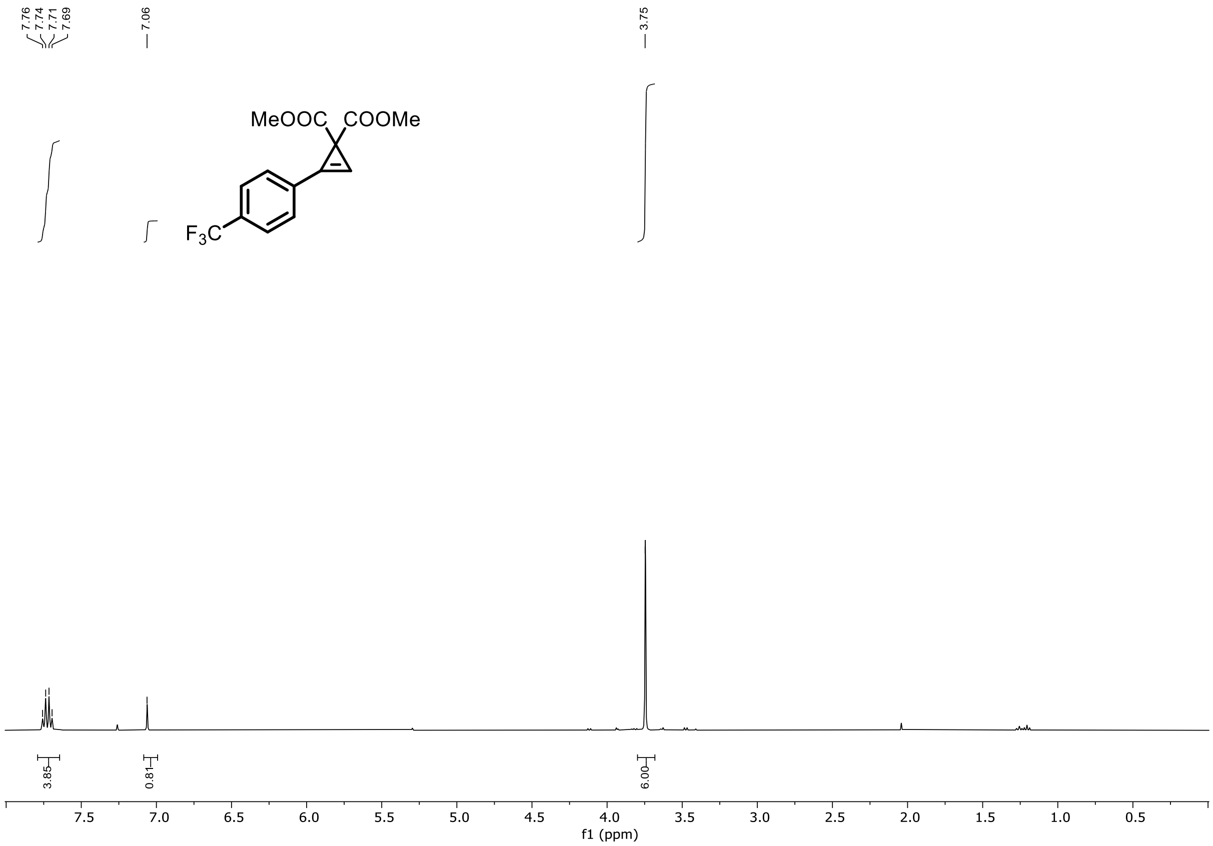

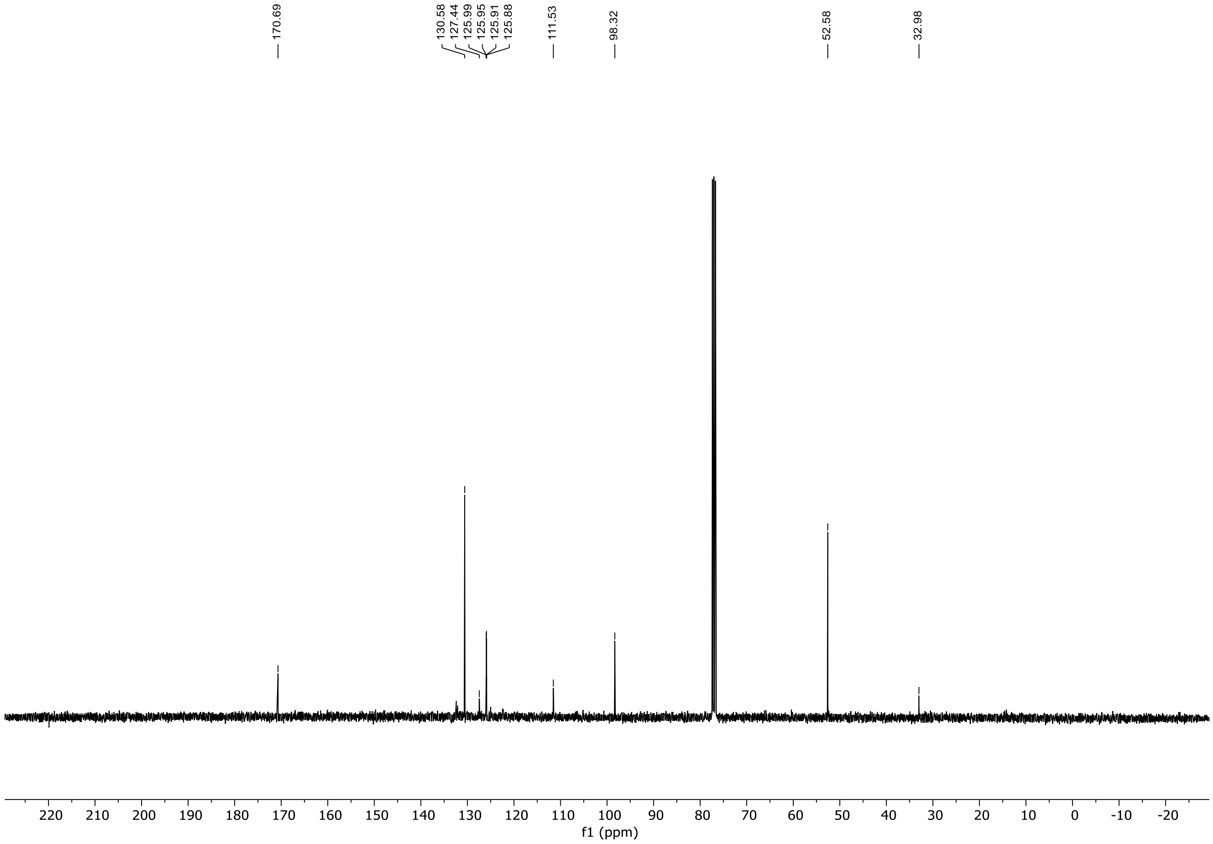

[back to Table of Contents]

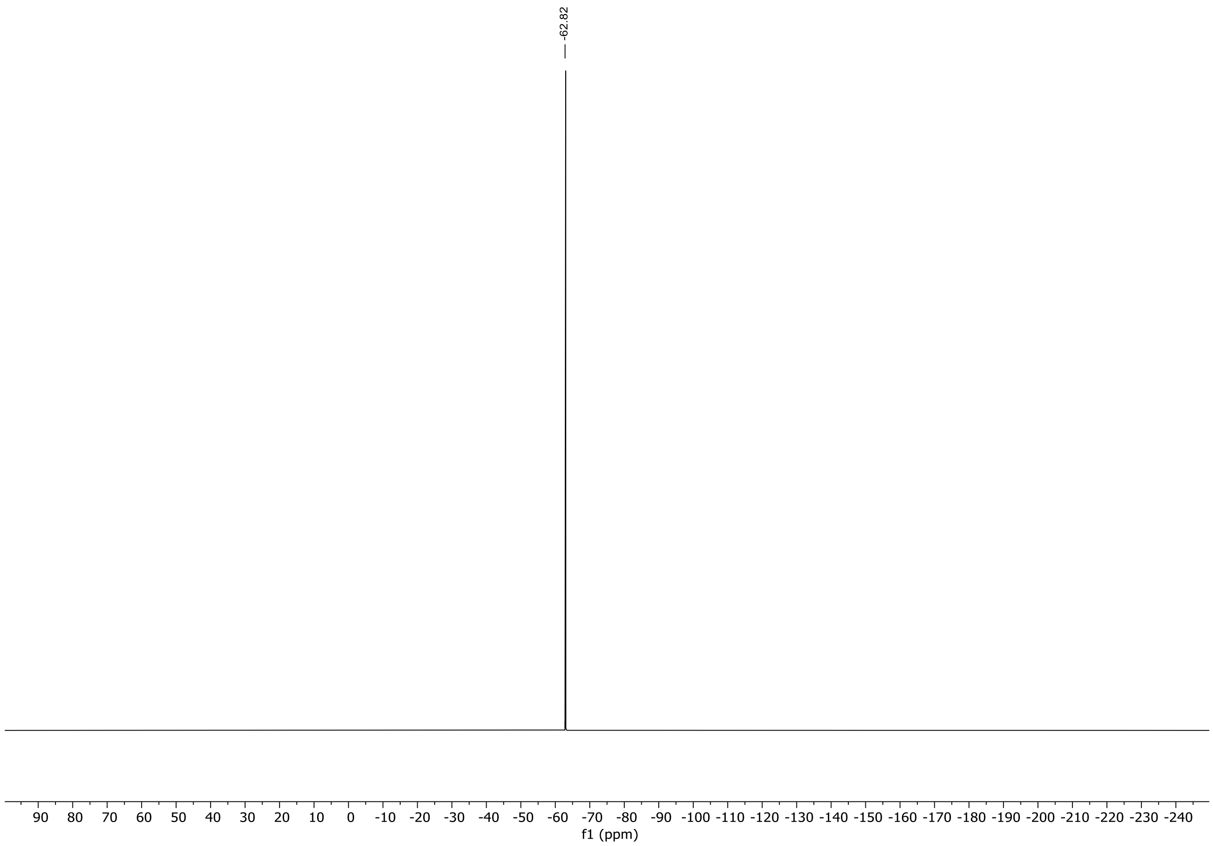

[back to Table of Contents]

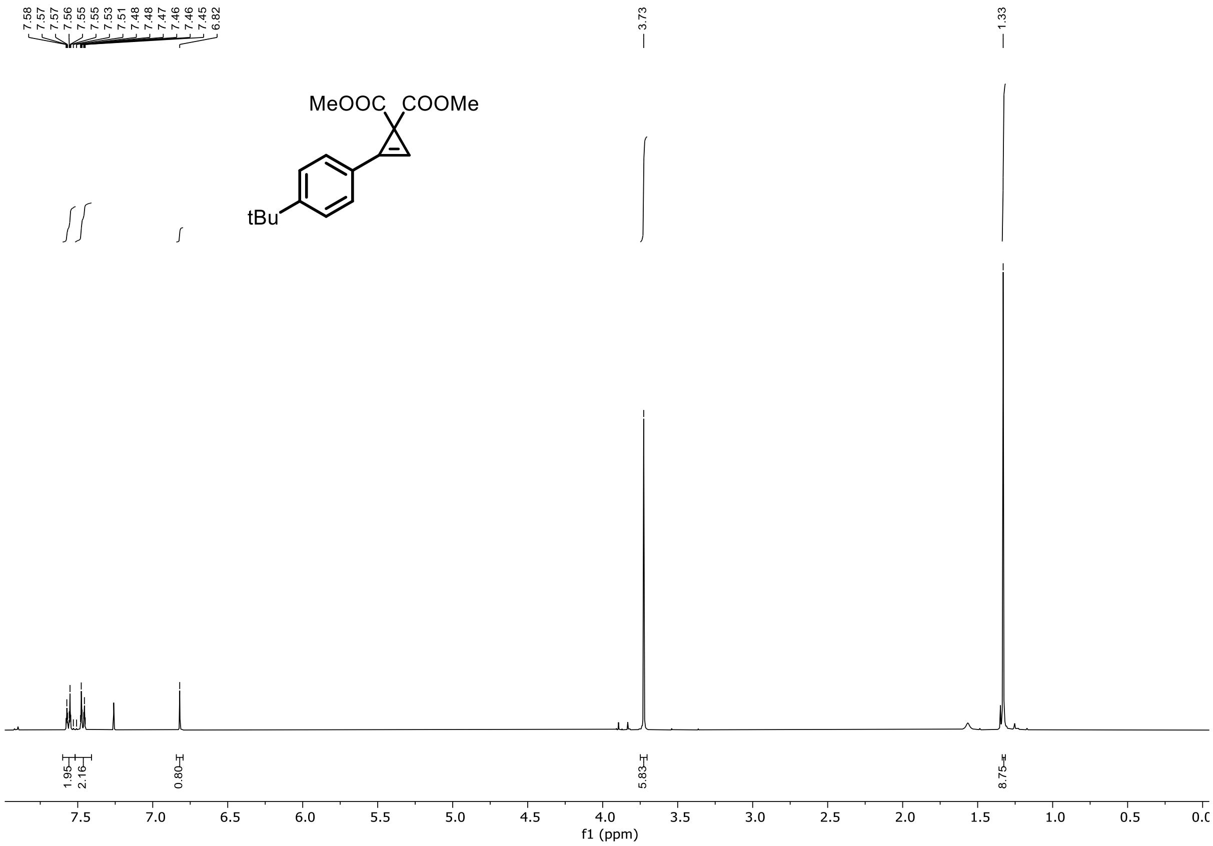

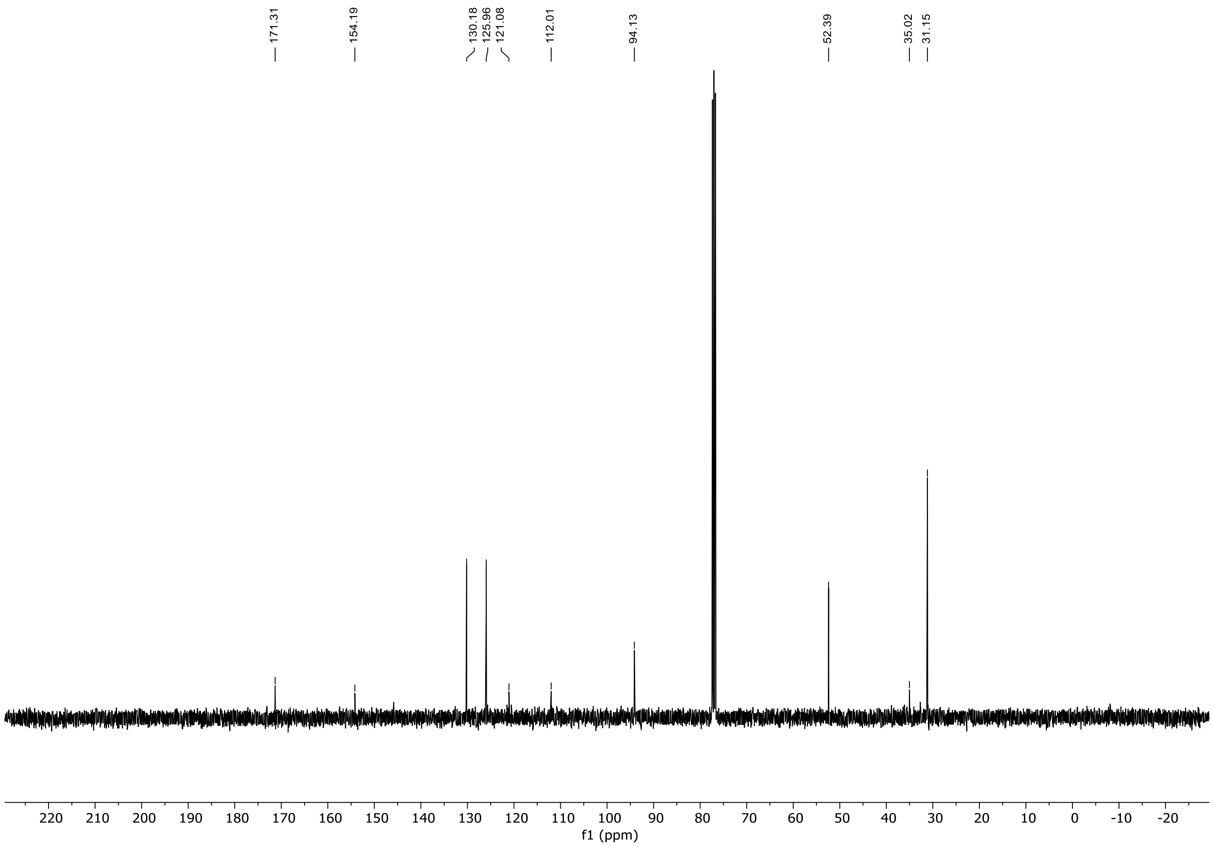

[back to Table of Contents]

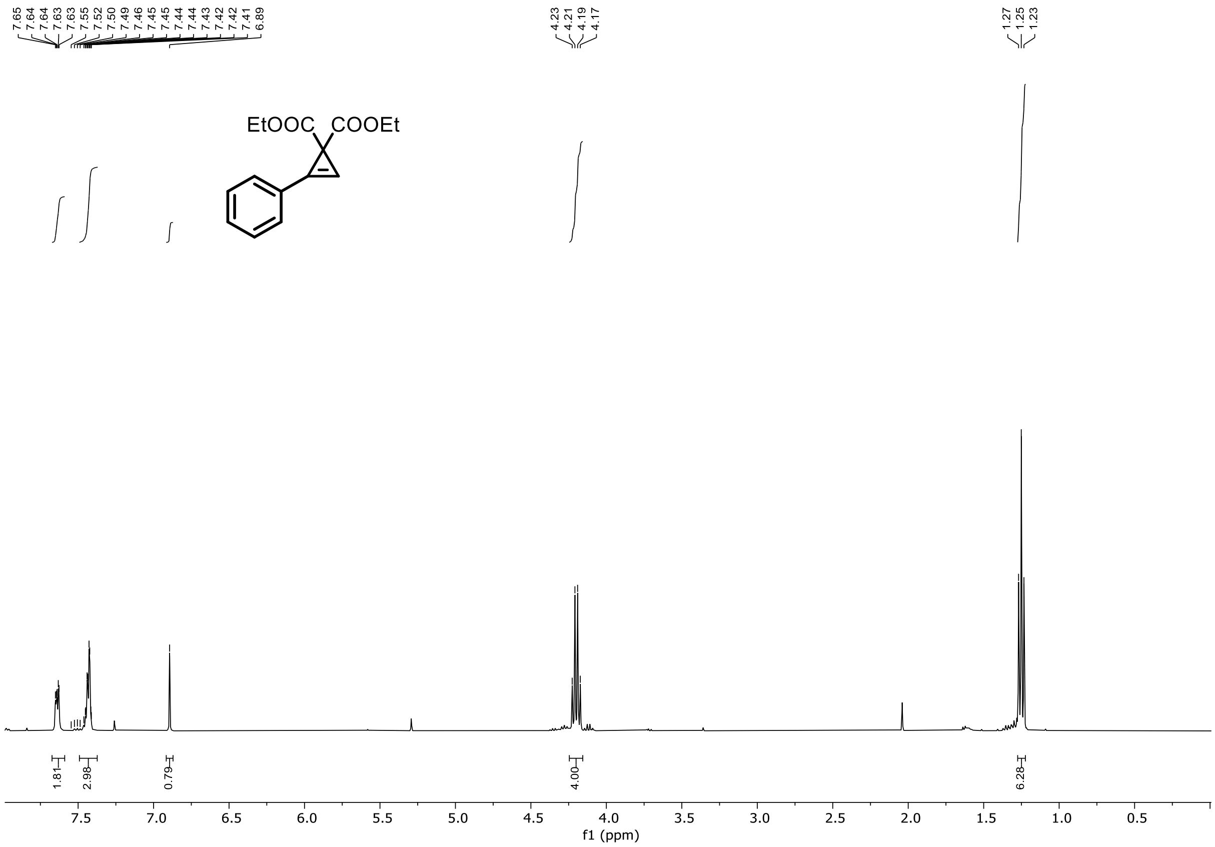

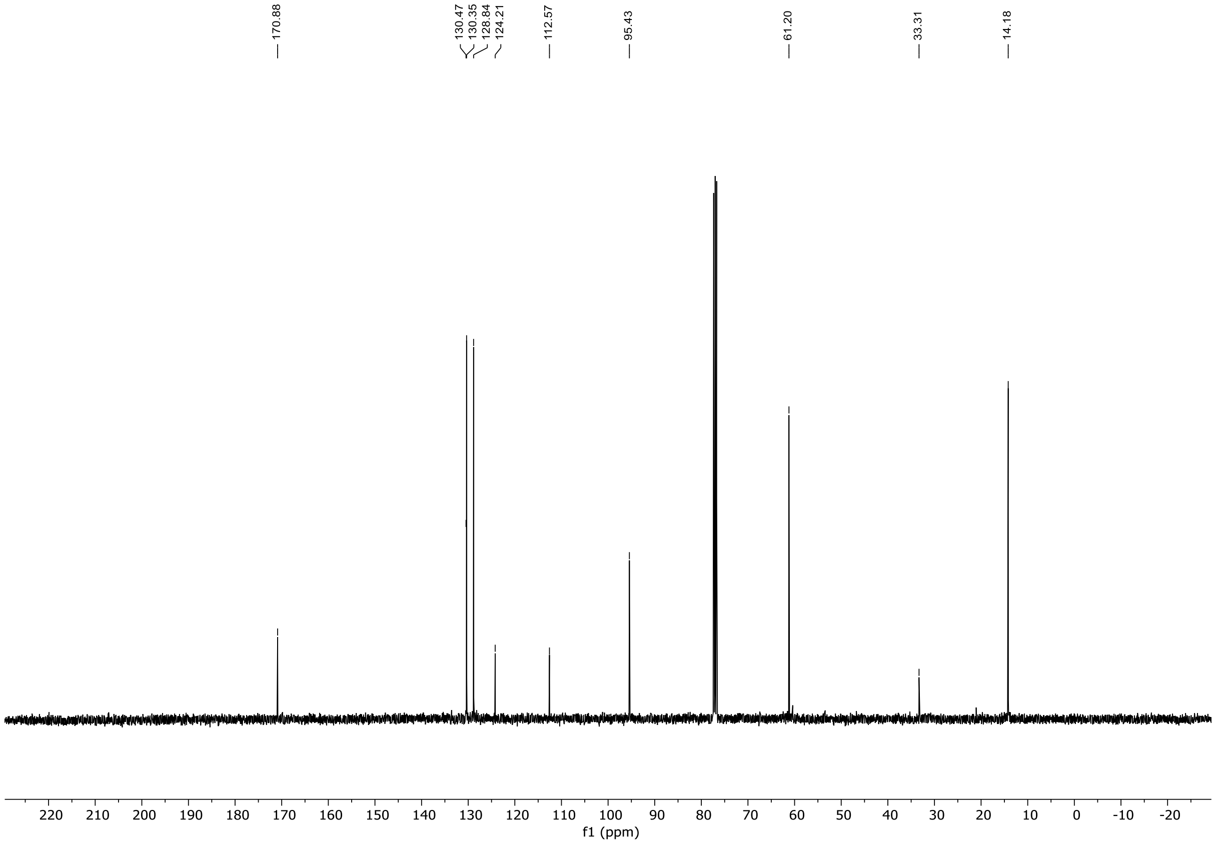

[back to Table of Contents]

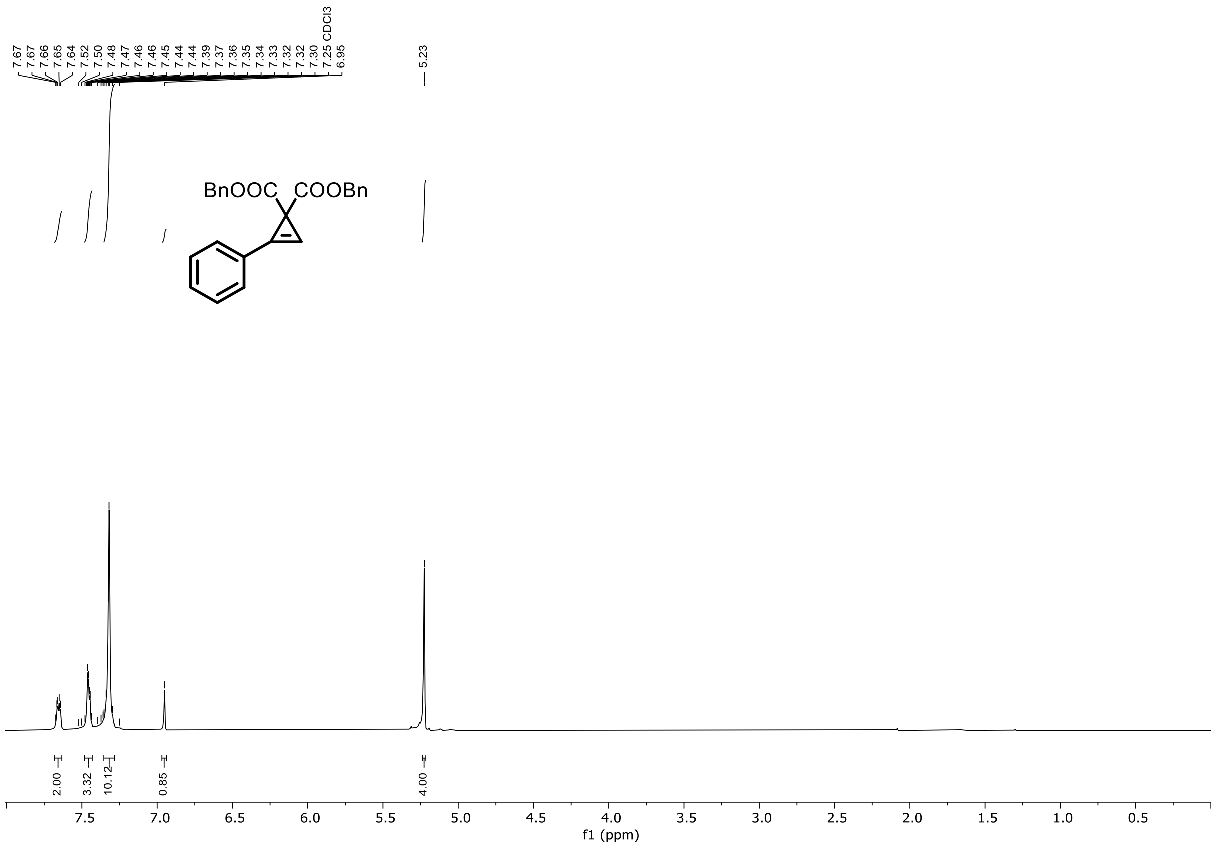

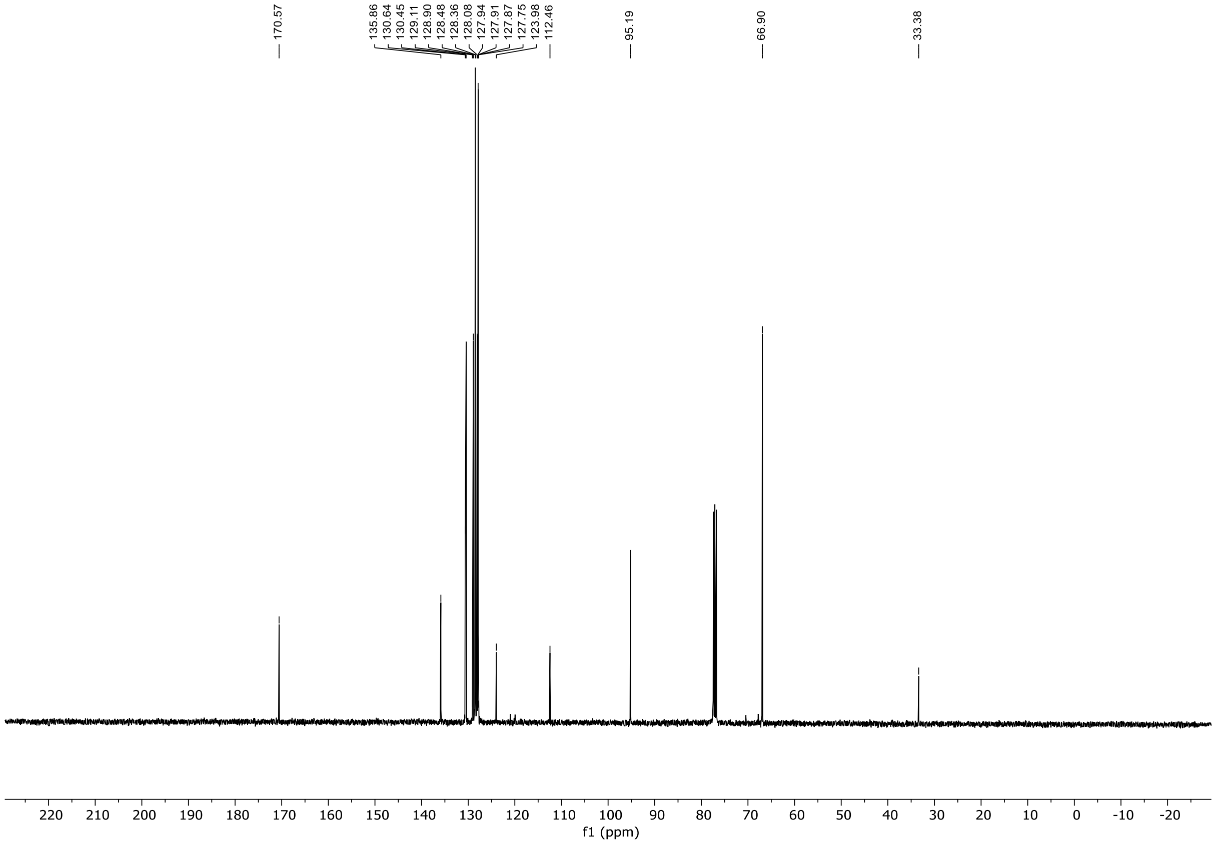

[back to Table of Contents]

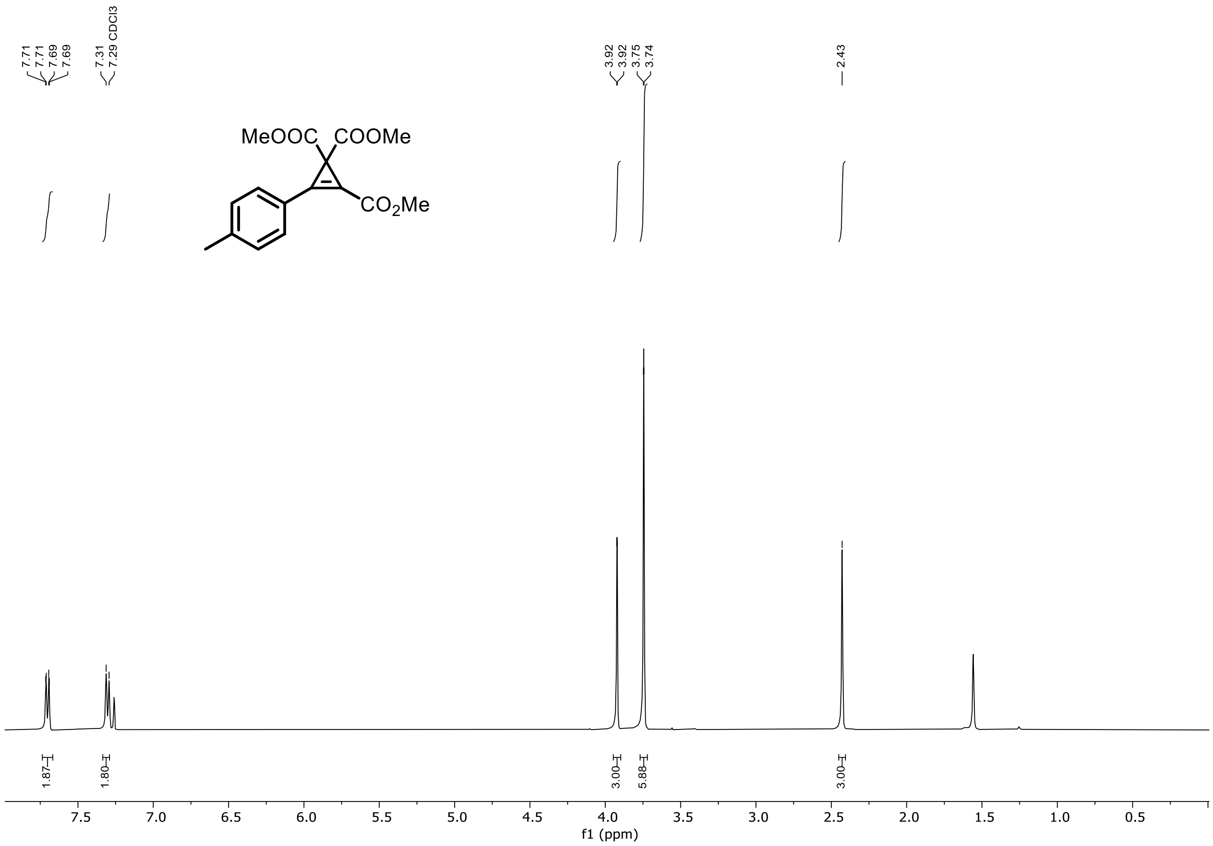

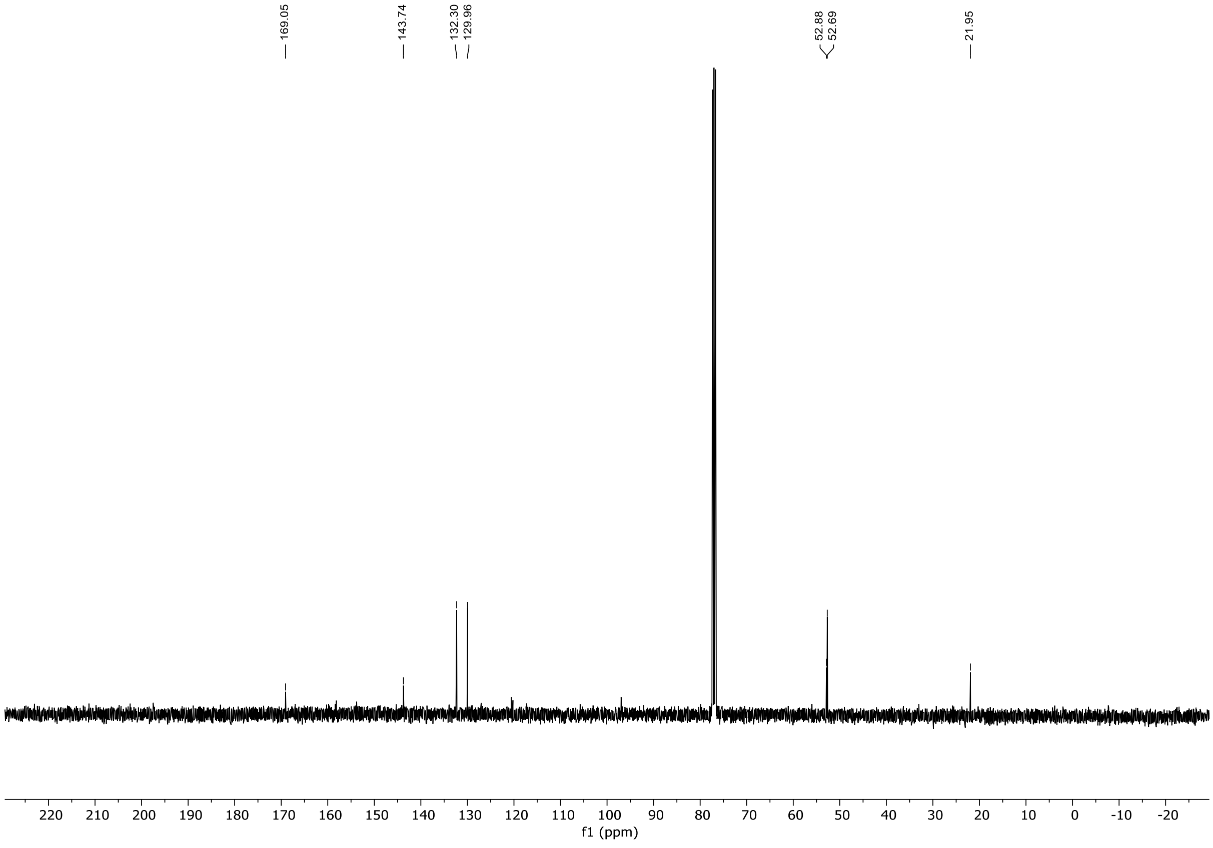

[back to Table of Contents]

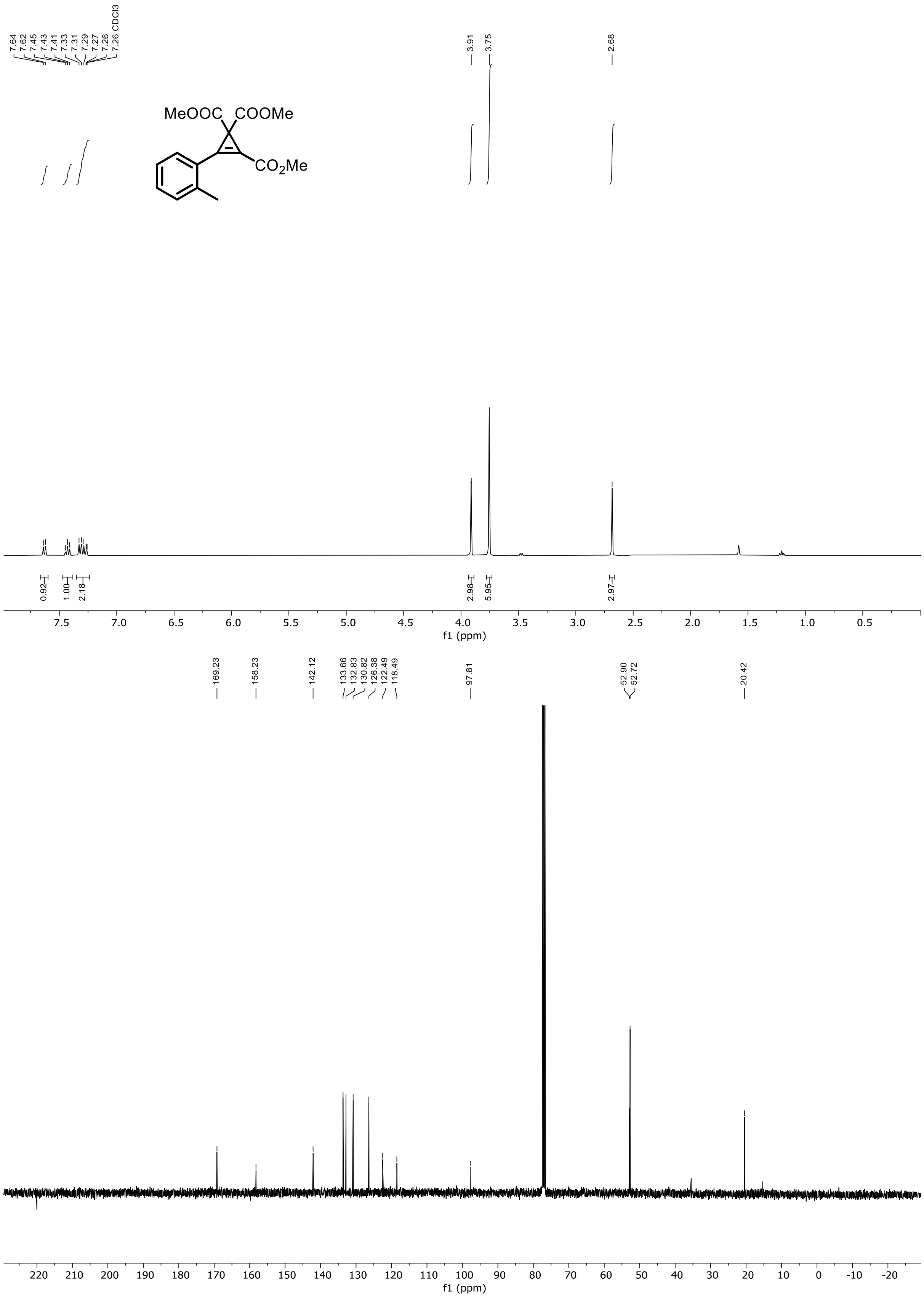

[back to Table of Contents]

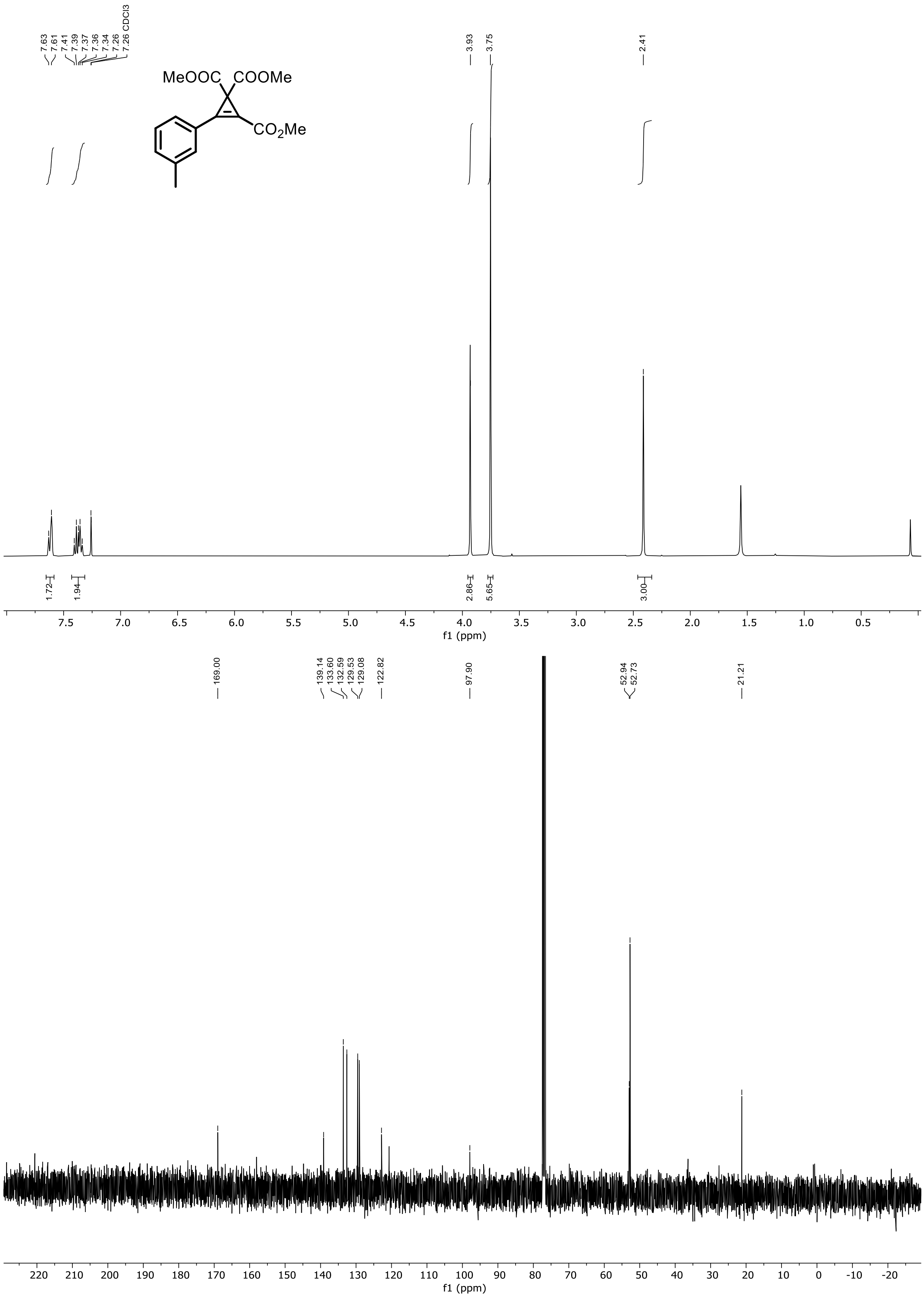

[back to Table of Contents]

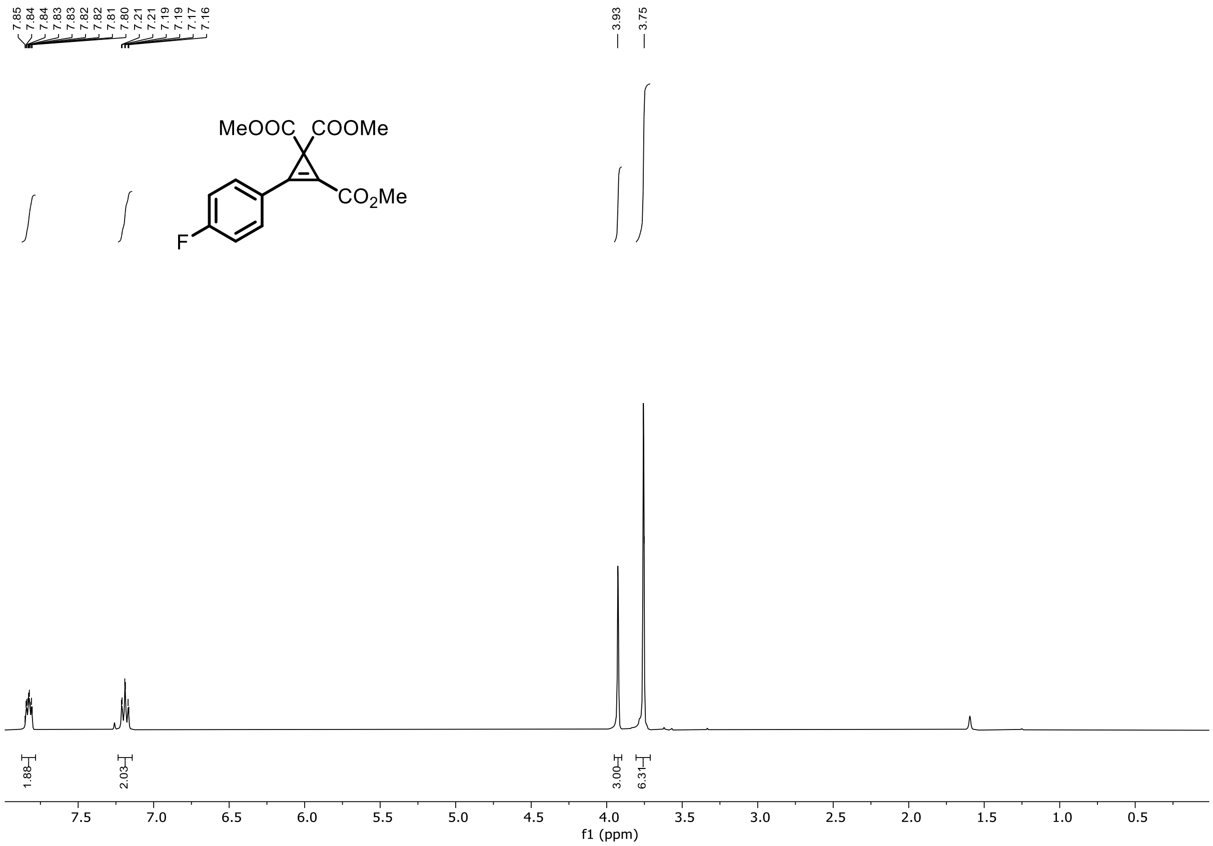

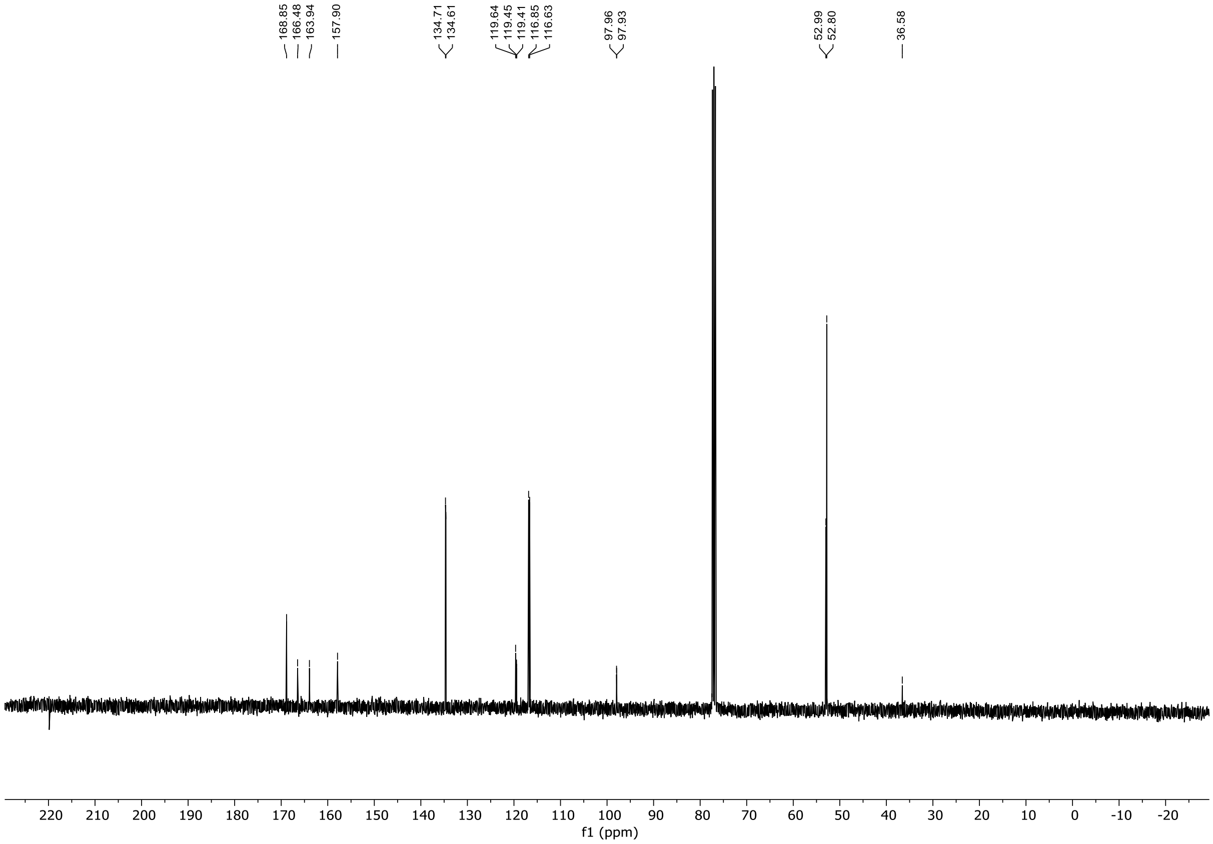

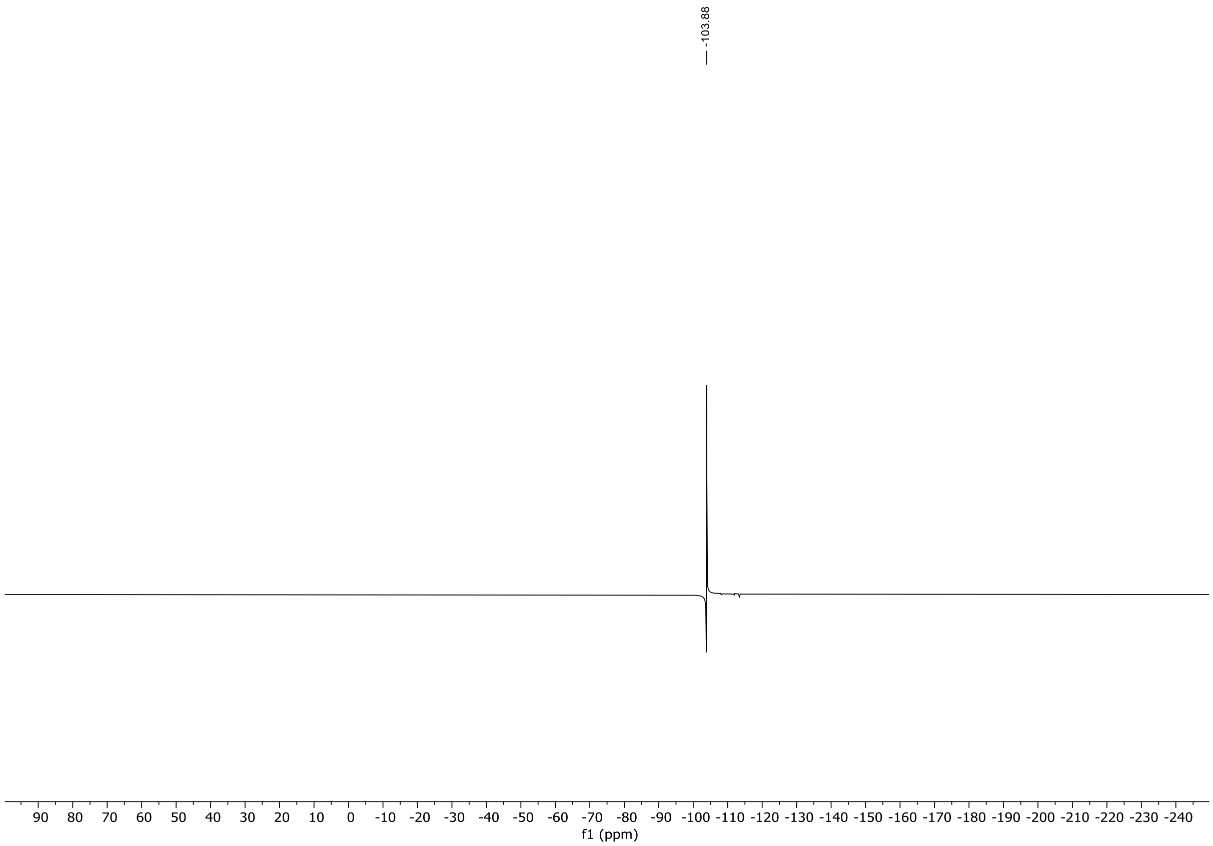
-103.88
90 80 70 60 50 40 30 20 10 0 -10 -20 -30 -40 -50 -60 -70 -80 -90 -100 -110 -120 -130 -140 -150 -160 -170 -180 -190 -200 -210 -220 -230 -240
f1 (ppm)

[back to Table of Contents]

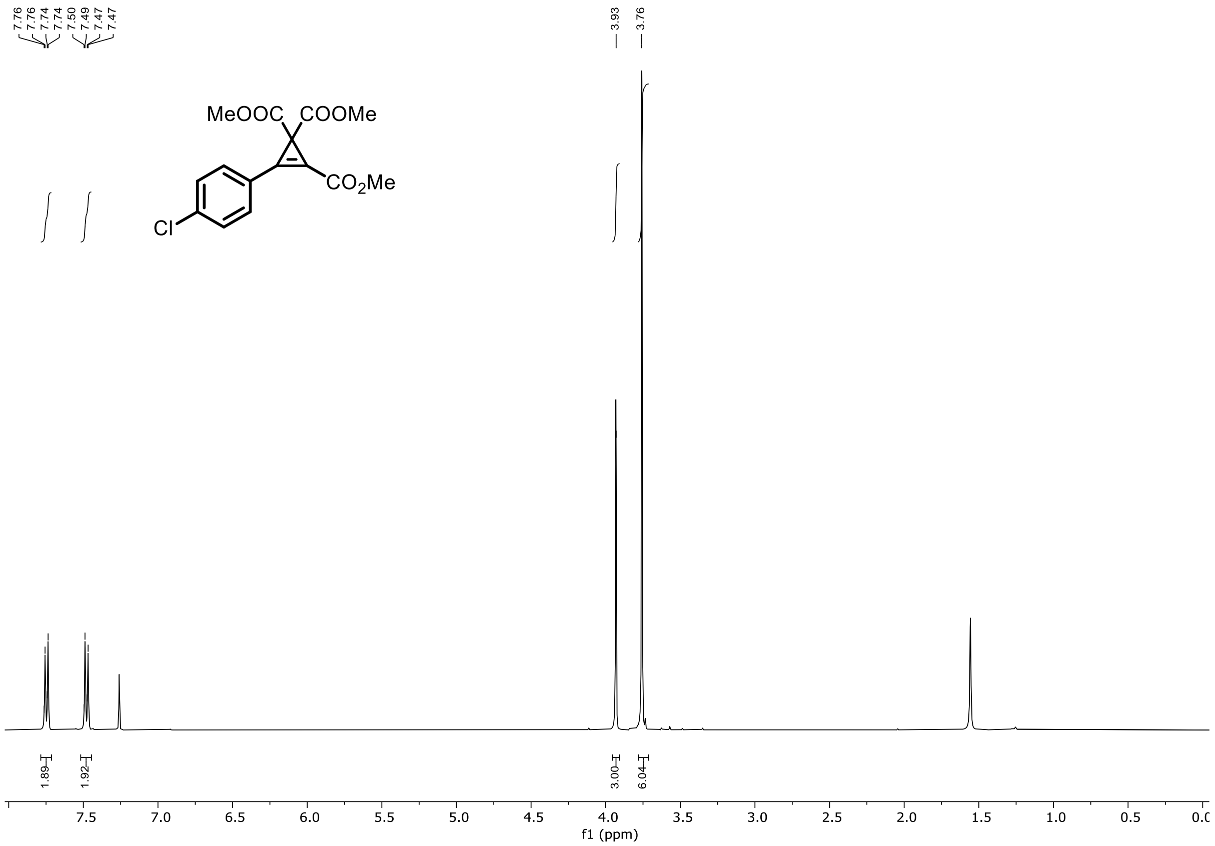

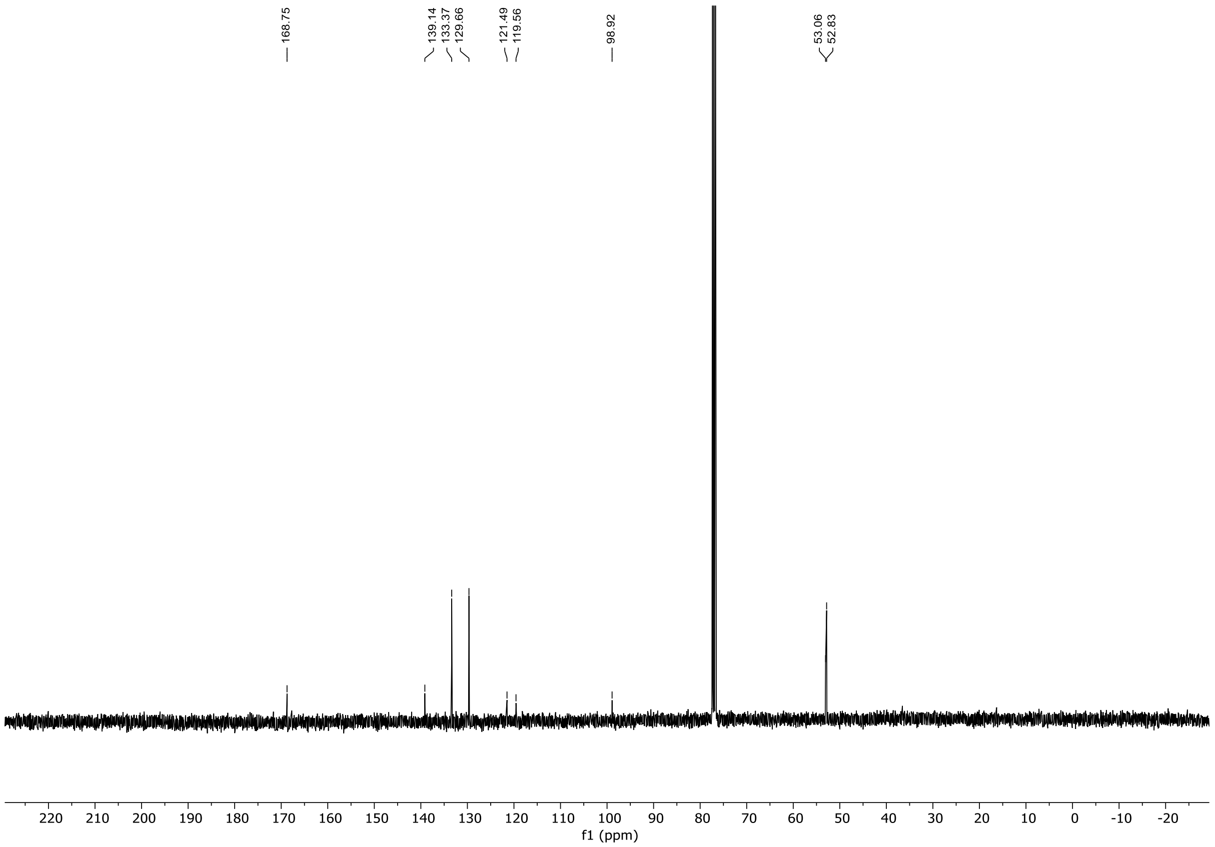

[back to Table of Contents]

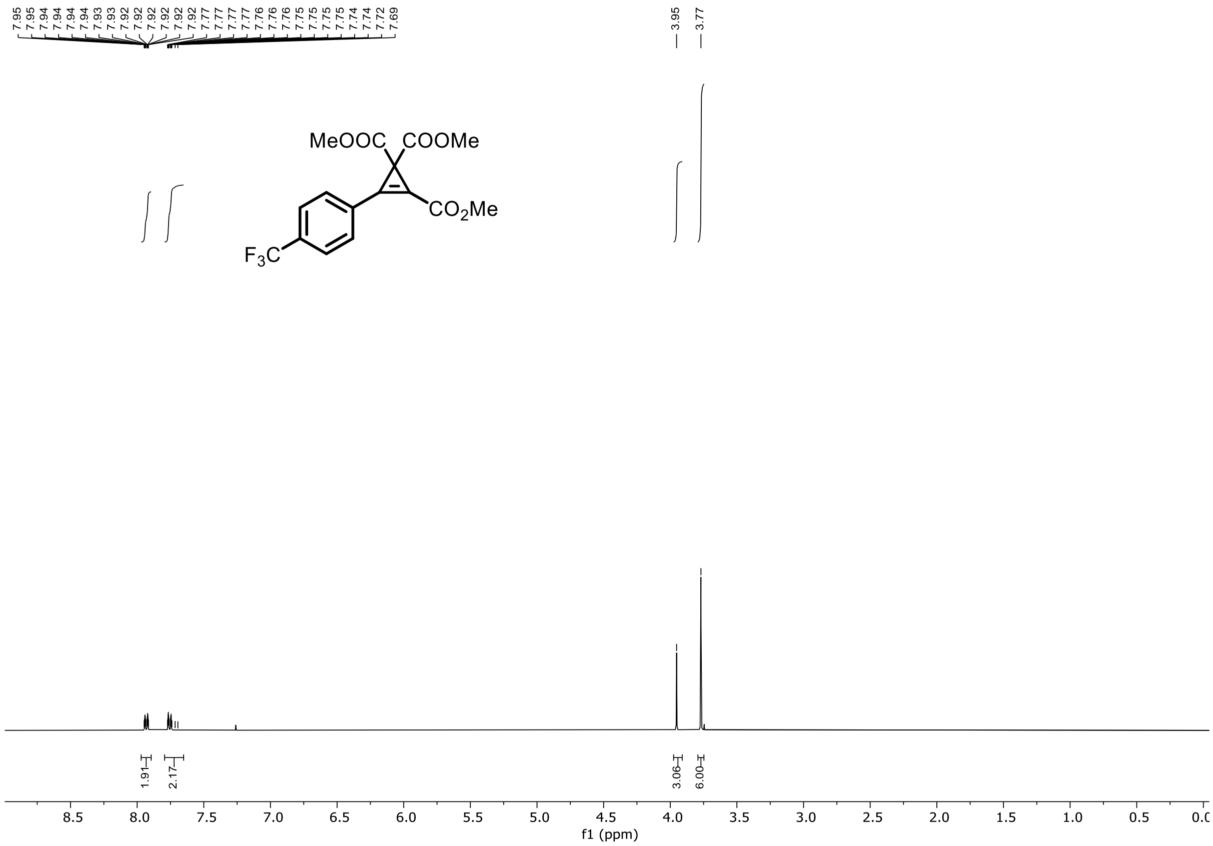

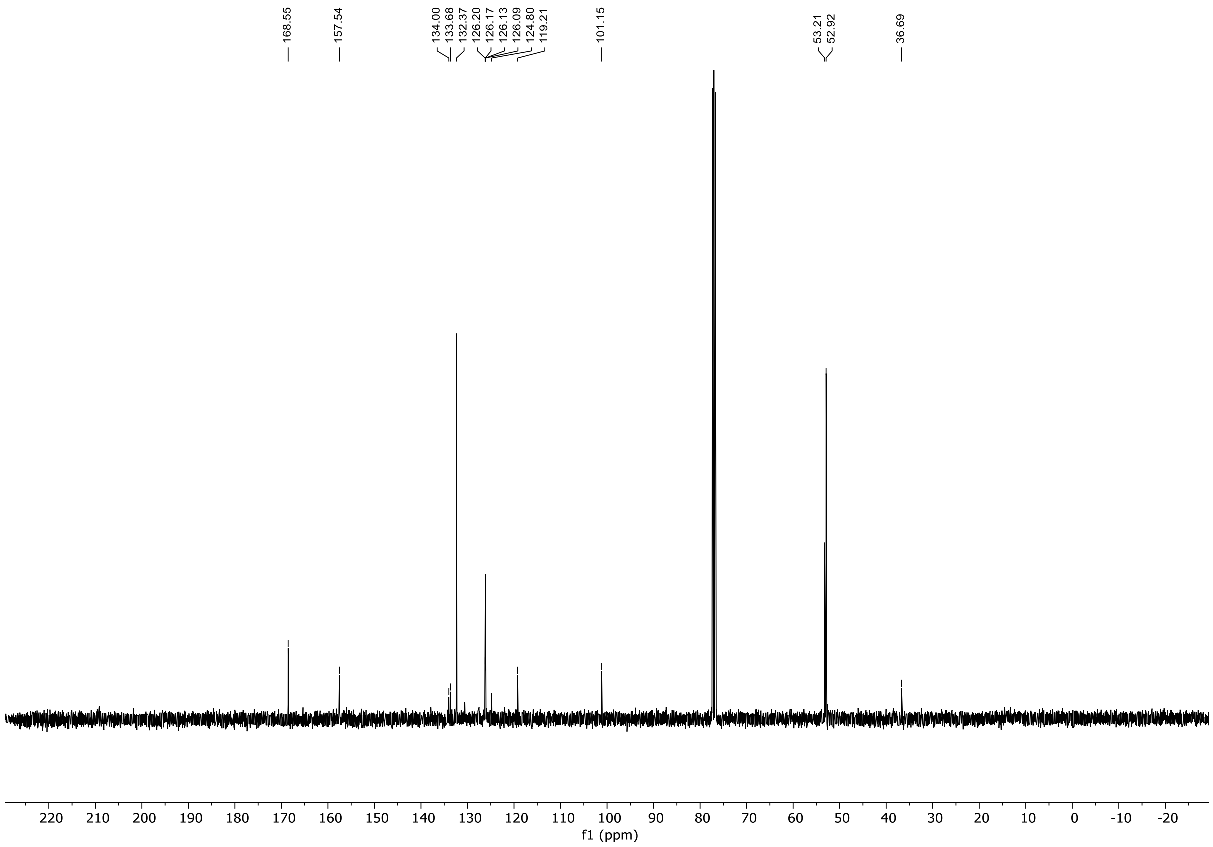

[back to Table of Contents]

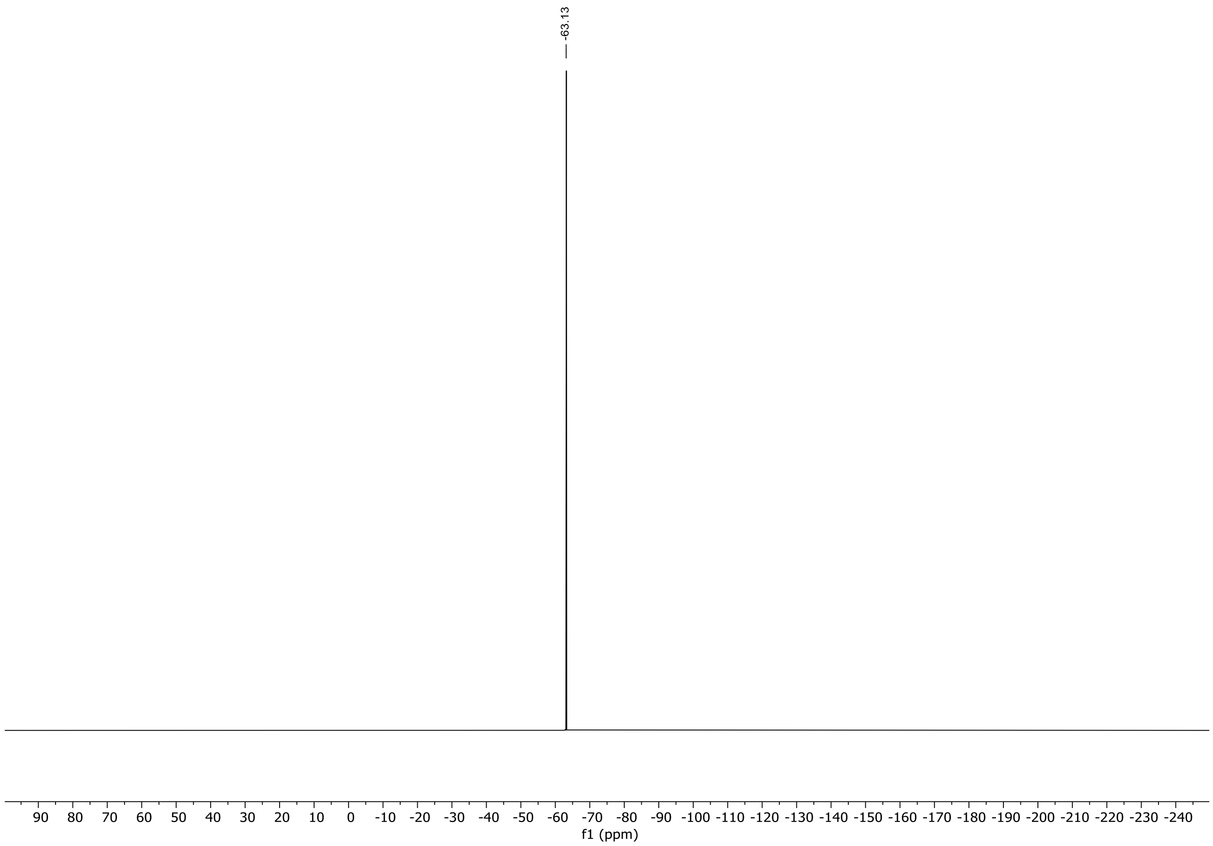

[back to Table of Contents]

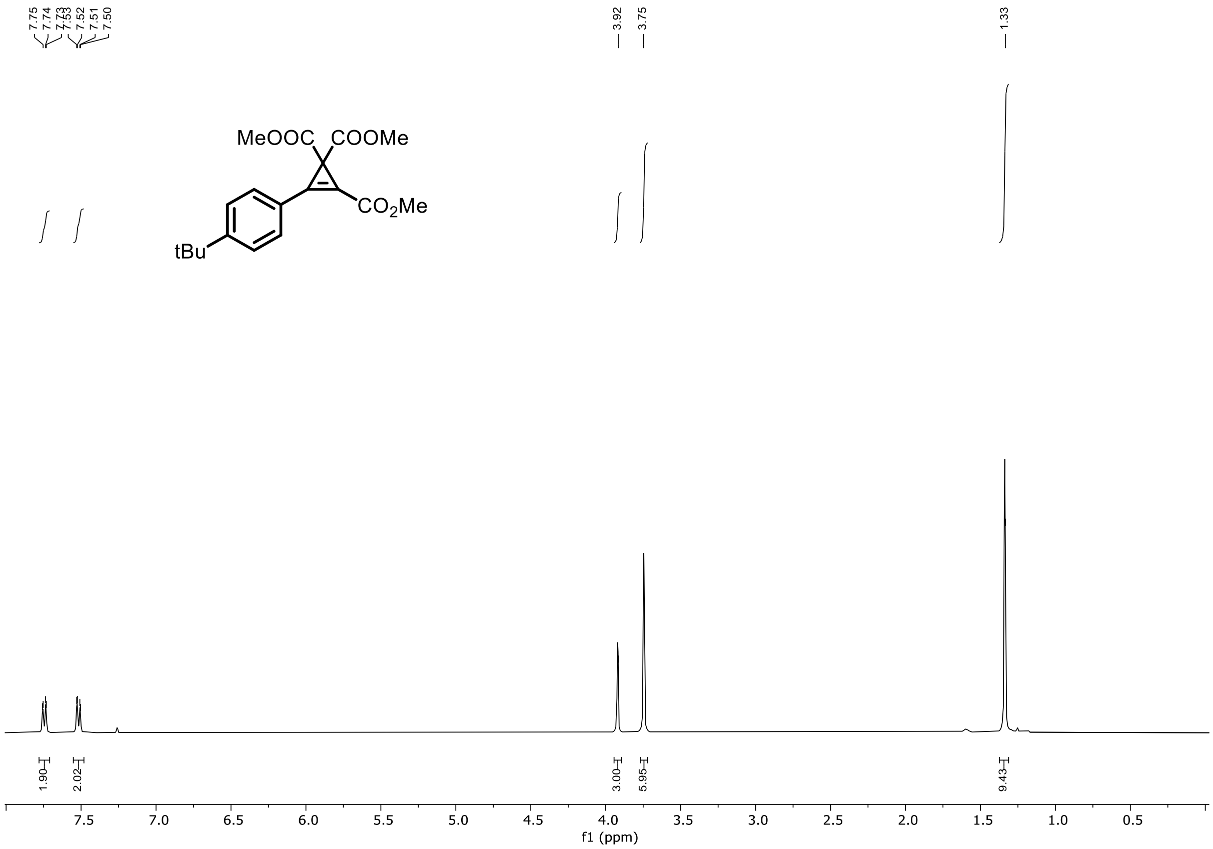

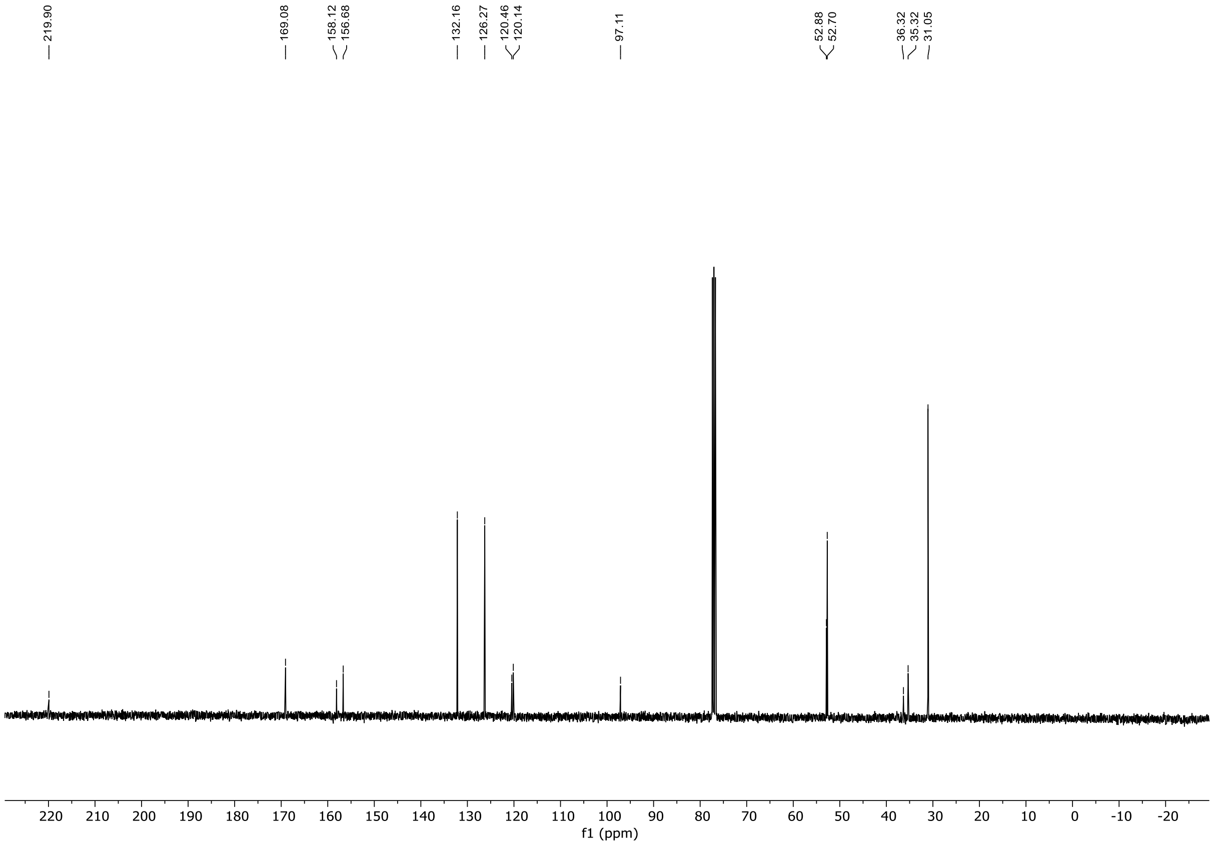

[back to Table of Contents]

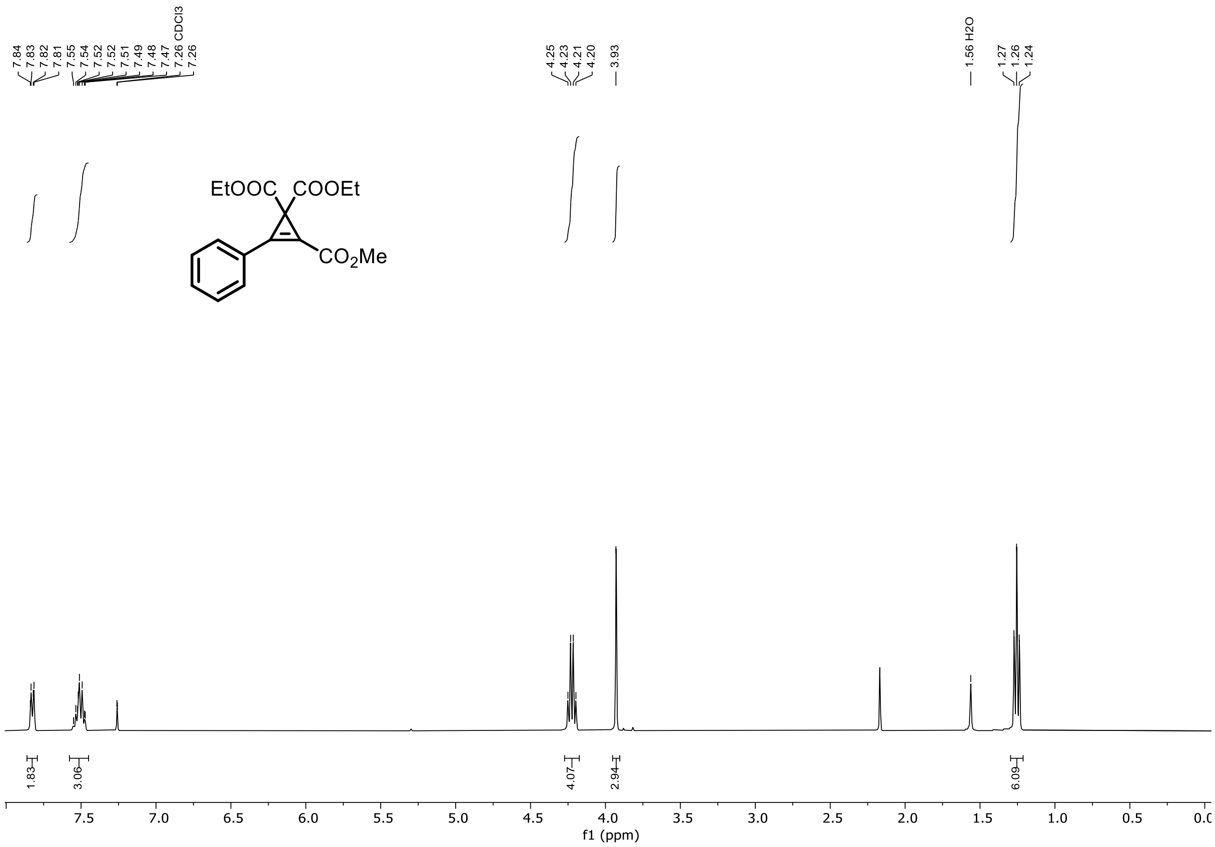

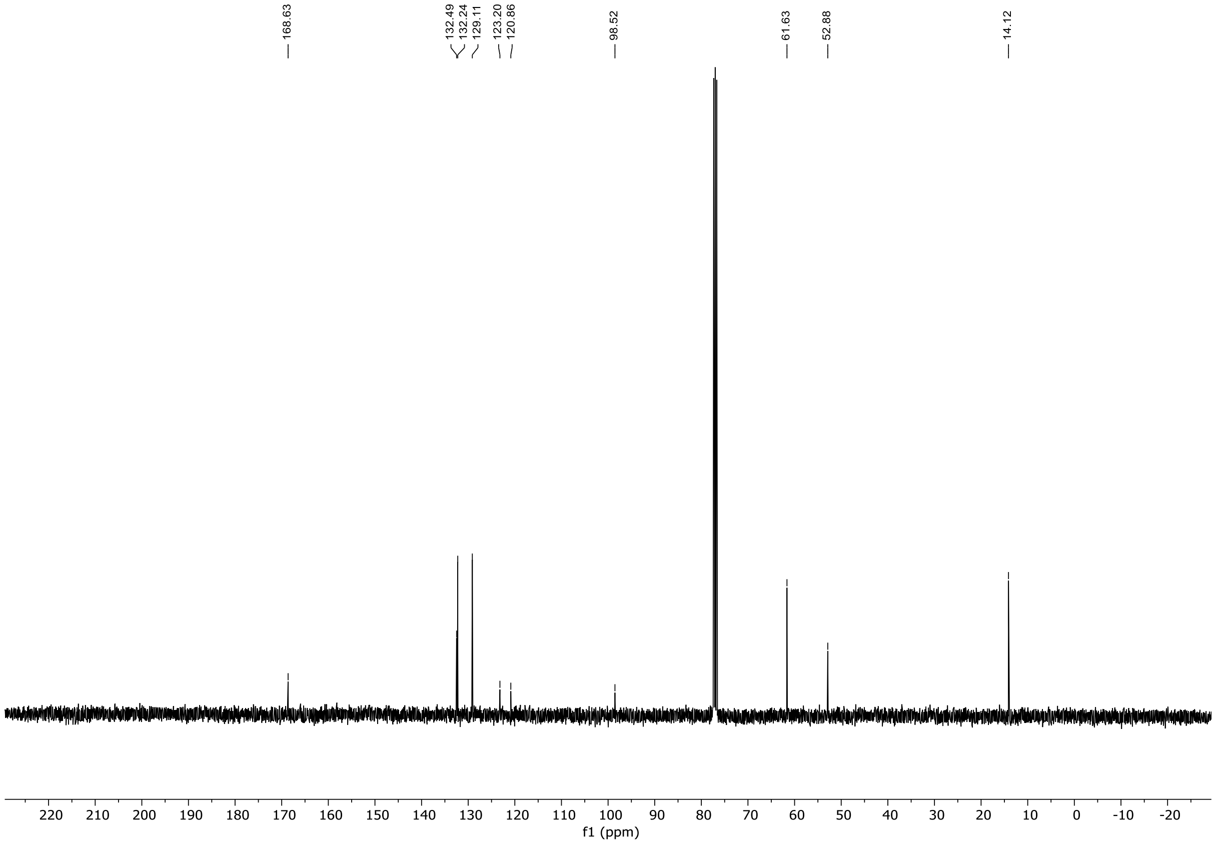

[back to Table of Contents]

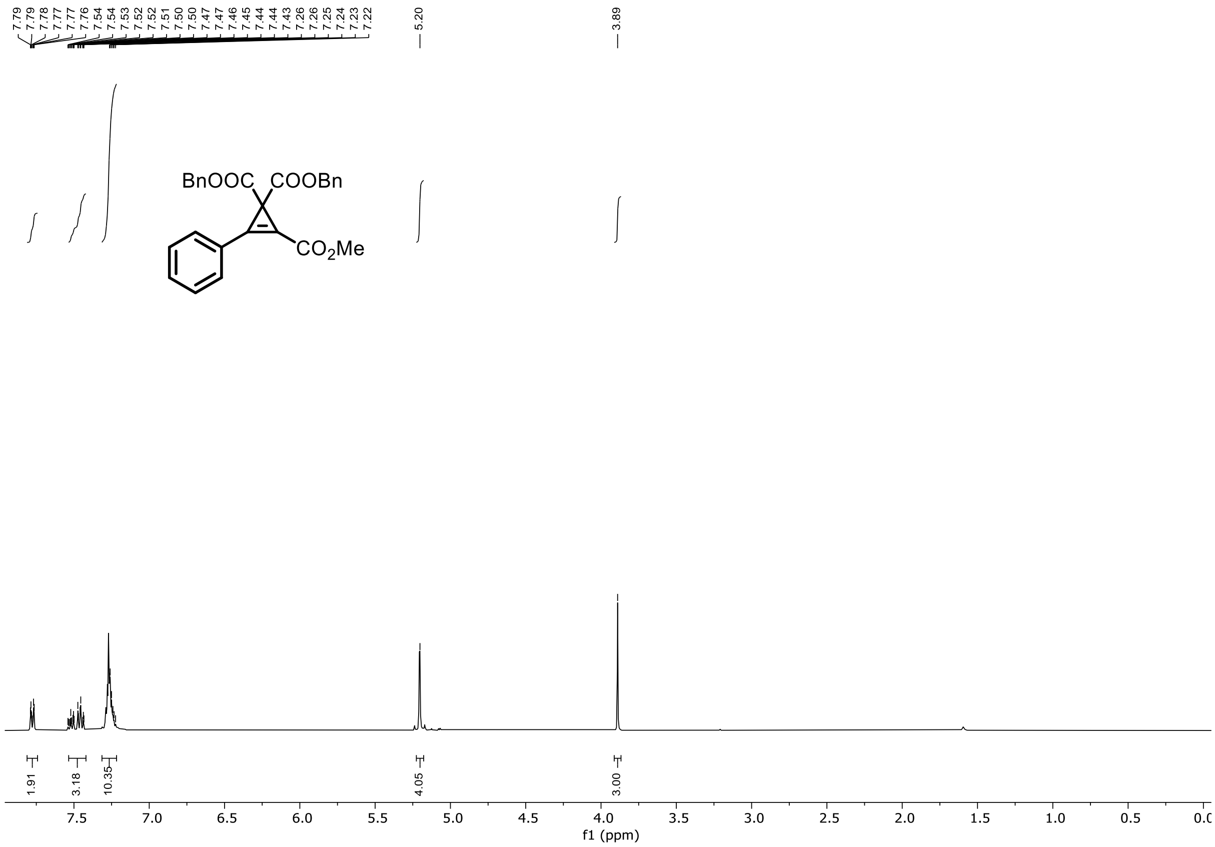

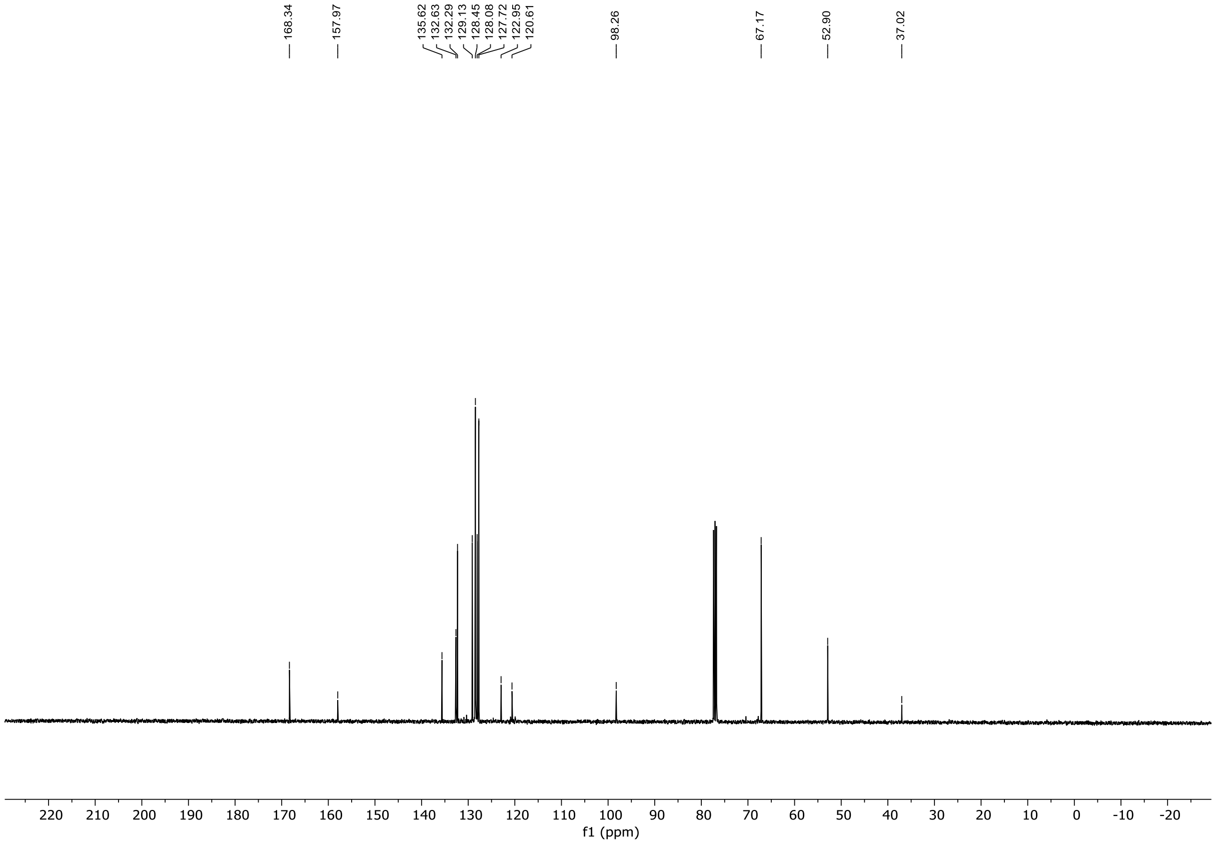

[back to Table of Contents]

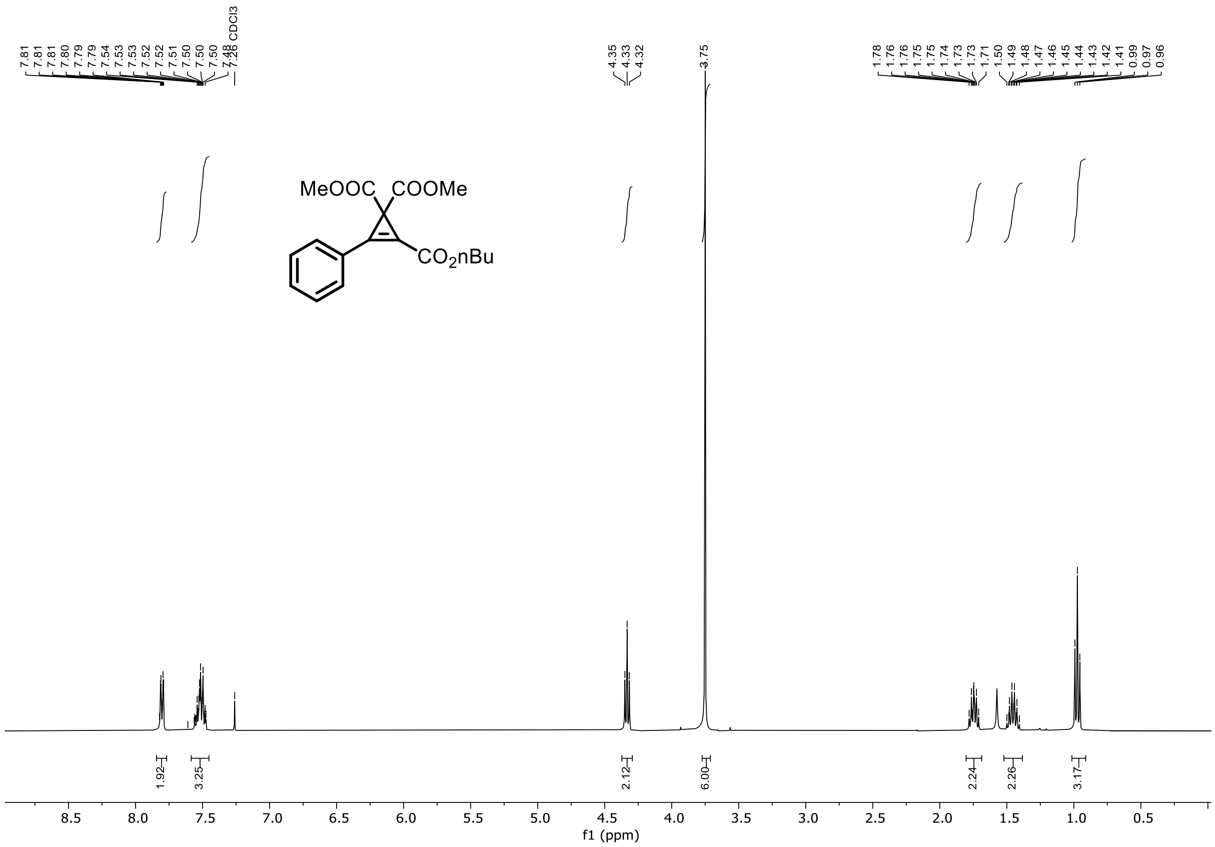

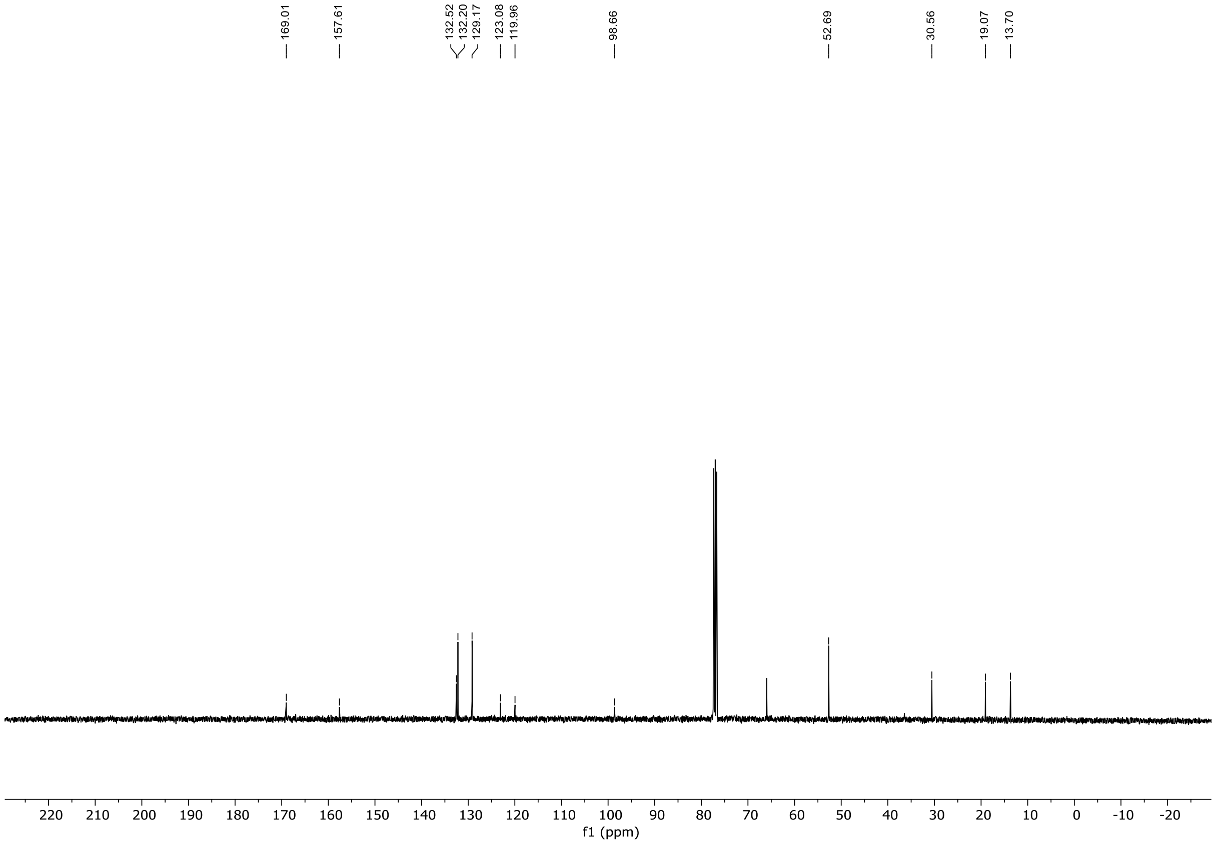

[back to Table of Contents]

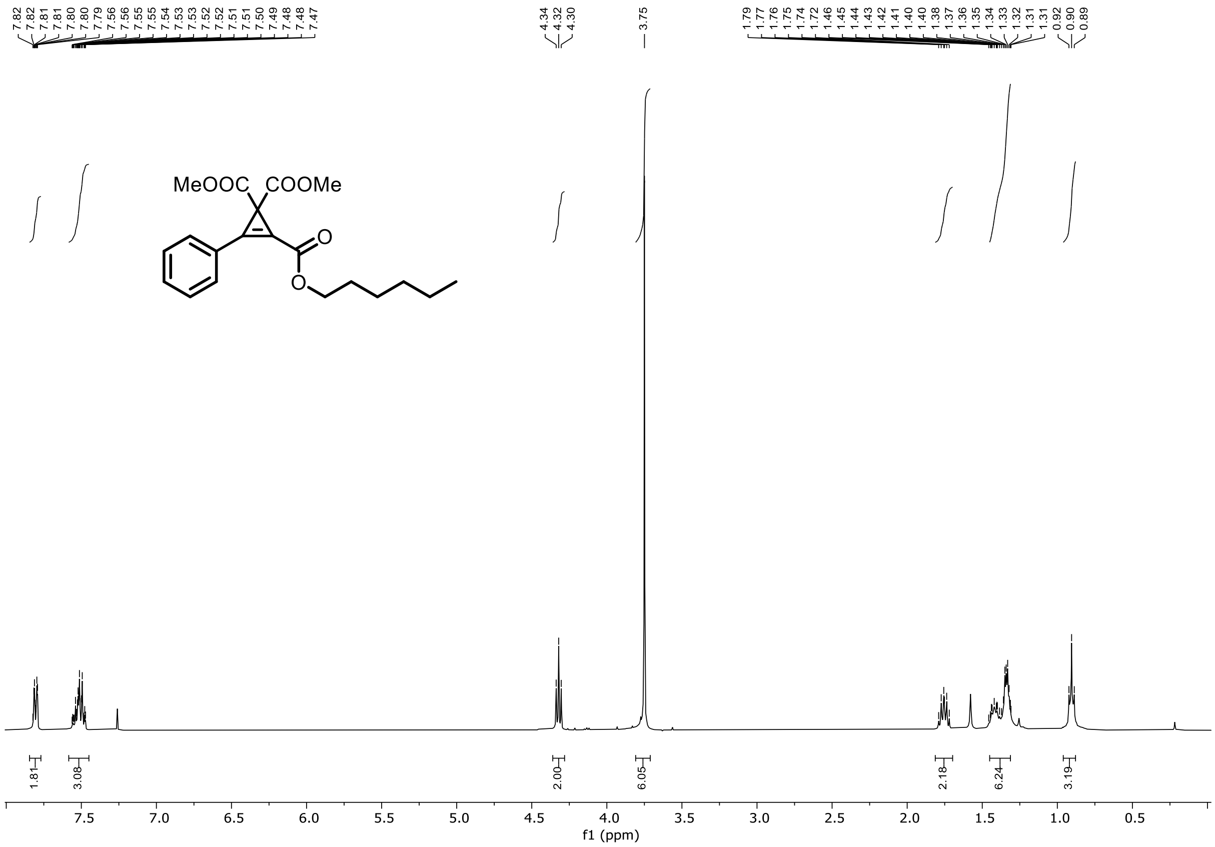

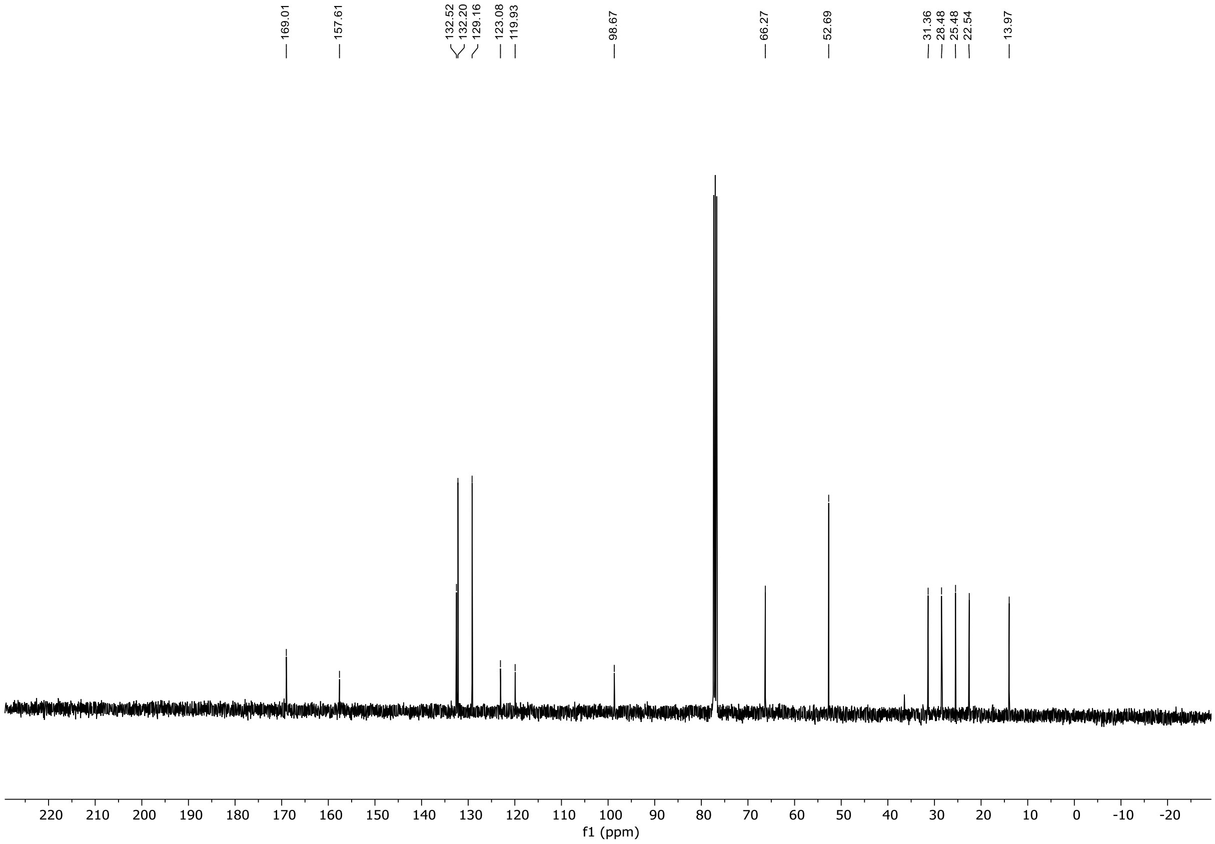

[back to Table of Contents]

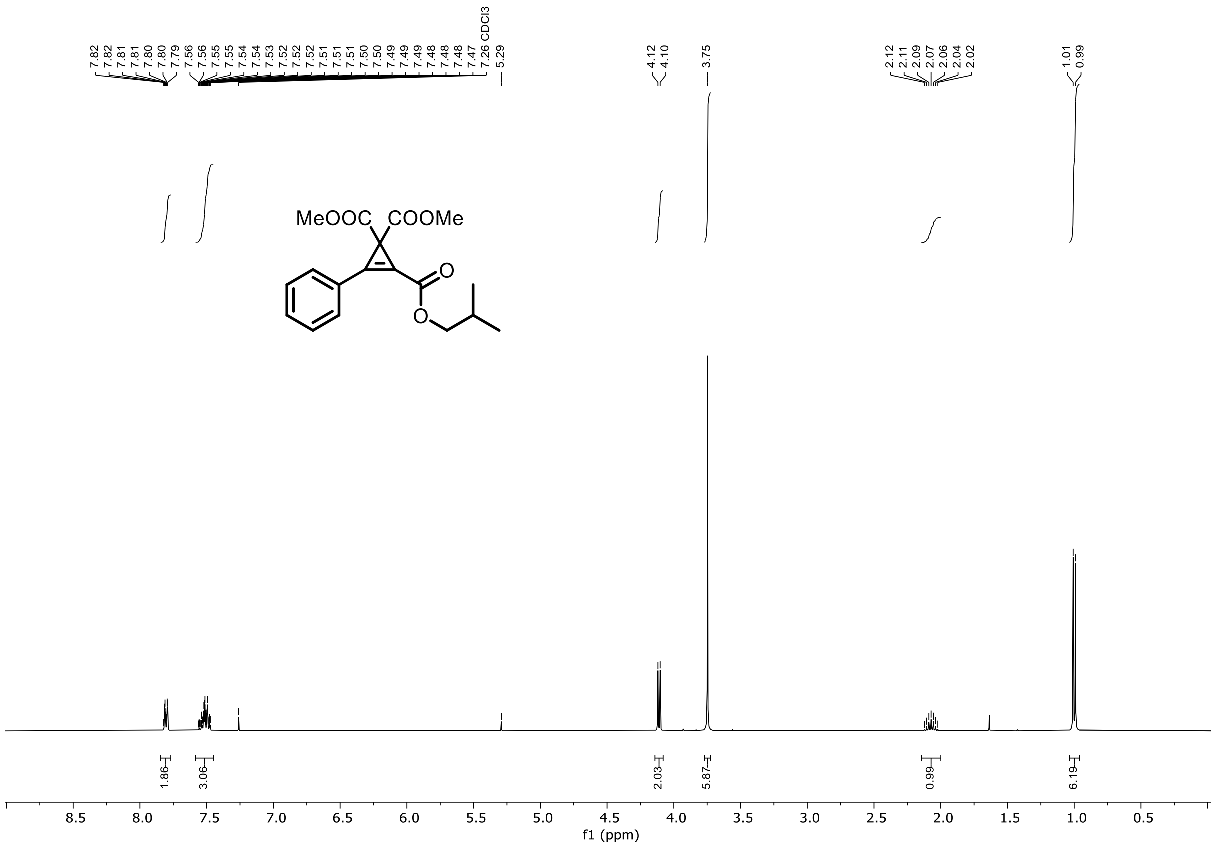

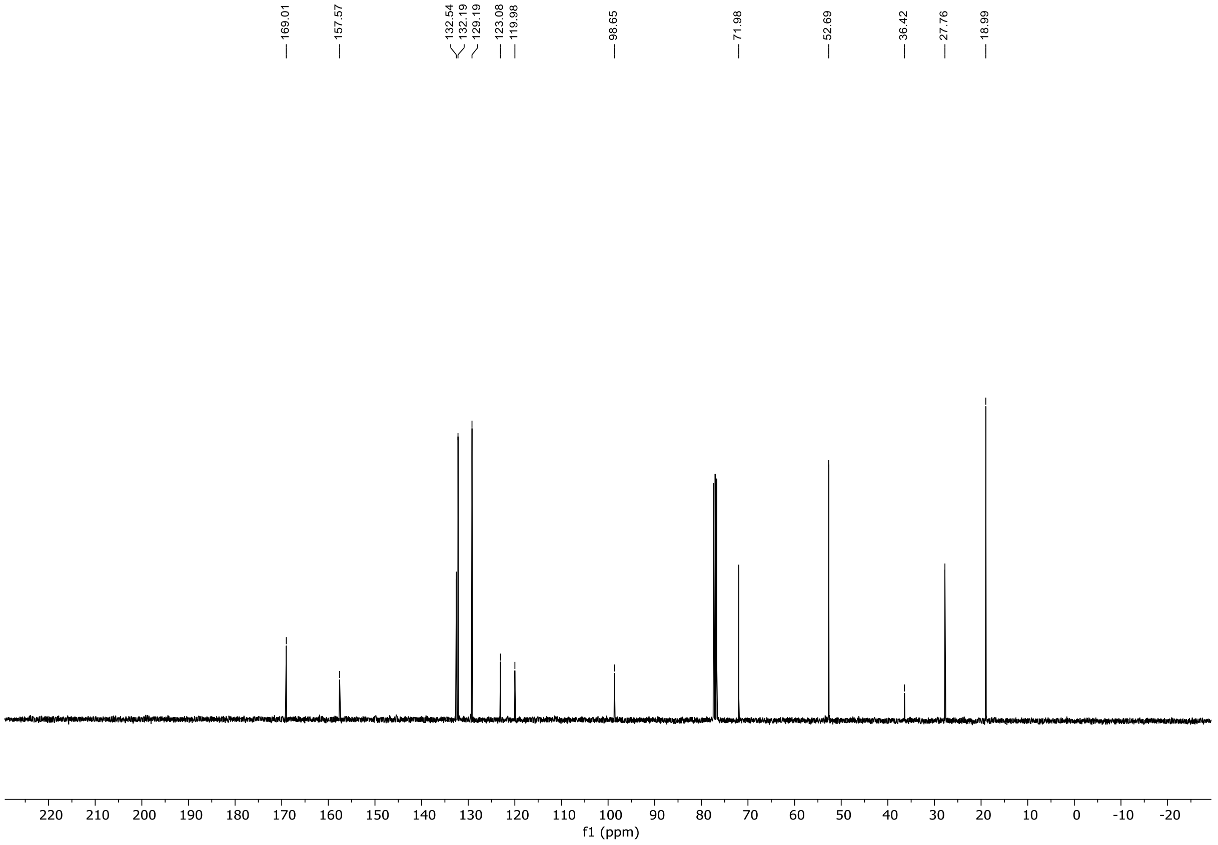

[back to Table of Contents]

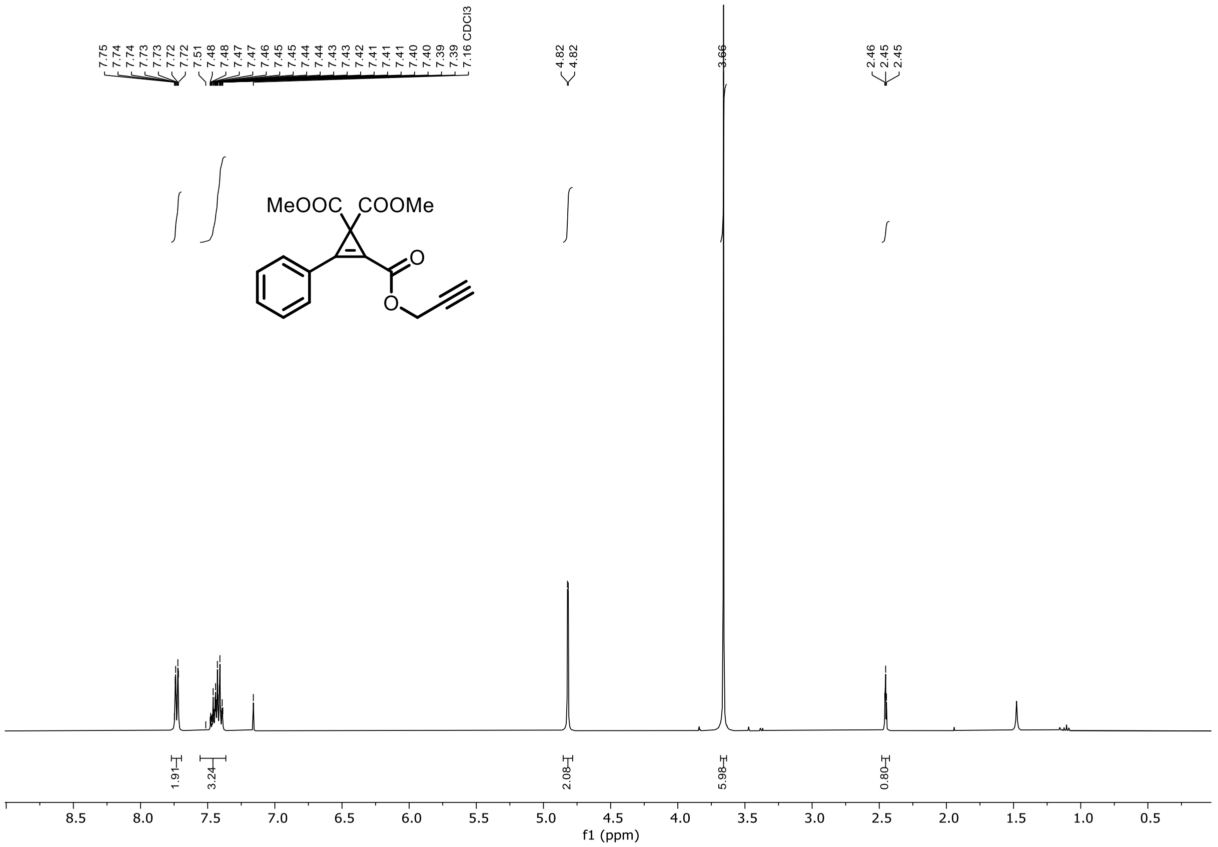

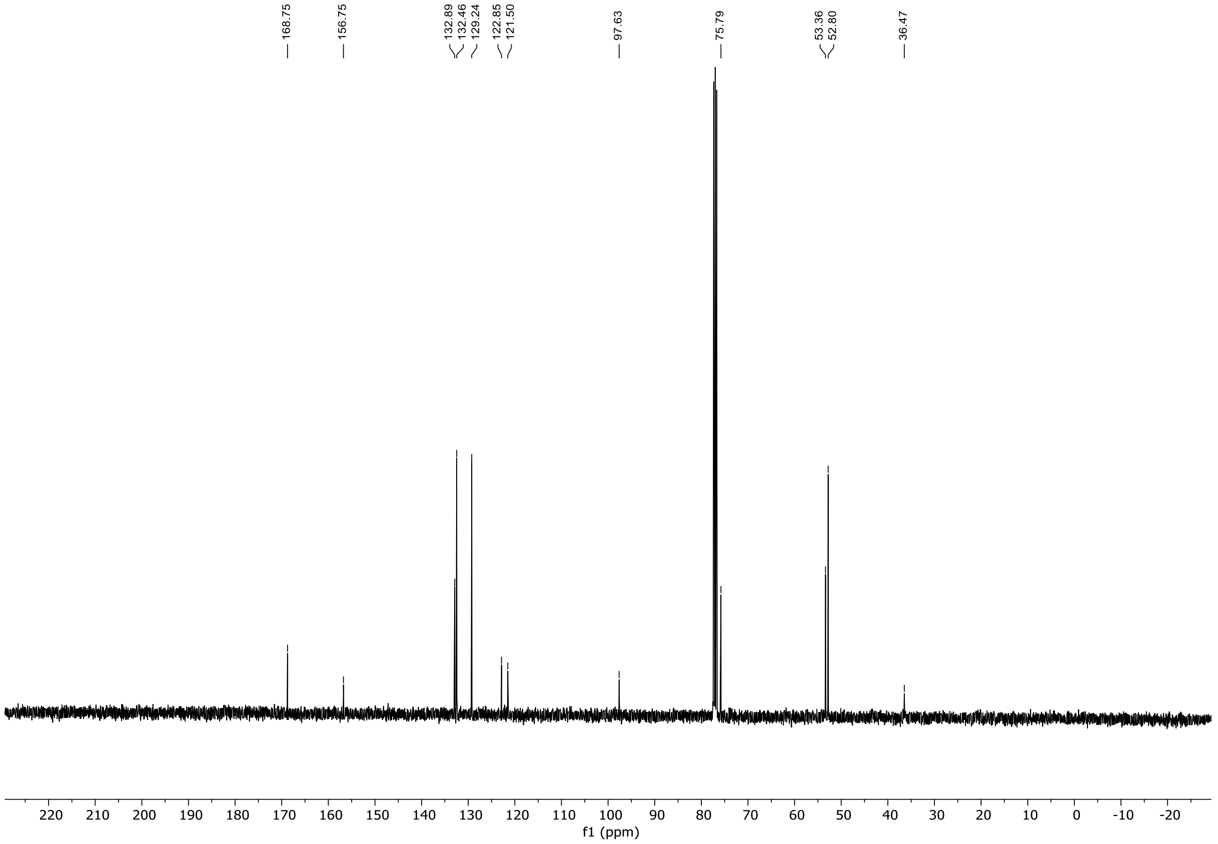

[back to Table of Contents]

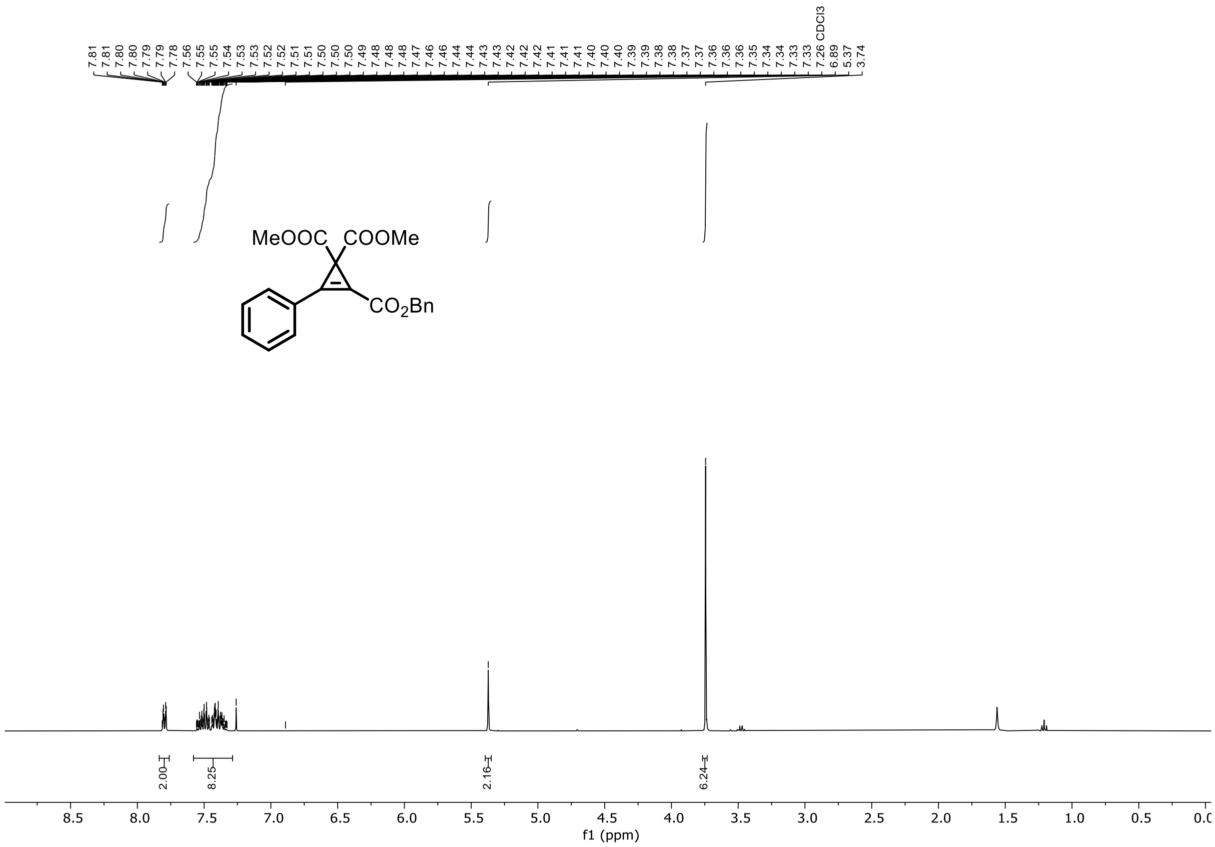

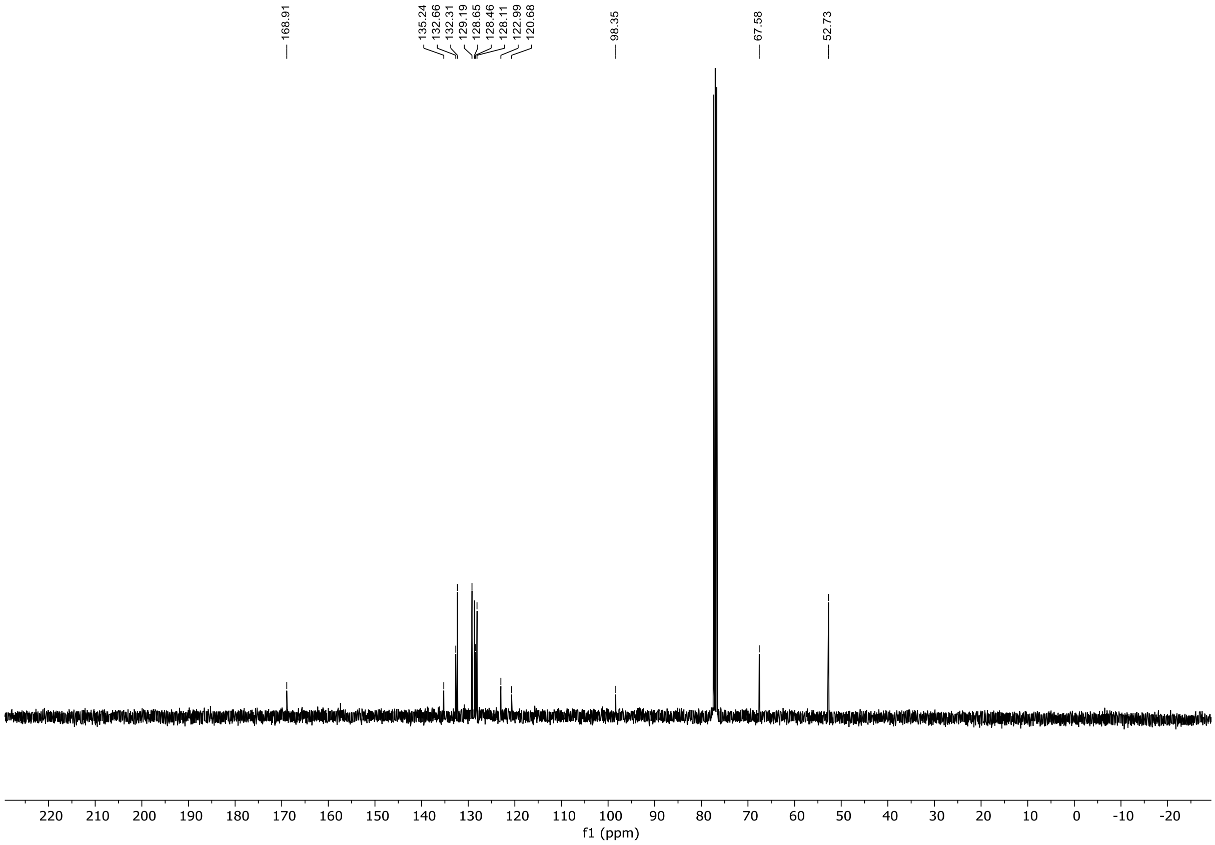

[back to Table of Contents]

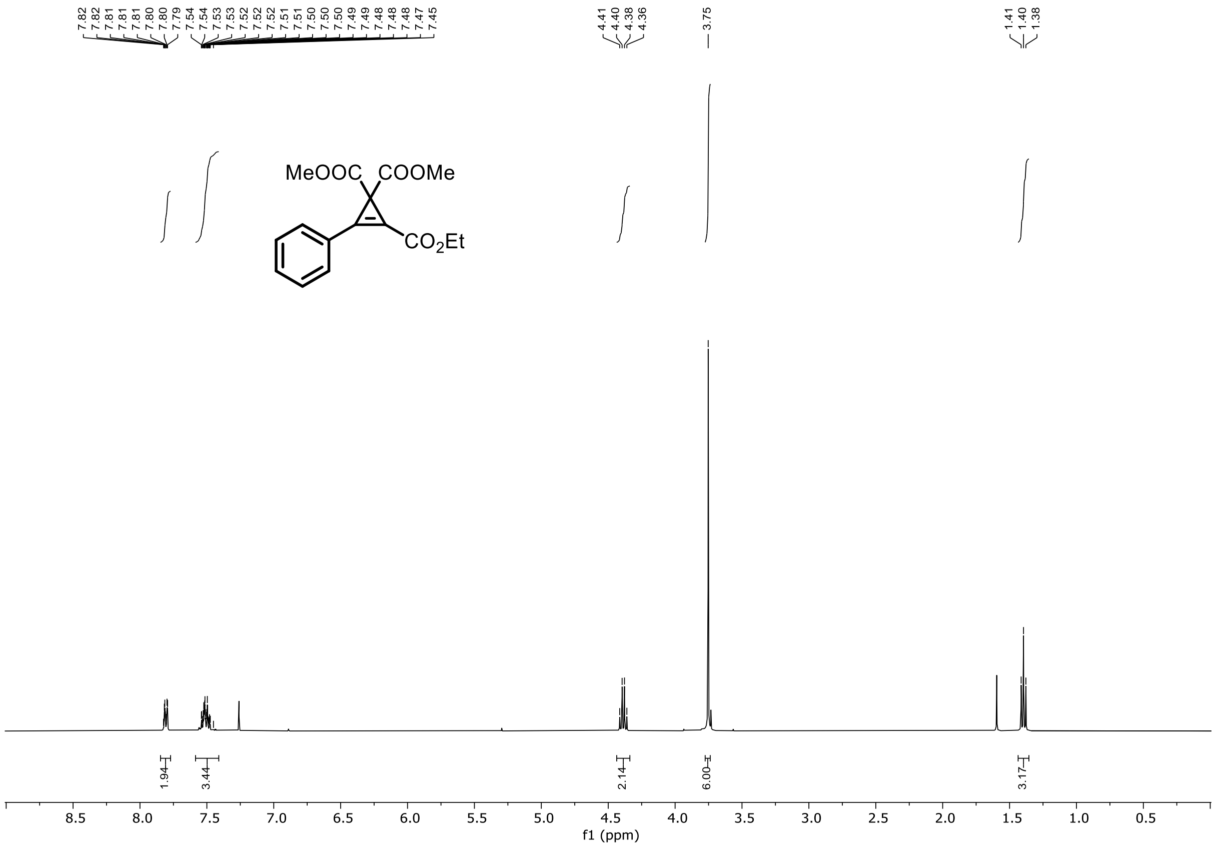

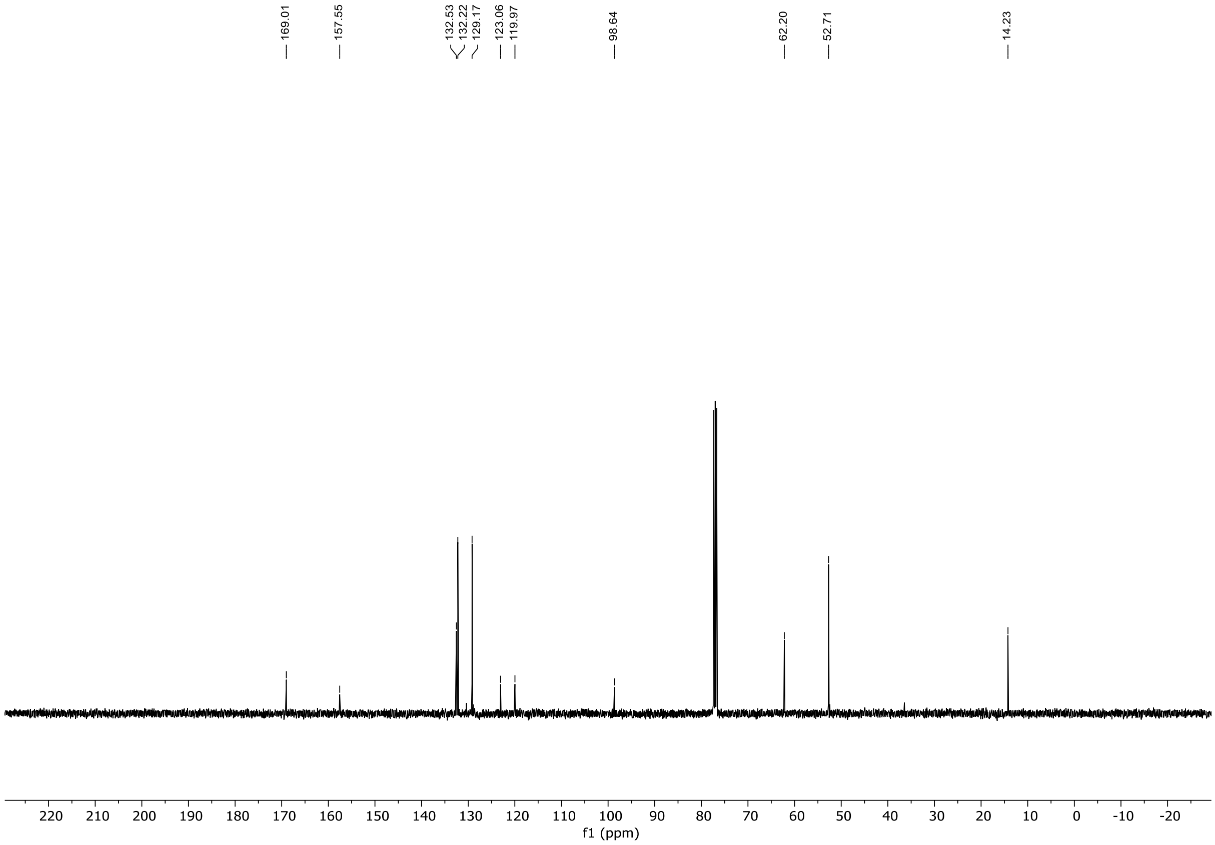

[back to Table of Contents]

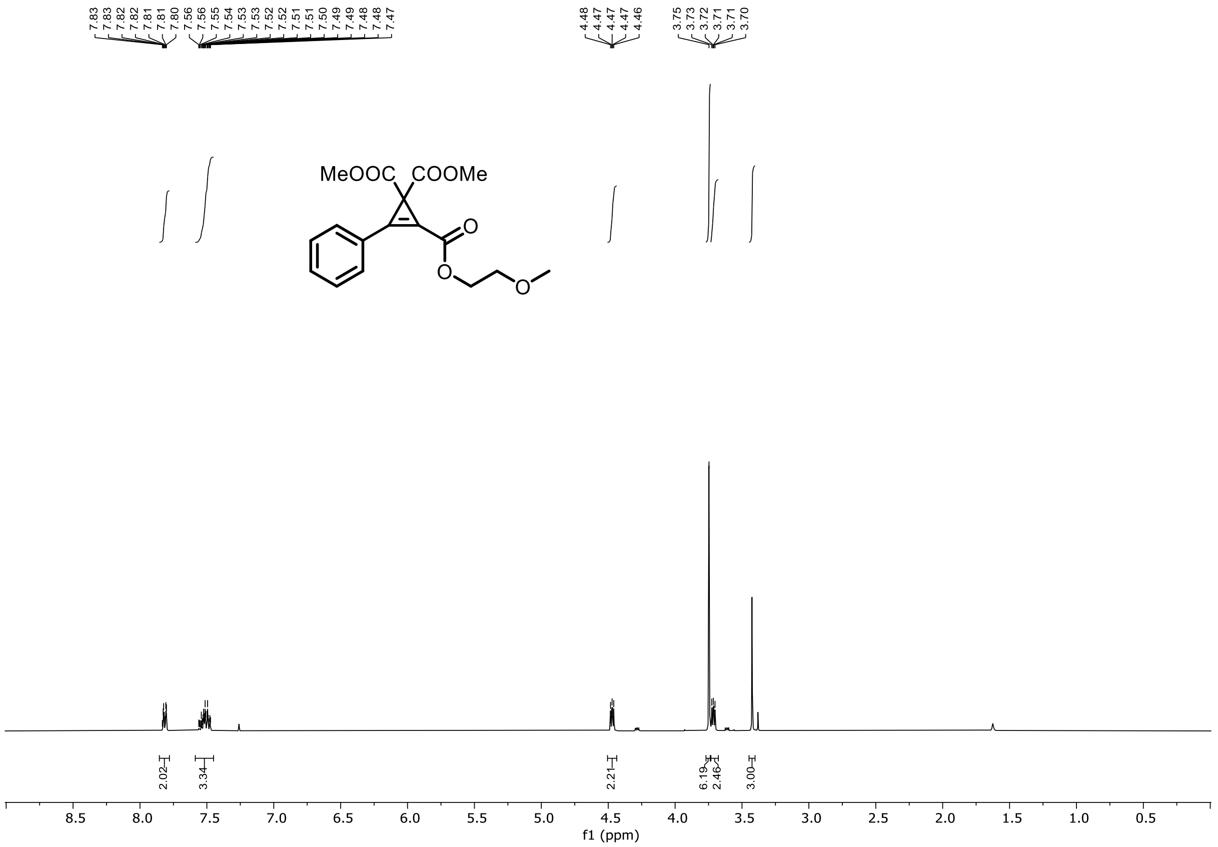

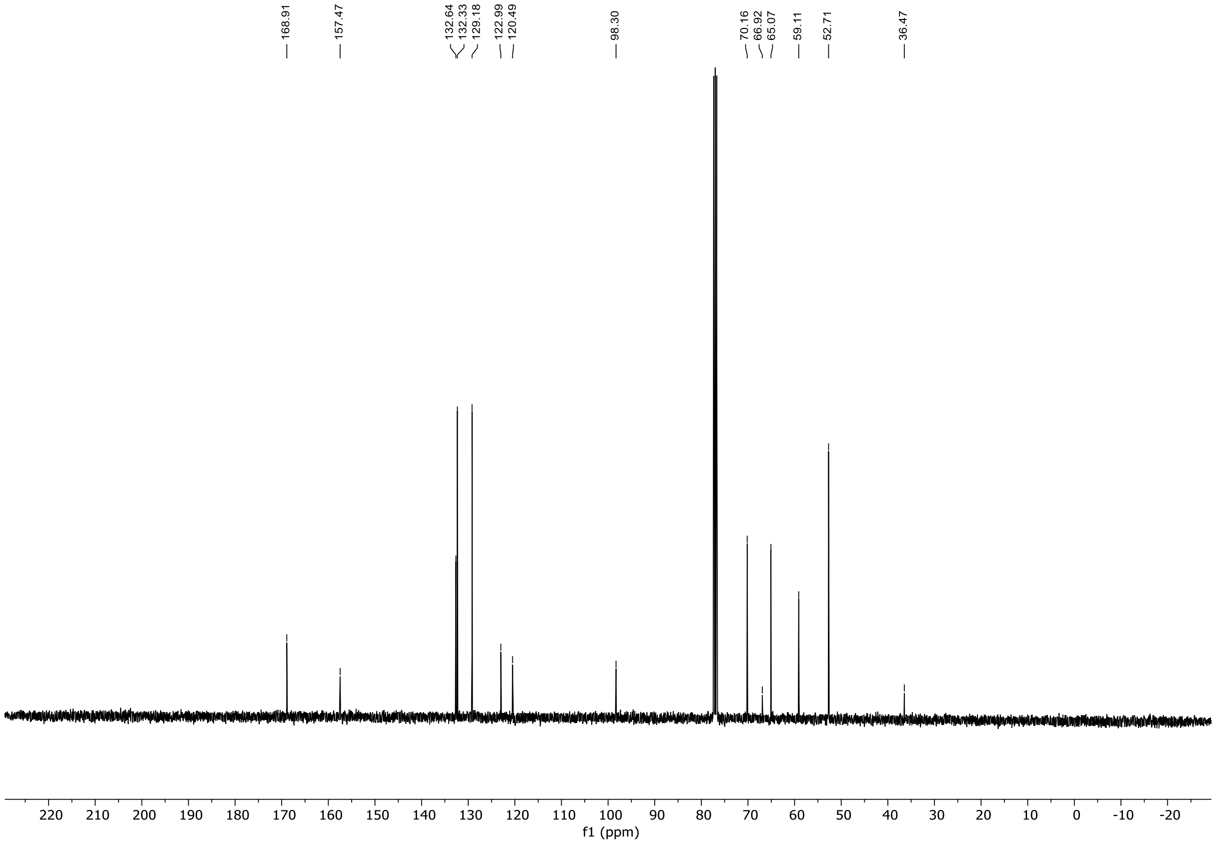

[back to Table of Contents]

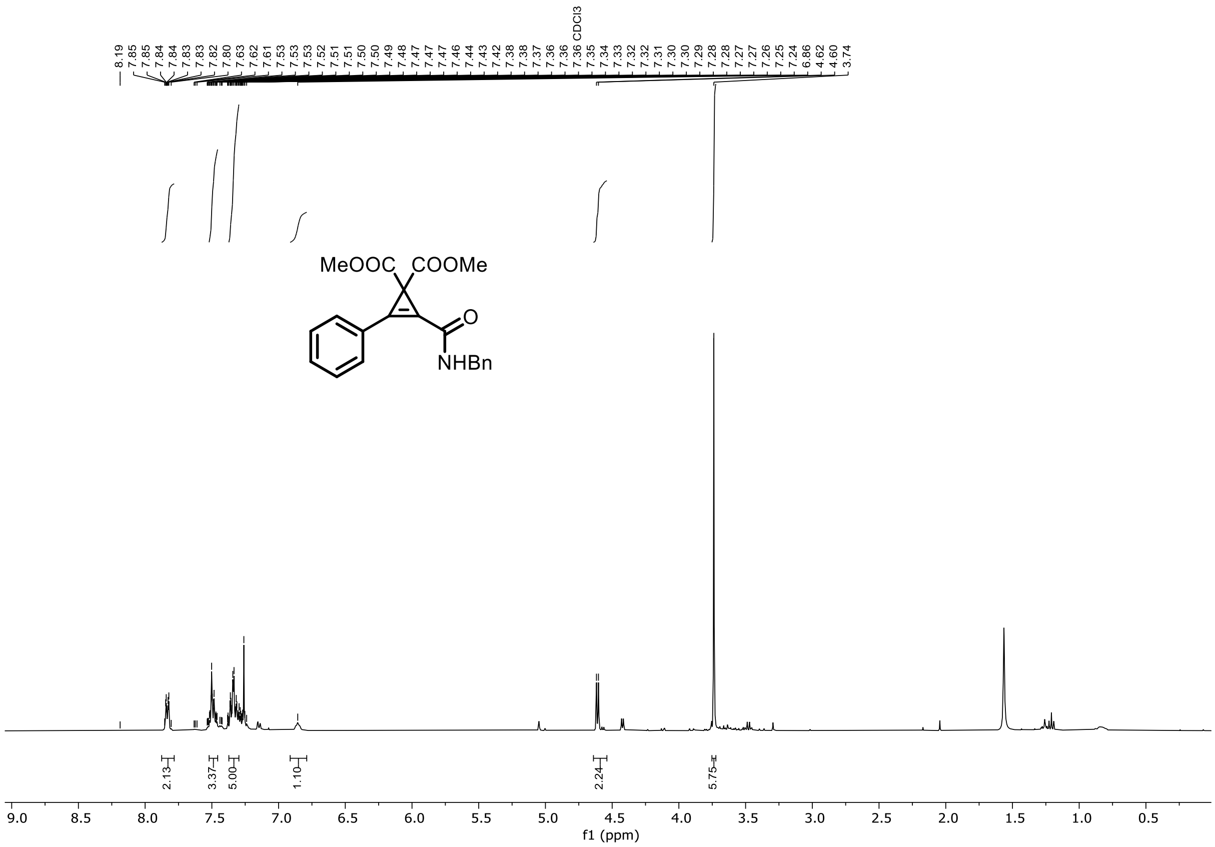

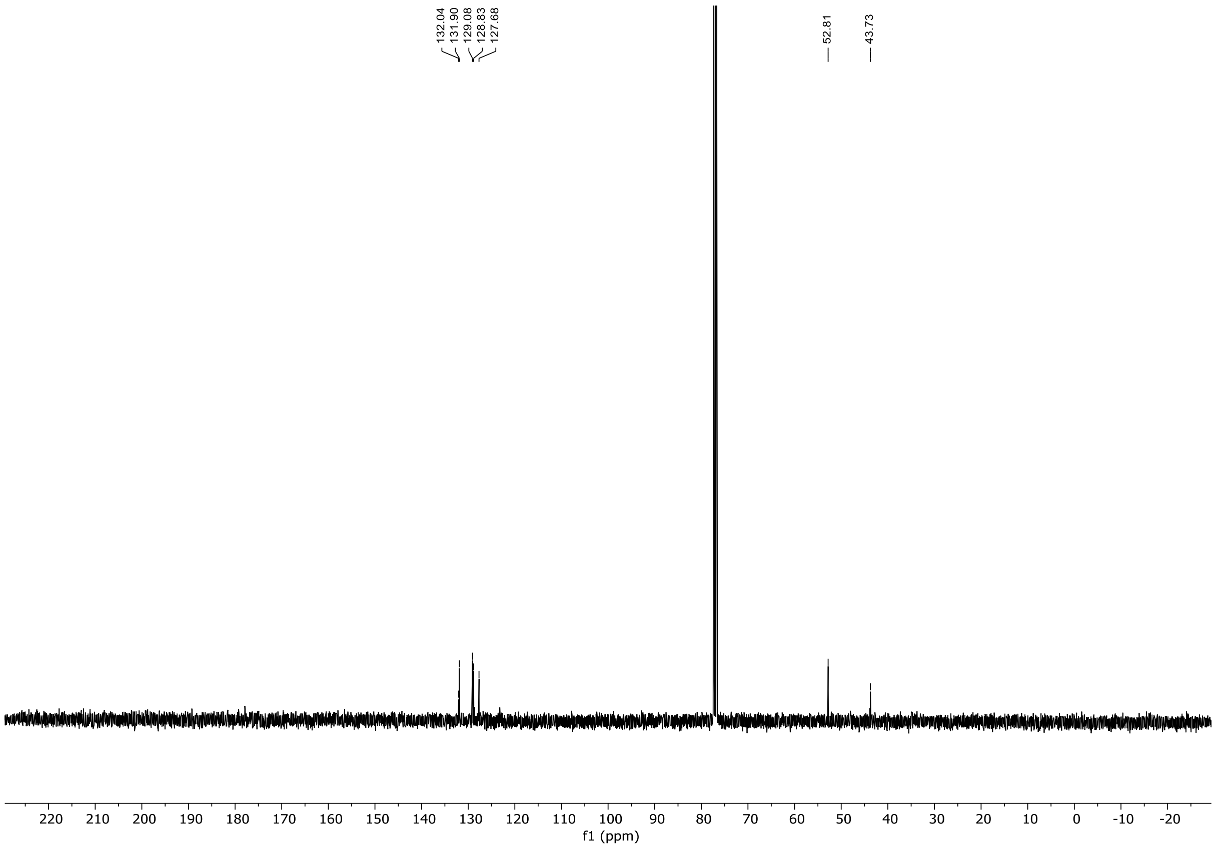

[back to Table of Contents]

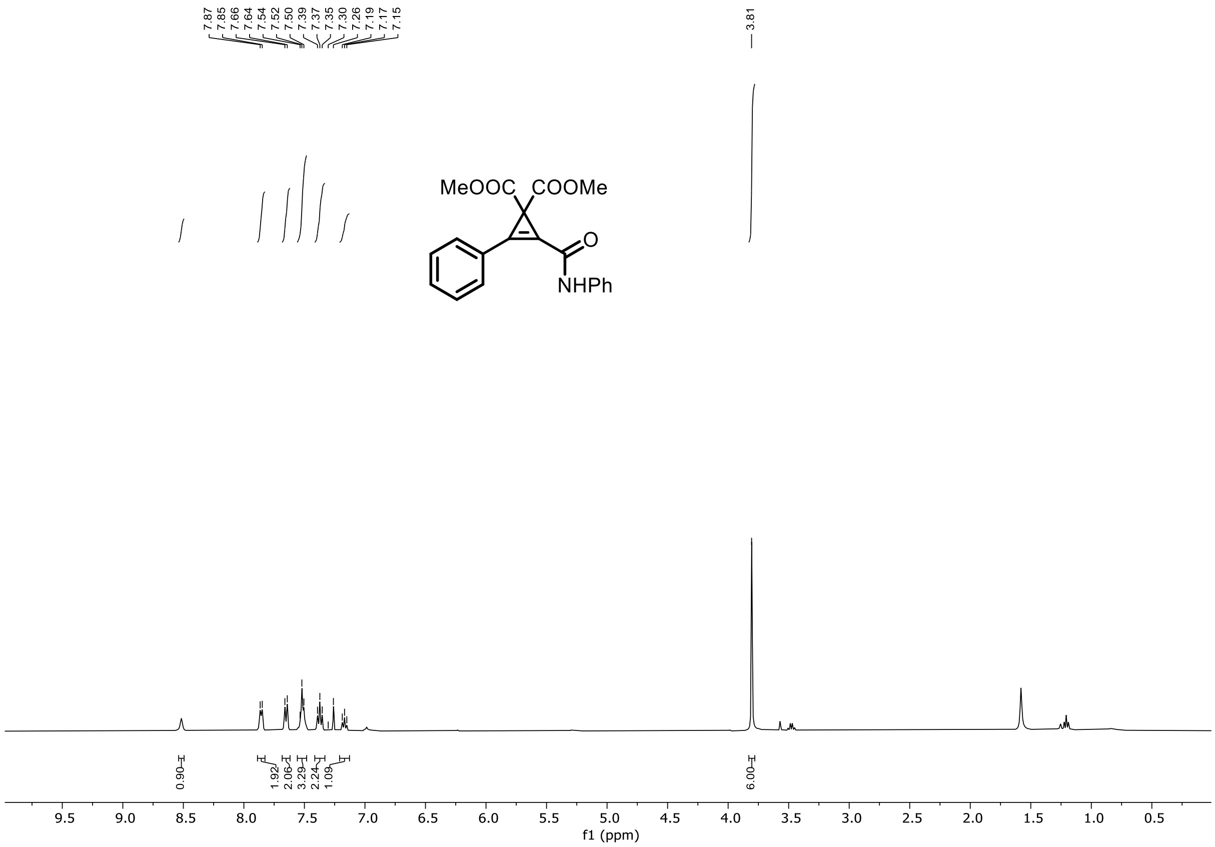

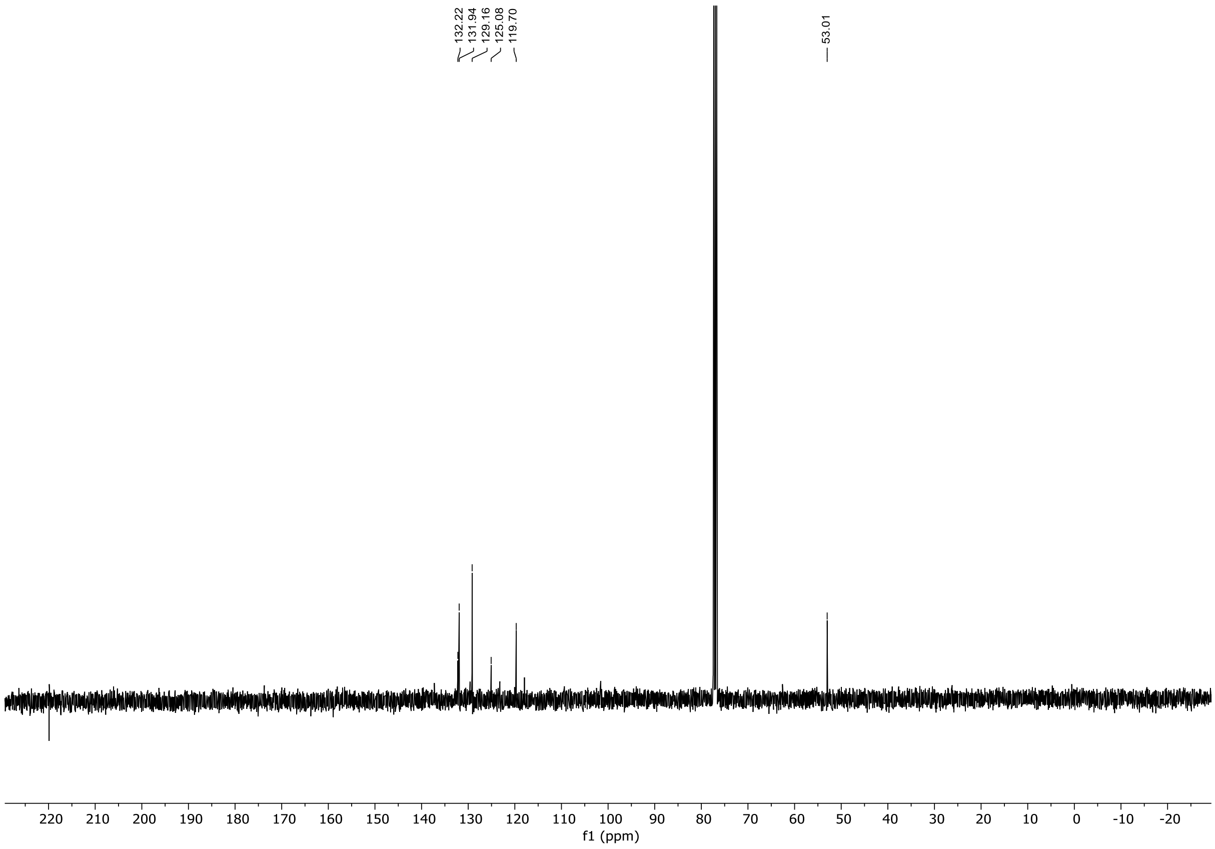

[back to Table of Contents]

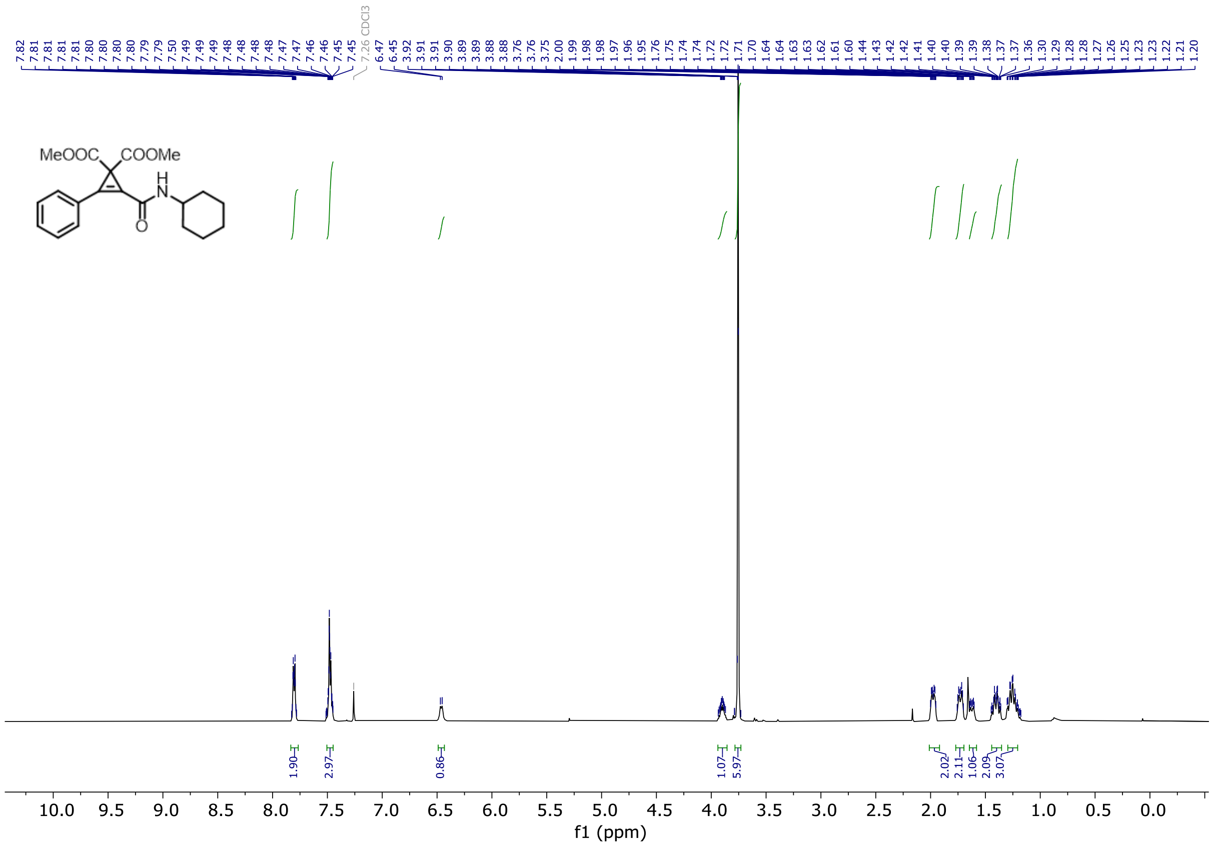

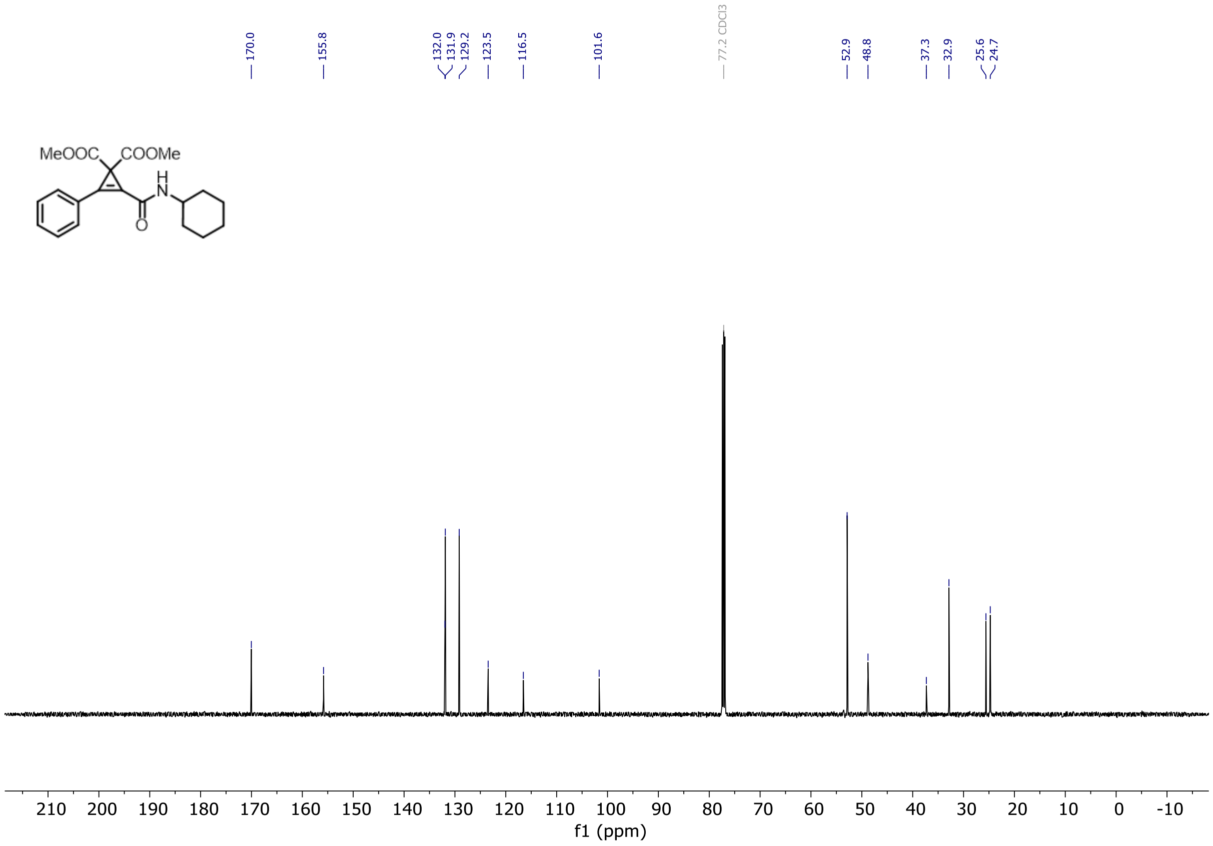

[back to Table of Contents]

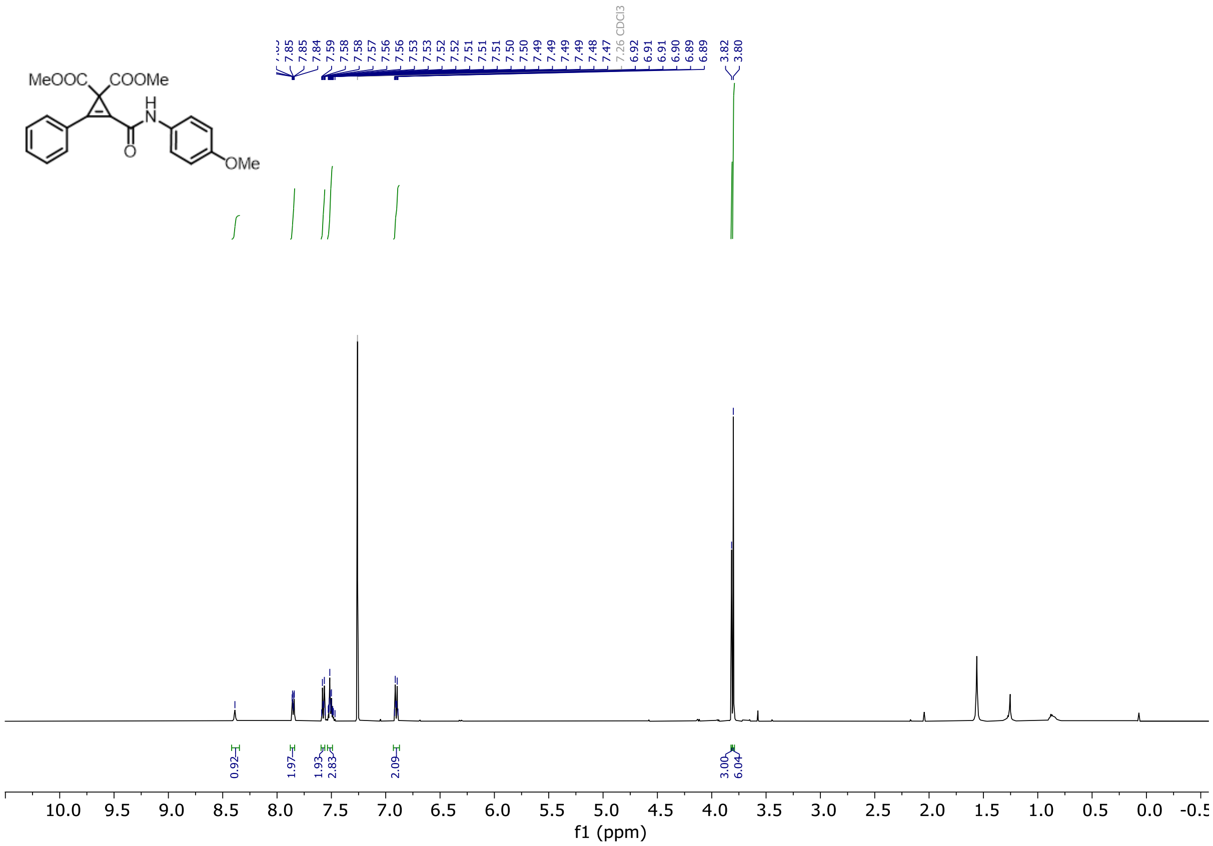

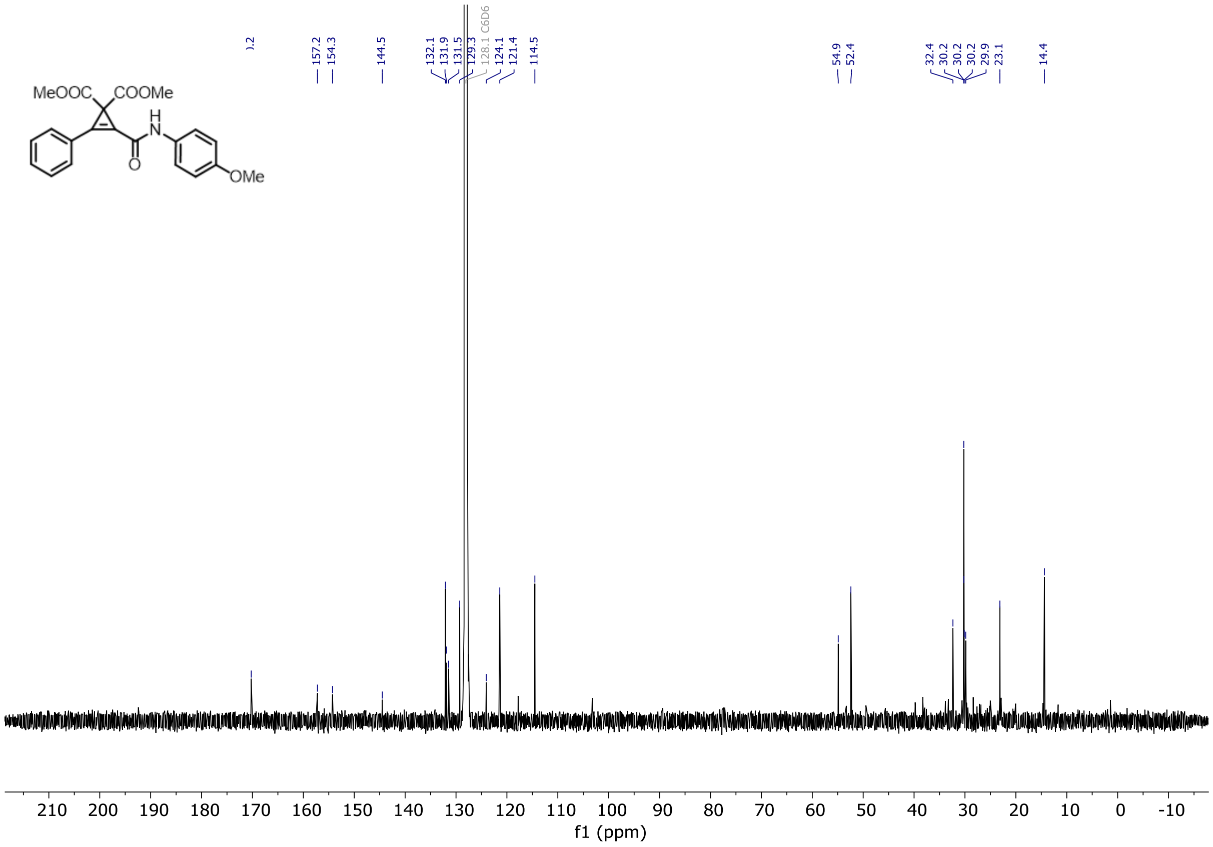

[back to Table of Contents]

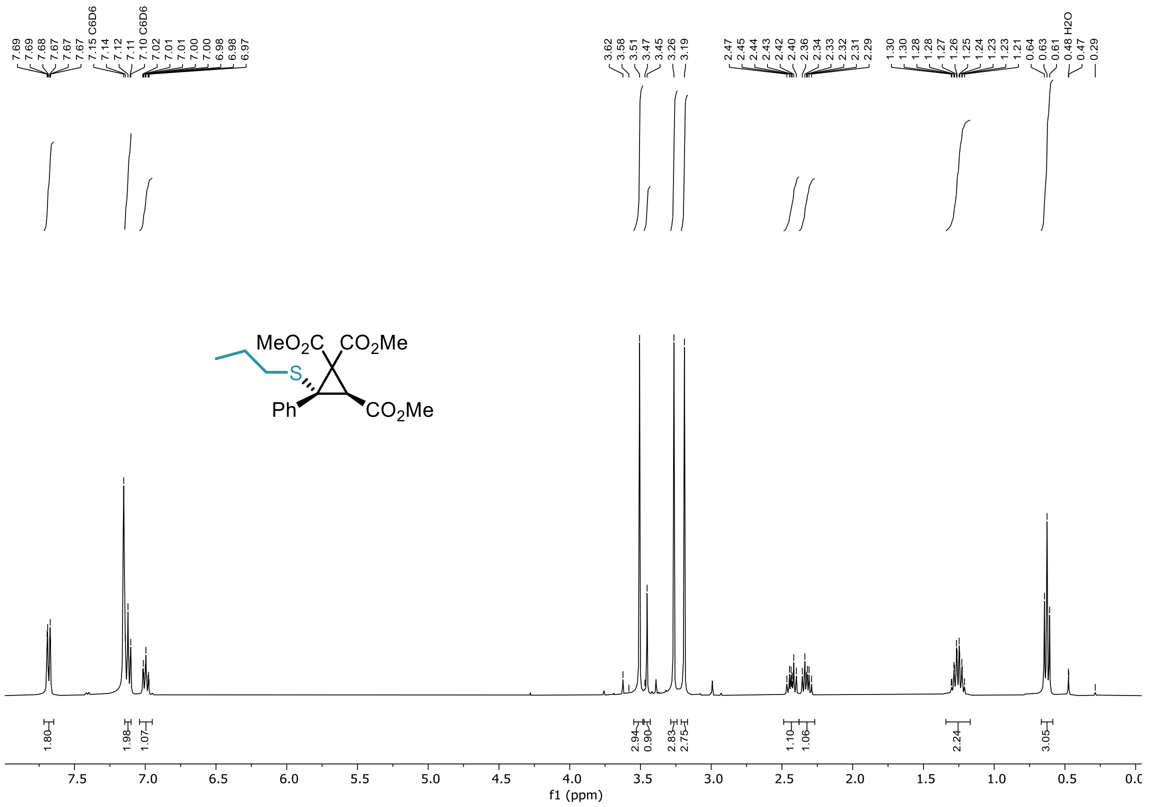

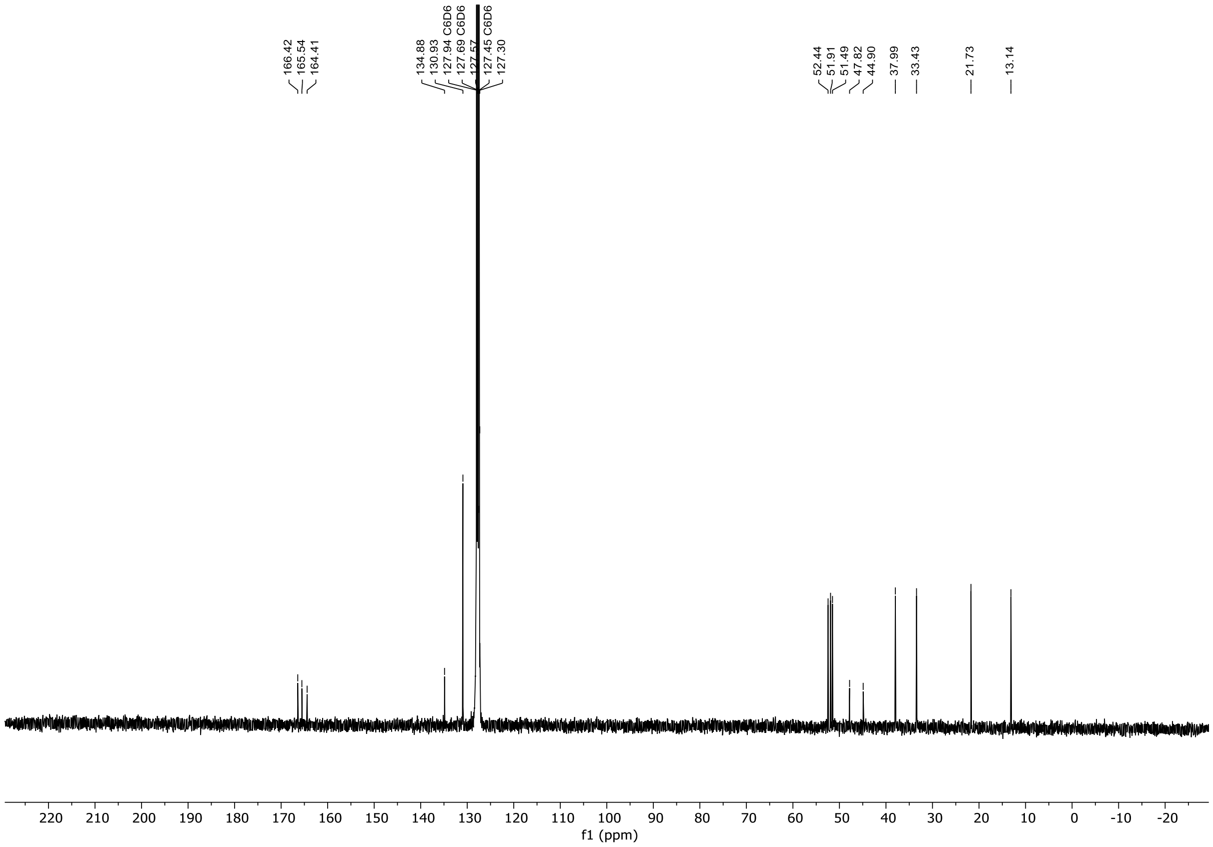

[back to Table of Contents]

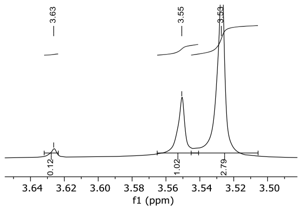

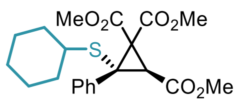

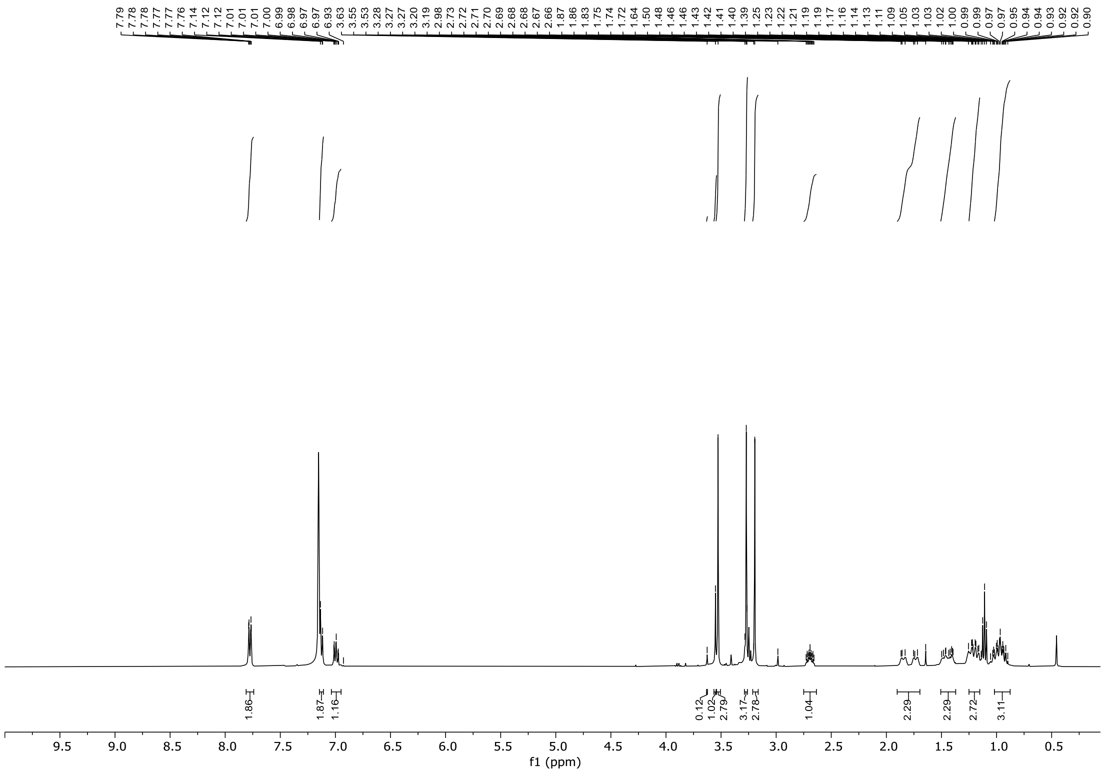

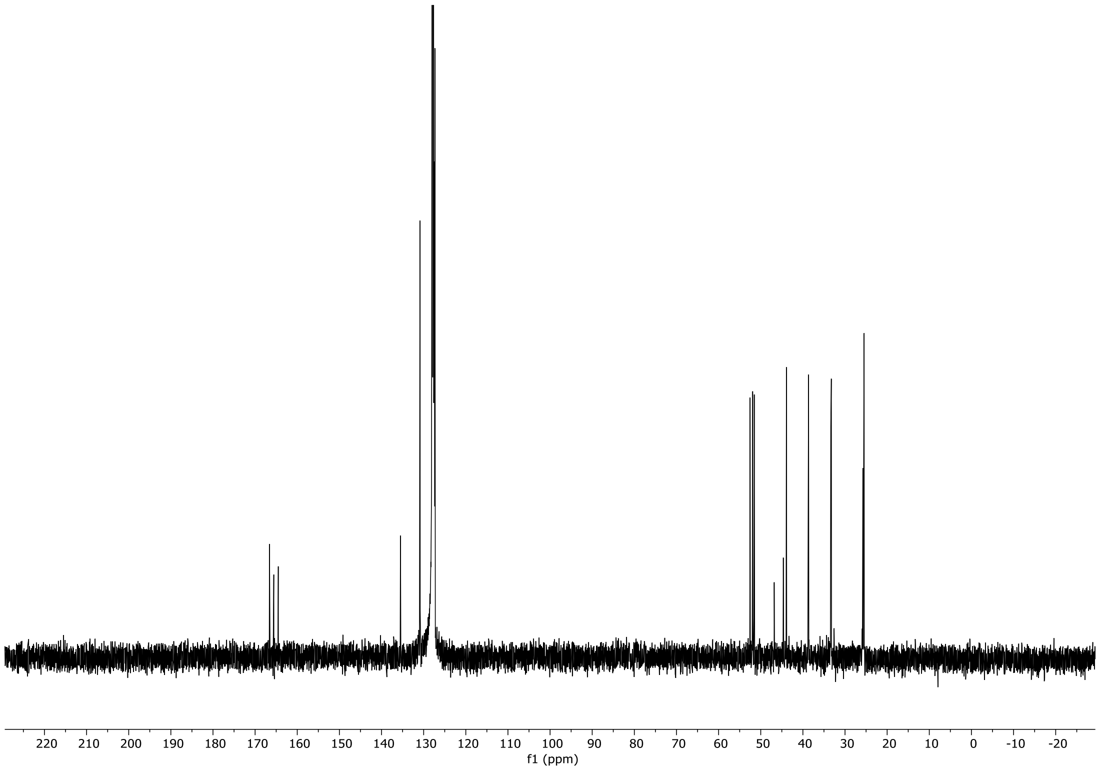

[back to Table of Contents]

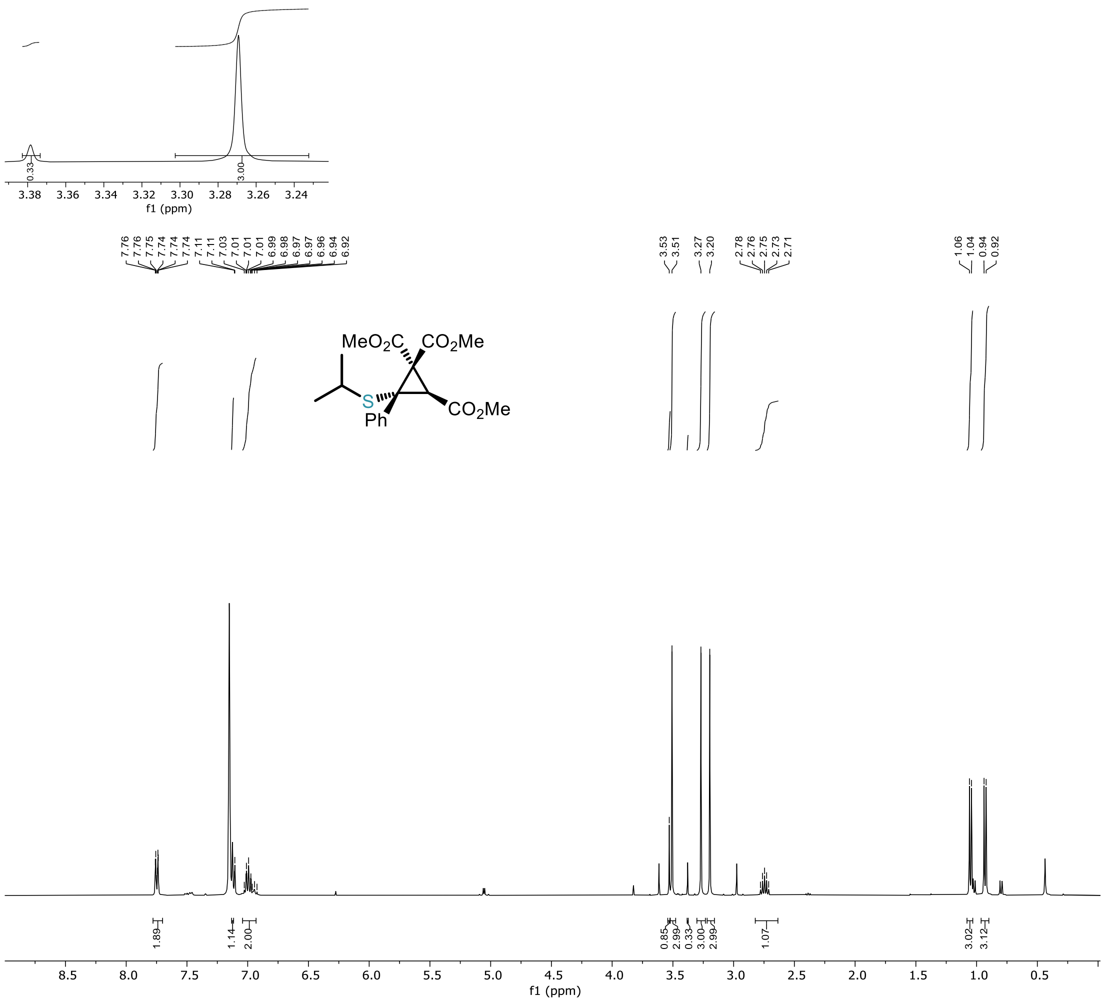

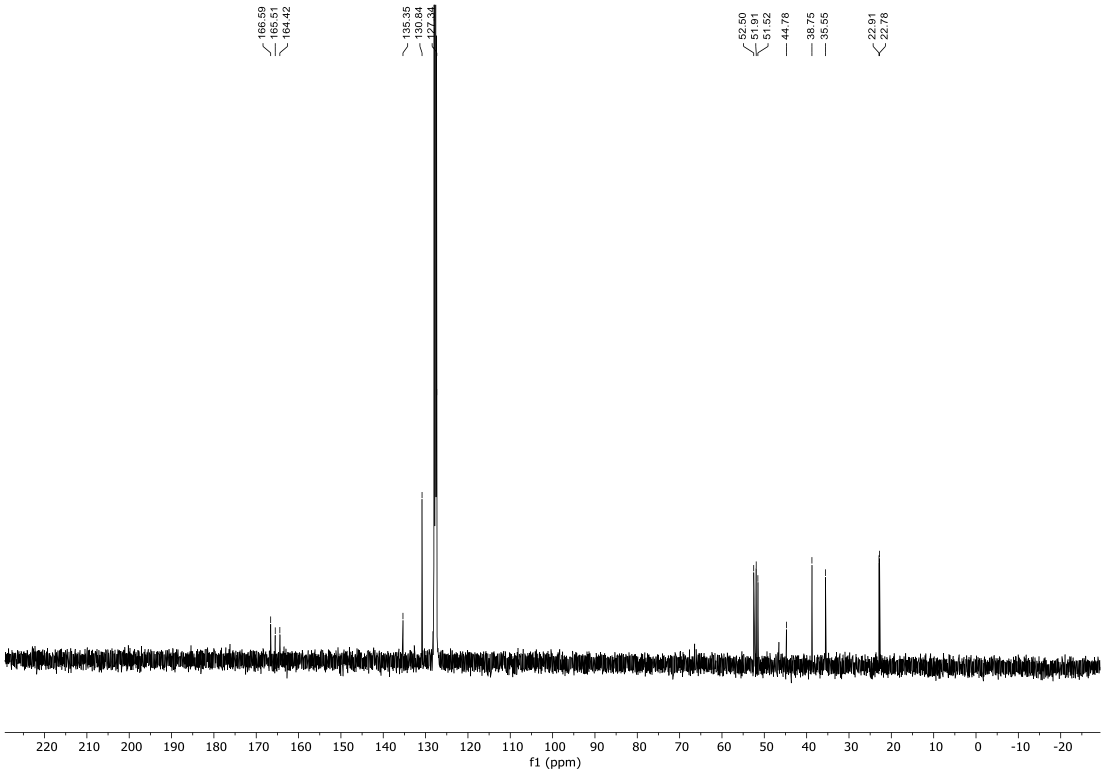

[back to Table of Contents]

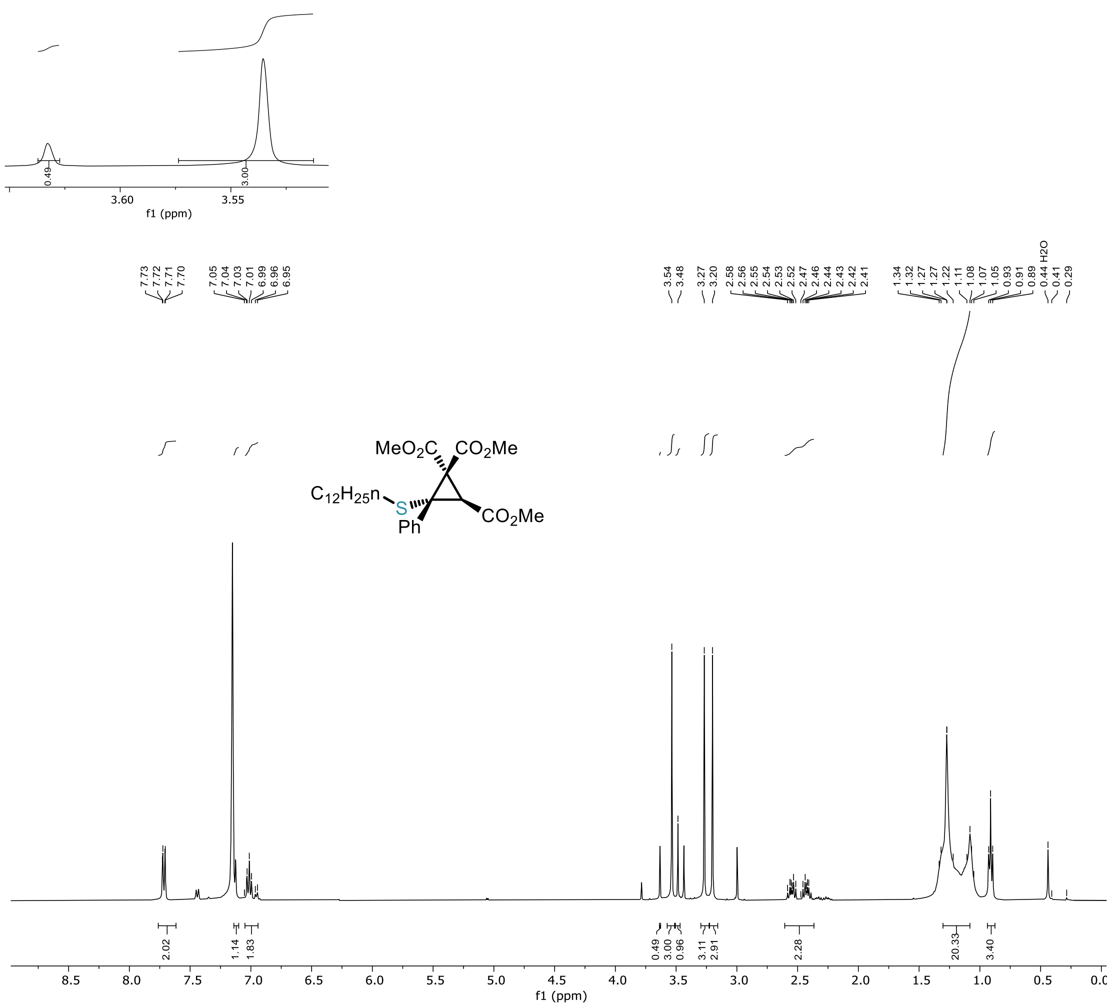

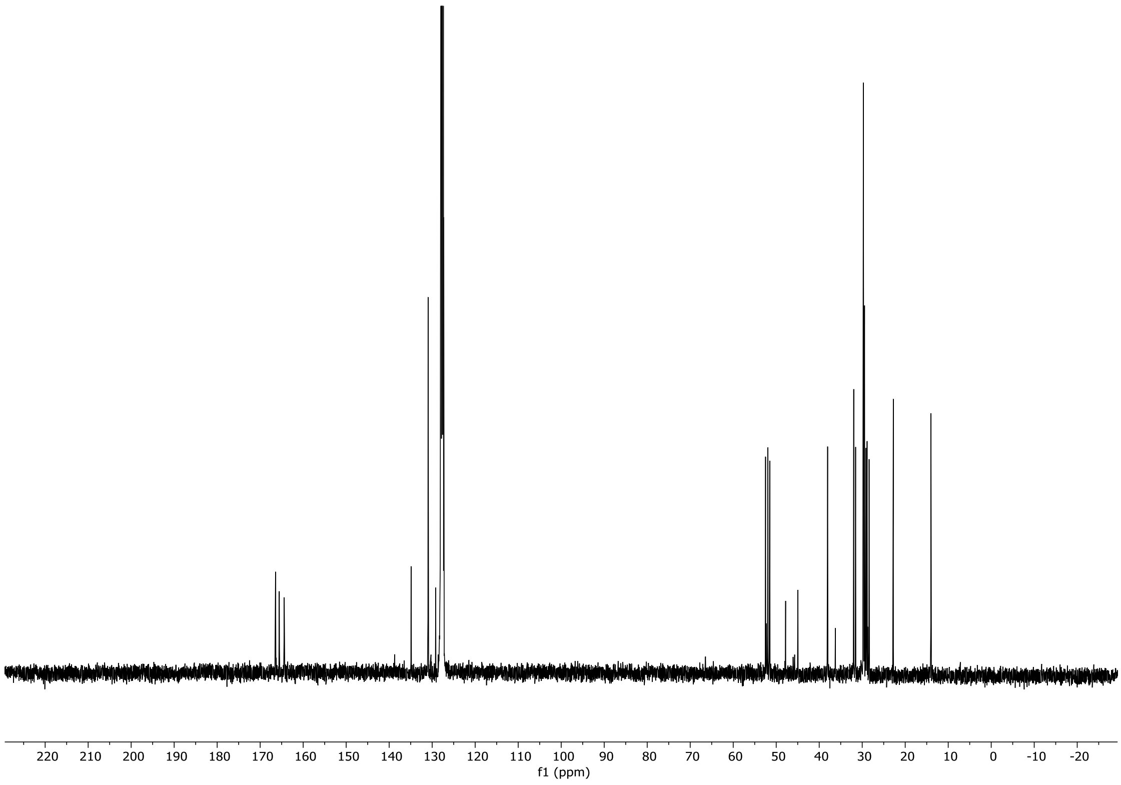

[back to Table of Contents]

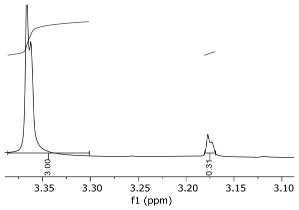

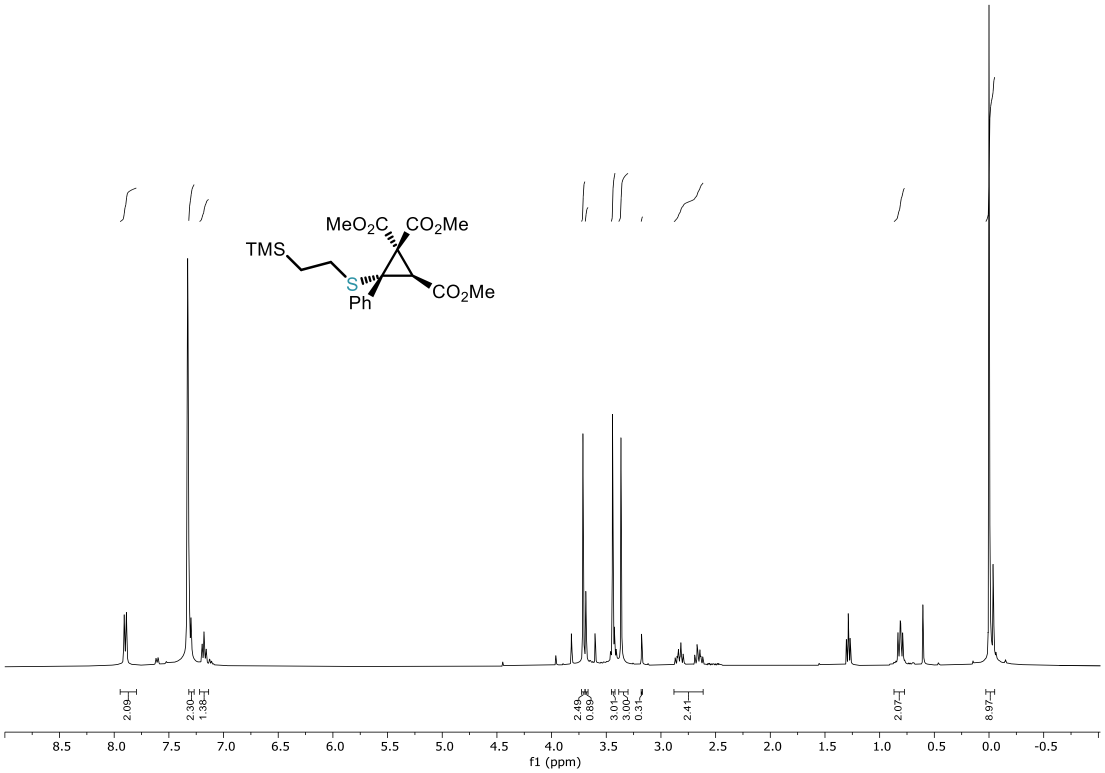

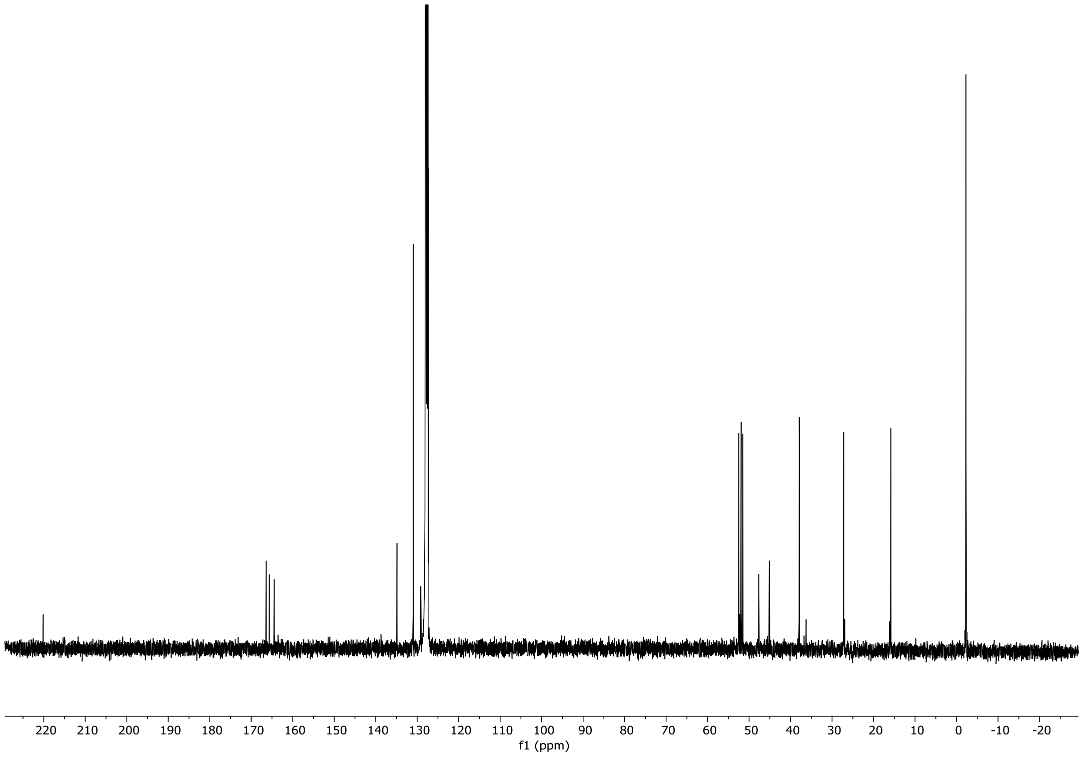

[back to Table of Contents]

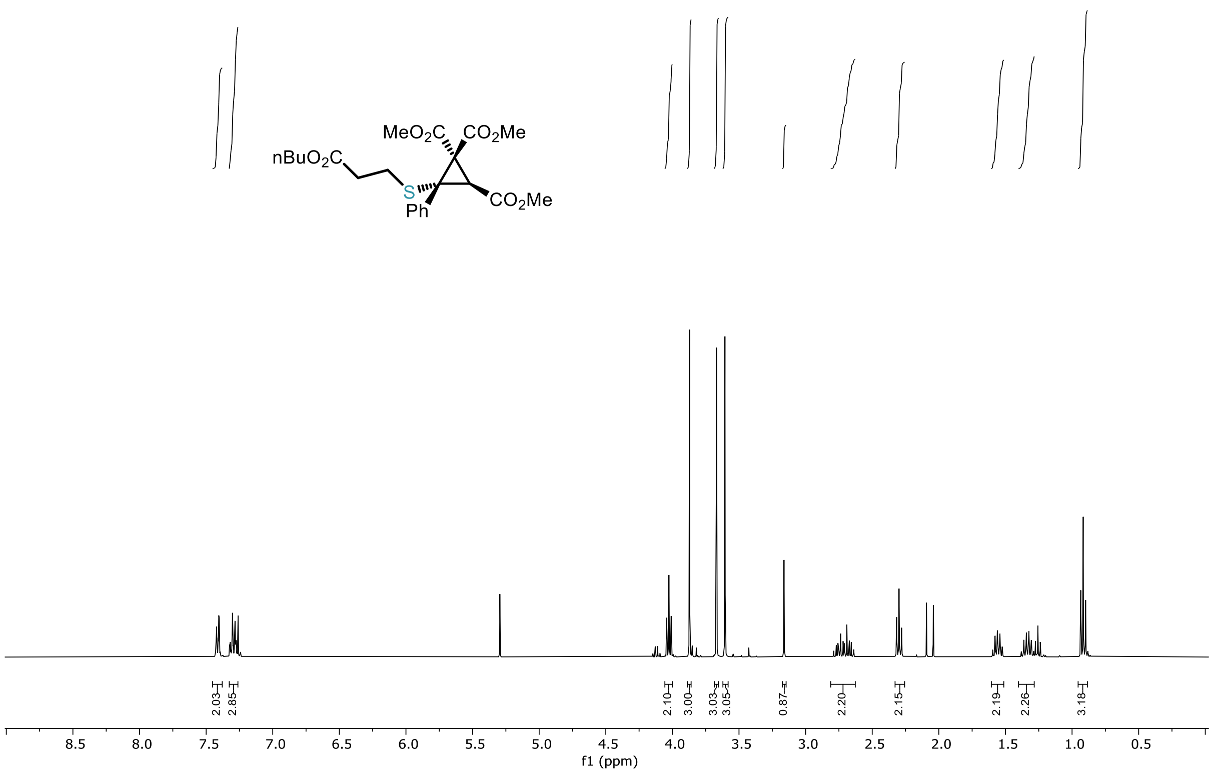

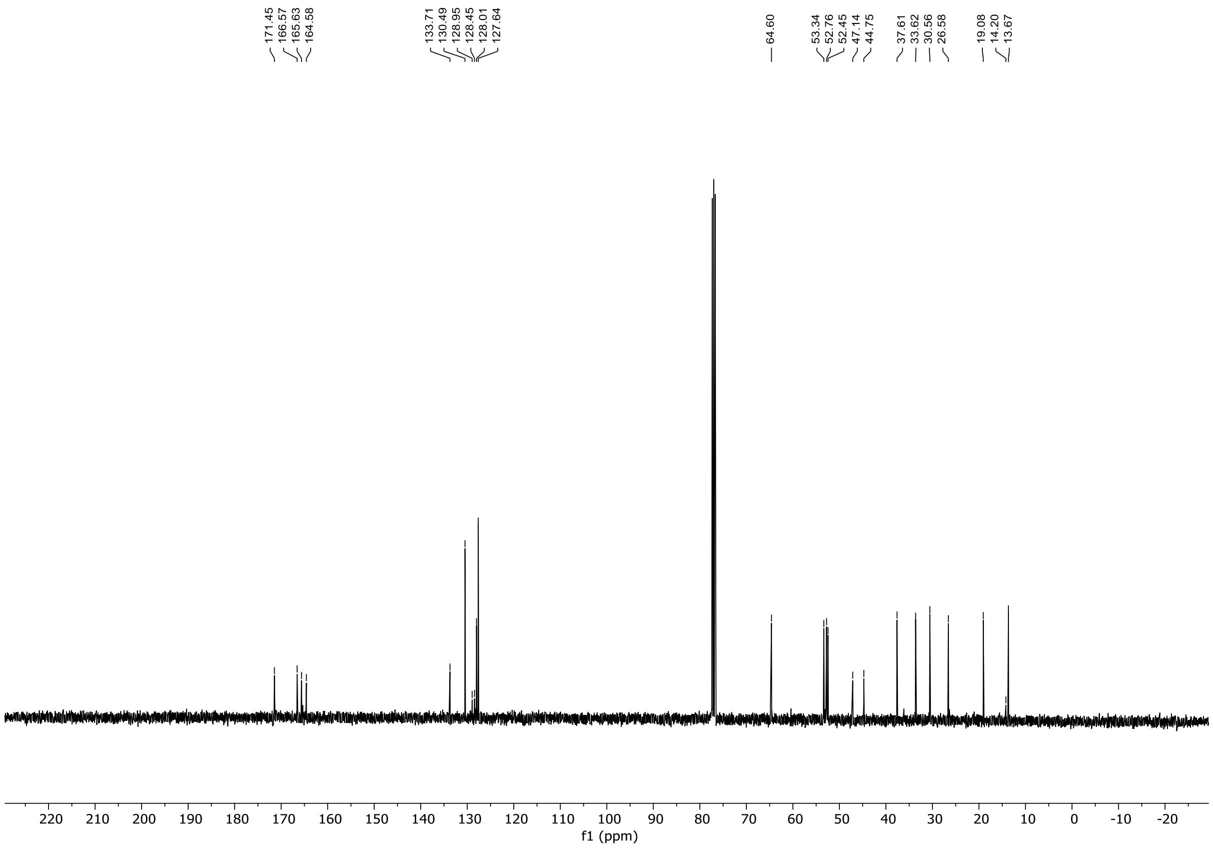

[back to Table of Contents]

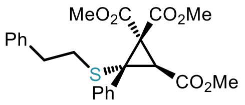

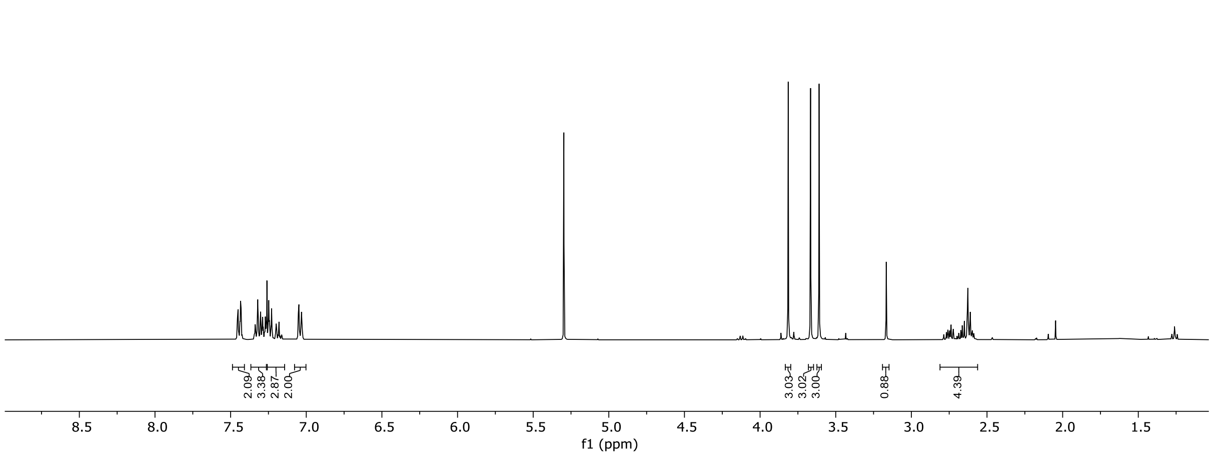

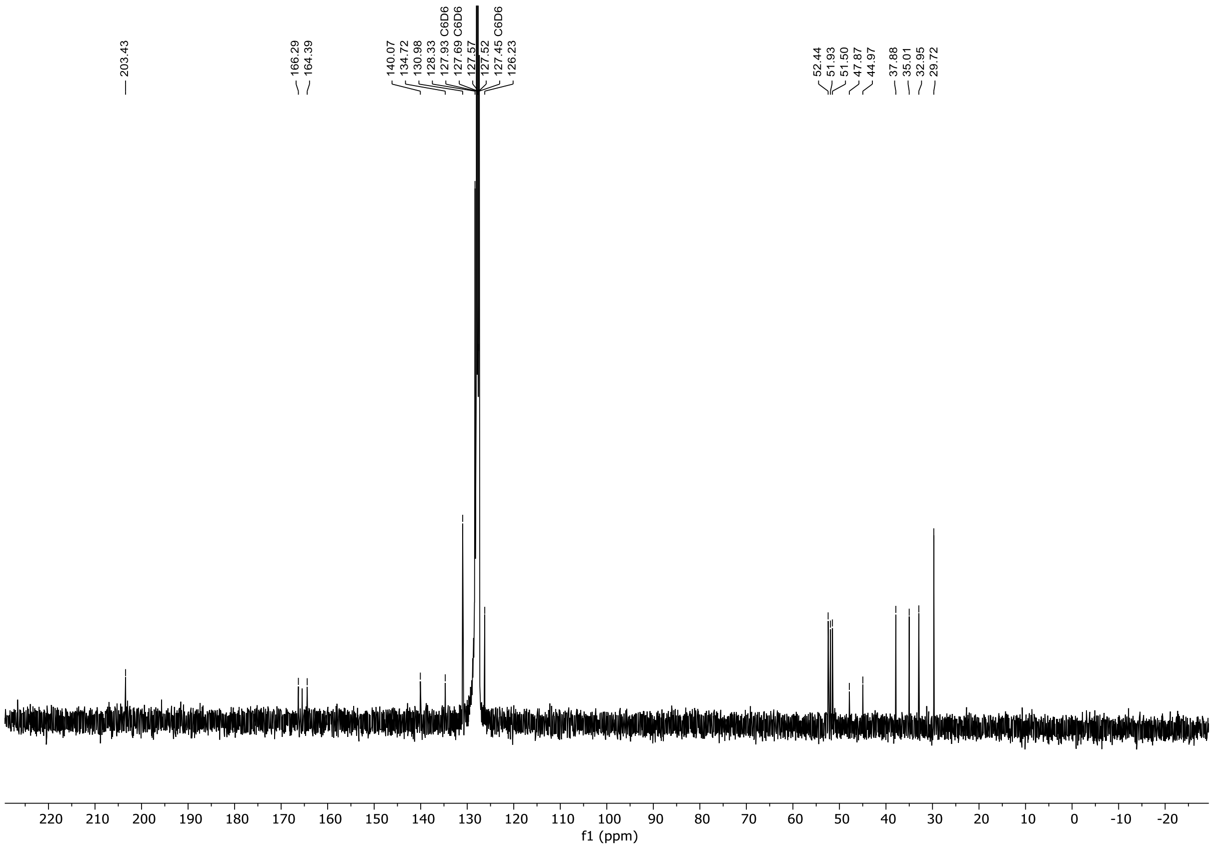

[back to Table of Contents]

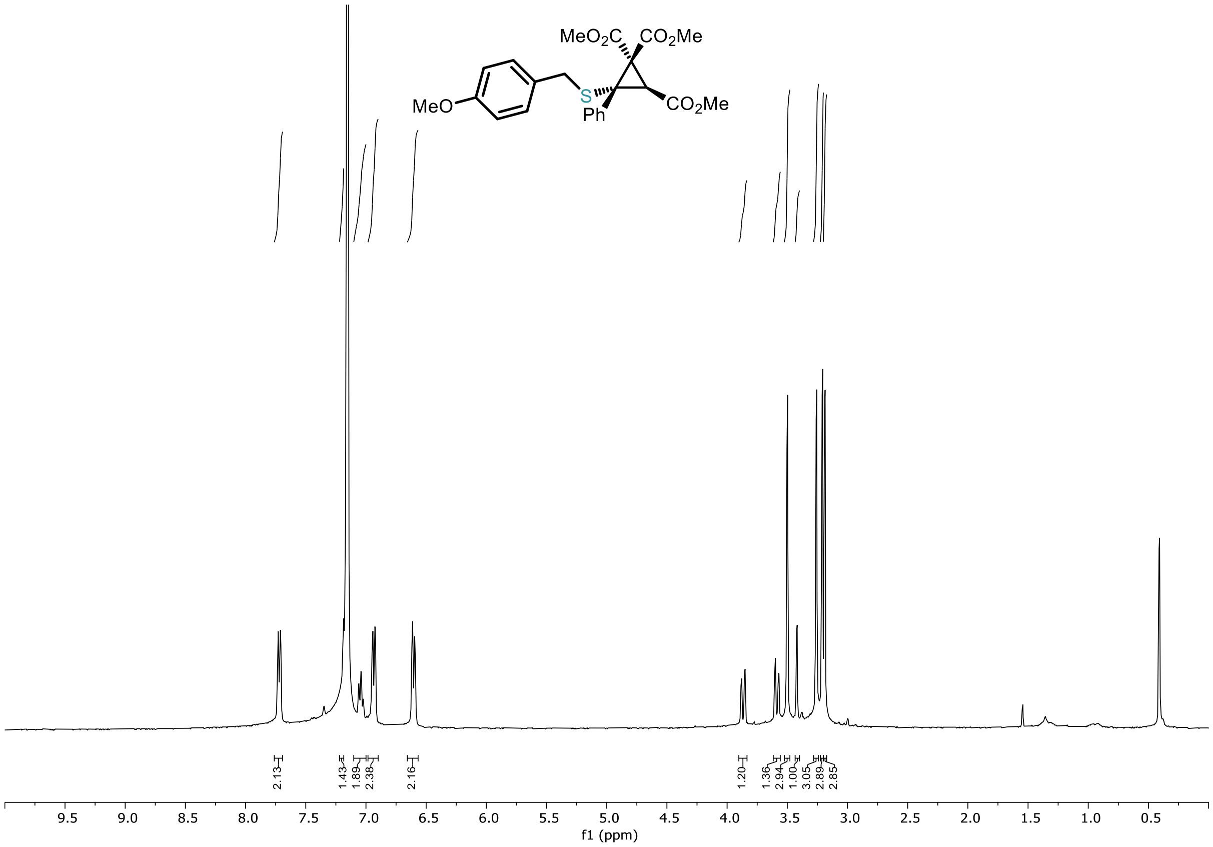

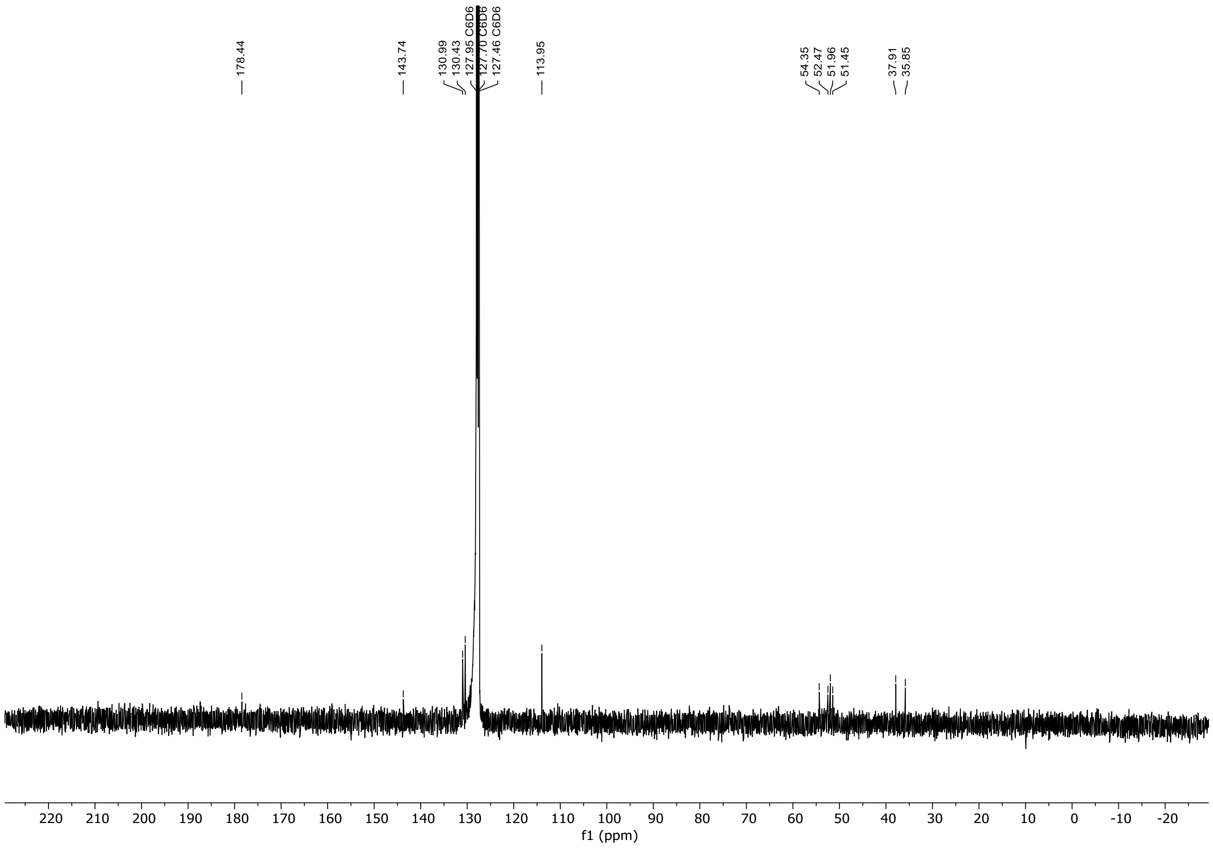

[back to Table of Contents]

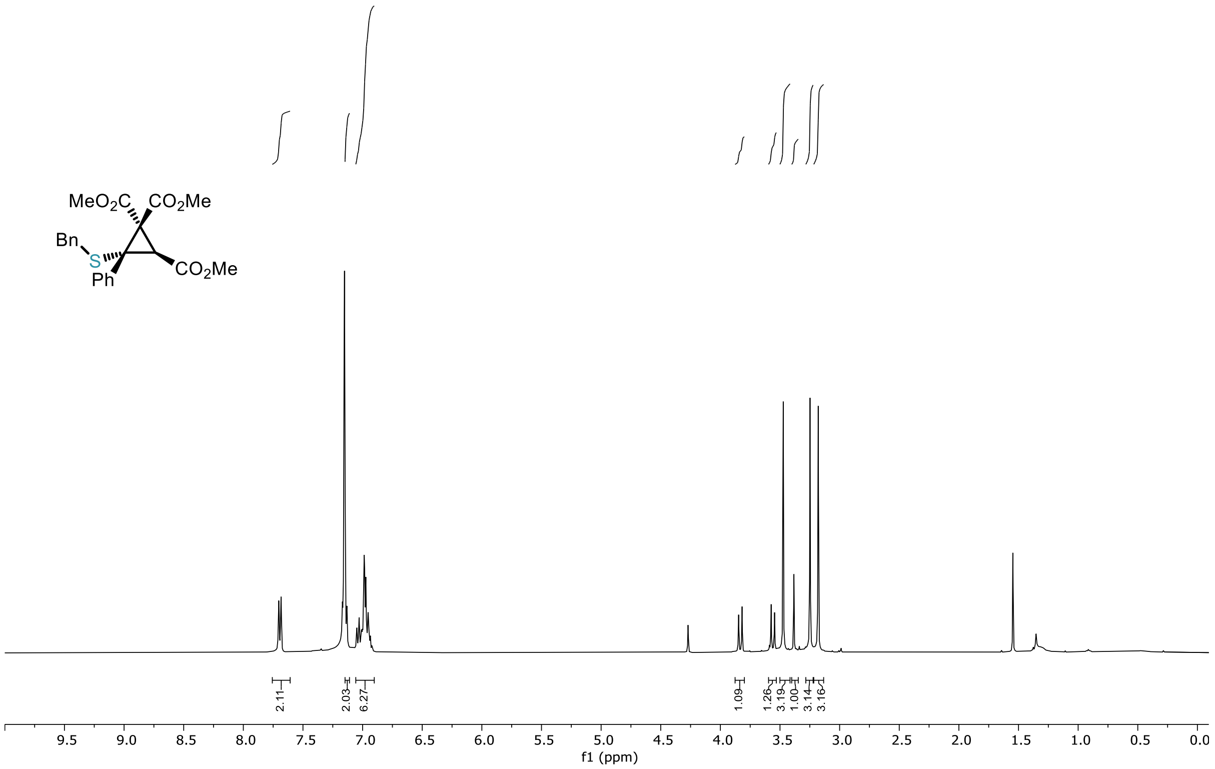

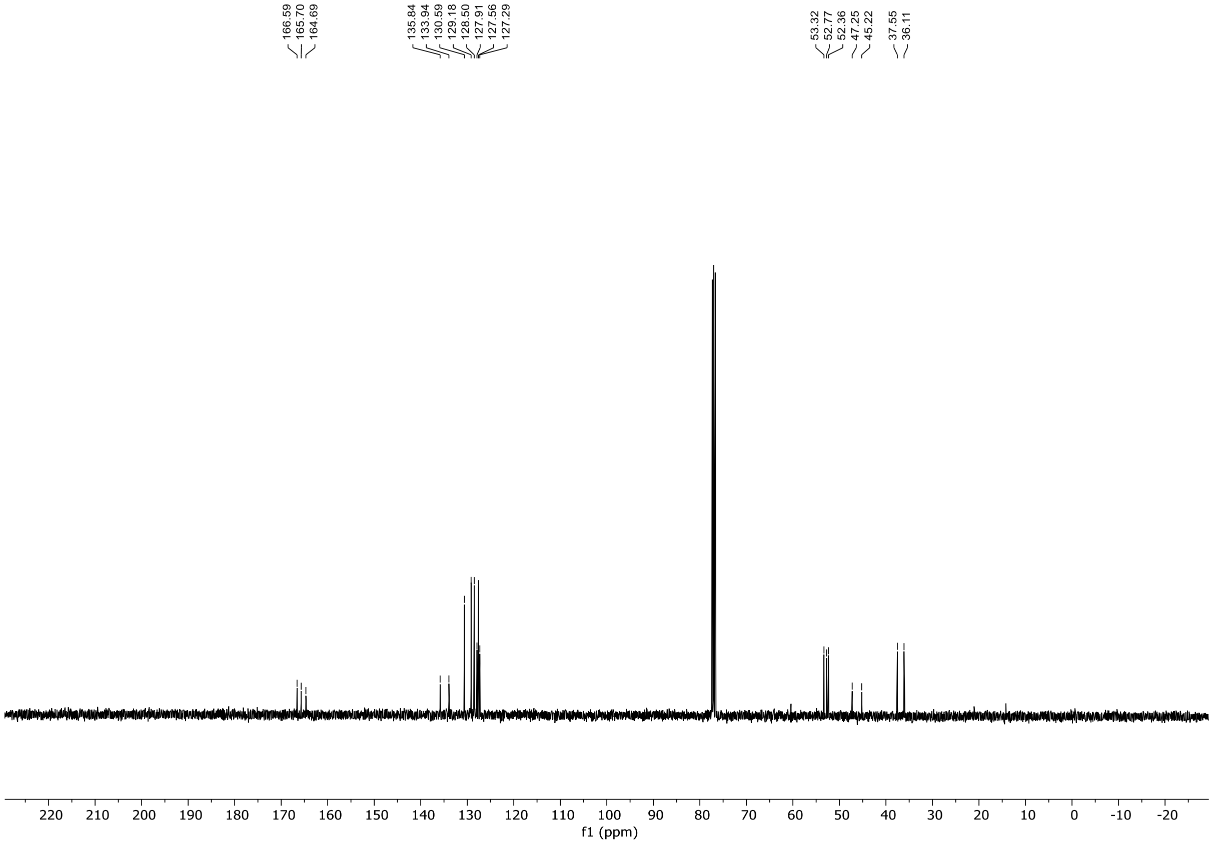

[back to Table of Contents]

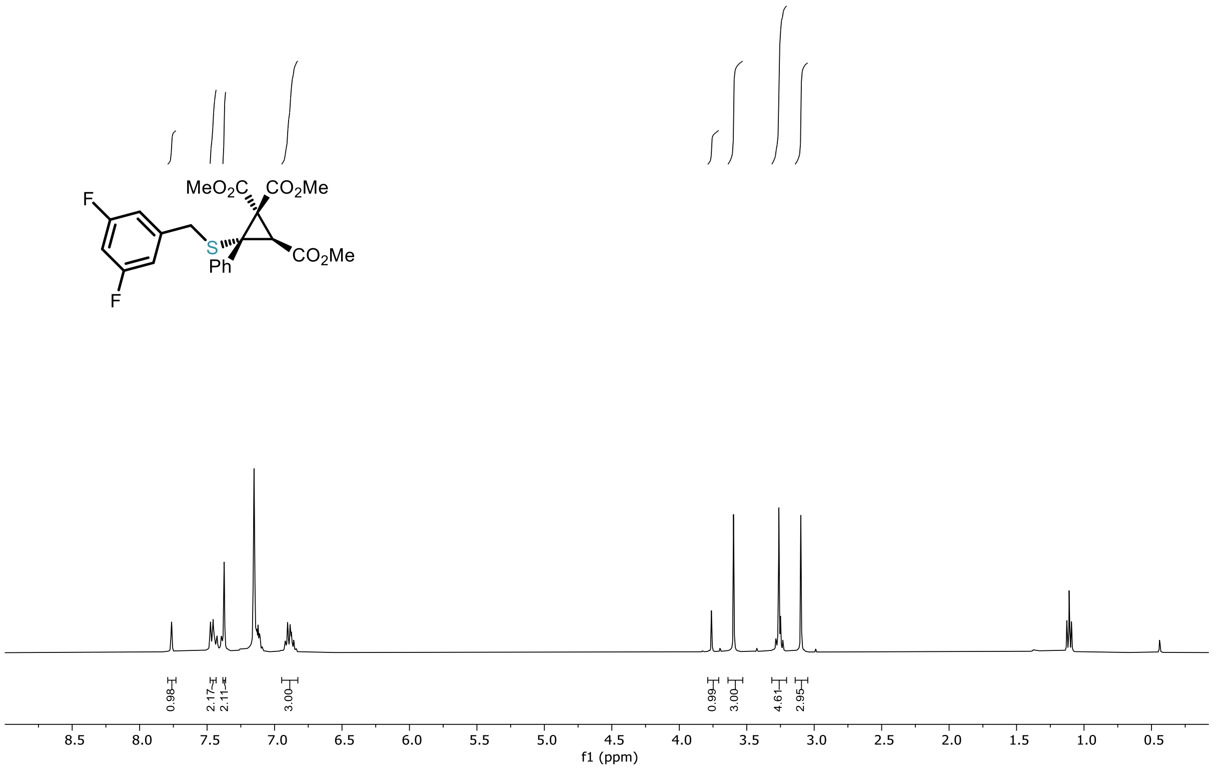

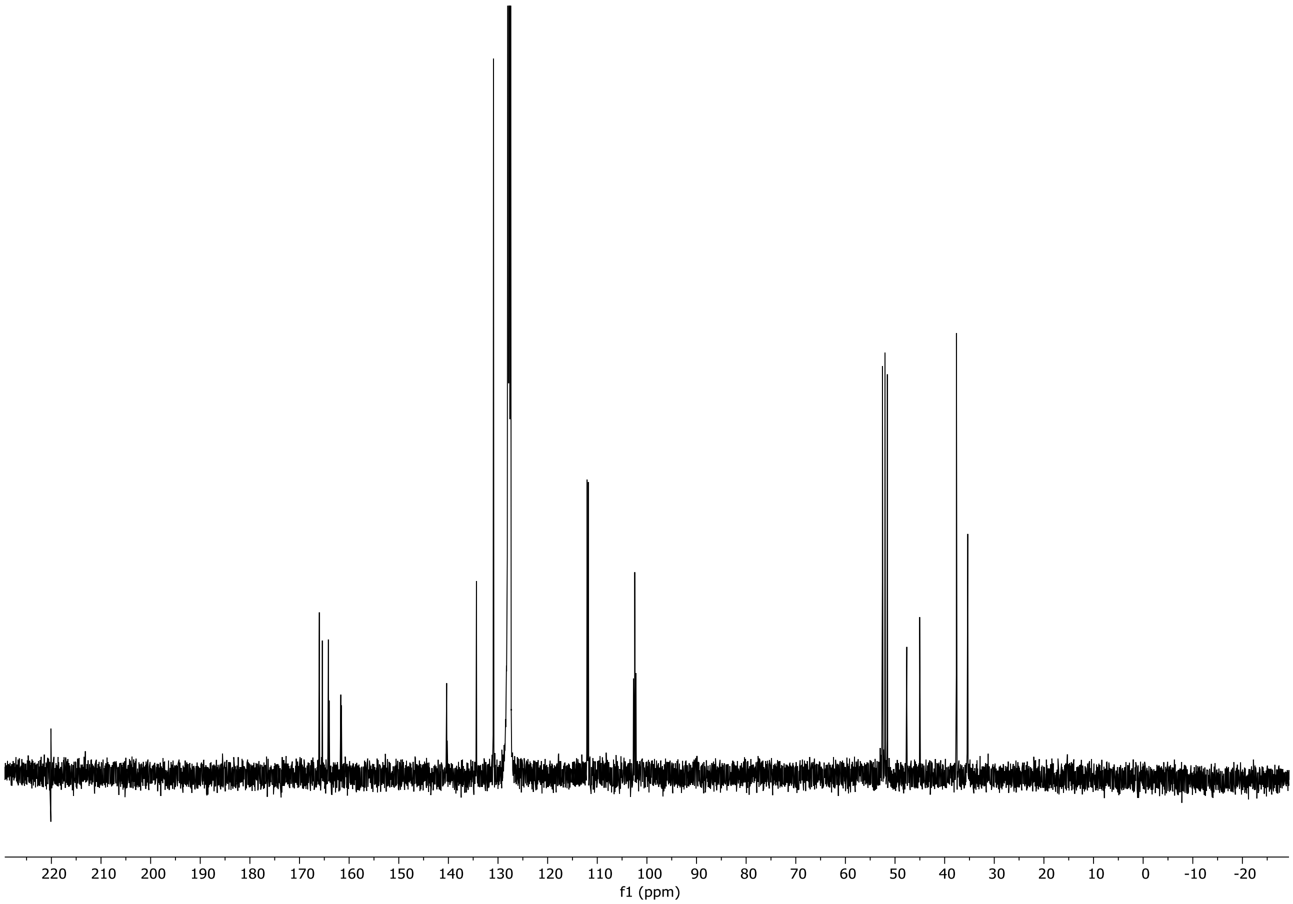

[back to Table of Contents]

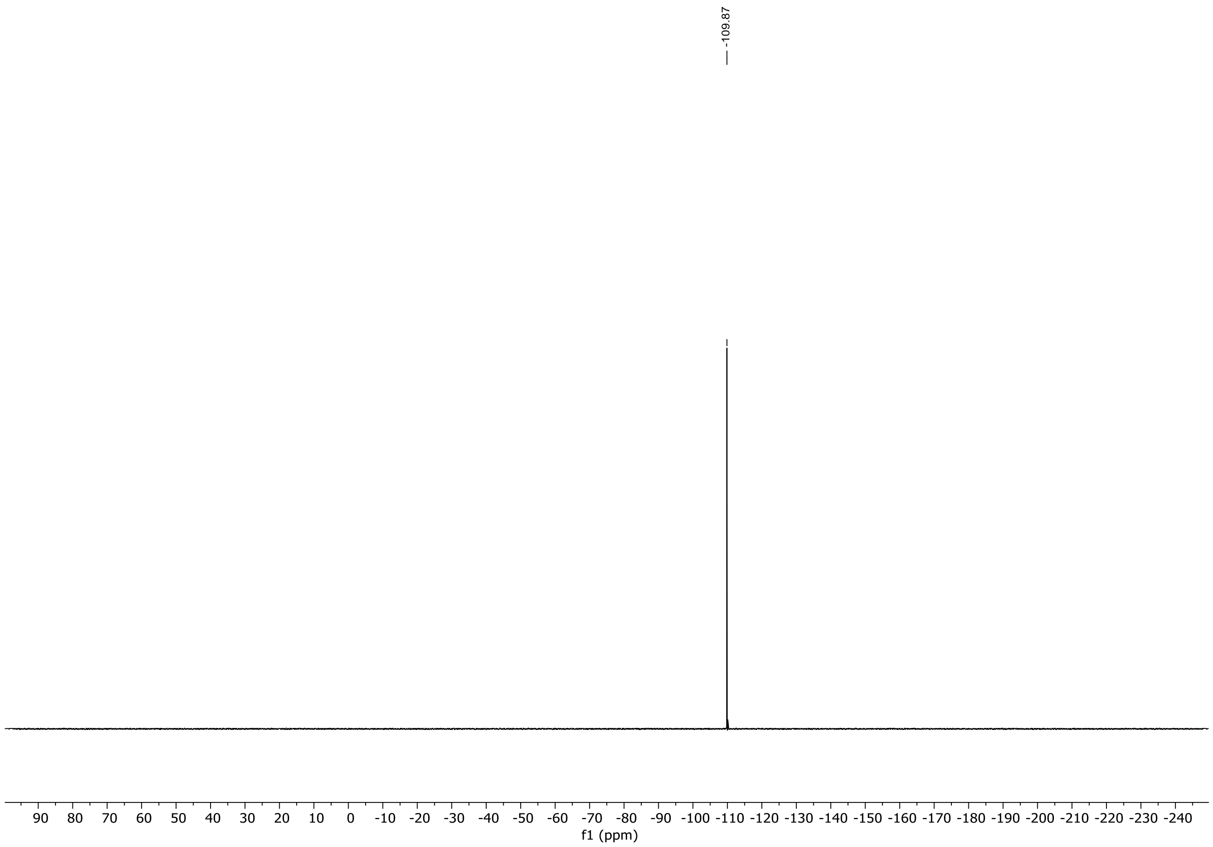

[back to Table of Contents]

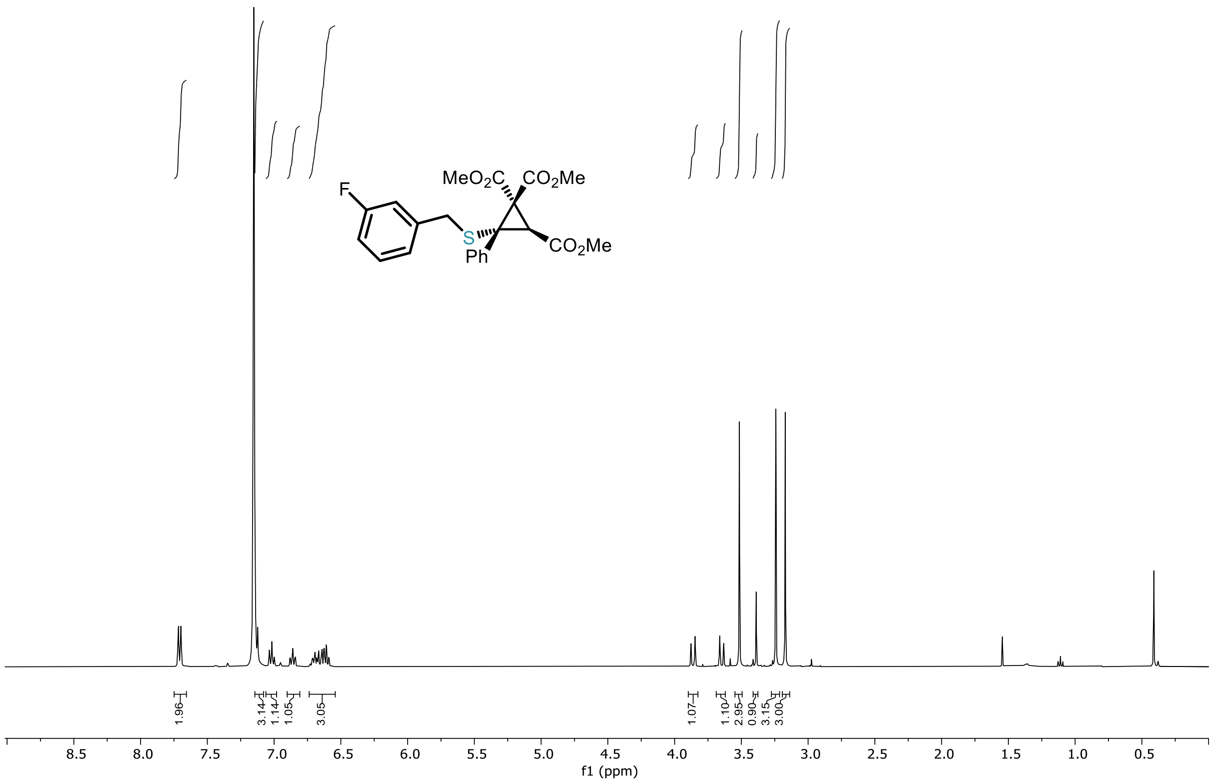

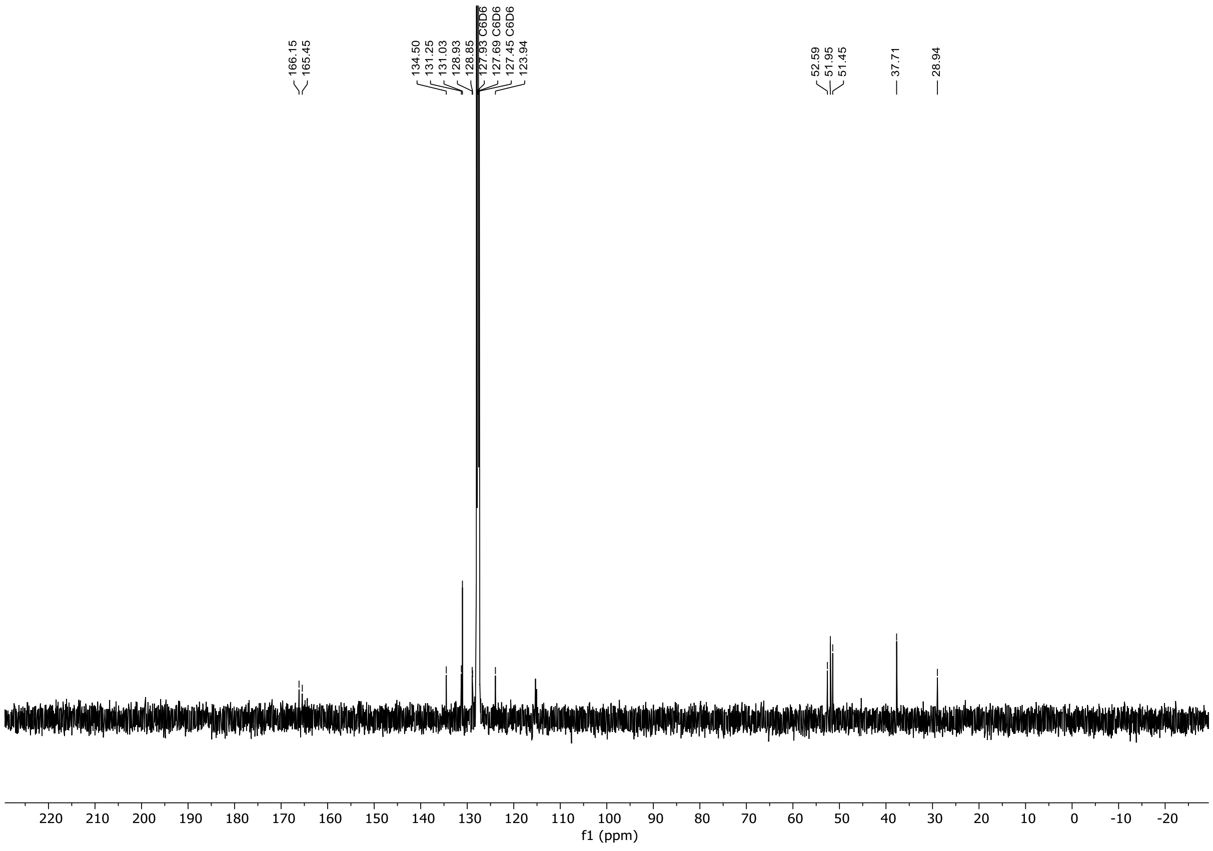

[back to Table of Contents]

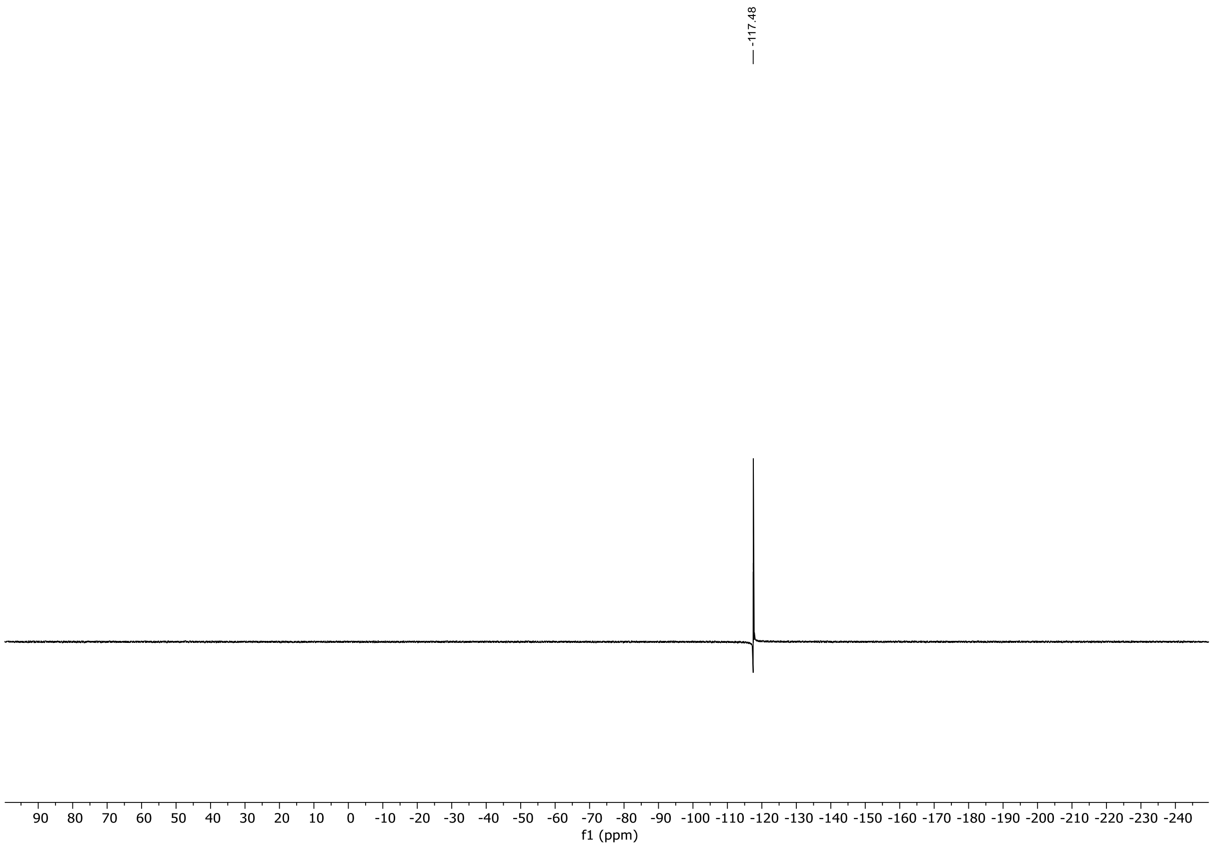

[back to Table of Contents]

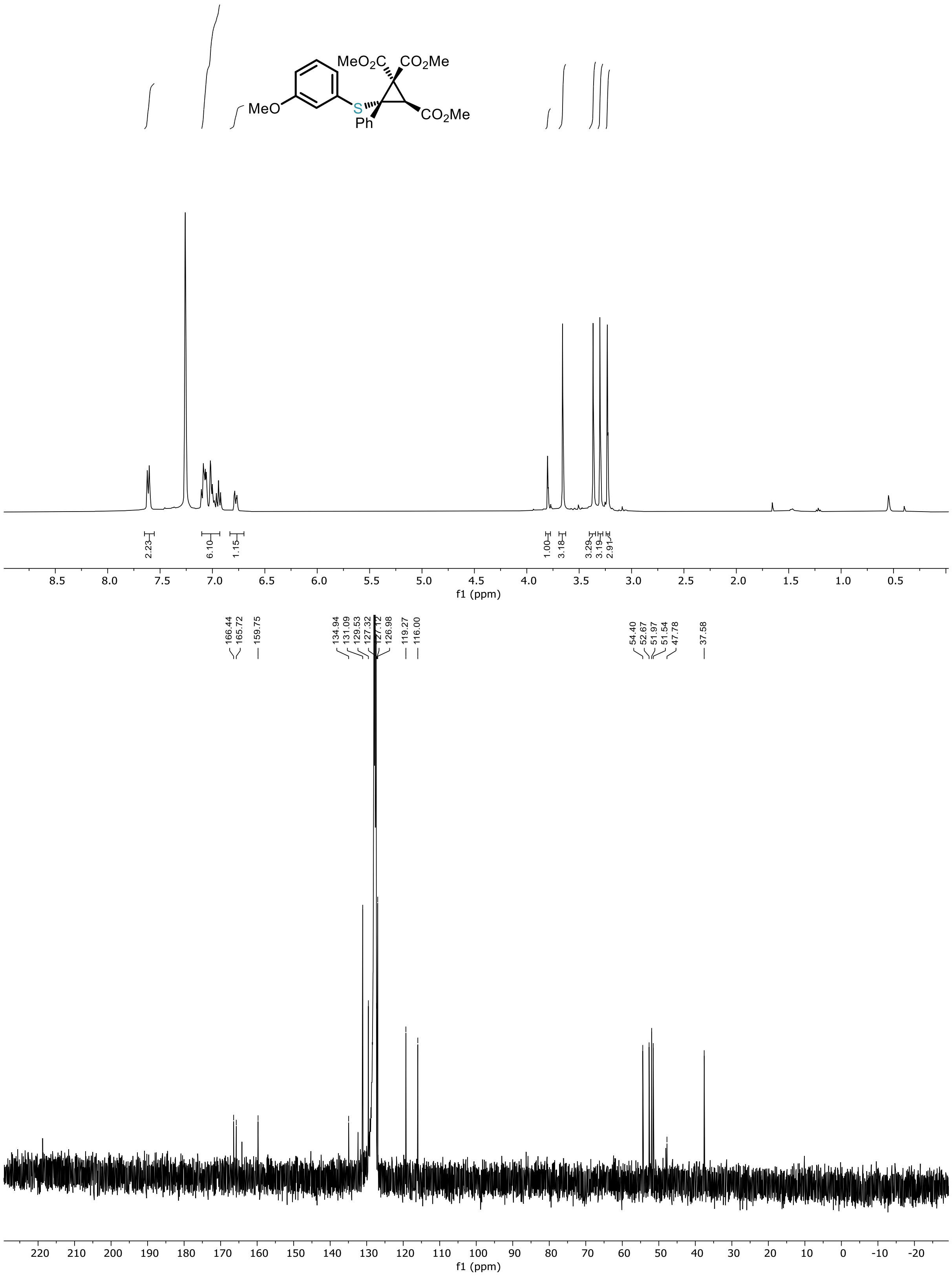

[back to Table of Contents]

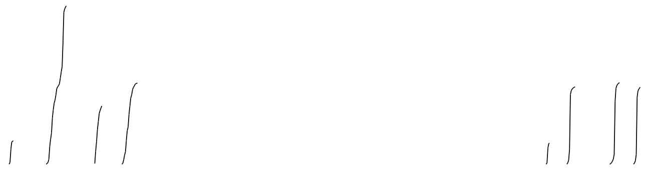

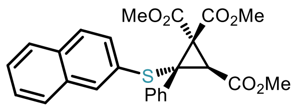

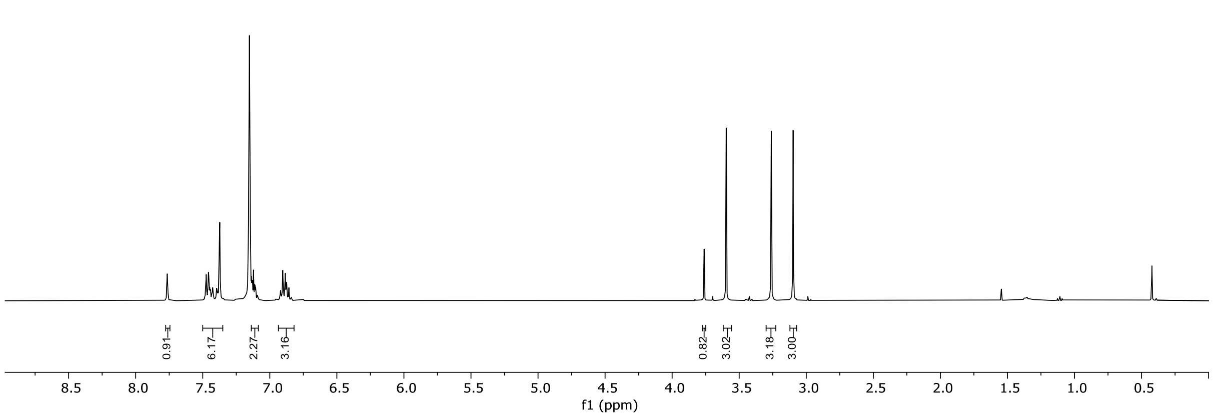

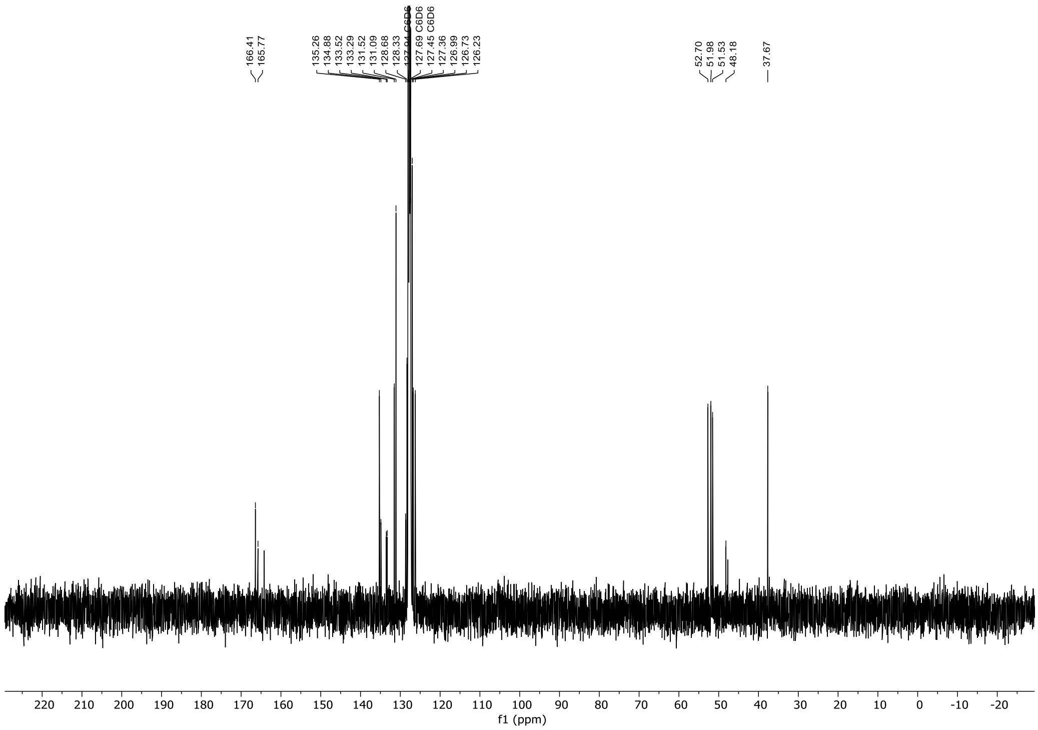

[back to Table of Contents]

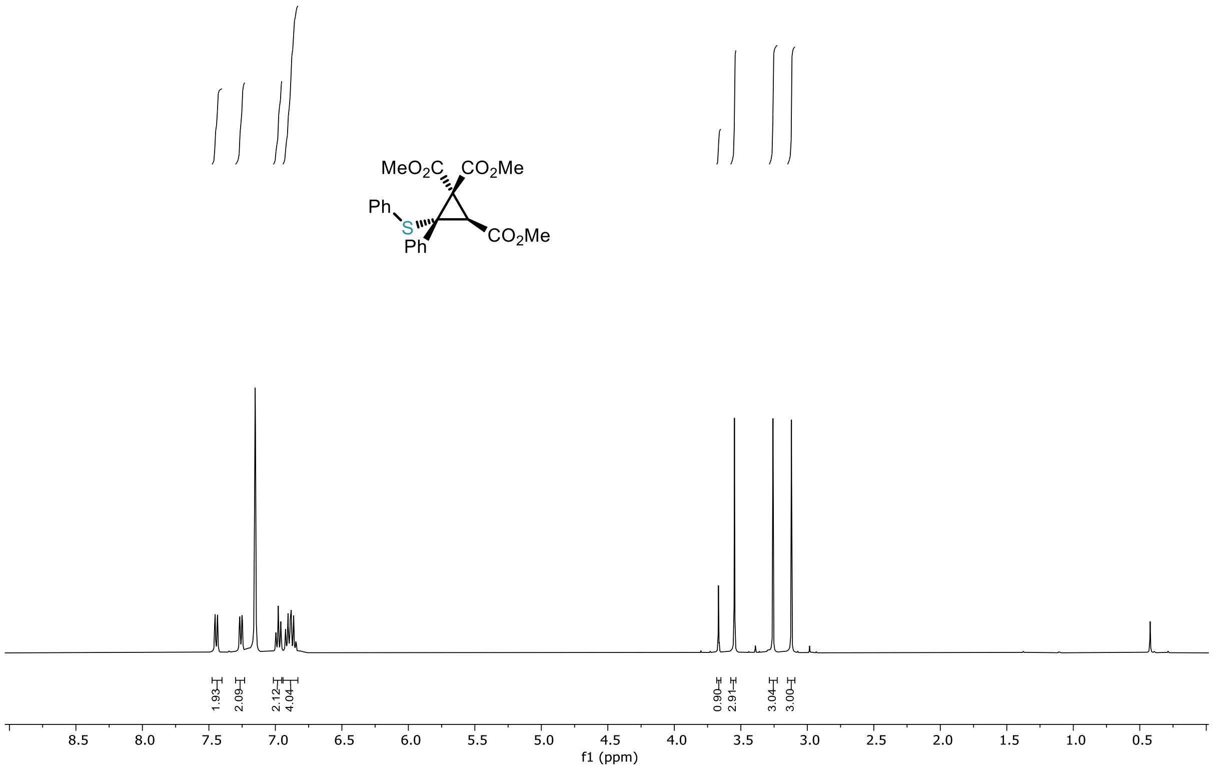

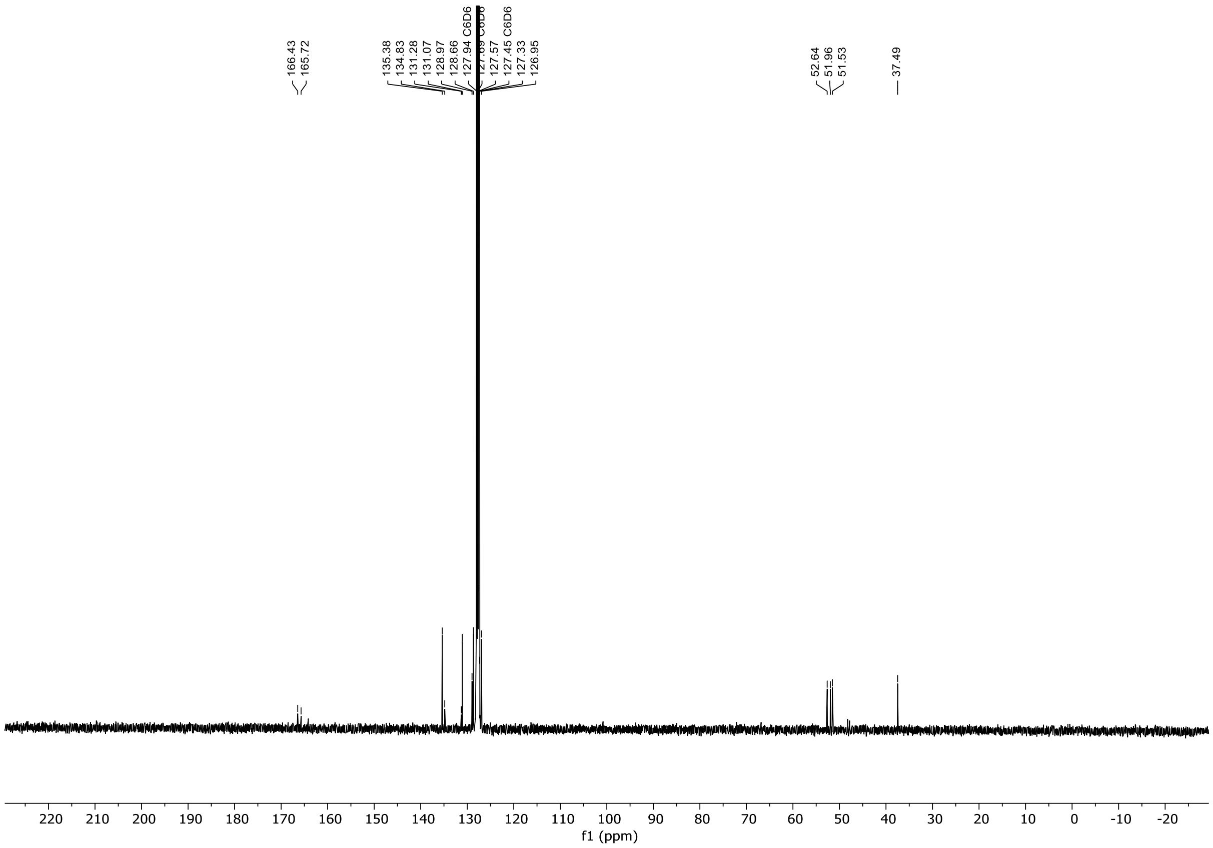

[back to Table of Contents]

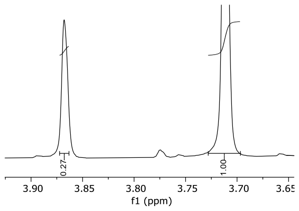

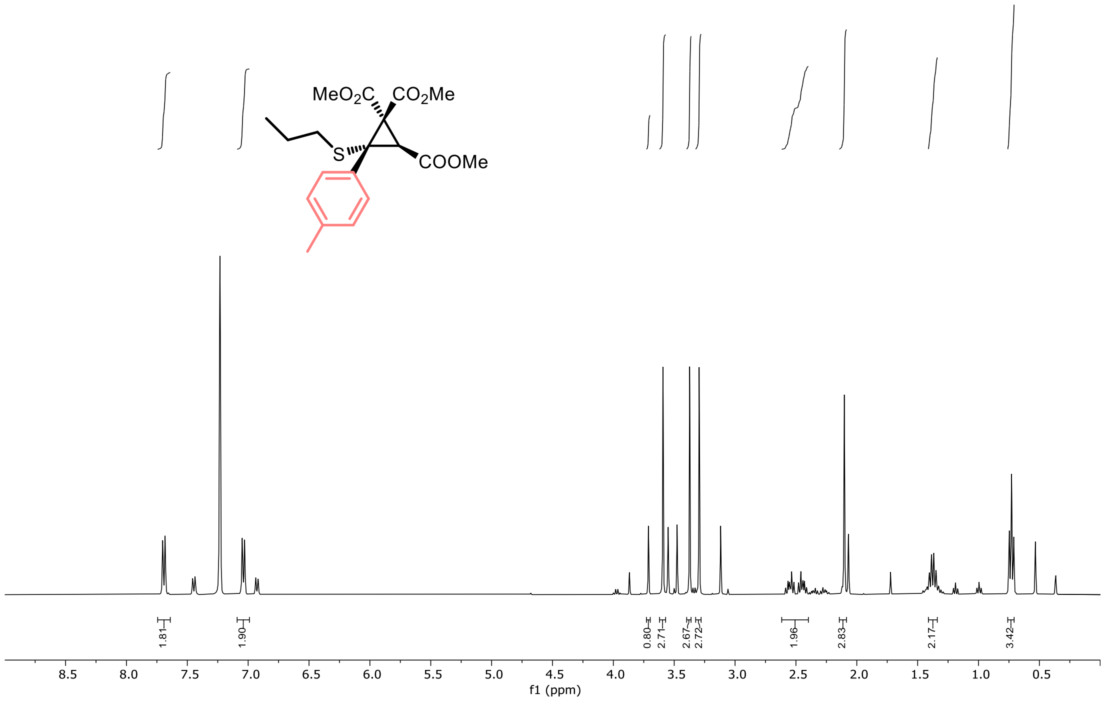

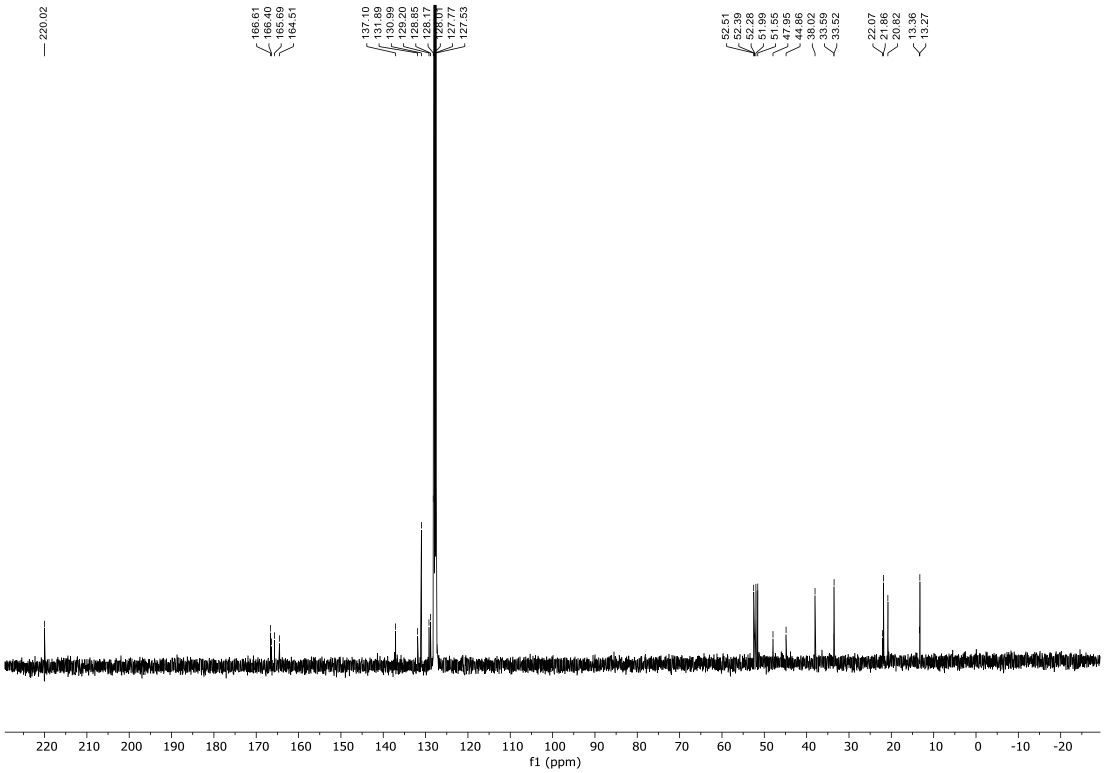

[back to Table of Contents]

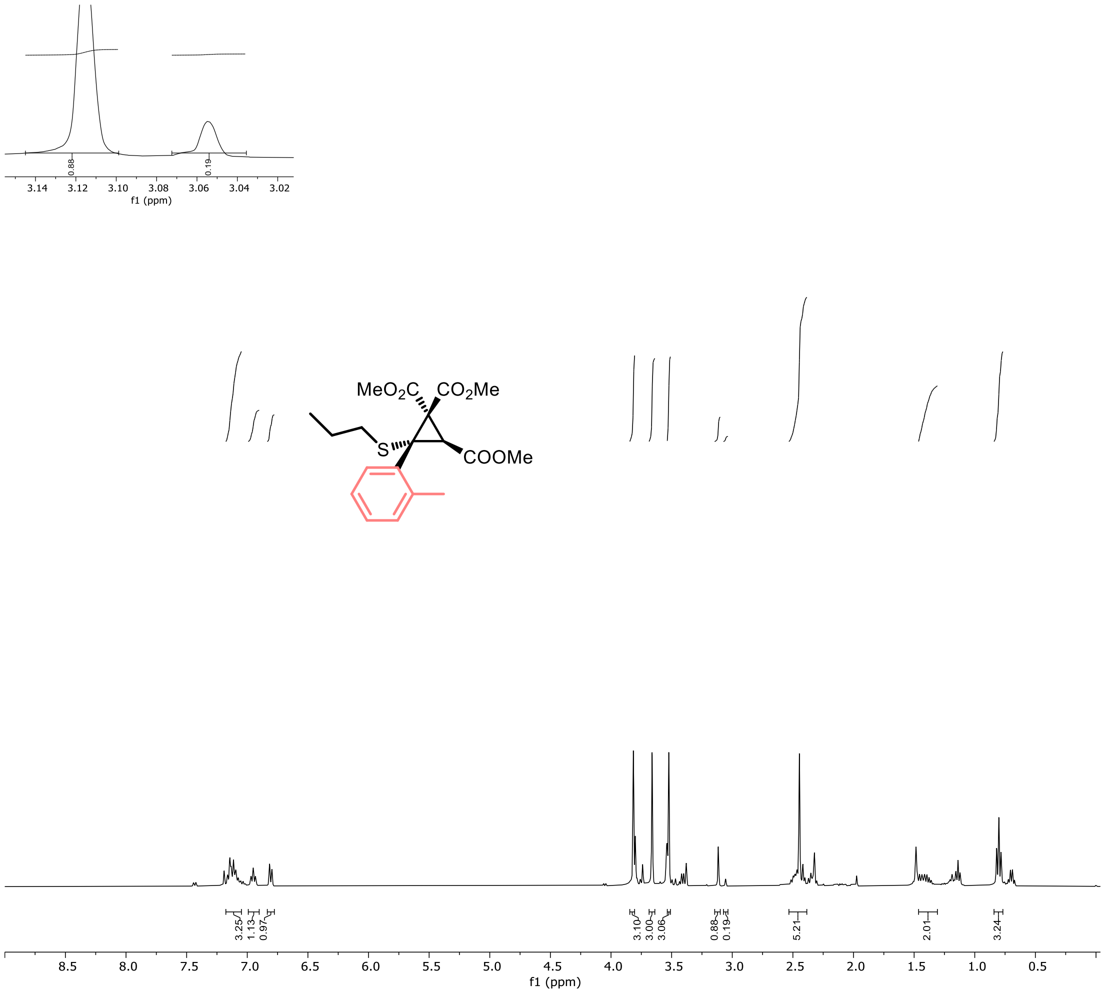

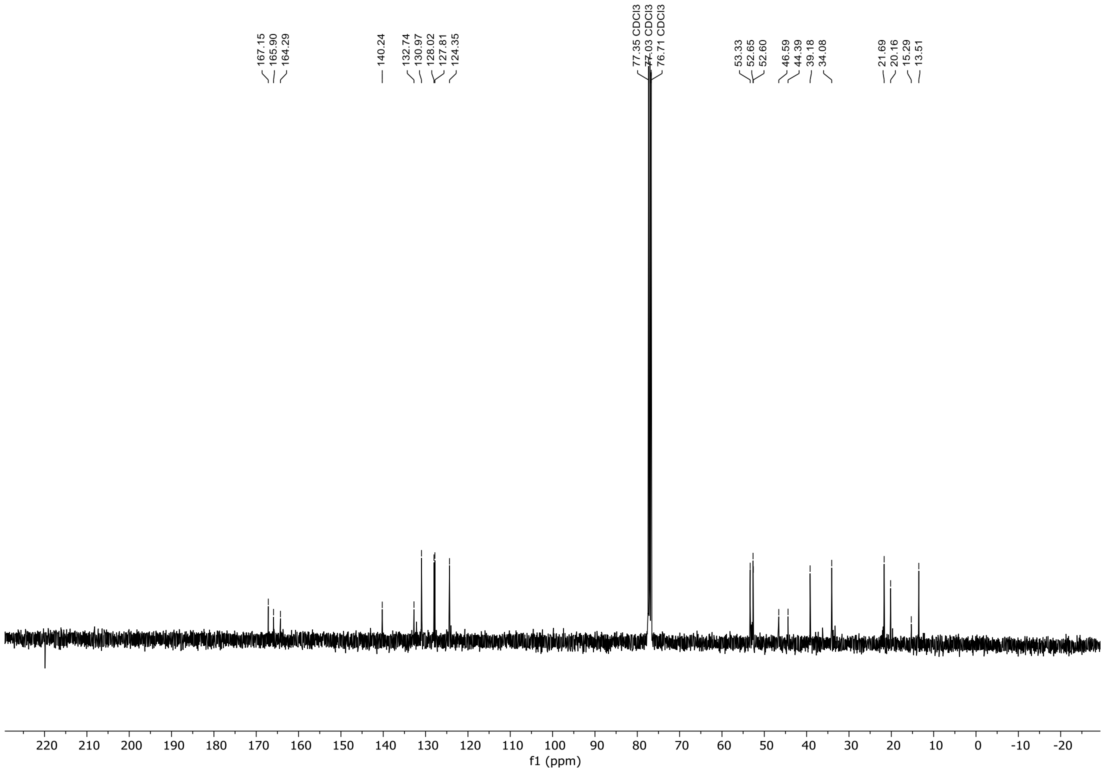

[back to Table of Contents]

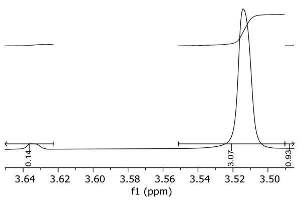

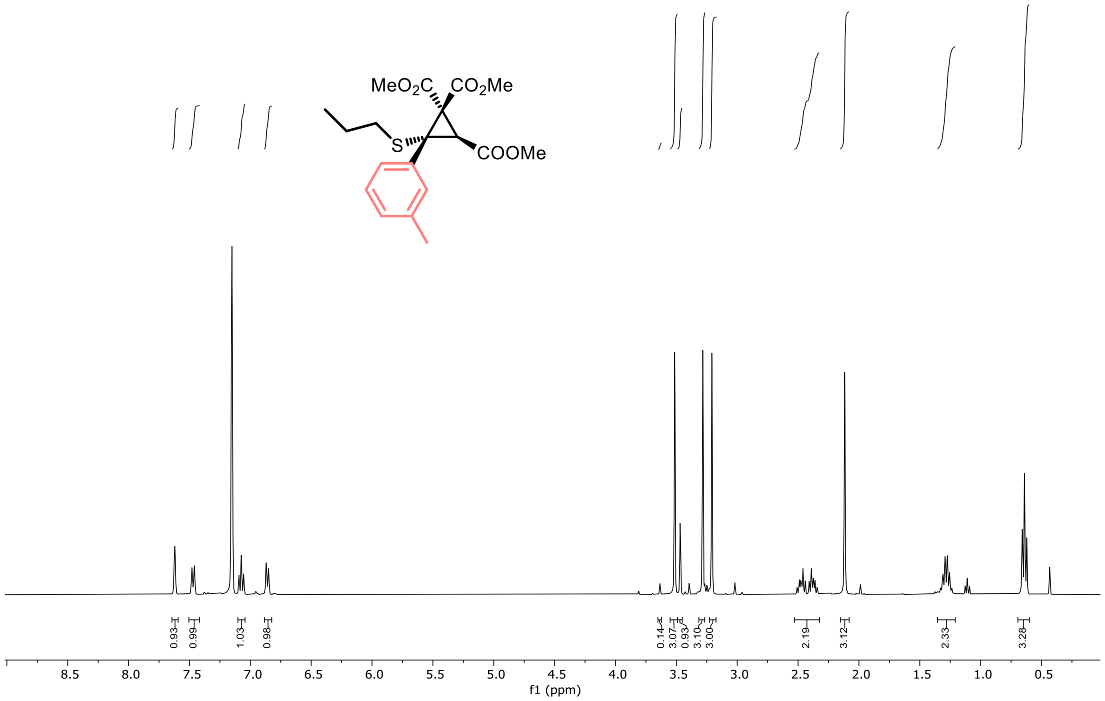

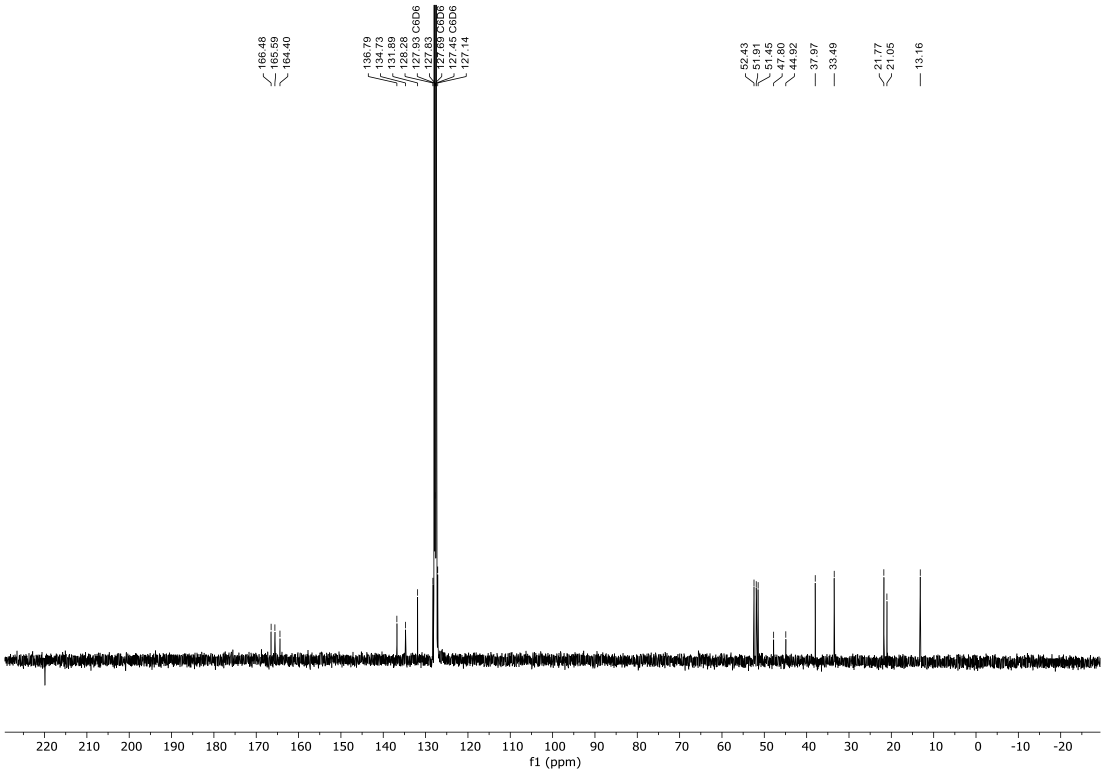

[back to Table of Contents]

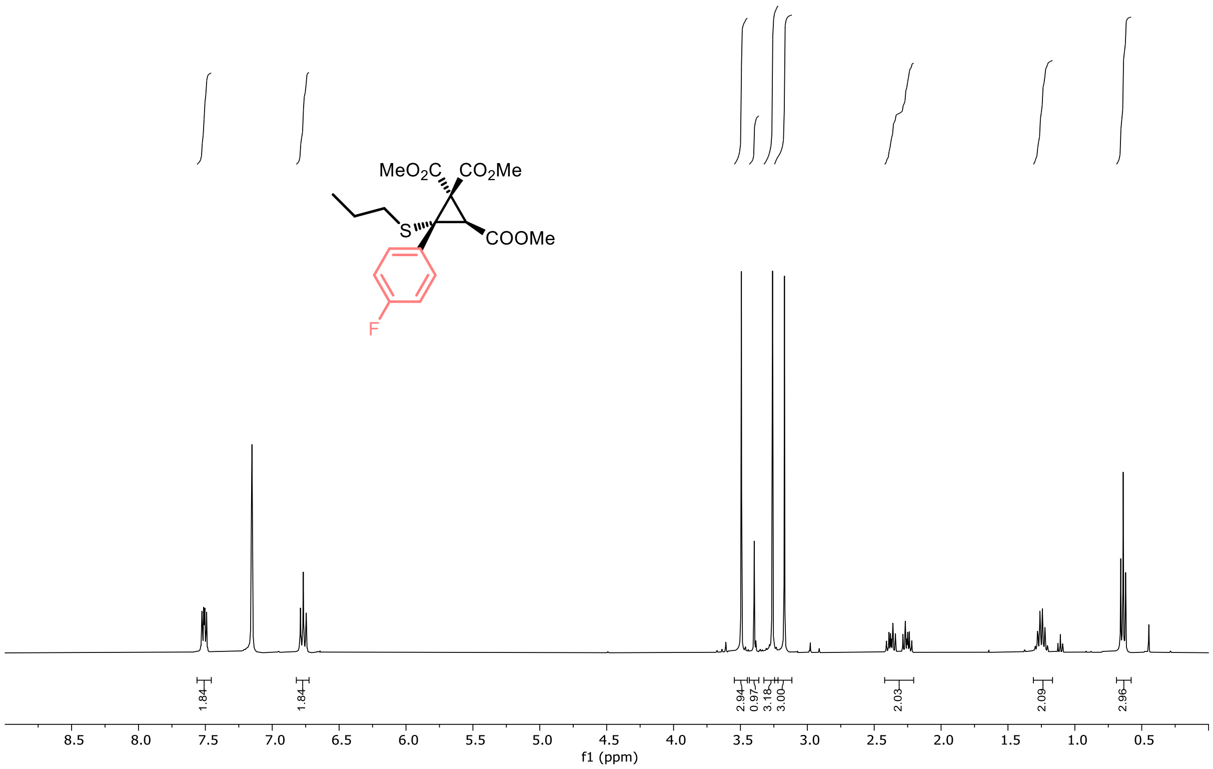

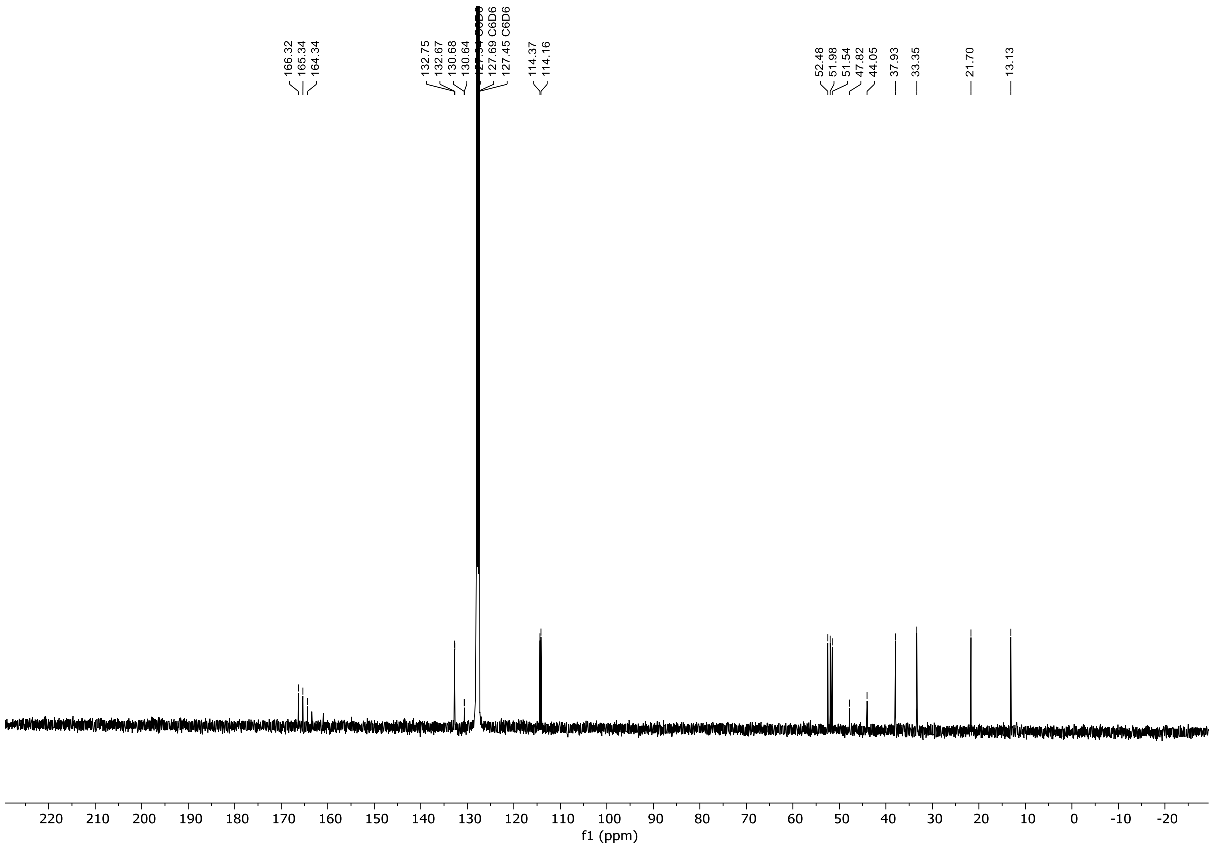

[back to Table of Contents]

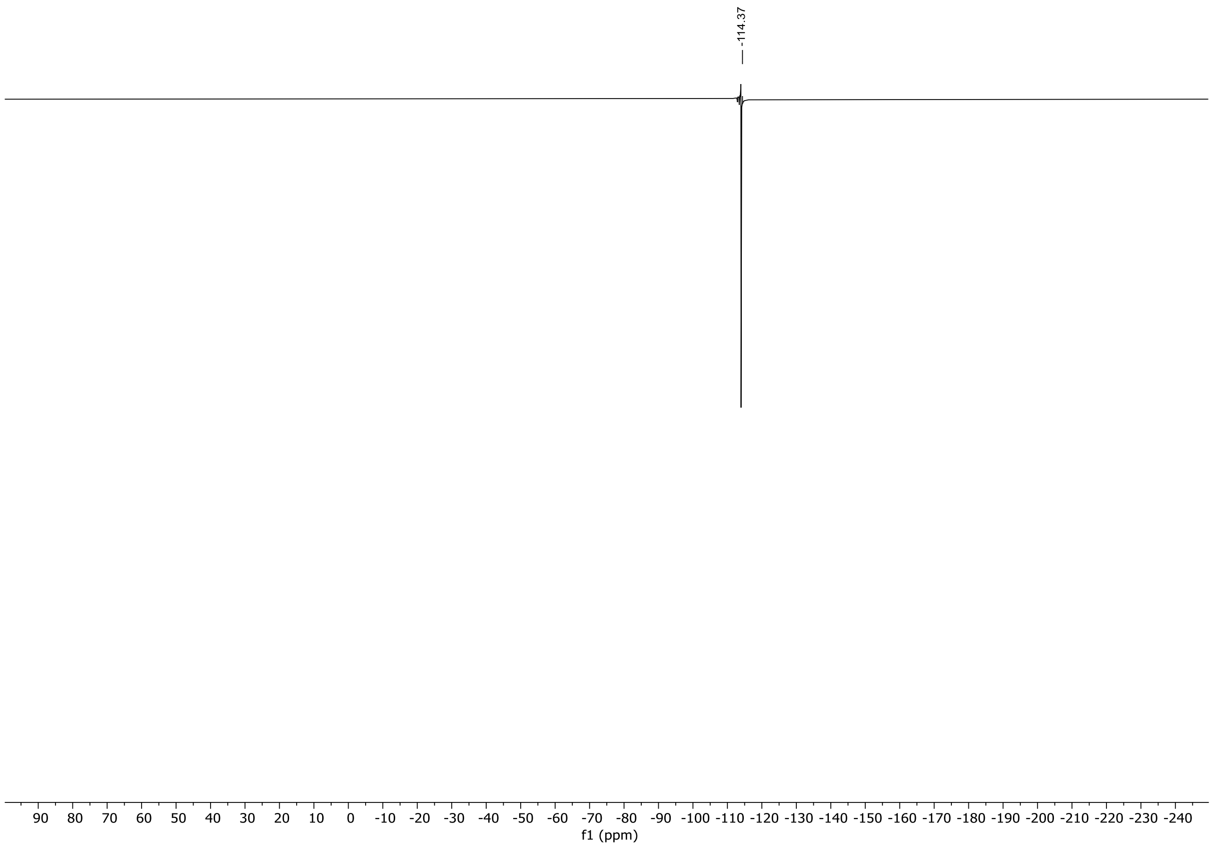

[back to Table of Contents]

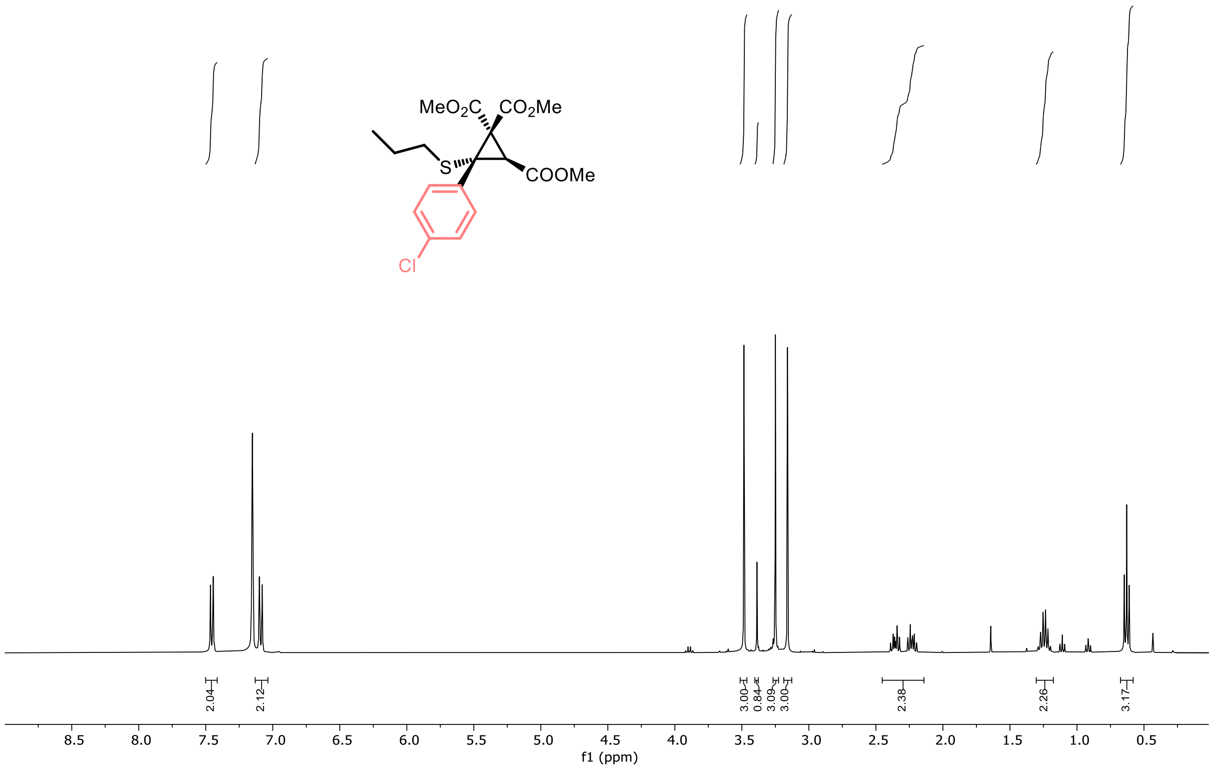

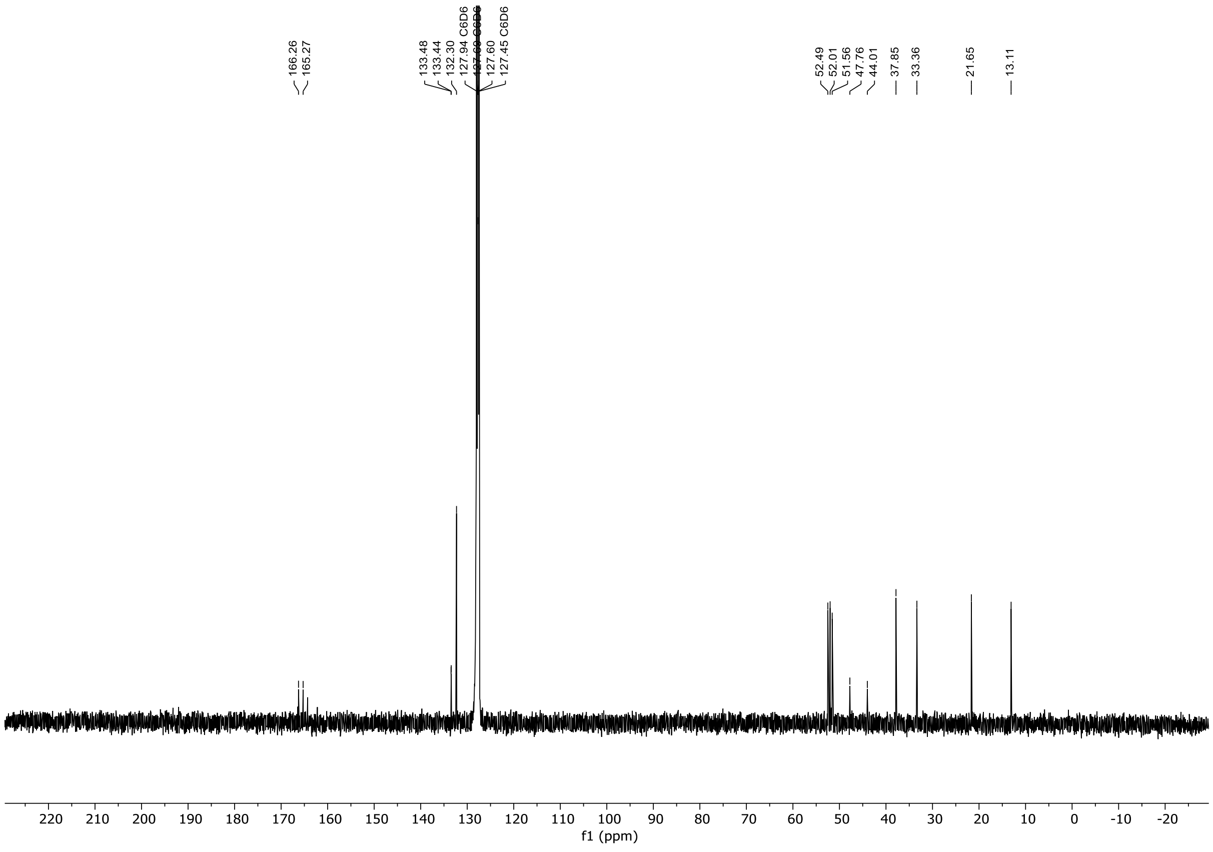

[back to Table of Contents]

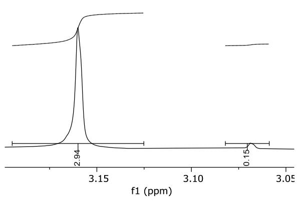

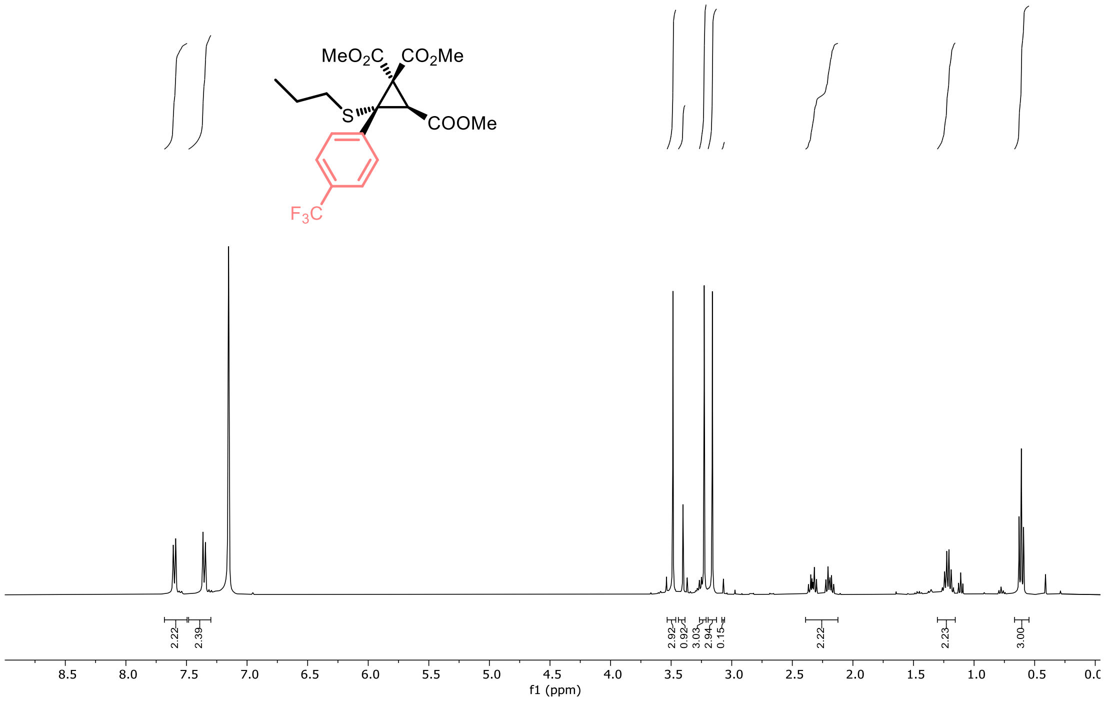

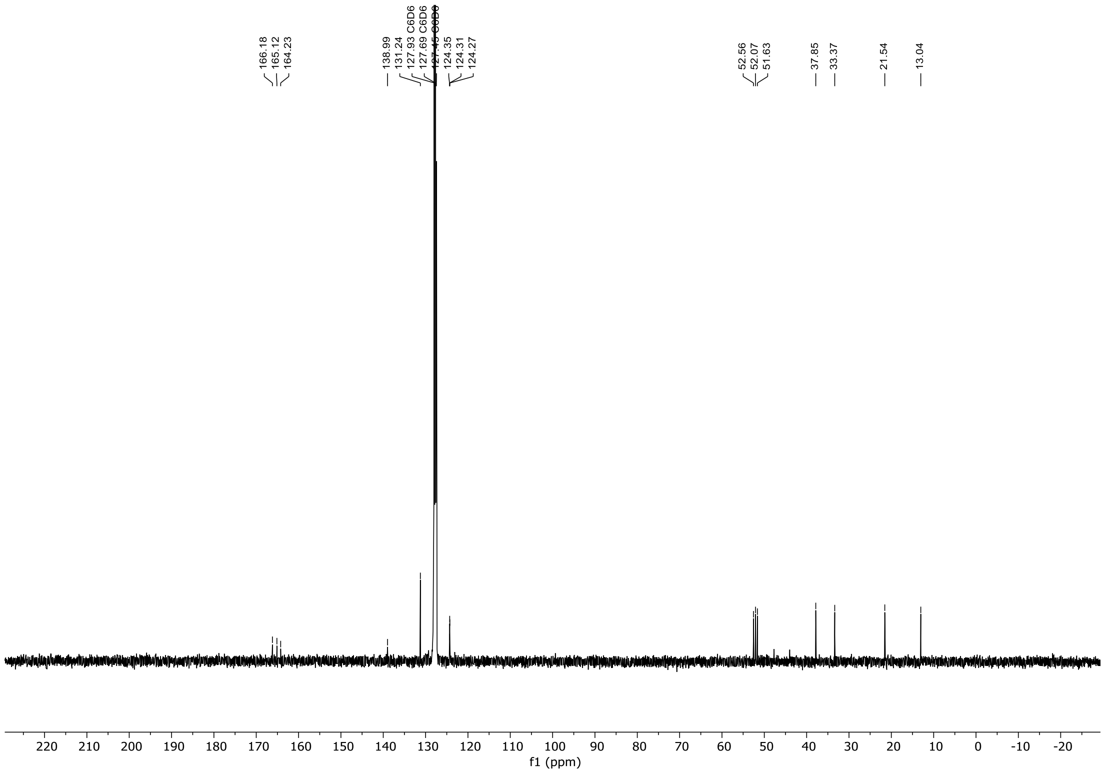

[back to Table of Contents]

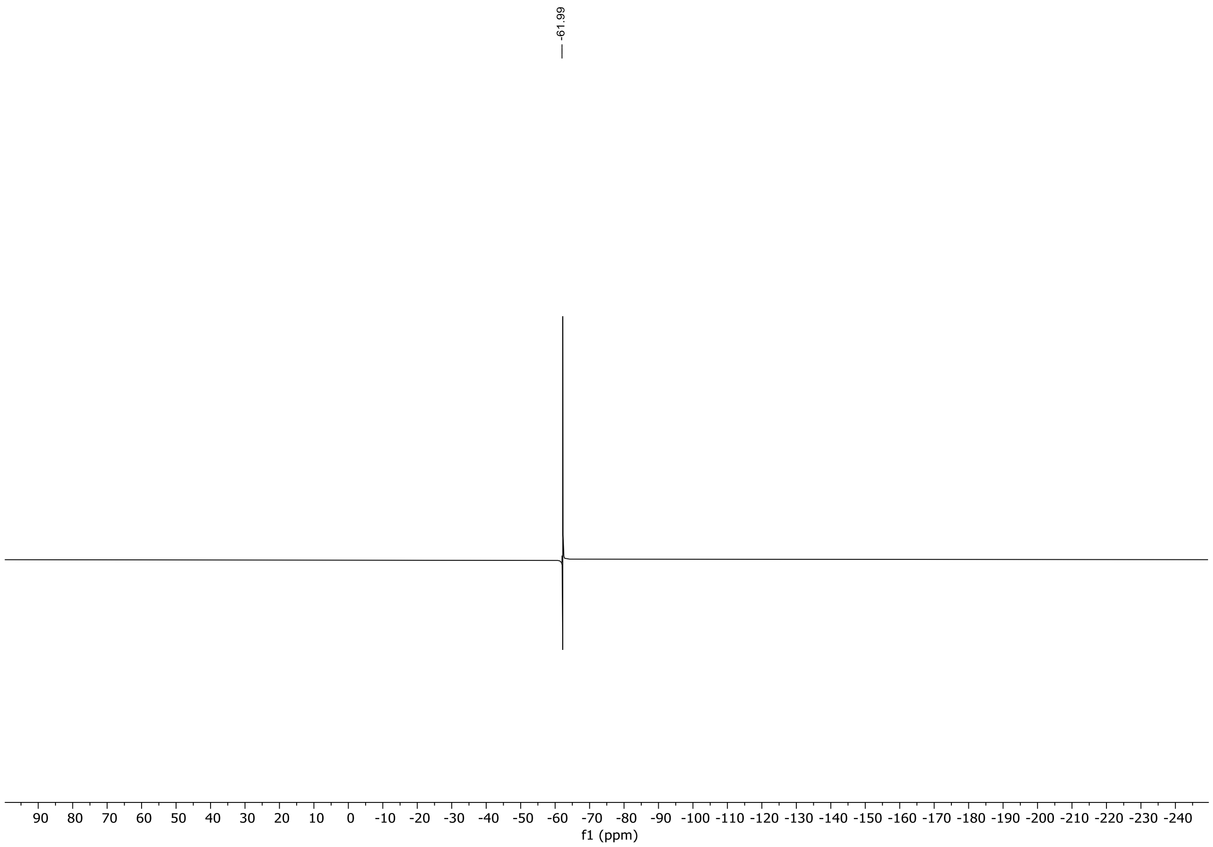

[back to Table of Contents]

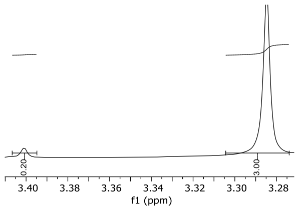

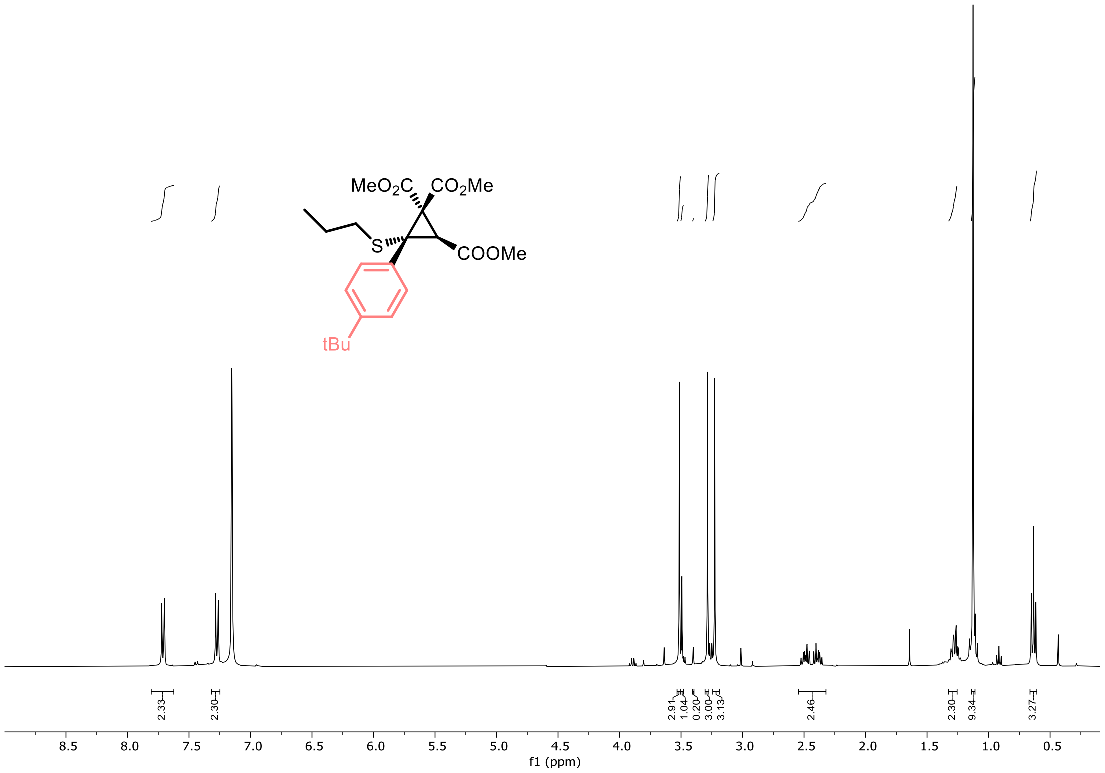

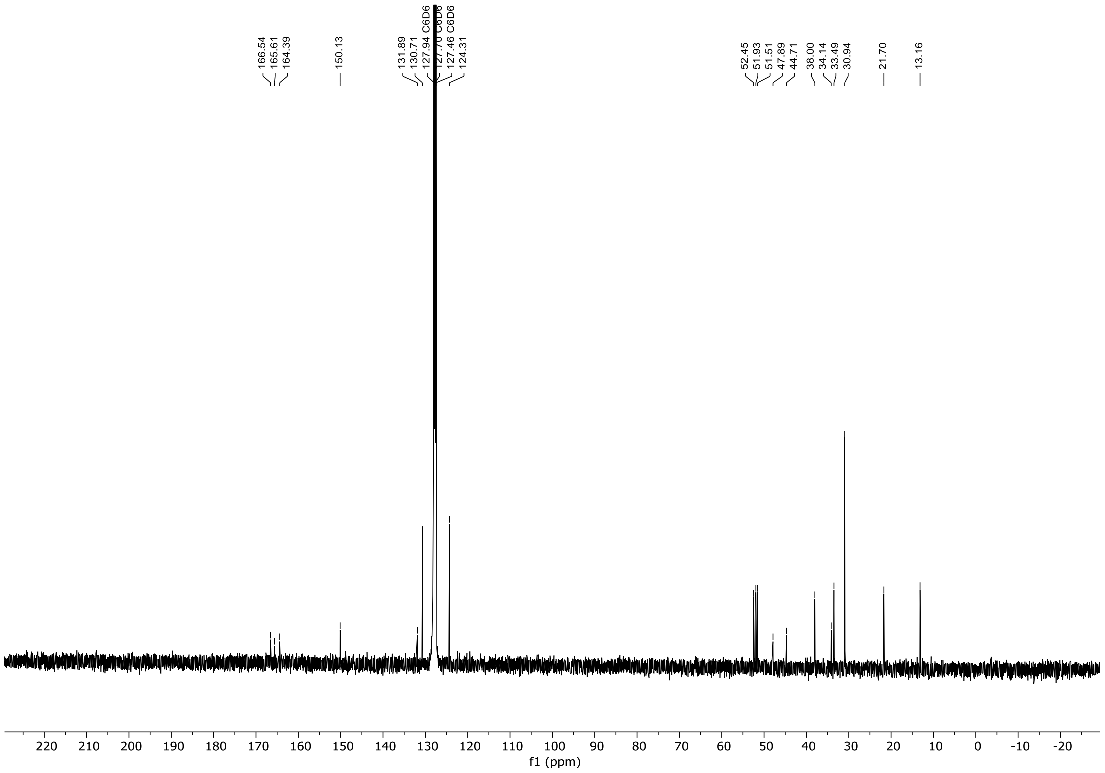

[back to Table of Contents]

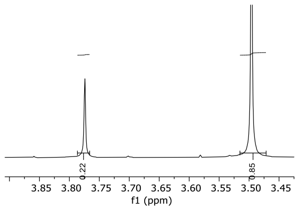

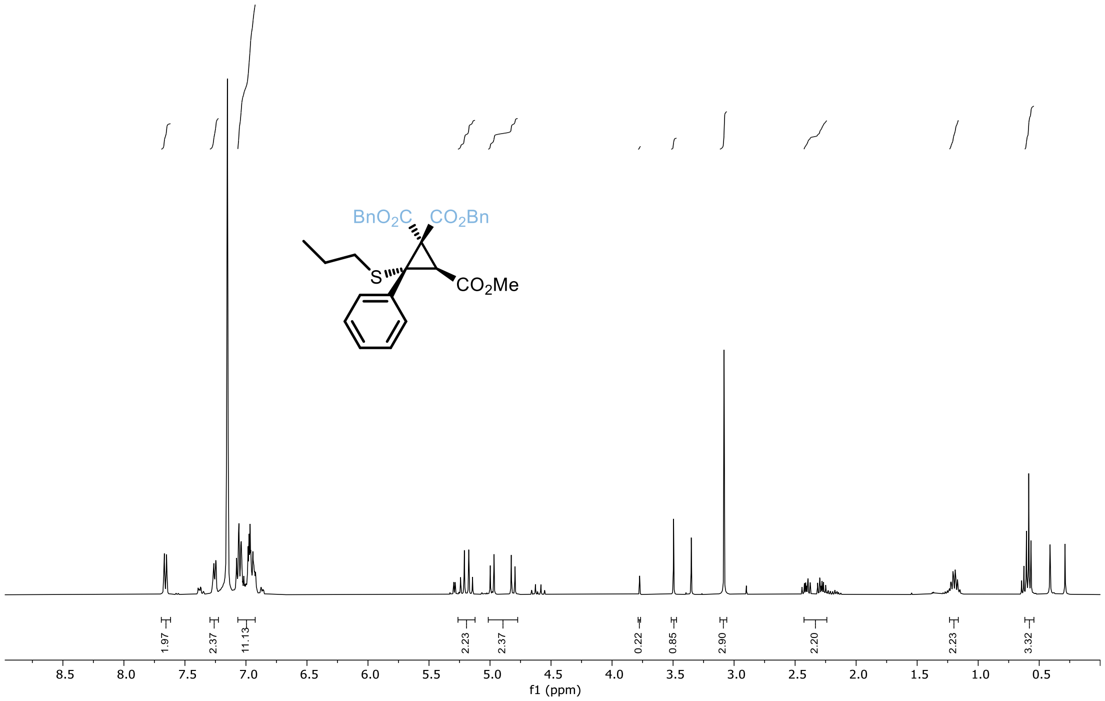

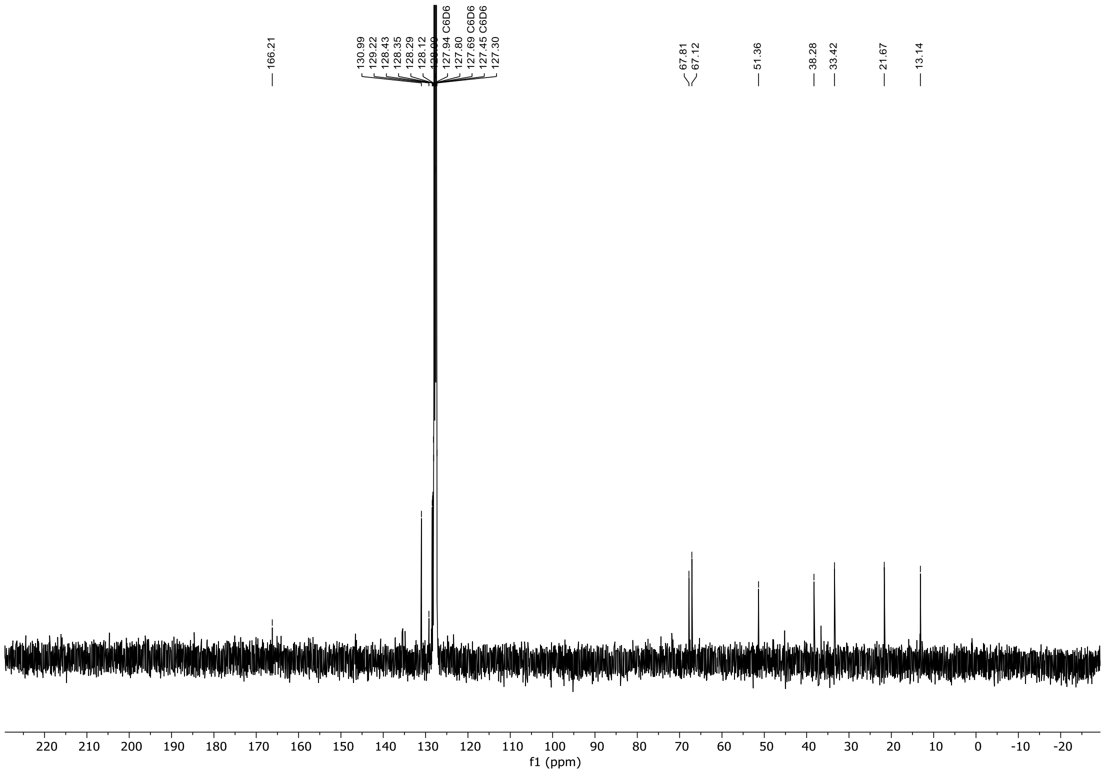

[back to Table of Contents]

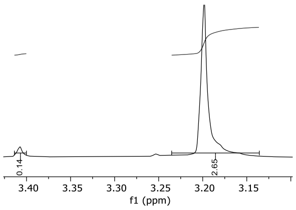

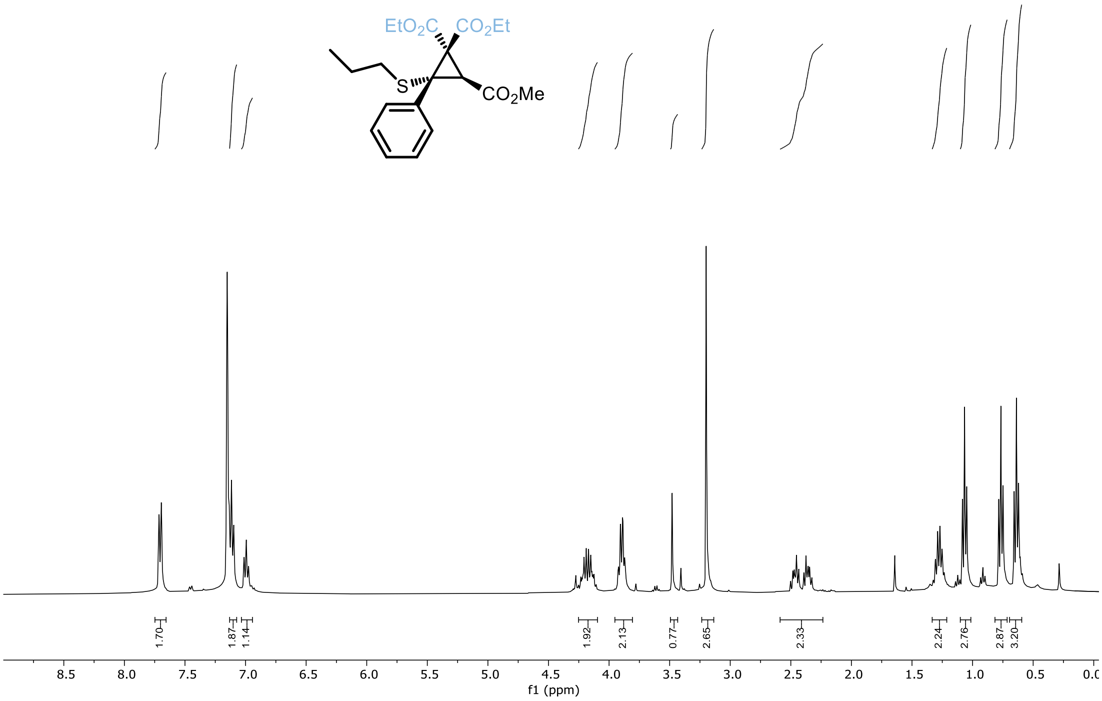

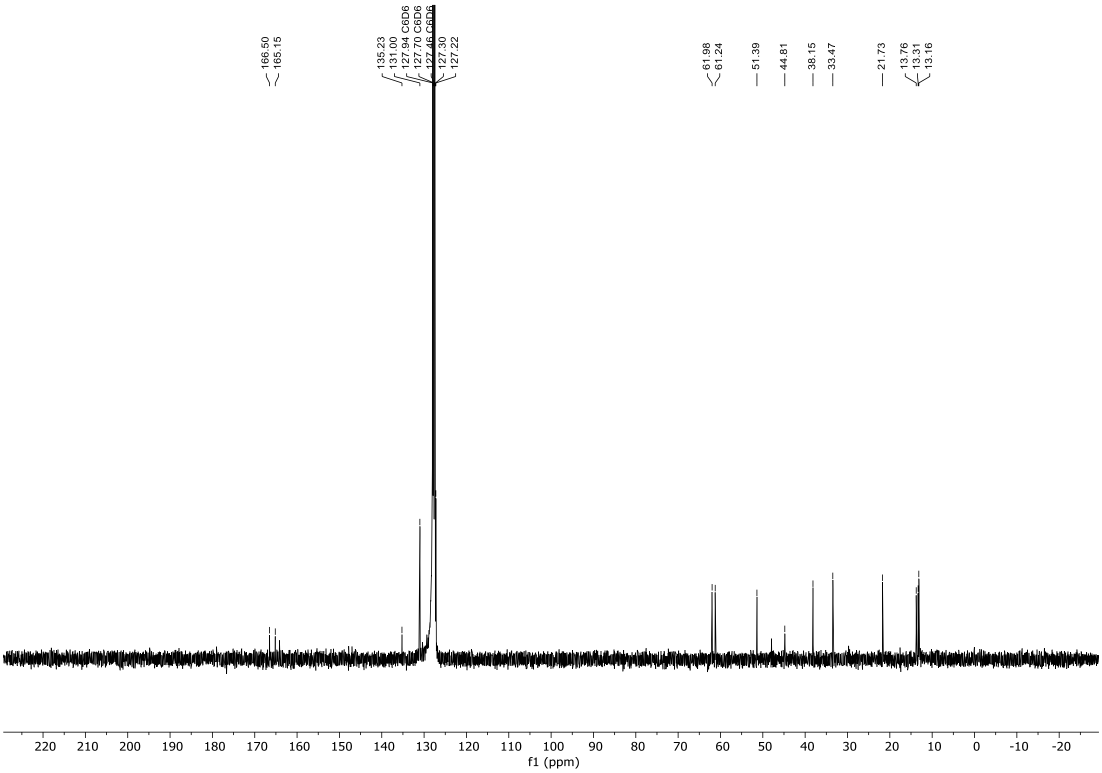

[back to Table of Contents]

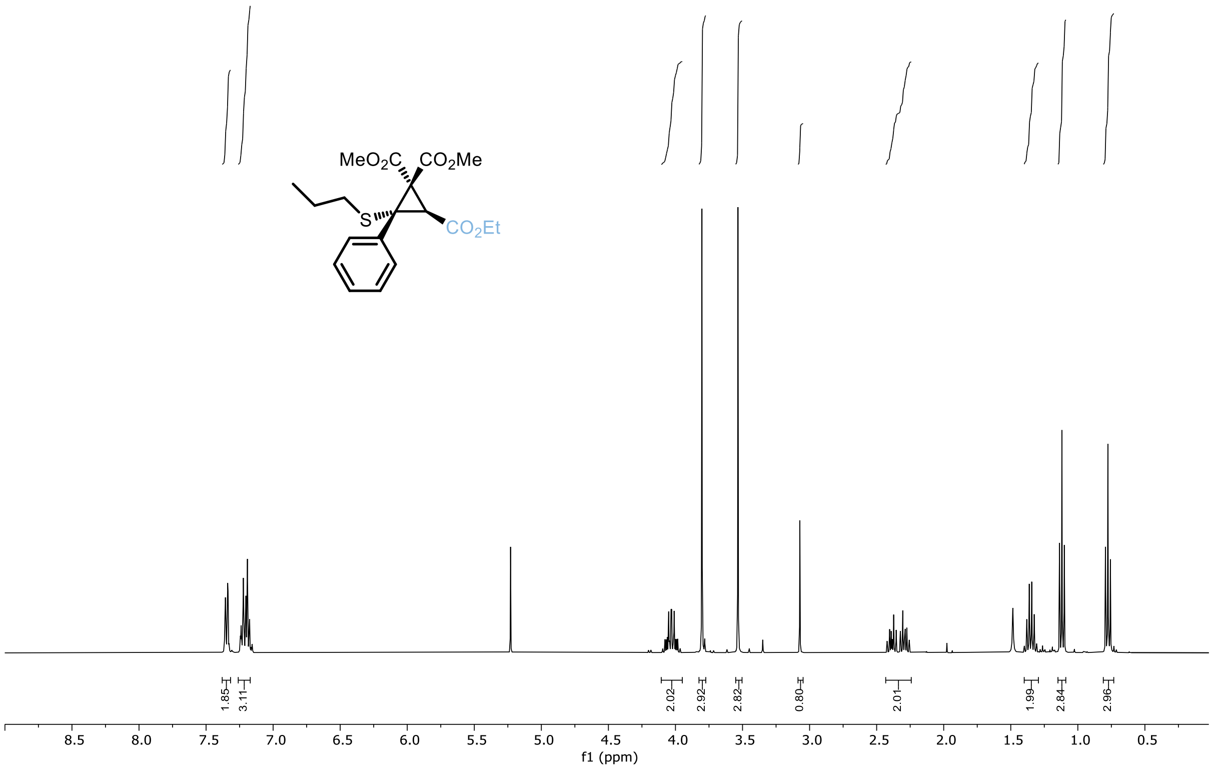

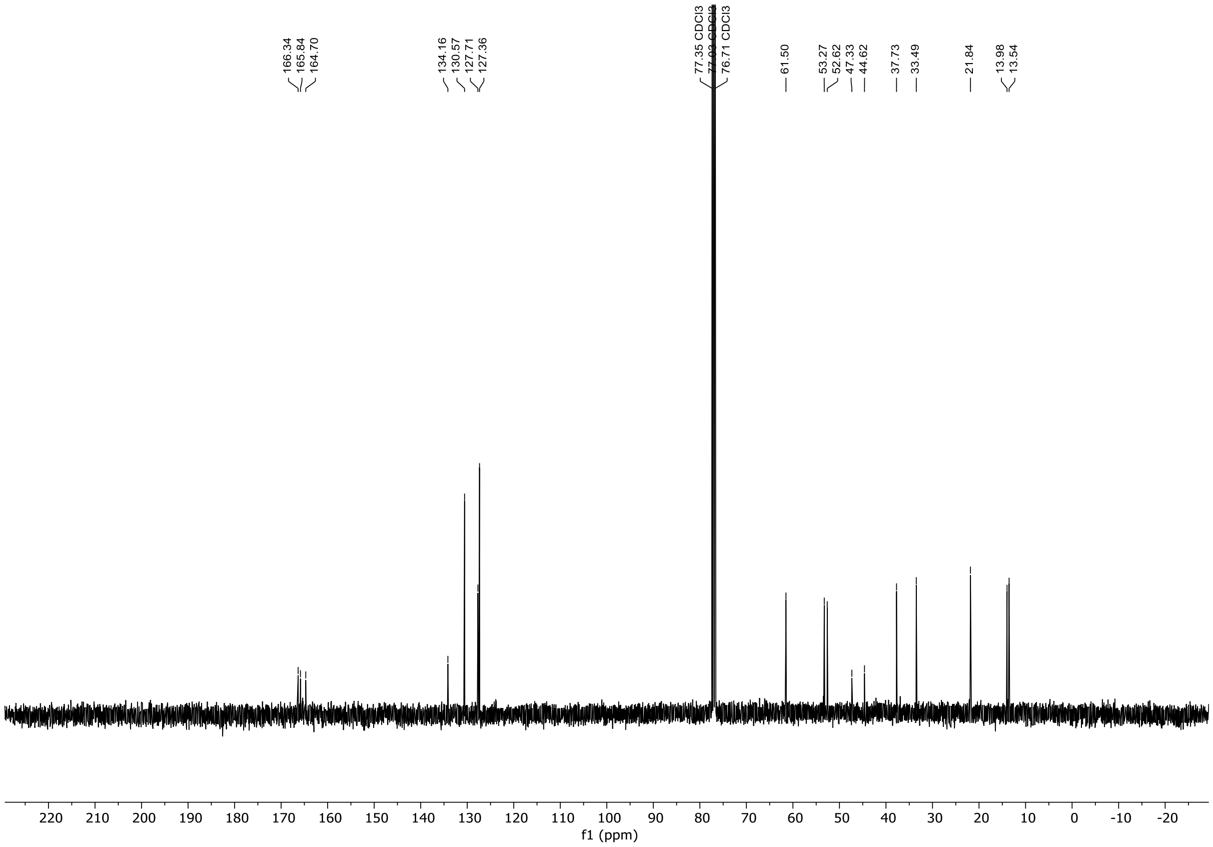

[back to Table of Contents]

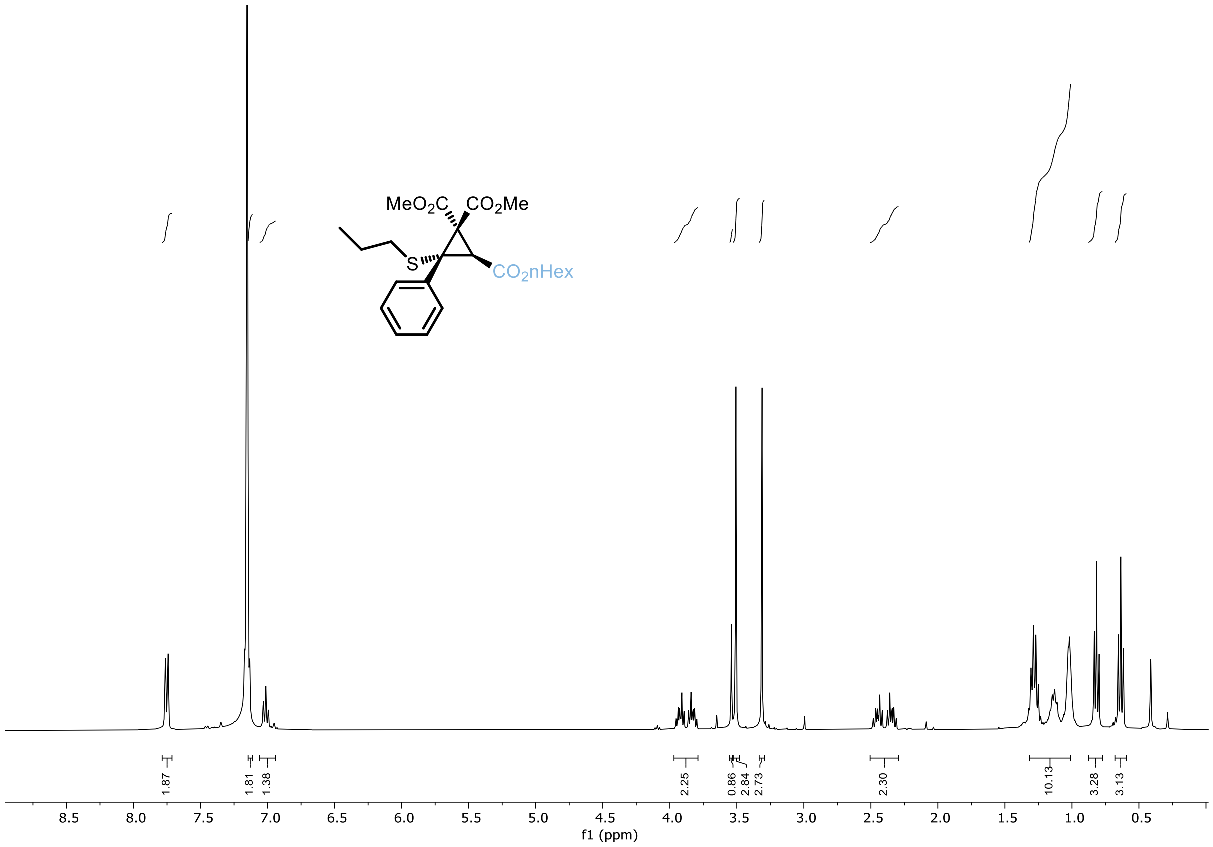

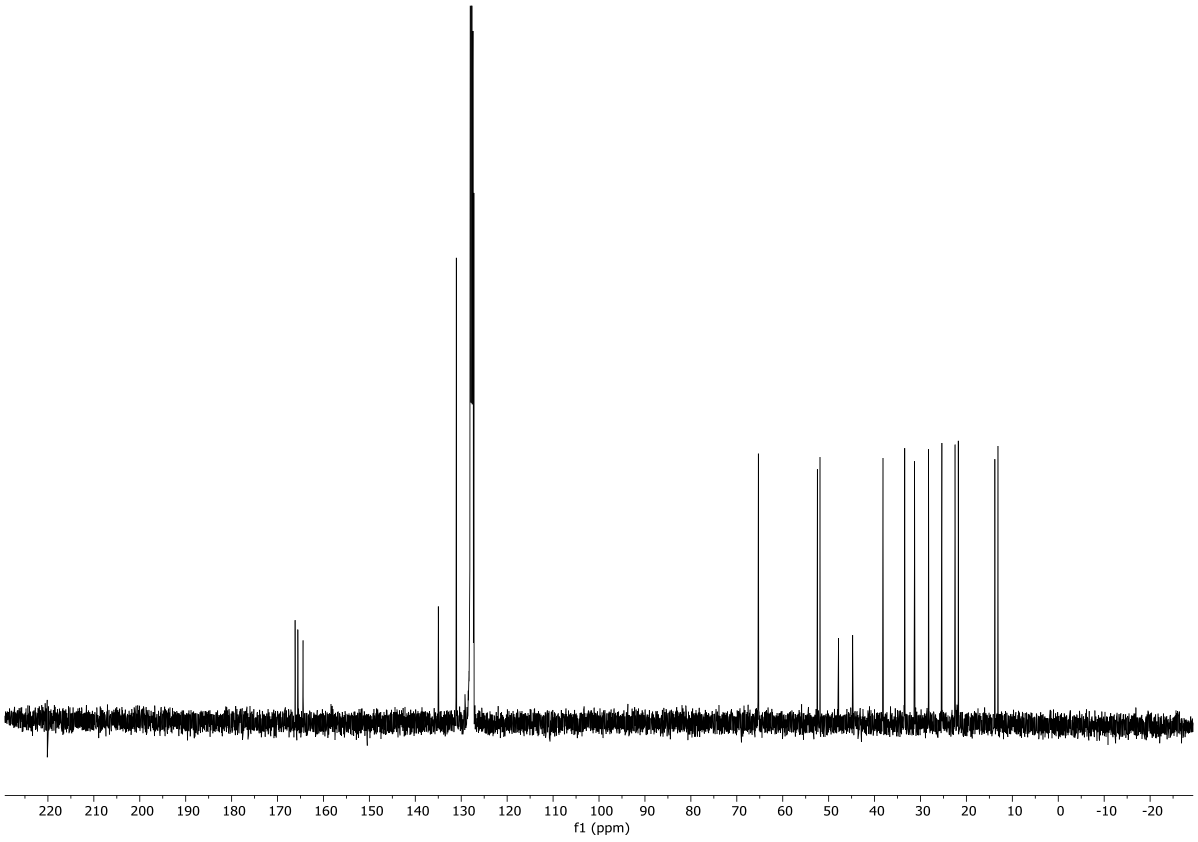

[back to Table of Contents]

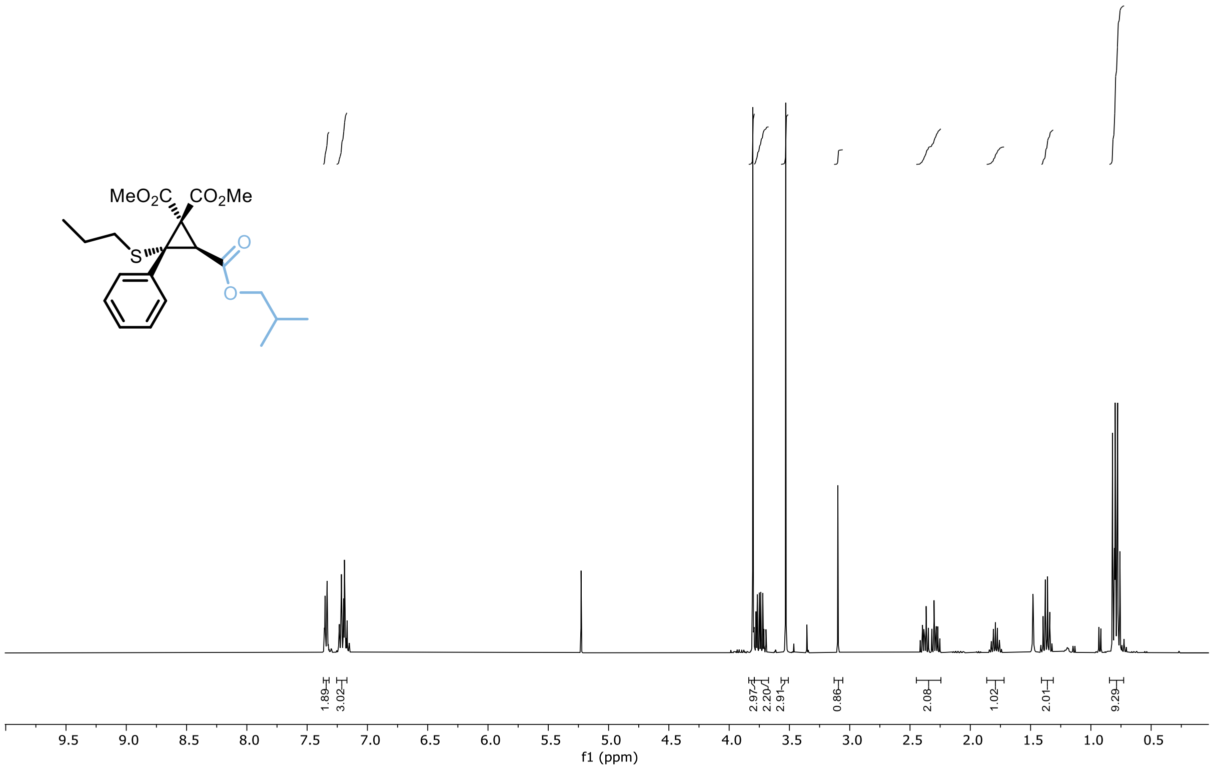

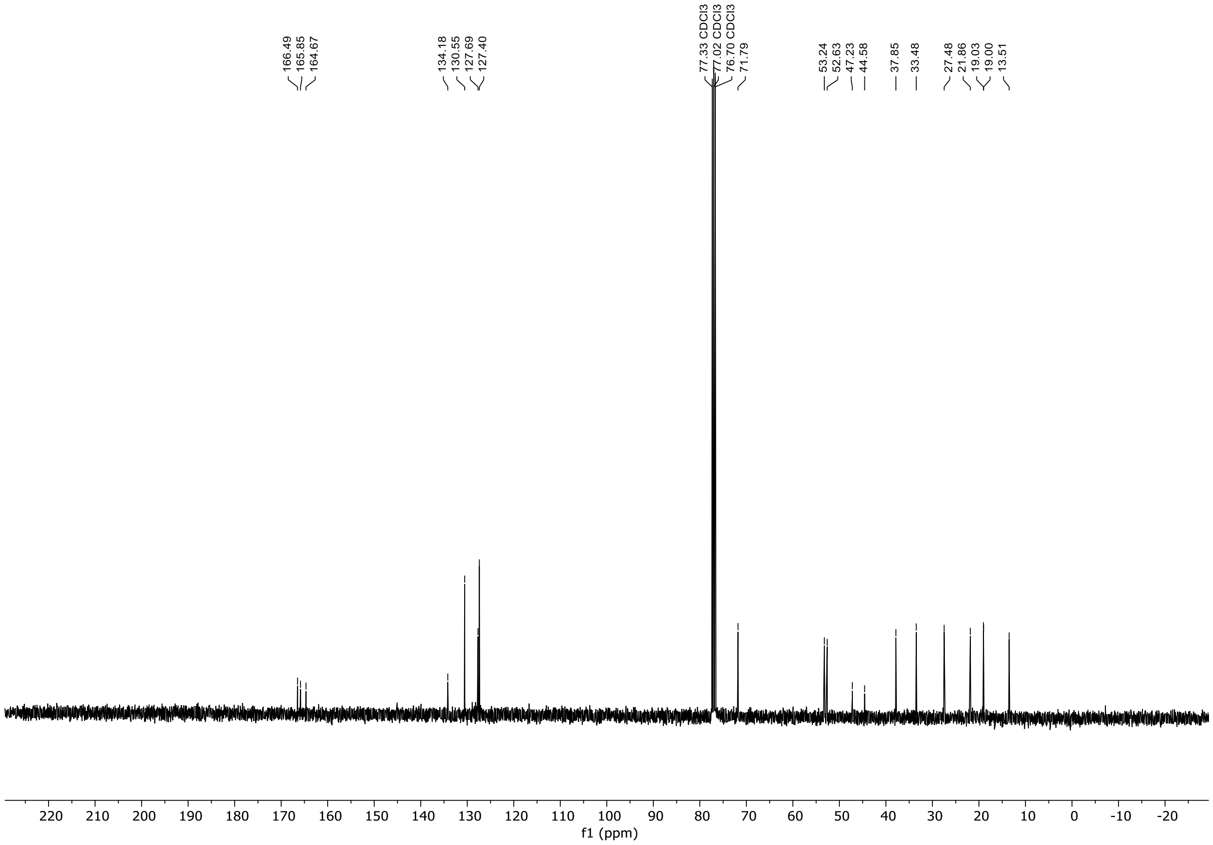

[back to Table of Contents]

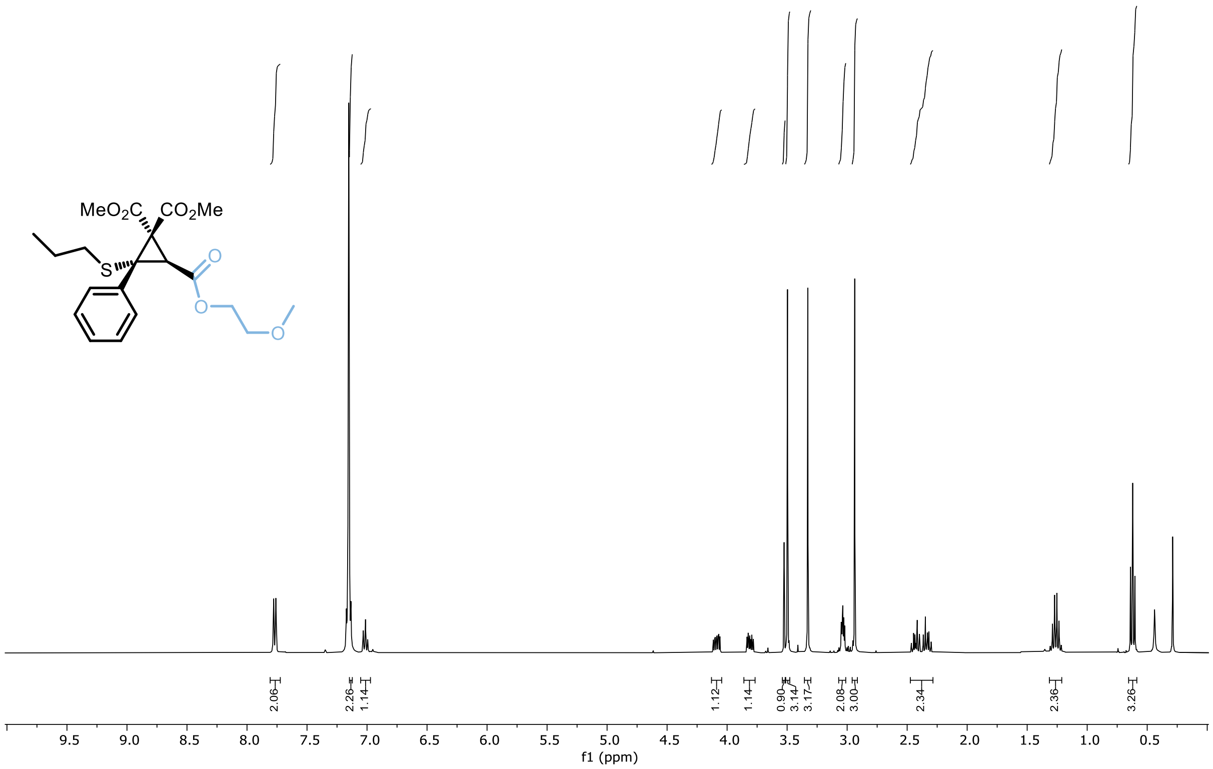

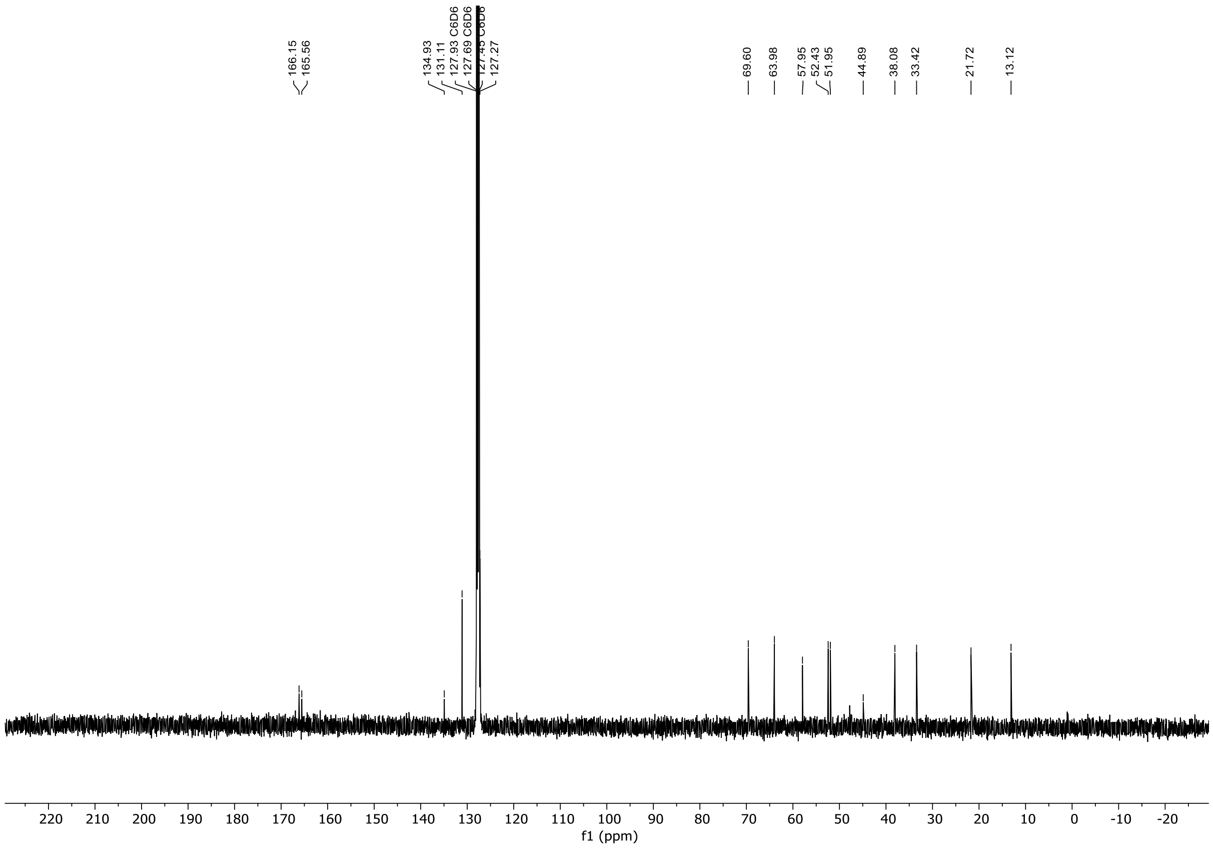

[back to Table of Contents]

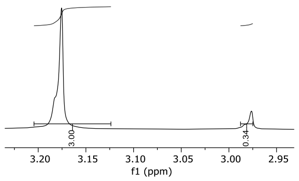

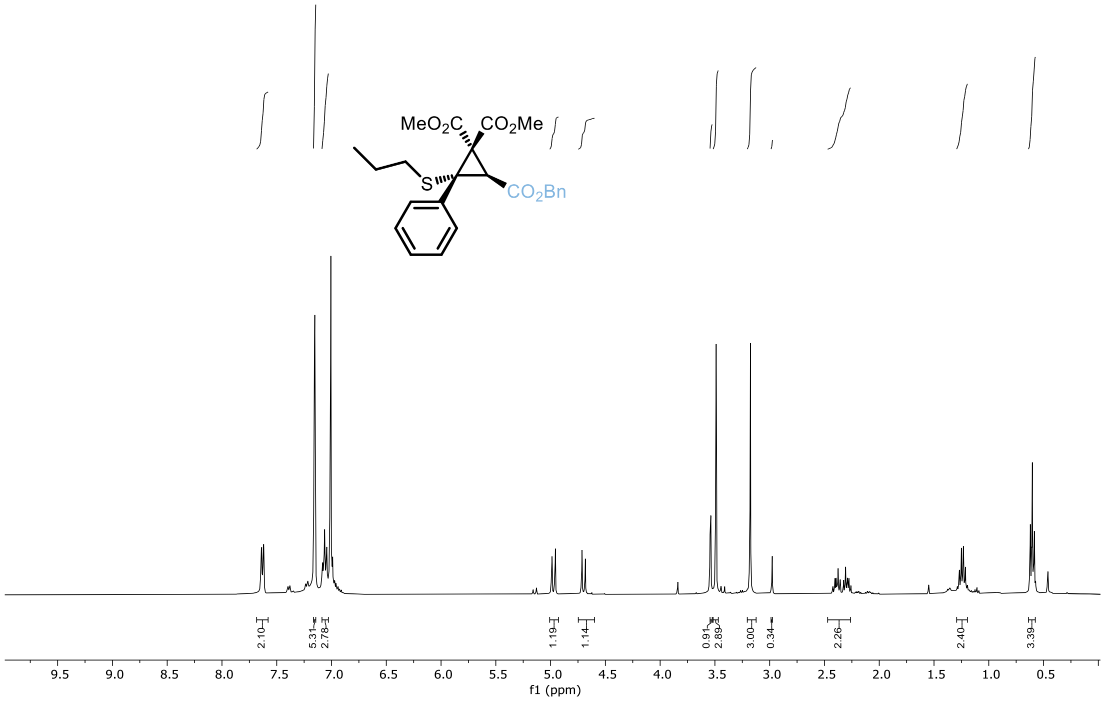

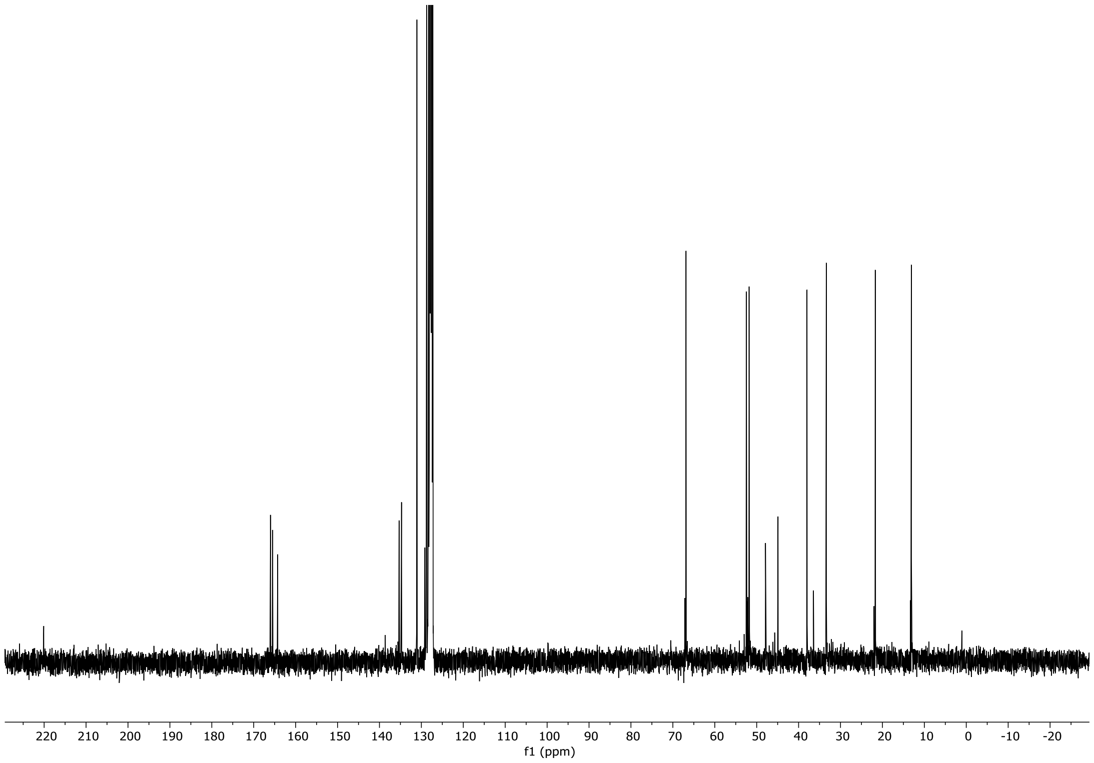

[back to Table of Contents]

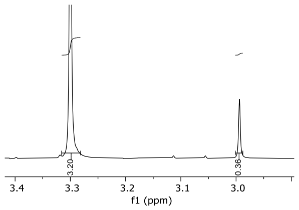

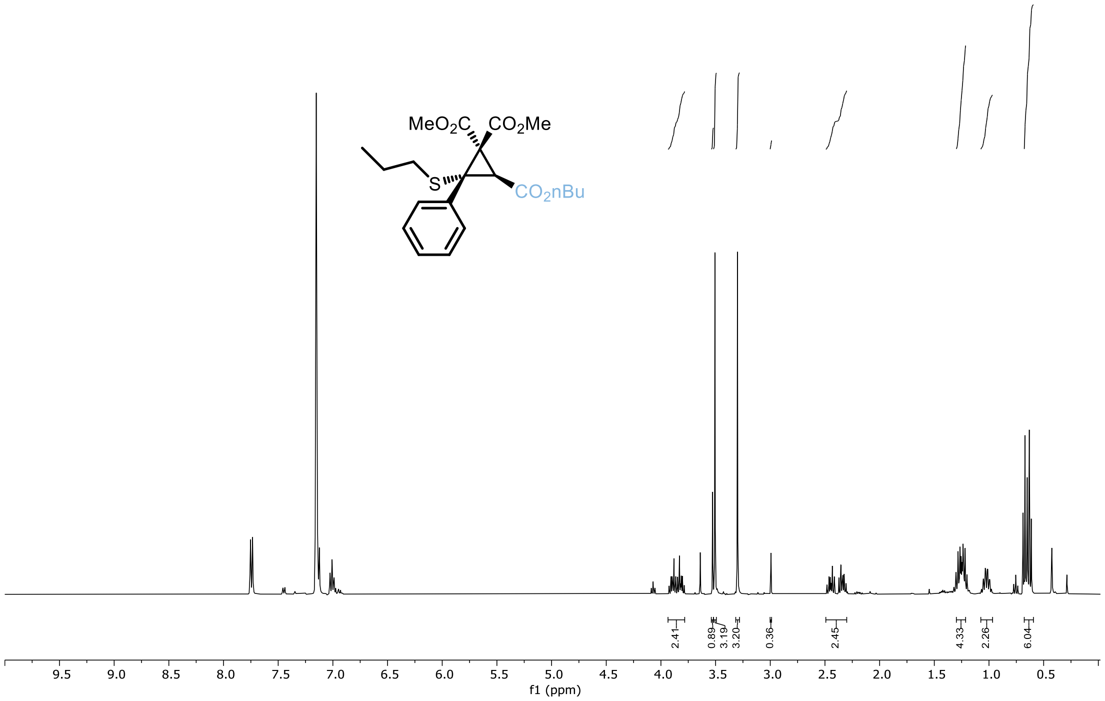

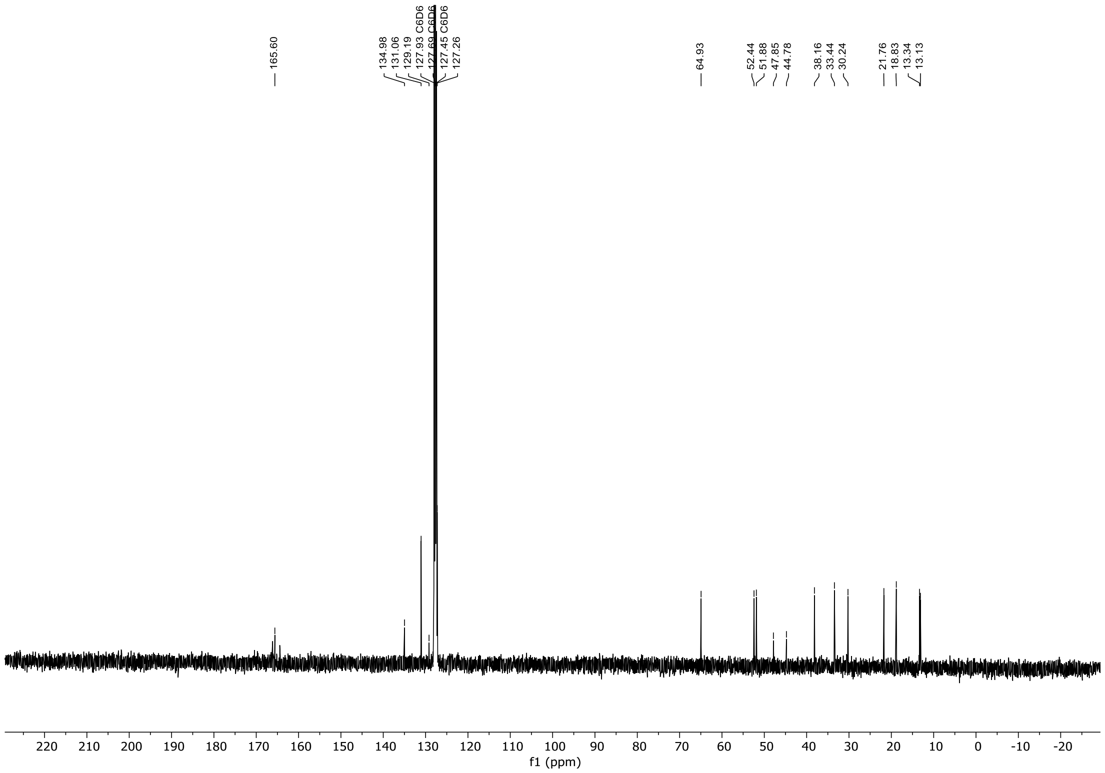

[back to Table of Contents]

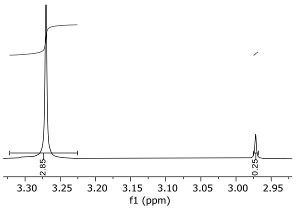

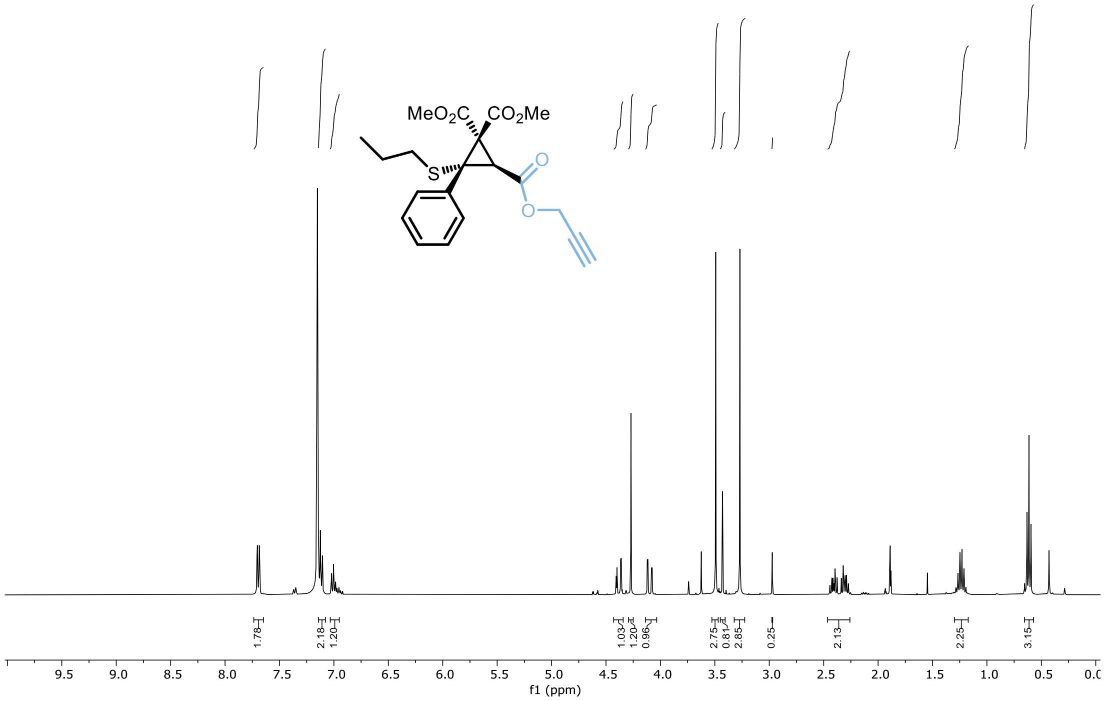

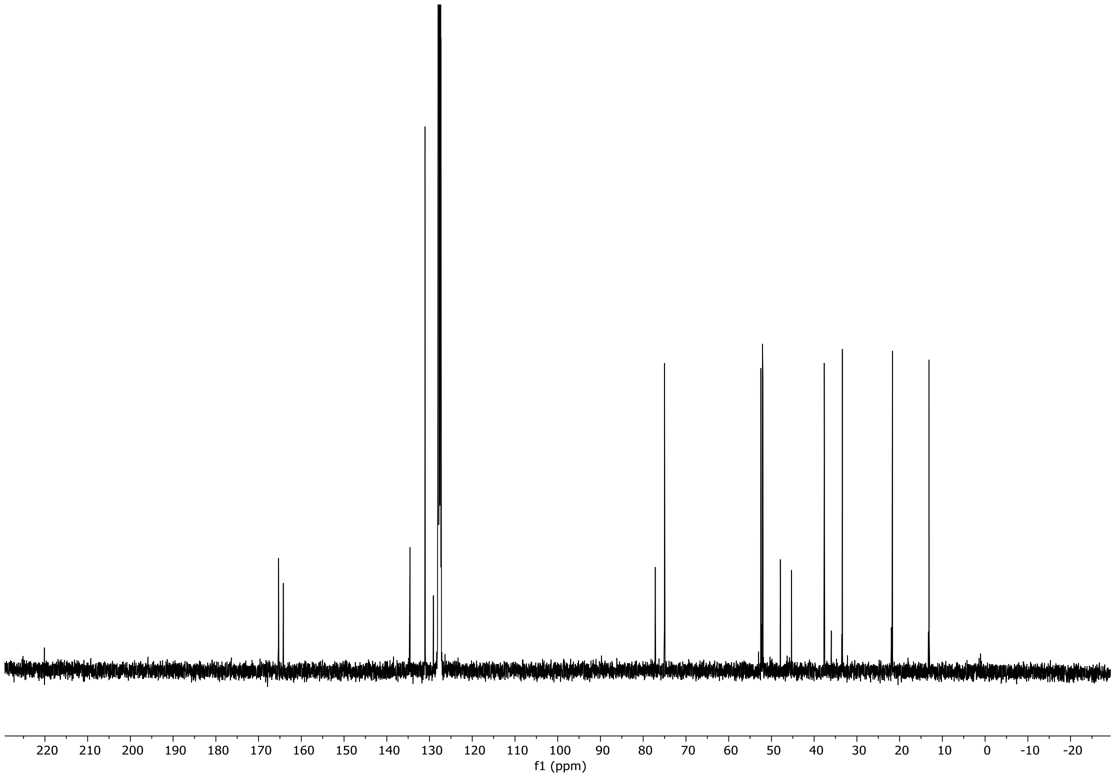

[back to Table of Contents]

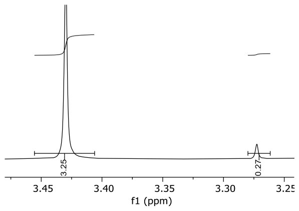

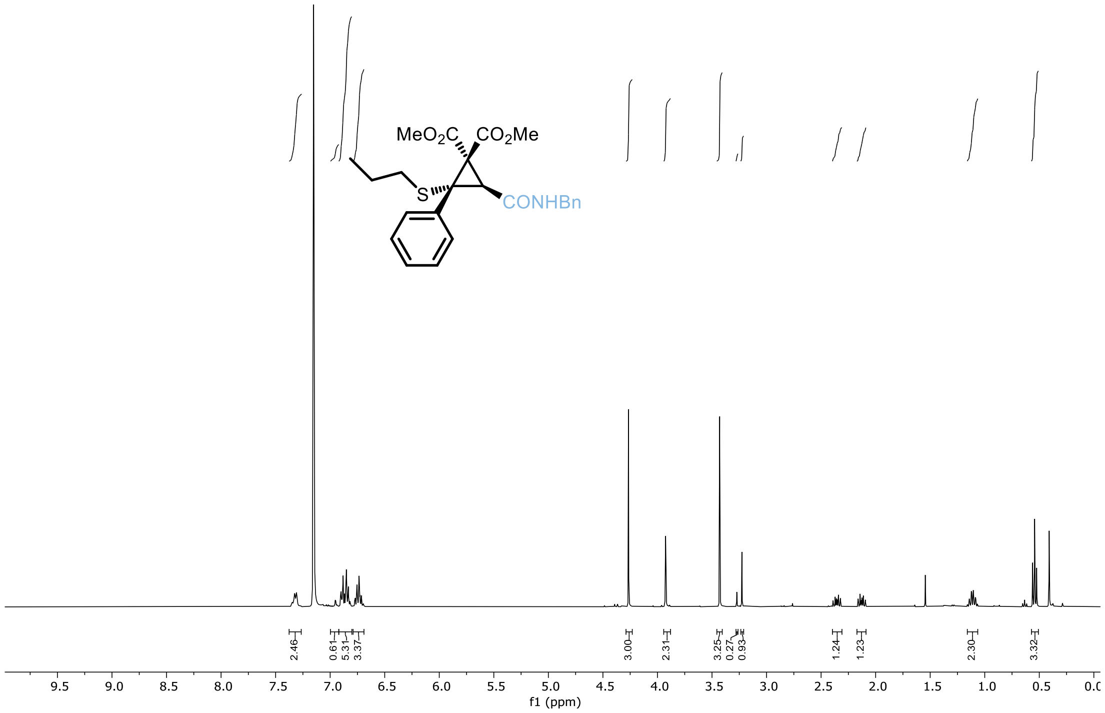

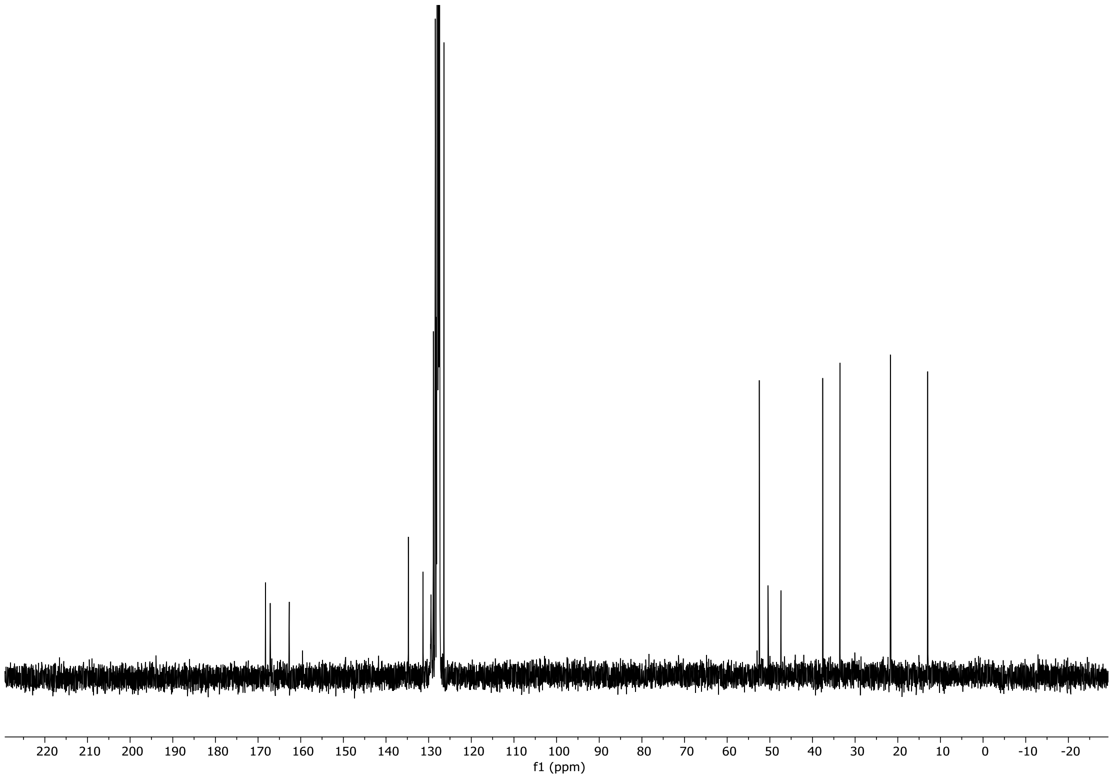

[back to Table of Contents]

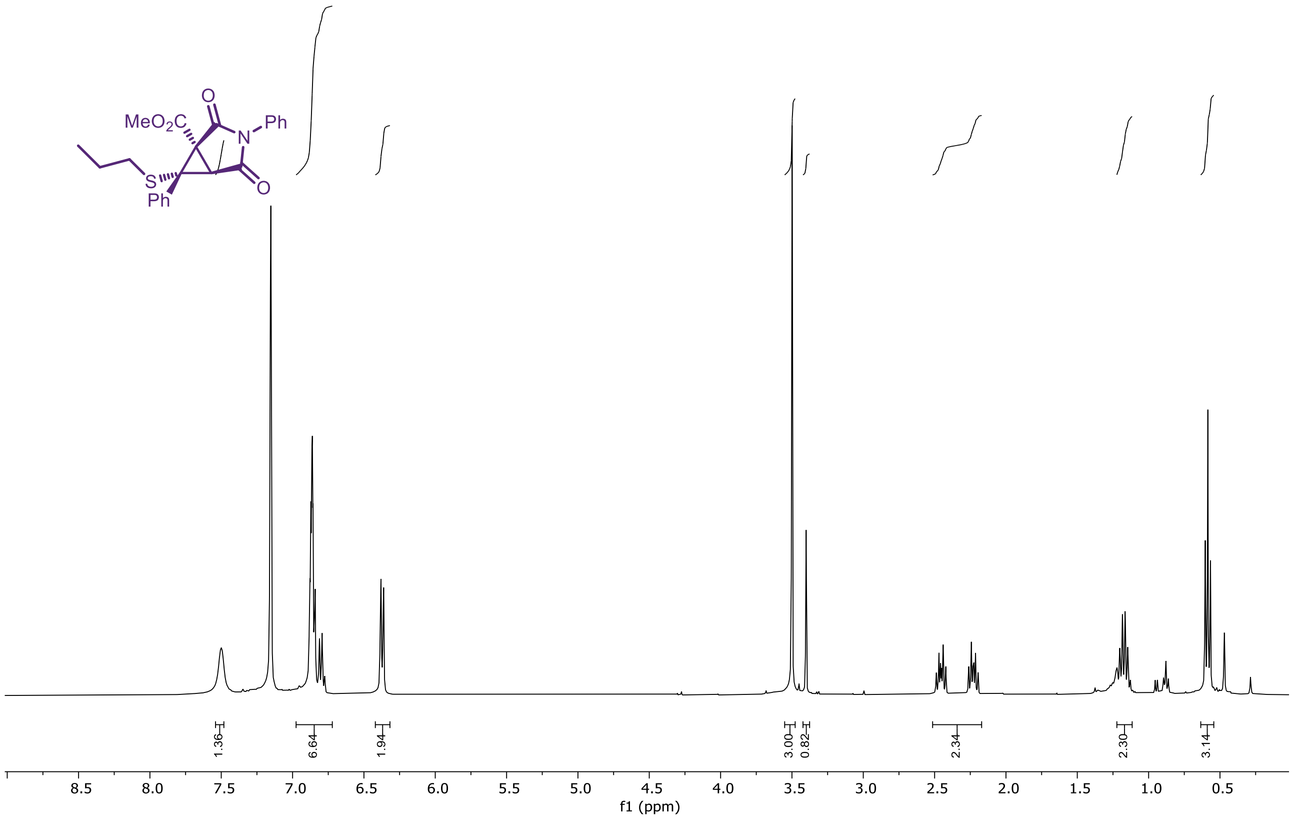

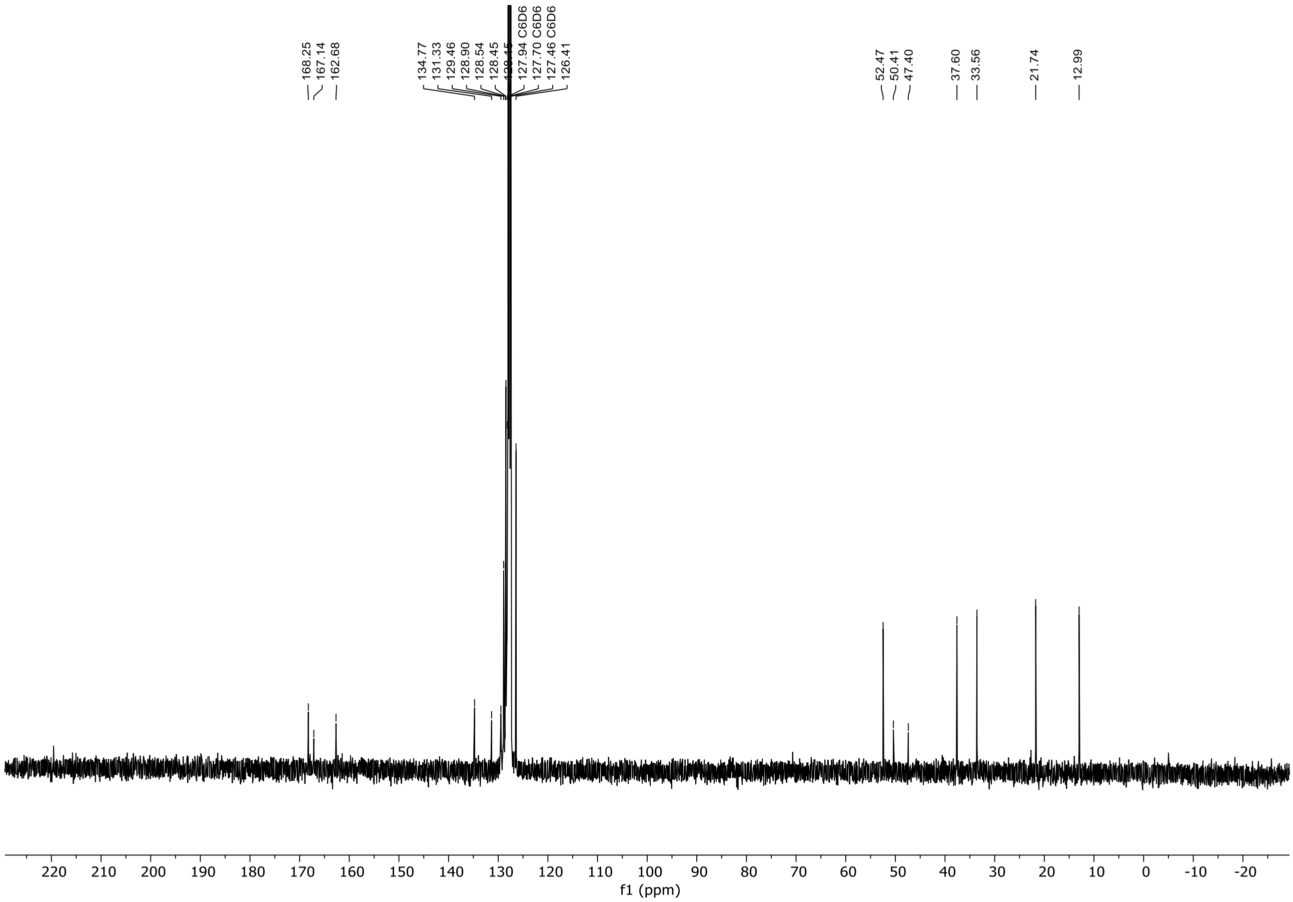

[back to Table of Contents]

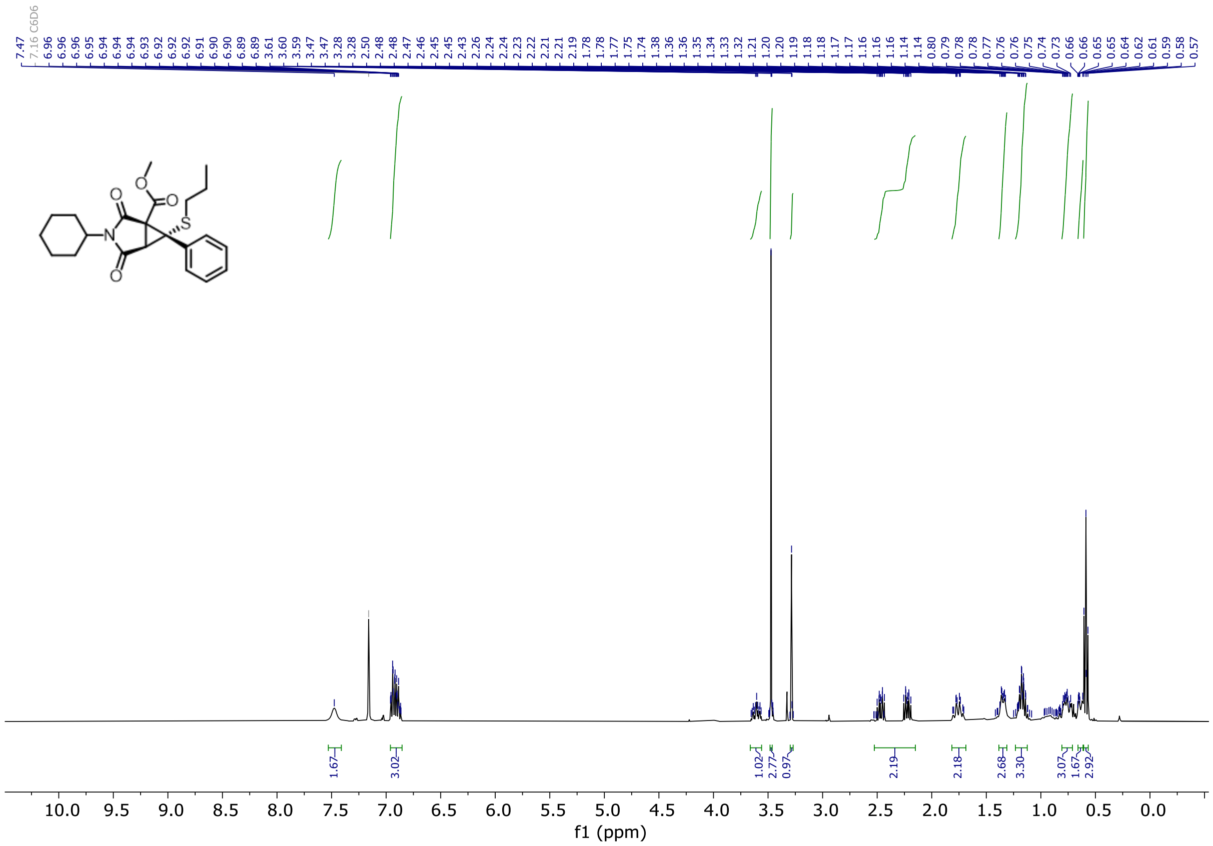

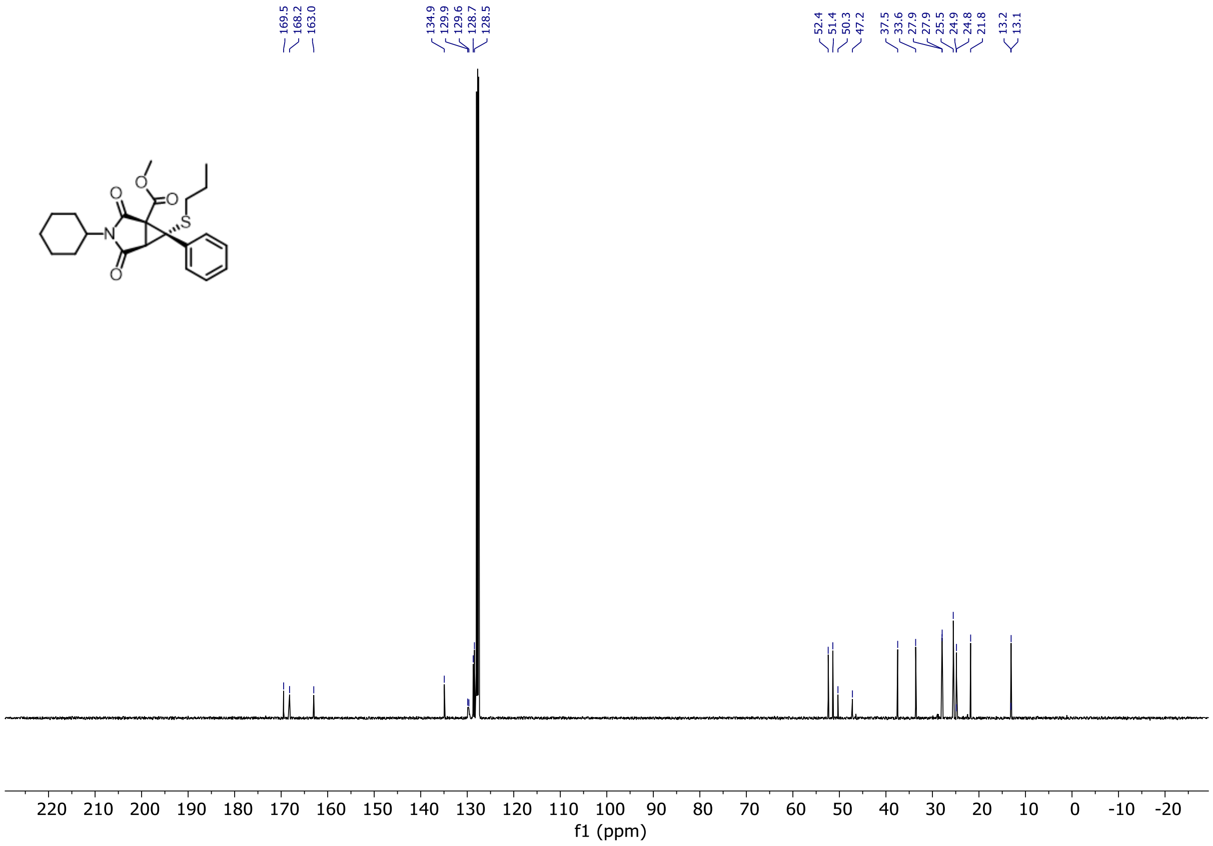

[back to Table of Contents]

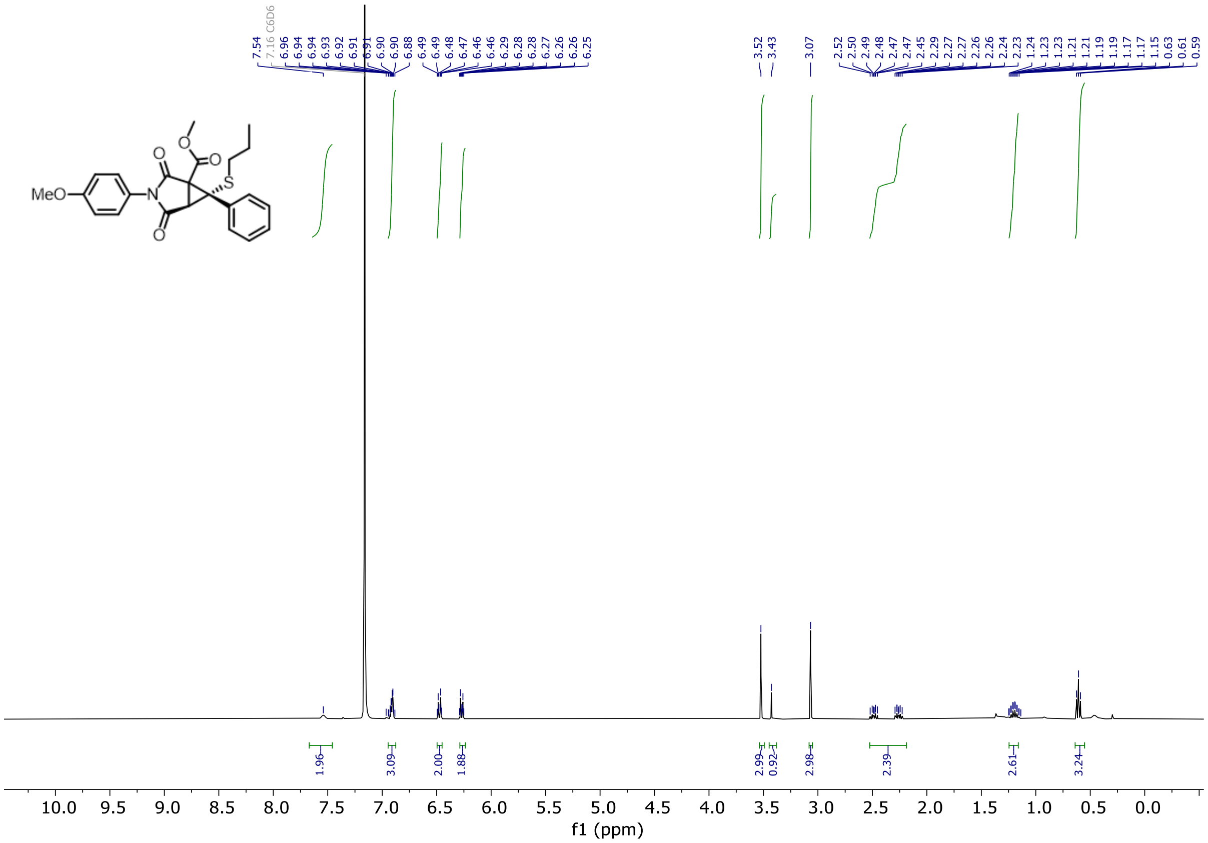

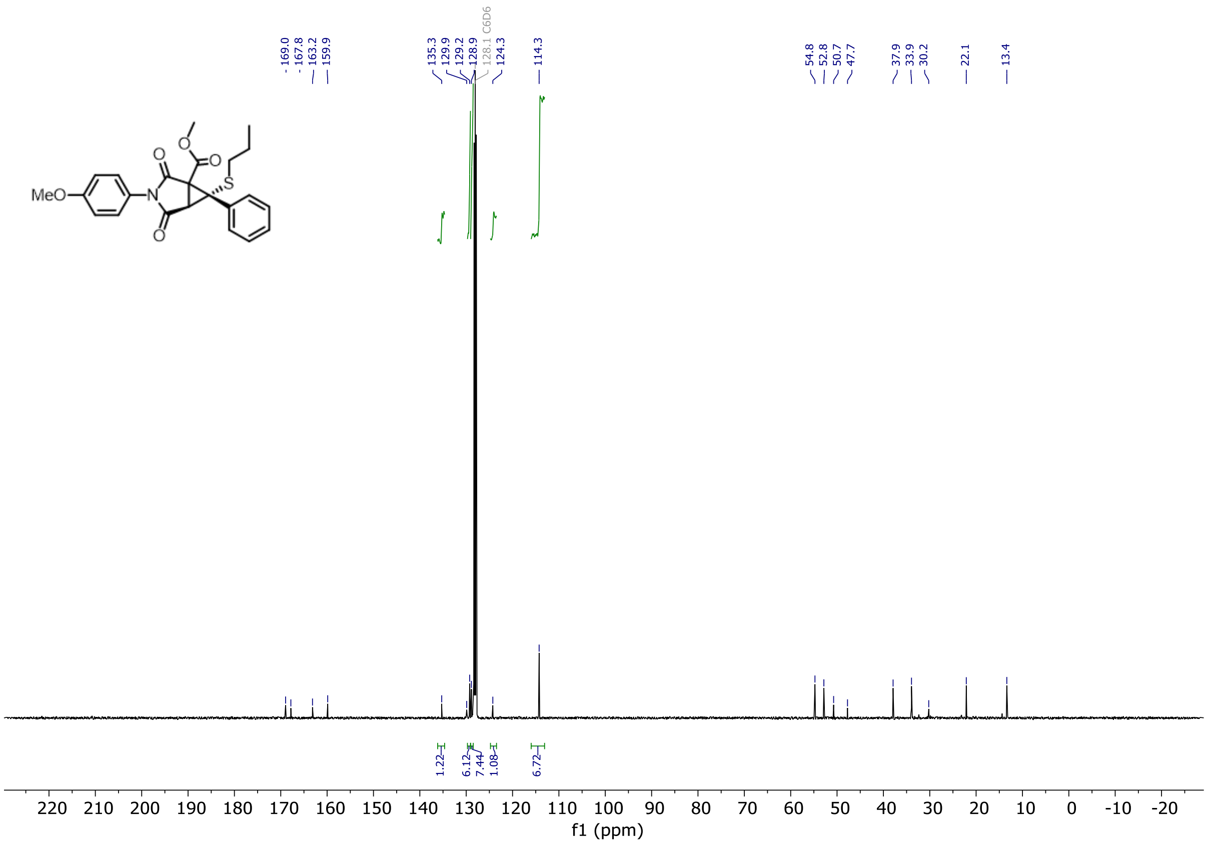

[back to Table of Contents]

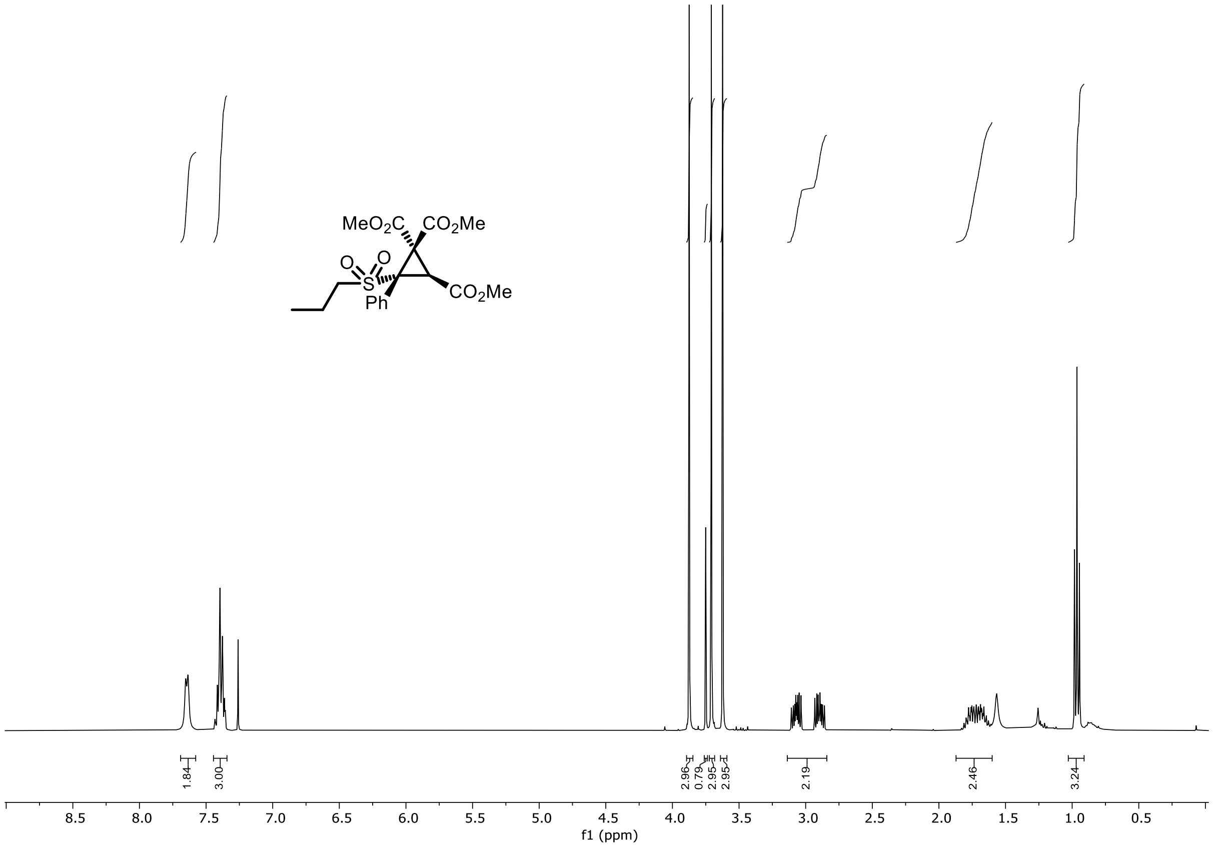

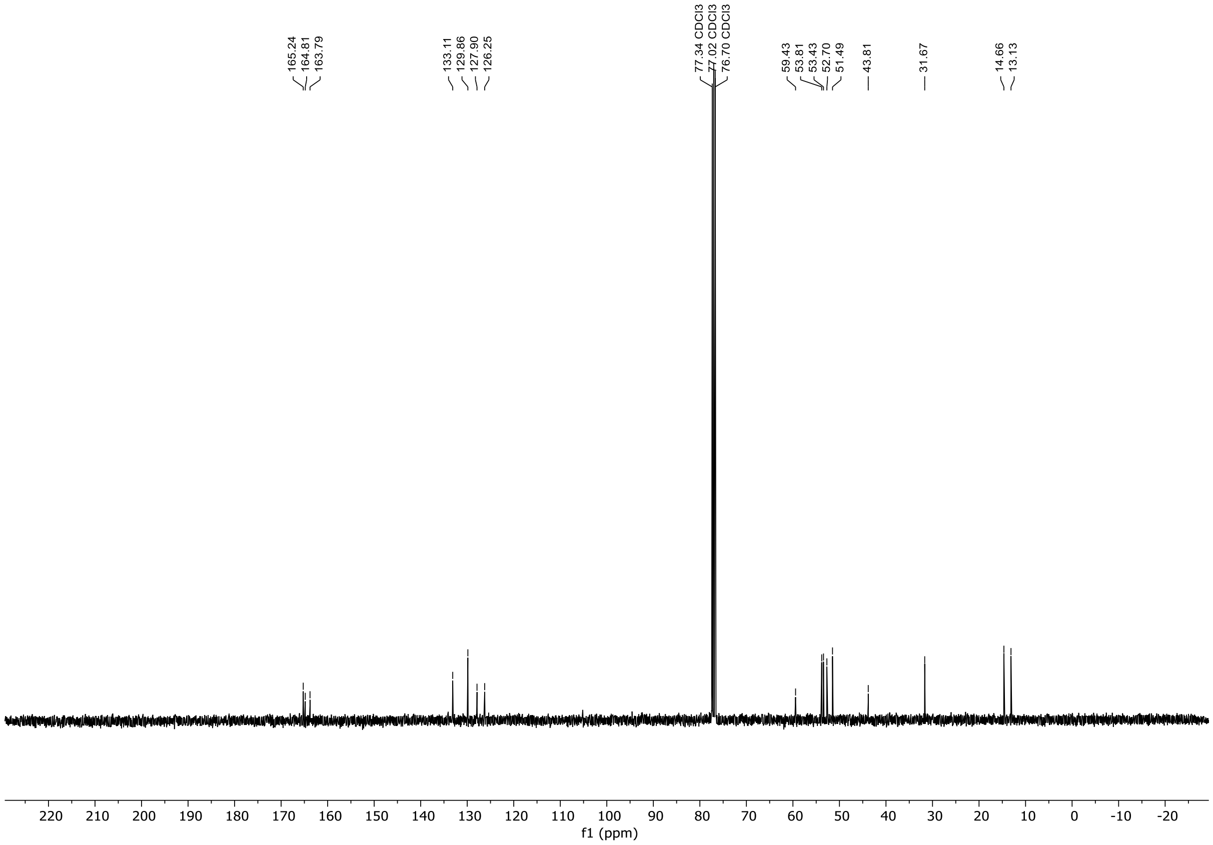

[back to Table of Contents]

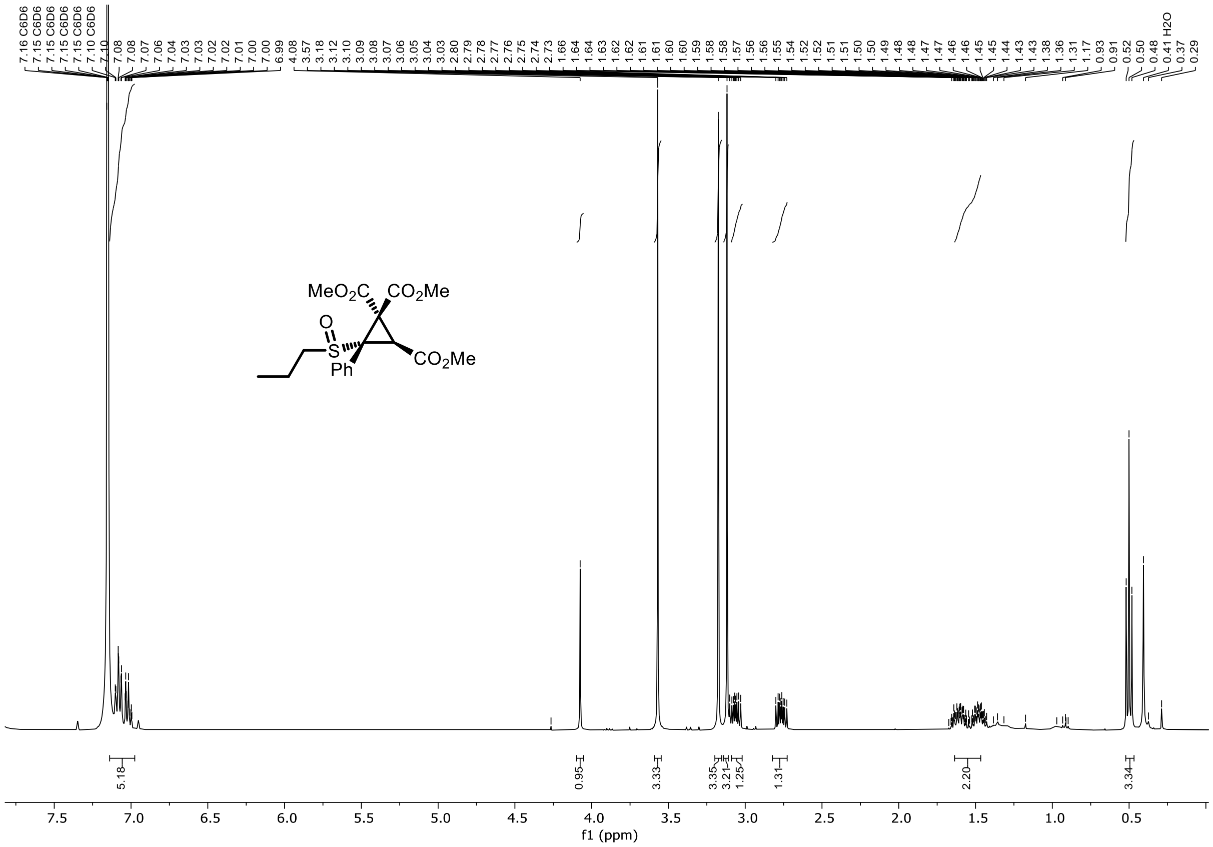

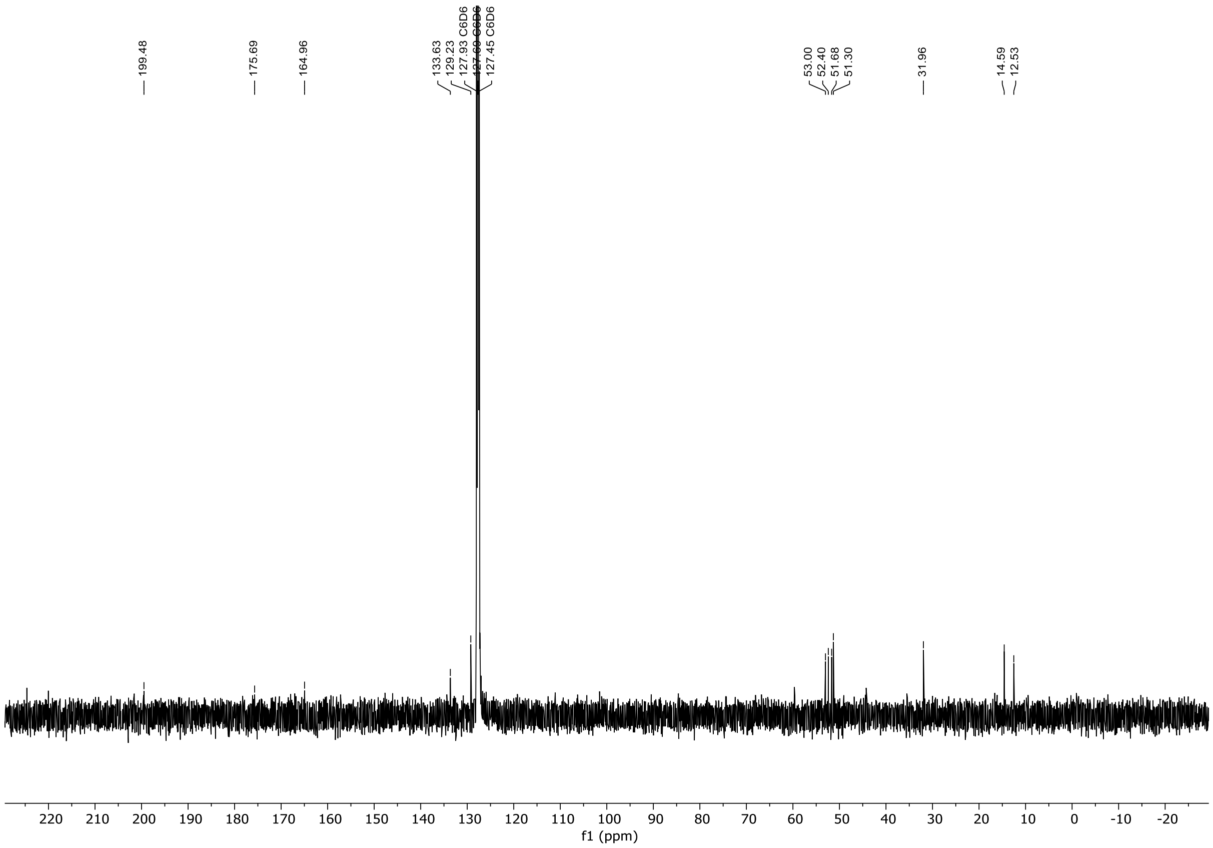

[back to Table of Contents]

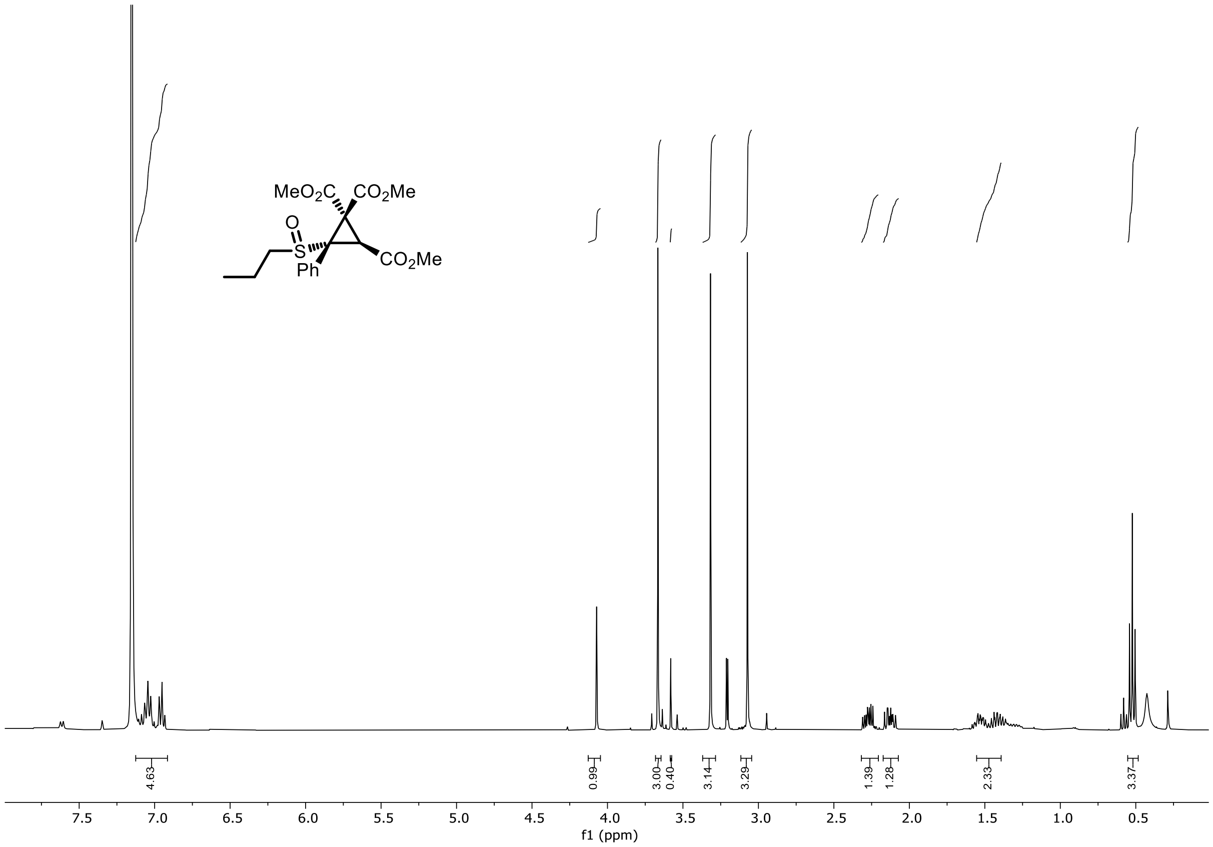

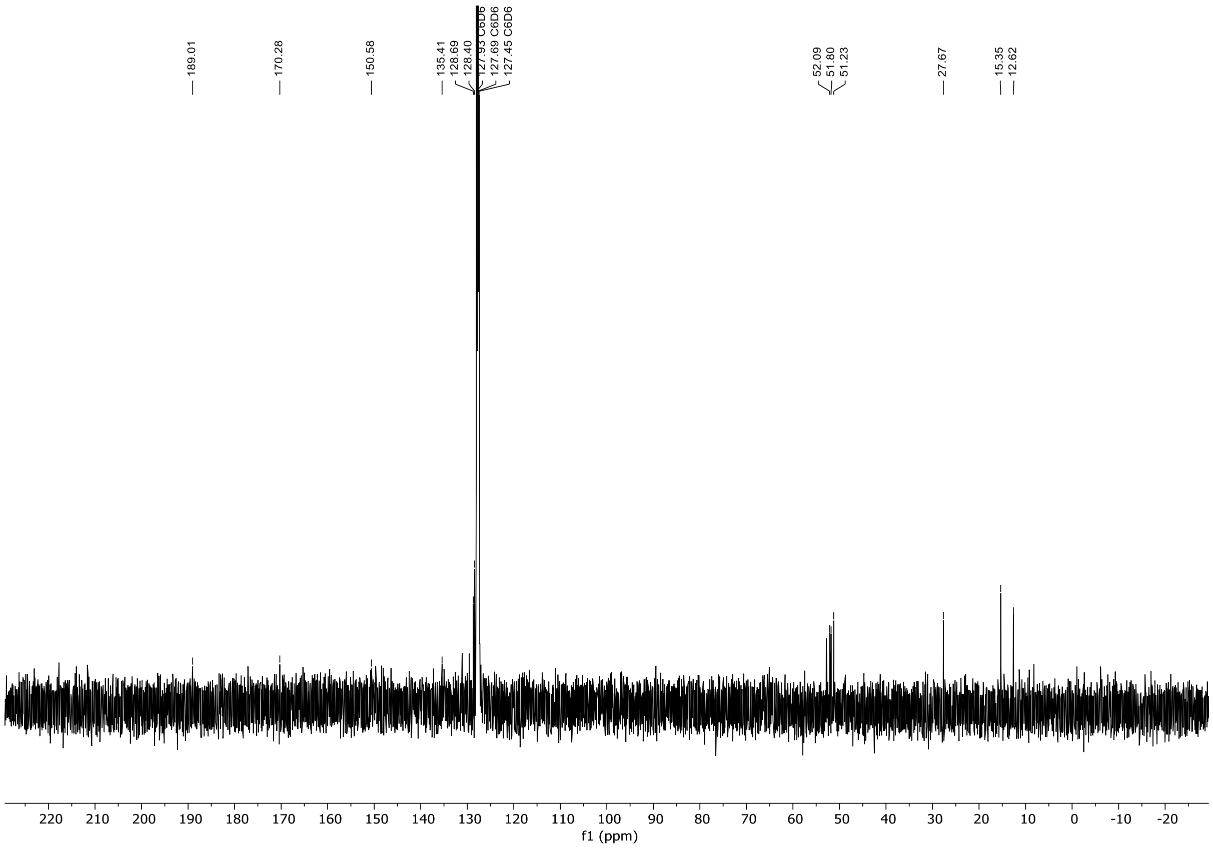

[back to Table of Contents]

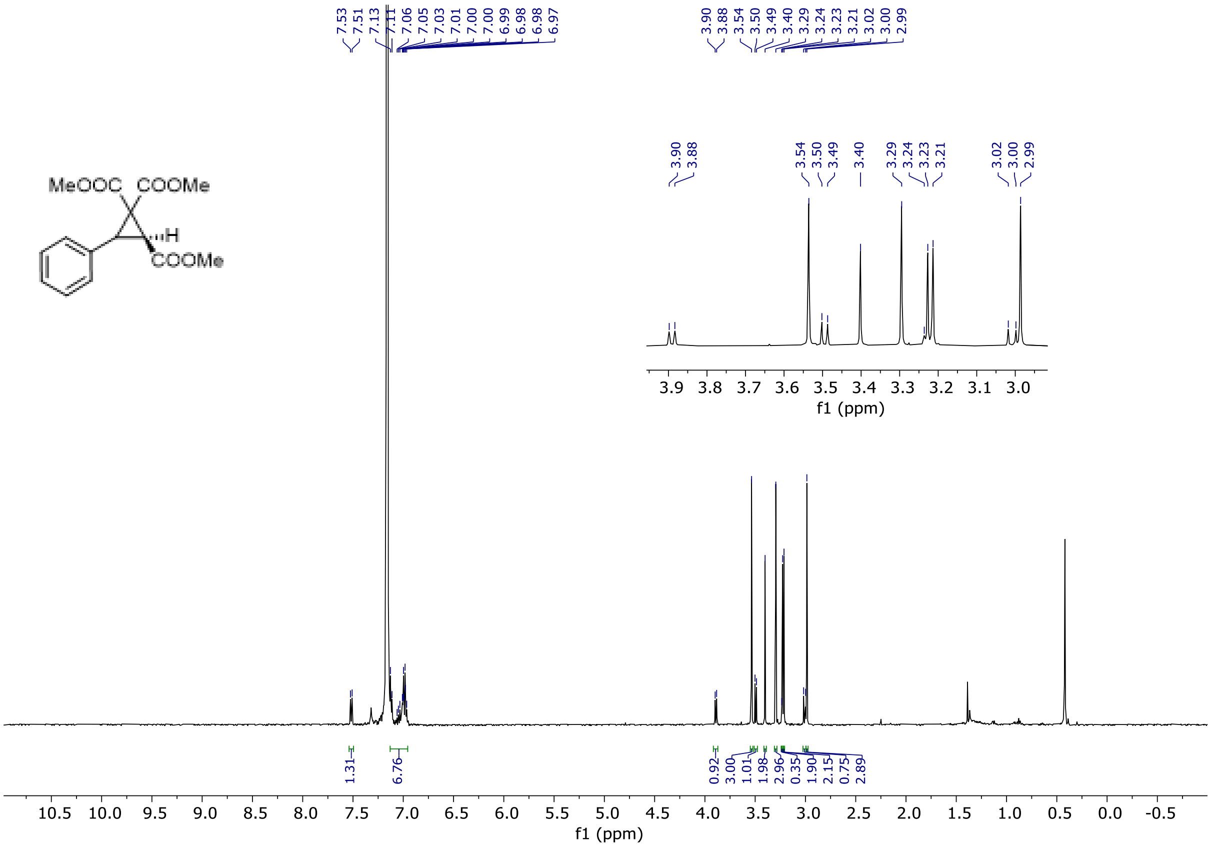

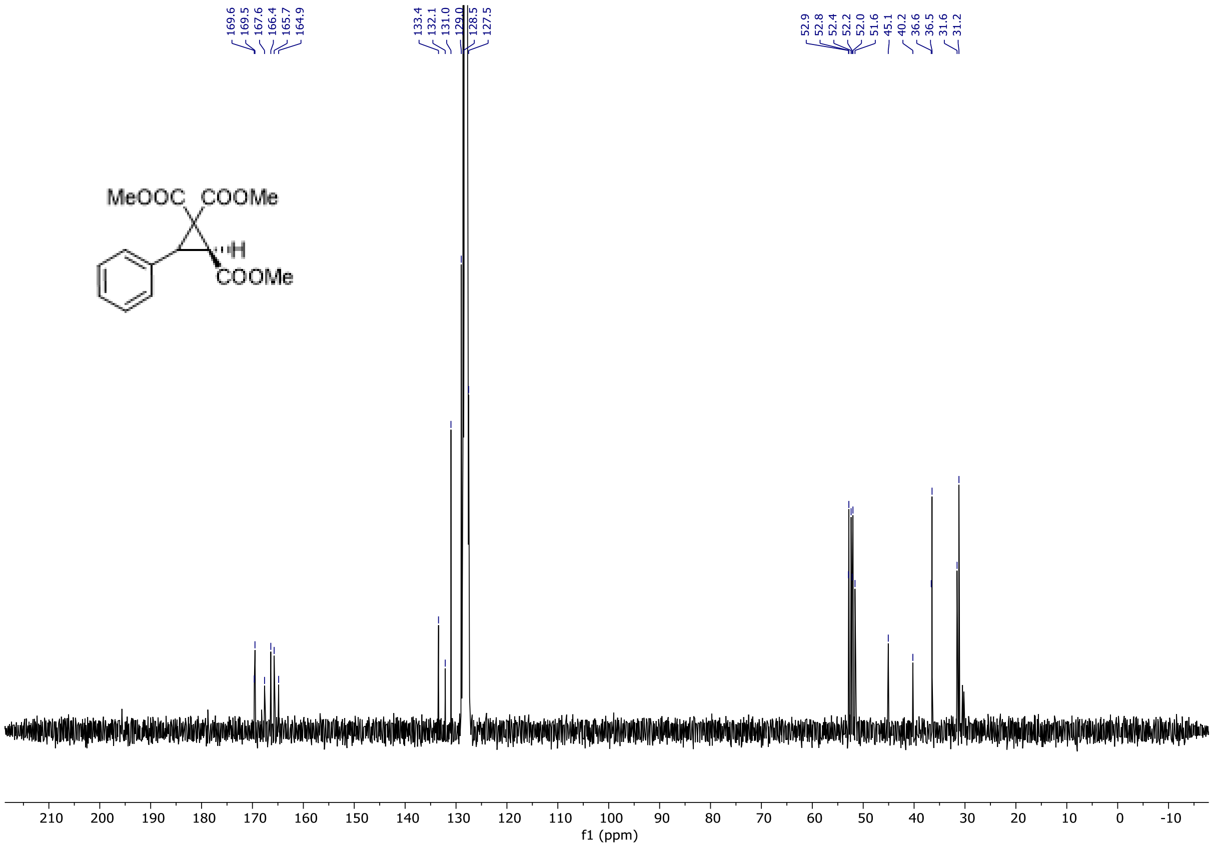

[back to Table of Contents]

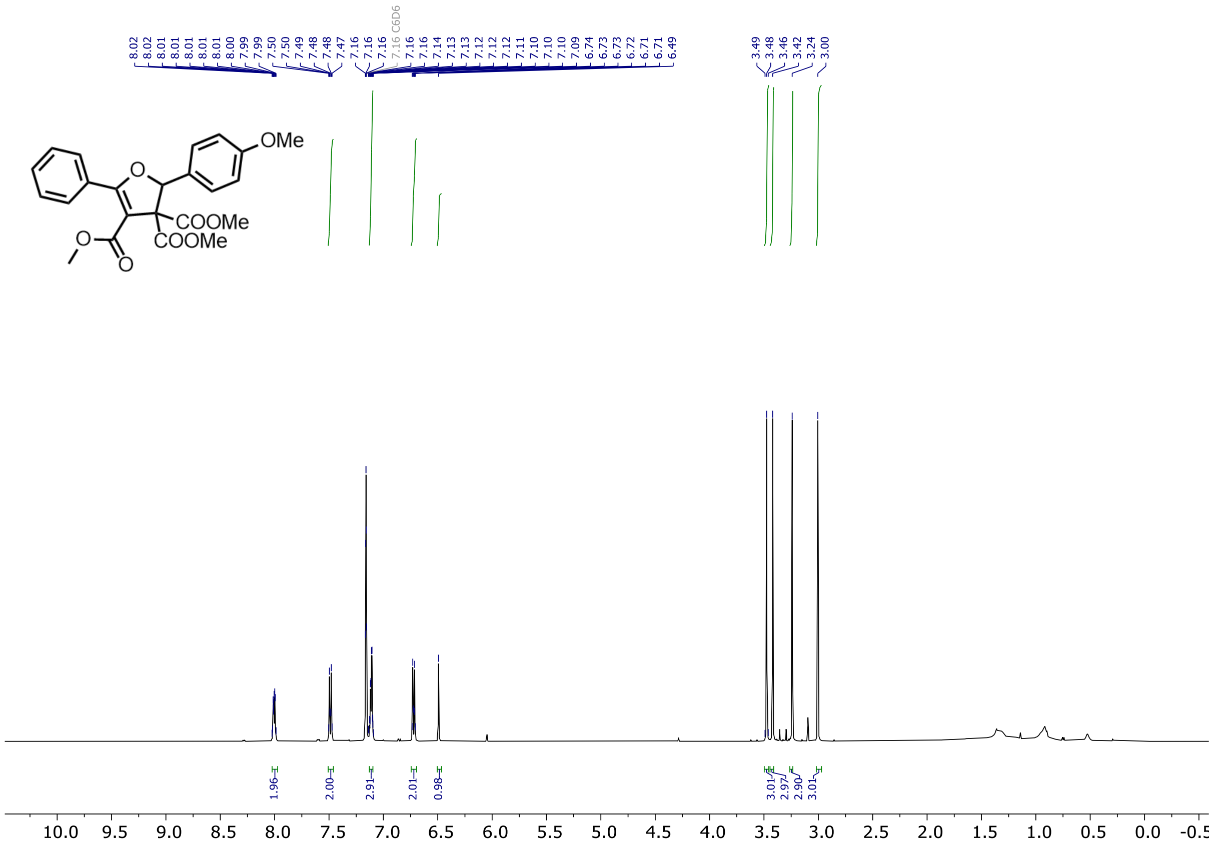

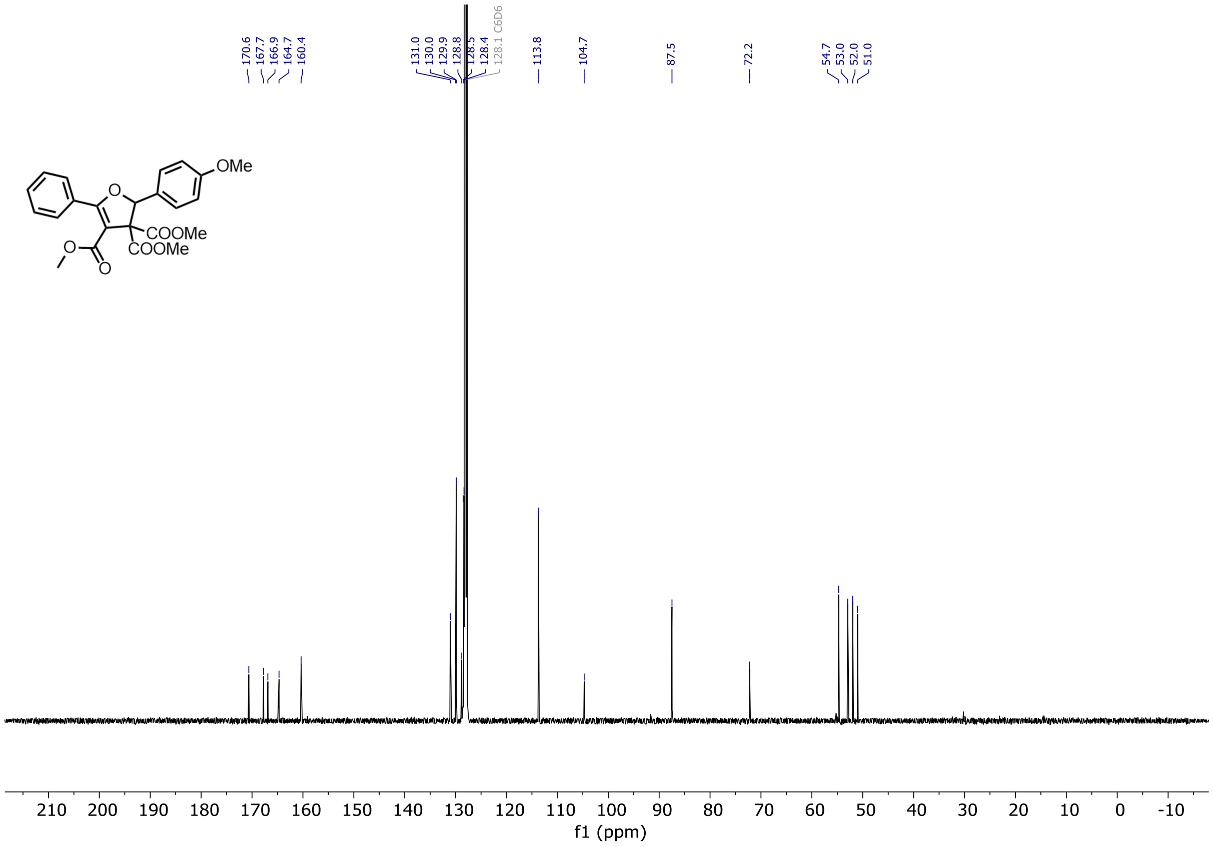

[back to Table of Contents]

HPLC Traces

Sulfa-Michael Addition Products

trimethyl (2S,3R)-3-phenyl-3-(propylthio)cyclopropane-1,1,2-tricarboxylate (**3a**)

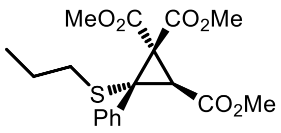

Racemic

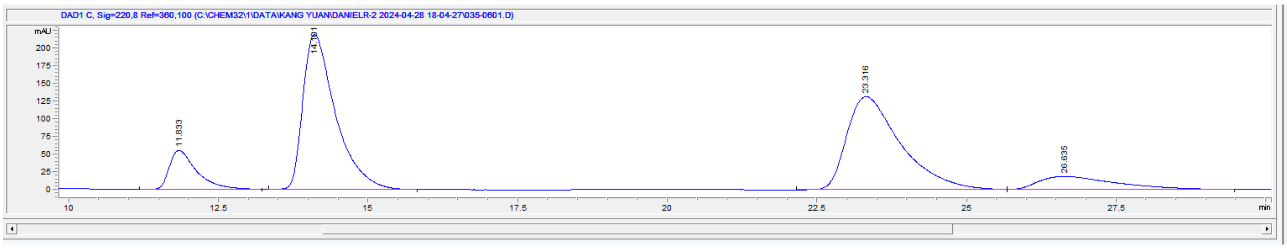

CHIRALPAK® OD, hexane/IPA = 98/2, 1 mL/min

#	Time	Area	Height	Width	Area%	Symmetry
1	11.833	1773.5	55.5	0.4749	8.850	0.489
2	14.101	8307.5	219	0.5686	41.457	0.518
3	23.316	8252.5	131.6	0.9359	41.183	0.516
4	26.635	1705.3	18.8	1.101	8.510	0.477

Enantioenriched (94% ee)

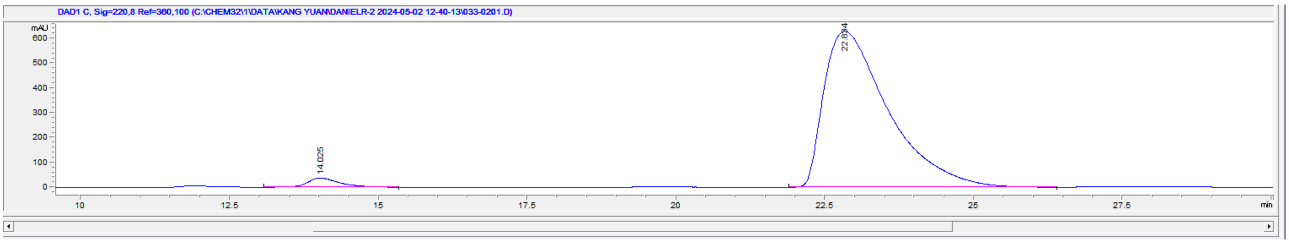

CHIRALPAK® OD, hexane/IPA = 98/2, 1 mL/min

#	Time	Area	Height	Width	Area%	Symmetry
1	14.025	1404.5	37.4	0.5487	2.891	0.661
2	22.834	47174.6	628.7	1.1154	97.109	0.446

[back to Table of Contents]

trimethyl (2S,3R)-3-(cyclohexylthio)-3-phenylcyclopropane-1,1,2-tricarboxylate (**3b**)

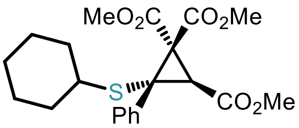

Racemic

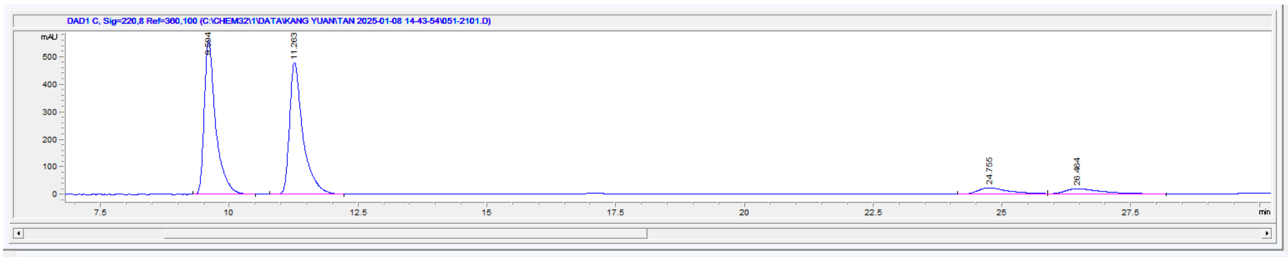

CHIRALPAK® IA, hexane/IPA = 97/3, 1 mL/min

#	Time	Area	Height	Width	Area%	Symmetry
1	9.594	8711.8	560.5	0.2293	43.727	0.552
2	11.263	8758.8	479.4	0.2671	43.963	0.543
3	24.755	1163.3	24.7	0.6241	5.839	0.529
4	26.464	1289.2	21.7	0.7496	6.471	0.427

Enantioenriched (94% ee)

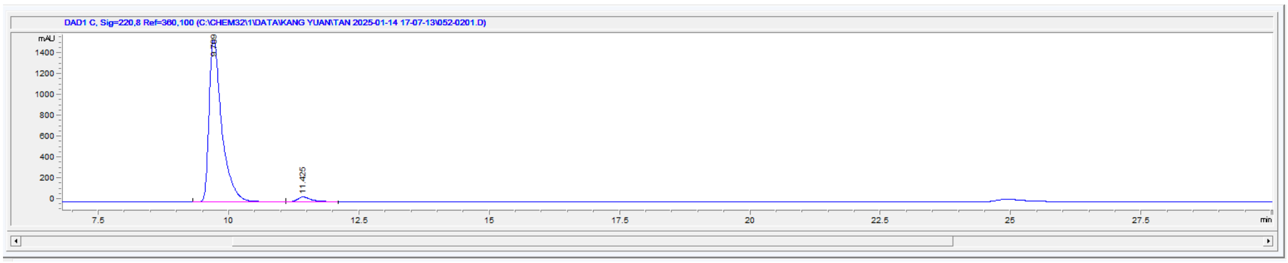

CHIRALPAK® IA, hexane/IPA = 97/3, 1 mL/min

#	Time	Area	Height	Width	Area%	Symmetry
1	9.709	26402.4	1556.9	0.2518	96.754	0.509
2	11.425	885.7	47.7	0.2706	3.246	0.61

[back to Table of Contents]

trimethyl (2S,3R)-3-(isopropylthio)-3-phenylcyclopropane-1,1,2-tricarboxylate (**3c**)

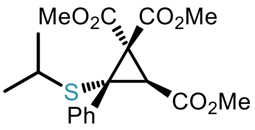

Racemic

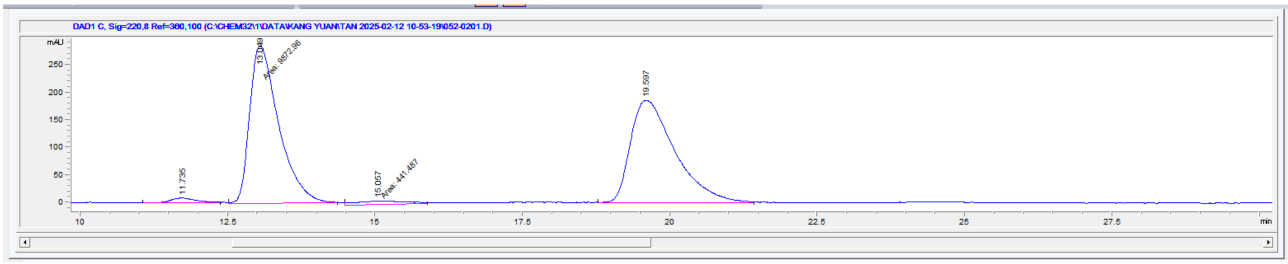

CHIRALPAK® OD, hexane/IPA = 98/2, 1 mL/min

#	Time	Area	Height	Width	Area%	Symmetry
1	11.735	300.5	9.2	0.3959	1.462	0.804
2	13.049	9873	287.1	0.5731	48.031	0.546
3	15.057	441.5	7	1.0472	2.148	0.713
4	19.597	9940.3	187.2	0.7495	48.359	0.513

Enantioenriched (93% ee)

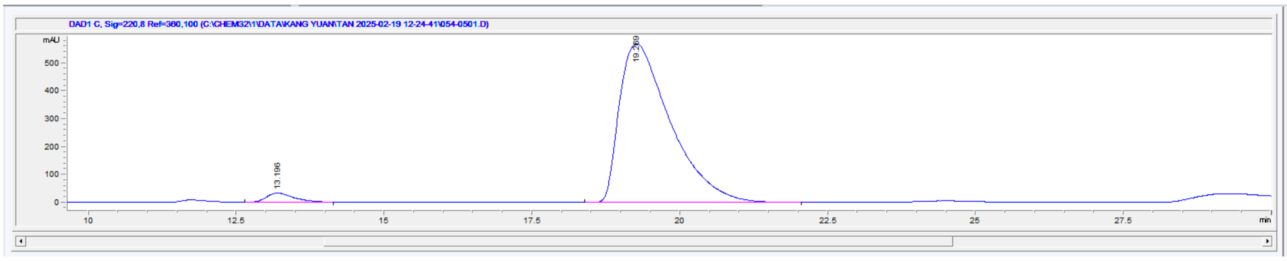

CHIRALPAK® OD, hexane/IPA = 98/2, 1 mL/min

#	Time	Area	Height	Width	Area%	Symmetry
1	13.196	1154.6	34	0.4926	3.265	0.619
2	19.269	34206.1	570.2	0.8819	96.735	0.476

[back to Table of Contents]

trimethyl (2S,3R)-3-(dodecylthio)-3-phenylcyclopropane-1,1,2-tricarboxylate (**3d**)

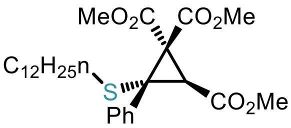

Racemic

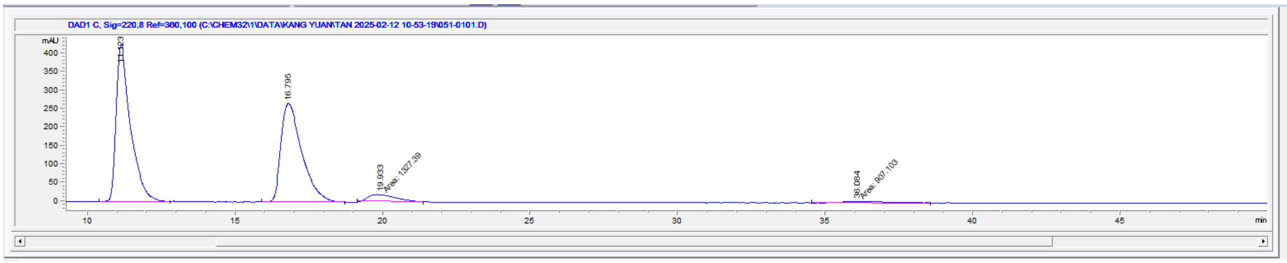

CHIRALPAK® OD, hexane/IPA = 98/2, 1 mL/min

#	Time	Area	Height	Width	Area%	Symmetry
1	11.123	14612	426.7	0.4995	48.646	0.472
2	16.795	13190.7	267.2	0.7138	43.914	0.513
3	19.933	1327.4	19.6	1.1281	4.419	0.737
4	36.084	907.1	6.1	2.46	3.020	0.433

Enantioenriched (93% ee)

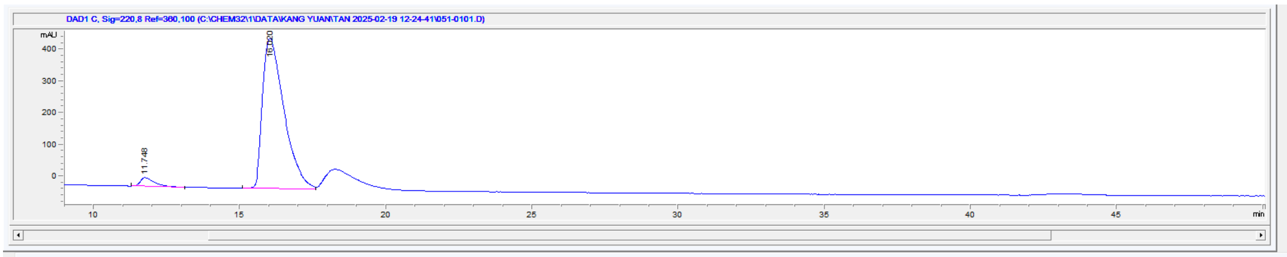

CHIRALPAK® OD, hexane/IPA = 98/2, 1 mL/min

#	Time	Area	Height	Width	Area%	Symmetry
1	11.748	942.1	27.6	0.4803	3.736	0.496
2	16.02	24273.1	474	0.7634	96.264	0.48

[back to Table of Contents]

trimethyl (2S,3R)-3-phenyl-3-((2-(trimethylsilyl)ethyl)thio)cyclopropane-1,1,2-tricarboxylate (**3e**)

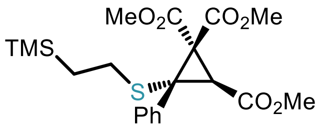

Racemic

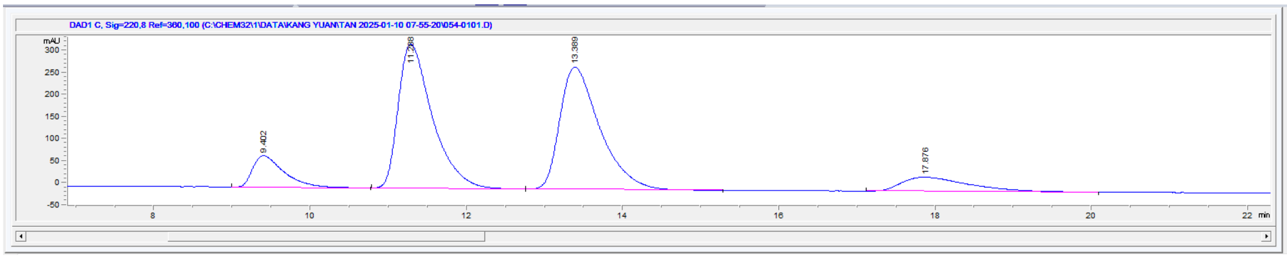

CHIRALPAK® OD, hexane/IPA = 97/3, 1 mL/min

#	Time	Area	Height	Width	Area%	Symmetry
1	9.402	2027.6	71.7	0.4278	8.541	0.481
2	11.288	9950.2	326.4	0.4597	41.916	0.546
3	13.389	9839.4	276.6	0.531	41.449	0.57
4	17.876	1921.3	32	0.8294	8.094	0.504

Enantioenriched (94% ee)

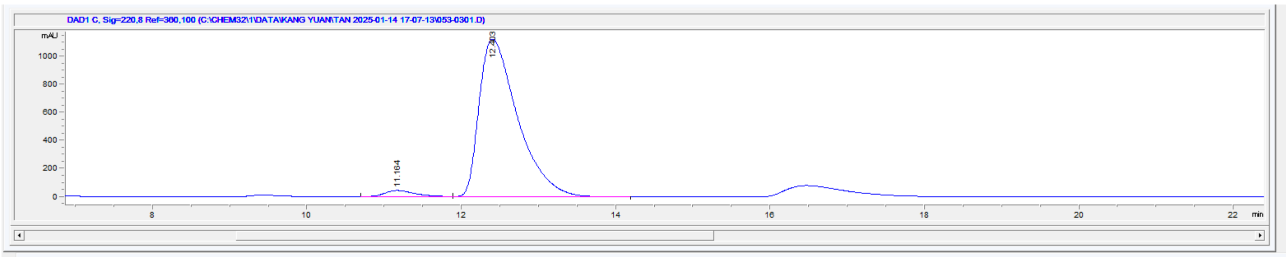

CHIRALPAK® OD, hexane/IPA = 97/3, 1 mL/min

#	Time	Area	Height	Width	Area%	Symmetry
1	11.164	1324.8	46	0.4237	3.281	0.664
2	12.403	39054.1	1124.5	0.5252	96.719	0.5

[back to Table of Contents]

trimethyl (2S,3R)-3-((3-butoxy-3-oxopropyl)thio)-3-phenylcyclopropane-1,1,2-tricarboxylate (**3f**)

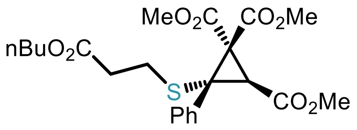

Racemic

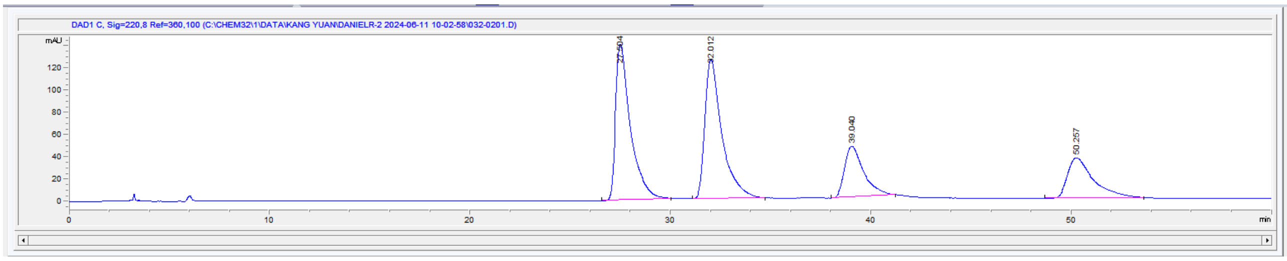

CHIRALPAK® IA, hexane/IPA = 98/2, 1 mL/min

#	Time	Area	Height	Width	Area%	Symmetry
1	27.504	7205.9	140.2	0.7536	34.697	0.456
2	32.012	7278.2	124.8	0.8545	35.046	0.489
3	39.04	3019.8	45.7	0.9288	14.541	0.556
4	50.257	3263.9	36.2	1.12	15.716	0.482

Enantioenriched (90% ee)

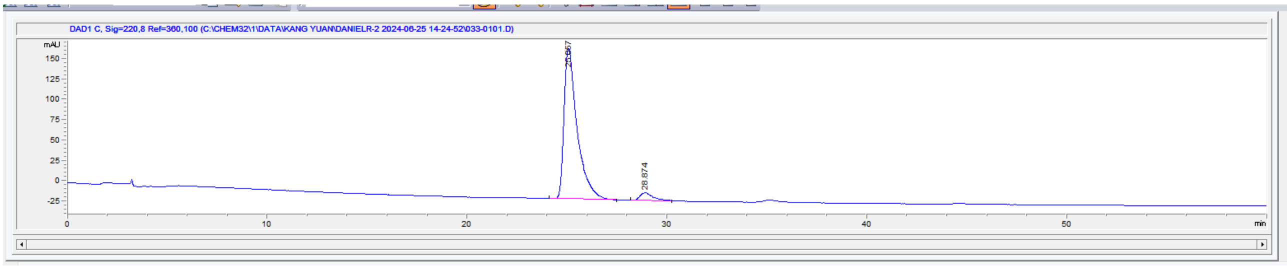

CHIRALPAK® IA, hexane/IPA = 98/2, 1 mL/min

#	Time	Area	Height	Width	Area%	Symmetry
1	25.057	8303	185.2	0.6591	94.980	0.454
2	28.874	438.8	9.4	0.6102	5.020	0.559

[back to Table of Contents]

trimethyl (2S,3R)-3-(phenethylthio)-3-phenylcyclopropane-1,1,2-tricarboxylate (**3g**)

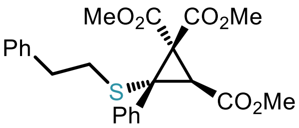

Racemic

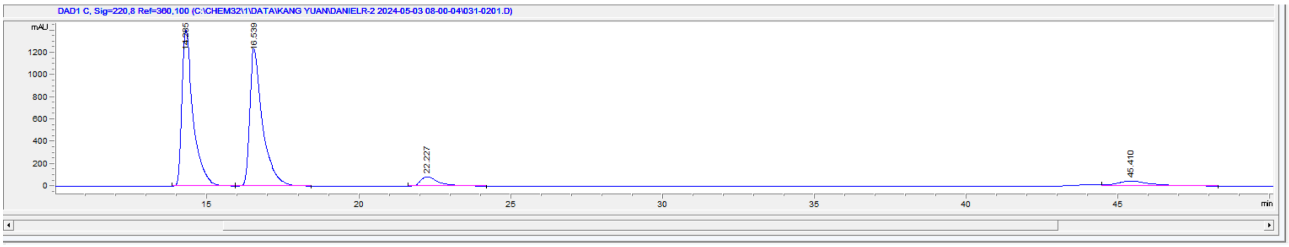

CHIRALPAK® IA, hexane/IPA = 97/3, 1 mL/min

#	Time	Area	Height	Width	Area%	Symmetry
1	14.285	36120.6	1399.2	0.3832	45.412	0.472
2	16.539	36450	1233.7	0.4367	45.826	0.488
3	22.227	3338.2	83.9	0.5841	4.197	0.51
4	45.41	3630.6	44.8	1.1223	4.565	0.662

Enantioenriched (94% ee)

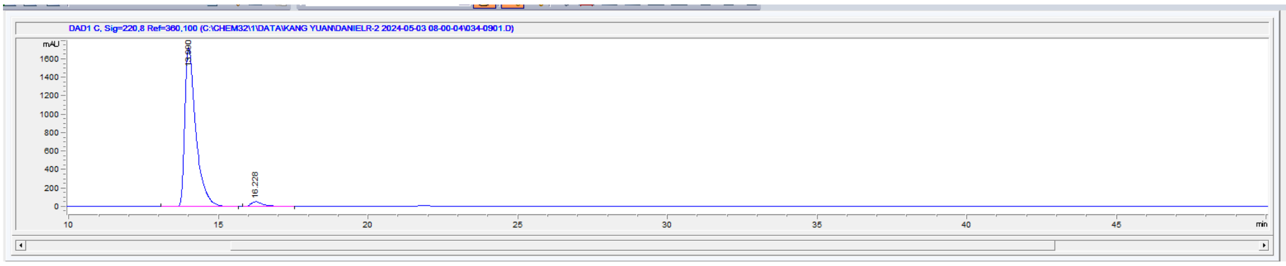

CHIRALPAK® IA, hexane/IPA = 97/3, 1 mL/min

#	Time	Area	Height	Width	Area%	Symmetry
1	13.99	43471.4	1723.7	0.3702	96.793	0.483
2	16.228	1440.5	52.2	0.4022	3.207	0.572

[back to Table of Contents]

trimethyl (2S,3R)-3-((4-methoxybenzyl)thio)-3-phenylcyclopropane-1,1,2-tricarboxylate (**3h**)

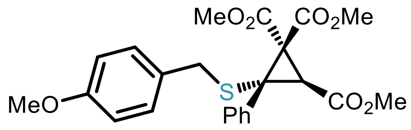

Racemic

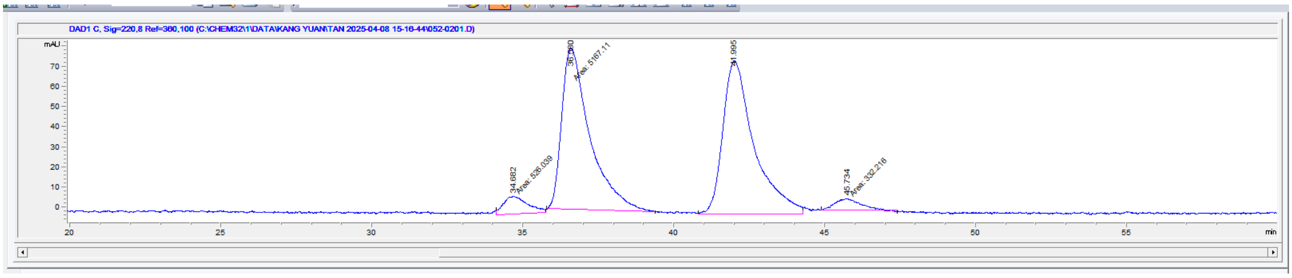

CHIRALPAK® IA, hexane/IPA = 97/3, 1 mL/min

#	Time	Area	Height	Width	Area%	Symmetry
1	34.682	526	9.1	0.9587	4.569	0.675
2	36.58	5167.1	79.8	1.079	44.875	0.465
3	41.995	5489.1	76.3	0.8651	47.671	0.548
4	45.734	332.2	5.6	0.9848	2.885	0.859

Enantioenriched (94% ee)

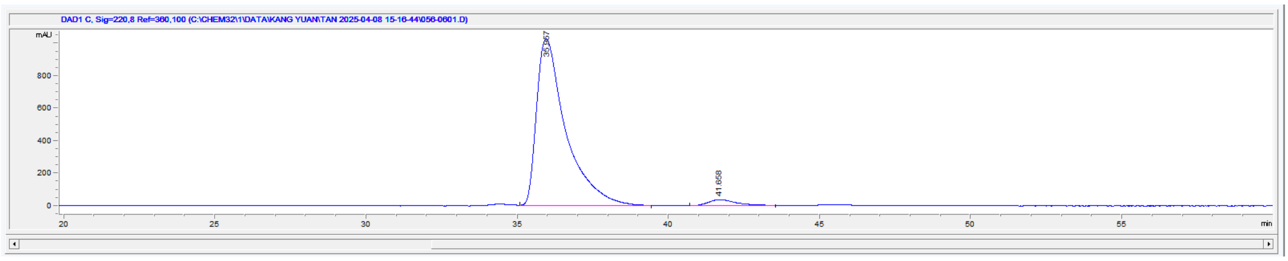

CHIRALPAK® IA, hexane/IPA = 97/3, 1 mL/min

#	Time	Area	Height	Width	Area%	Symmetry
1	35.957	69731	1028	0.9234	96.178	0.46
2	41.658	2770.8	38.4	0.8848	3.822	0.519

[back to Table of Contents]

trimethyl (2S,3R)-3-(benzylthio)-3-phenylcyclopropane-1,1,2-tricarboxylate (**3i**)

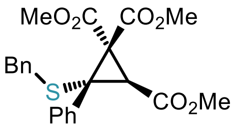

Racemic

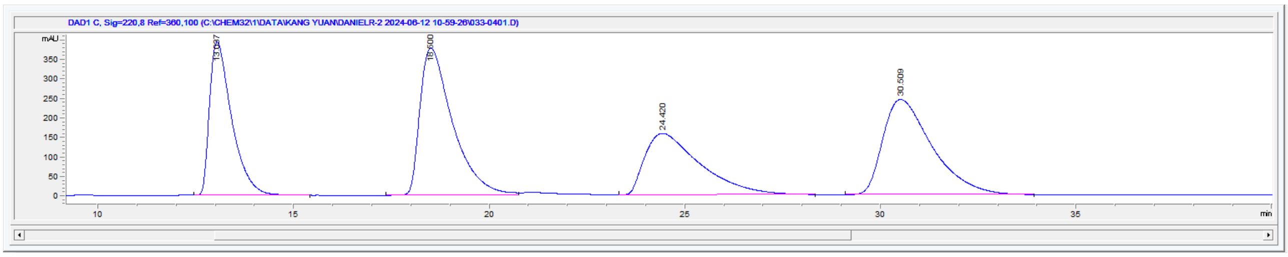

CHIRALPAK® OD, hexane/IPA = 97/3, 1 mL/min

#	Time	Area	Height	Width	Area%	Symmetry
1	13.037	15581.2	393.5	0.5959	20.914	0.479
2	18.5	21936.2	377.6	0.8698	29.443	0.457
3	24.42	15552.7	157.6	1.4192	20.875	0.431
4	30.509	21433	244.1	1.3124	28.768	0.529

Enantioenriched (93% ee)

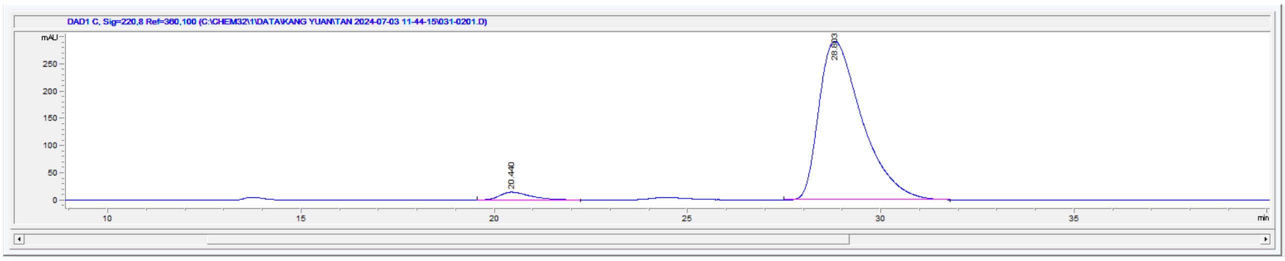

CHIRALPAK® OD, hexane/IPA = 97/3, 1 mL/min

#	Time	Area	Height	Width	Area%	Symmetry
1	20.44	856.5	14.8	0.7446	3.639	0.589
2	28.803	22682.4	291.9	1.1545	96.361	0.543

[back to Table of Contents]

trimethyl (2S,3R)-3-((3,5-difluorobenzyl)thio)-3-phenylcyclopropane-1,1,2-tricarboxylate (**3j**)

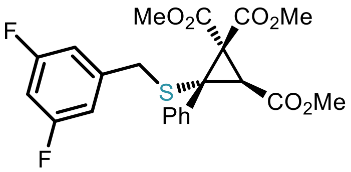

Racemic

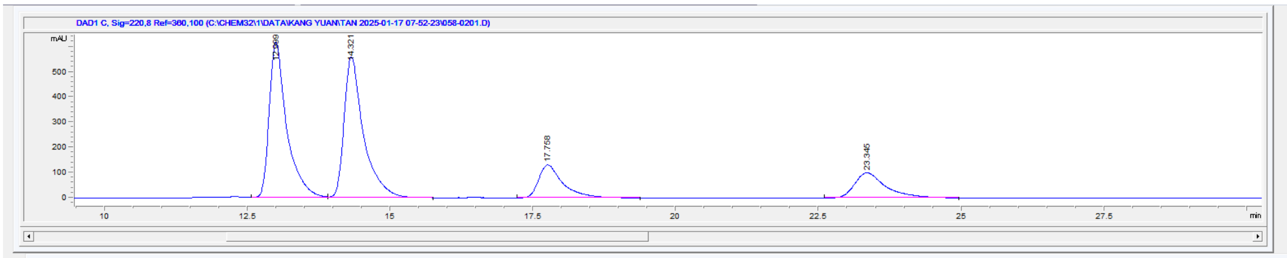

CHIRALPAK® IA, hexane/IPA = 97/3, 1 mL/min

#	Time	Area	Height	Width	Area%	Symmetry
1	12.999	13268.6	619.5	0.3159	38.611	0.553
2	14.321	13317.5	560.2	0.3493	38.753	0.544
3	17.758	3913.4	131.2	0.44	11.388	0.534
4	23.345	3865.6	100.2	0.5581	11.249	0.586

Enantioenriched (86% ee)

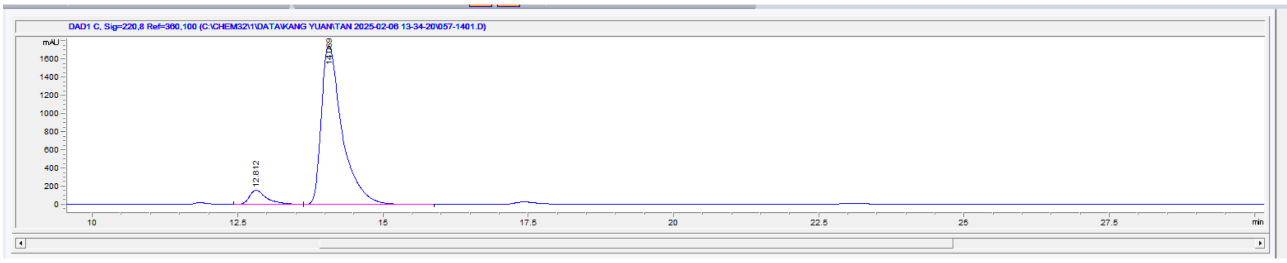

CHIRALPAK® IA, hexane/IPA = 97/3, 1 mL/min

#	Time	Area	Height	Width	Area%	Symmetry
1	12.812	3390.3	158.4	0.3117	7.195	0.565
2	14.069	43729.6	1746.3	0.3761	92.805	0.53

[back to Table of Contents]

trimethyl (2S,3R)-3-((3-fluorobenzyl)thio)-3-phenylcyclopropane-1,1,2-tricarboxylate (**3k**)

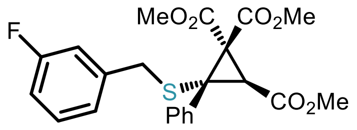

Racemic

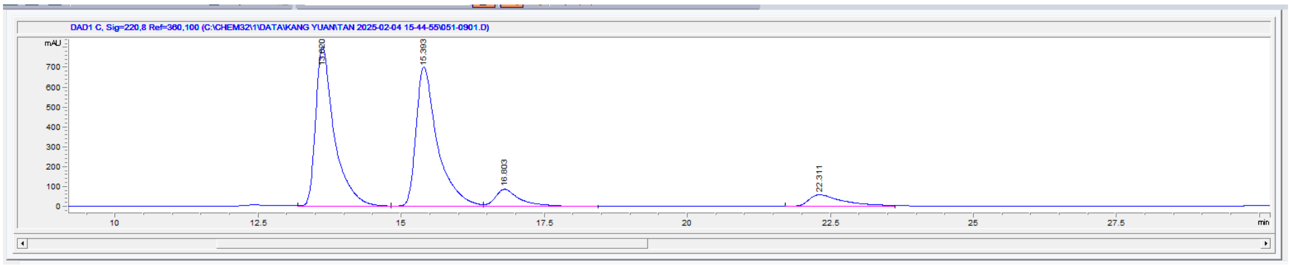

CHIRALPAK® IA, hexane/IPA = 95/5, 1 mL/min

#	Time	Area	Height	Width	Area%	Symmetry
1	13.62	18393.3	805.3	0.3345	43.712	0.525
2	15.393	18378.7	705.3	0.382	43.677	0.531
3	16.803	2708.7	87	0.4472	6.437	0.528
4	22.311	2597.9	59.4	0.6105	6.174	0.421

Enantioenriched (90% ee)

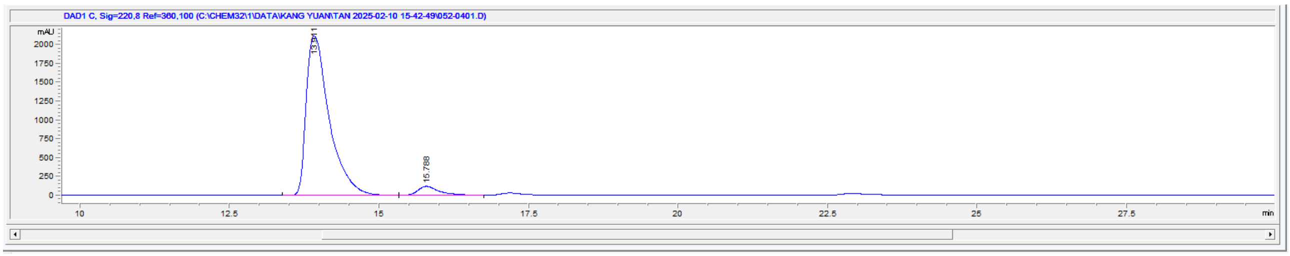

CHIRALPAK® IA, hexane/IPA = 95/5, 1 mL/min

#	Time	Area	Height	Width	Area%	Symmetry
1	13.911	56785.3	2121.8	0.3882	94.697	0.484
2	15.788	3179.7	121.3	0.3817	5.303	0.587

[back to Table of Contents]

trimethyl (2S,3R)-3-((3-methoxyphenyl)thio)-3-phenylcyclopropane-1,1,2-tricarboxylate (**3l**)

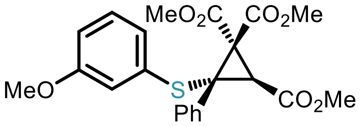

Racemic

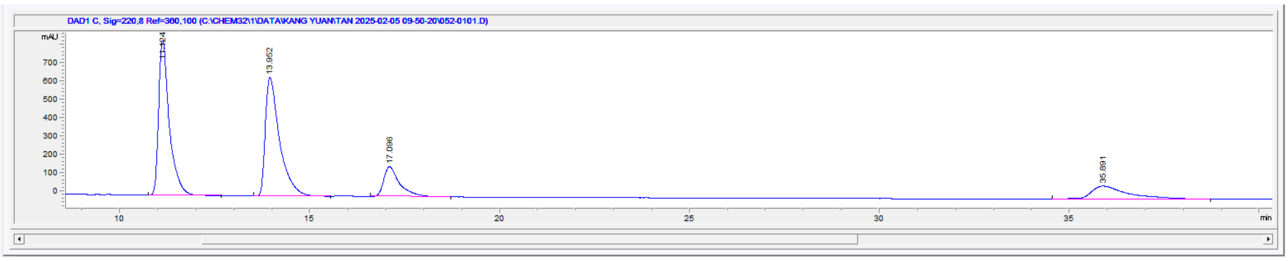

CHIRALPAK® IA, hexane/IPA = 95/5, 1 mL/min

#	Time	Area	Height	Width	Area%	Symmetry
1	11.124	16517.4	844.6	0.2902	38.336	0.548
2	13.952	16831	645.9	0.388	39.064	0.446
3	17.096	4772.5	163	0.4296	11.077	0.514
4	35.891	4964.5	71.3	0.9438	11.522	0.442

Enantioenriched (85% ee)

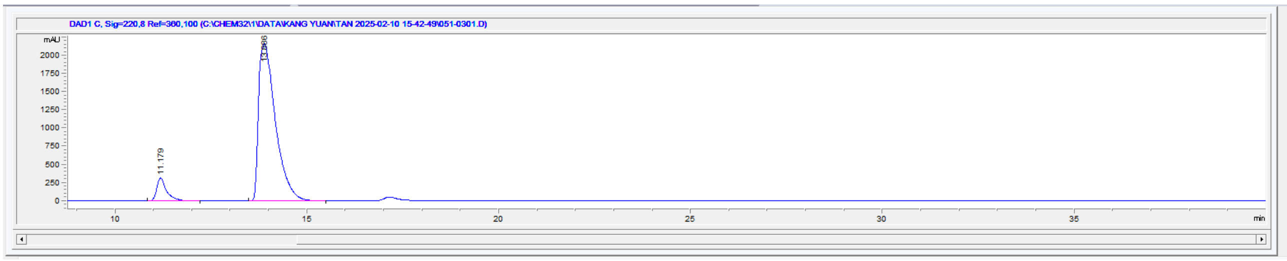

CHIRALPAK® IA, hexane/IPA = 95/5, 1 mL/min

#	Time	Area	Height	Width	Area%	Symmetry
1	11.179	5662.5	317.3	0.2602	7.883	0.575
2	13.886	66171.3	2169.9	0.4158	92.117	0.467

[back to Table of Contents]

trimethyl (2S,3R)-3-(naphthalen-2-ylthio)-3-phenylcyclopropane-1,1,2-tricarboxylate (**3m**)

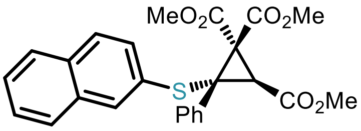

Racemic

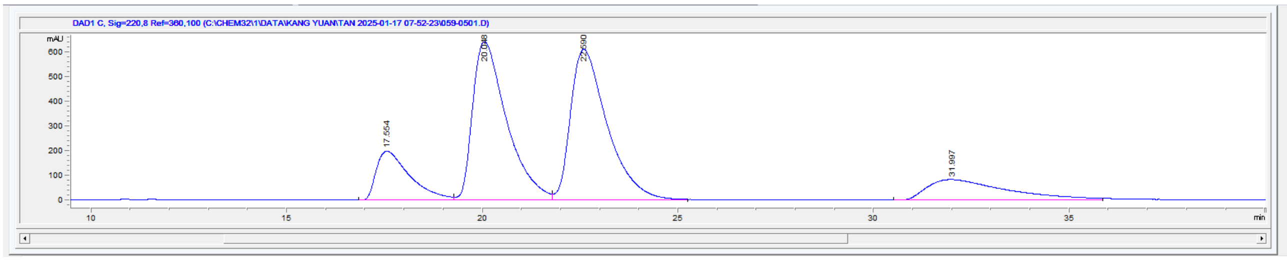

CHIRALPAK® OD, hexane/IPA = 97/3, 1 mL/min

#	Time	Area	Height	Width	Area%	Symmetry
1	17.554	11859	198.5	0.8629	11.556	0.427
2	20.048	38917.9	642.9	0.8843	37.923	0.497
3	22.59	39975.5	611.5	0.9307	38.954	0.534
4	31.997	11870.7	83.5	1.6756	11.567	0.432

Enantioenriched (80% ee)

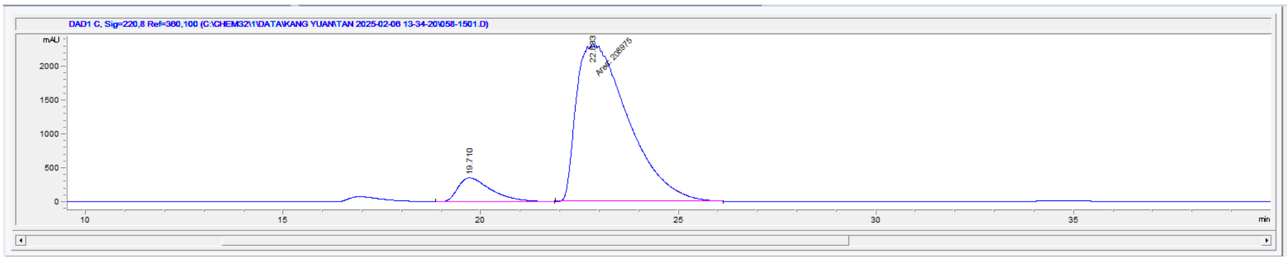

CHIRALPAK® OD, hexane/IPA = 97/3, 1 mL/min

#	Time	Area	Height	Width	Area%	Symmetry
1	19.71	21174.1	355.4	0.821	9.019	0.491
2	22.833	213608.1	2340	1.5214	90.981	0.442

[back to Table of Contents]

trimethyl (2S,3R)-3-phenyl-3-(phenylthio)cyclopropane-1,1,2-tricarboxylate (**3n**)

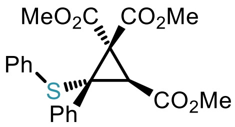

Racemic

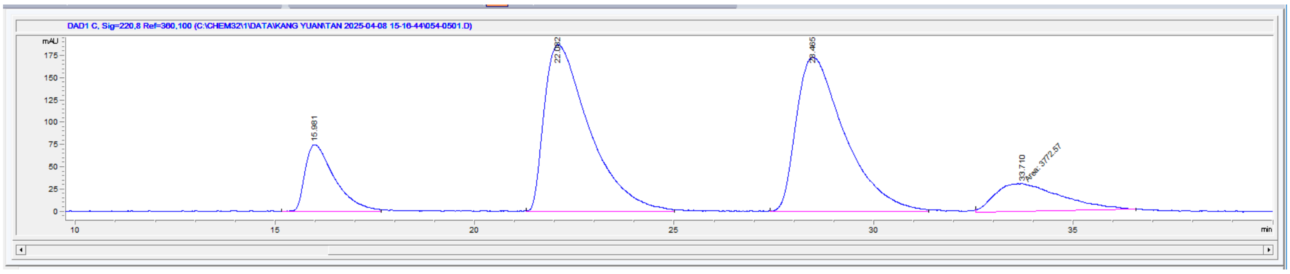

CHIRALPAK® OD, hexane/IPA = 98/2, 1 mL/min

#	Time	Area	Height	Width	Area%	Symmetry
1	15.981	3940.9	75.4	0.7416	10.562	0.442
2	22.082	14783.8	188.1	1.0649	39.624	0.402
3	28.465	14813.3	174.1	1.0244	39.703	0.48
4	33.71	3772.6	32.1	1.9577	10.111	0.651

Enantioenriched (87% ee)

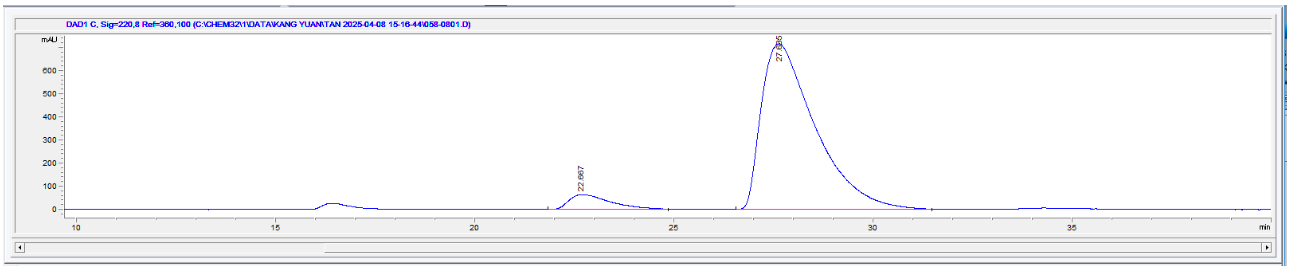

CHIRALPAK® OD, hexane/IPA = 98/2, 1 mL/min

#	Time	Area	Height	Width	Area%	Symmetry
1	22.667	4859.5	65.6	0.9049	6.703	0.402
2	27.635	67642.4	718	1.1851	93.297	0.481

[back to Table of Contents]

trimethyl (2S,3R)-3-(propylthio)-3-(p-tolyl)cyclopropane-1,1,2-tricarboxylate (**3o**)

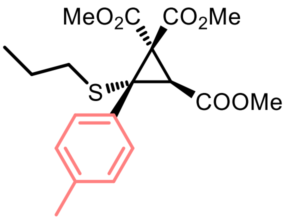

Racemic

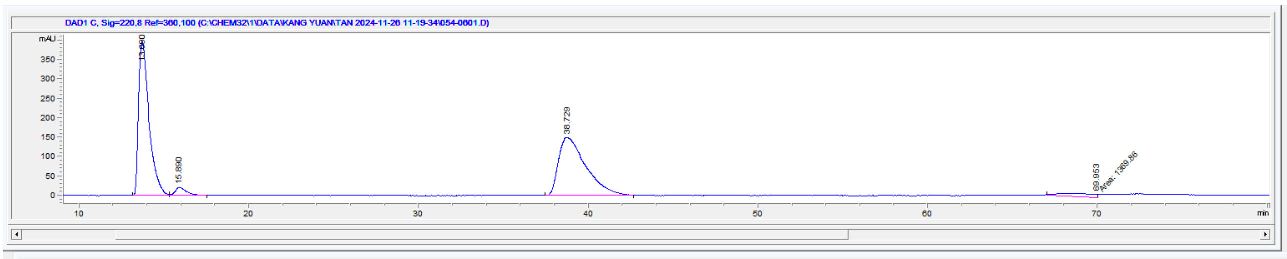

CHIRALPAK® OD, hexane/IPA = 99/1, 1 mL/min

#	Time	Area	Height	Width	Area%	Symmetry
1	13.69	16864	396.1	0.6427	46.583	0.452
2	15.89	1042.4	21.6	0.6147	2.879	0.548
3	38.729	16926	150.2	1.499	46.754	0.423
4	69.953	1369.9	10.6	2.1535	3.784	33.347

Enantioenriched (94% ee)

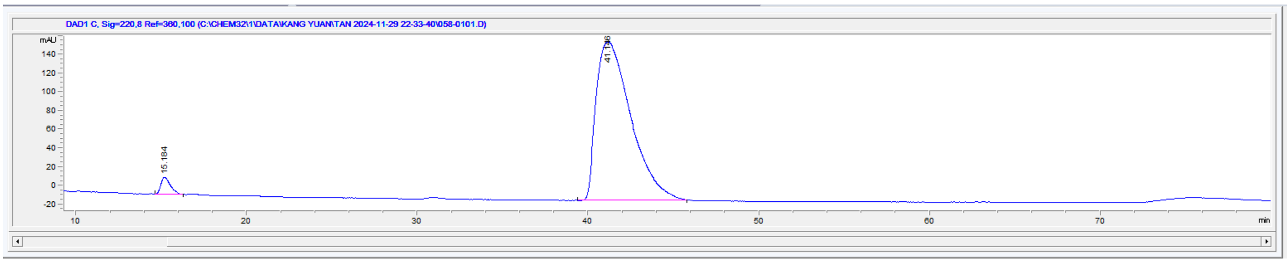

CHIRALPAK® OD, hexane/IPA = 99/1, 1 mL/min

#	Time	Area	Height	Width	Area%	Symmetry
1	15.18	534.2	13.8	0.5283	2.849	0.573
2	41.135	18220.5	128	1.6752	97.151	0.477

[back to Table of Contents]

trimethyl (2S,3R)-3-(propylthio)-3-(o-tolyl)cyclopropane-1,1,2-tricarboxylate (**3p**)

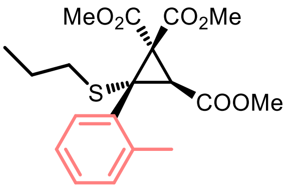

Racemic

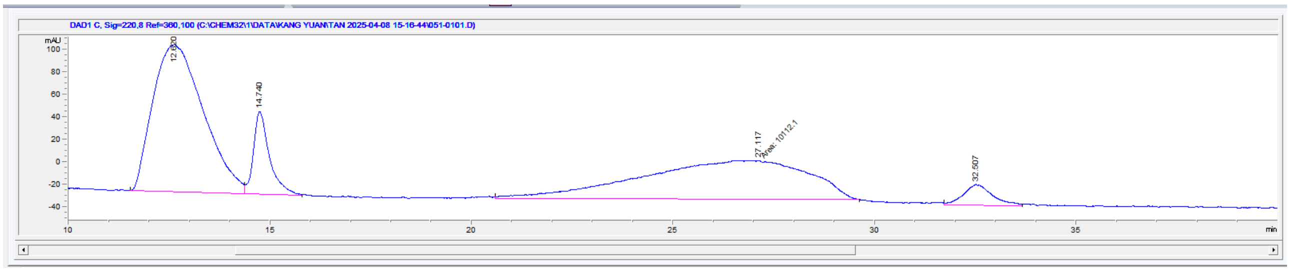

CHIRALPAK® AD-H, hexane/IPA = 98/2, 1 mL/min

#	Time	Area	Height	Width	Area%	Symmetry
1	12.62	11447.6	131.2	1.0357	46.563	0.7
2	14.74	2086.5	73.8	0.4057	8.487	0.582
3	27.117	10112.1	34.9	4.8298	41.131	2.206
4	32.507	938.8	18.6	0.6141	3.819	0.727

Enantioenriched (92% ee)

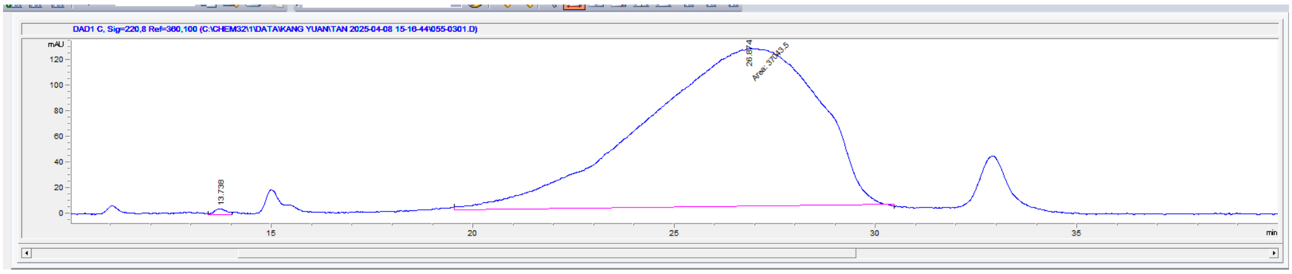

CHIRALPAK® AD-H, hexane/IPA = 98/2, 1 mL/min

#	Time	Area	Height	Width	Area%	Symmetry
1	13.738	102.1	4.7	0.2669	0.275	1.051
2	26.874	37043.5	123.2	5.0121	99.725	1.51

[back to Table of Contents]

trimethyl (2S,3R)-3-(propylthio)-3-(m-tolyl)cyclopropane-1,1,2-tricarboxylate (**3q**)

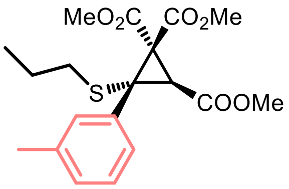

Racemic

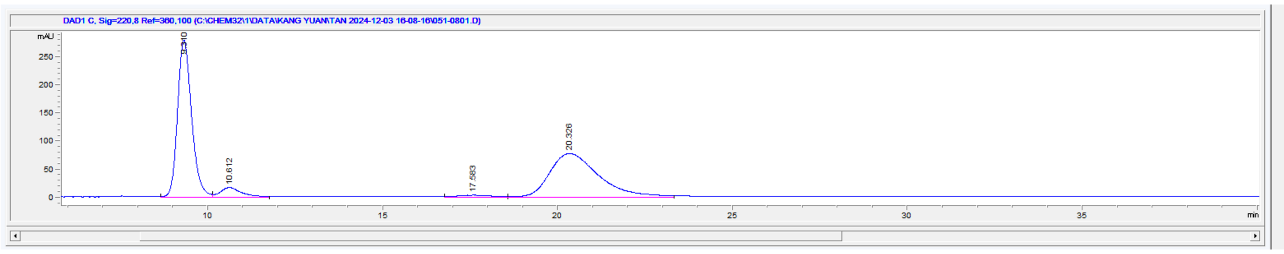

CHIRALPAK® AS-H, hexane/IPA = 98/2, 1 mL/min

#	Time	Area	Height	Width	Area%	Symmetry
1	9.31	7876.9	280.7	0.4273	46.777	0.762
2	10.612	805.2	18	0.602	4.782	0.622
3	17.583	336.9	4.4	0.901	2.001	0.828
4	20.326	7820.4	78.6	1.1725	46.441	0.637

Enantioenriched (93% ee)

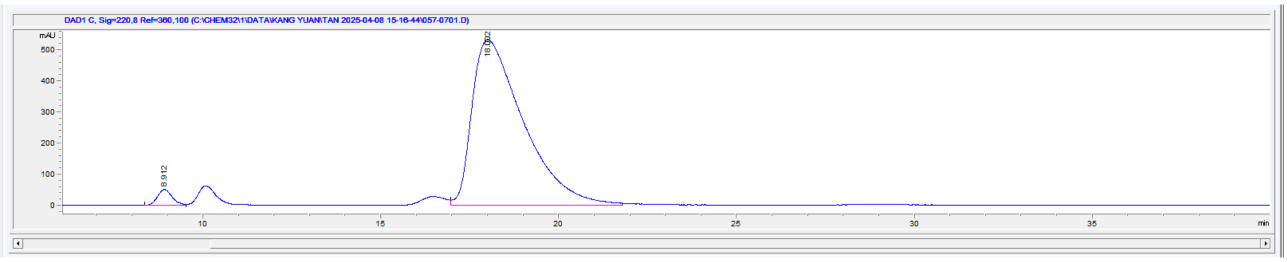

#	Time	Area	Height	Width	Area%	Symmetry
1	8.912	1521.5	51.9	0.4199	2.746	0.855
2	18.002	53886	534.4	1.2044	97.254	0.425

CHIRALPAK® AS-H, hexane/IPA = 98/2, 1 mL/min

[back to Table of Contents]

trimethyl (2S,3R)-3-(4-fluorophenyl)-3-(propylthio)cyclopropane-1,1,2-tricarboxylate (**3r**)

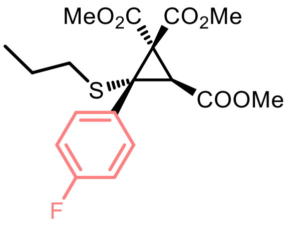

Racemic

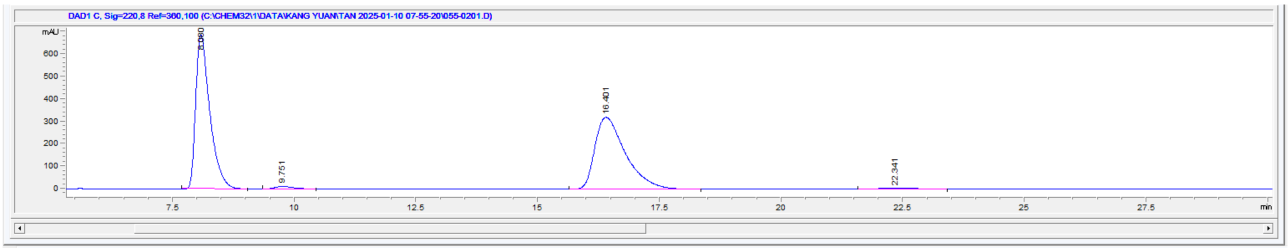

CHIRALPAK® OD, hexane/IPA = 97/3, 1 mL/min

#	Time	Area	Height	Width	Area%	Symmetry
1	8.08	13869.4	684.6	0.3043	48.721	0.544
2	9.751	317.7	12.2	0.3618	1.116	0.595
3	16.401	13970	320.1	0.6571	49.075	0.53
4	22.341	309.7	4.7	0.7818	1.088	0.636

Enantioenriched (91% ee)

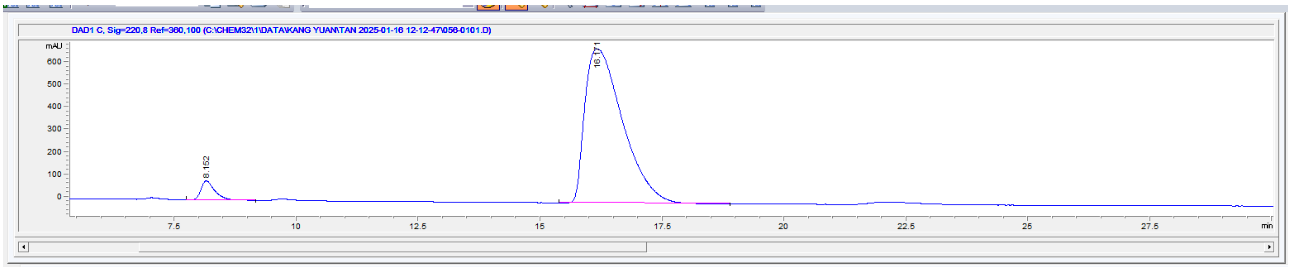

CHIRALPAK® OD, hexane/IPA = 97/3, 1 mL/min

#	Time	Area	Height	Width	Area%	Symmetry
1	8.152	1678.3	85.1	0.292	4.334	0.575
2	16.171	37041.1	688.2	0.8039	95.666	0.466

[back to Table of Contents]

trimethyl (2S,3R)-3-(4-chlorophenyl)-3-(propylthio)cyclopropane-1,1,2-tricarboxylate (**3s**)

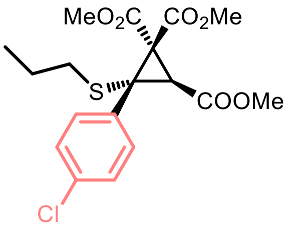

Racemic

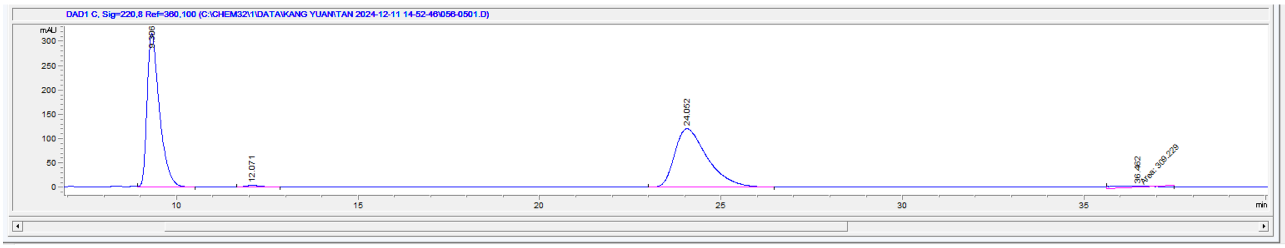

CHIRALPAK® OD, hexane/IPA = 97/3, 1 mL/min

#	Time	Area	Height	Width	Area%	Symmetry
1	9.306	7540.5	316	0.3584	48.546	0.554
2	12.071	146.3	4.1	0.4407	0.942	0.639
3	24.052	7536.6	120.2	0.9094	48.521	0.571
4	36.462	309.2	3.4	1.0825	1.991	0

Enantioenriched (92% ee)

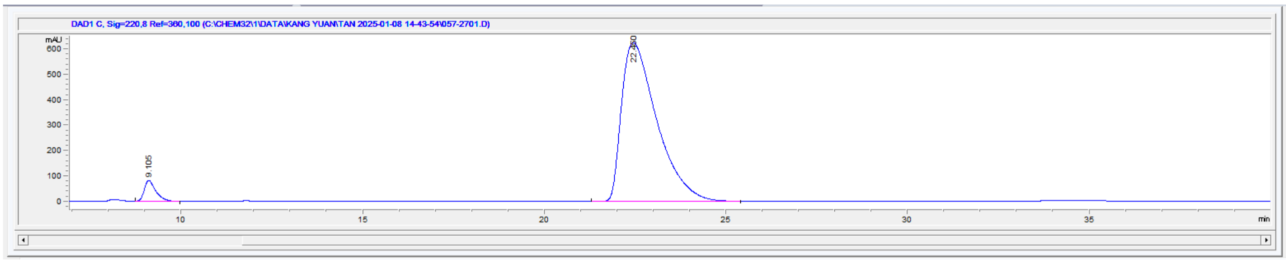

CHIRALPAK® OD, hexane/IPA = 97/3, 1 mL/min

#	Time	Area	Height	Width	Area%	Symmetry
1	9.105	1885.2	83	0.3331	4.250	0.594
2	22.45	42470.1	626.4	0.9671	95.750	0.473

[back to Table of Contents]

trimethyl (2S,3R)-3-(propylthio)-3-(4-(trifluoromethyl)phenyl)cyclopropane-1,1,2-tricarboxylateate (**3t**)

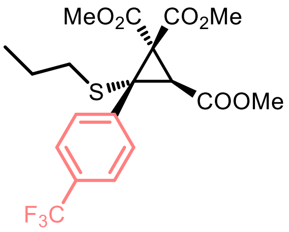

Racemic

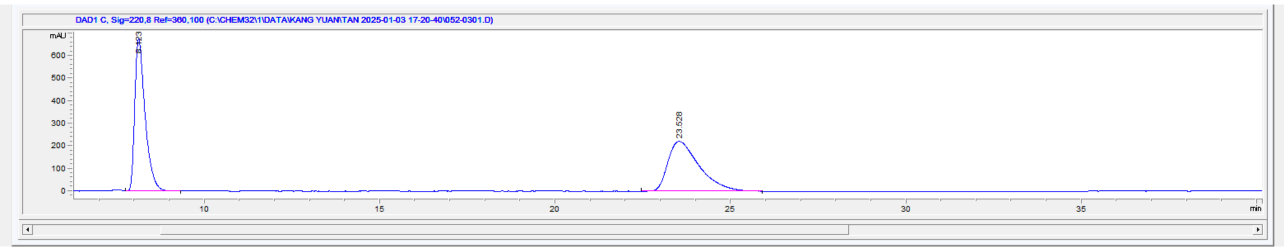

CHIRALPAK® OD, hexane/IPA = 98/2, 1 mL/min

#	Time	Area	Height	Width	Area%	Symmetry
1	8.123	13852.5	675	0.3074	50.122	0.552
2	23.528	13785	221.1	0.9132	49.878	0.527

Enantioenriched (84% ee)

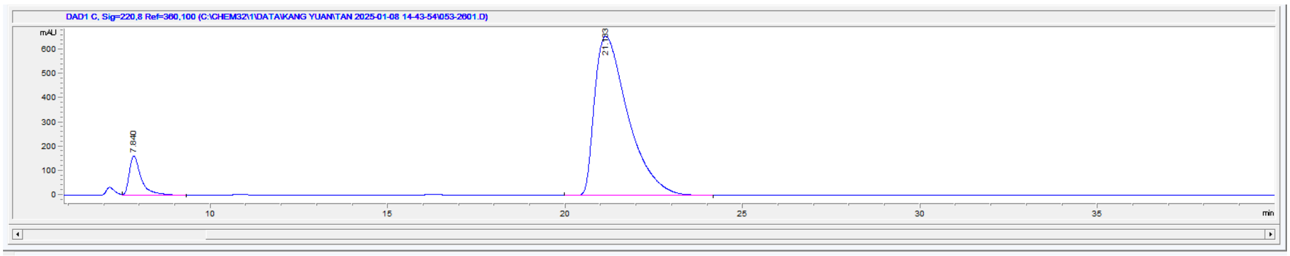

CHIRALPAK® OD, hexane/IPA = 98/2, 1 mL/min

#	Time	Area	Height	Width	Area%	Symmetry
1	7.84	3916.2	163.9	0.3568	8.338	0.54
2	21.133	43051.3	658.9	0.9222	91.662	0.461

[back to Table of Contents]

trimethyl (2S,3R)-3-(4-(tert-butyl)phenyl)-3-(propylthio)cyclopropane-1,1,2-tricarboxylate (**3u**)

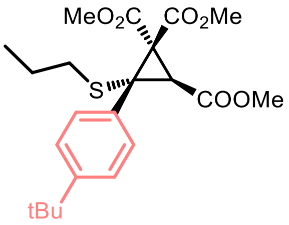

Racemic

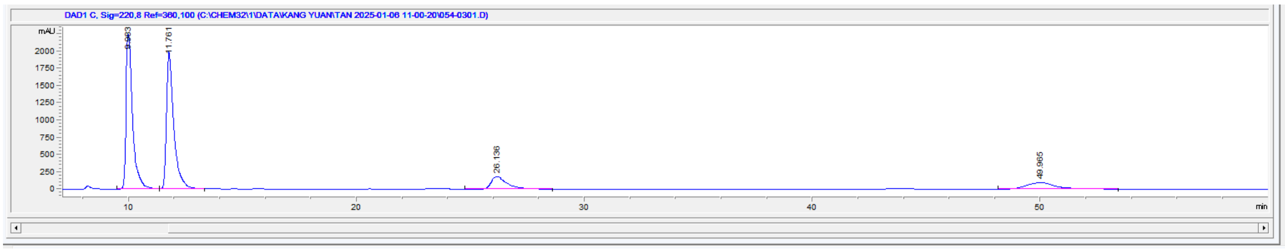

CHIRALPAK® AD-H, hexane/IPA = 98/2, 1 mL/min

#	Time	Area	Height	Width	Area%	Symmetry
1	9.983	44610.1	2244.5	0.2979	41.416	0.534
2	11.761	45257.2	2000.5	0.3359	42.017	0.499
3	26.136	8962.7	183.5	0.7178	8.321	0.515
4	49.965	8881.6	96.7	1.1196	8.246	0.785

Enantioenriched (93% ee)

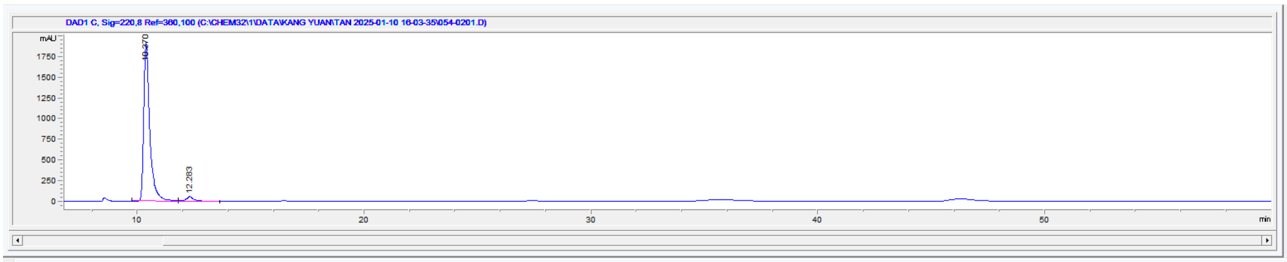

CHIRALPAK® AD-H, hexane/IPA = 98/2, 1 mL/min

#	Time	Area	Height	Width	Area%	Symmetry
1	10.37	37036.9	1923.5	0.2906	96.160	0.595
2	12.283	1479	56.6	0.3687	3.840	0.772

[back to Table of Contents]

1,1-dibenzyl 2-methyl (2S,3R)-3-phenyl-3-(propylthio)cyclopropane-1,1,2-tricarboxylate (**3v**)

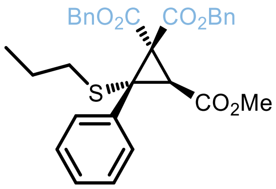

Racemic

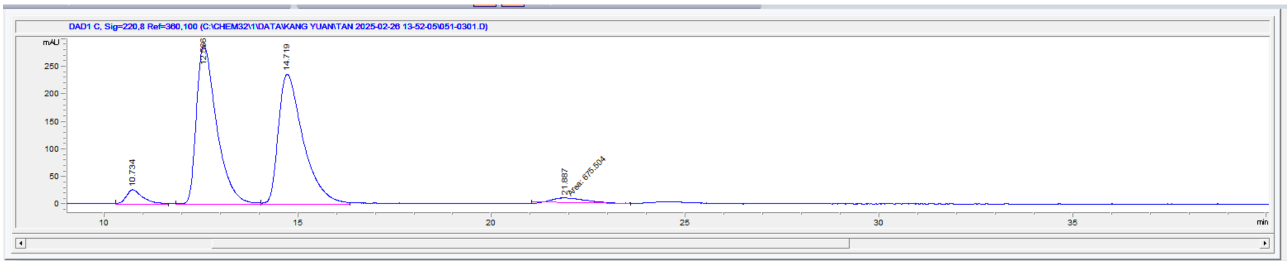

CHIRALPAK® OD, hexane/IPA = 95/5, 1 mL/min

#	Time	Area	Height	Width	Area%	Symmetry
1	10.734	840.3	26.8	0.4233	3.657	0.587
2	12.566	10568.9	289.3	0.5442	45.997	0.533
3	14.719	10611.4	236.4	0.6717	46.182	0.513
4	21.888	956.6	12.1	0.9478	4.163	0.706

Enantioenriched (91% ee)

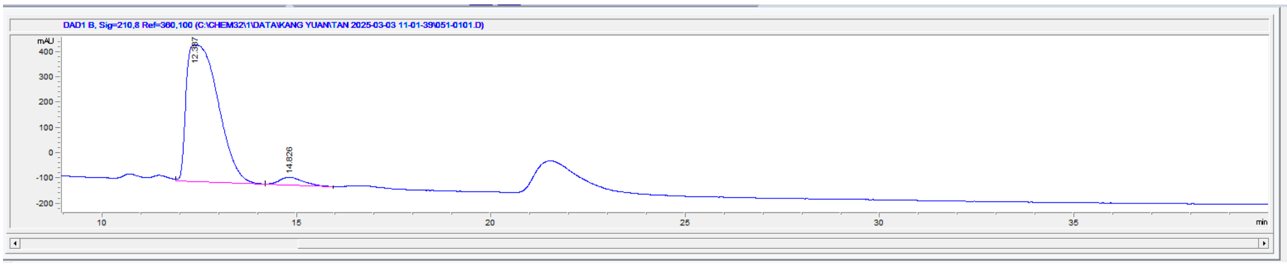

CHIRALPAK® OD, hexane/IPA = 95/5, 1 mL/min

#	Time	Area	Height	Width	Area%	Symmetry
1	12.387	30777.7	541.9	0.6786	95.691	0.368
2	14.826	1386	32.2	0.5189	4.309	0.72

[back to Table of Contents]

1,1-diethyl 2-methyl (2S,3R)-3-phenyl-3-(propylthio)cyclopropane-1,1,2-tricarboxylate (**3w**)

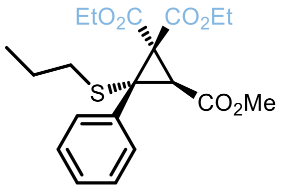

Racemic

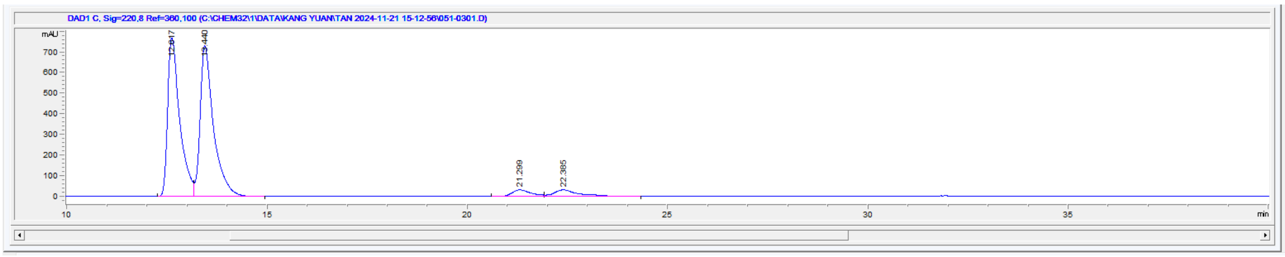

CHIRALPAK® OD, hexane/IPA = 98/2, 1 mL/min

#	Time	Area	Height	Width	Area%	Symmetry
1	12.617	16110.5	770.3	0.308	45.291	0.494
2	13.44	16958	730.3	0.3409	47.674	0.499
3	21.299	1067.7	32	0.5012	3.002	0.62
4	22.385	1434.8	32.3	0.6227	4.034	0.511

Enantioenriched (94% ee)

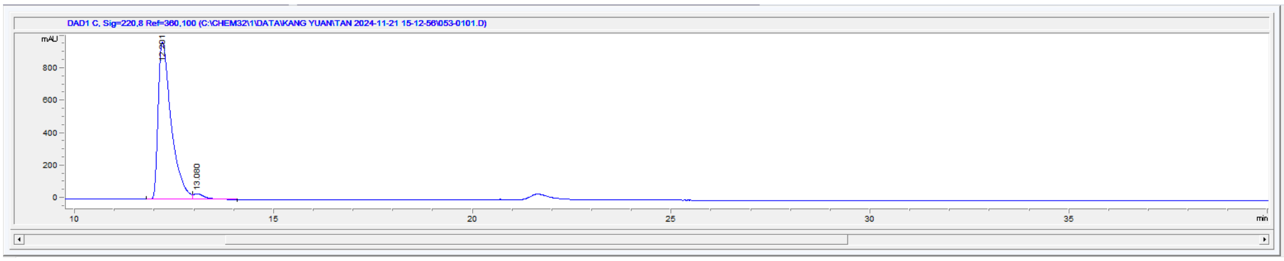

CHIRALPAK® OD, hexane/IPA = 98/2, 1 mL/min

#	Time	Area	Height	Width	Area%	Symmetry
1	12.201	22154.1	970.9	0.3422	96.940	0.436
2	13.08	699.4	34.4	0.2915	3.060	0.442

[back to Table of Contents]

2-ethyl 1,1-dimethyl (2S,3R)-3-phenyl-3-(propylthio)cyclopropane-1,1,2-tricarboxylate (**3x**)

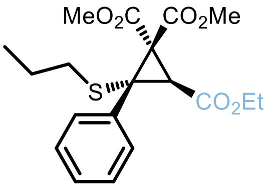

Racemic

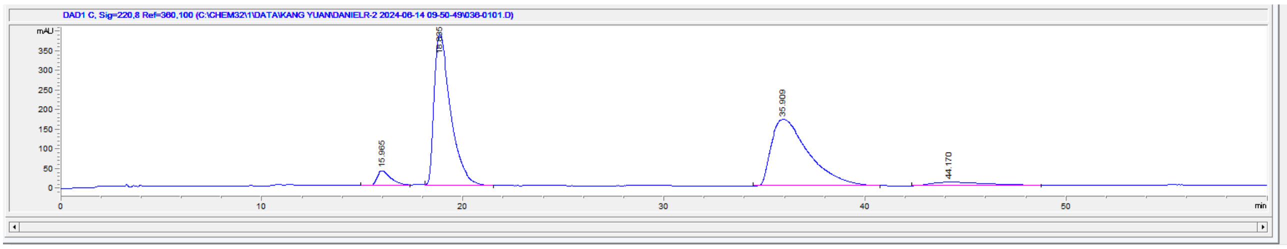

CHIRALPAK® OD, hexane/IPA = 99/1, 1 mL/min

#	Time	Area	Height	Width	Area%	Symmetry
1	15.965	1816.4	37.6	0.7215	3.847	0.519
2	18.835	21949.4	385.5	0.8542	46.488	0.477
3	35.909	21791.9	169.3	1.8478	46.154	0.44
4	44.17	1658	9	2.1657	3.512	0.474

Enantioenriched (94% ee)

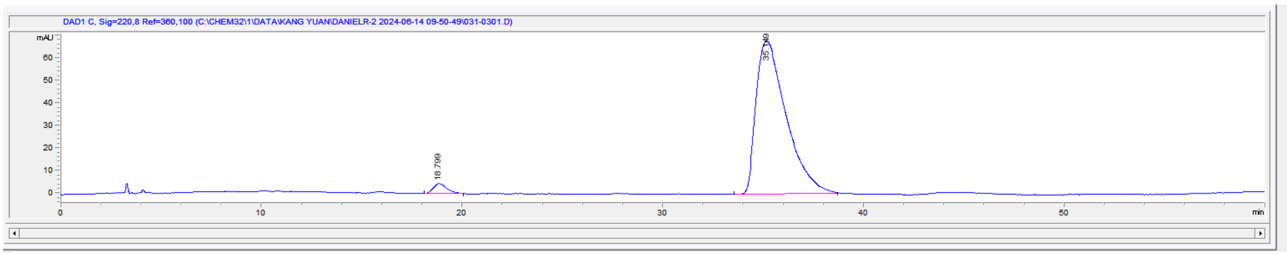

CHIRALPAK® OD, hexane/IPA = 99/1, 1 mL/min

#	Time	Area	Height	Width	Area%	Symmetry
1	18.799	213.7	4.4	0.5864	2.927	0.57
2	35.149	7087	67.9	1.2877	97.073	0.47

[back to Table of Contents]

2-hexyl 1,1-dimethyl (2S,3R)-3-phenyl-3-(propylthio)cyclopropane-1,1,2-tricarboxylate (**3y**)

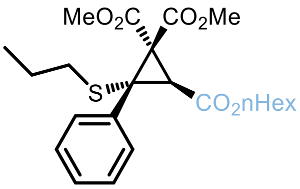

Racemic

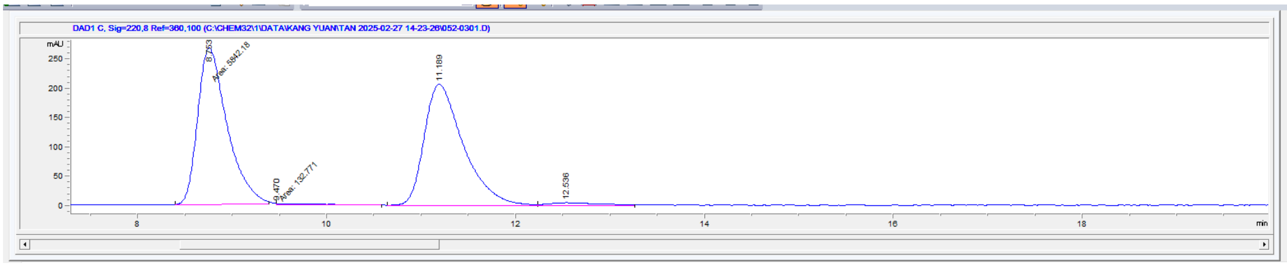

CHIRALPAK® OD, hexane/IPA = 97/3, 1 mL/min

#	Time	Area	Height	Width	Area%	Symmetry
1	8.753	5919.3	269	0.3667	47.887	0.615
2	9.947	200.7	3.9	0.8596	1.624	0.602
3	11.189	6024.4	207.5	0.4347	48.737	0.583
4	12.536	216.6	5.5	0.4733	1.752	0.502

Enantioenriched (94% ee)

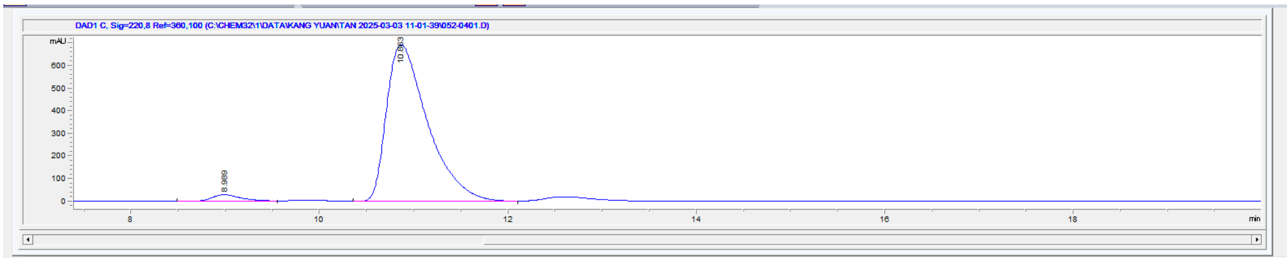

CHIRALPAK® OD, hexane/IPA = 97/3, 1 mL/min

#	Time	Area	Height	Width	Area%	Symmetry
1	8.989	699	29.5	0.3402	3.204	0.692
2	10.863	21118.7	693.9	0.4651	96.796	0.504

[back to Table of Contents]

2-isobutyl 1,1-dimethyl (2S,3R)-3-phenyl-3-(propylthio)cyclopropane-1,1,2-tricarboxylate (**3z**)

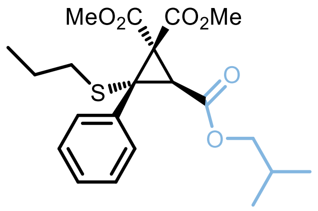

Racemic

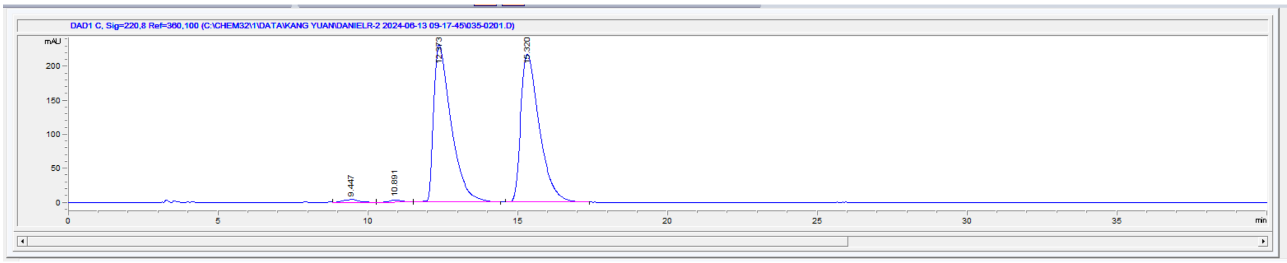

CHIRALPAK® OD, hexane/IPA = 99/1, 1 mL/min

#	Time	Area	Height	Width	Area%	Symmetry
1	9.447	182.6	4.6	0.5179	0.945	1.092
2	10.891	131.2	3.9	0.4145	0.680	0.749
3	12.373	9587.6	231.7	0.6288	49.656	0.446
4	15.32	9406.8	218	0.6633	48.719	0.531

Enantioenriched (94% ee)

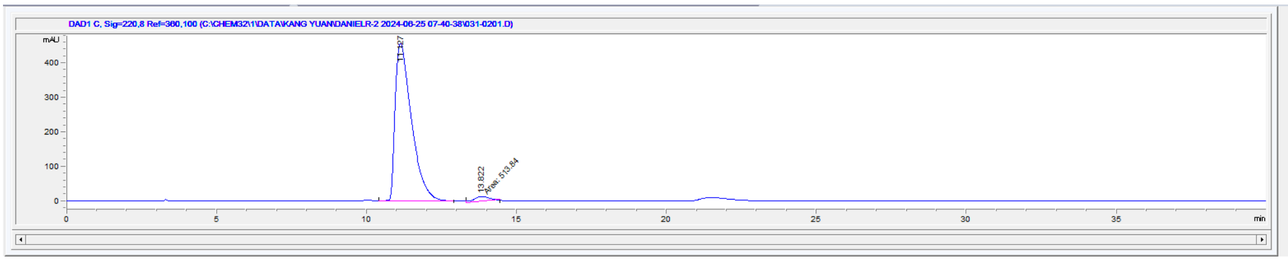

CHIRALPAK® OD, hexane/IPA = 99/1, 1 mL/min

#	Time	Area	Height	Width	Area%	Symmetry
1	11.127	17026.3	457.3	0.5624	97.070	0.44
2	13.822	513.8	14.7	0.5844	2.930	1.04

[back to Table of Contents]

2-(2-methoxyethyl) 1,1-dimethyl (2S,3R)-3-phenyl-3-(propylthio)cyclopropane-1,1,2-tricarboxylate (**3aa**)

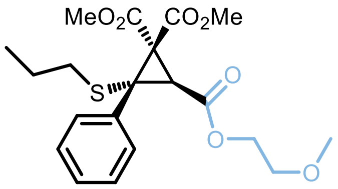

Racemic

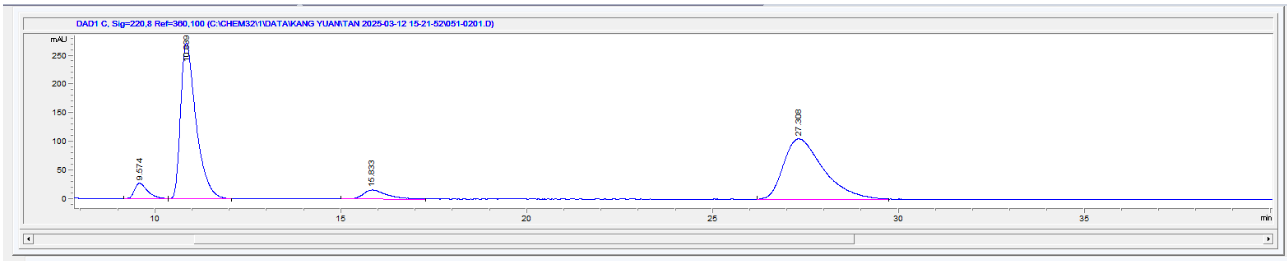

CHIRALPAK® OD, hexane/IPA = 95/5, 1 mL/min

#	Time	Area	Height	Width	Area%	Symmetry
1	9.574	737.2	28.2	0.3873	4.204	0.56
2	10.839	7957.8	273.1	0.4399	45.382	0.548
3	15.833	804.5	16.6	0.603	4.588	0.579
4	27.308	8035.8	106.3	1.0502	45.826	0.569

Enantioenriched (94% ee)

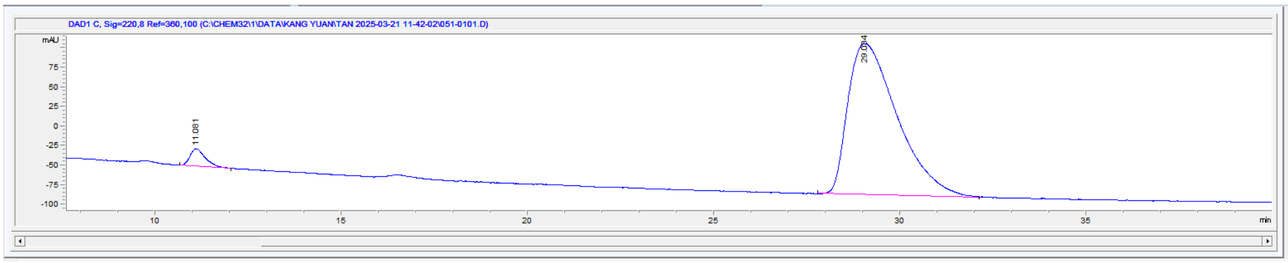

CHIRALPAK® OD, hexane/IPA = 95/5, 1 mL/min

#	Time	Area	Height	Width	Area%	Symmetry
1	11.081	658.8	22.5	0.4249	3.554	0.583
2	29.034	17878.2	193.3	1.1869	96.446	0.458

[back to Table of Contents]

2-benzyl 1,1-dimethyl (2S,3R)-3-phenyl-3-(propylthio)cyclopropane-1,1,2-tricarboxylate (**3ab**)

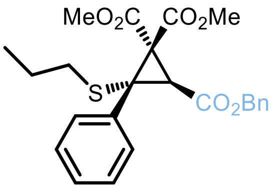

Racemic

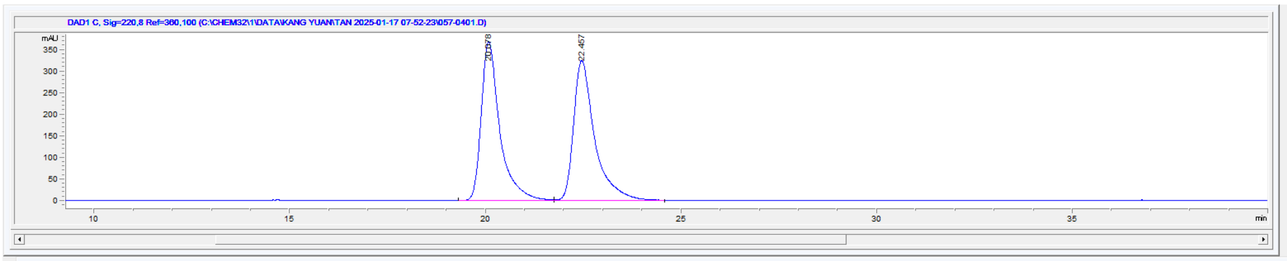

CHIRALPAK® AD-H, hexane/IPA = 98/2, 1 mL/min

#	Time	Area	Height	Width	Area%	Symmetry
1	20.078	12173.3	368.5	0.4914	49.833	0.609
2	22.457	12255.1	324.5	0.5606	50.167	0.568

Enantioenriched (91% ee)

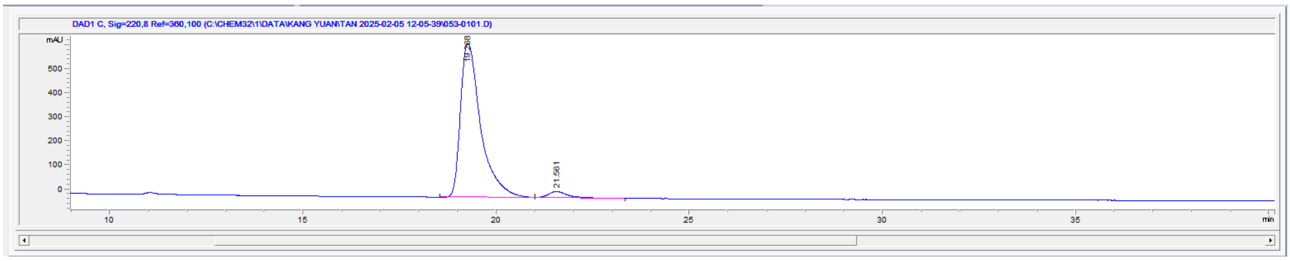

CHIRALPAK® AD-H, hexane/IPA = 98/2, 1 mL/min

#	Time	Area	Height	Width	Area%	Symmetry
1	19.268	23374.8	640.4	0.5519	95.924	0.529
2	21.561	993.3	27.3	0.5238	4.076	0.669

[back to Table of Contents]

2-butyl 1,1-dimethyl (2S,3R)-3-phenyl-3-(propylthio)cyclopropane-1,1,2-tricarboxylate (**3ac**)

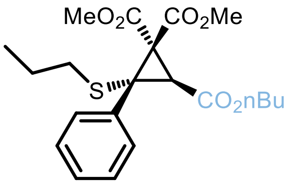

Racemic

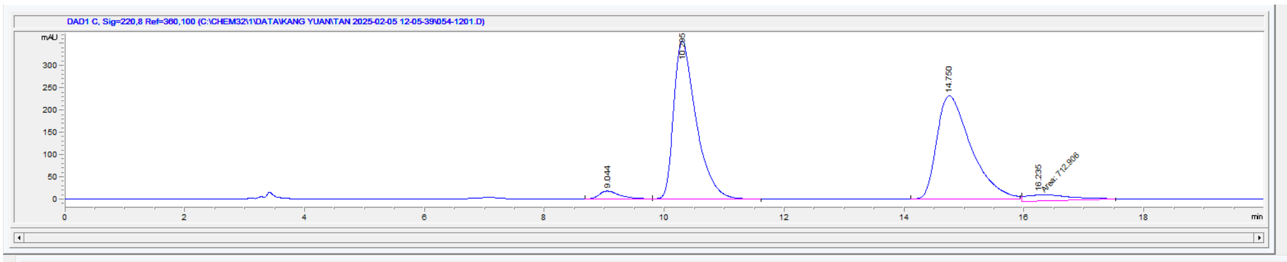

CHIRALPAK® OD, hexane/IPA = 98/2, 1 mL/min

#	Time	Area	Height	Width	Area%	Symmetry
1	9.044	480.3	18.5	0.3724	2.465	0.566
2	10.295	9316.1	356	0.3913	47.809	0.563
3	14.75	9071.8	231.5	0.6531	46.555	0.576
4	16.235	617.8	12.4	0.8311	3.171	0.446

Enantioenriched (93% ee)

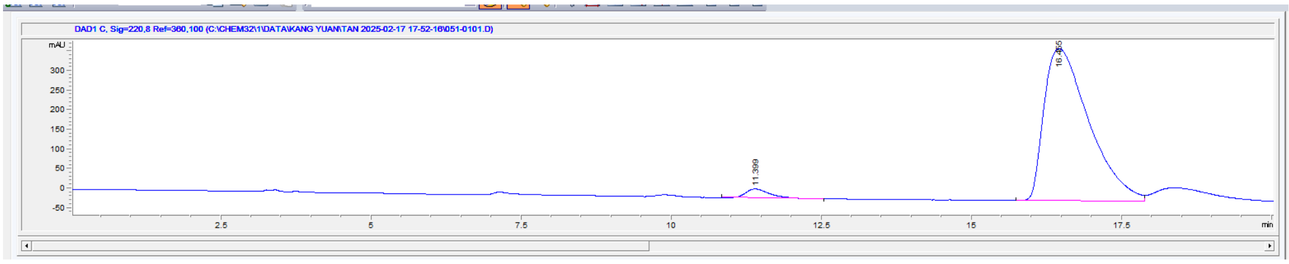

CHIRALPAK® OD, hexane/IPA = 98/2, 1 mL/min

#	Time	Area	Height	Width	Area%	Symmetry
1	11.399	678.5	23.4	0.4084	3.242	0.638
2	16.455	20253.5	389	0.7794	96.758	0.465

[back to Table of Contents]

1,1-dimethyl 2-(prop-2-yn-1-yl) (2S,3R)-3-phenyl-3-(propylthio)cyclopropane-1,1,2-tricarboxylate (**3ad**)

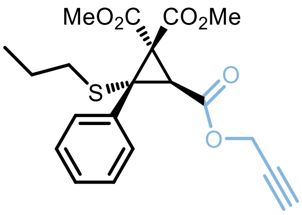

Racemic

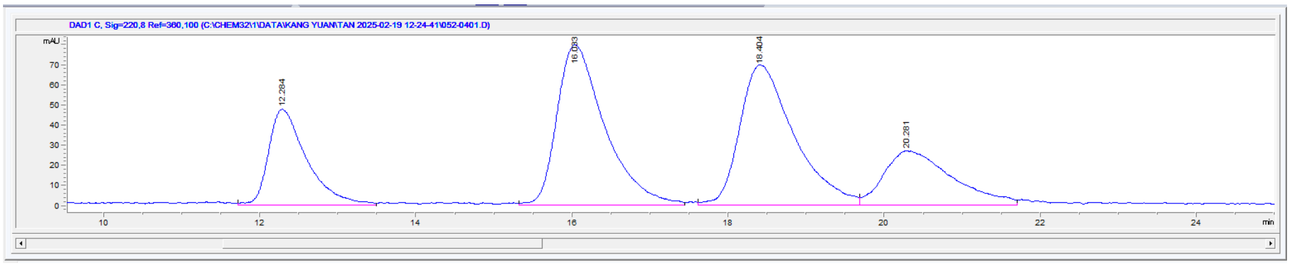

CHIRALPAK® OD, hexane/IPA = 97/3, 1 mL/min

#	Time	Area	Height	Width	Area%	Symmetry
1	12.284	1657.4	47.9	0.506	16.306	0.523
2	16.033	3432.3	80	0.6105	33.767	0.564
3	18.404	3409.2	70	0.656	33.540	0.56
4	20.281	1665.7	27.1	0.752	16.387	0.47

Enantioenriched (94% ee)

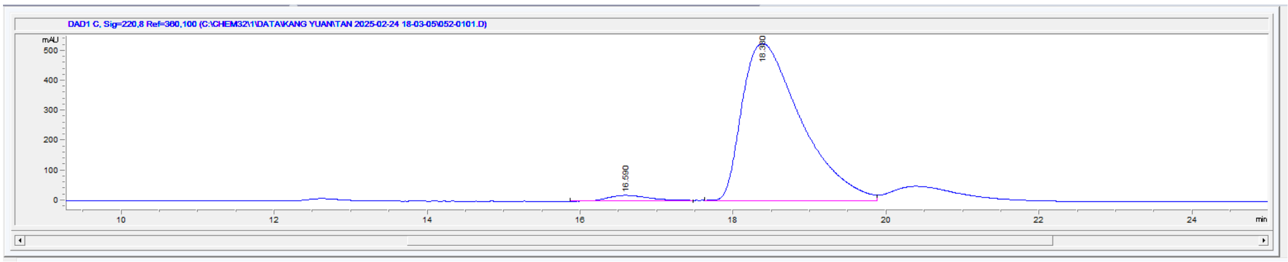

CHIRALPAK® OD, hexane/IPA = 97/3, 1 mL/min

#	Time	Area	Height	Width	Area%	Symmetry
1	16.59	854.5	20.4	0.5517	2.897	0.622
2	18.38	28636.1	528.6	0.7941	97.103	0.494

[back to Table of Contents]

dimethyl (2R,3S)-3-(benzylcarbamoyl)-2-phenyl-2-(propylthio)cyclopropane-1,1-dicarboxylate (**3ae**)

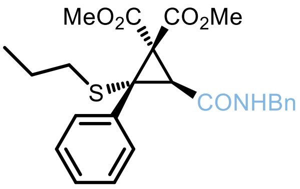

Racemic

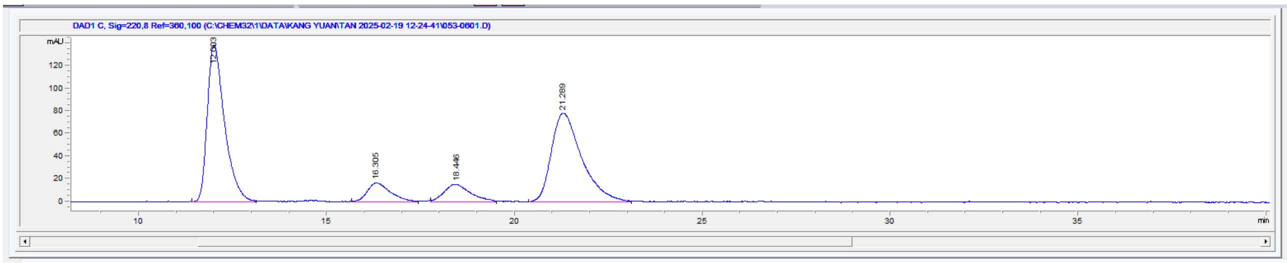

CHIRALPAK® OD, hexane/IPA = 97/3, 1 mL/min

#	Time	Area	Height	Width	Area%	Symmetry
1	12.003	4510.3	139.3	0.4816	42.348	0.593
2	16.305	790.3	17.5	0.5538	7.420	0.555
3	18.446	832	16	0.6374	7.811	0.786
4	21.289	4518.1	79.2	0.7497	42.421	0.58

Enantioenriched (87% ee)

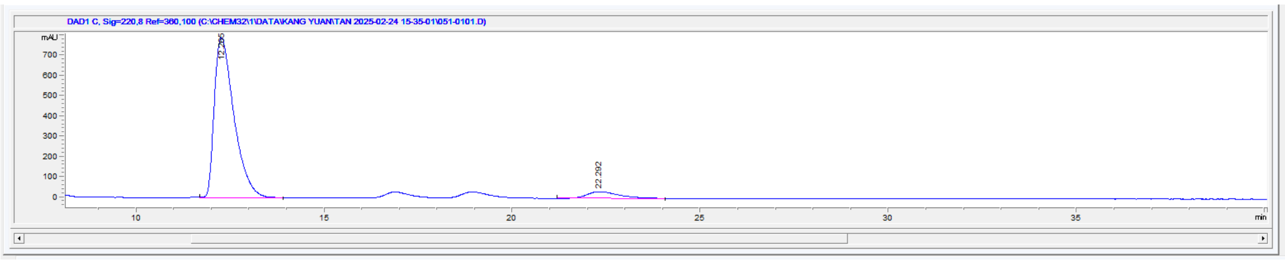

CHIRALPAK® OD, hexane/IPA = 97/3, 1 mL/min

#	Time	Area	Height	Width	Area%	Symmetry
1	12.265	29077.6	792.3	0.5462	93.237	0.525
2	22.292	2109.2	33.8	0.7471	6.763	0.569

[back to Table of Contents]

methyl (1R,5S,6R)-2,4-dioxo-3,6-diphenyl-6-(propylthio)-3-azabicyclo[3.1.0]hexane-1-carboxylate (**3af**)

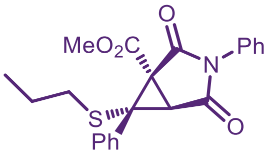

Racemic

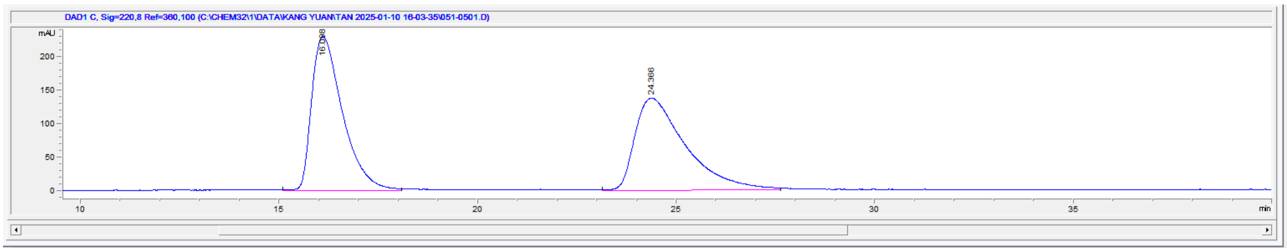

CHIRALPAK® OD, hexane/IPA = 95/5, 1 mL/min

#	Time	Area	Height	Width	Area%	Symmetry
1	16.098	12488	229.9	0.8236	50.210	0.527
2	24.366	12383.5	138	1.1931	49.790	0.435

Enantioenriched (91% ee)

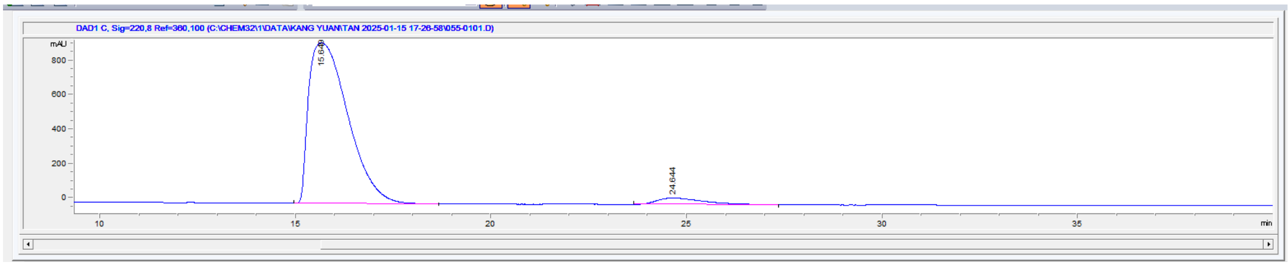

CHIRALPAK® OD, hexane/IPA = 95/5, 1 mL/min

#	Time	Area	Height	Width	Area%	Symmetry
1	15.649	65009.3	934.5	0.9776	95.279	0.447
2	24.644	3221.5	38.2	1.0028	4.721	0.492

[back to Table of Contents]

methyl (1S,5R,6S)-3-cyclohexyl-2,4-dioxo-6-phenyl-6-(propylthio)-3-azabicyclo[3.1.0]hexane-1-carboxylate (3ag)

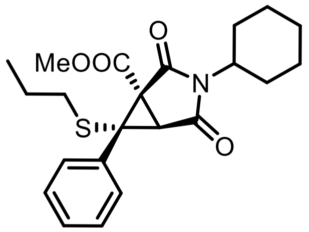

Racemic

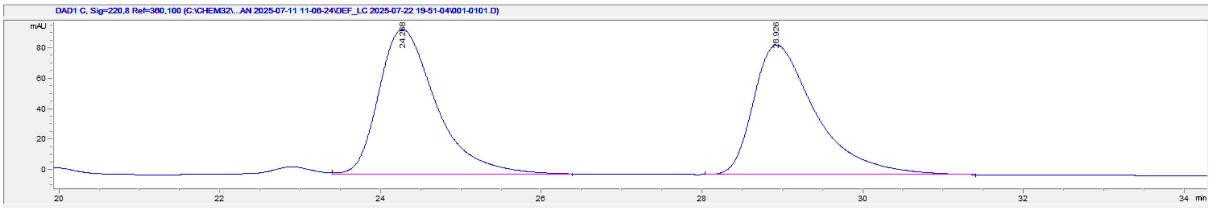

CHIRALPAK® AD-H, hexane/IPA = 99/1, 1 mL/min

#	Time	Area	Height	Width	Area%	Symmetry
1	24.268	4736.9	95.2	0.7642	50.688	0.65
2	28.926	4608.3	85.2	0.8148	49.312	0.547

Enantioenriched (93% ee)

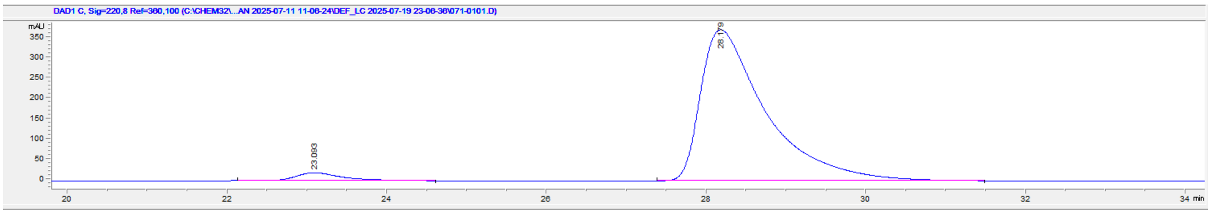

CHIRALPAK® AD-H, hexane/IPA = 99/1, 1 mL/min

#	Time	Area	Height	Width	Area%	Symmetry
1	23.093	805.9	19.5	0.6195	3.671	0.612
2	28.179	21146.2	372.3	0.8306	96.329	0.402

[*back to Table of Contents*]

methyl (5R,6S)-3-(4-methoxyphenyl)-2,4-dioxo-6-phenyl-6-(propylthio)-3-azabicyclo[3.1.0]hexane-1-carboxylate (3ah)

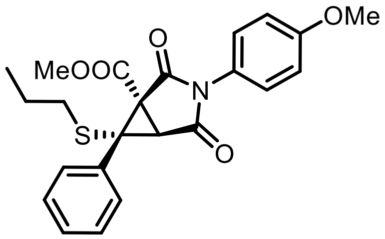

Racemic

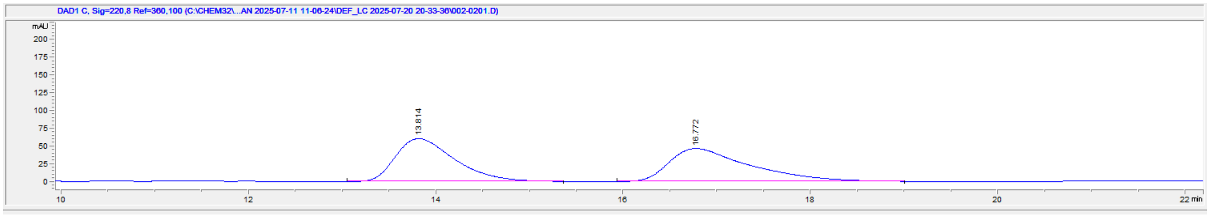

#	Time	Area	Height	Width	Area%	Symmetry
1	13.814	2665	60.5	0.6743	48.863	0.598
2	16.772	2789.1	46.4	0.8688	51.137	0.458

CHIRALCEL® OD, hexane/IPA = 90/10, 1 mL/min

Enantioenriched (90% ee)

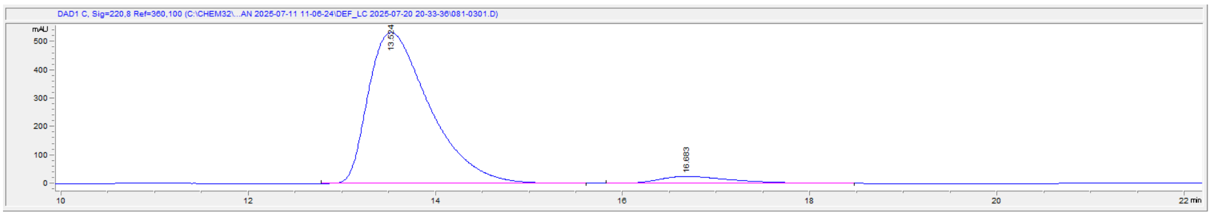

#	Time	Area	Height	Width	Area%	Symmetry
1	13.524	24214.9	532.9	0.6981	94.702	0.543
2	16.683	1354.6	24.7	0.8025	5.298	0.571

[back to Table of Contents]

trimethyl (2S,3R)-3-phenyl-3-(propylsulfonyl)cyclopropane-1,1,2-tricarboxylate(4a)

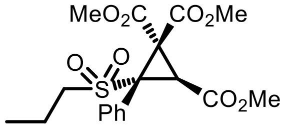

Racemic

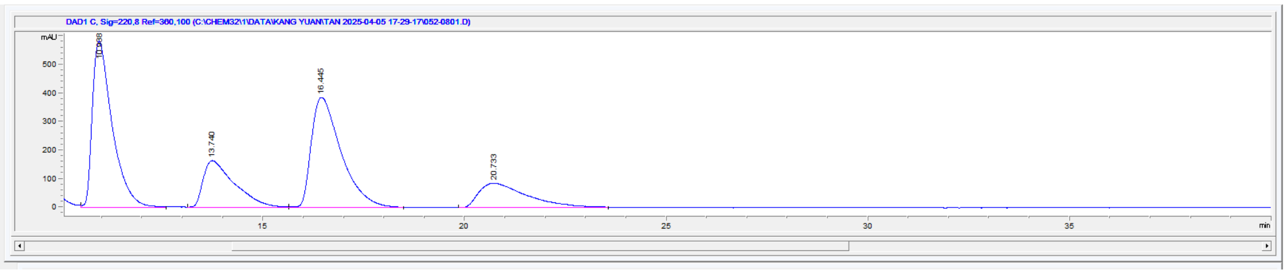

CHIRALPAK® OD, hexane/IPA = 90/10, 1 mL/min

#	Time	Area	Height	Width	Area%	Symmetry
1	10.938	20087.7	583.1	0.5199	35.685	0.472
2	13.74	9008.2	165.1	0.7525	16.003	0.379
3	16.445	19922.5	386.7	0.7611	35.392	0.475
4	20.733	7272.8	85.8	1.0068	12.920	0.444

Enantioenriched (93% ee)

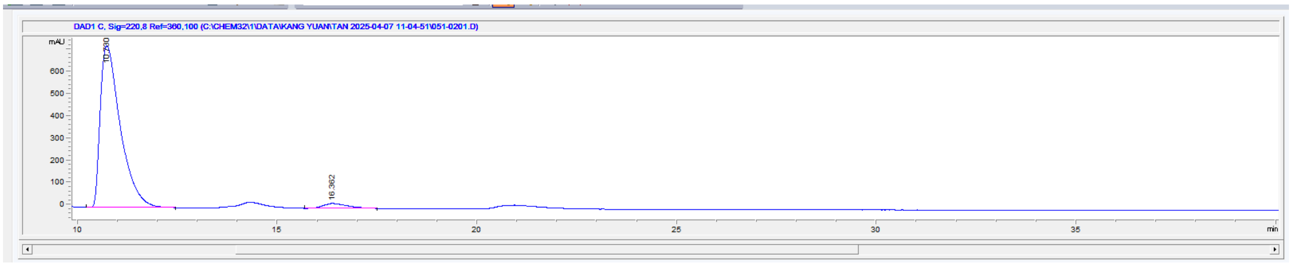

CHIRALPAK® OD, hexane/IPA = 90/10, 1 mL/min

#	Time	Area	Height	Width	Area%	Symmetry
1	10.73	25535.9	728.9	0.5247	96.167	0.447
2	16.362	1017.8	22.6	0.5408	3.833	0.505

[back to Table of Contents]

trimethyl (2S,3R)-3-phenyl-3-((R)-propylsulfinyl)cyclopropane-1,1,2-tricarboxylate (4b)

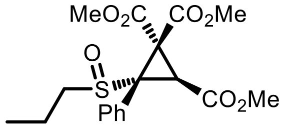

Racemic

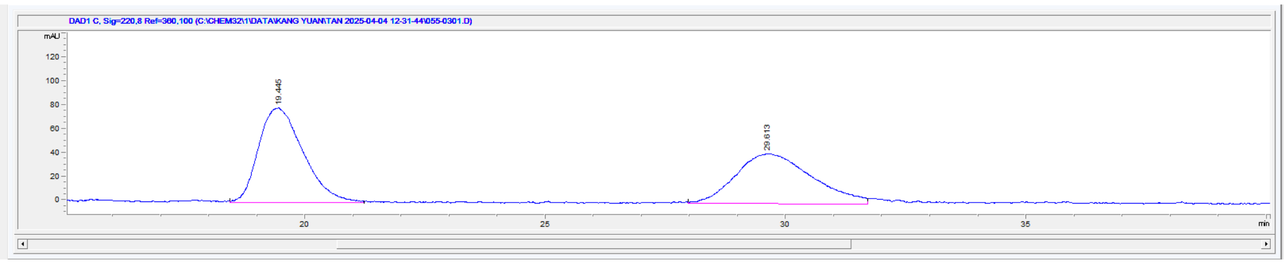

CHIRALPAK® AS-H, hexane/IPA = 90/10, 1 mL/min

#	Time	Area	Height	Width	Area%	Symmetry
1	19.445	5192.2	79.7	0.774	52.588	0.766
2	29.613	4681	42.3	1.3079	47.412	0.702

Enantioenriched (93% ee)

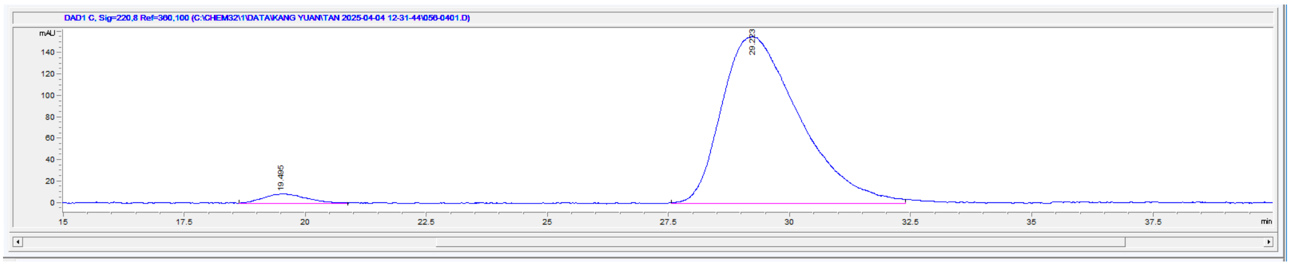

CHIRALPAK® OD, hexane/IPA = 90/10, 1 mL/min

#	Time	Area	Height	Width	Area%	Symmetry
1	19.495	605.7	9.3	0.7768	3.344	0.682
2	29.223	17508.1	156.4	1.3321	96.656	0.618

[back to Table of Contents]

trimethyl (2S,3R)-3-phenyl-3-((R)-propylsulfinyl)cyclopropane-1,1,2-tricarboxylate (4b')

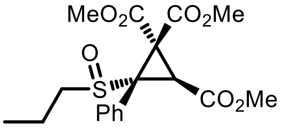

Racemic

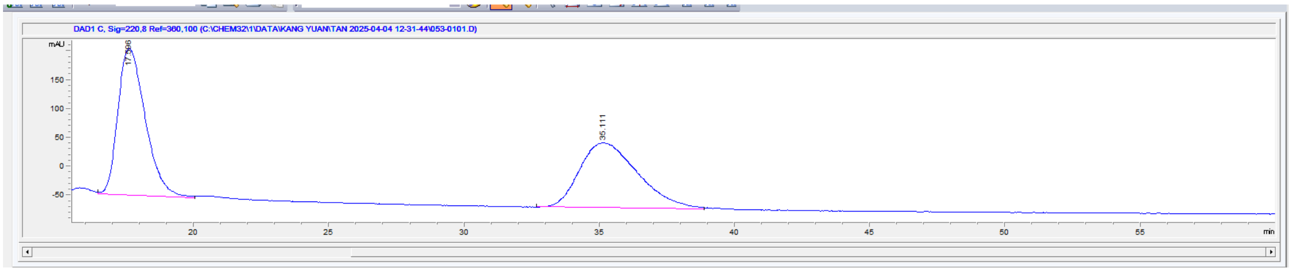

CHIRALPAK® AS-H, hexane/IPA = 90/10, 1 mL/min

#	Time	Area	Height	Width	Area%	Symmetry
1	17.596	17887.5	254.5	0.986	51.172	0.638
2	35.111	17068	112.9	1.7764	48.828	0.639

Enantioenriched (85% ee)

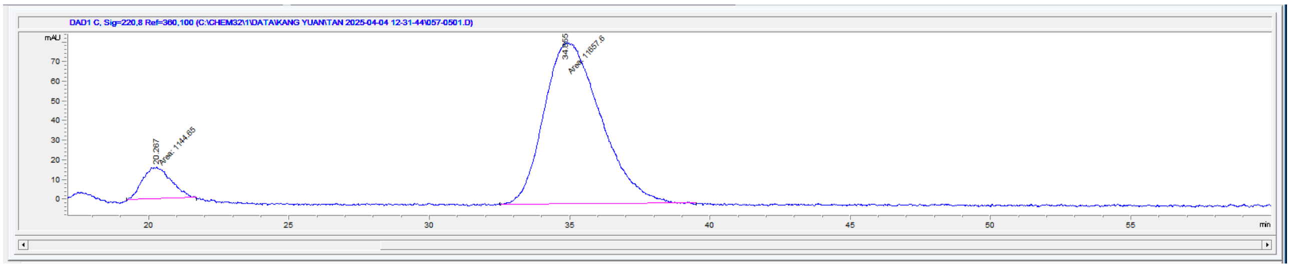

CHIRALPAK® OD, hexane/IPA = 90/10, 1 mL/min

#	Time	Area	Height	Width	Area%	Symmetry
1	20.267	1144.6	16.1	1.1885	8.941	0.905
2	34.855	11657.6	82.8	2.3456	91.059	0.628

[back to Table of Contents]

trimethyl (2S)-3-phenylcyclopropane-1,1,2-tricarboxylate (4c)

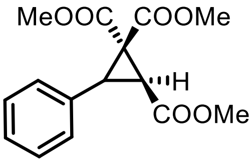

Racemic

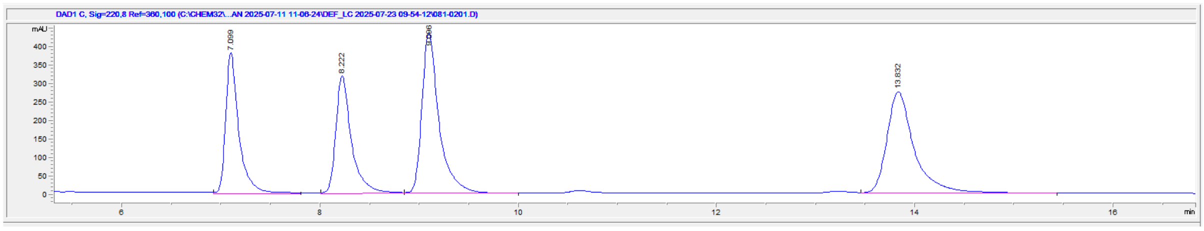

#	Time	Area	Height	Width	Area%	Symmetry
1	7.099	3581.2	381	0.1377	20.289	0.581
2	8.222	3530.1	318.2	0.1634	20.000	0.61
3	9.096	5245.8	434	0.179	29.720	0.618
4	13.832	5293.7	274	0.2874	29.991	0.638

CHIRALPAK® AD-H, hexane/IPA = 90/10, 1 mL/min

Enantioenriced (93% ee)

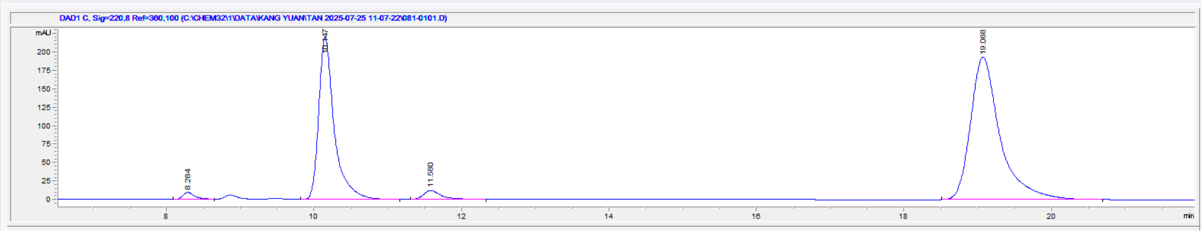

#	Time	Area	Height	Width	Area%	Symmetry
1	8.284	114.7	9.7	0.1766	1.287	0.616
2	10.147	3224.8	220.3	0.2188	36.165	0.627
3	11.58	208.2	12.2	0.2504	2.335	0.623
4	19.068	5369.1	192.9	0.4128	60.213	0.659

[back to Table of Contents]

trimethyl 2-(4-methoxyphenyl)-5-phenylfuran-3,3,4(2H)-tricarboxylate (4d)

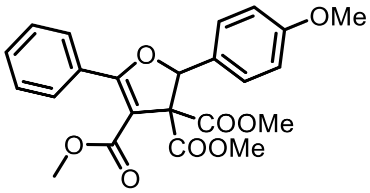

Racemic

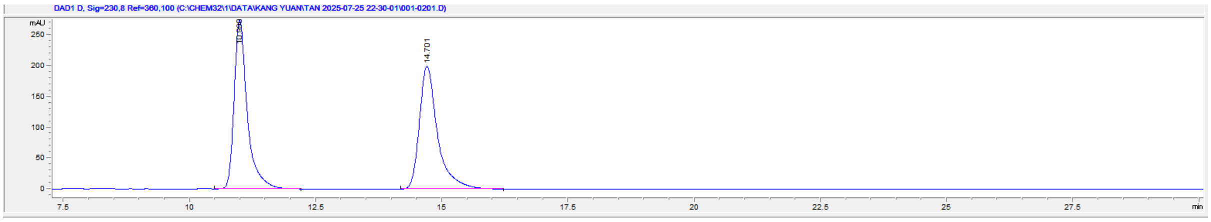

#	Time	Area	Height	Width	Area%	Symmetry
1	10.99	4970.2	273.9	0.2697	49.987	0.655
2	14.701	4972.9	199.3	0.3712	50.013	0.654

CHIRALPAK® AD-H, hexane/IPA = 80/20, 1 mL/min

Enantioenriced (racemised)

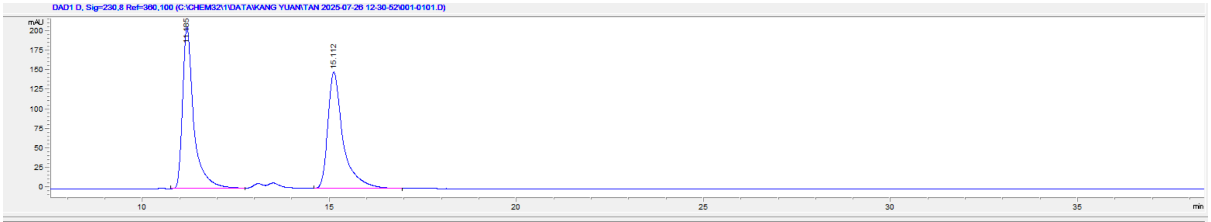

#	Time	Area	Height	Width	Area%	Symmetry
1	11.185	4334.1	207.9	0.3052	50.591	0.567
2	15.112	4232.8	149.3	0.4187	49.409	0.587

[back to Table of Contents]

References

(1) Rozsar, D.; Formica, M.; Yamazaki, K.; Hamlin, T. A.; Dixon, D. J. Bifunctional Iminophosphorane-Catalyzed Enantioselective Sulfa-Michael Addition to Unactivated α,β-Unsaturated Amides. *Journal of the American Chemical Society* **2022**, *144* (2), 1006–1015. DOI: 10.1021/jacs.1c11898.

(2) Poh, C. Y. X.; Rozsar, D.; Yang, J.; Christensen, K. E.; Dixon, D. J. Bifunctional Iminophosphorane Catalyzed Amide Enolization for Enantioselective Cyclohexadienone Desymmetrization. *Angewandte Chemie International Edition* **2024**, *63* (5), e202315401. DOI: https://doi.org/10.1002/anie.202315401.

(3) de Nanteuil, F.; Waser, J. Catalytic [3+2] Annulation of Aminocyclopropanes for the Enantiospecific Synthesis of Cyclopentylamines. *Angewandte Chemie International Edition* **2011**, *50* (50), 12075–12079. DOI: https://doi.org/10.1002/anie.201106255.

(4) Zhang, F.; Fox, J. M. Synthesis of Cyclopropene α-Amino Acids via Enantioselective Desymmetrization. *Organic Letters* **2006**, *8* (14), 2965–2968. DOI: 10.1021/ol060847l.

(5) Isobe, H.; Sato, S.; Tanaka, T.; Tokuyama, H.; Nakamura, E. Thermal and Palladium-Catalyzed [3 + 2] Synthesis of Cyclopentadienone Acetals from Cyclopropenone Acetals and Acetylenes. *Organic Letters* **2004**, *6* (20), 3569–3571. DOI: 10.1021/ol0483450.

(6) Cassagne, T.; Cristau, H.-J.; Delmas, G.; Desgranges, M.; Lion, C.; Magnaud, G.; Torreilles, É.; Virieux, D. Comparative Evaluation of Oxidising and Nucleophilic Properties of Some α-Nucleophiles†. *Journal of Chemical Research* **2002**, *2002* 336–338. DOI: 10.3184/030823402103172194

(7) Jiang, Z.; Niu, S.-L.; Zeng, Q.; Ouyang, Q.; Chen, Y.-C.; Xiao, Q. Selective Alkynylallylation of the C−C σ Bond of Cyclopropenes. *Angewandte Chemie International Edition* **2021**, *60* (1), 297–303. DOI: https://doi.org/10.1002/anie.202008886.

(8) Chuprakov, S.; Rubin, M.; Gevorgyan, V. Direct Palladium-Catalyzed Arylation of Cyclopropenes. *Journal of the American Chemical Society* **2005**, *127* (11), 3714–3715. DOI: 10.1021/ja042380k.

(9) Li, T.; Julaiti, Y.; Wu, X.; Han, J.; Xie, J. Gold-Catalyzed Divergent Ring-Opening Rearrangement of Cyclopropenes Enabled by Dichotomous Gold−Carbenes. *Chemistry – A European Journal* **2022**, *28* (71), e202202851. DOI: https://doi.org/10.1002/chem.202202851.

(10) Ye, Q.; Ye, H.; Cheng, D.; Li, X.; Xu, X. Regioselective oxidative ring-opening of cyclopropenyl carboxylates by visible light photoredox catalysis. *Tetrahedron Letters* **2018**, *59* (26), 2546–2549. DOI: https://doi.org/10.1016/j.tetlet.2018.05.047.

(11) Song, C.; Ju, L.; Wang, M.; Liu, P.; Zhang, Y.; Wang, J.; Xu, Z. From Cyclopropenes to Tetrasubstituted Furans: Tandem Isomerization/Alkenylation Sequence with Cu/Pd Relay Catalysis. *Chemistry – A European Journal* **2013**, *19* (11), 3584–3589. DOI: https://doi.org/10.1002/chem.201203997 (accessed 2025/04/07).

(12) Biswas, S.; Mallick, M.; K S, G. N.; Chandu, P.; Sureshkumar, D. A Visible Light Photoredox Approach for Synthesizing Sulfone-Functionalized Cyclopropenes. *Organic Letters* **2024**, *26* (48), 10207–10212. DOI: 10.1021/acs.orglett.4c03278.

(13) Peng, C.; Gu, F.; Lin, X.; Ding, N.; Zhan, Q.; Cao, P.; Cao, T. Highly selective catalyst- and additive-free iodosulfonylation of cyclopropenes in water. *Green Chemistry* **2023**, *25* (2), 671–677, 10.1039/D2GC04296G. DOI: 10.1039/D2GC04296G.

(14) Mallick, M.; Pal, K.; Das, D.; Biswas, S.; Das, S.; Sureshkumar, D. Visible Light-Induced Photocatalyst-Free Diastereoselective Iodosulfonylation of Cyclopropenes in Water. *The Journal of Organic Chemistry* **2024**, *89* (24), 18218–18226. DOI: 10.1021/acs.joc.4c02076.

(15) Kempson, J.; Pitts, W. J.; Barbosa, J.; Guo, J.; Omotoso, O.; Watson, A.; Stebbins, K.; Starling, G. C.; Dodd, J. H.; Barrish, J. C.; et al. Fused pyrimidine based inhibitors of phosphodiesterase 7 (PDE7): synthesis and initial structure–activity relationships. *Bioorganic & Medicinal Chemistry Letters* **2005**, *15* (7), 1829–1833. DOI: https://doi.org/10.1016/j.bmcl.2005.02.025.

(16) Liu, L.-Q.; Hong, P.-X.; Song, X.-H.; Zhou, C.-C.; Ling, R.; Kang, Y.; Qi, Q.-R.; Yang, J. Design, Synthesis, and Activity Study of Water-Soluble, Rapid-Release Propofol Prodrugs. *Journal of Medicinal Chemistry* **2020**, *63* (14), 7857–7866. DOI: 10.1021/acs.jmedchem.0c00698.

(17) Wang, Y.; Fordyce, E. A. F.; Chen, F. Y.; Lam, H. W. Stereoselective Synthesis of Tri- and Tetrasubstituted Alkenes by Iron-Catalyzed Carbometalation Ring-Opening Reactions of Cyclopropenes. *Angewandte Chemie International Edition* **2008**, *47* (38), 7350–7353. DOI: https://doi.org/10.1002/anie.200802391.

(18) Yasukawa, T.; Gilles, P.; Martin, J.; Boutet, J.; Cossy, J. Biocatalytic Enantioselective Reduction of Cyclopropenyl Esters and Ketones Using Ene-Reductases. *ACS Catalysis* **2024**, *14* (8), 6188–6193. DOI: 10.1021/acscatal.4c00899.

(19) Del Fiandra, C.; Piras, L.; Fini, F.; Disetti P.; Moccia, M.; Adamo, M.F.A. Phase transfer catalyzed enantioselective cyclopropanation of 4-nitro-5-styrylisoxazoles. Chemical Communications **2012**, *48* (32), 3863-3865. DOI: 10.1039/c2cc30401e

(20) Sanders, S. D.; Ruiz-Olalla, A.; Johnson, J. S. *Chem. Commun.* **2009**, 5135-5137
